# Supplementary material for: Neuronal parts list and wiring diagram for a visual system
Source: Nature. 2024 Oct 2;634(8032):166–80. doi: 10.1038/s41586-024-07981-1 (PMC11446827; doi:10.1038/s41586-024-07981-1)
Supplement: Supplementary file 1 — EyeWire consortium list with usernames. [file 41586_2024_7981_MOESM1_ESM.docx]

Supplementary Note

Eyewire consortium list with Usernames

Krzysztof Kruk^3,7^, Celia David^1,7^, Anne Kristiansen^7^, Thomas Stocks^7^, Jaime Skelton (AzureJay)^7^, Travis R. Aiken (TR77)^7^, Marissa Sorek^1,7^, Nikitas Serafetinidis (Nseraf)^7^, Amy R. Sterling^1,7^

^1^Princeton Neuroscience Institute, Princeton University, Princeton, NJ, USA

^3^ul. Grunwaldzka 22/91, 25-736, Kielce, Poland

^7^Eyewire, Boston, MA, USA
