# Supplementary figures and images for: Neuronal parts list and wiring diagram for a visual system (part 1 of 3)
Source: Nature. 2024 Oct 2;634(8032):166–80. doi: 10.1038/s41586-024-07981-1 (PMC11446827; doi:10.1038/s41586-024-07981-1)

Centrifugal Predicates

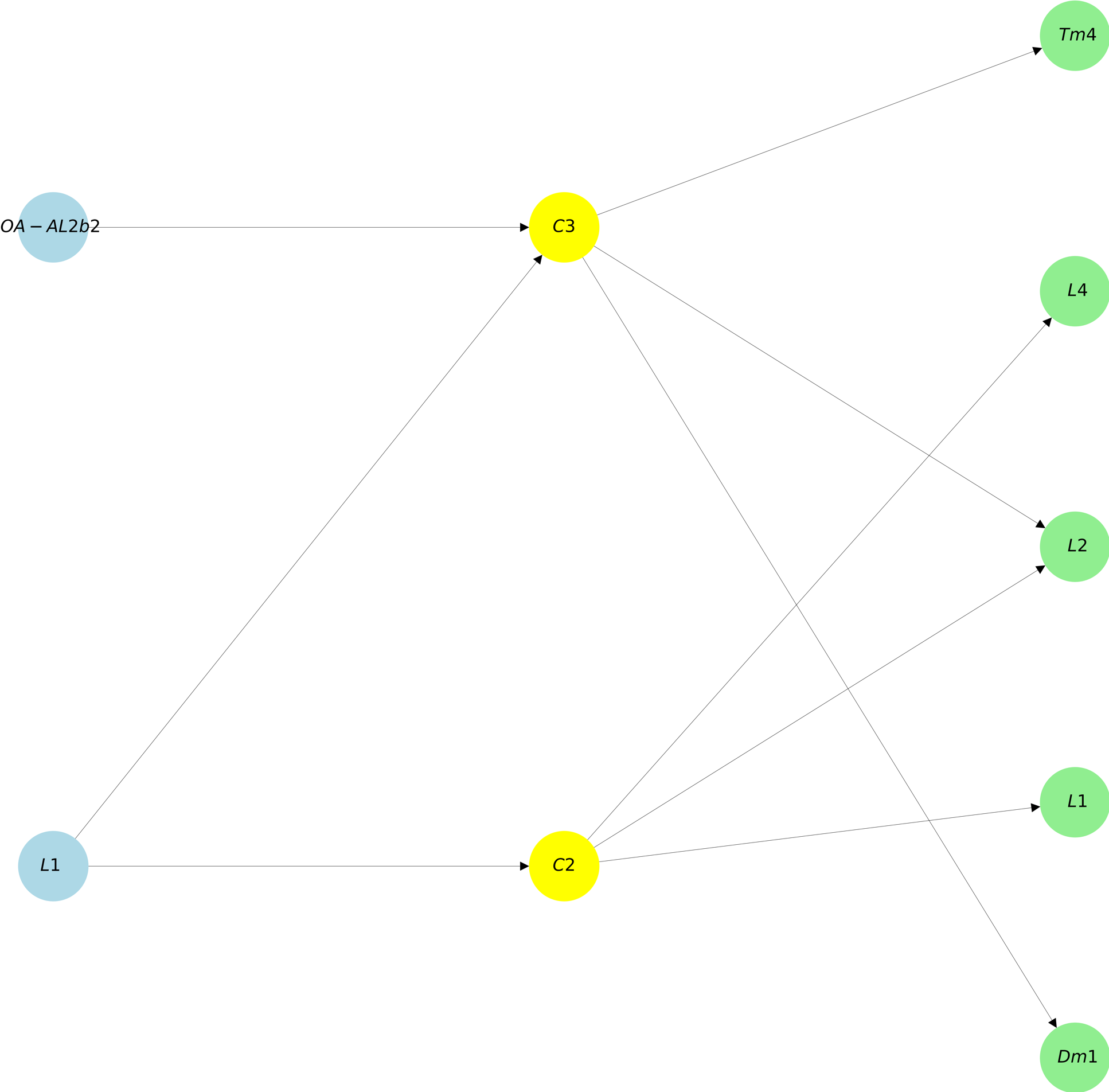

Supplement: Supplementary file 6 — Discriminating logical predicates for all types. Each figure contains types from the same family (middle layer) with shared input attributes (left layer) and output attributes (right layer) that are sufficient for discriminating all types in the middle layer. Families with many types are split into multiple figures for clarity of presentation. [file 41586_2024_7981_MOESM6_ESM.zip › DataS2/pdf/Centrifugal_Predicates.pdf]

## Distal Medulla Dorsal Rim Area Predicates

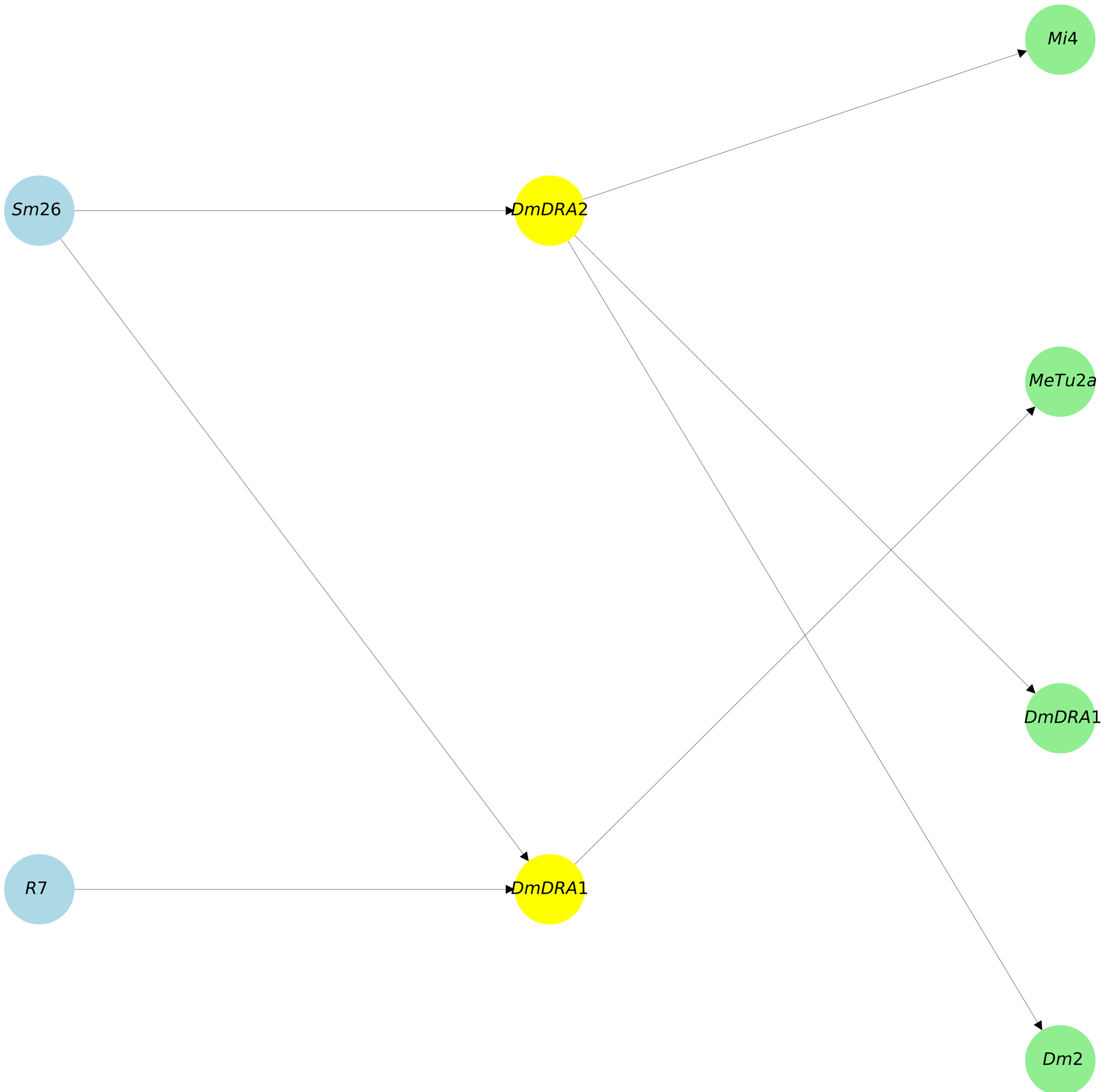

Supplement: Supplementary file 6 — Discriminating logical predicates for all types. Each figure contains types from the same family (middle layer) with shared input attributes (left layer) and output attributes (right layer) that are sufficient for discriminating all types in the middle layer. Families with many types are split into multiple figures for clarity of presentation. [file 41586_2024_7981_MOESM6_ESM.zip › DataS2/pdf/Distal_Medulla_Dorsal_Rim_Area_Predicates.pdf]

## Distal Medulla Predicates (part 1 of 5)

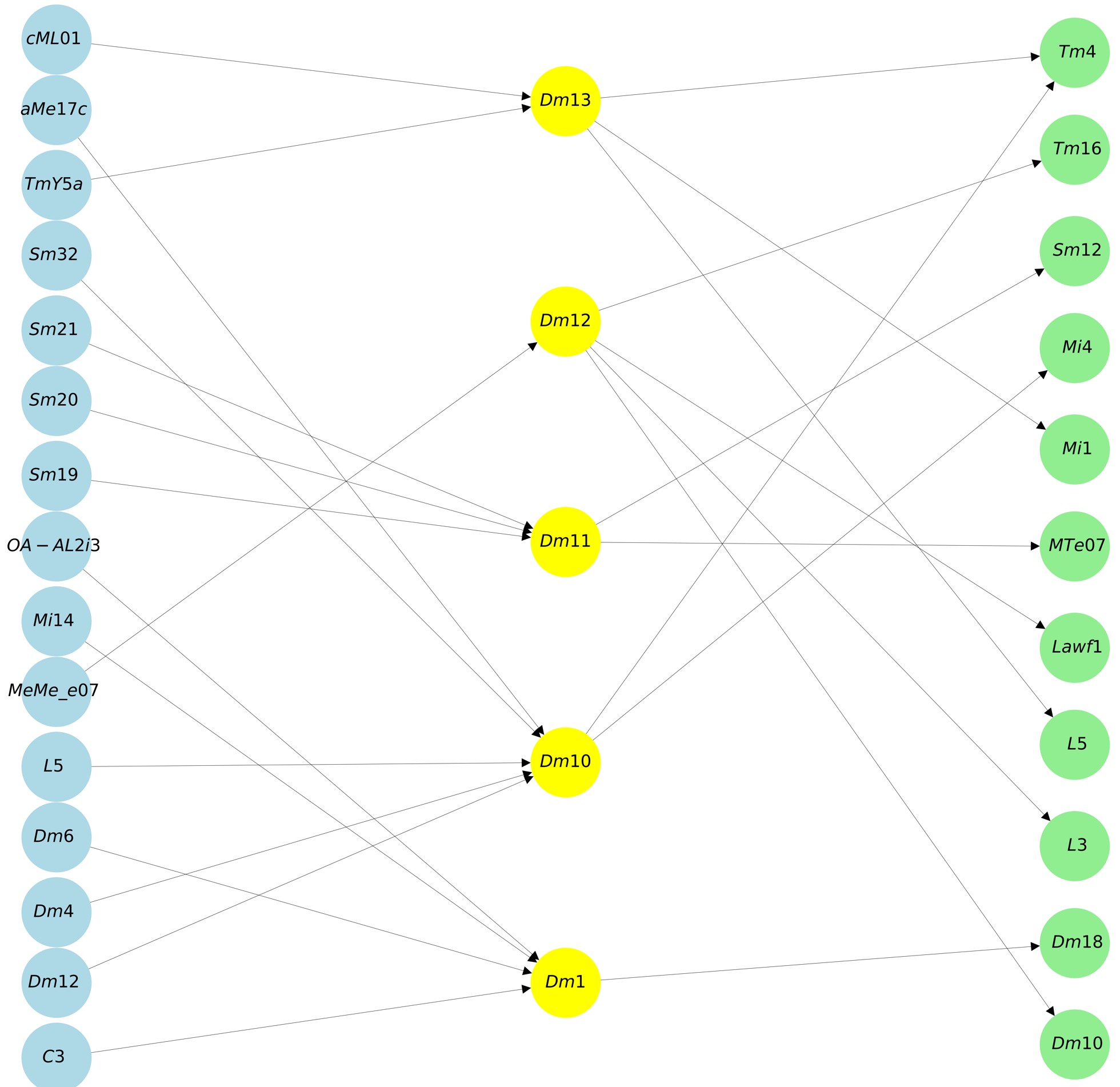

Supplement: Supplementary file 6 — Discriminating logical predicates for all types. Each figure contains types from the same family (middle layer) with shared input attributes (left layer) and output attributes (right layer) that are sufficient for discriminating all types in the middle layer. Families with many types are split into multiple figures for clarity of presentation. [file 41586_2024_7981_MOESM6_ESM.zip › DataS2/pdf/Distal_Medulla_Predicates_(part_1_of_5).pdf]

## Distal Medulla Predicates (part 2 of 5)

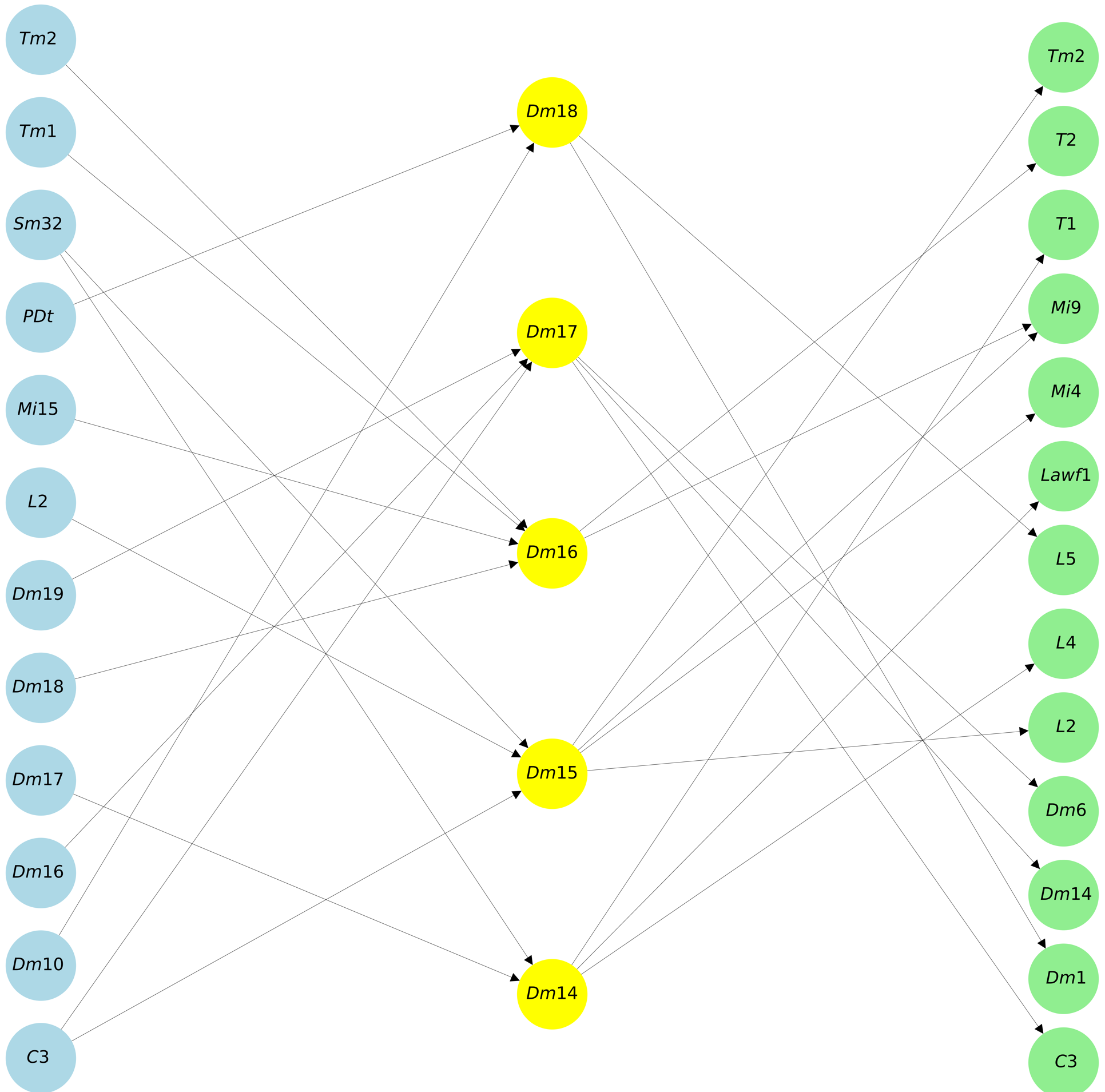

Supplement: Supplementary file 6 — Discriminating logical predicates for all types. Each figure contains types from the same family (middle layer) with shared input attributes (left layer) and output attributes (right layer) that are sufficient for discriminating all types in the middle layer. Families with many types are split into multiple figures for clarity of presentation. [file 41586_2024_7981_MOESM6_ESM.zip › DataS2/pdf/Distal_Medulla_Predicates_(part_2_of_5).pdf]

## Distal Medulla Predicates (part 3 of 5)

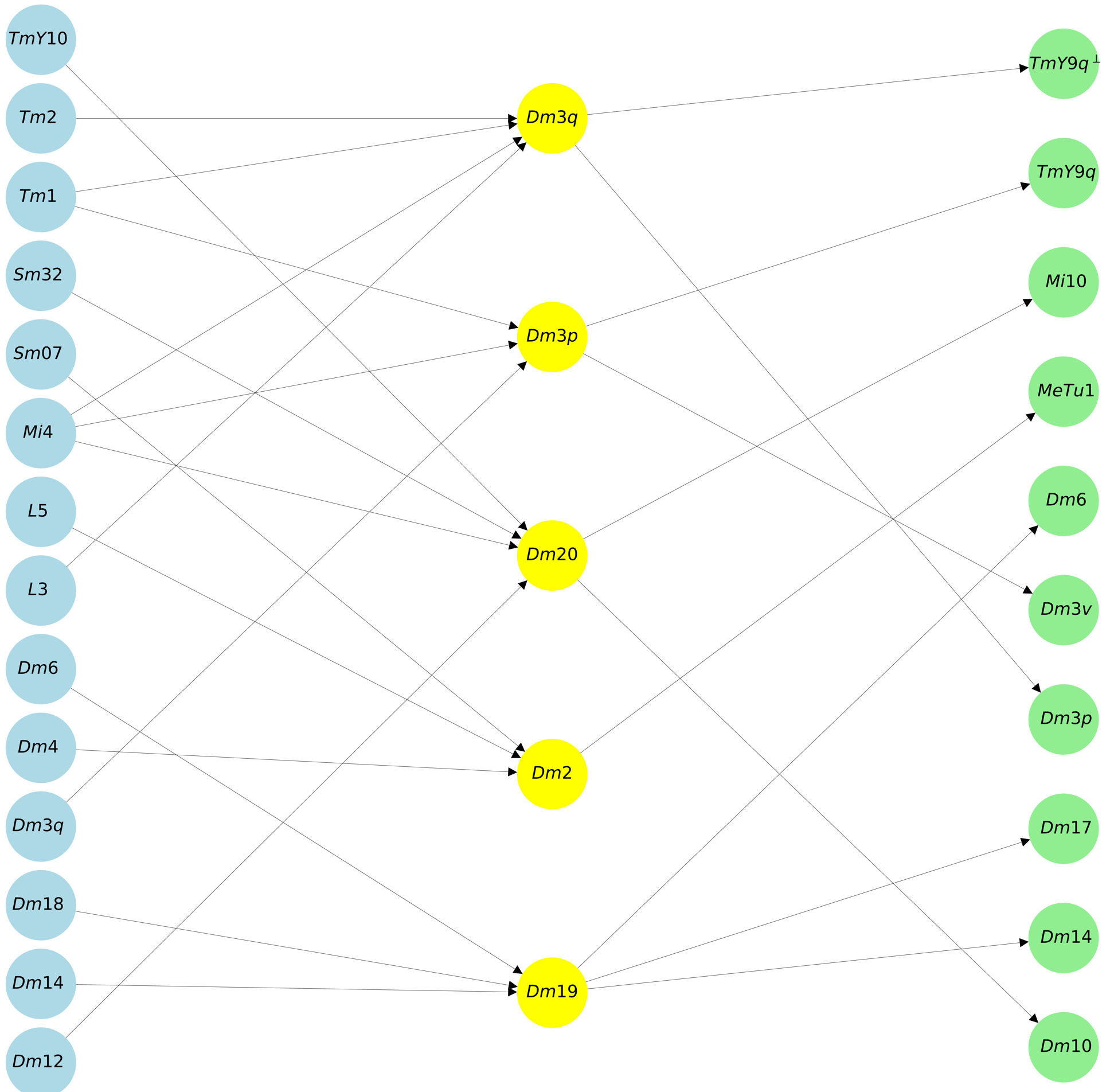

Supplement: Supplementary file 6 — Discriminating logical predicates for all types. Each figure contains types from the same family (middle layer) with shared input attributes (left layer) and output attributes (right layer) that are sufficient for discriminating all types in the middle layer. Families with many types are split into multiple figures for clarity of presentation. [file 41586_2024_7981_MOESM6_ESM.zip › DataS2/pdf/Distal_Medulla_Predicates_(part_3_of_5).pdf]

## Distal Medulla Predicates (part 4 of 5)

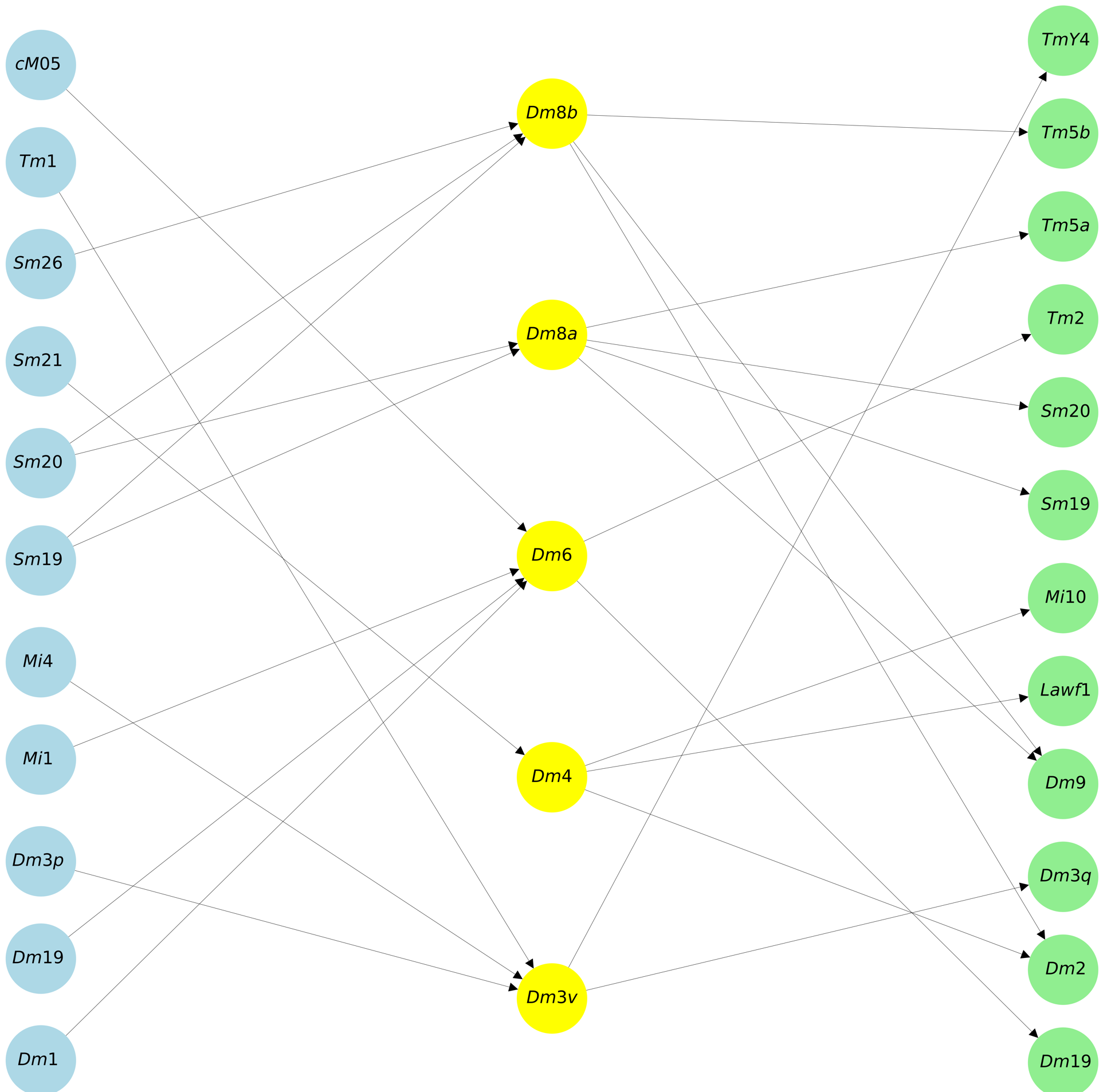

Supplement: Supplementary file 6 — Discriminating logical predicates for all types. Each figure contains types from the same family (middle layer) with shared input attributes (left layer) and output attributes (right layer) that are sufficient for discriminating all types in the middle layer. Families with many types are split into multiple figures for clarity of presentation. [file 41586_2024_7981_MOESM6_ESM.zip › DataS2/pdf/Distal_Medulla_Predicates_(part_4_of_5).pdf]

## Distal Medulla Predicates (part 5 of 5)

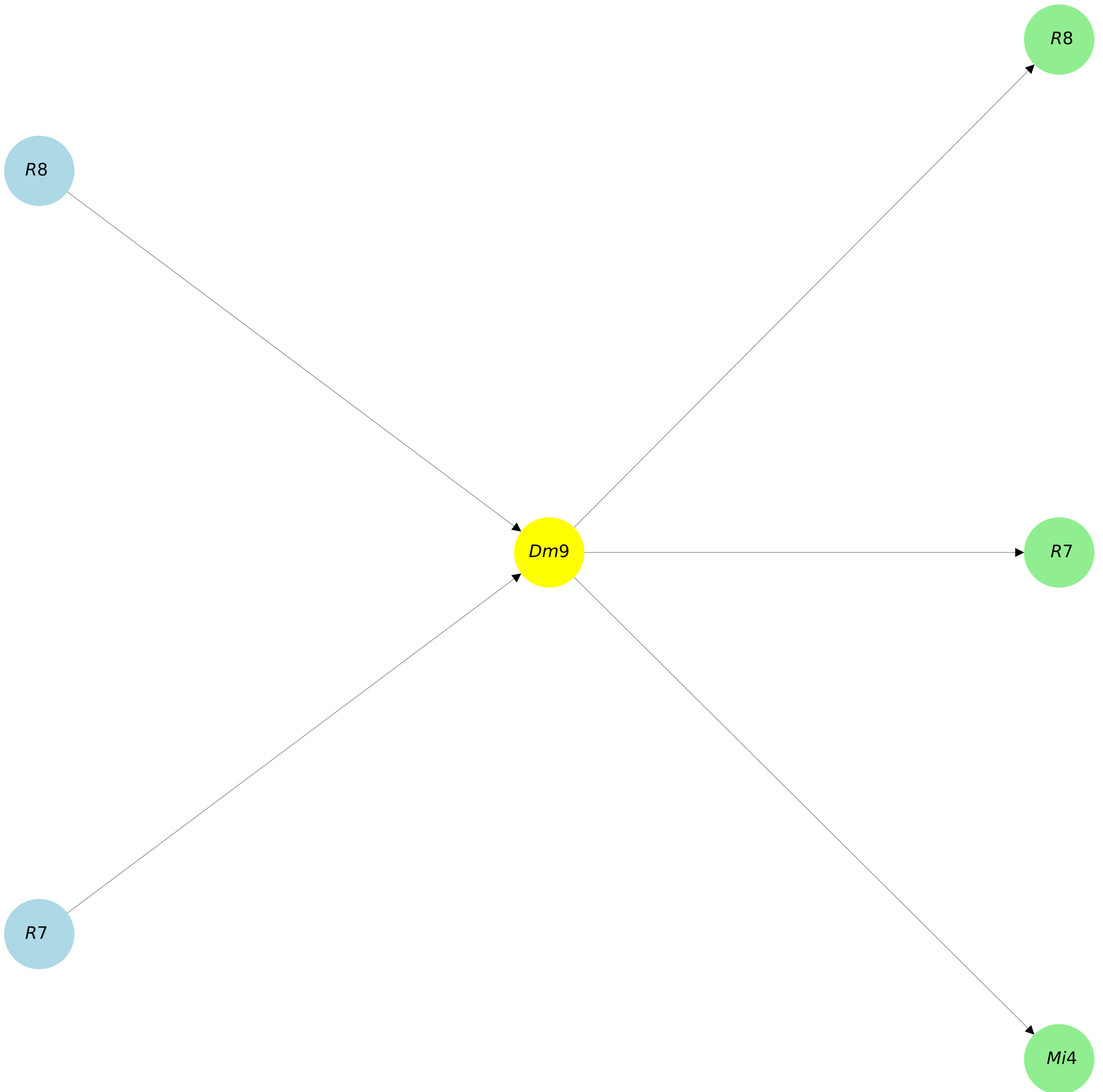

Supplement: Supplementary file 6 — Discriminating logical predicates for all types. Each figure contains types from the same family (middle layer) with shared input attributes (left layer) and output attributes (right layer) that are sufficient for discriminating all types in the middle layer. Families with many types are split into multiple figures for clarity of presentation. [file 41586_2024_7981_MOESM6_ESM.zip › DataS2/pdf/Distal_Medulla_Predicates_(part_5_of_5).pdf]

# Lamina Intrinsic Predicates

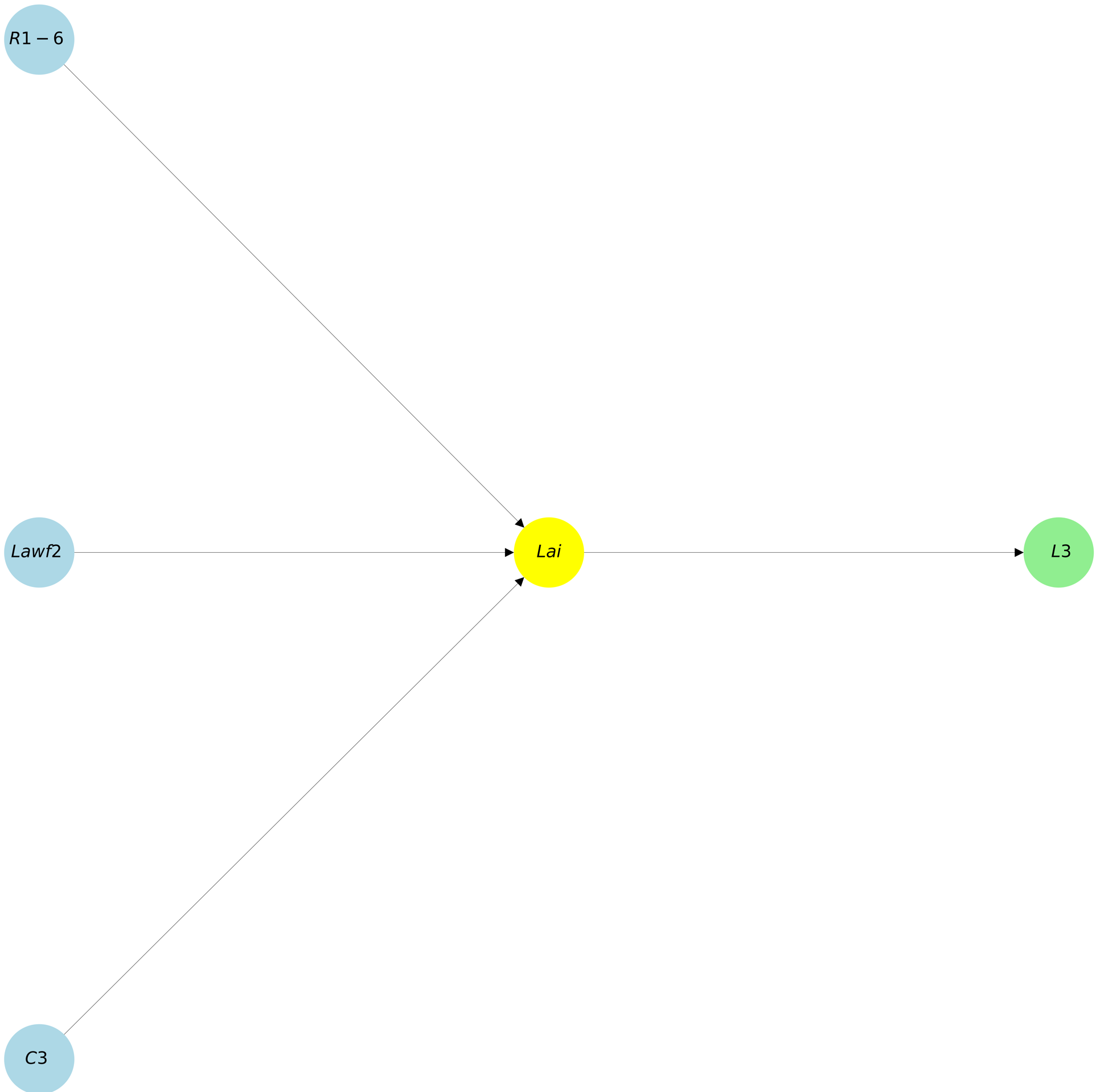

Supplement: Supplementary file 6 — Discriminating logical predicates for all types. Each figure contains types from the same family (middle layer) with shared input attributes (left layer) and output attributes (right layer) that are sufficient for discriminating all types in the middle layer. Families with many types are split into multiple figures for clarity of presentation. [file 41586_2024_7981_MOESM6_ESM.zip › DataS2/pdf/Lamina_Intrinsic_Predicates.pdf]

Lamina Monopolar Predicates

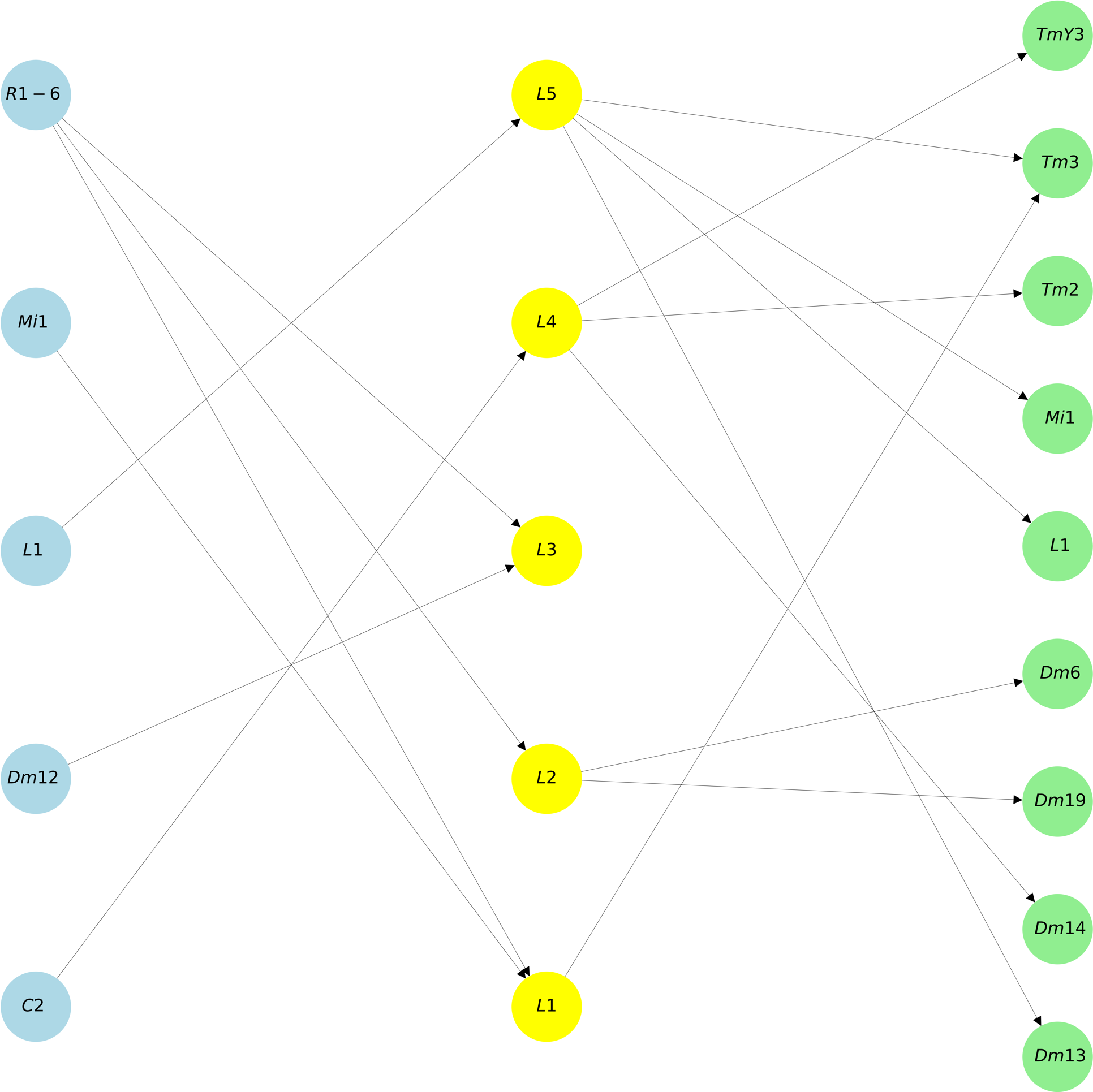

Supplement: Supplementary file 6 — Discriminating logical predicates for all types. Each figure contains types from the same family (middle layer) with shared input attributes (left layer) and output attributes (right layer) that are sufficient for discriminating all types in the middle layer. Families with many types are split into multiple figures for clarity of presentation. [file 41586_2024_7981_MOESM6_ESM.zip › DataS2/pdf/Lamina_Monopolar_Predicates.pdf]

# Lamina Tangential Predicates

*LMTe01*

*Lat*

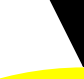

Supplement: Supplementary file 6 — Discriminating logical predicates for all types. Each figure contains types from the same family (middle layer) with shared input attributes (left layer) and output attributes (right layer) that are sufficient for discriminating all types in the middle layer. Families with many types are split into multiple figures for clarity of presentation. [file 41586_2024_7981_MOESM6_ESM.zip › DataS2/pdf/Lamina_Tangential_Predicates.pdf]

**Lamina Wide Field Predicates**

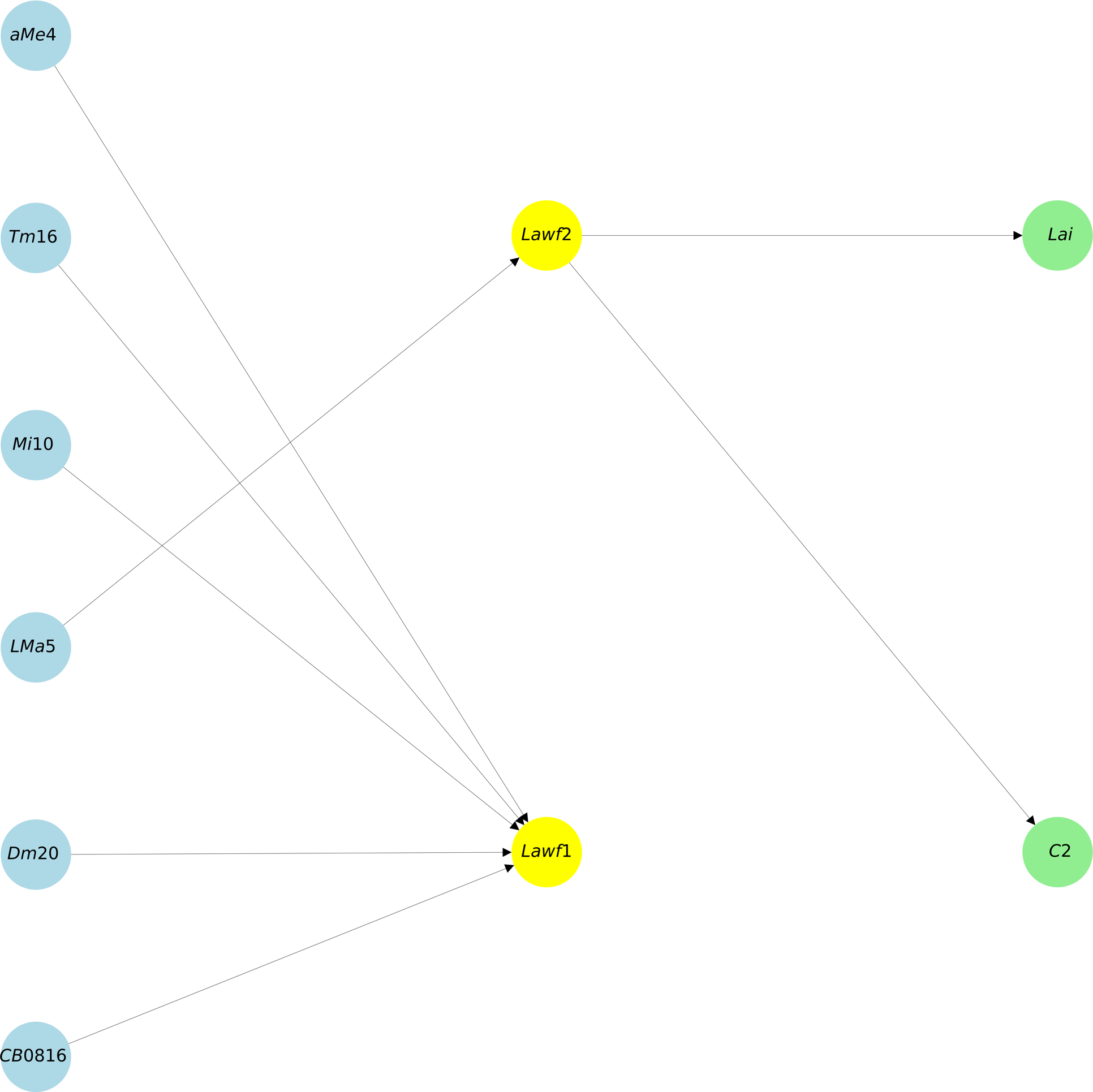

Supplement: Supplementary file 6 — Discriminating logical predicates for all types. Each figure contains types from the same family (middle layer) with shared input attributes (left layer) and output attributes (right layer) that are sufficient for discriminating all types in the middle layer. Families with many types are split into multiple figures for clarity of presentation. [file 41586_2024_7981_MOESM6_ESM.zip › DataS2/pdf/Lamina_Wide_Field_Predicates.pdf]

**Lobula Intrinsic Predicates (part 1 of 7)**

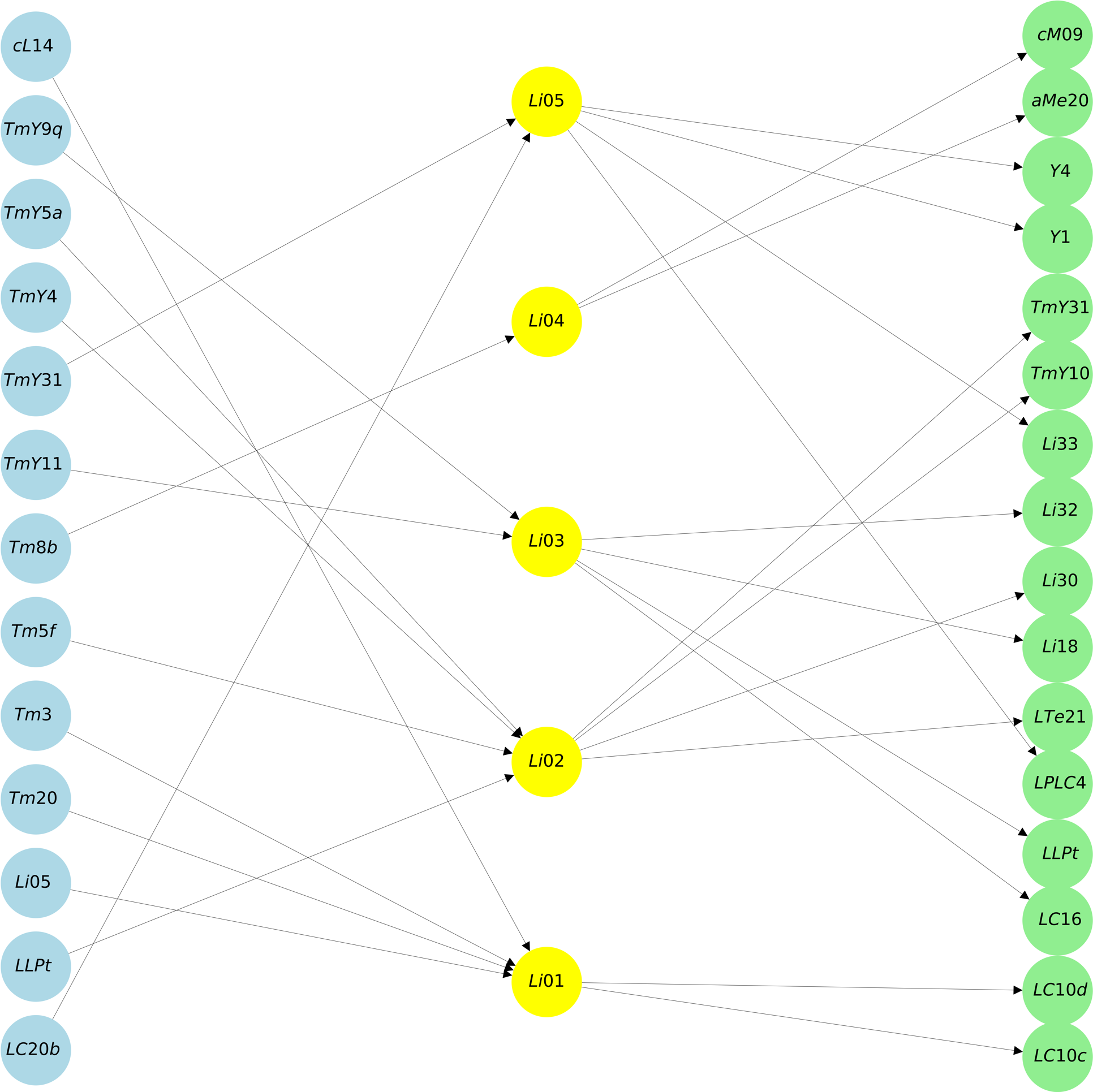

Supplement: Supplementary file 6 — Discriminating logical predicates for all types. Each figure contains types from the same family (middle layer) with shared input attributes (left layer) and output attributes (right layer) that are sufficient for discriminating all types in the middle layer. Families with many types are split into multiple figures for clarity of presentation. [file 41586_2024_7981_MOESM6_ESM.zip › DataS2/pdf/Lobula_Intrinsic_Predicates_(part_1_of_7).pdf]

Lobula Intrinsic Predicates (part 2 of 7)

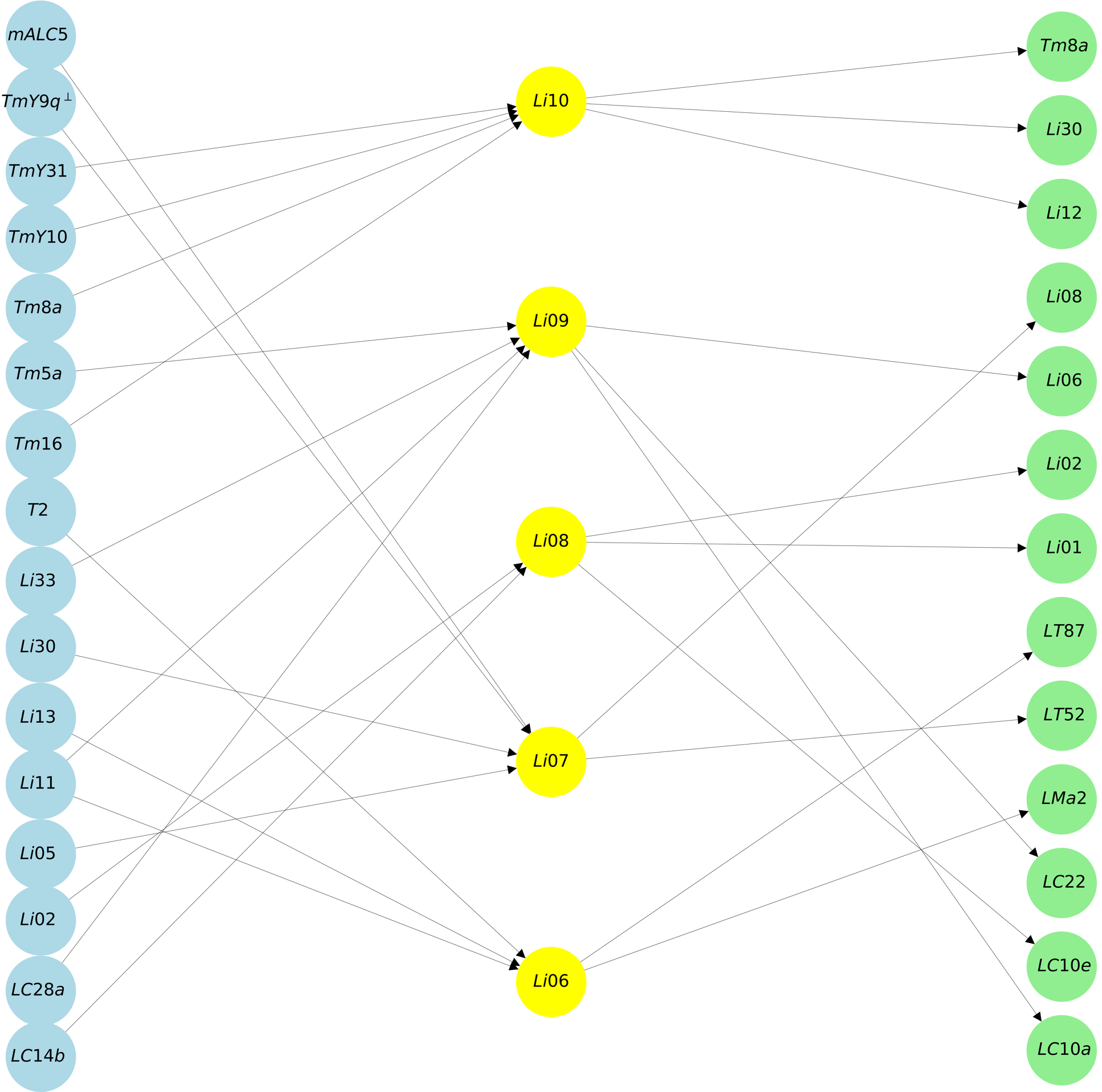

Supplement: Supplementary file 6 — Discriminating logical predicates for all types. Each figure contains types from the same family (middle layer) with shared input attributes (left layer) and output attributes (right layer) that are sufficient for discriminating all types in the middle layer. Families with many types are split into multiple figures for clarity of presentation. [file 41586_2024_7981_MOESM6_ESM.zip › DataS2/pdf/Lobula_Intrinsic_Predicates_(part_2_of_7).pdf]

## Lobula Intrinsic Predicates (part 3 of 7)

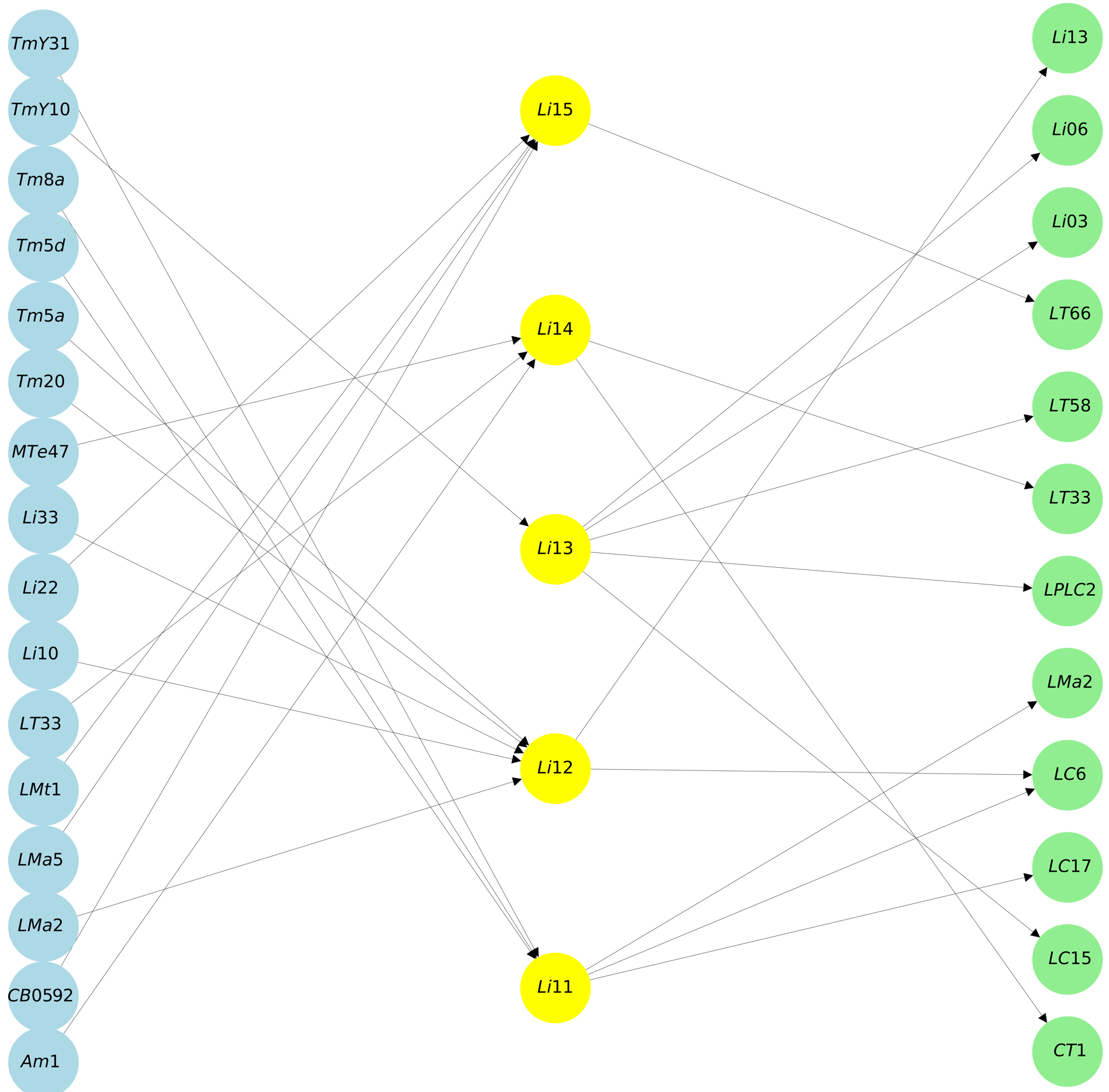

Supplement: Supplementary file 6 — Discriminating logical predicates for all types. Each figure contains types from the same family (middle layer) with shared input attributes (left layer) and output attributes (right layer) that are sufficient for discriminating all types in the middle layer. Families with many types are split into multiple figures for clarity of presentation. [file 41586_2024_7981_MOESM6_ESM.zip › DataS2/pdf/Lobula_Intrinsic_Predicates_(part_3_of_7).pdf]

**Lobula Intrinsic Predicates (part 4 of 7)**

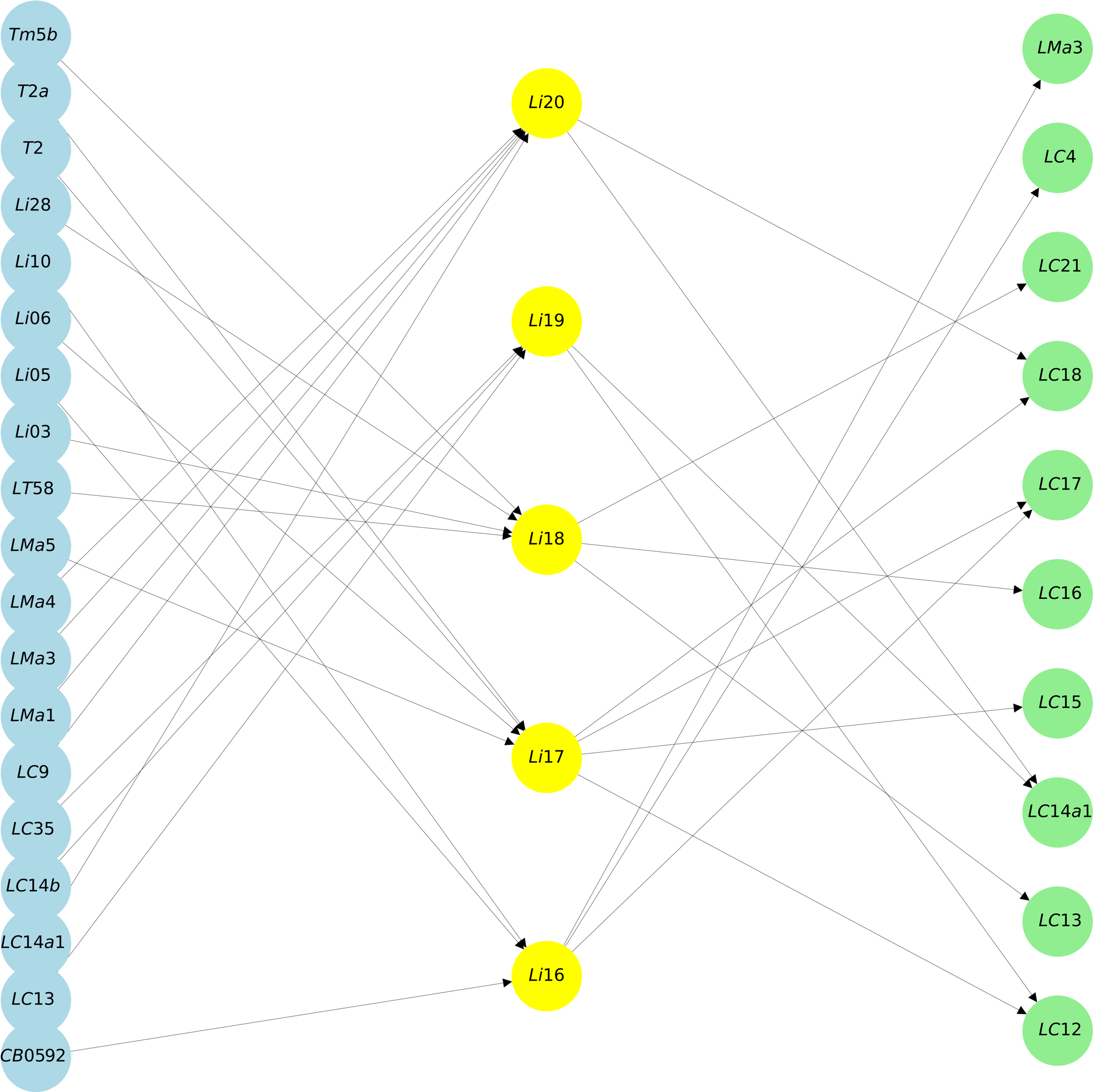

Supplement: Supplementary file 6 — Discriminating logical predicates for all types. Each figure contains types from the same family (middle layer) with shared input attributes (left layer) and output attributes (right layer) that are sufficient for discriminating all types in the middle layer. Families with many types are split into multiple figures for clarity of presentation. [file 41586_2024_7981_MOESM6_ESM.zip › DataS2/pdf/Lobula_Intrinsic_Predicates_(part_4_of_7).pdf]

Lobula Intrinsic Predicates (part 5 of 7)

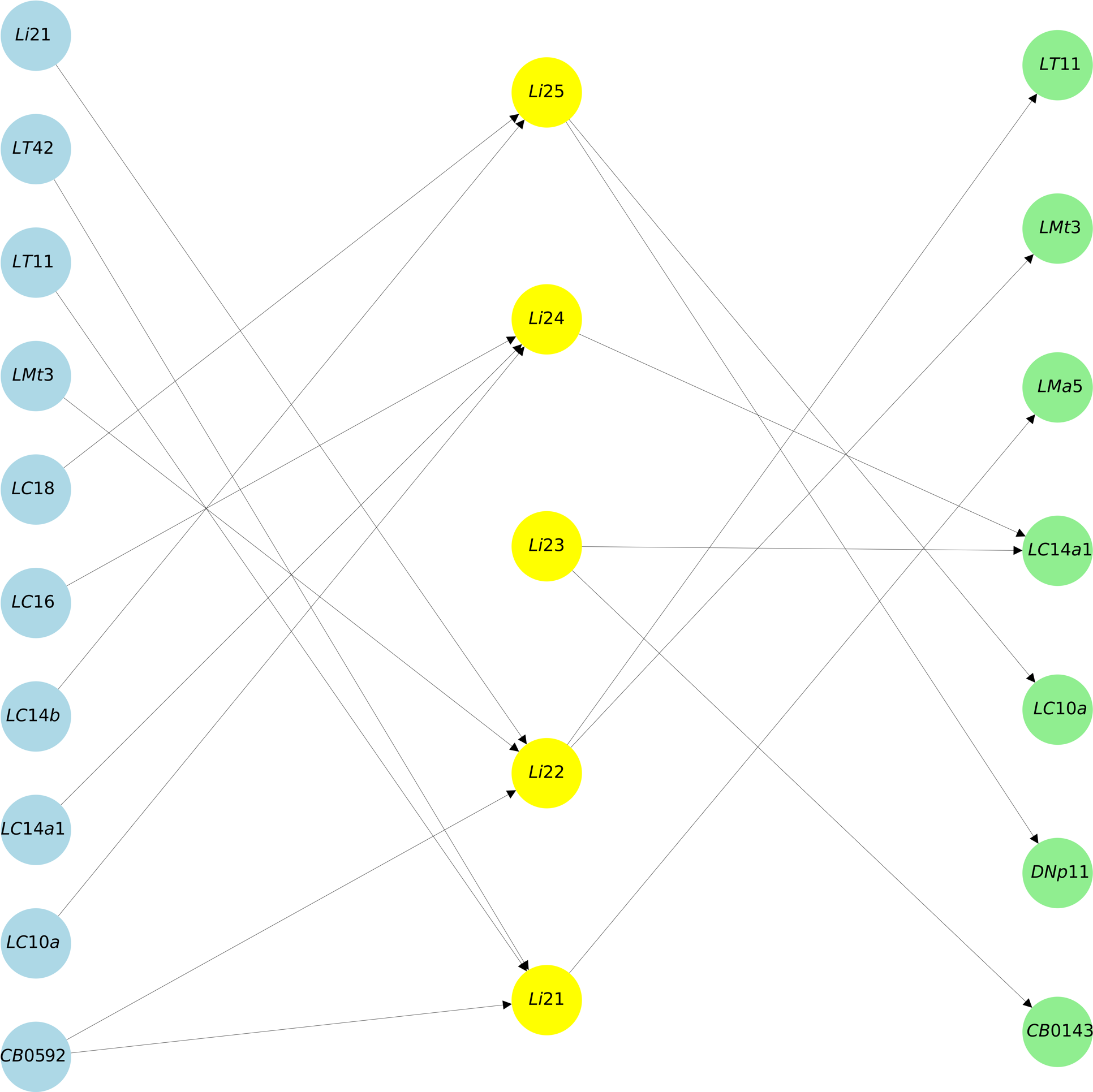

Supplement: Supplementary file 6 — Discriminating logical predicates for all types. Each figure contains types from the same family (middle layer) with shared input attributes (left layer) and output attributes (right layer) that are sufficient for discriminating all types in the middle layer. Families with many types are split into multiple figures for clarity of presentation. [file 41586_2024_7981_MOESM6_ESM.zip › DataS2/pdf/Lobula_Intrinsic_Predicates_(part_5_of_7).pdf]

**Lobula Intrinsic Predicates (part 6 of 7)**

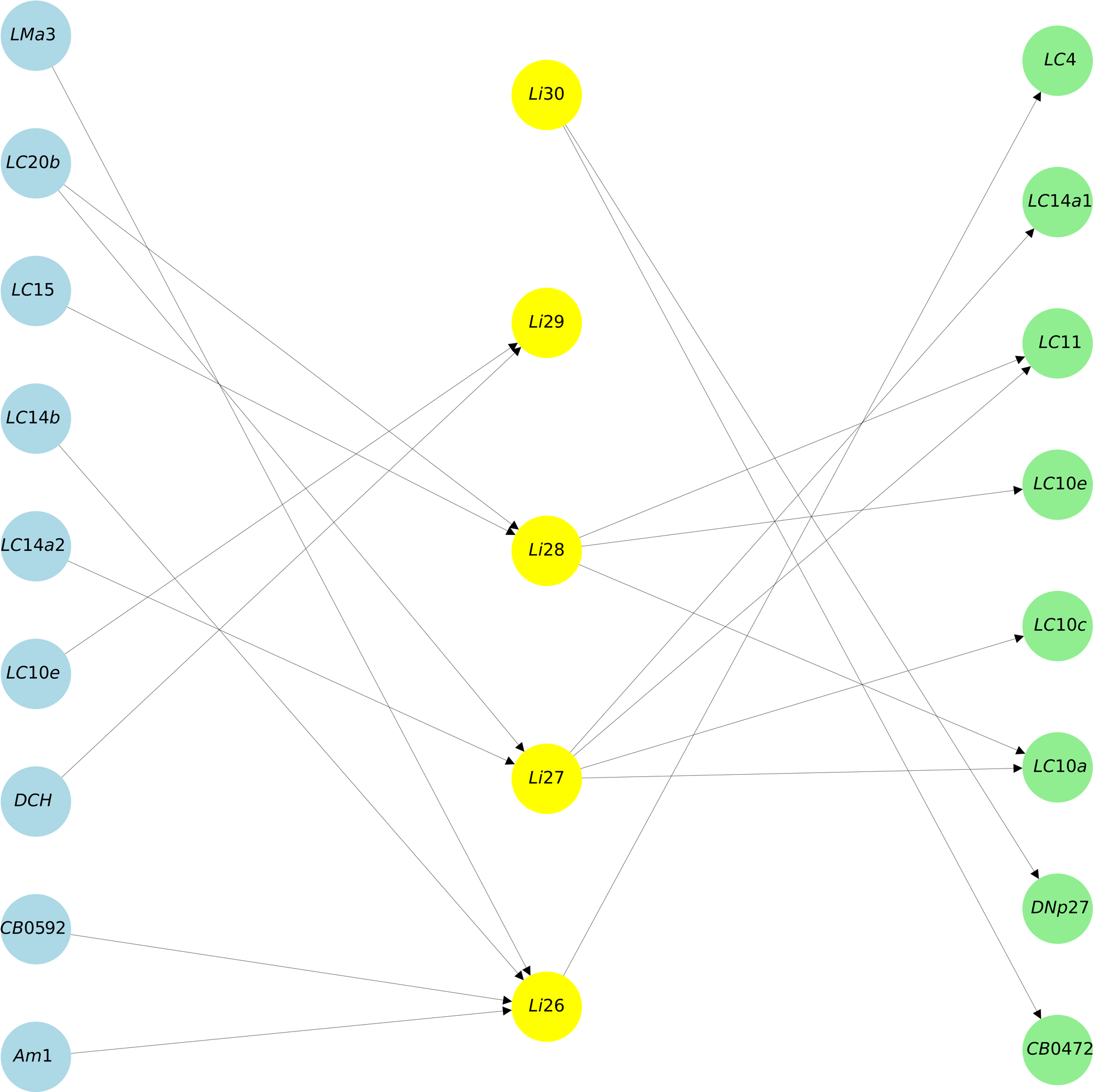

Supplement: Supplementary file 6 — Discriminating logical predicates for all types. Each figure contains types from the same family (middle layer) with shared input attributes (left layer) and output attributes (right layer) that are sufficient for discriminating all types in the middle layer. Families with many types are split into multiple figures for clarity of presentation. [file 41586_2024_7981_MOESM6_ESM.zip › DataS2/pdf/Lobula_Intrinsic_Predicates_(part_6_of_7).pdf]

**Lobula Intrinsic Predicates (part 7 of 7)**

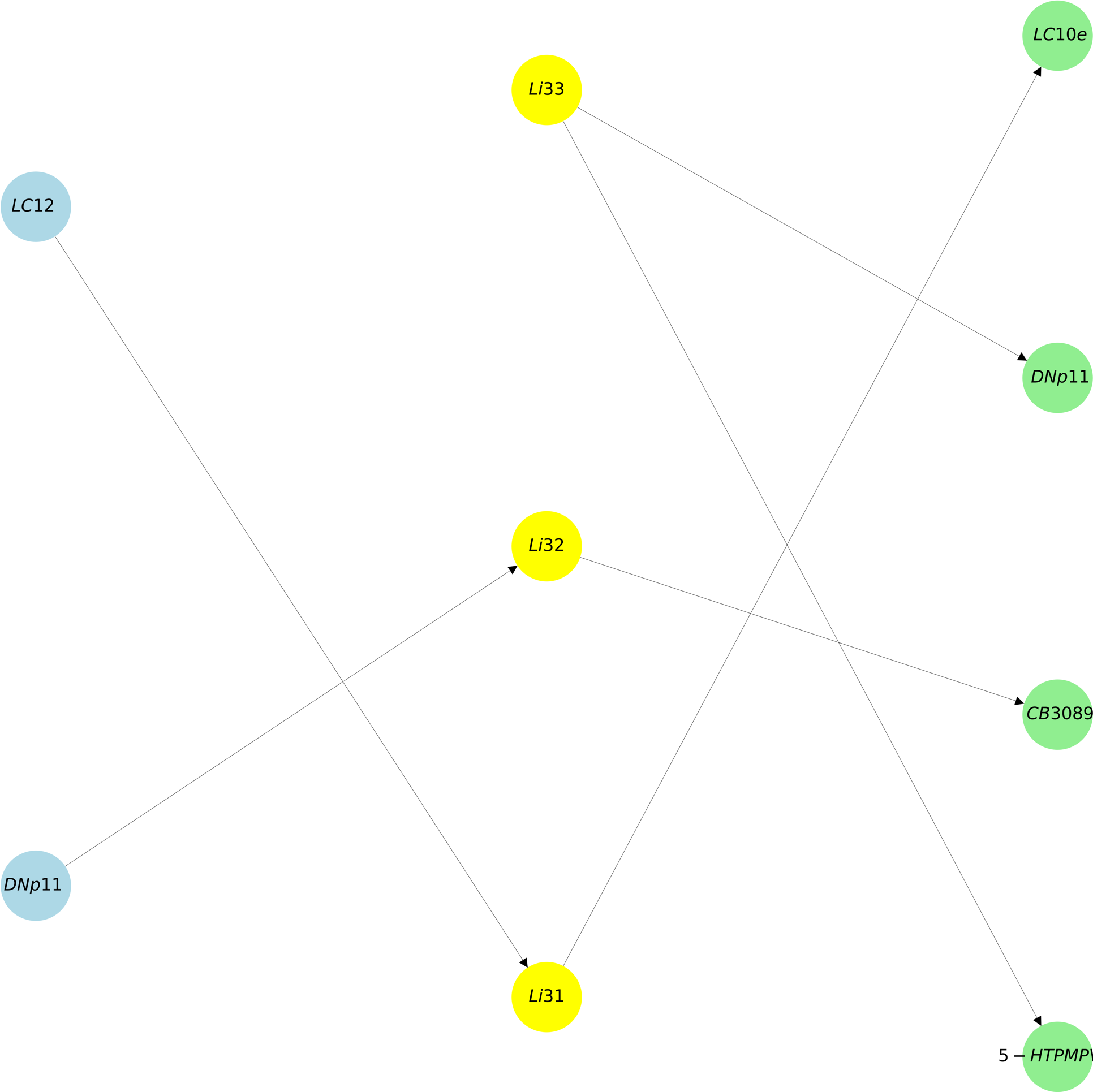

Supplement: Supplementary file 6 — Discriminating logical predicates for all types. Each figure contains types from the same family (middle layer) with shared input attributes (left layer) and output attributes (right layer) that are sufficient for discriminating all types in the middle layer. Families with many types are split into multiple figures for clarity of presentation. [file 41586_2024_7981_MOESM6_ESM.zip › DataS2/pdf/Lobula_Intrinsic_Predicates_(part_7_of_7).pdf]

Lobula Lobula Plate Tangential Predicates

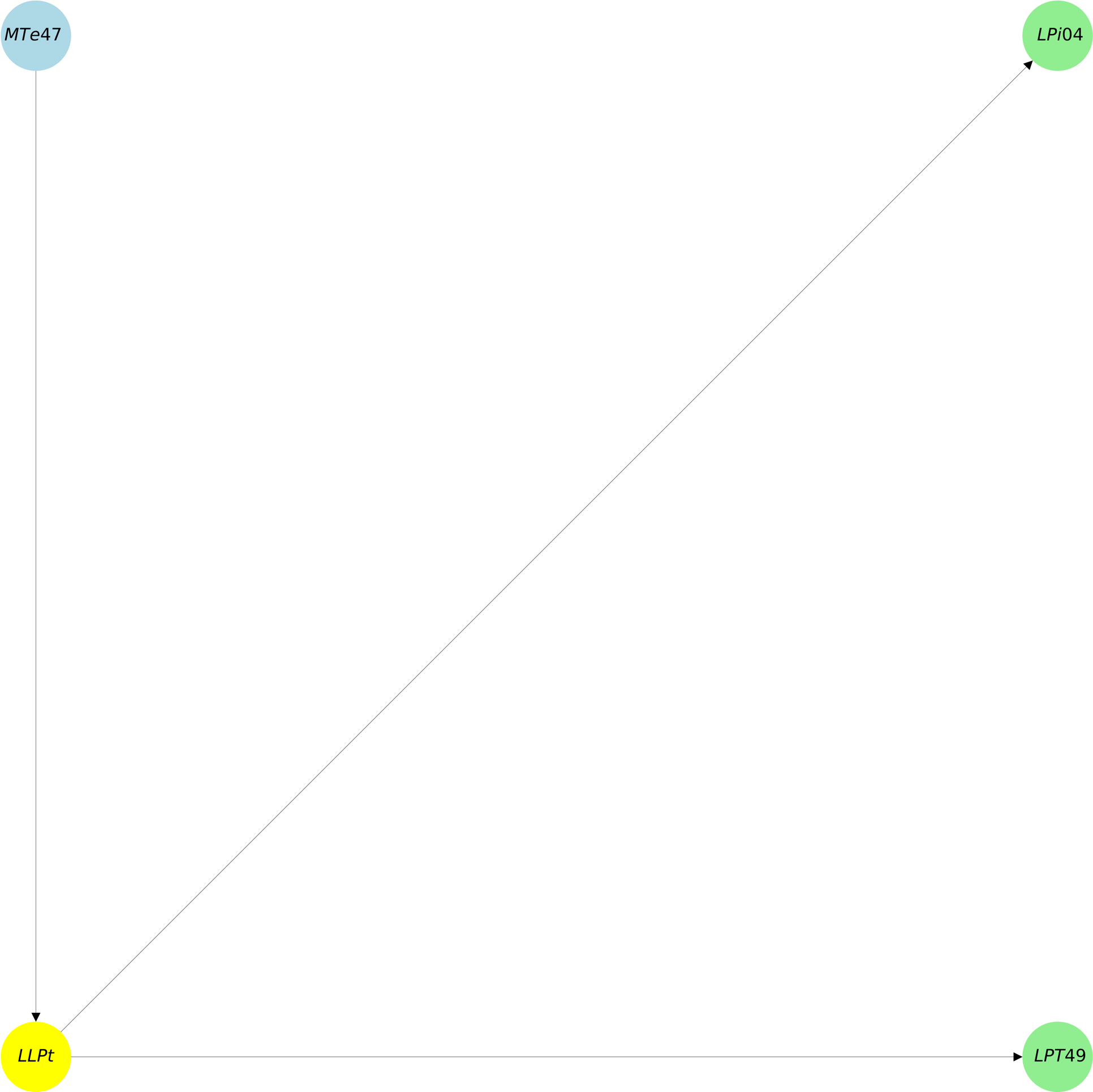

Supplement: Supplementary file 6 — Discriminating logical predicates for all types. Each figure contains types from the same family (middle layer) with shared input attributes (left layer) and output attributes (right layer) that are sufficient for discriminating all types in the middle layer. Families with many types are split into multiple figures for clarity of presentation. [file 41586_2024_7981_MOESM6_ESM.zip › DataS2/pdf/Lobula_Lobula_Plate_Tangential_Predicates.pdf]

Lobula Medulla Amacrine Predicates (part 1 of 2)

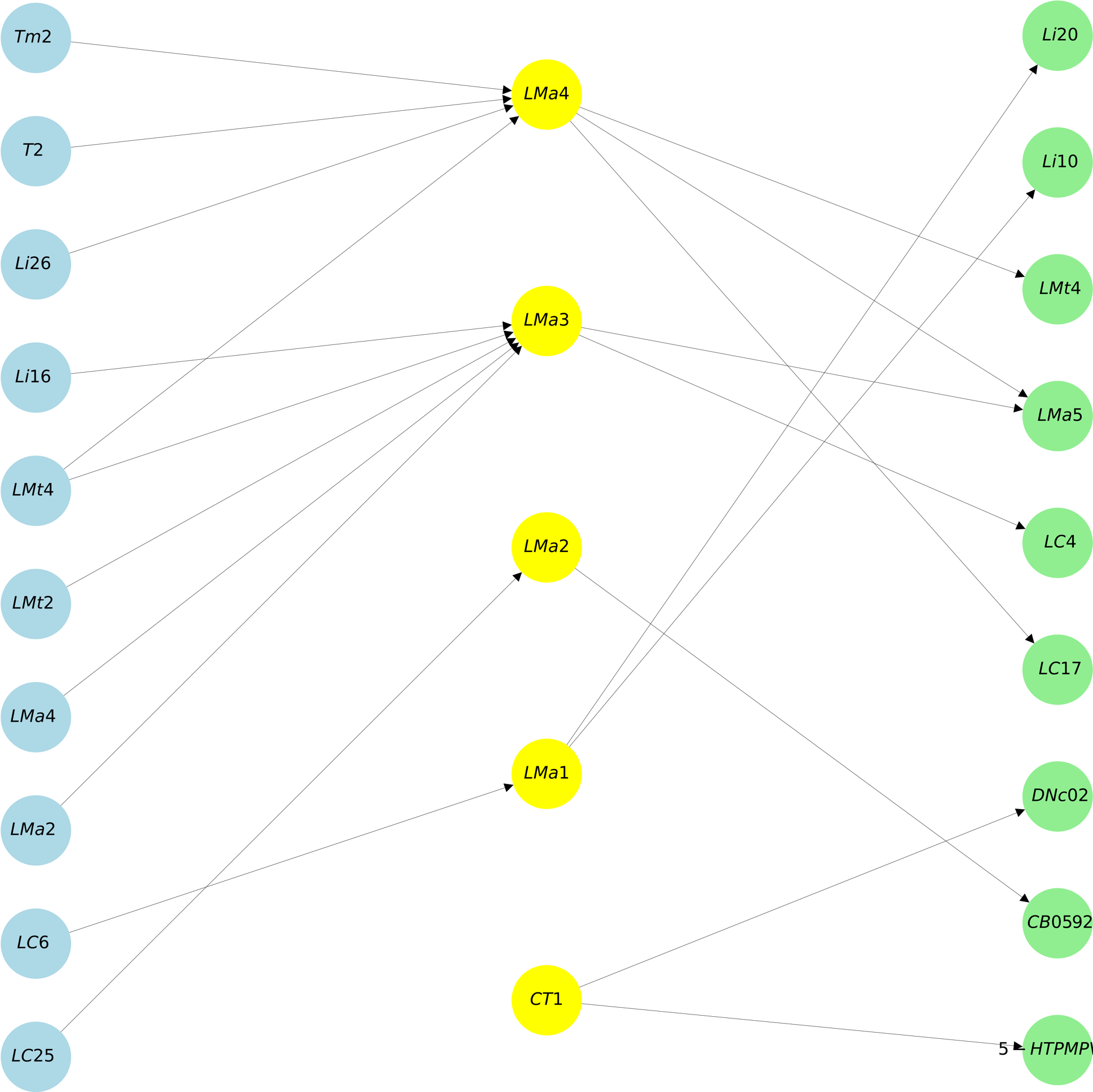

Supplement: Supplementary file 6 — Discriminating logical predicates for all types. Each figure contains types from the same family (middle layer) with shared input attributes (left layer) and output attributes (right layer) that are sufficient for discriminating all types in the middle layer. Families with many types are split into multiple figures for clarity of presentation. [file 41586_2024_7981_MOESM6_ESM.zip › DataS2/pdf/Lobula_Medulla_Amacrine_Predicates_(part_1_of_2).pdf]

Lobula Medulla Amacrine Predicates (part 2 of 2)

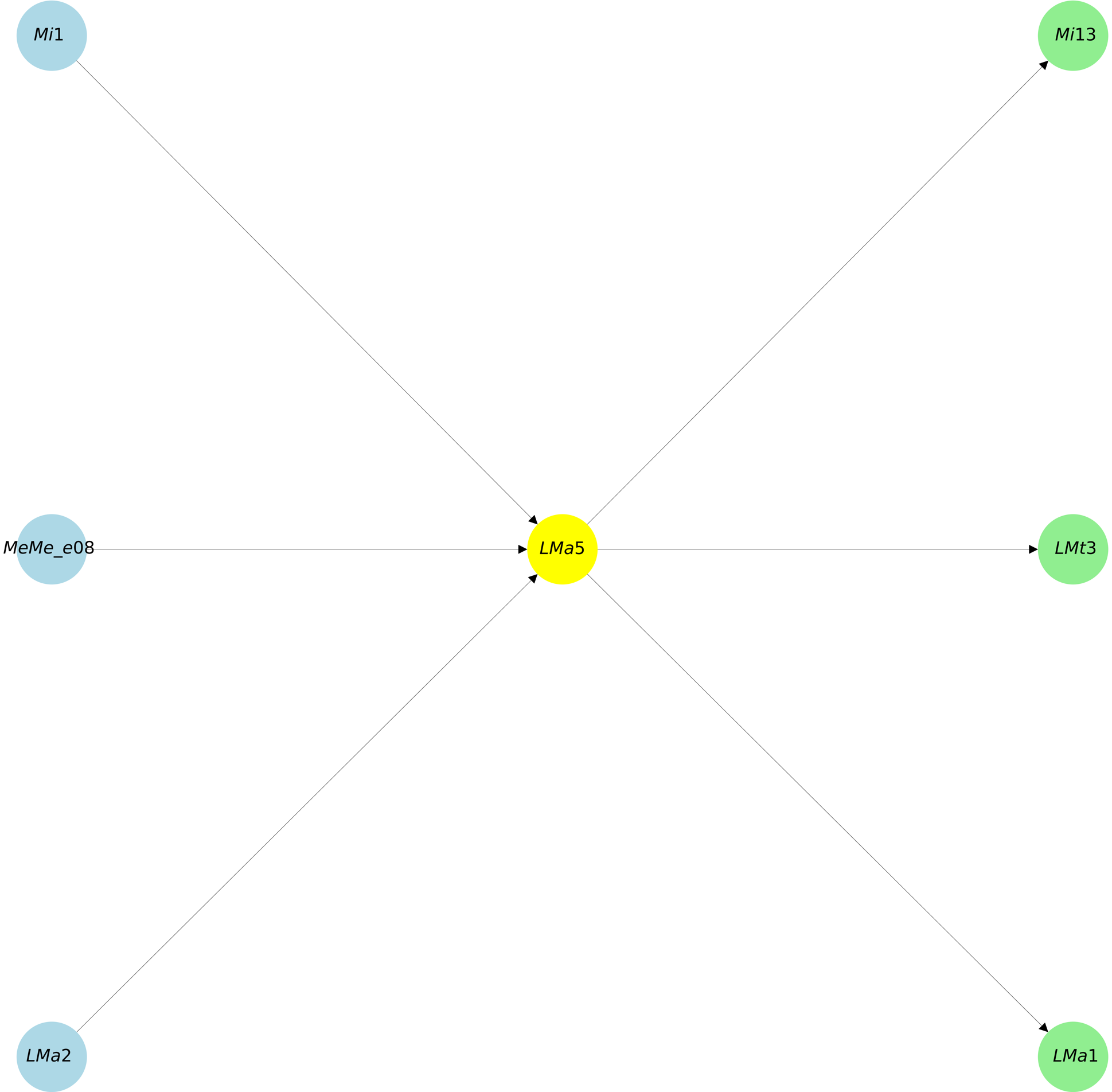

Supplement: Supplementary file 6 — Discriminating logical predicates for all types. Each figure contains types from the same family (middle layer) with shared input attributes (left layer) and output attributes (right layer) that are sufficient for discriminating all types in the middle layer. Families with many types are split into multiple figures for clarity of presentation. [file 41586_2024_7981_MOESM6_ESM.zip › DataS2/pdf/Lobula_Medulla_Amacrine_Predicates_(part_2_of_2).pdf]

**Lobula Medulla Tangential Predicates**

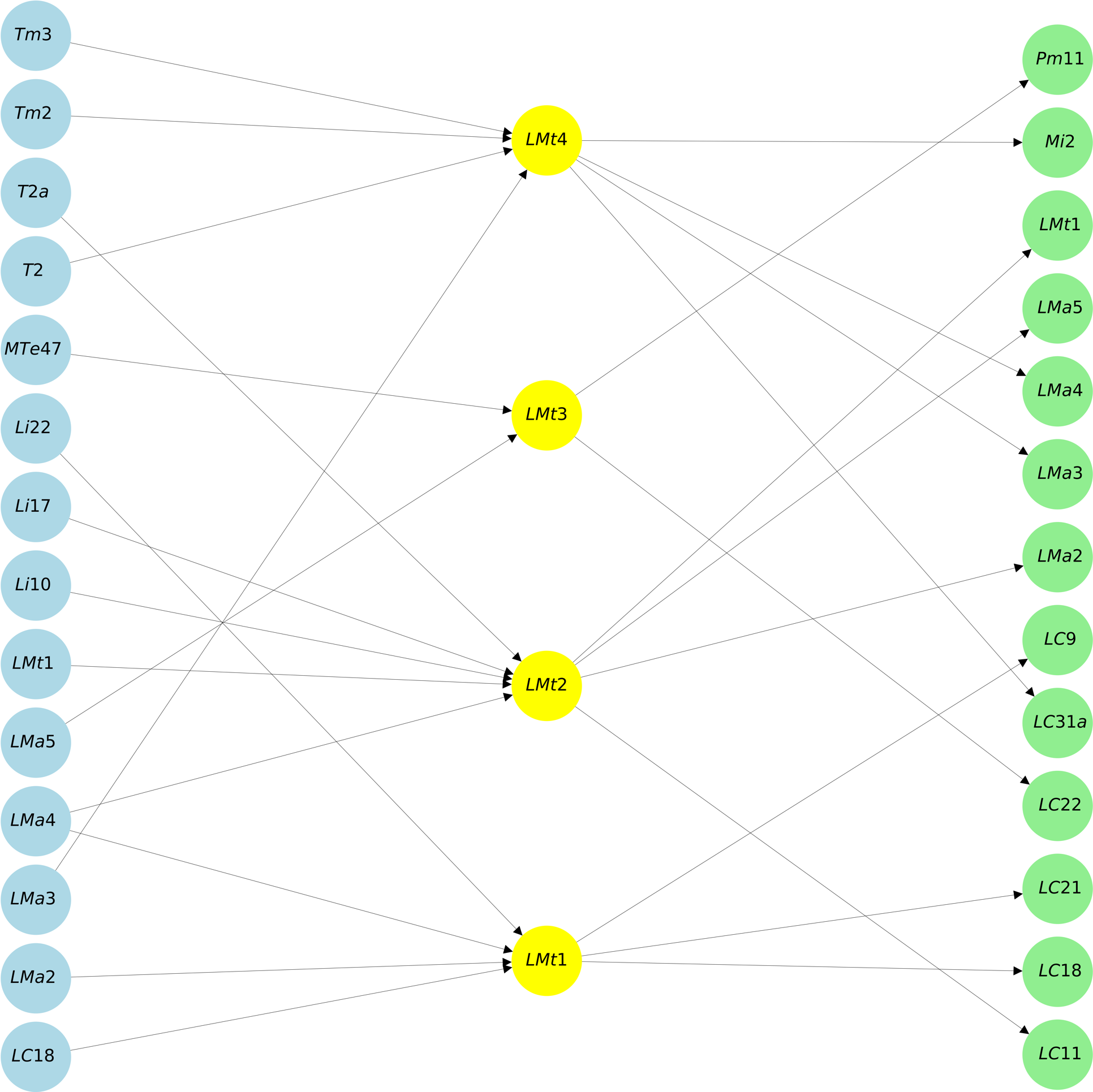

Supplement: Supplementary file 6 — Discriminating logical predicates for all types. Each figure contains types from the same family (middle layer) with shared input attributes (left layer) and output attributes (right layer) that are sufficient for discriminating all types in the middle layer. Families with many types are split into multiple figures for clarity of presentation. [file 41586_2024_7981_MOESM6_ESM.zip › DataS2/pdf/Lobula_Medulla_Tangential_Predicates.pdf]

## Lobula Plate Intrinsic Predicates (part 1 of 3)

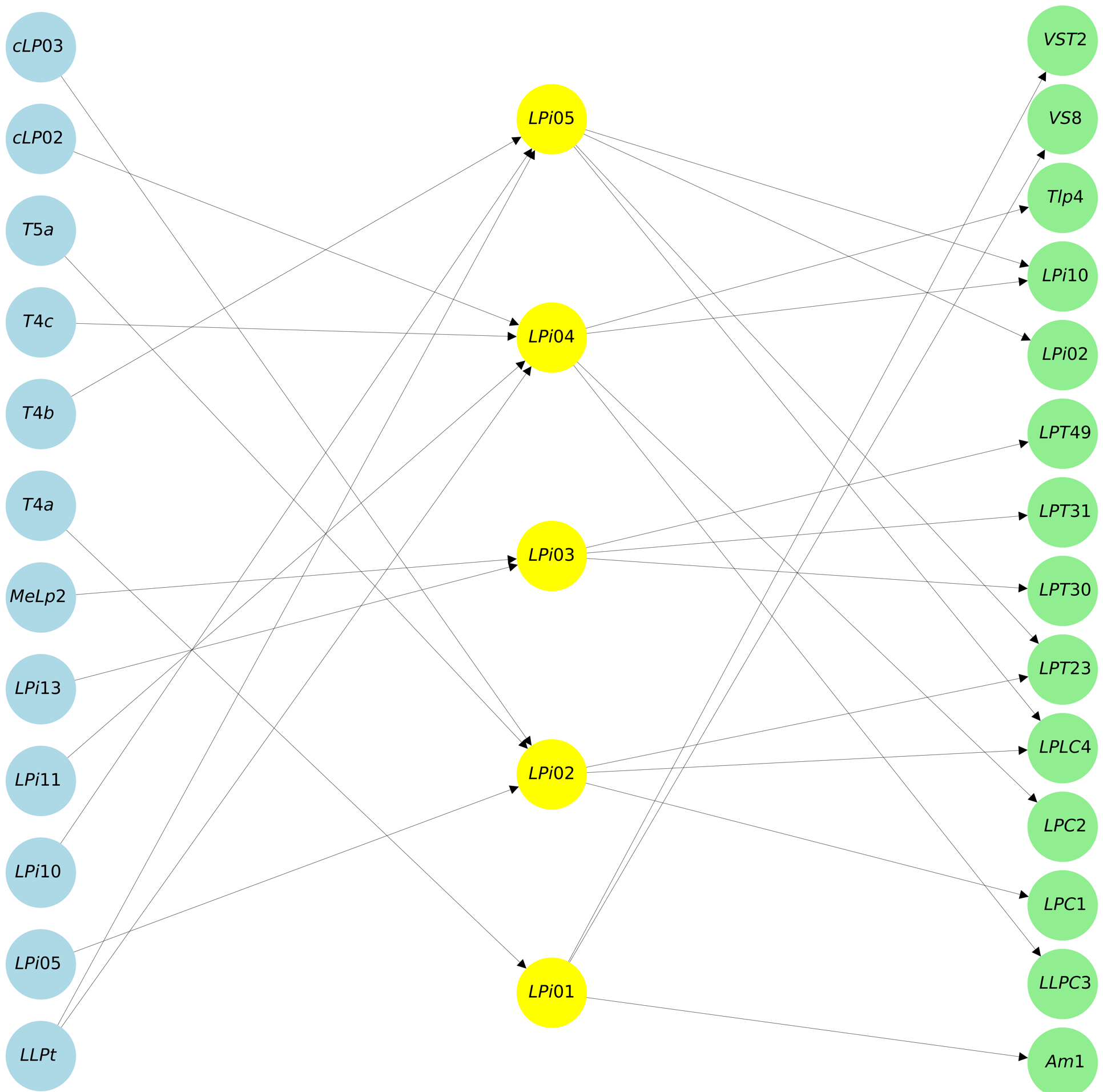

Supplement: Supplementary file 6 — Discriminating logical predicates for all types. Each figure contains types from the same family (middle layer) with shared input attributes (left layer) and output attributes (right layer) that are sufficient for discriminating all types in the middle layer. Families with many types are split into multiple figures for clarity of presentation. [file 41586_2024_7981_MOESM6_ESM.zip › DataS2/pdf/Lobula_Plate_Intrinsic_Predicates_(part_1_of_3).pdf]

Lobula Plate Intrinsic Predicates (part 2 of 3)

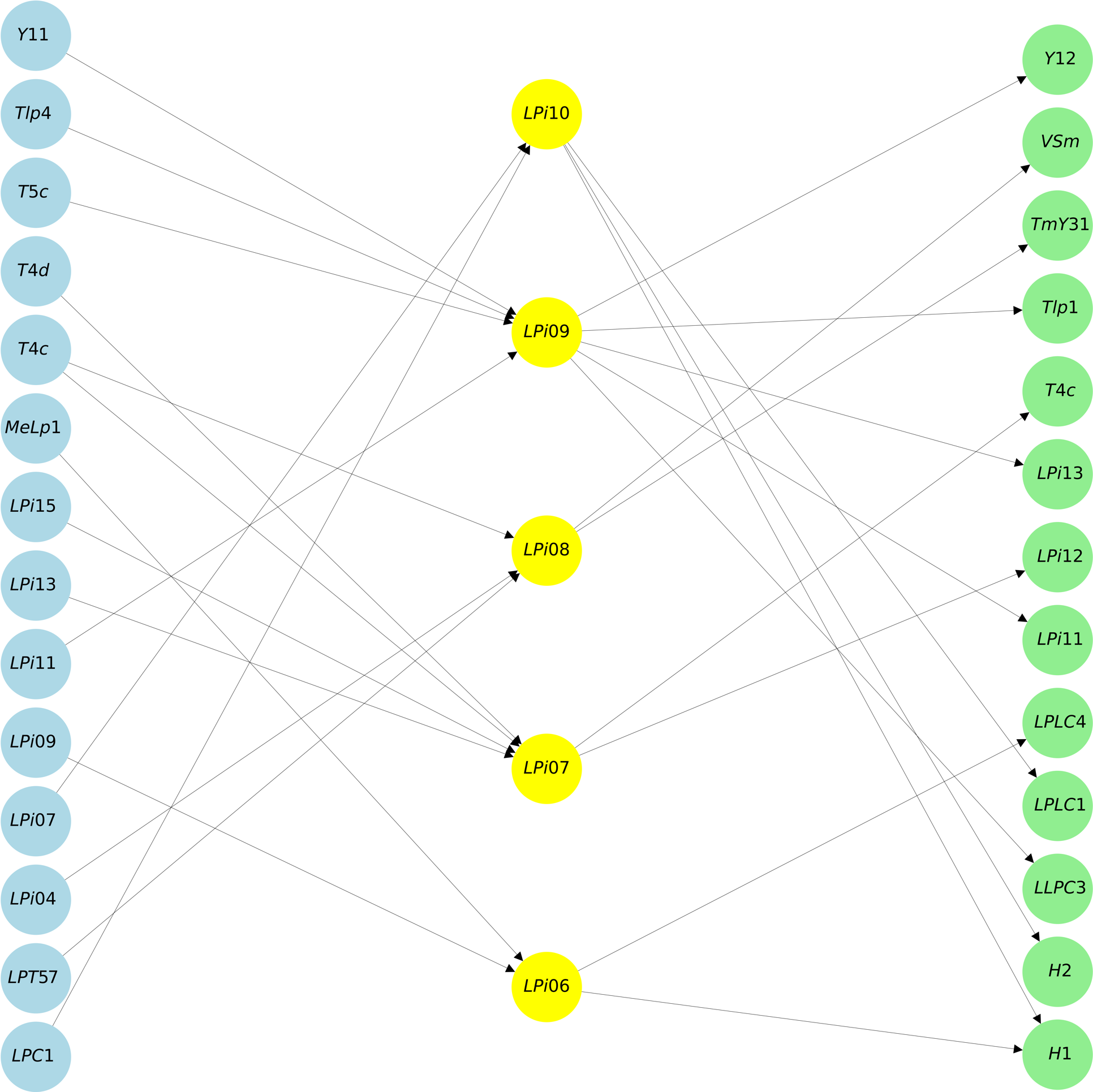

Supplement: Supplementary file 6 — Discriminating logical predicates for all types. Each figure contains types from the same family (middle layer) with shared input attributes (left layer) and output attributes (right layer) that are sufficient for discriminating all types in the middle layer. Families with many types are split into multiple figures for clarity of presentation. [file 41586_2024_7981_MOESM6_ESM.zip › DataS2/pdf/Lobula_Plate_Intrinsic_Predicates_(part_2_of_3).pdf]

**Lobula Plate Intrinsic Predicates (part 3 of 3)**

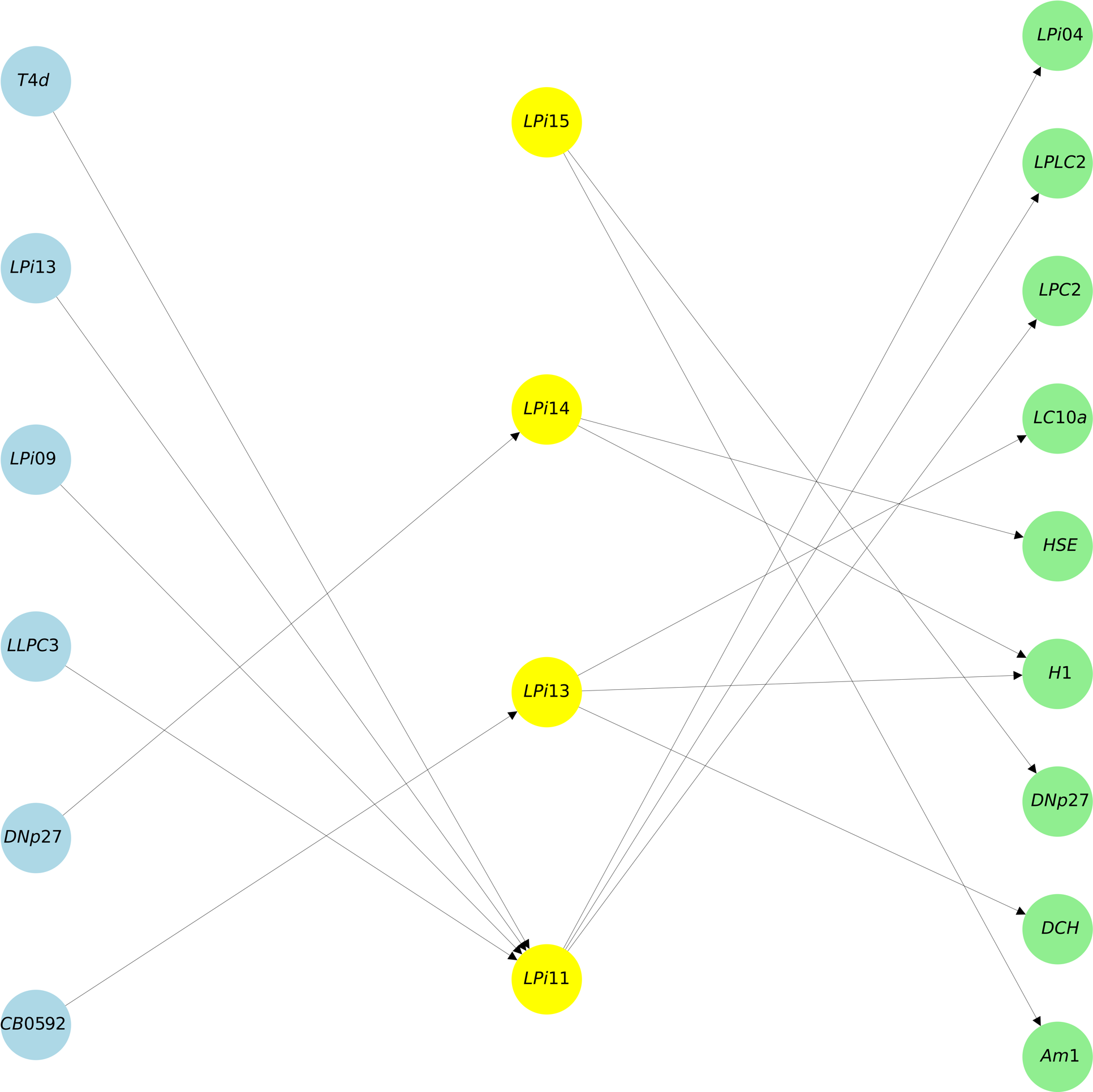

Supplement: Supplementary file 6 — Discriminating logical predicates for all types. Each figure contains types from the same family (middle layer) with shared input attributes (left layer) and output attributes (right layer) that are sufficient for discriminating all types in the middle layer. Families with many types are split into multiple figures for clarity of presentation. [file 41586_2024_7981_MOESM6_ESM.zip › DataS2/pdf/Lobula_Plate_Intrinsic_Predicates_(part_3_of_3).pdf]

## Medulla Intrinsic Predicates (part 1 of 2)

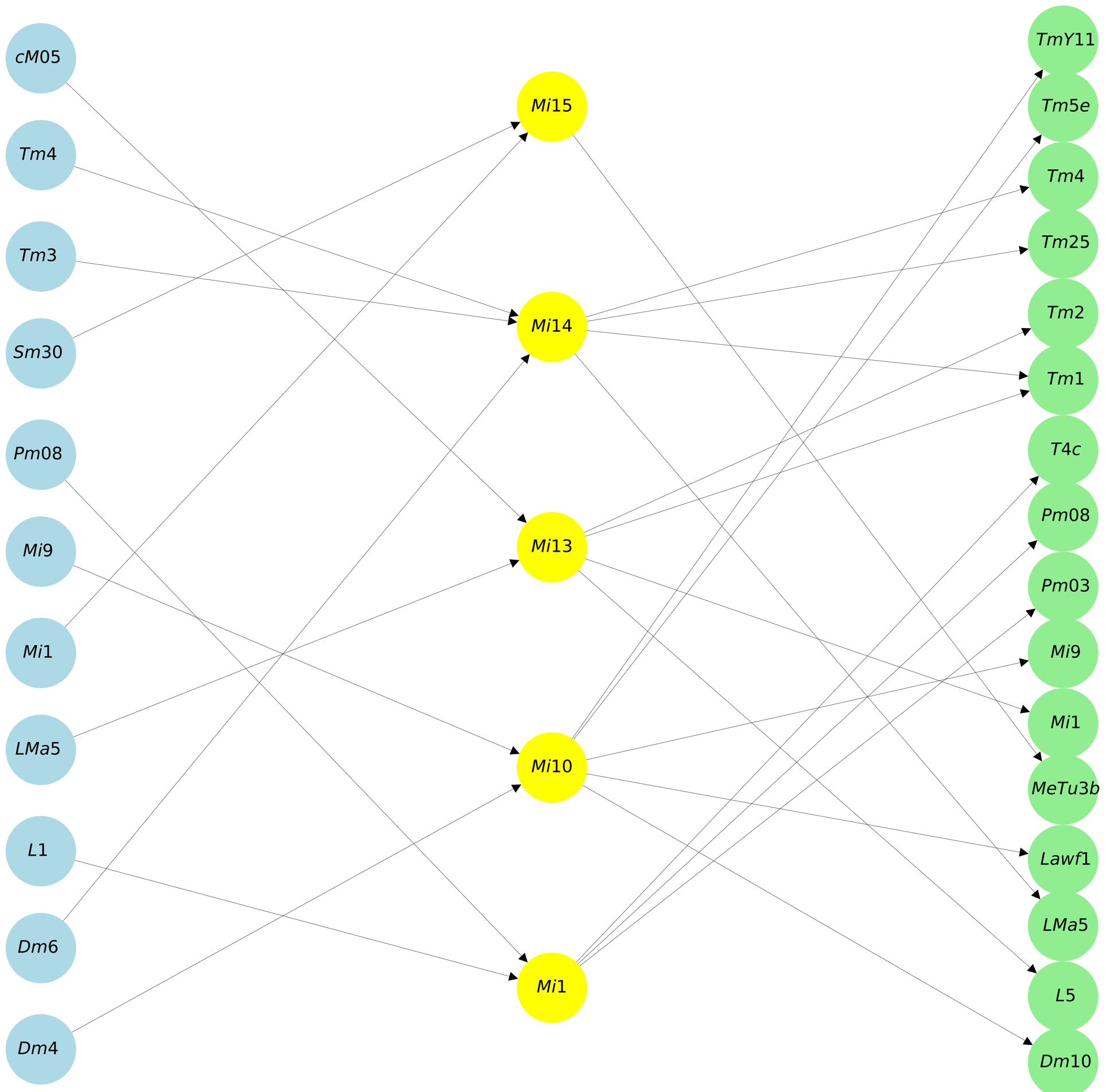

Supplement: Supplementary file 6 — Discriminating logical predicates for all types. Each figure contains types from the same family (middle layer) with shared input attributes (left layer) and output attributes (right layer) that are sufficient for discriminating all types in the middle layer. Families with many types are split into multiple figures for clarity of presentation. [file 41586_2024_7981_MOESM6_ESM.zip › DataS2/pdf/Medulla_Intrinsic_Predicates_(part_1_of_2).pdf]

## Medulla Intrinsic Predicates (part 2 of 2)

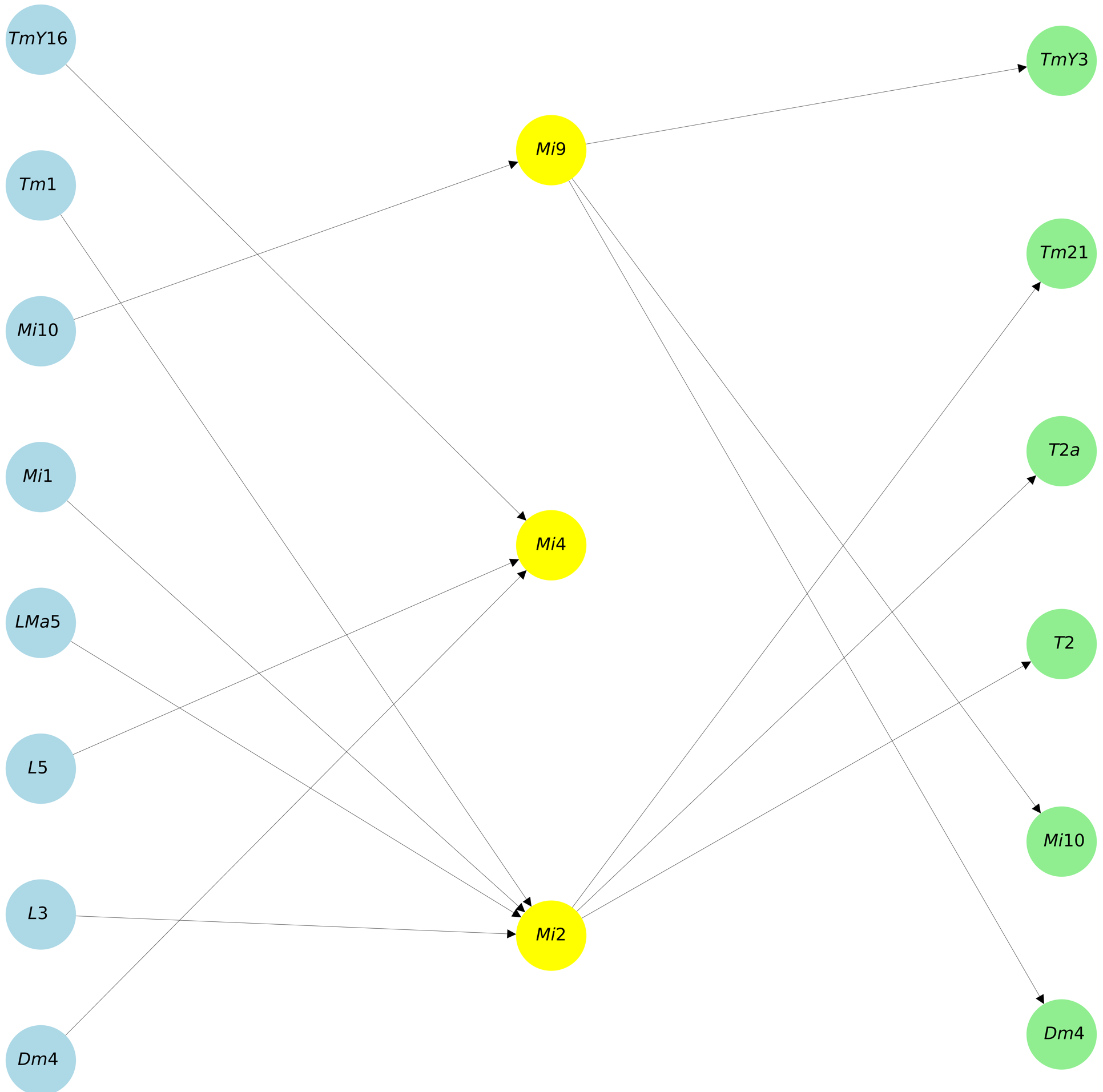

Supplement: Supplementary file 6 — Discriminating logical predicates for all types. Each figure contains types from the same family (middle layer) with shared input attributes (left layer) and output attributes (right layer) that are sufficient for discriminating all types in the middle layer. Families with many types are split into multiple figures for clarity of presentation. [file 41586_2024_7981_MOESM6_ESM.zip › DataS2/pdf/Medulla_Intrinsic_Predicates_(part_2_of_2).pdf]

Medulla Lobula Lobula Plate Amacrine Predicates

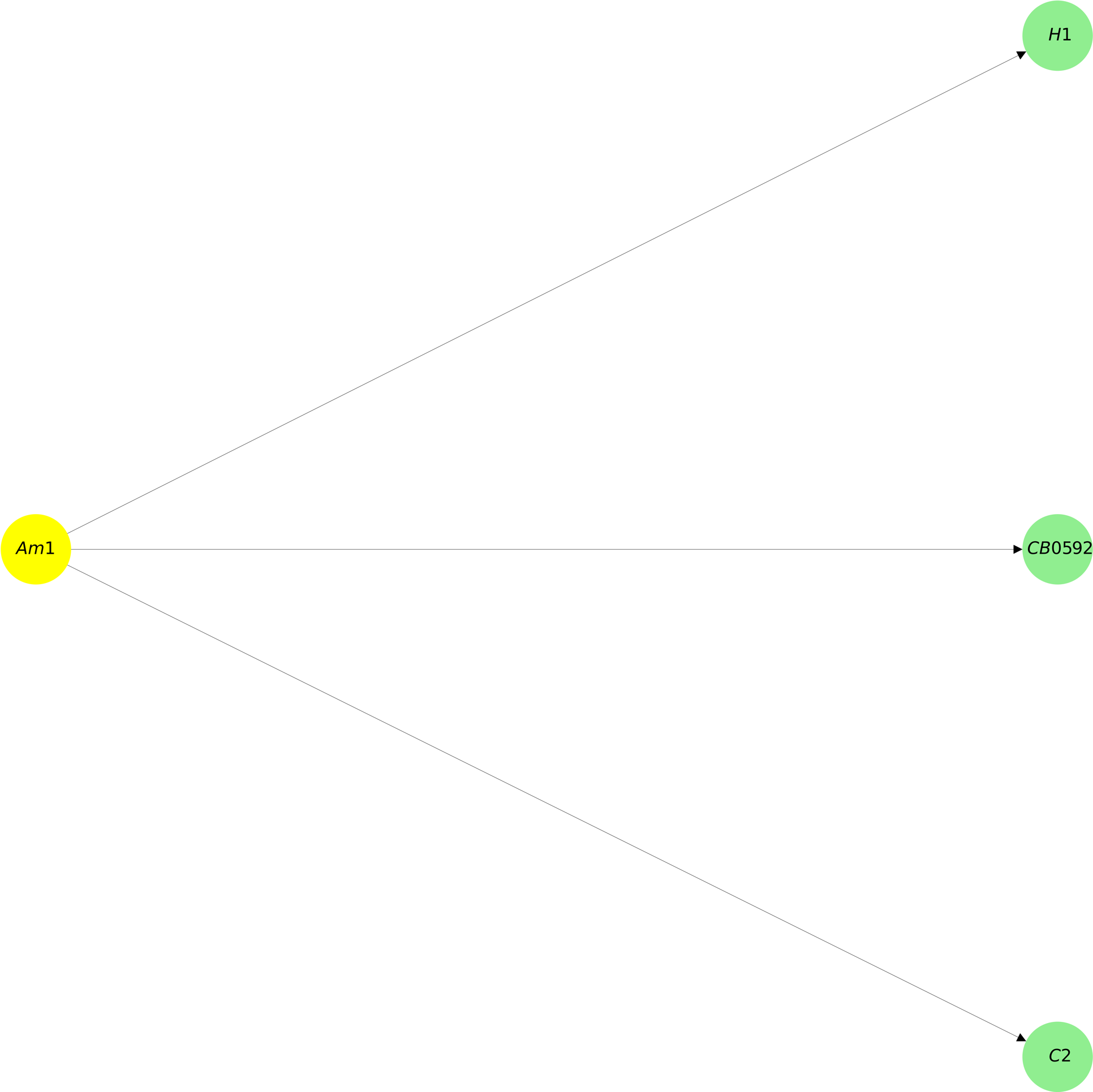

Supplement: Supplementary file 6 — Discriminating logical predicates for all types. Each figure contains types from the same family (middle layer) with shared input attributes (left layer) and output attributes (right layer) that are sufficient for discriminating all types in the middle layer. Families with many types are split into multiple figures for clarity of presentation. [file 41586_2024_7981_MOESM6_ESM.zip › DataS2/pdf/Medulla_Lobula_Lobula_Plate_Amacrine_Predicates.pdf]

## Medulla Lobula Tangential Predicates (part 1 of 2)

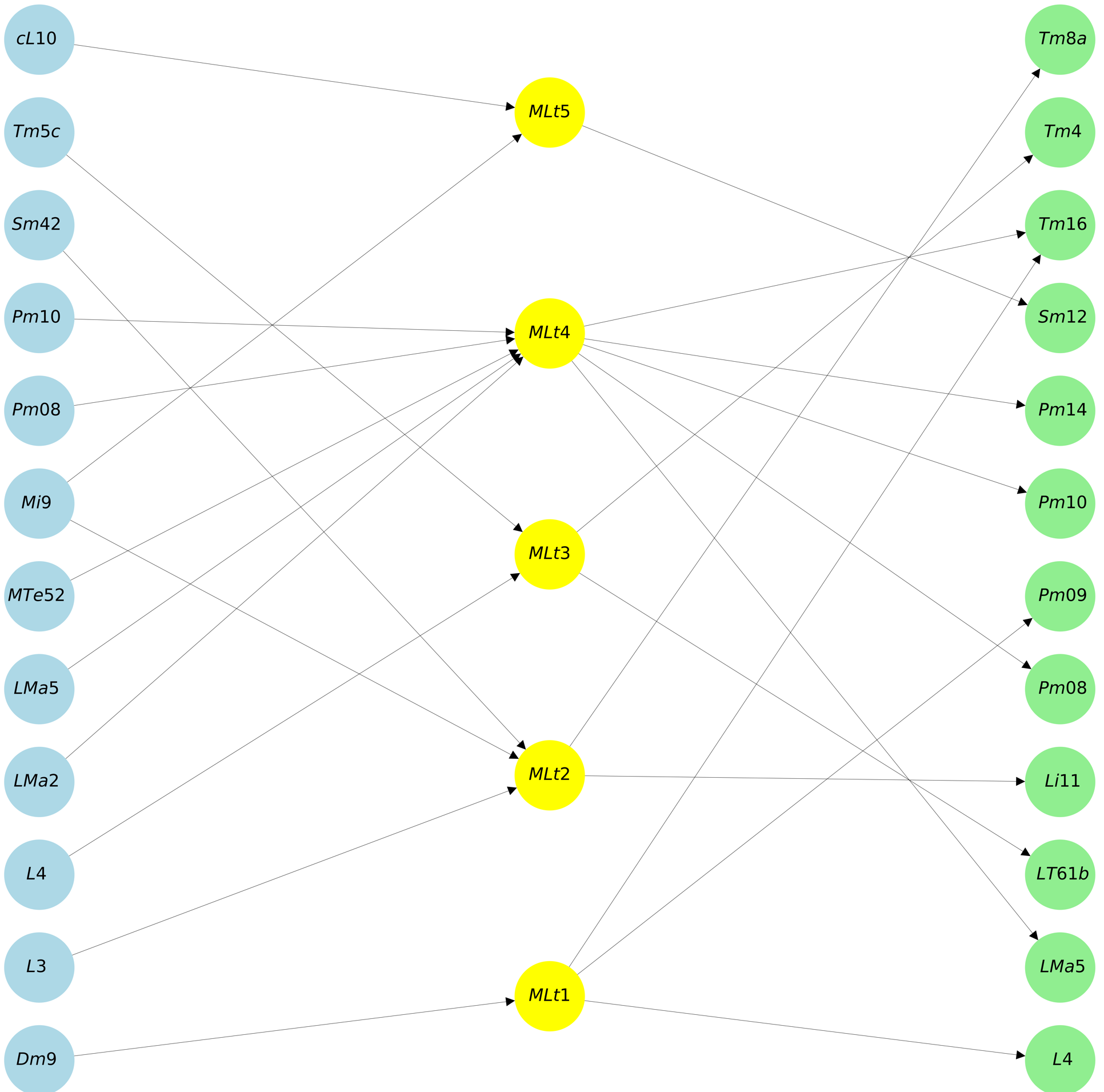

Supplement: Supplementary file 6 — Discriminating logical predicates for all types. Each figure contains types from the same family (middle layer) with shared input attributes (left layer) and output attributes (right layer) that are sufficient for discriminating all types in the middle layer. Families with many types are split into multiple figures for clarity of presentation. [file 41586_2024_7981_MOESM6_ESM.zip › DataS2/pdf/Medulla_Lobula_Tangential_Predicates_(part_1_of_2).pdf]

## Medulla Lobula Tangential Predicates (part 2 of 2)

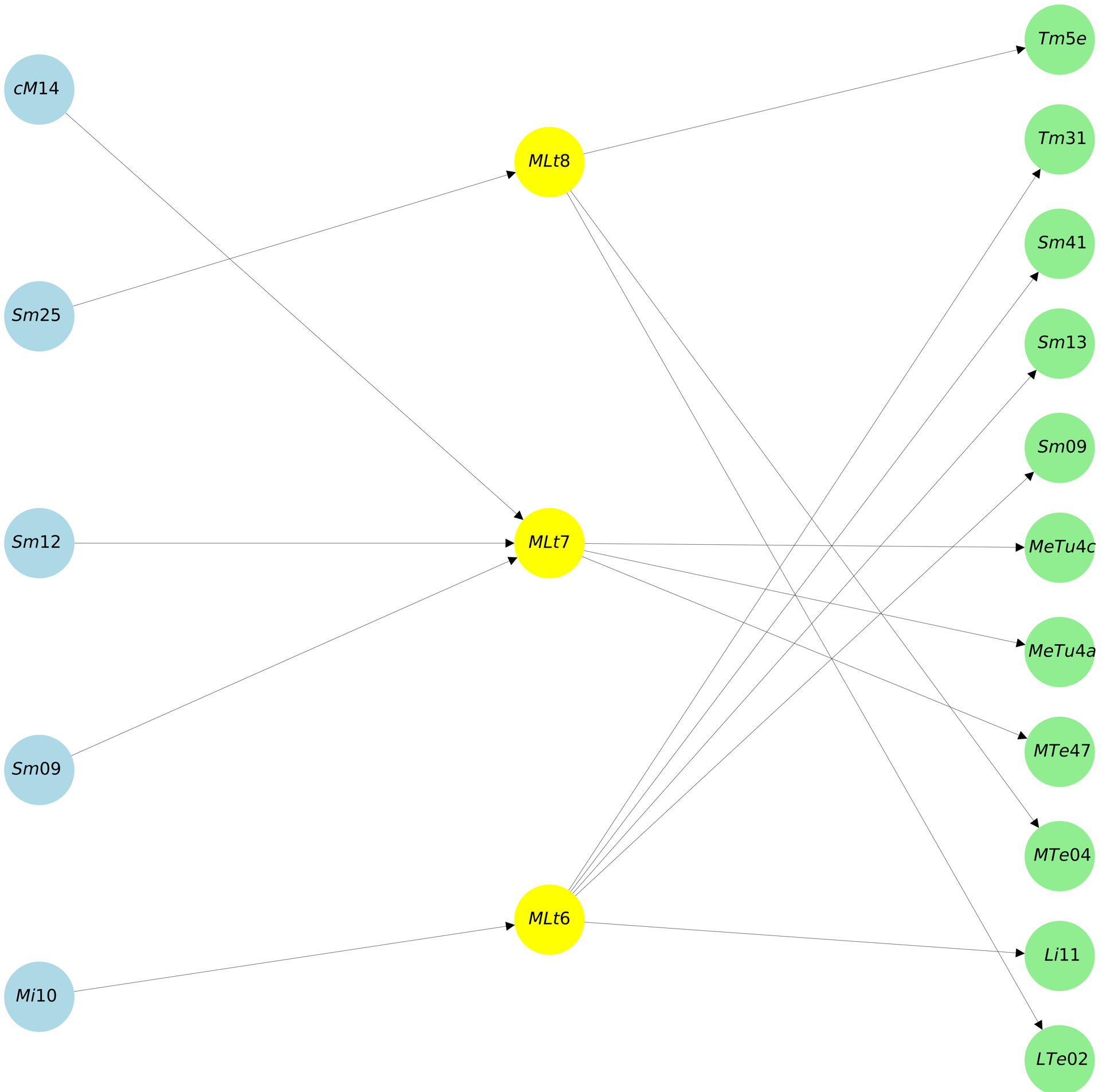

Supplement: Supplementary file 6 — Discriminating logical predicates for all types. Each figure contains types from the same family (middle layer) with shared input attributes (left layer) and output attributes (right layer) that are sufficient for discriminating all types in the middle layer. Families with many types are split into multiple figures for clarity of presentation. [file 41586_2024_7981_MOESM6_ESM.zip › DataS2/pdf/Medulla_Lobula_Tangential_Predicates_(part_2_of_2).pdf]

# Photo Receptors Predicates

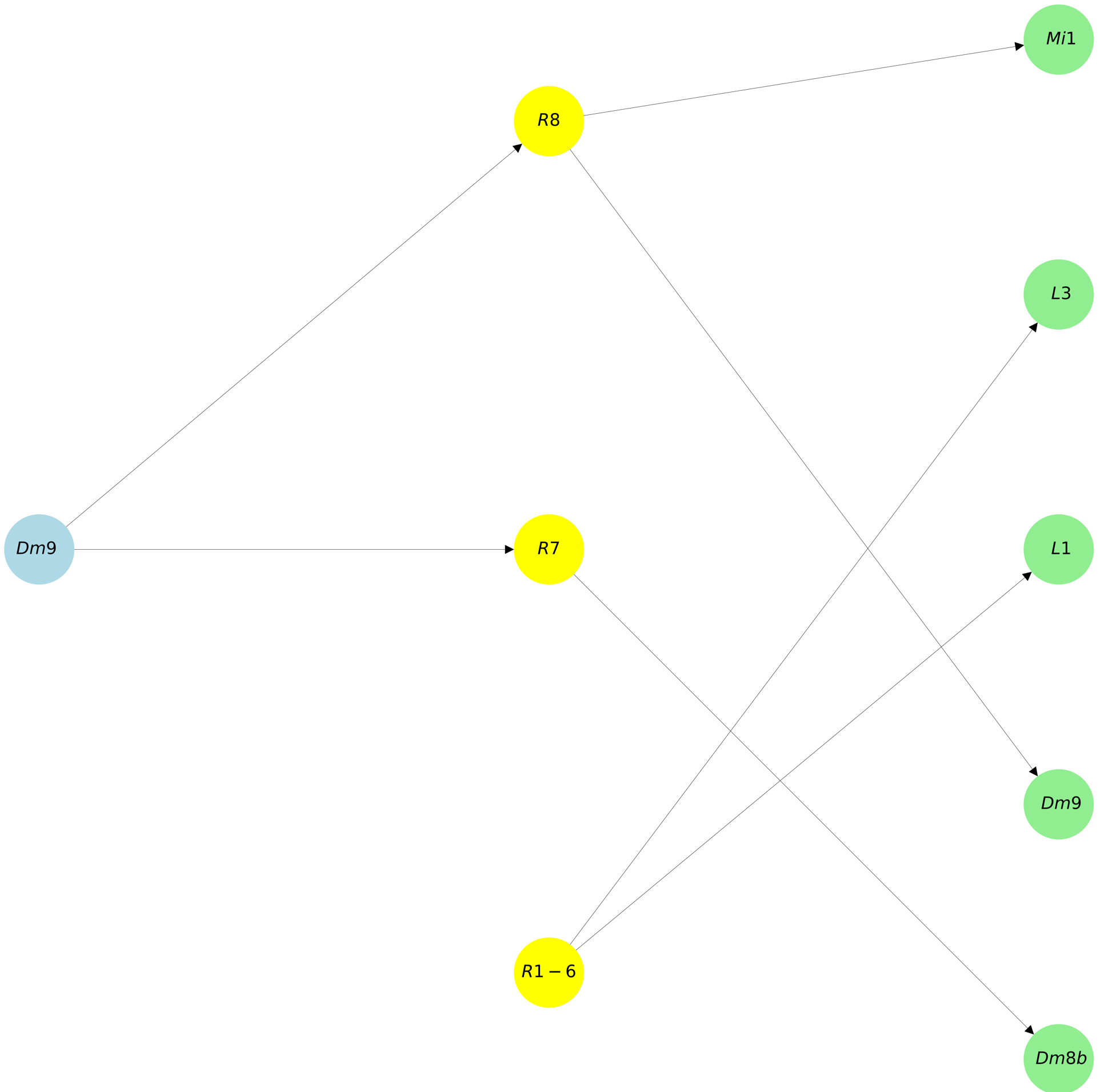

Supplement: Supplementary file 6 — Discriminating logical predicates for all types. Each figure contains types from the same family (middle layer) with shared input attributes (left layer) and output attributes (right layer) that are sufficient for discriminating all types in the middle layer. Families with many types are split into multiple figures for clarity of presentation. [file 41586_2024_7981_MOESM6_ESM.zip › DataS2/pdf/Photo_Receptors_Predicates.pdf]

# Proximal Distal Medulla Tangential Predicates

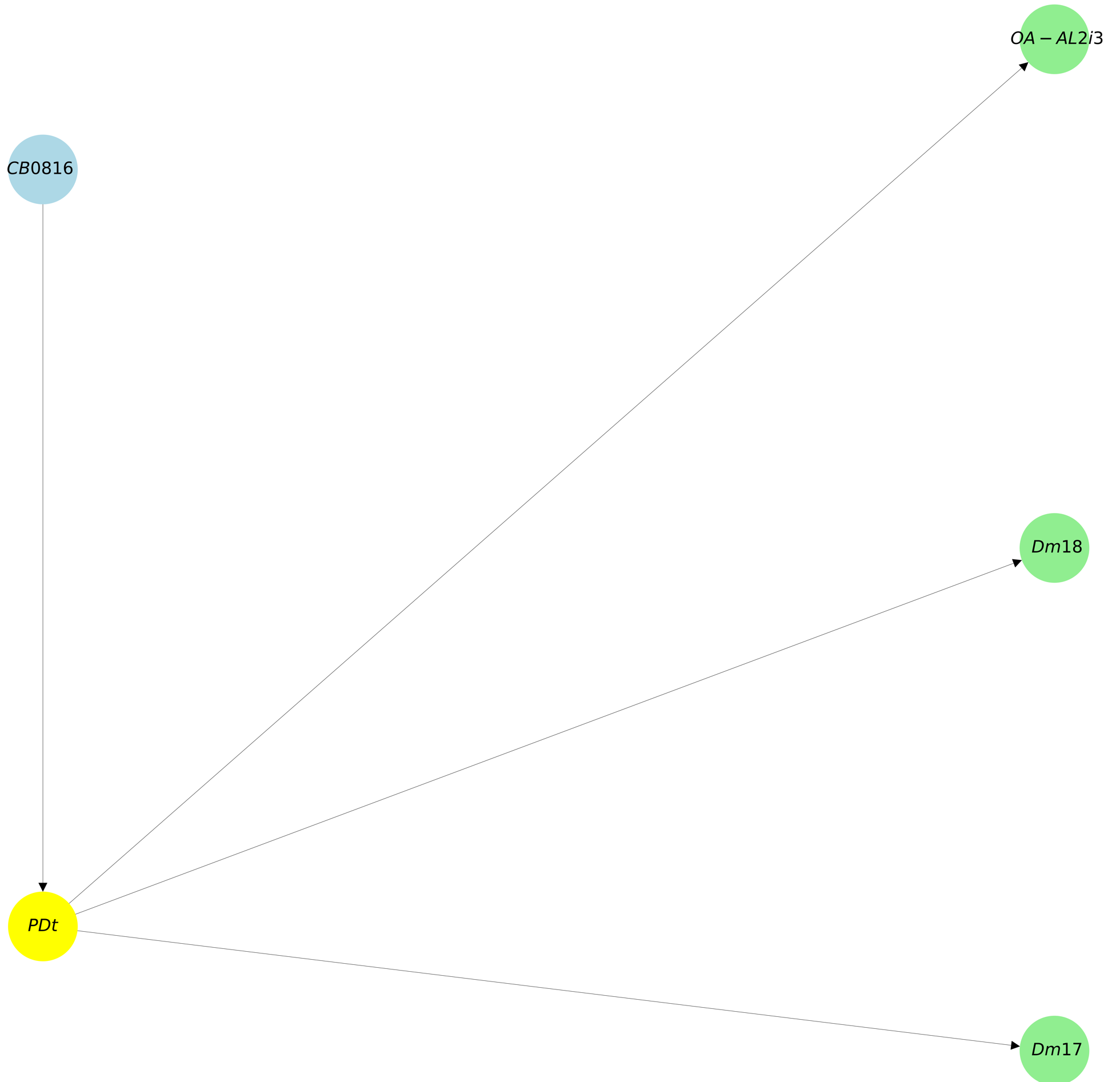

Supplement: Supplementary file 6 — Discriminating logical predicates for all types. Each figure contains types from the same family (middle layer) with shared input attributes (left layer) and output attributes (right layer) that are sufficient for discriminating all types in the middle layer. Families with many types are split into multiple figures for clarity of presentation. [file 41586_2024_7981_MOESM6_ESM.zip › DataS2/pdf/Proximal_Distal_Medulla_Tangential_Predicates.pdf]

Proximal Medulla Predicates (part 1 of 3)

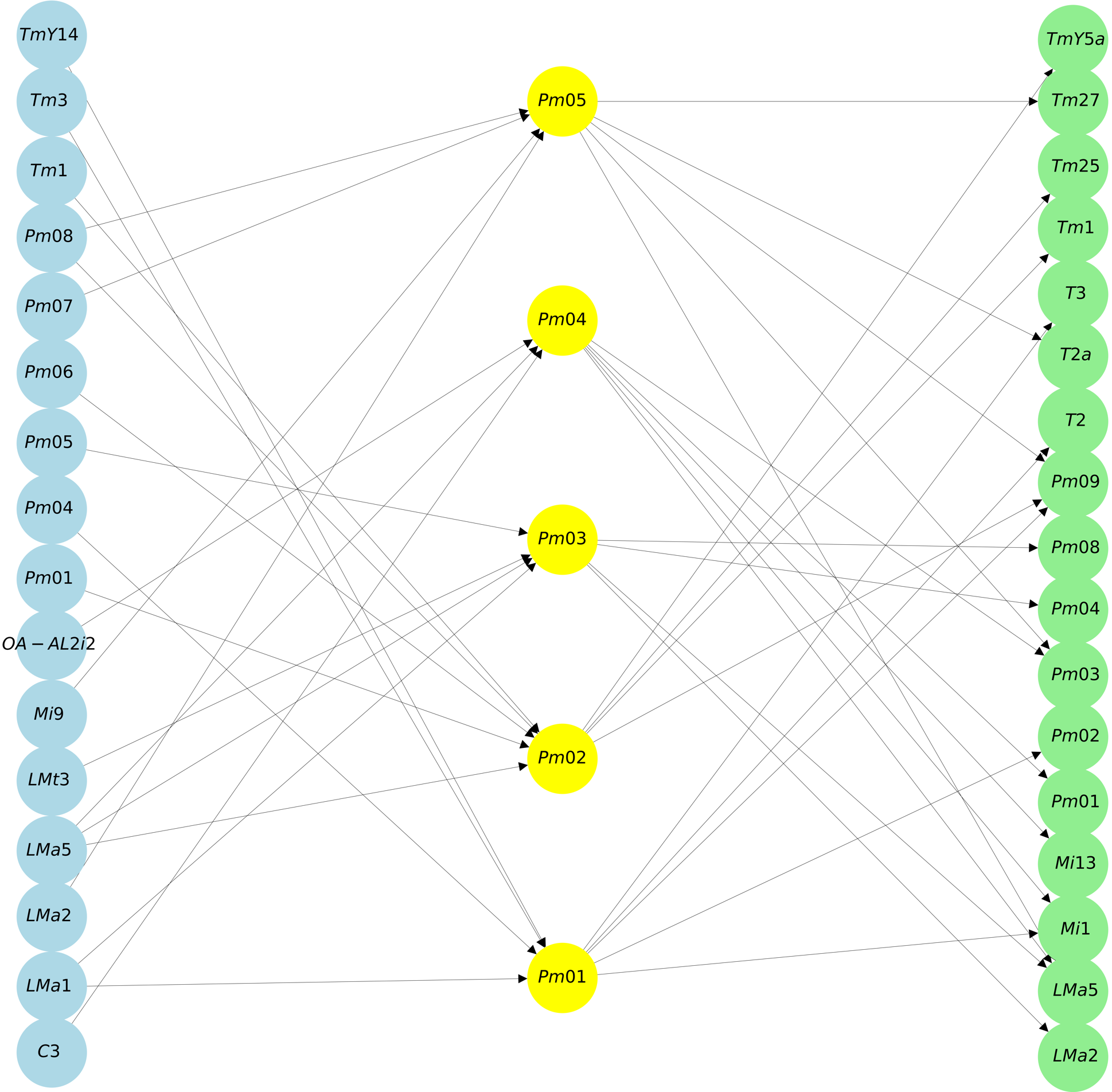

Supplement: Supplementary file 6 — Discriminating logical predicates for all types. Each figure contains types from the same family (middle layer) with shared input attributes (left layer) and output attributes (right layer) that are sufficient for discriminating all types in the middle layer. Families with many types are split into multiple figures for clarity of presentation. [file 41586_2024_7981_MOESM6_ESM.zip › DataS2/pdf/Proximal_Medulla_Predicates_(part_1_of_3).pdf]

Proximal Medulla Predicates (part 2 of 3)

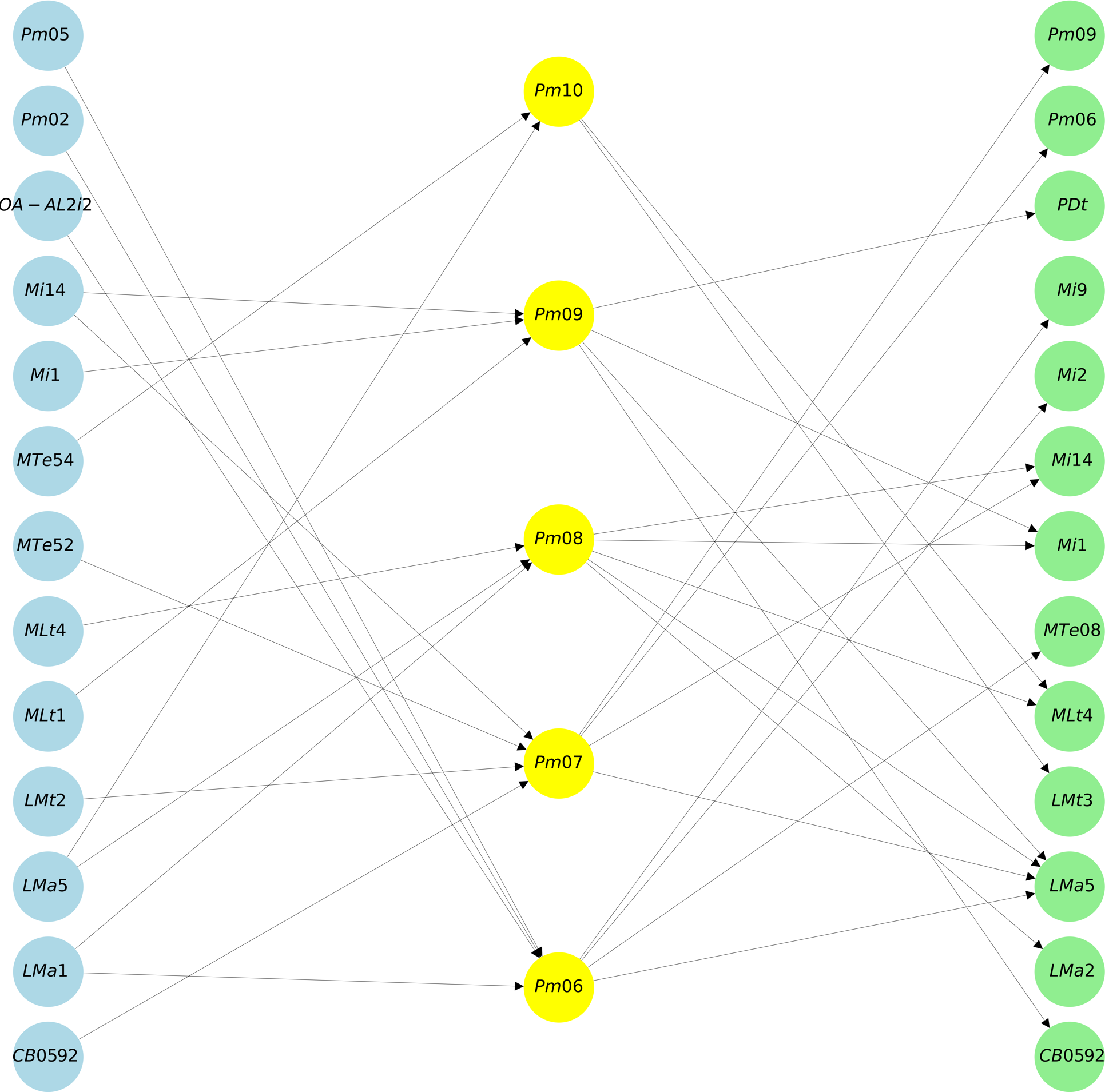

Supplement: Supplementary file 6 — Discriminating logical predicates for all types. Each figure contains types from the same family (middle layer) with shared input attributes (left layer) and output attributes (right layer) that are sufficient for discriminating all types in the middle layer. Families with many types are split into multiple figures for clarity of presentation. [file 41586_2024_7981_MOESM6_ESM.zip › DataS2/pdf/Proximal_Medulla_Predicates_(part_2_of_3).pdf]

Proximal Medulla Predicates (part 3 of 3)

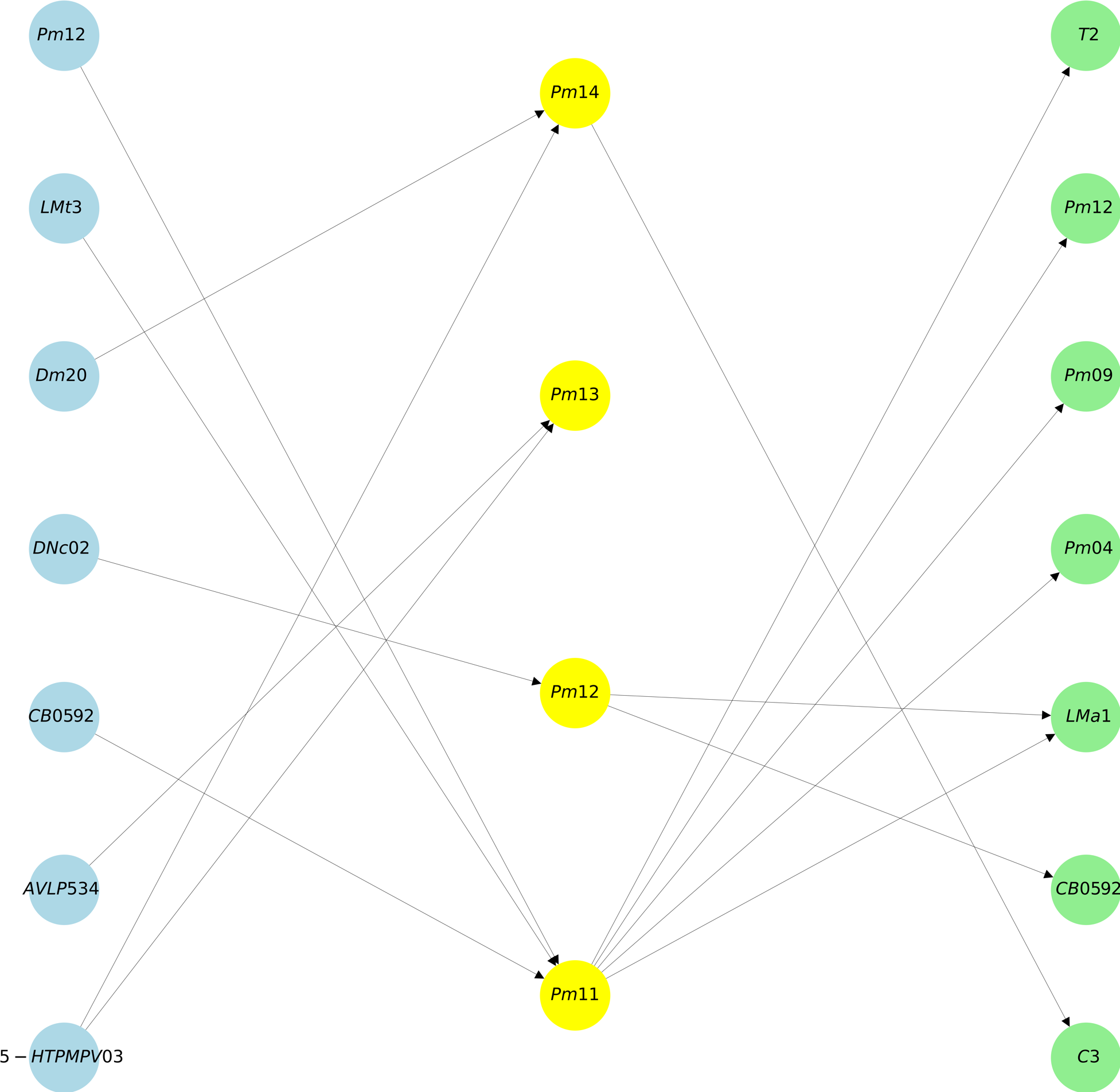

Supplement: Supplementary file 6 — Discriminating logical predicates for all types. Each figure contains types from the same family (middle layer) with shared input attributes (left layer) and output attributes (right layer) that are sufficient for discriminating all types in the middle layer. Families with many types are split into multiple figures for clarity of presentation. [file 41586_2024_7981_MOESM6_ESM.zip › DataS2/pdf/Proximal_Medulla_Predicates_(part_3_of_3).pdf]

Serpentine Medulla Predicates (part 1 of 9)

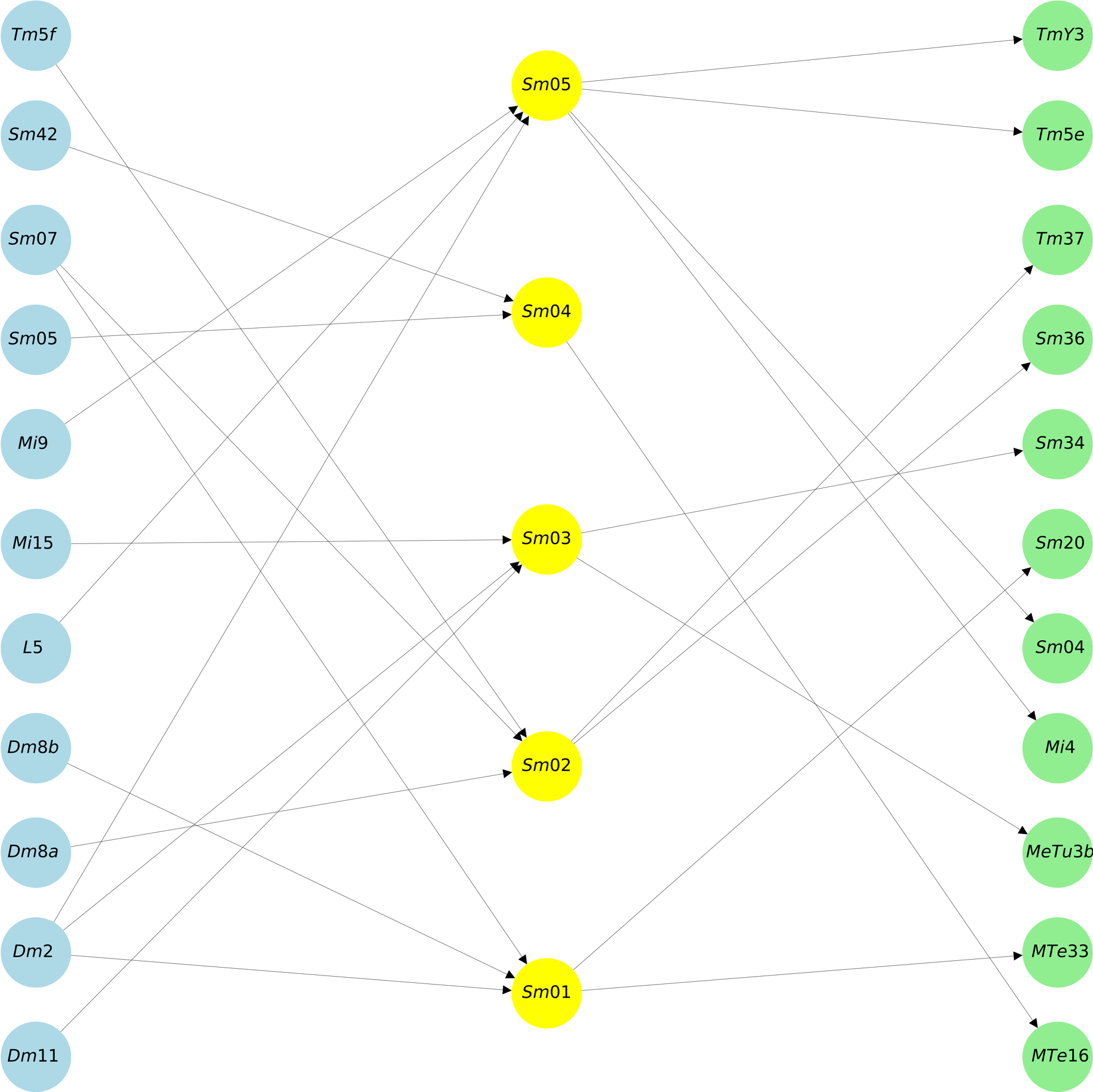

Supplement: Supplementary file 6 — Discriminating logical predicates for all types. Each figure contains types from the same family (middle layer) with shared input attributes (left layer) and output attributes (right layer) that are sufficient for discriminating all types in the middle layer. Families with many types are split into multiple figures for clarity of presentation. [file 41586_2024_7981_MOESM6_ESM.zip › DataS2/pdf/Serpentine_Medulla_Predicates_(part_1_of_9).pdf]

Serpentine Medulla Predicates (part 2 of 9)

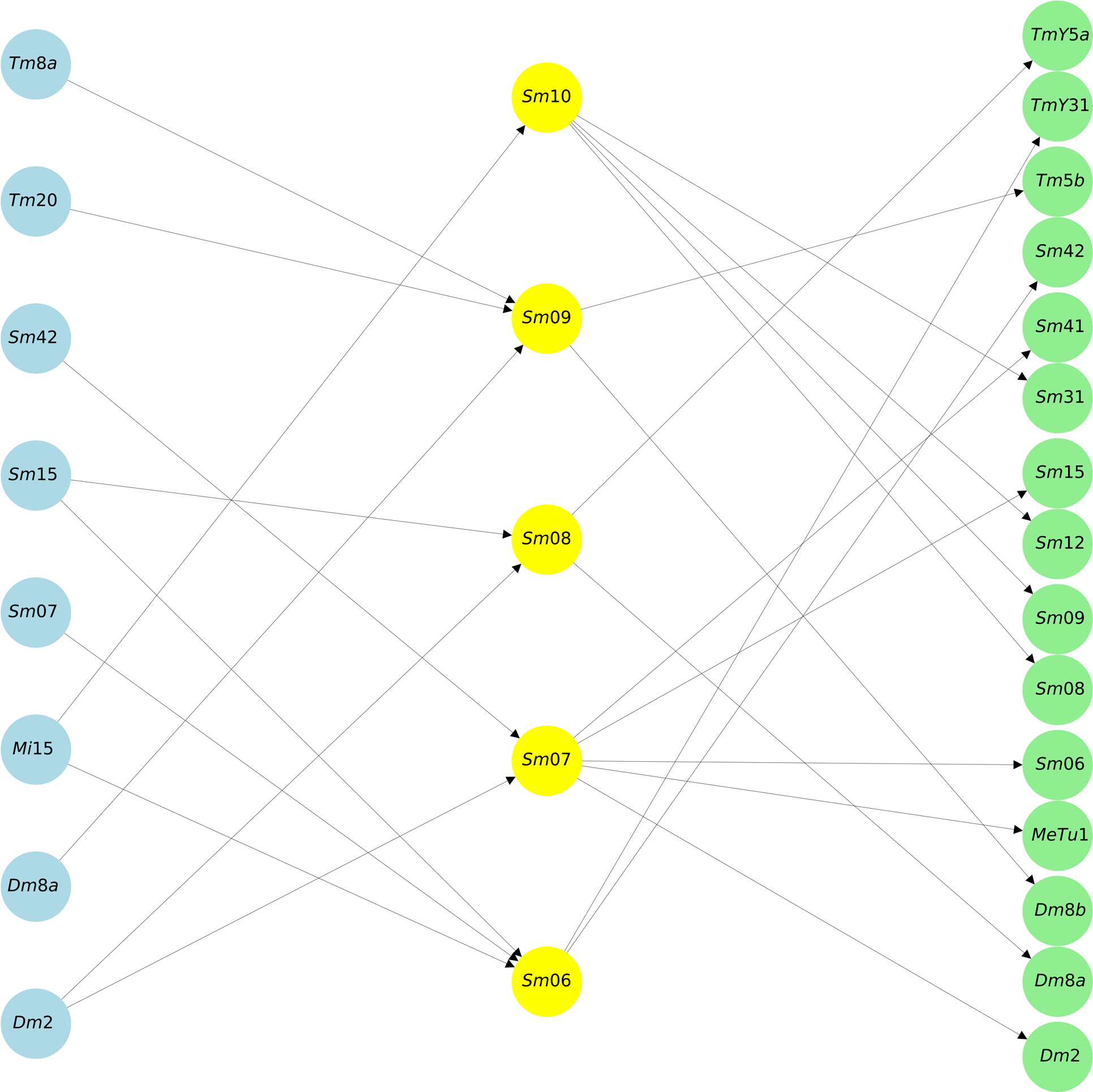

Supplement: Supplementary file 6 — Discriminating logical predicates for all types. Each figure contains types from the same family (middle layer) with shared input attributes (left layer) and output attributes (right layer) that are sufficient for discriminating all types in the middle layer. Families with many types are split into multiple figures for clarity of presentation. [file 41586_2024_7981_MOESM6_ESM.zip › DataS2/pdf/Serpentine_Medulla_Predicates_(part_2_of_9).pdf]

Serpentine Medulla Predicates (part 3 of 9)

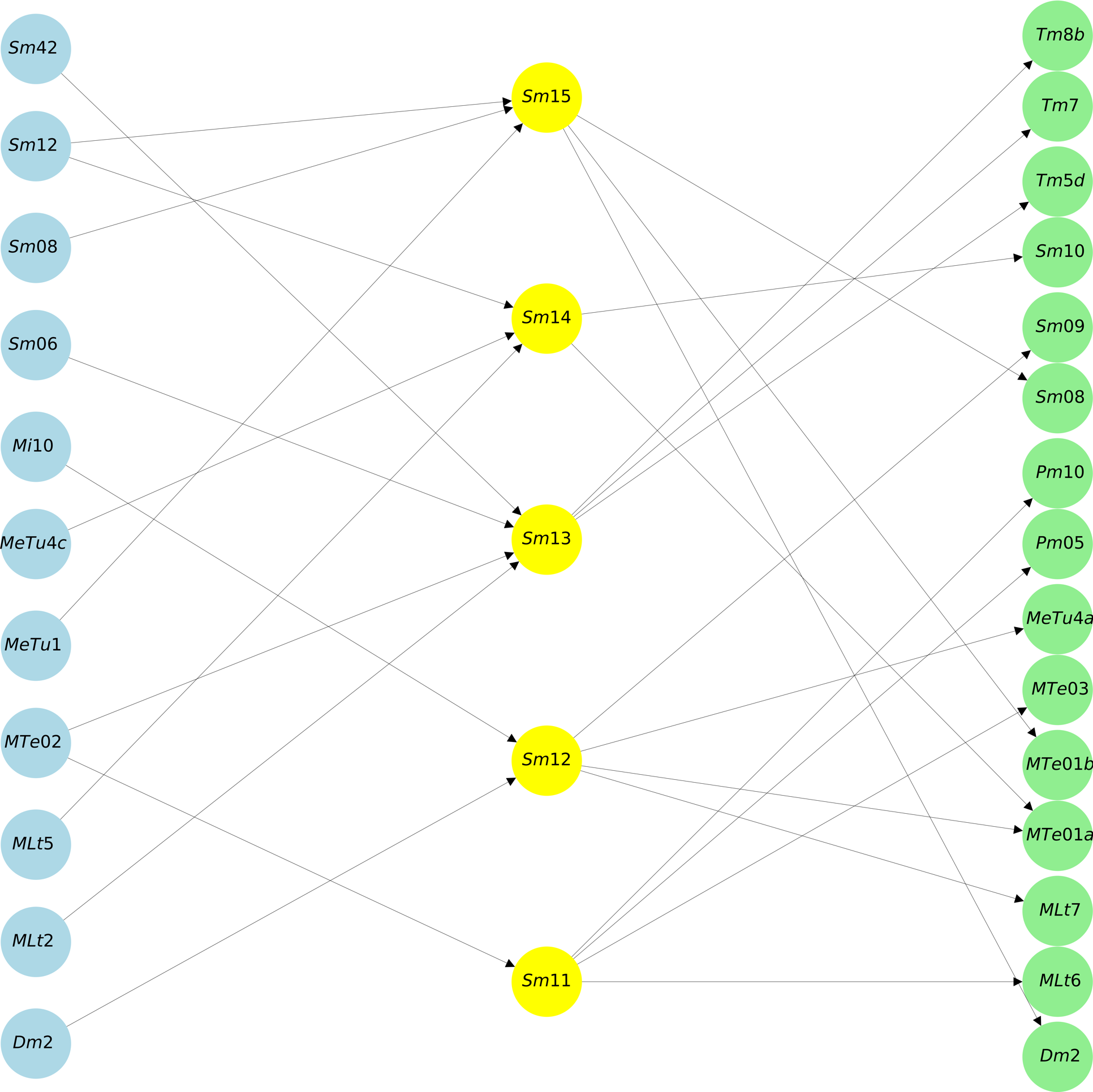

Supplement: Supplementary file 6 — Discriminating logical predicates for all types. Each figure contains types from the same family (middle layer) with shared input attributes (left layer) and output attributes (right layer) that are sufficient for discriminating all types in the middle layer. Families with many types are split into multiple figures for clarity of presentation. [file 41586_2024_7981_MOESM6_ESM.zip › DataS2/pdf/Serpentine_Medulla_Predicates_(part_3_of_9).pdf]

## Serpentine Medulla Predicates (part 4 of 9)

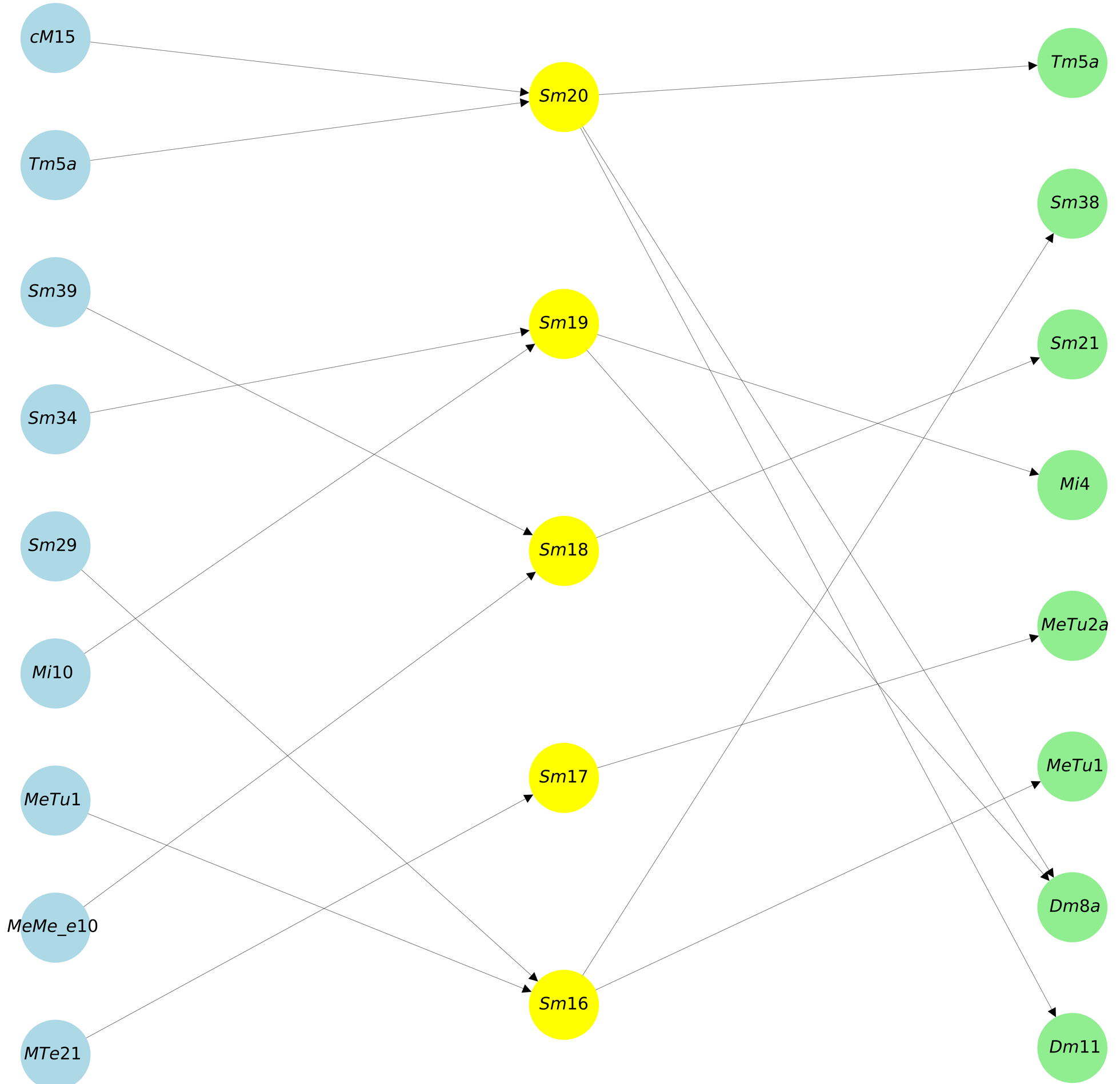

Supplement: Supplementary file 6 — Discriminating logical predicates for all types. Each figure contains types from the same family (middle layer) with shared input attributes (left layer) and output attributes (right layer) that are sufficient for discriminating all types in the middle layer. Families with many types are split into multiple figures for clarity of presentation. [file 41586_2024_7981_MOESM6_ESM.zip › DataS2/pdf/Serpentine_Medulla_Predicates_(part_4_of_9).pdf]

## Serpentine Medulla Predicates (part 5 of 9)

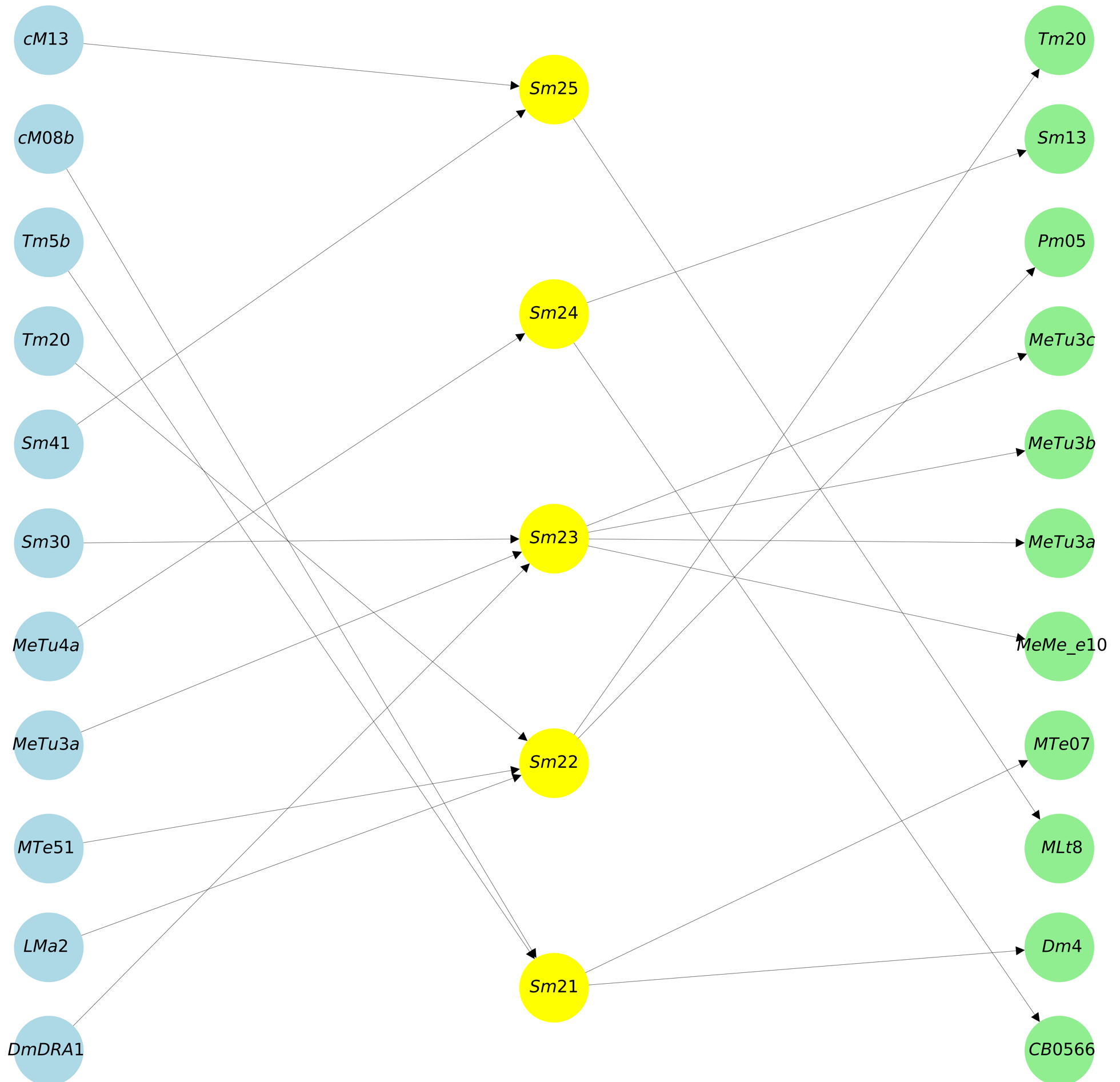

Supplement: Supplementary file 6 — Discriminating logical predicates for all types. Each figure contains types from the same family (middle layer) with shared input attributes (left layer) and output attributes (right layer) that are sufficient for discriminating all types in the middle layer. Families with many types are split into multiple figures for clarity of presentation. [file 41586_2024_7981_MOESM6_ESM.zip › DataS2/pdf/Serpentine_Medulla_Predicates_(part_5_of_9).pdf]

Serpentine Medulla Predicates (part 6 of 9)

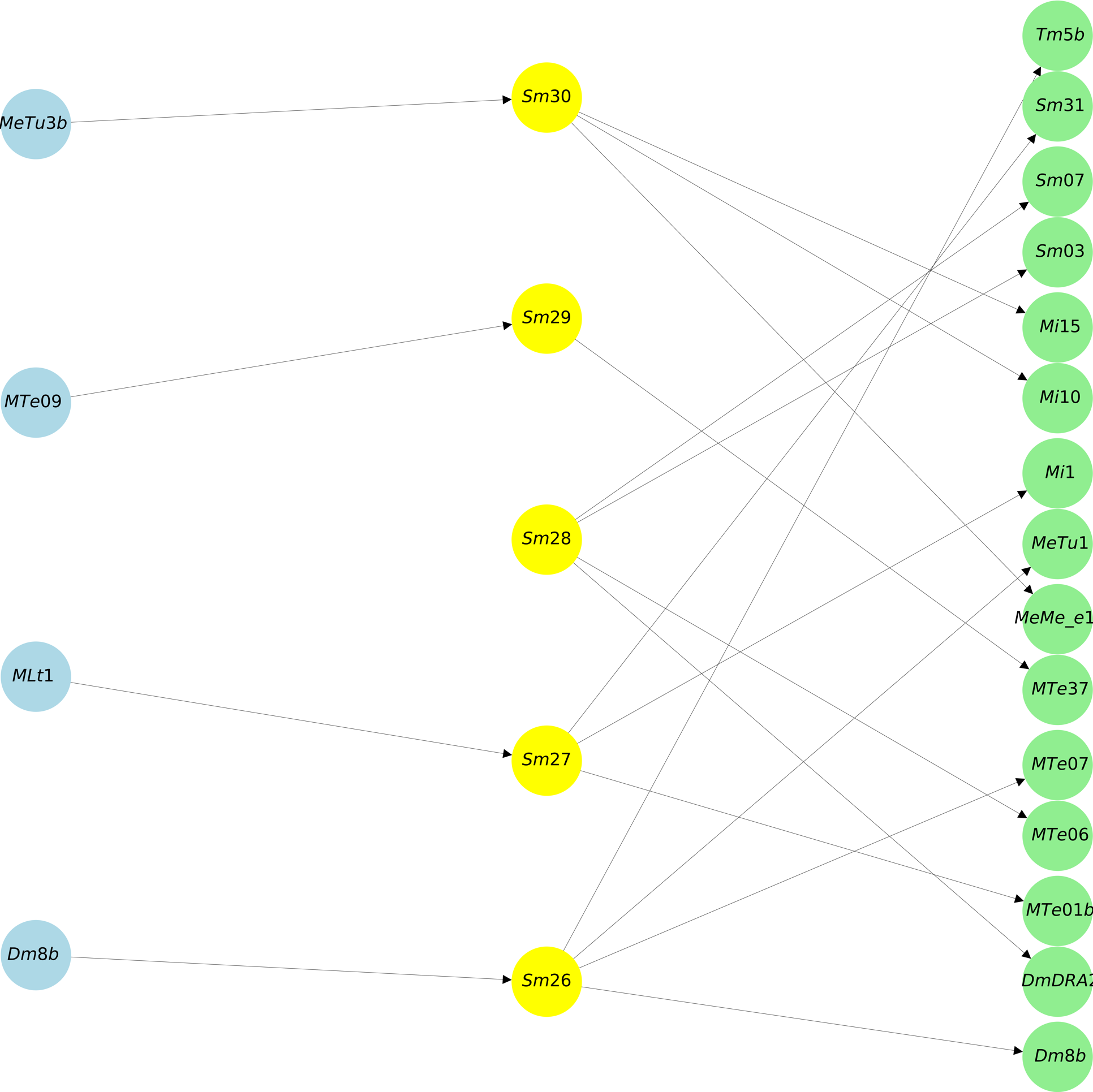

Supplement: Supplementary file 6 — Discriminating logical predicates for all types. Each figure contains types from the same family (middle layer) with shared input attributes (left layer) and output attributes (right layer) that are sufficient for discriminating all types in the middle layer. Families with many types are split into multiple figures for clarity of presentation. [file 41586_2024_7981_MOESM6_ESM.zip › DataS2/pdf/Serpentine_Medulla_Predicates_(part_6_of_9).pdf]

Serpentine Medulla Predicates (part 7 of 9)

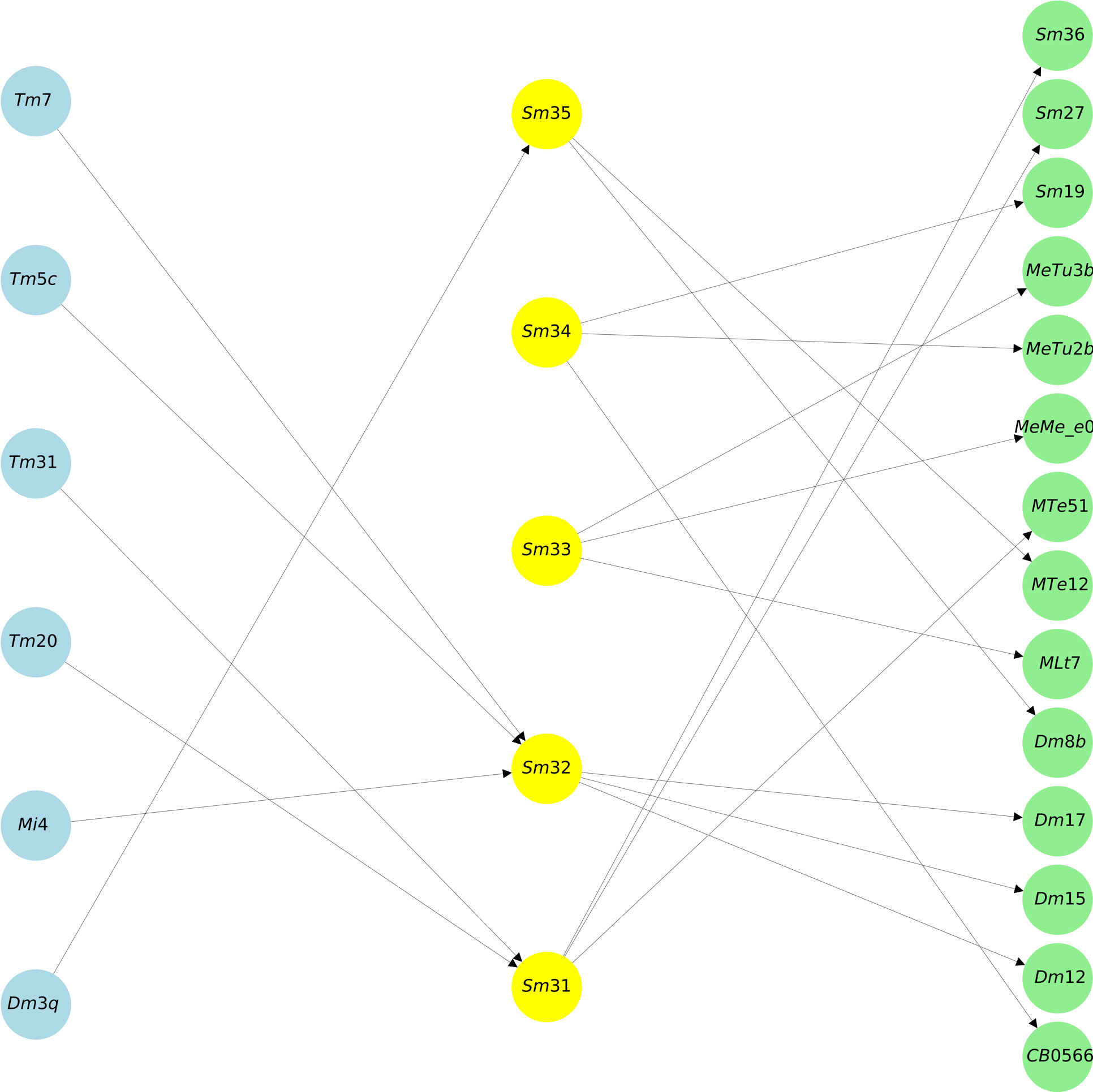

Supplement: Supplementary file 6 — Discriminating logical predicates for all types. Each figure contains types from the same family (middle layer) with shared input attributes (left layer) and output attributes (right layer) that are sufficient for discriminating all types in the middle layer. Families with many types are split into multiple figures for clarity of presentation. [file 41586_2024_7981_MOESM6_ESM.zip › DataS2/pdf/Serpentine_Medulla_Predicates_(part_7_of_9).pdf]

Serpentine Medulla Predicates (part 8 of 9)

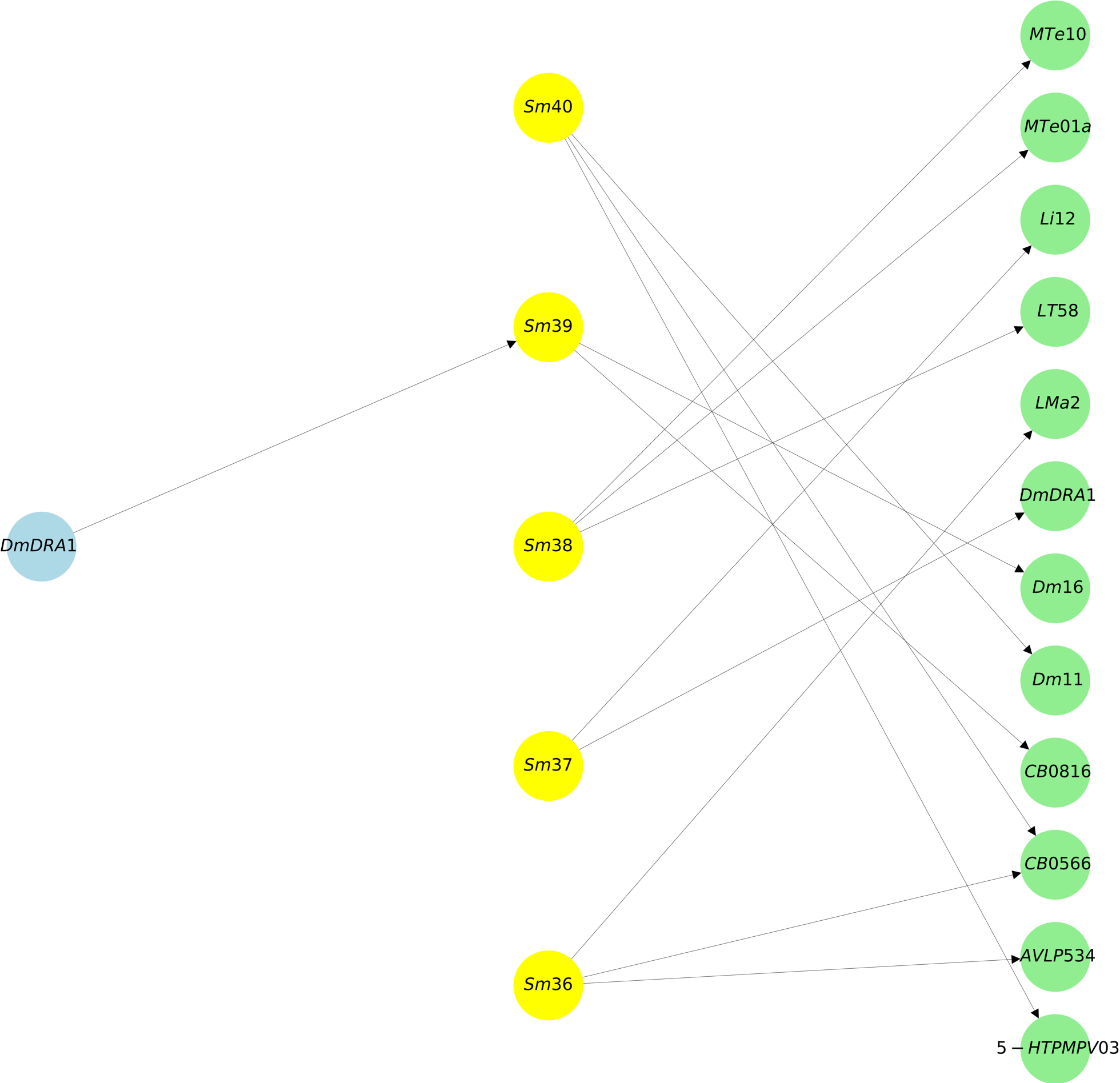

Supplement: Supplementary file 6 — Discriminating logical predicates for all types. Each figure contains types from the same family (middle layer) with shared input attributes (left layer) and output attributes (right layer) that are sufficient for discriminating all types in the middle layer. Families with many types are split into multiple figures for clarity of presentation. [file 41586_2024_7981_MOESM6_ESM.zip › DataS2/pdf/Serpentine_Medulla_Predicates_(part_8_of_9).pdf]

Serpentine Medulla Predicates (part 9 of 9)

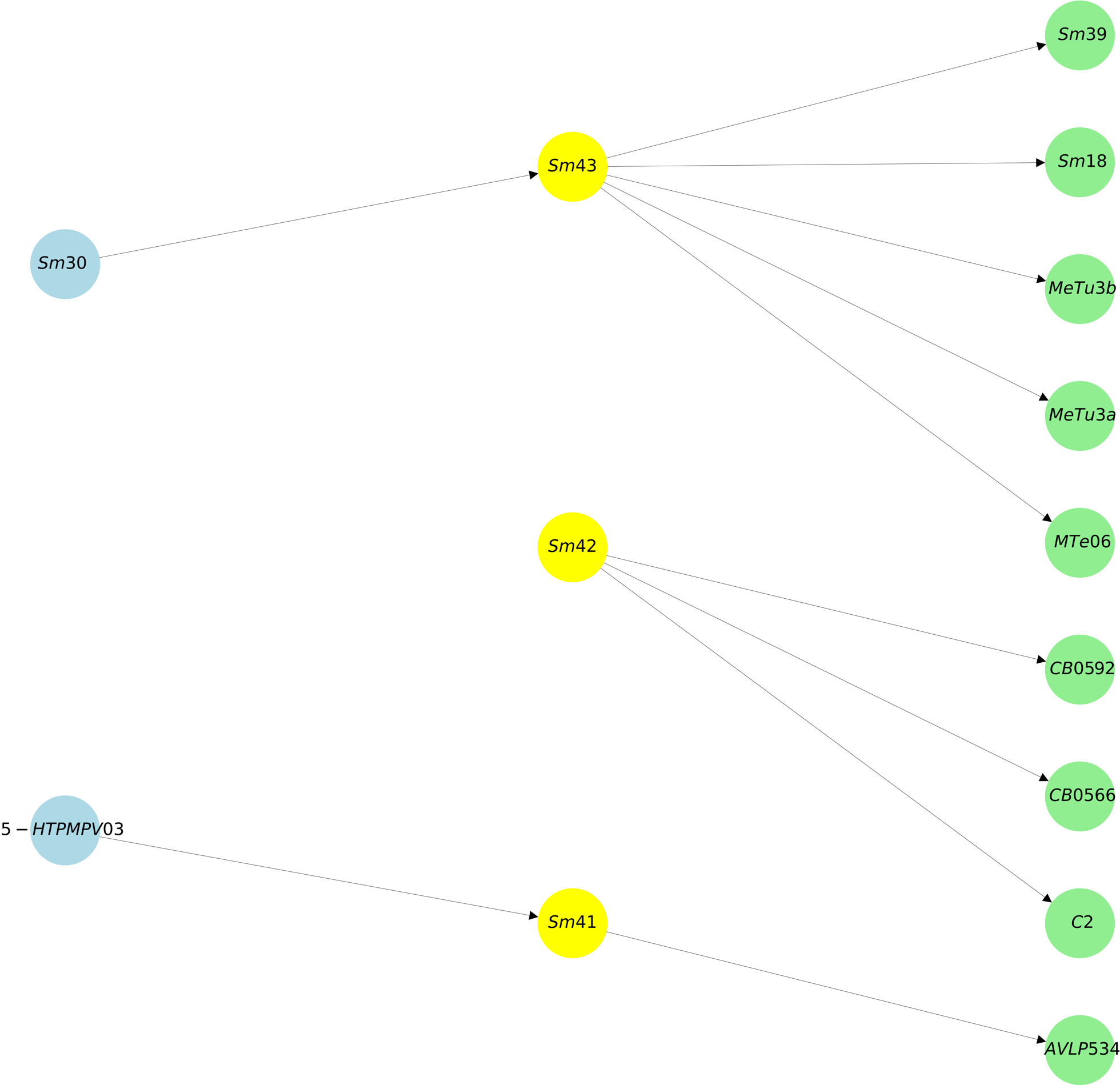

Supplement: Supplementary file 6 — Discriminating logical predicates for all types. Each figure contains types from the same family (middle layer) with shared input attributes (left layer) and output attributes (right layer) that are sufficient for discriminating all types in the middle layer. Families with many types are split into multiple figures for clarity of presentation. [file 41586_2024_7981_MOESM6_ESM.zip › DataS2/pdf/Serpentine_Medulla_Predicates_(part_9_of_9).pdf]

## T Neuron Predicates (part 1 of 3)

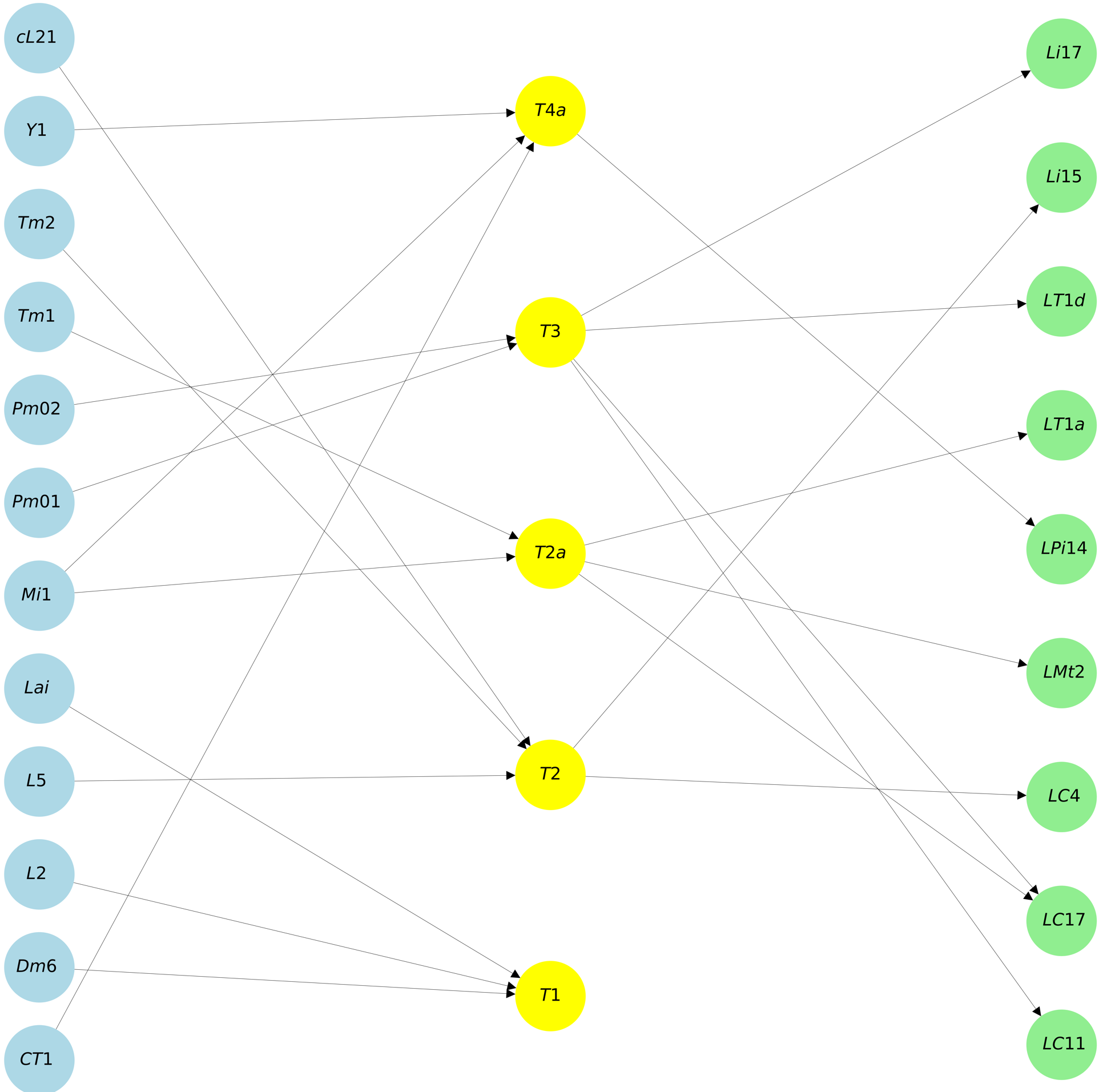

Supplement: Supplementary file 6 — Discriminating logical predicates for all types. Each figure contains types from the same family (middle layer) with shared input attributes (left layer) and output attributes (right layer) that are sufficient for discriminating all types in the middle layer. Families with many types are split into multiple figures for clarity of presentation. [file 41586_2024_7981_MOESM6_ESM.zip › DataS2/pdf/T_Neuron_Predicates_(part_1_of_3).pdf]

## T Neuron Predicates (part 2 of 3)

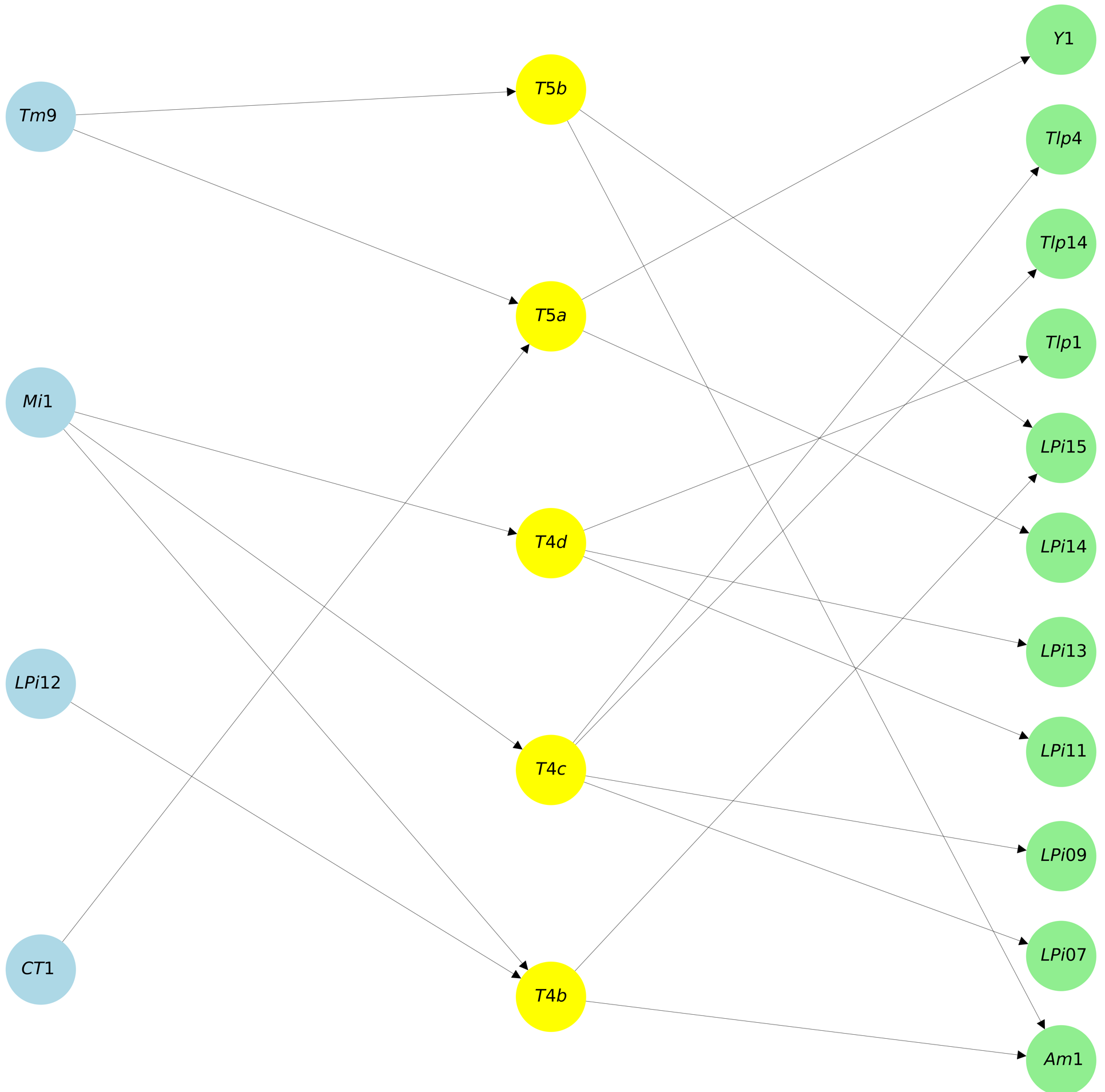

Supplement: Supplementary file 6 — Discriminating logical predicates for all types. Each figure contains types from the same family (middle layer) with shared input attributes (left layer) and output attributes (right layer) that are sufficient for discriminating all types in the middle layer. Families with many types are split into multiple figures for clarity of presentation. [file 41586_2024_7981_MOESM6_ESM.zip › DataS2/pdf/T_Neuron_Predicates_(part_2_of_3).pdf]

### T Neuron Predicates (part 3 of 3)

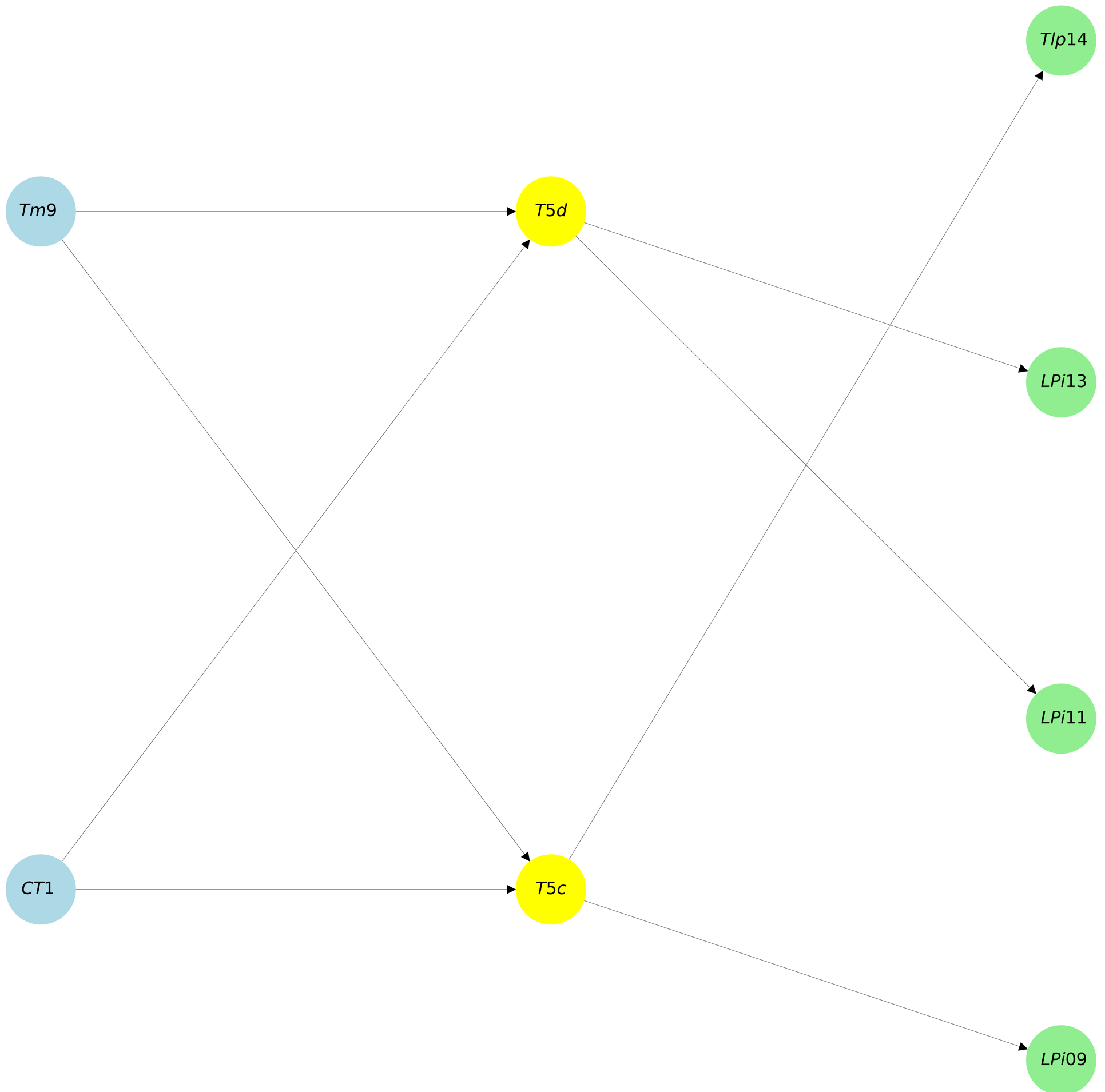

Supplement: Supplementary file 6 — Discriminating logical predicates for all types. Each figure contains types from the same family (middle layer) with shared input attributes (left layer) and output attributes (right layer) that are sufficient for discriminating all types in the middle layer. Families with many types are split into multiple figures for clarity of presentation. [file 41586_2024_7981_MOESM6_ESM.zip › DataS2/pdf/T_Neuron_Predicates_(part_3_of_3).pdf]

## Translobula Plate Predicates

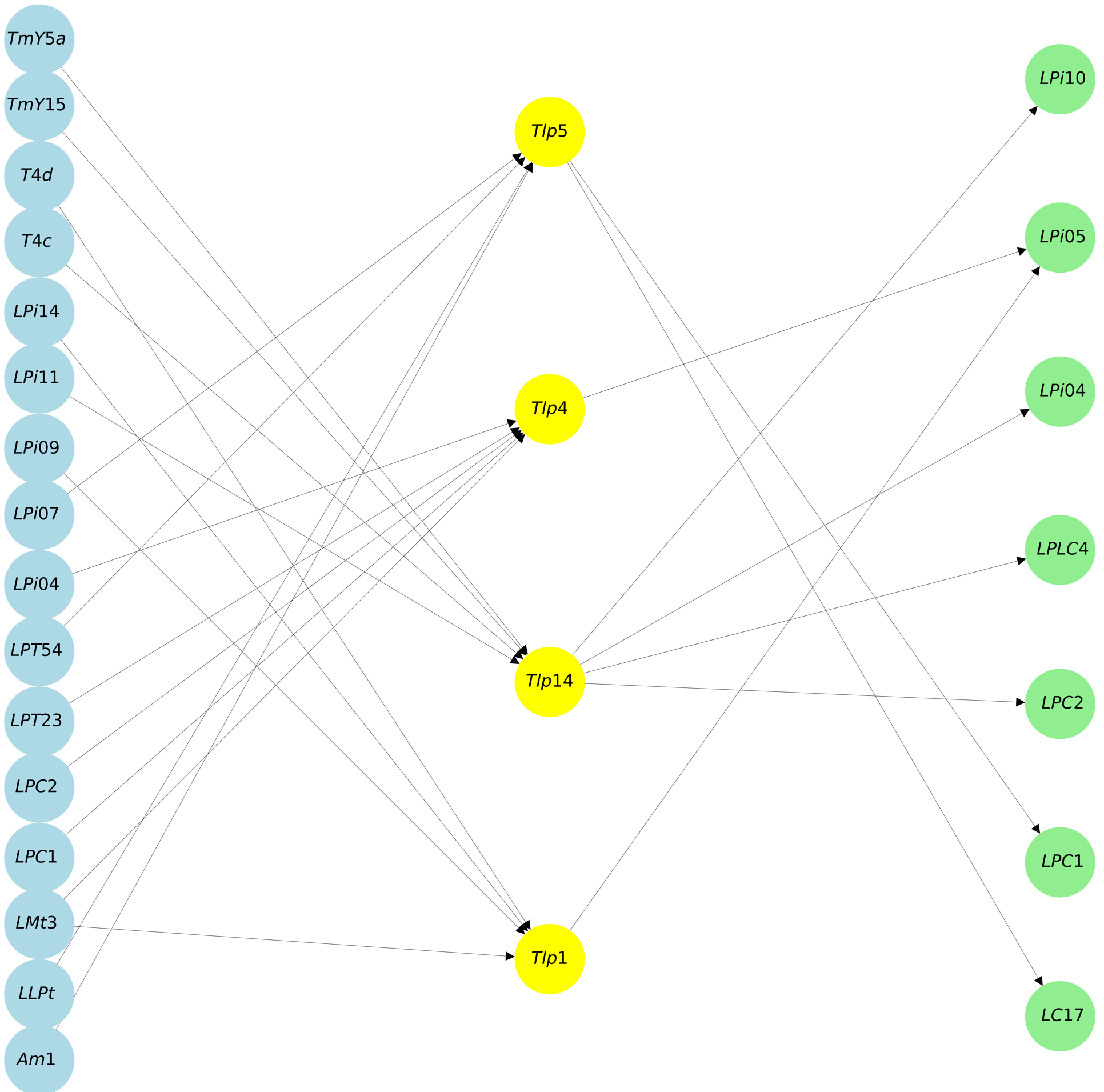

Supplement: Supplementary file 6 — Discriminating logical predicates for all types. Each figure contains types from the same family (middle layer) with shared input attributes (left layer) and output attributes (right layer) that are sufficient for discriminating all types in the middle layer. Families with many types are split into multiple figures for clarity of presentation. [file 41586_2024_7981_MOESM6_ESM.zip › DataS2/pdf/Translobula_Plate_Predicates.pdf]

## Transmedullary Predicates (part 1 of 6)

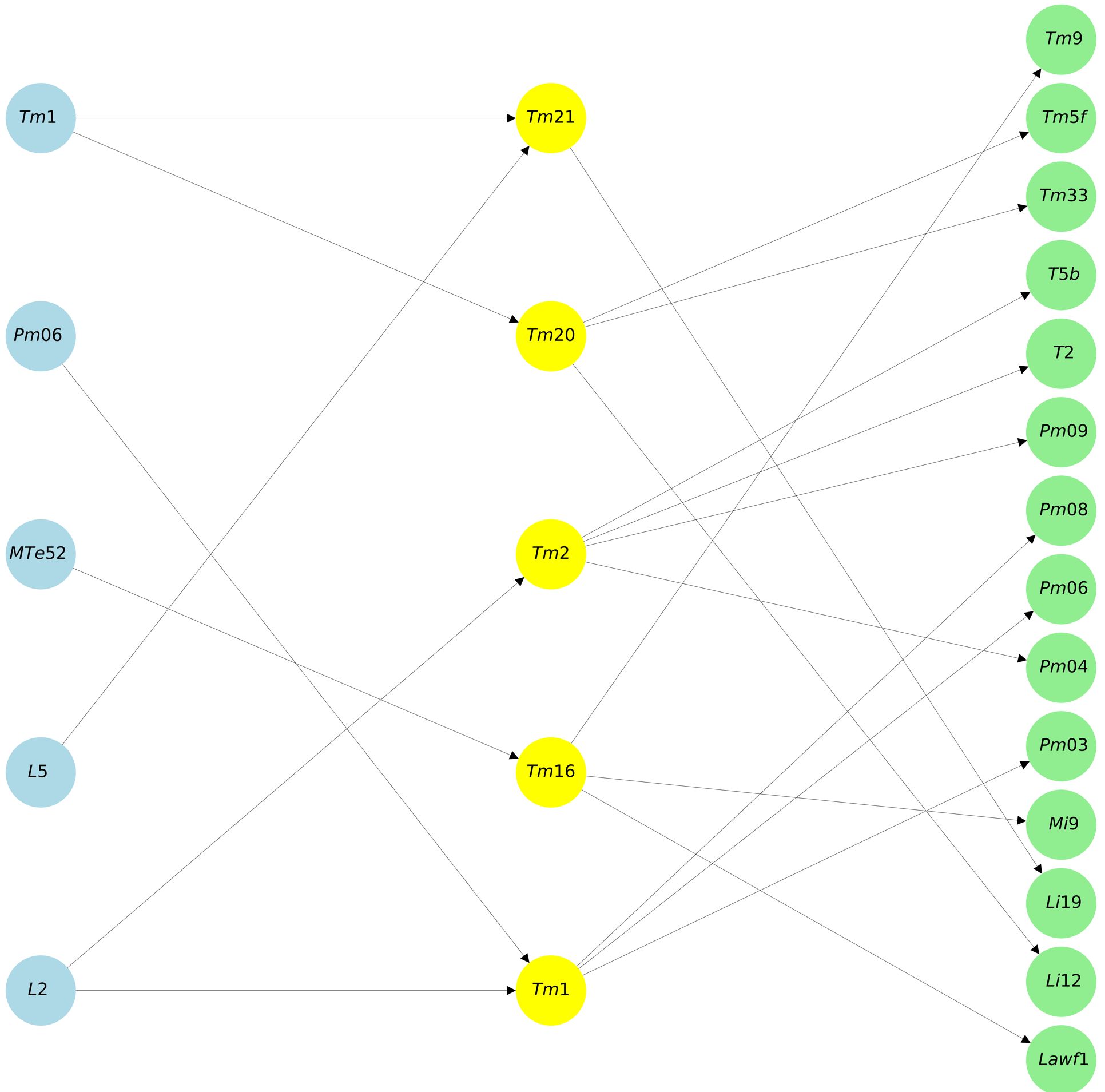

Supplement: Supplementary file 6 — Discriminating logical predicates for all types. Each figure contains types from the same family (middle layer) with shared input attributes (left layer) and output attributes (right layer) that are sufficient for discriminating all types in the middle layer. Families with many types are split into multiple figures for clarity of presentation. [file 41586_2024_7981_MOESM6_ESM.zip › DataS2/pdf/Transmedullary_Predicates_(part_1_of_6).pdf]

## Transmedullary Predicates (part 2 of 6)

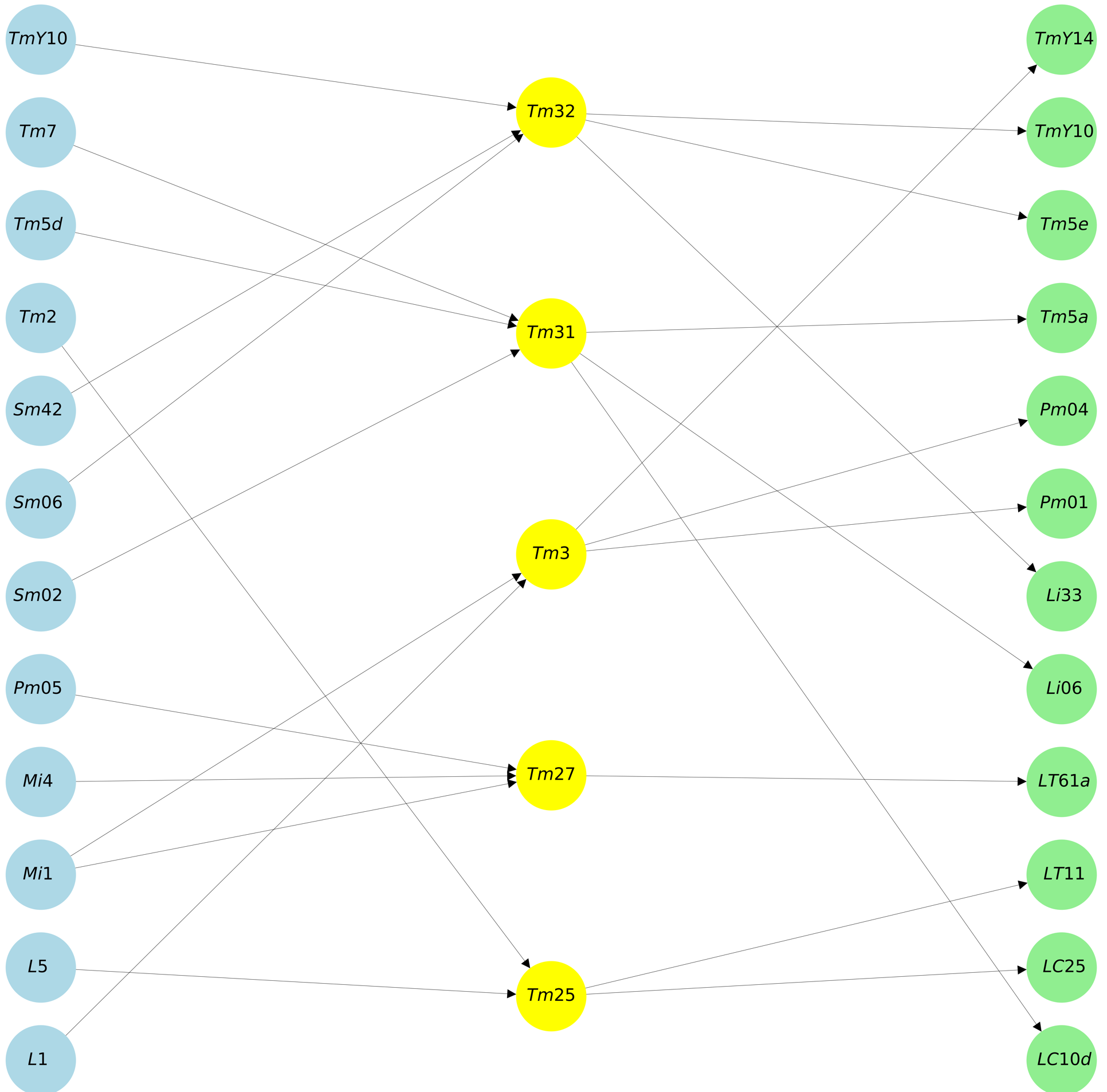

Supplement: Supplementary file 6 — Discriminating logical predicates for all types. Each figure contains types from the same family (middle layer) with shared input attributes (left layer) and output attributes (right layer) that are sufficient for discriminating all types in the middle layer. Families with many types are split into multiple figures for clarity of presentation. [file 41586_2024_7981_MOESM6_ESM.zip › DataS2/pdf/Transmedullary_Predicates_(part_2_of_6).pdf]

## Transmedullary Predicates (part 3 of 6)

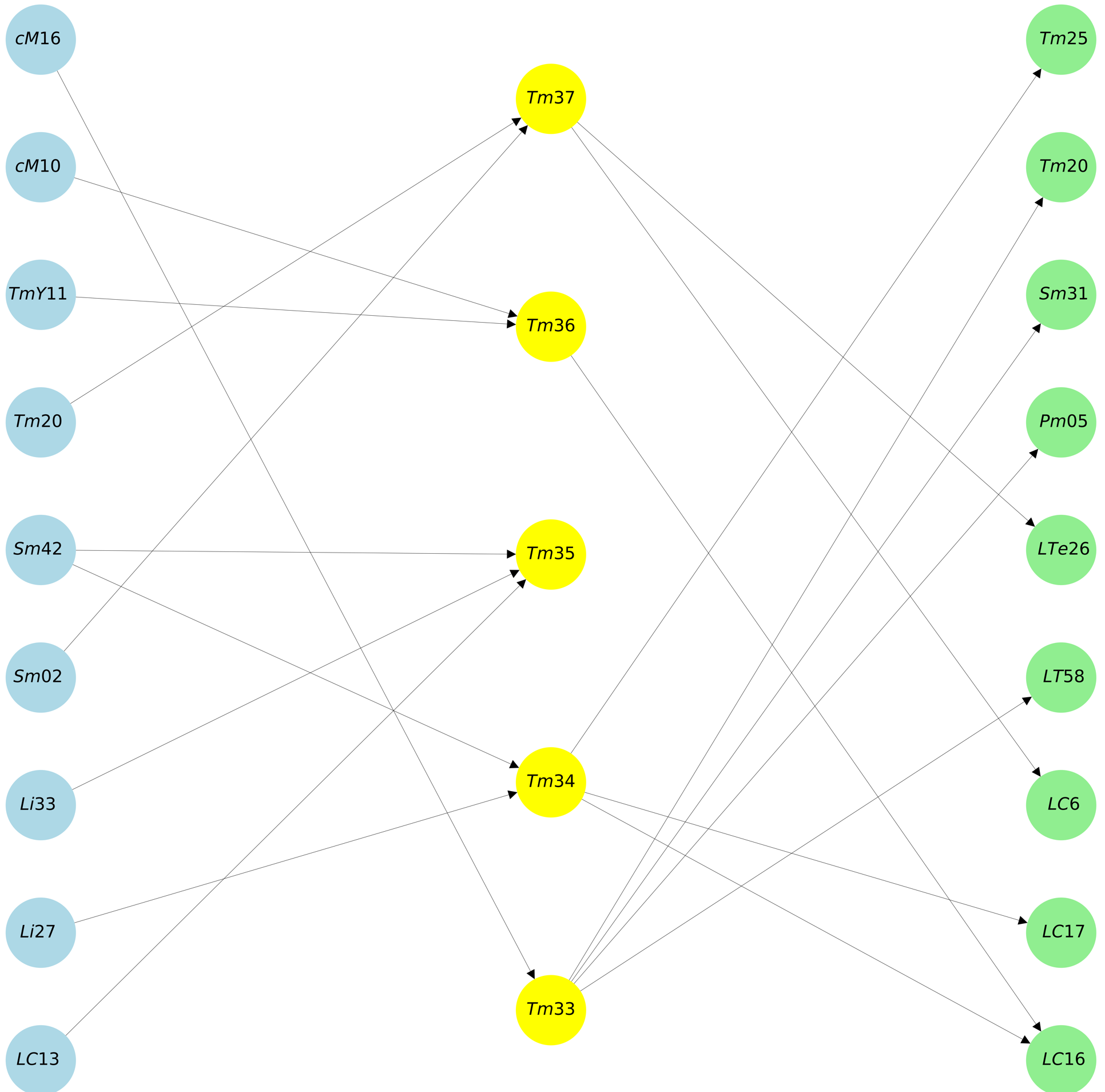

Supplement: Supplementary file 6 — Discriminating logical predicates for all types. Each figure contains types from the same family (middle layer) with shared input attributes (left layer) and output attributes (right layer) that are sufficient for discriminating all types in the middle layer. Families with many types are split into multiple figures for clarity of presentation. [file 41586_2024_7981_MOESM6_ESM.zip › DataS2/pdf/Transmedullary_Predicates_(part_3_of_6).pdf]

## Transmedullary Predicates (part 4 of 6)

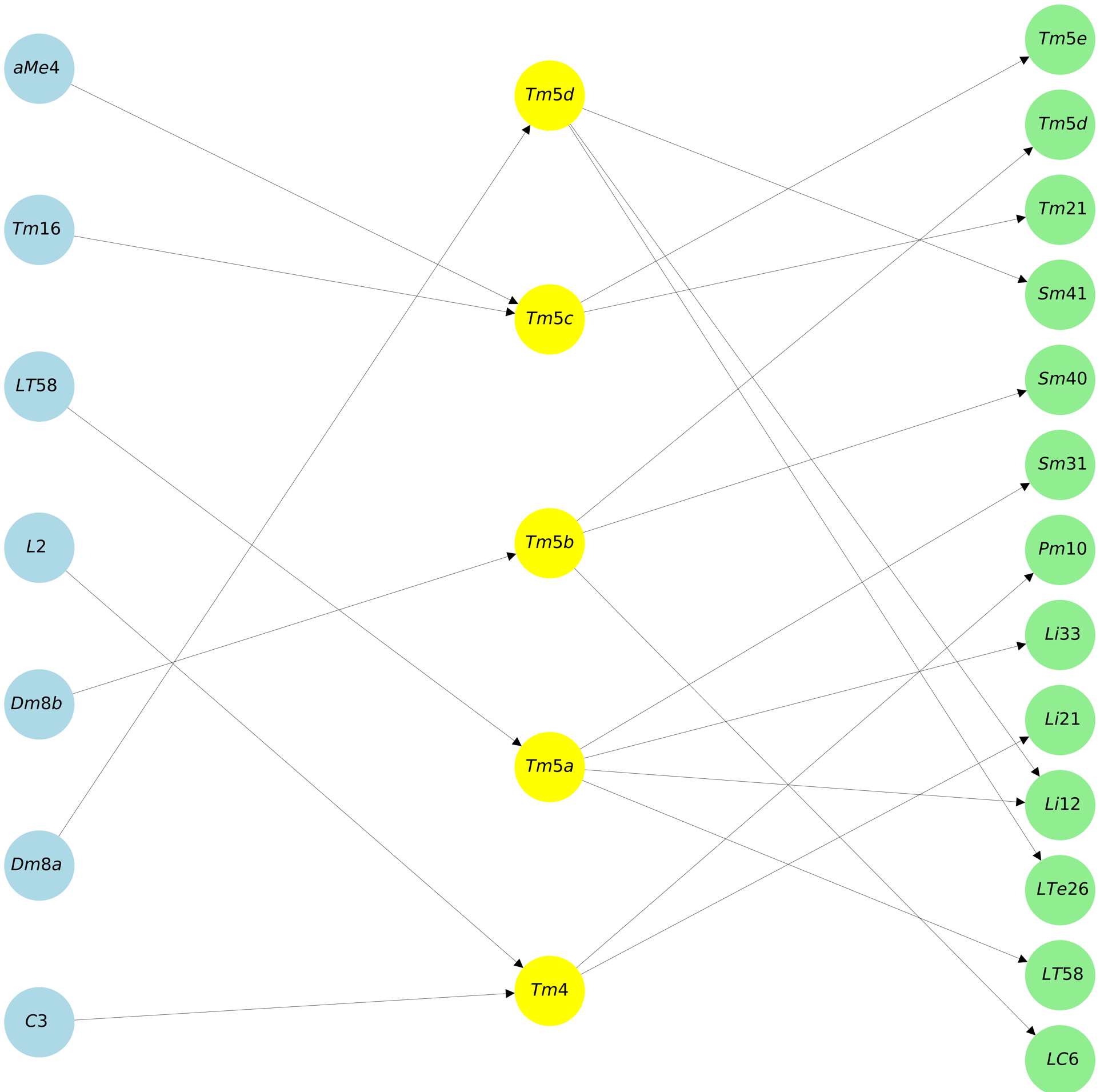

Supplement: Supplementary file 6 — Discriminating logical predicates for all types. Each figure contains types from the same family (middle layer) with shared input attributes (left layer) and output attributes (right layer) that are sufficient for discriminating all types in the middle layer. Families with many types are split into multiple figures for clarity of presentation. [file 41586_2024_7981_MOESM6_ESM.zip › DataS2/pdf/Transmedullary_Predicates_(part_4_of_6).pdf]

## Transmedullary Predicates (part 5 of 6)

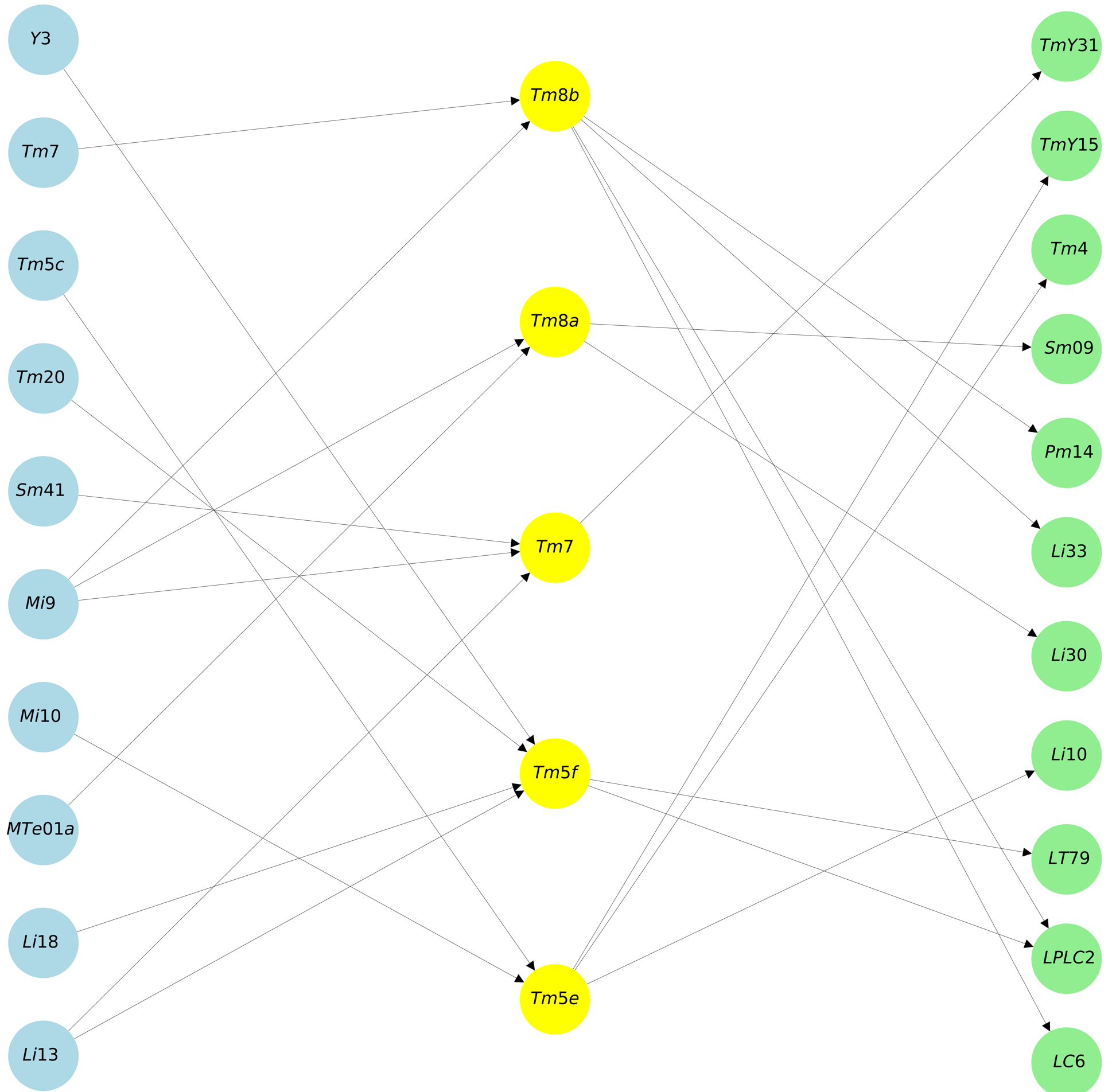

Supplement: Supplementary file 6 — Discriminating logical predicates for all types. Each figure contains types from the same family (middle layer) with shared input attributes (left layer) and output attributes (right layer) that are sufficient for discriminating all types in the middle layer. Families with many types are split into multiple figures for clarity of presentation. [file 41586_2024_7981_MOESM6_ESM.zip › DataS2/pdf/Transmedullary_Predicates_(part_5_of_6).pdf]

Transmedullary Predicates (part 6 of 6)

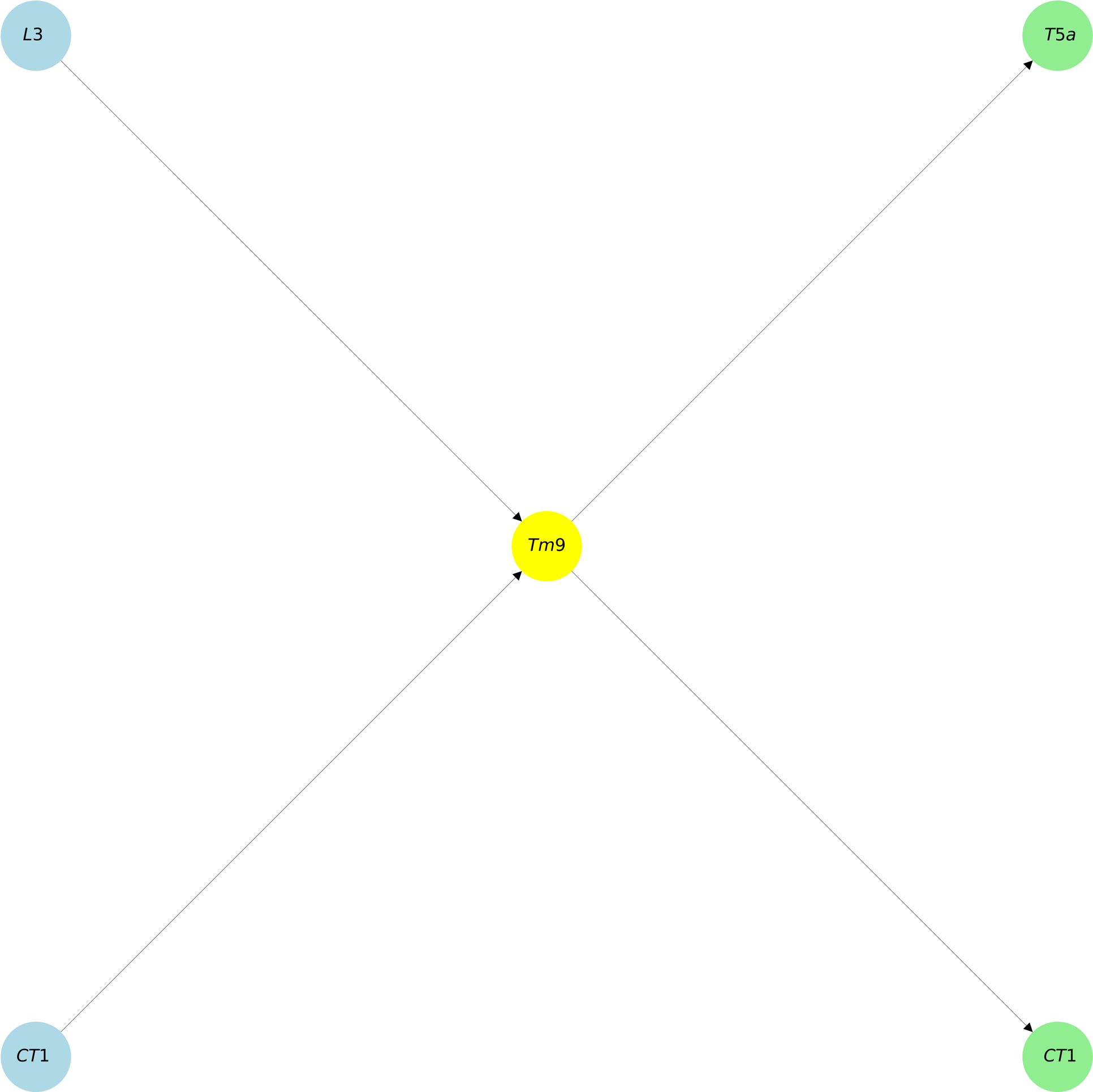

Supplement: Supplementary file 6 — Discriminating logical predicates for all types. Each figure contains types from the same family (middle layer) with shared input attributes (left layer) and output attributes (right layer) that are sufficient for discriminating all types in the middle layer. Families with many types are split into multiple figures for clarity of presentation. [file 41586_2024_7981_MOESM6_ESM.zip › DataS2/pdf/Transmedullary_Predicates_(part_6_of_6).pdf]

## Transmedullary Y Predicates (part 1 of 3)

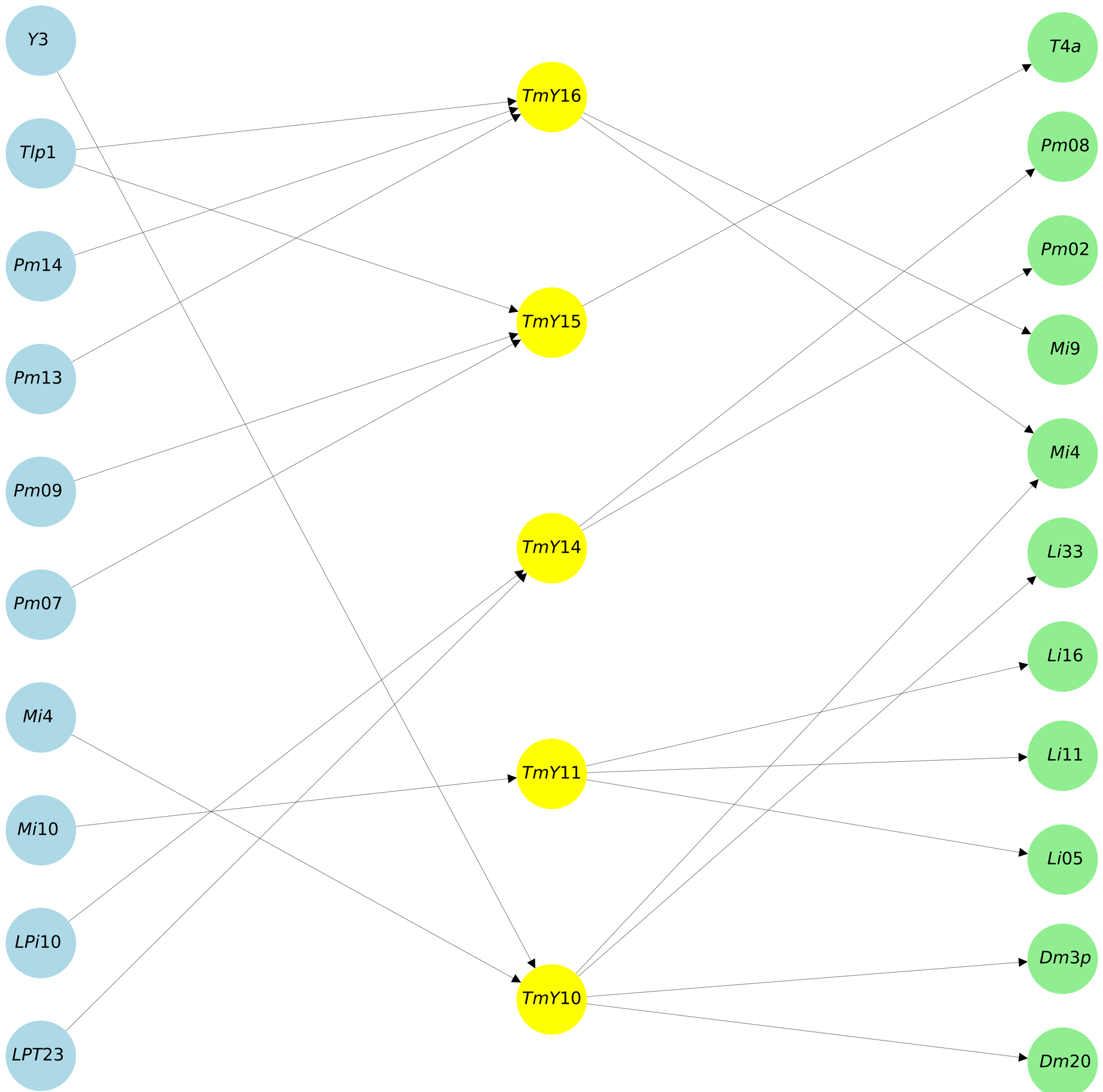

Supplement: Supplementary file 6 — Discriminating logical predicates for all types. Each figure contains types from the same family (middle layer) with shared input attributes (left layer) and output attributes (right layer) that are sufficient for discriminating all types in the middle layer. Families with many types are split into multiple figures for clarity of presentation. [file 41586_2024_7981_MOESM6_ESM.zip › DataS2/pdf/Transmedullary_Y_Predicates_(part_1_of_3).pdf]

## Transmedullary Y Predicates (part 2 of 3)

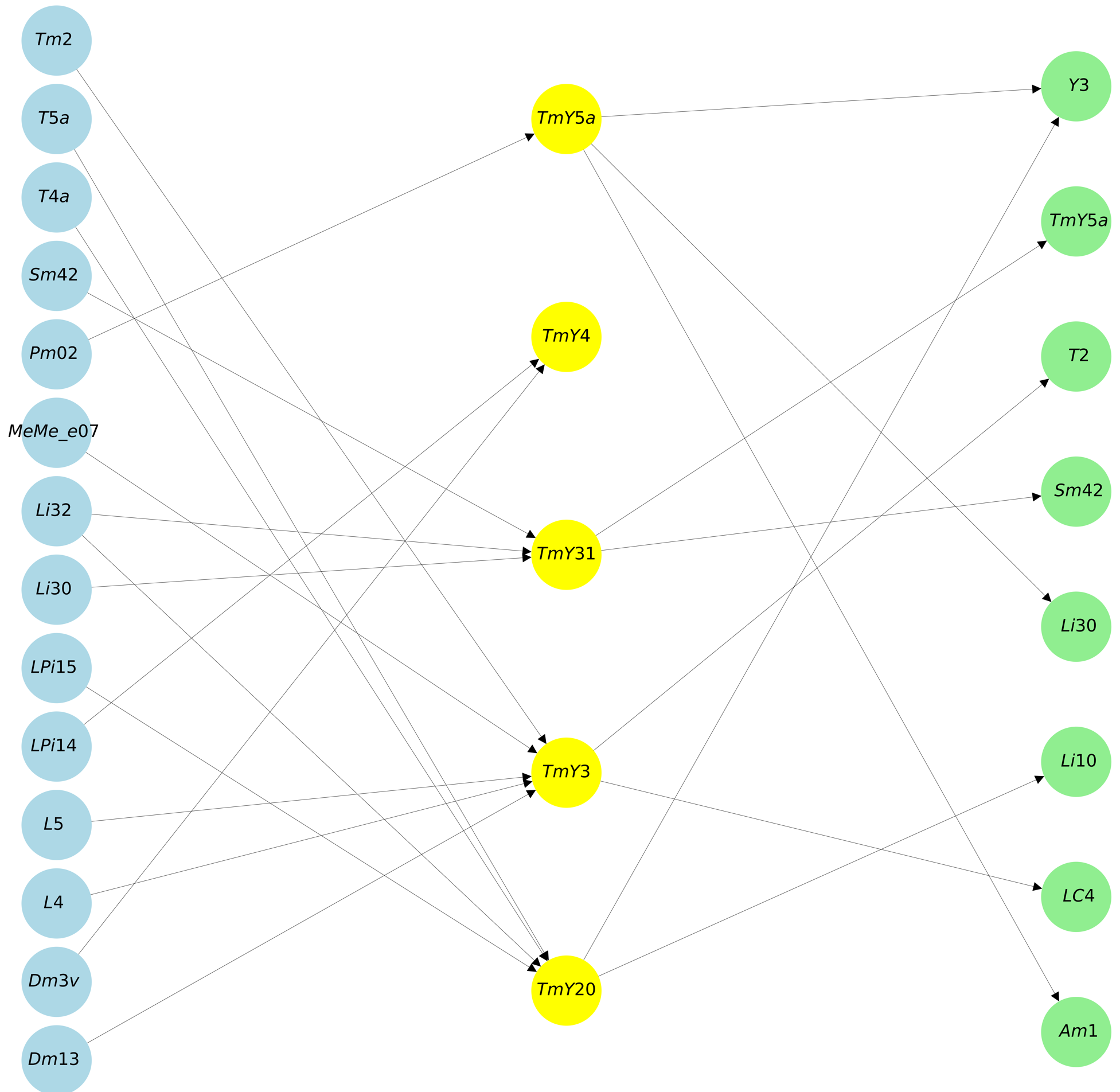

Supplement: Supplementary file 6 — Discriminating logical predicates for all types. Each figure contains types from the same family (middle layer) with shared input attributes (left layer) and output attributes (right layer) that are sufficient for discriminating all types in the middle layer. Families with many types are split into multiple figures for clarity of presentation. [file 41586_2024_7981_MOESM6_ESM.zip › DataS2/pdf/Transmedullary_Y_Predicates_(part_2_of_3).pdf]

Transmedullary Y Predicates (part 3 of 3)

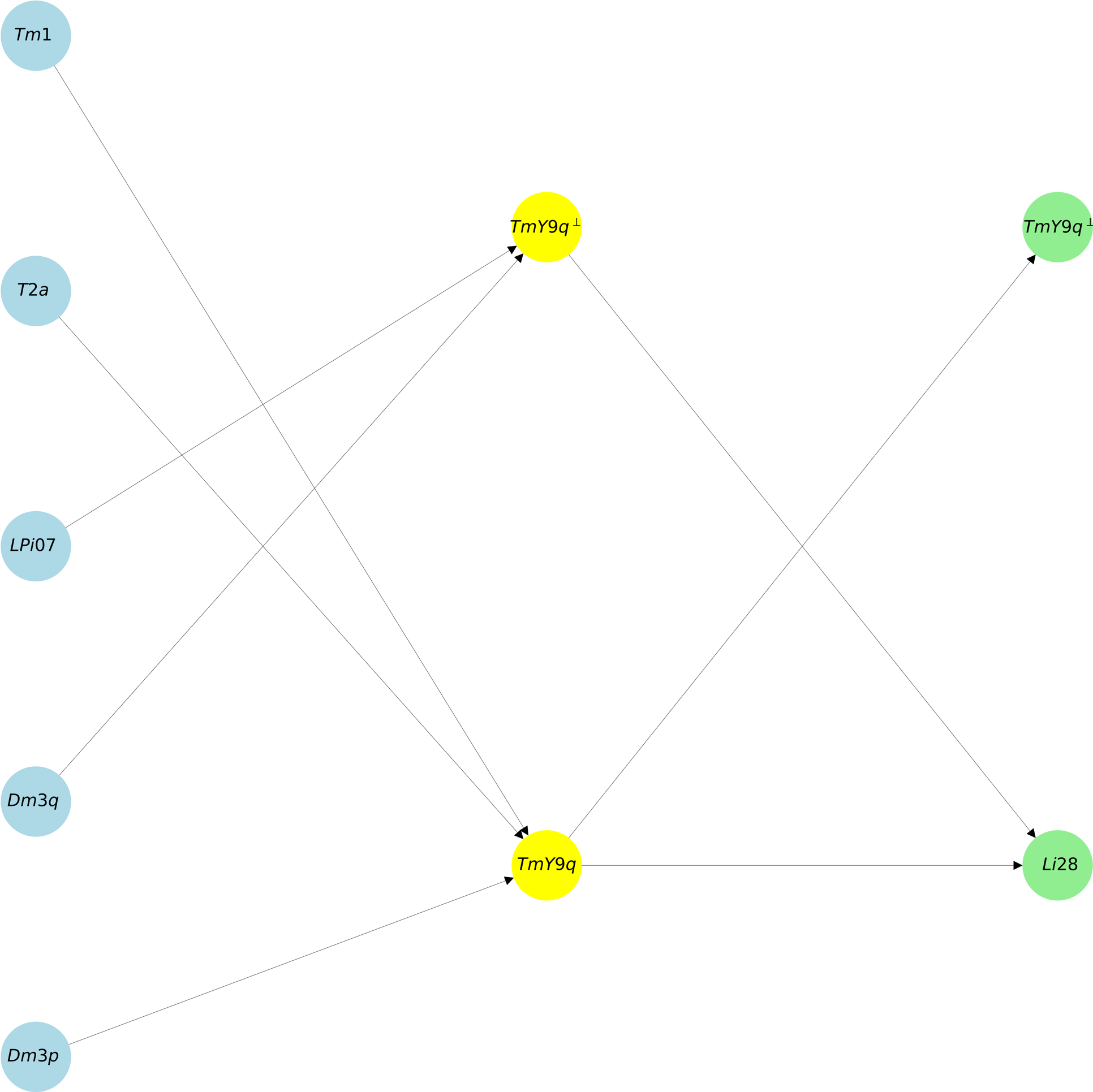

Supplement: Supplementary file 6 — Discriminating logical predicates for all types. Each figure contains types from the same family (middle layer) with shared input attributes (left layer) and output attributes (right layer) that are sufficient for discriminating all types in the middle layer. Families with many types are split into multiple figures for clarity of presentation. [file 41586_2024_7981_MOESM6_ESM.zip › DataS2/pdf/Transmedullary_Y_Predicates_(part_3_of_3).pdf]

## Y Neuron Predicates

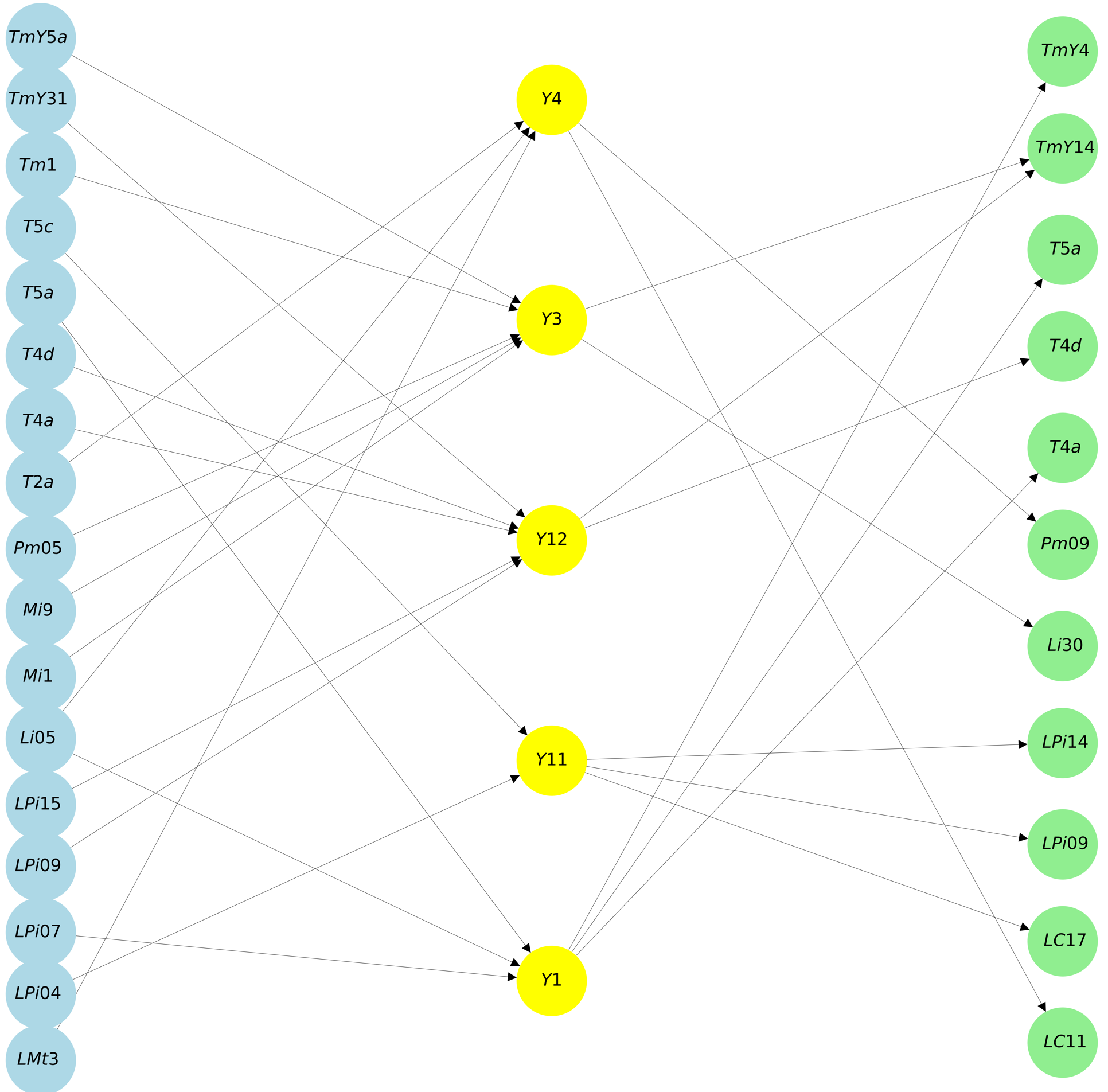

Supplement: Supplementary file 6 — Discriminating logical predicates for all types. Each figure contains types from the same family (middle layer) with shared input attributes (left layer) and output attributes (right layer) that are sufficient for discriminating all types in the middle layer. Families with many types are split into multiple figures for clarity of presentation. [file 41586_2024_7981_MOESM6_ESM.zip › DataS2/pdf/Y_Neuron_Predicates.pdf]

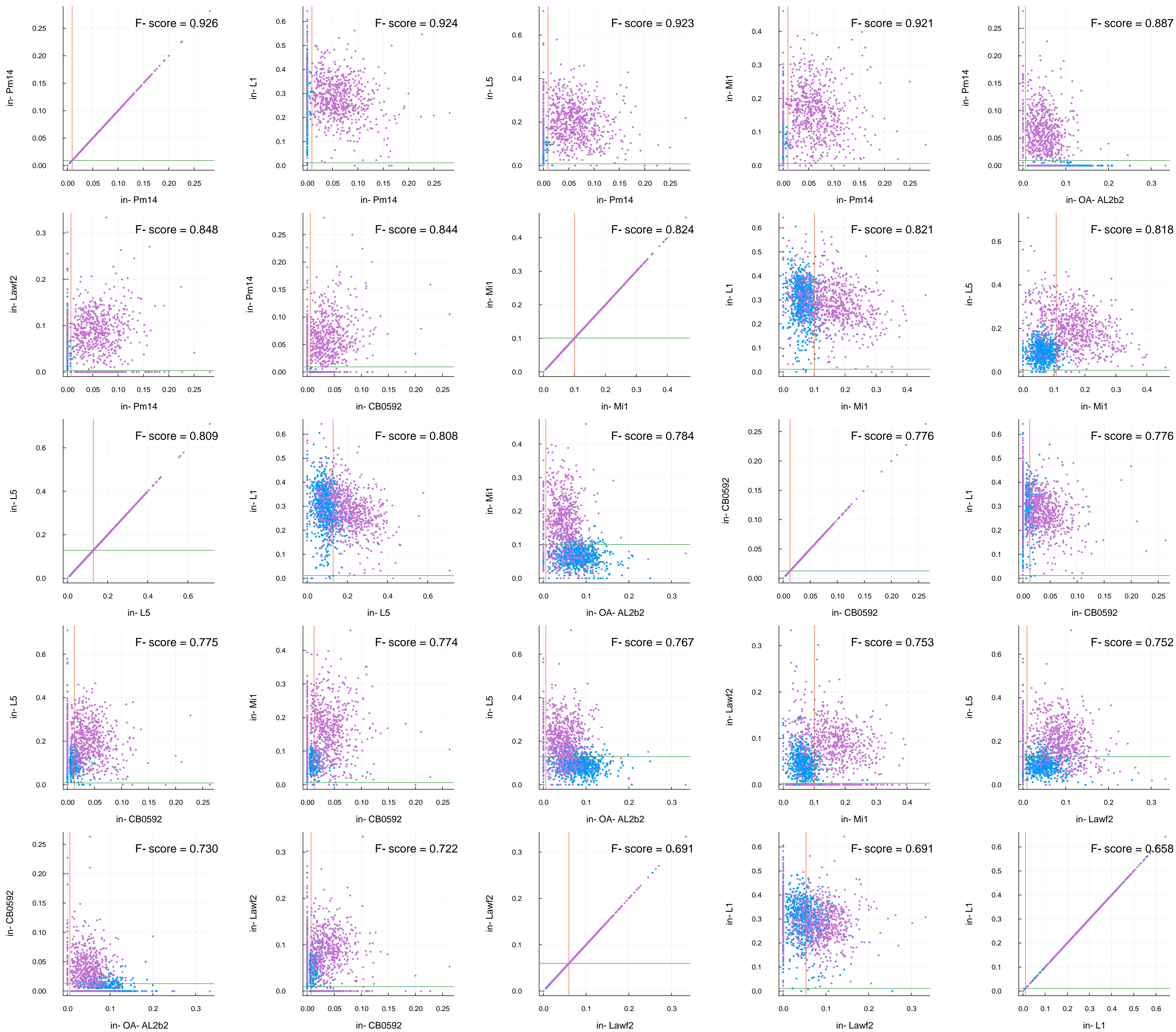

Supplement: Supplementary file 7 — Discriminating 2D projections for neuropil-intrinsic types. For each interneuron type, a pair of features is shown that can be used to discriminate that type from others in the same neuropil. Many although not all discriminations are highly accurate. Both intrinsic and boundary types are included as discriminative features. [file 41586_2024_7981_MOESM7_ESM.zip › DataS3/C2.pdf]

C3

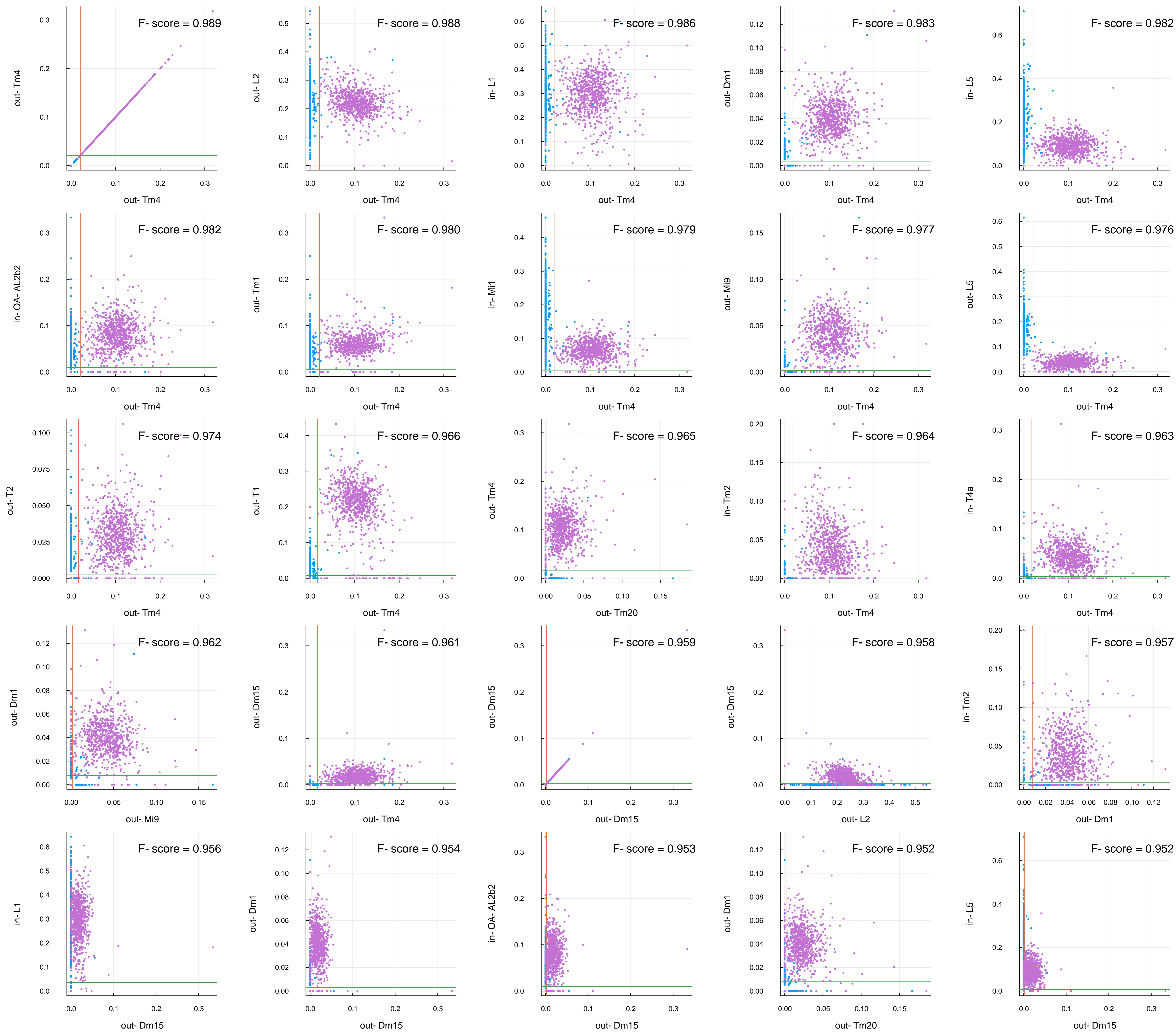

Supplement: Supplementary file 7 — Discriminating 2D projections for neuropil-intrinsic types. For each interneuron type, a pair of features is shown that can be used to discriminate that type from others in the same neuropil. Many although not all discriminations are highly accurate. Both intrinsic and boundary types are included as discriminative features. [file 41586_2024_7981_MOESM7_ESM.zip › DataS3/C3.pdf]

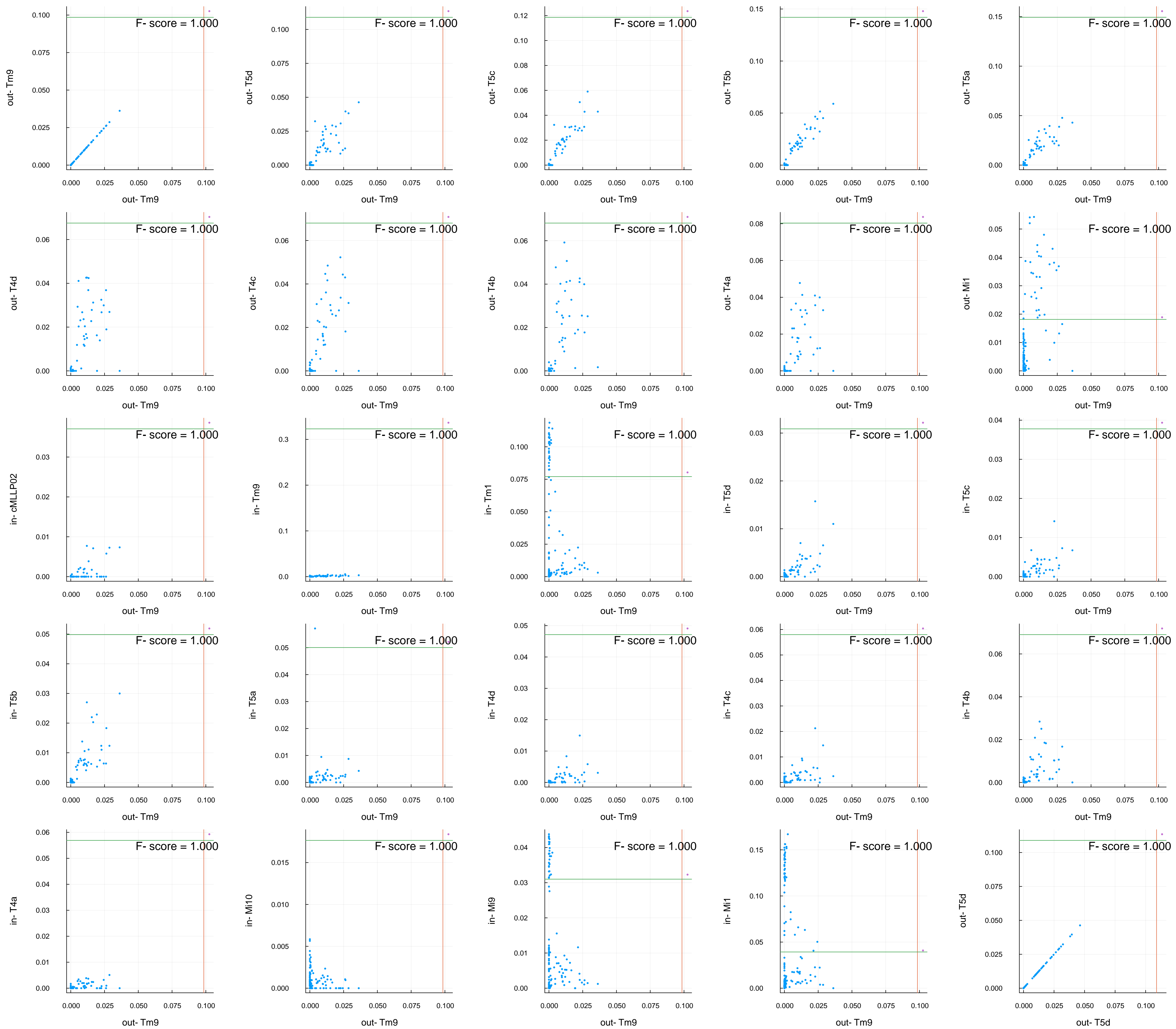

Supplement: Supplementary file 7 — Discriminating 2D projections for neuropil-intrinsic types. For each interneuron type, a pair of features is shown that can be used to discriminate that type from others in the same neuropil. Many although not all discriminations are highly accurate. Both intrinsic and boundary types are included as discriminative features. [file 41586_2024_7981_MOESM7_ESM.zip › DataS3/CT1.pdf]

Dm1

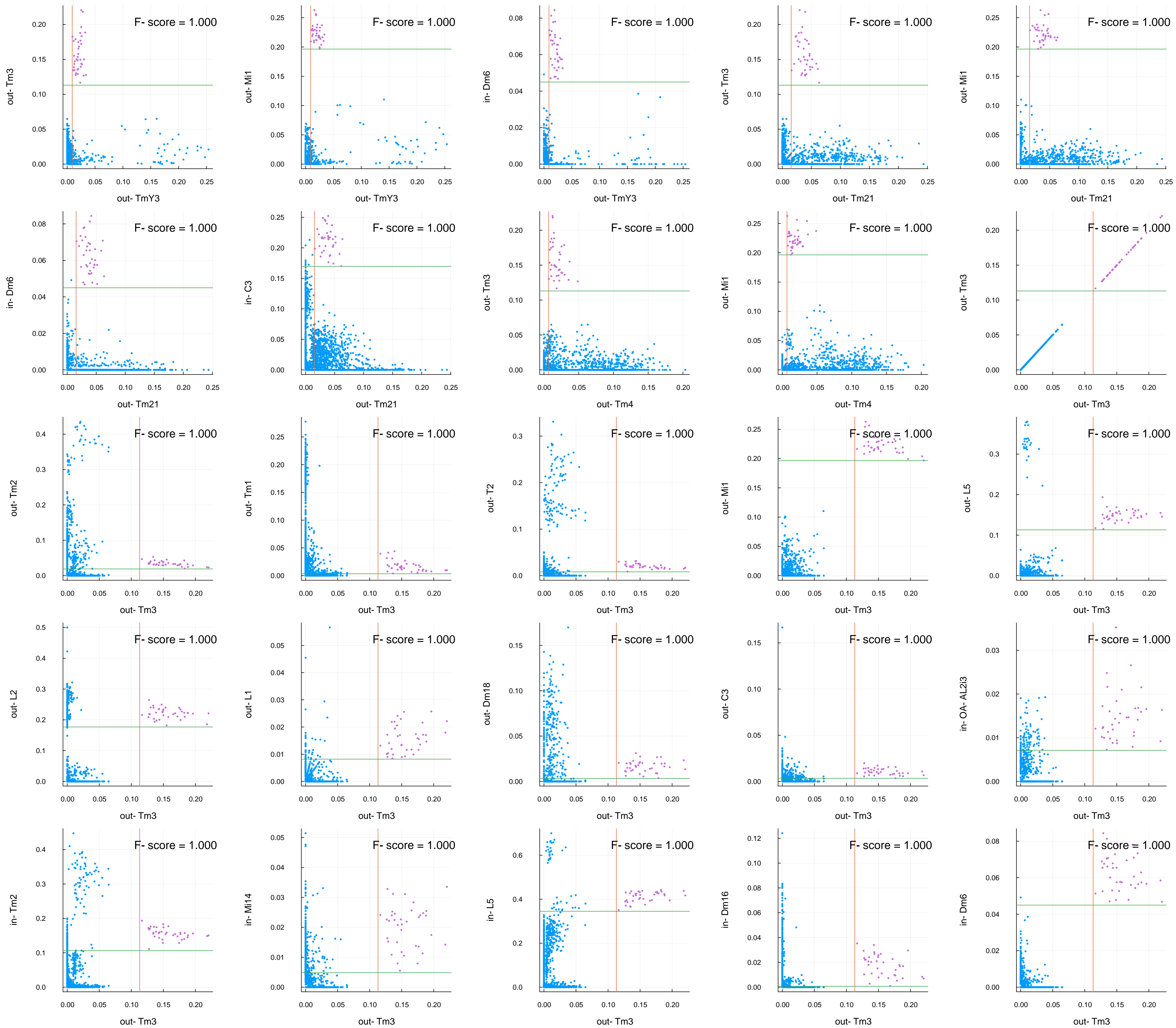

Supplement: Supplementary file 7 — Discriminating 2D projections for neuropil-intrinsic types. For each interneuron type, a pair of features is shown that can be used to discriminate that type from others in the same neuropil. Many although not all discriminations are highly accurate. Both intrinsic and boundary types are included as discriminative features. [file 41586_2024_7981_MOESM7_ESM.zip › DataS3/Dm1.pdf]

# Dm10

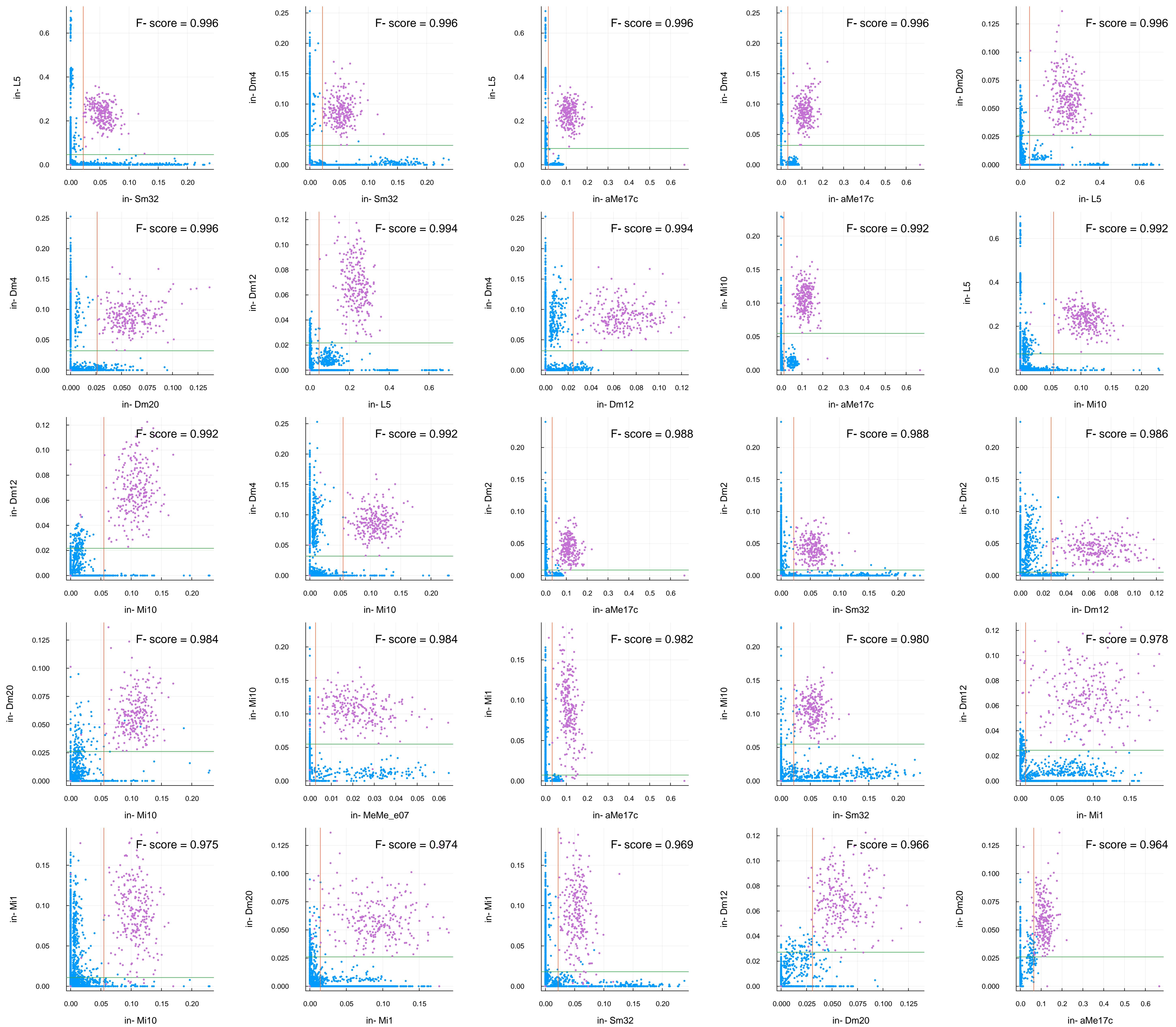

Supplement: Supplementary file 7 — Discriminating 2D projections for neuropil-intrinsic types. For each interneuron type, a pair of features is shown that can be used to discriminate that type from others in the same neuropil. Many although not all discriminations are highly accurate. Both intrinsic and boundary types are included as discriminative features. [file 41586_2024_7981_MOESM7_ESM.zip › DataS3/Dm10.pdf]

Dm11

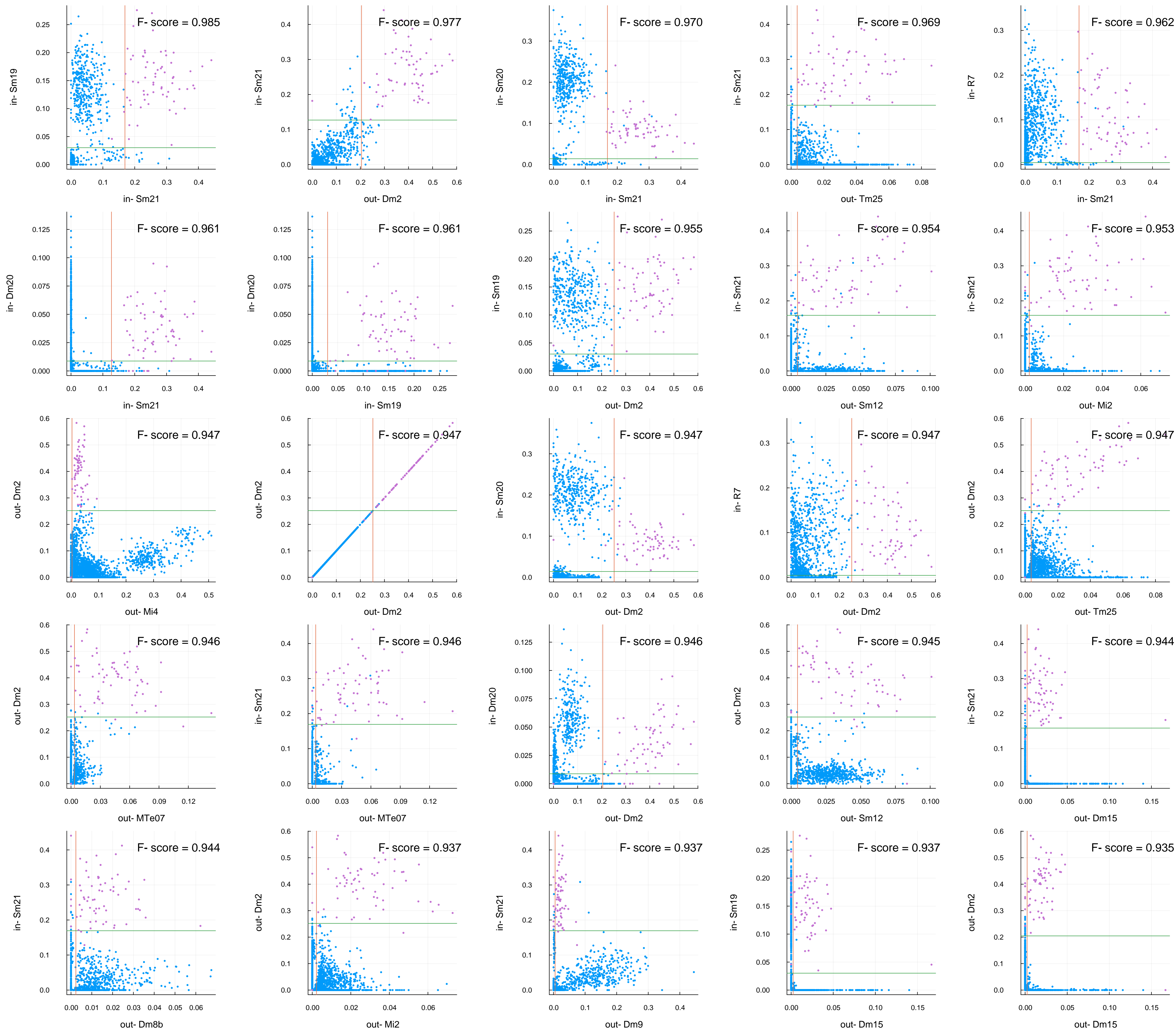

Supplement: Supplementary file 7 — Discriminating 2D projections for neuropil-intrinsic types. For each interneuron type, a pair of features is shown that can be used to discriminate that type from others in the same neuropil. Many although not all discriminations are highly accurate. Both intrinsic and boundary types are included as discriminative features. [file 41586_2024_7981_MOESM7_ESM.zip › DataS3/Dm11.pdf]

Dm12

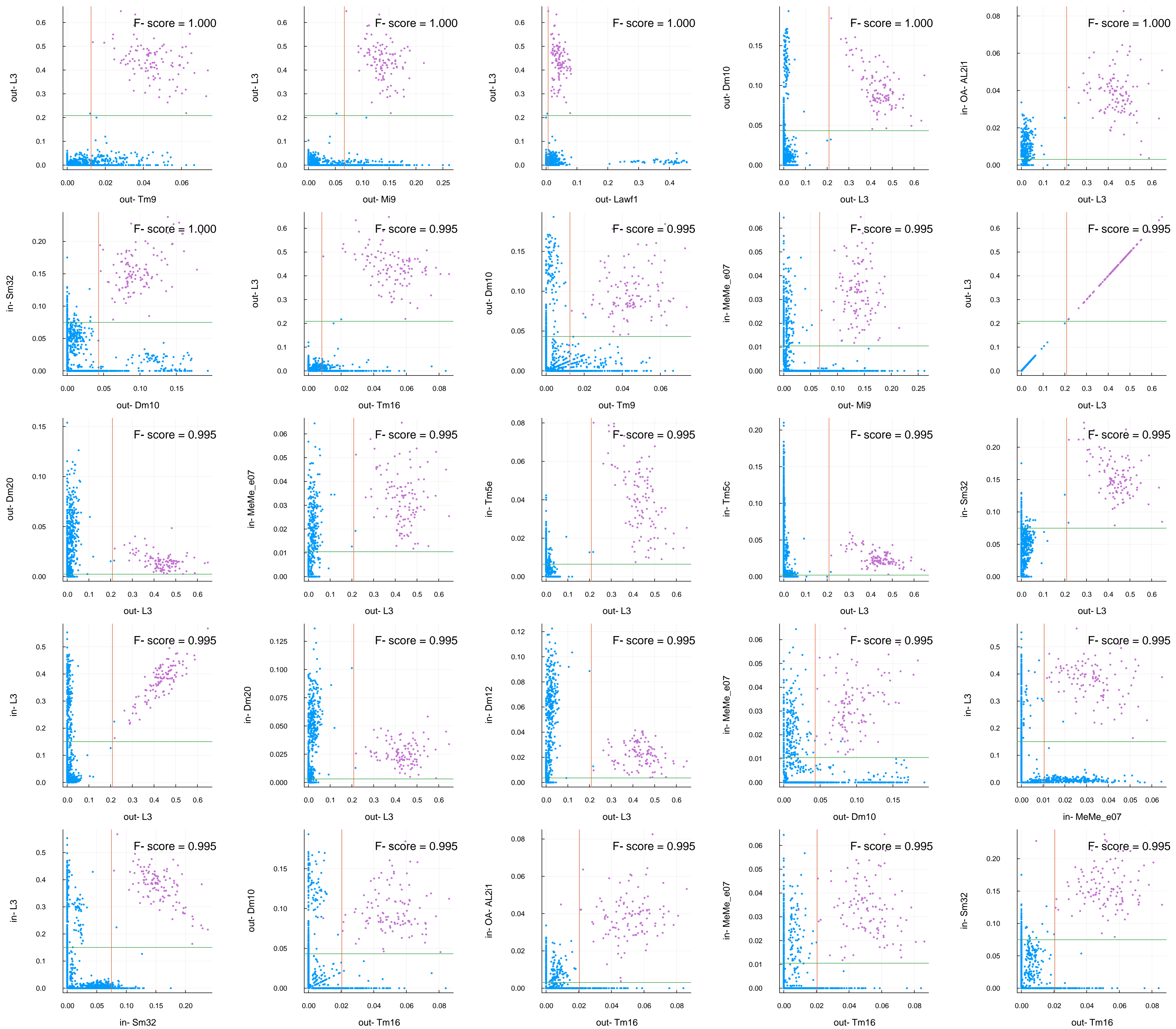

Supplement: Supplementary file 7 — Discriminating 2D projections for neuropil-intrinsic types. For each interneuron type, a pair of features is shown that can be used to discriminate that type from others in the same neuropil. Many although not all discriminations are highly accurate. Both intrinsic and boundary types are included as discriminative features. [file 41586_2024_7981_MOESM7_ESM.zip › DataS3/Dm12.pdf]

Dm13

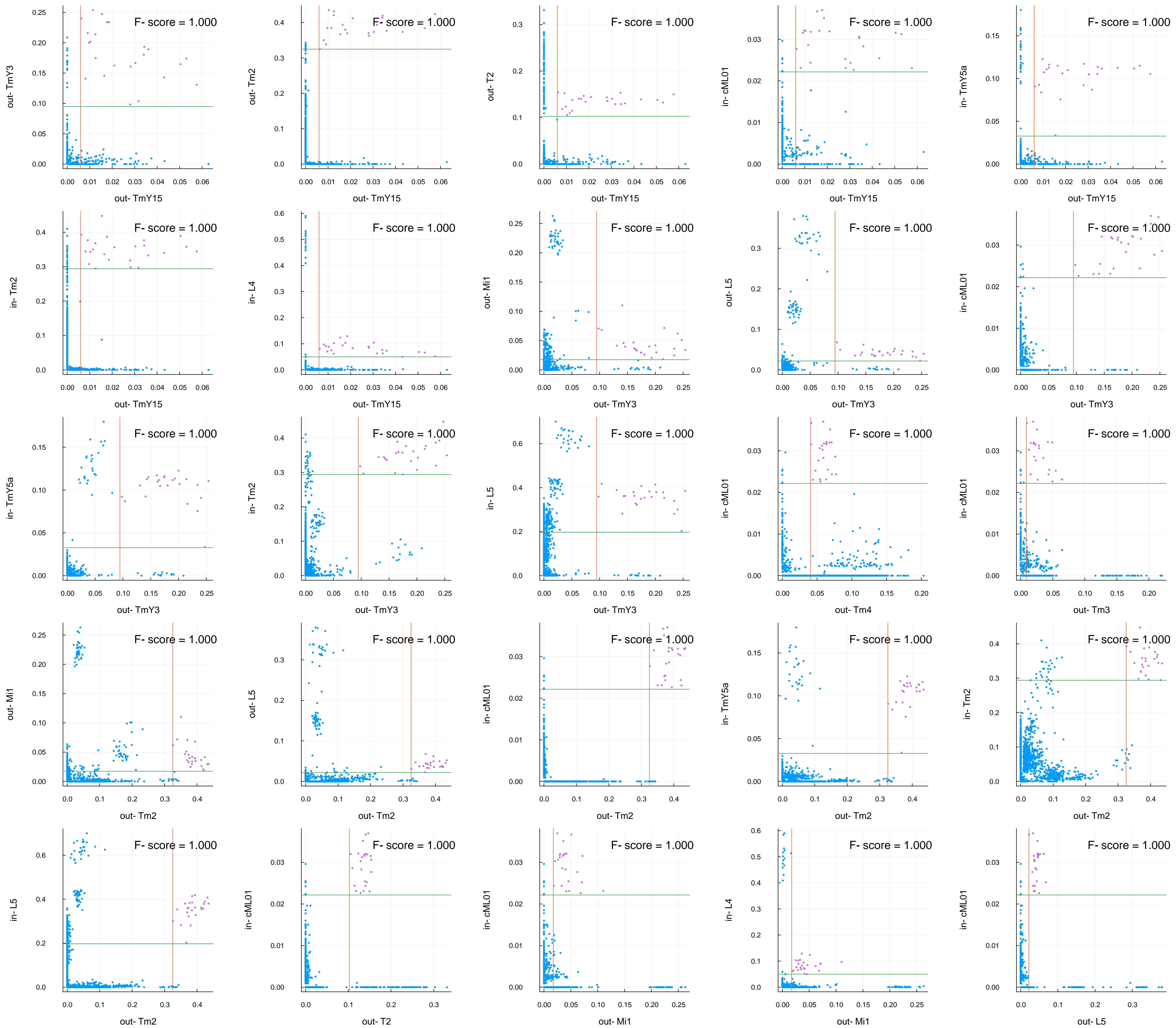

Supplement: Supplementary file 7 — Discriminating 2D projections for neuropil-intrinsic types. For each interneuron type, a pair of features is shown that can be used to discriminate that type from others in the same neuropil. Many although not all discriminations are highly accurate. Both intrinsic and boundary types are included as discriminative features. [file 41586_2024_7981_MOESM7_ESM.zip › DataS3/Dm13.pdf]

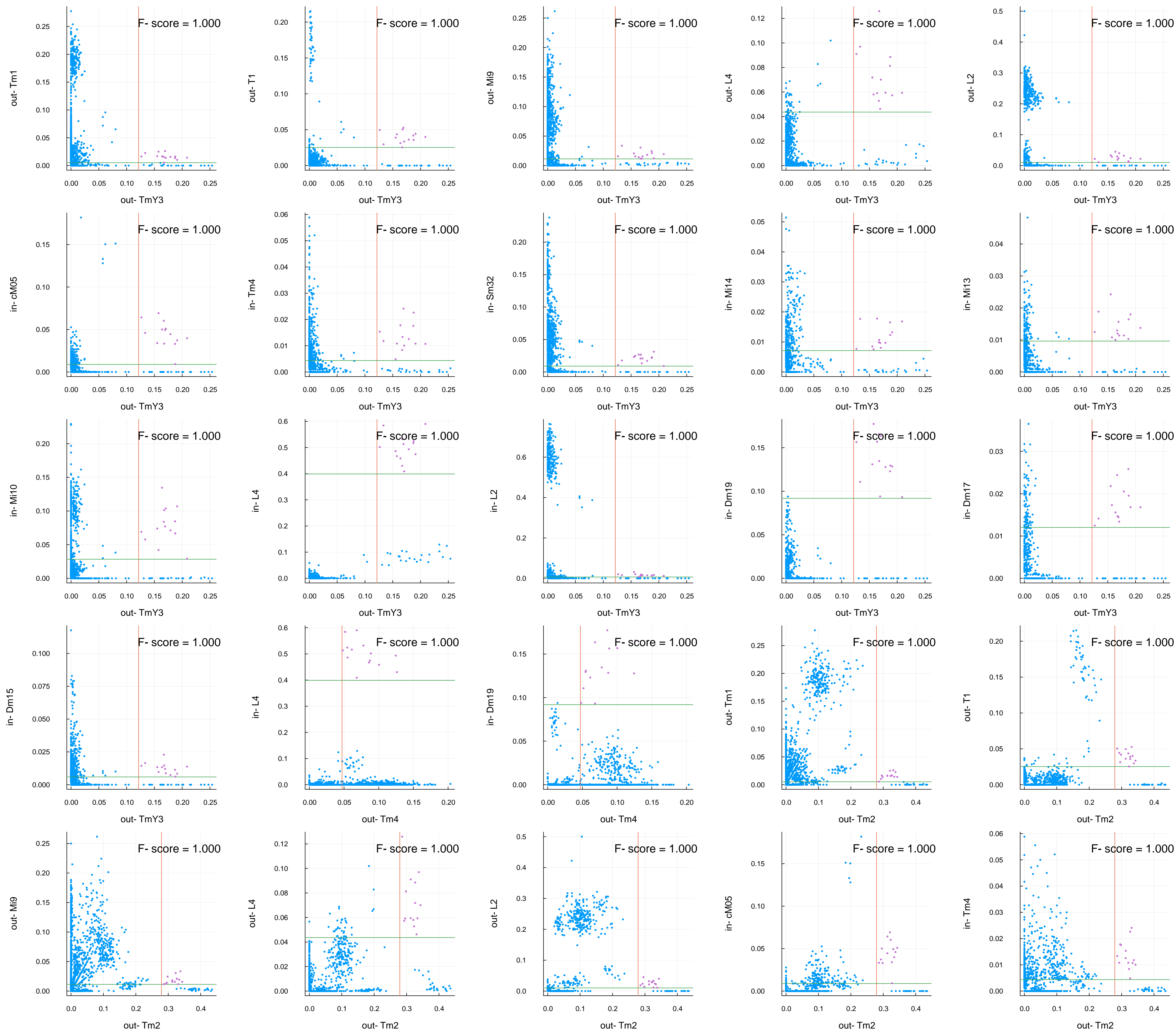

Supplement: Supplementary file 7 — Discriminating 2D projections for neuropil-intrinsic types. For each interneuron type, a pair of features is shown that can be used to discriminate that type from others in the same neuropil. Many although not all discriminations are highly accurate. Both intrinsic and boundary types are included as discriminative features. [file 41586_2024_7981_MOESM7_ESM.zip › DataS3/Dm14.pdf]

Dm15

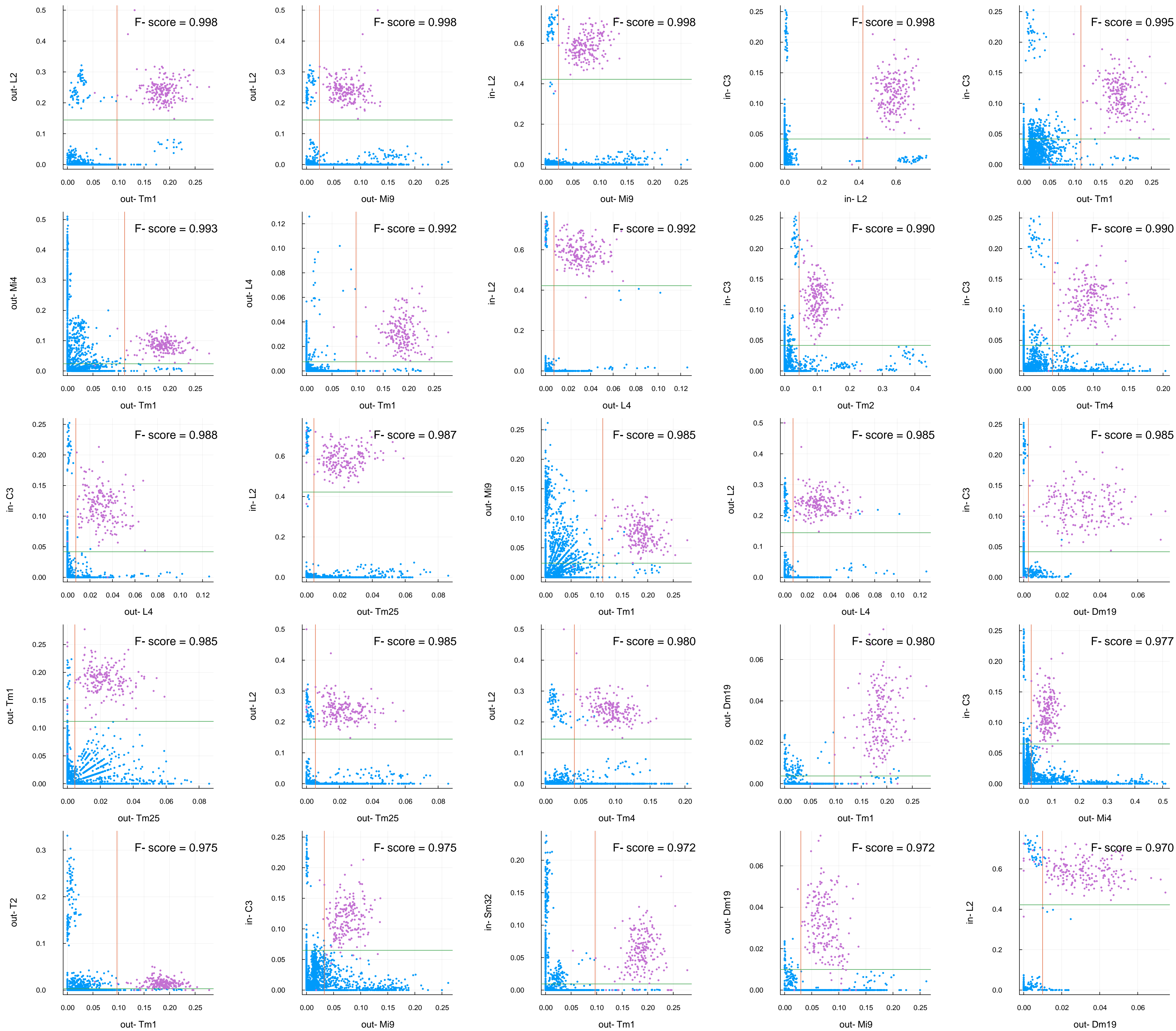

Supplement: Supplementary file 7 — Discriminating 2D projections for neuropil-intrinsic types. For each interneuron type, a pair of features is shown that can be used to discriminate that type from others in the same neuropil. Many although not all discriminations are highly accurate. Both intrinsic and boundary types are included as discriminative features. [file 41586_2024_7981_MOESM7_ESM.zip › DataS3/Dm15.pdf]

Dm16

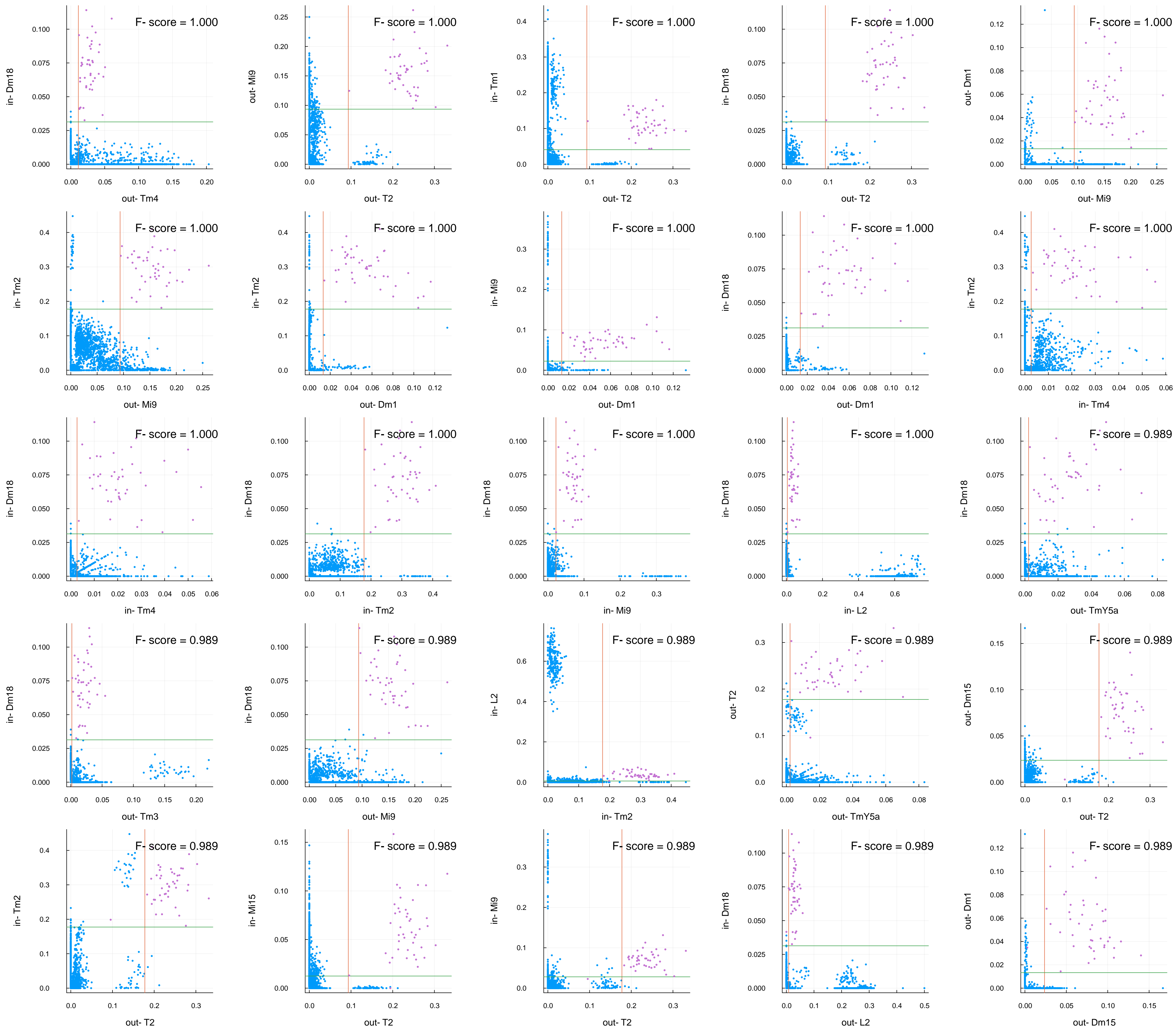

Supplement: Supplementary file 7 — Discriminating 2D projections for neuropil-intrinsic types. For each interneuron type, a pair of features is shown that can be used to discriminate that type from others in the same neuropil. Many although not all discriminations are highly accurate. Both intrinsic and boundary types are included as discriminative features. [file 41586_2024_7981_MOESM7_ESM.zip › DataS3/Dm16.pdf]

Dm17

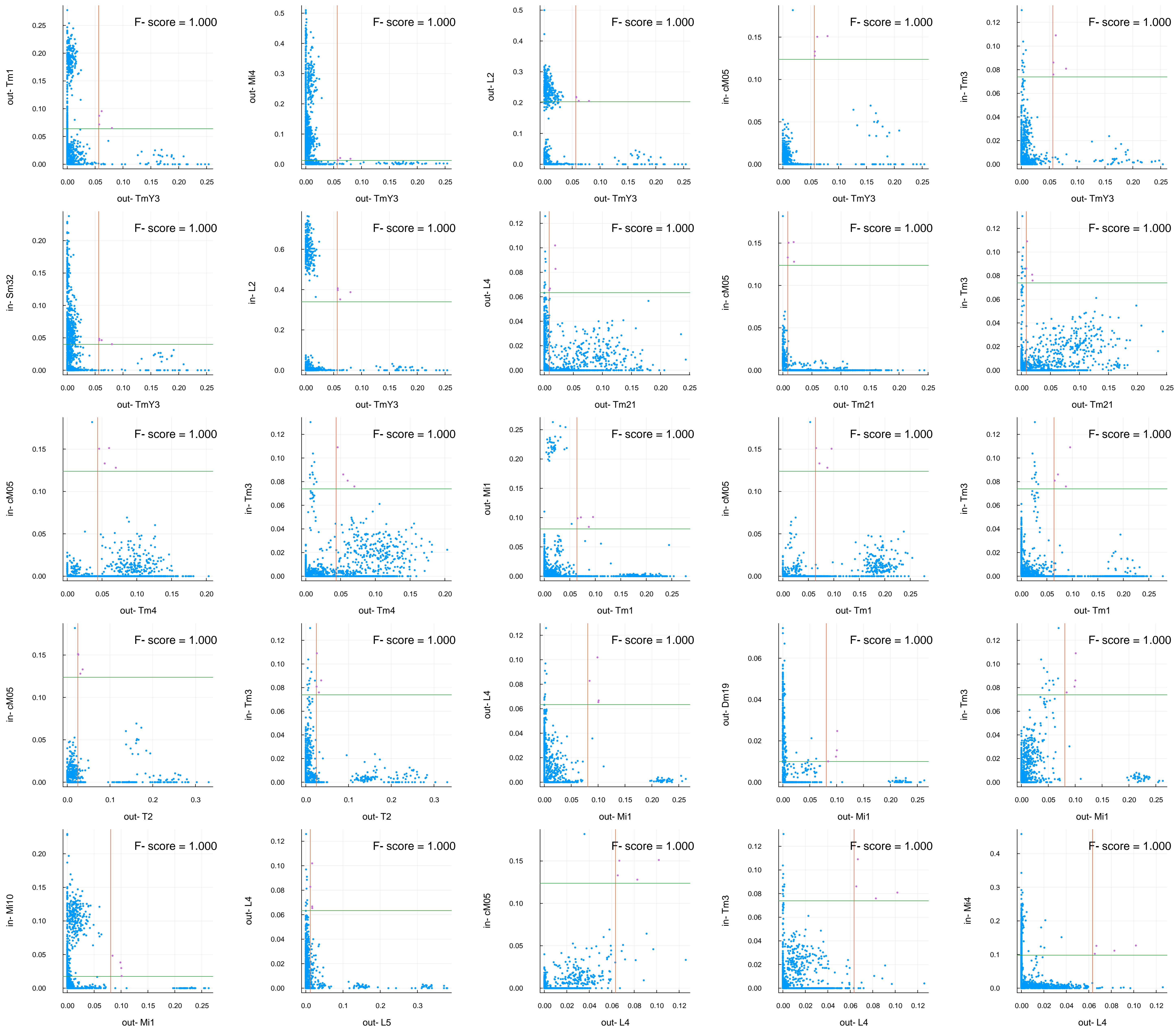

Supplement: Supplementary file 7 — Discriminating 2D projections for neuropil-intrinsic types. For each interneuron type, a pair of features is shown that can be used to discriminate that type from others in the same neuropil. Many although not all discriminations are highly accurate. Both intrinsic and boundary types are included as discriminative features. [file 41586_2024_7981_MOESM7_ESM.zip › DataS3/Dm17.pdf]

Dm18

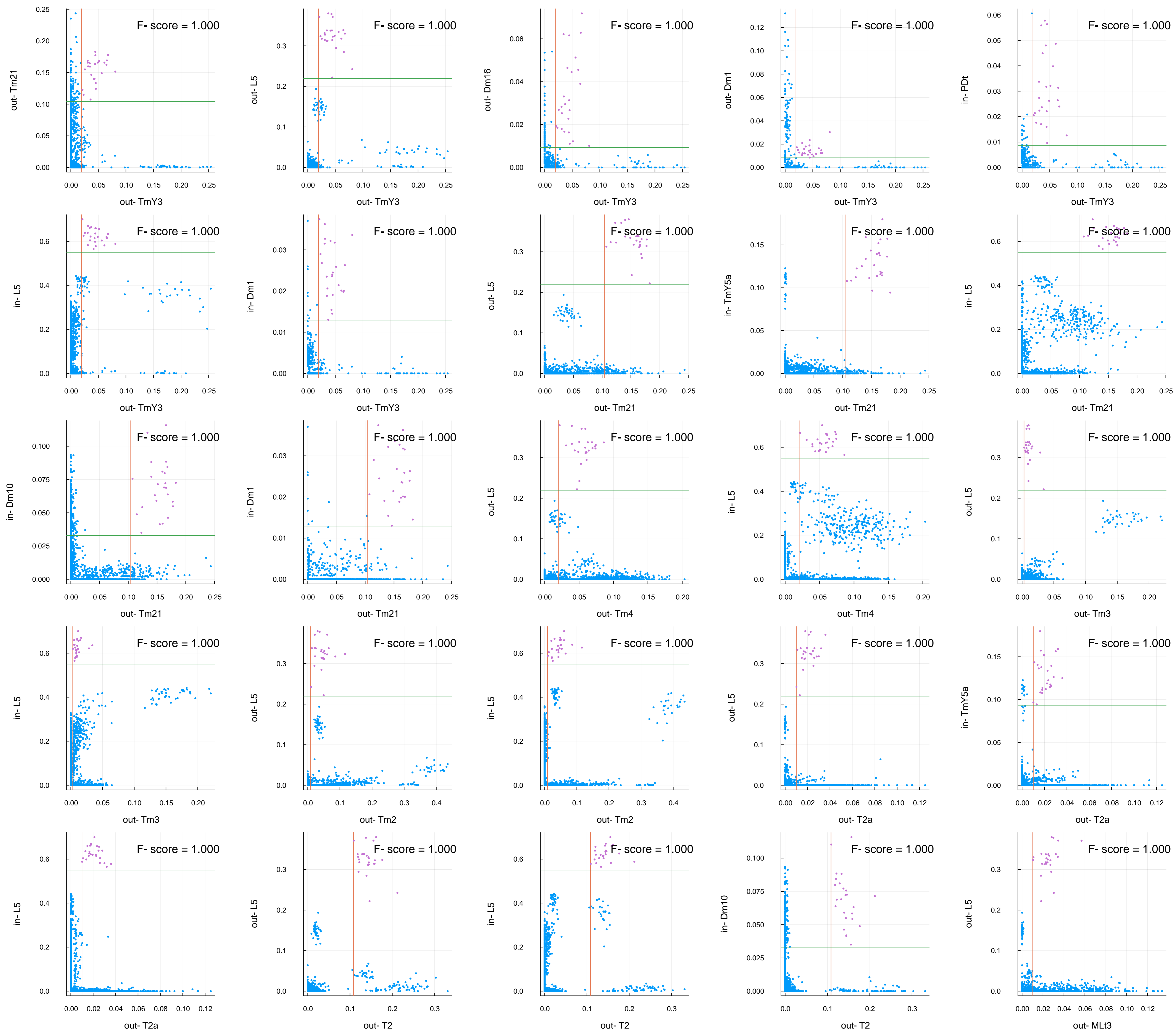

Supplement: Supplementary file 7 — Discriminating 2D projections for neuropil-intrinsic types. For each interneuron type, a pair of features is shown that can be used to discriminate that type from others in the same neuropil. Many although not all discriminations are highly accurate. Both intrinsic and boundary types are included as discriminative features. [file 41586_2024_7981_MOESM7_ESM.zip › DataS3/Dm18.pdf]

Dm19

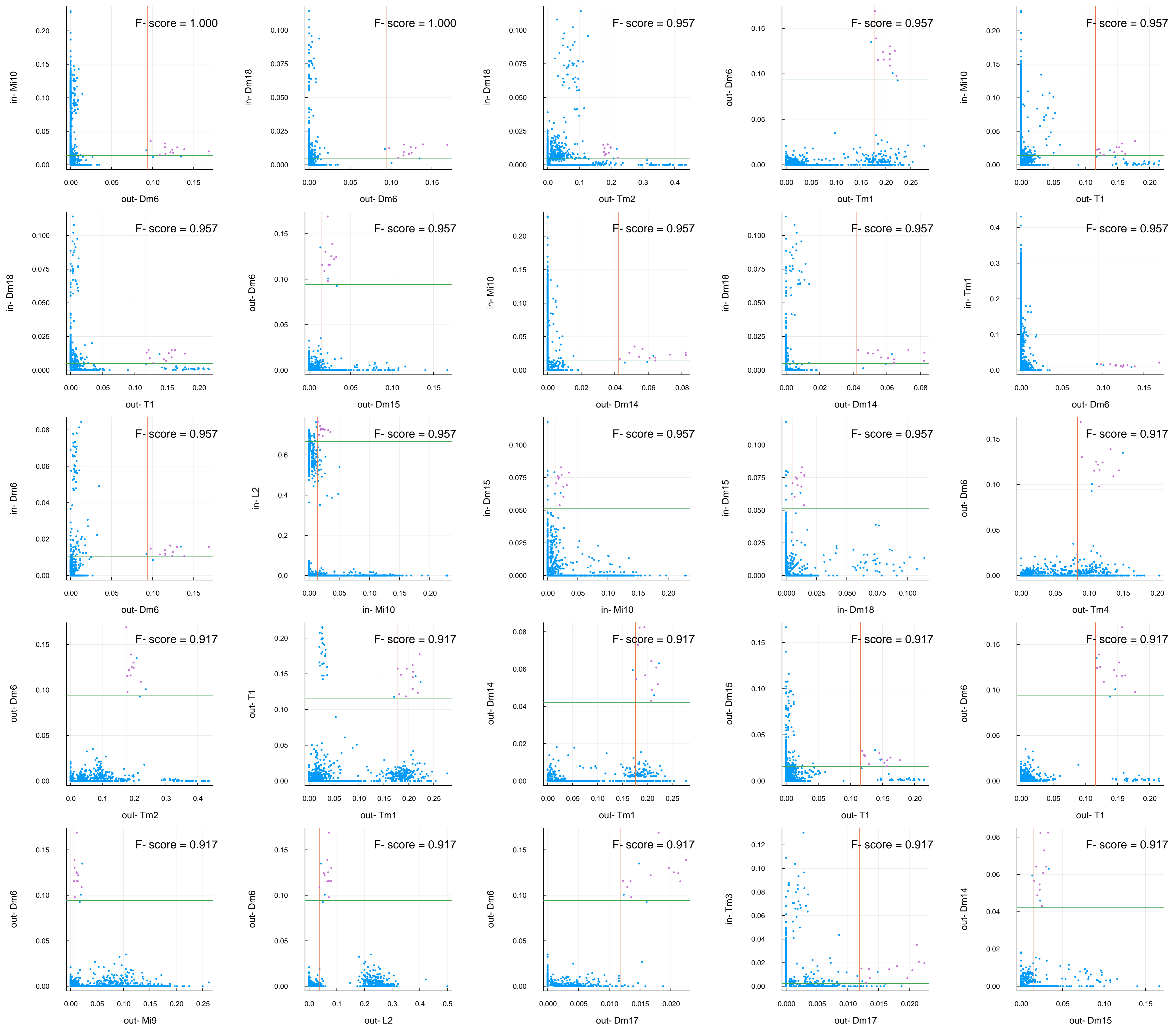

Supplement: Supplementary file 7 — Discriminating 2D projections for neuropil-intrinsic types. For each interneuron type, a pair of features is shown that can be used to discriminate that type from others in the same neuropil. Many although not all discriminations are highly accurate. Both intrinsic and boundary types are included as discriminative features. [file 41586_2024_7981_MOESM7_ESM.zip › DataS3/Dm19.pdf]

Dm2

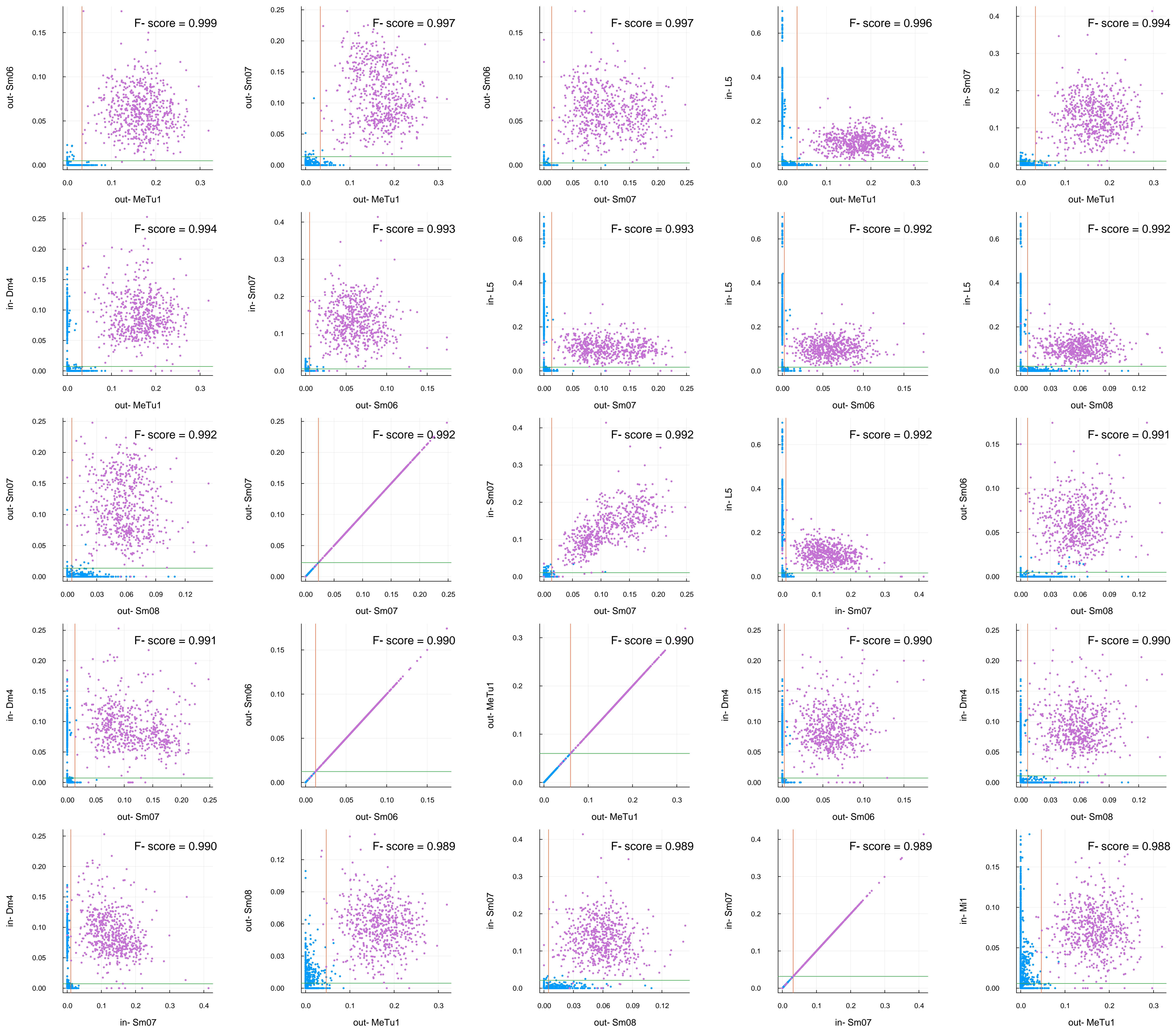

Supplement: Supplementary file 7 — Discriminating 2D projections for neuropil-intrinsic types. For each interneuron type, a pair of features is shown that can be used to discriminate that type from others in the same neuropil. Many although not all discriminations are highly accurate. Both intrinsic and boundary types are included as discriminative features. [file 41586_2024_7981_MOESM7_ESM.zip › DataS3/Dm2.pdf]

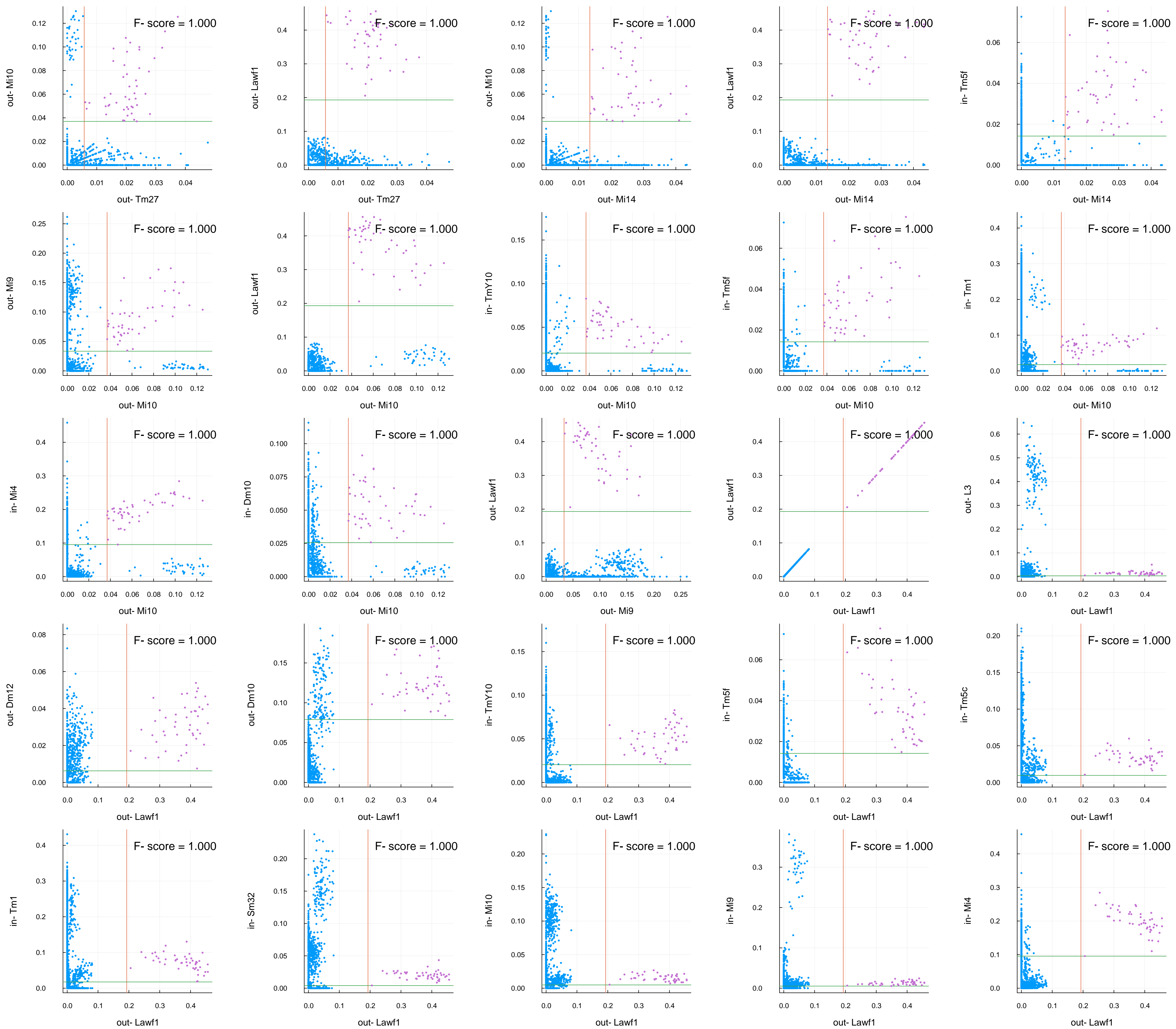

Supplement: Supplementary file 7 — Discriminating 2D projections for neuropil-intrinsic types. For each interneuron type, a pair of features is shown that can be used to discriminate that type from others in the same neuropil. Many although not all discriminations are highly accurate. Both intrinsic and boundary types are included as discriminative features. [file 41586_2024_7981_MOESM7_ESM.zip › DataS3/Dm20.pdf]

Dm3p

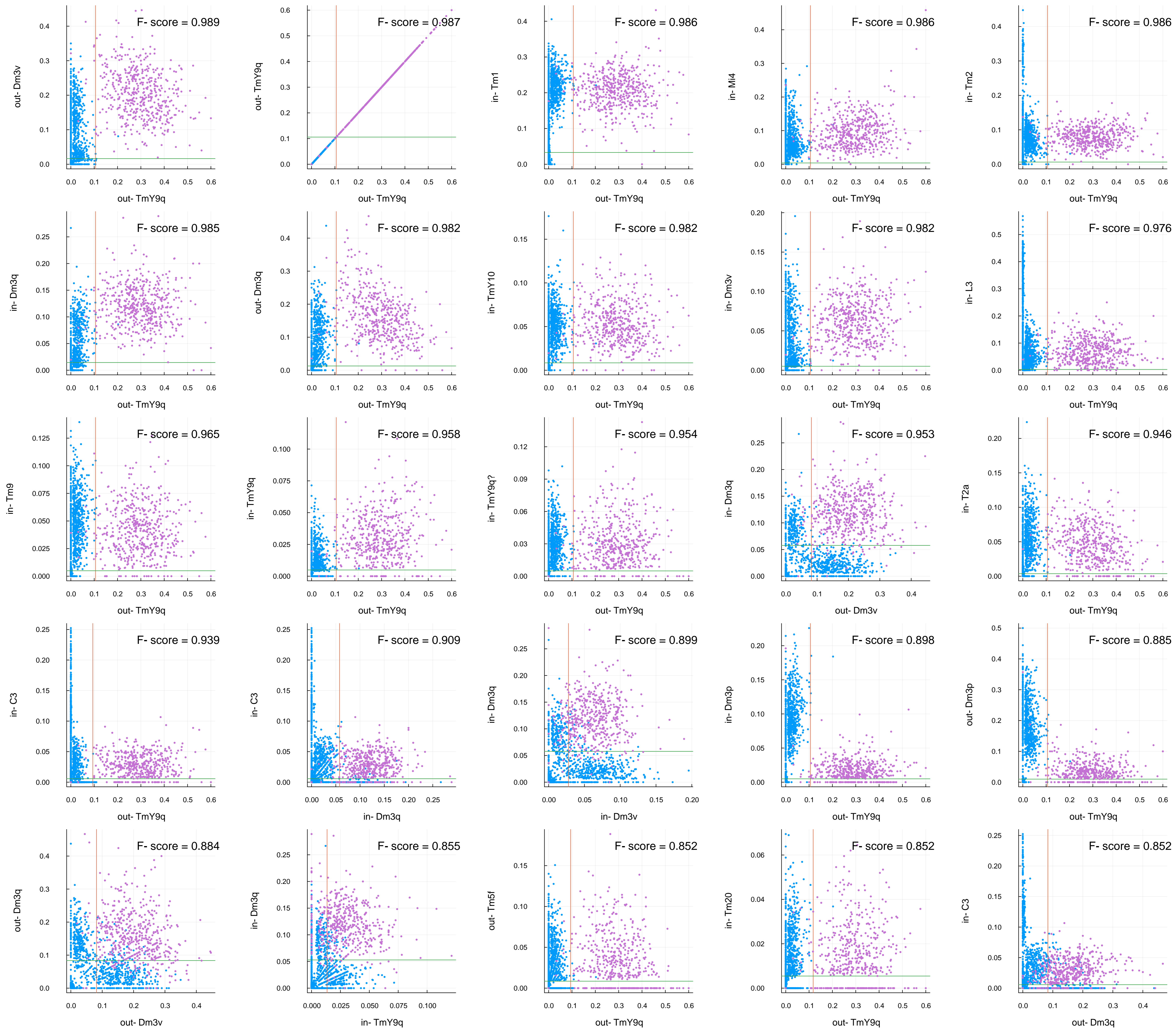

Supplement: Supplementary file 7 — Discriminating 2D projections for neuropil-intrinsic types. For each interneuron type, a pair of features is shown that can be used to discriminate that type from others in the same neuropil. Many although not all discriminations are highly accurate. Both intrinsic and boundary types are included as discriminative features. [file 41586_2024_7981_MOESM7_ESM.zip › DataS3/Dm3p.pdf]

Dm3q

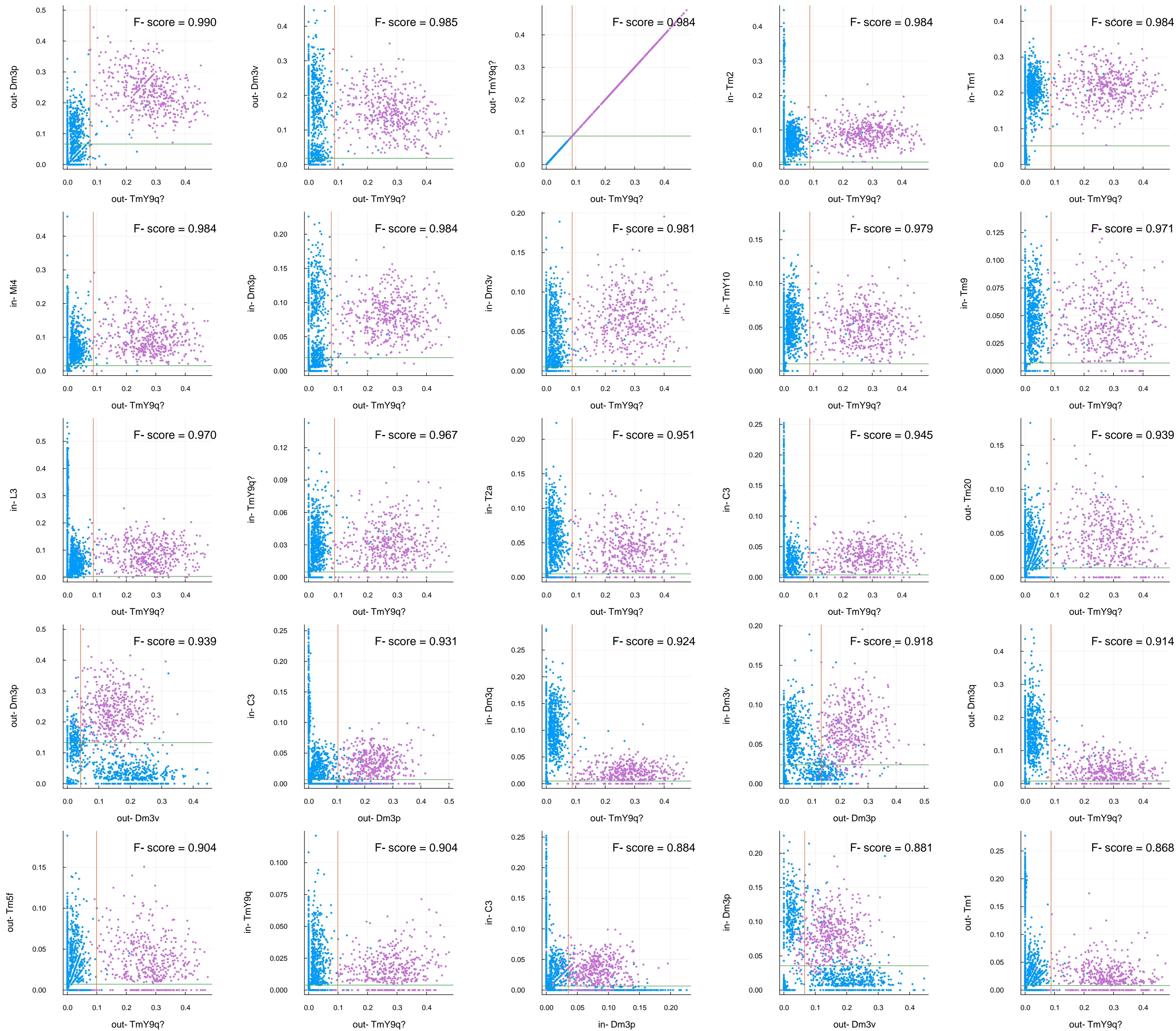

Supplement: Supplementary file 7 — Discriminating 2D projections for neuropil-intrinsic types. For each interneuron type, a pair of features is shown that can be used to discriminate that type from others in the same neuropil. Many although not all discriminations are highly accurate. Both intrinsic and boundary types are included as discriminative features. [file 41586_2024_7981_MOESM7_ESM.zip › DataS3/Dm3q.pdf]

Dm3v

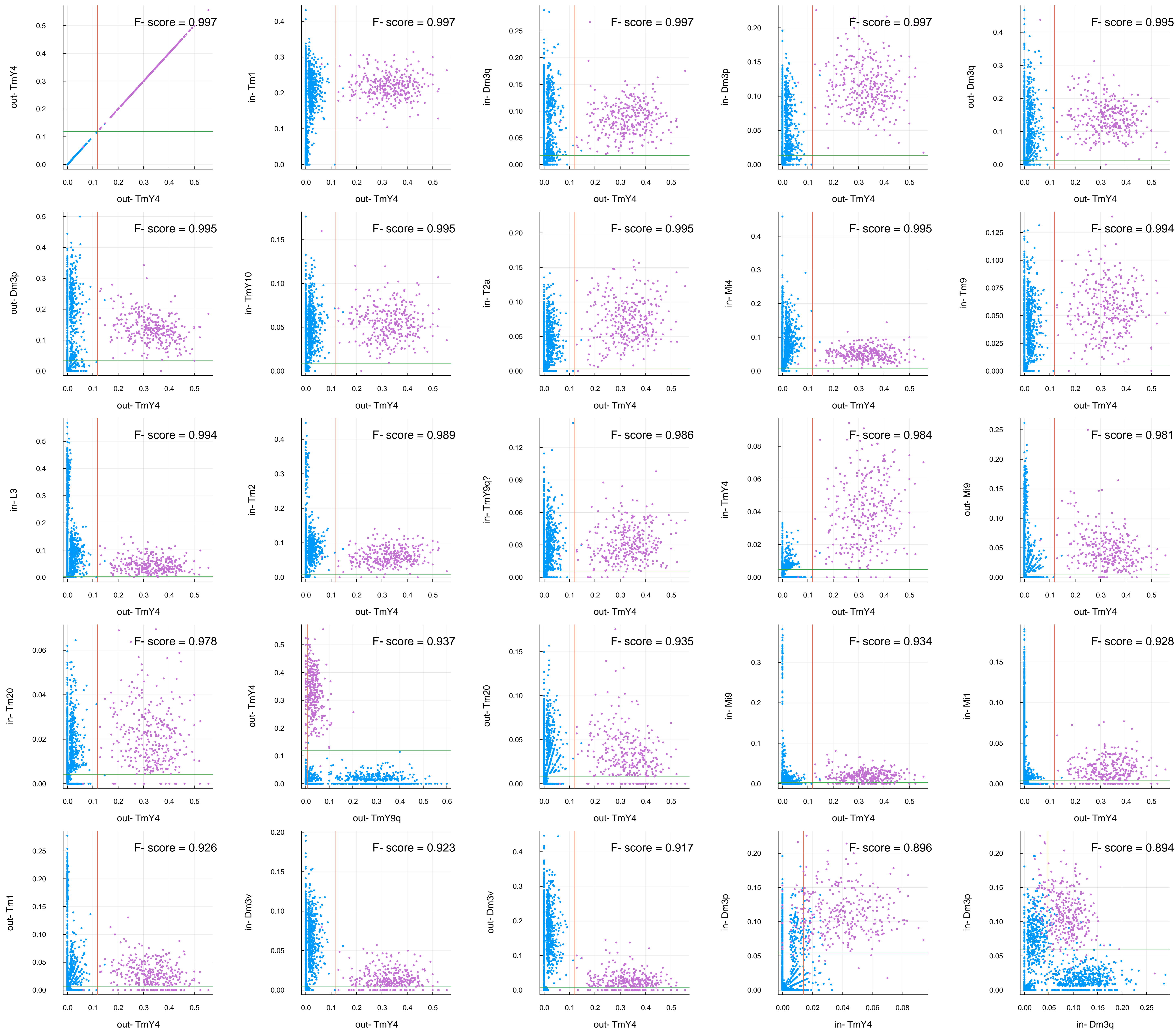

Supplement: Supplementary file 7 — Discriminating 2D projections for neuropil-intrinsic types. For each interneuron type, a pair of features is shown that can be used to discriminate that type from others in the same neuropil. Many although not all discriminations are highly accurate. Both intrinsic and boundary types are included as discriminative features. [file 41586_2024_7981_MOESM7_ESM.zip › DataS3/Dm3v.pdf]

Dm4

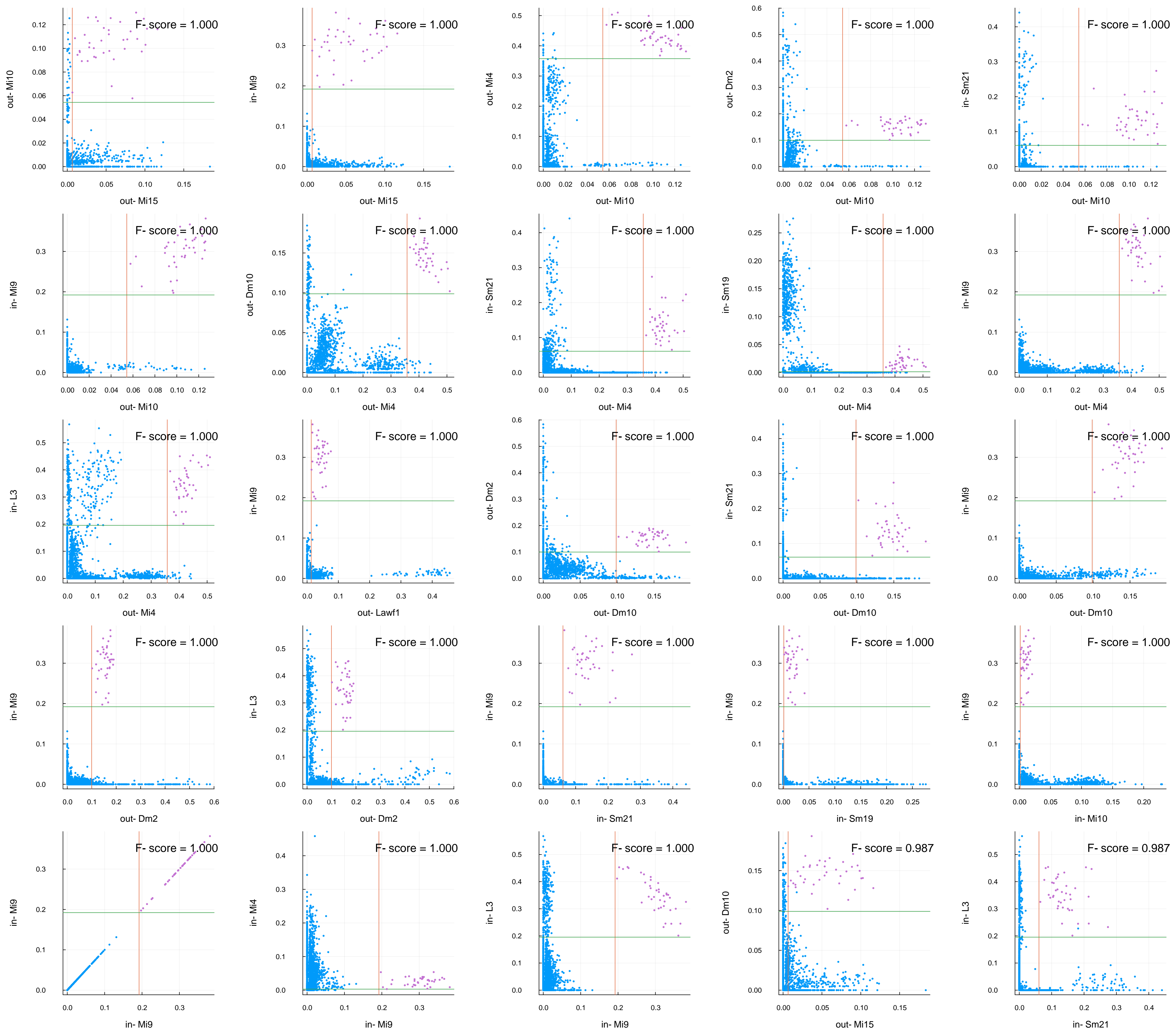

Supplement: Supplementary file 7 — Discriminating 2D projections for neuropil-intrinsic types. For each interneuron type, a pair of features is shown that can be used to discriminate that type from others in the same neuropil. Many although not all discriminations are highly accurate. Both intrinsic and boundary types are included as discriminative features. [file 41586_2024_7981_MOESM7_ESM.zip › DataS3/Dm4.pdf]

Dm6

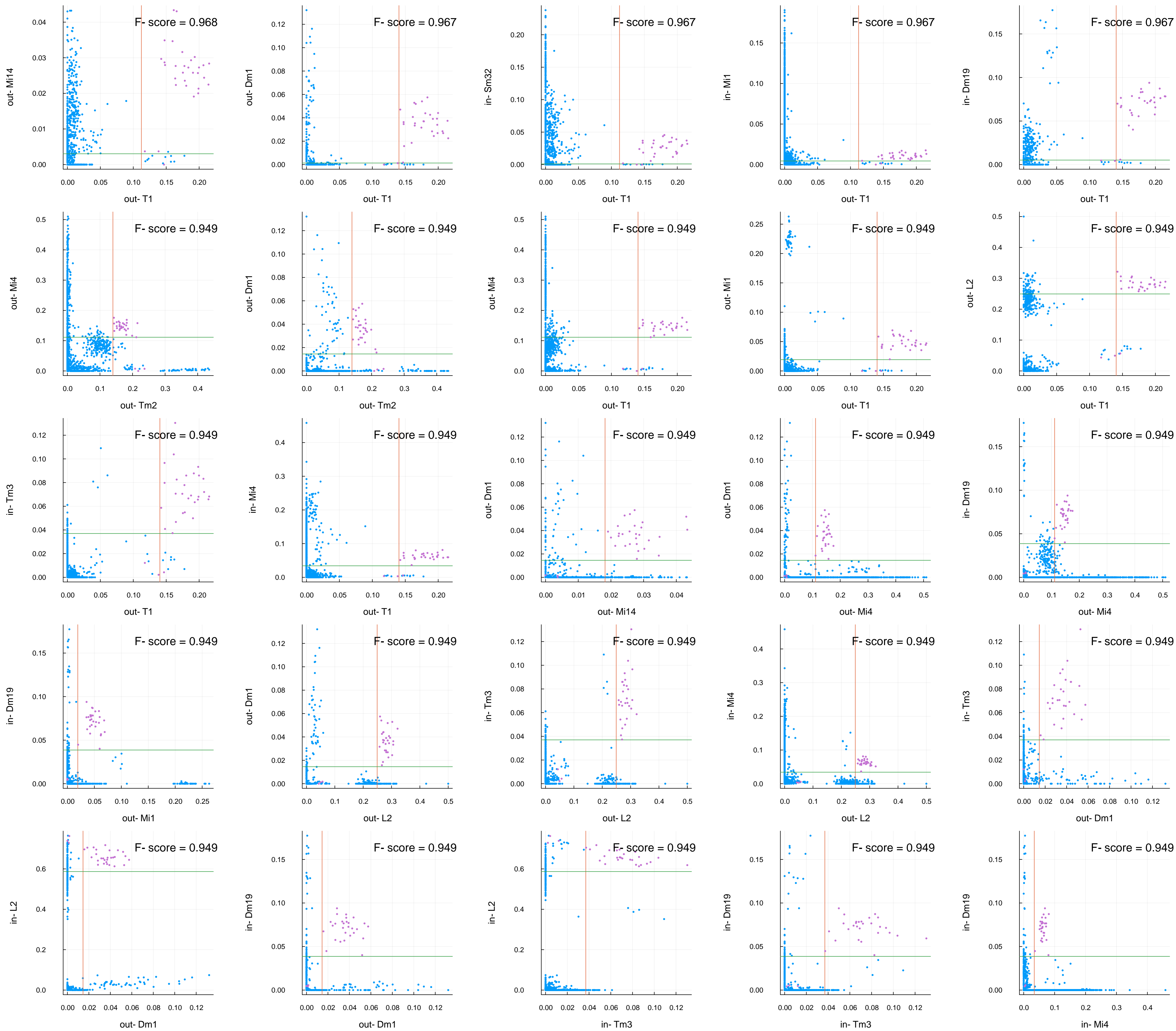

Supplement: Supplementary file 7 — Discriminating 2D projections for neuropil-intrinsic types. For each interneuron type, a pair of features is shown that can be used to discriminate that type from others in the same neuropil. Many although not all discriminations are highly accurate. Both intrinsic and boundary types are included as discriminative features. [file 41586_2024_7981_MOESM7_ESM.zip › DataS3/Dm6.pdf]

Dm8a

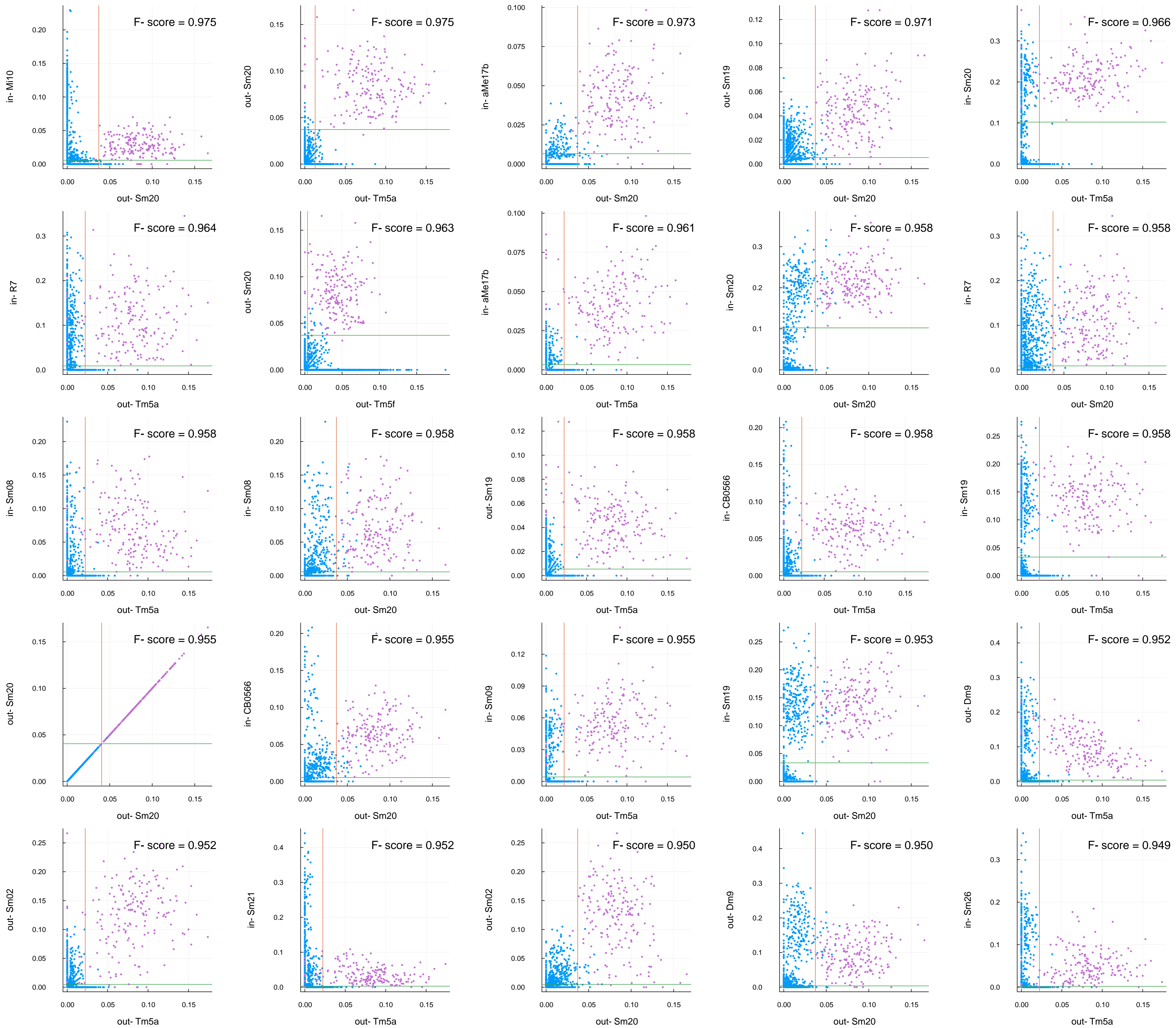

Supplement: Supplementary file 7 — Discriminating 2D projections for neuropil-intrinsic types. For each interneuron type, a pair of features is shown that can be used to discriminate that type from others in the same neuropil. Many although not all discriminations are highly accurate. Both intrinsic and boundary types are included as discriminative features. [file 41586_2024_7981_MOESM7_ESM.zip › DataS3/Dm8a.pdf]

Dm8b

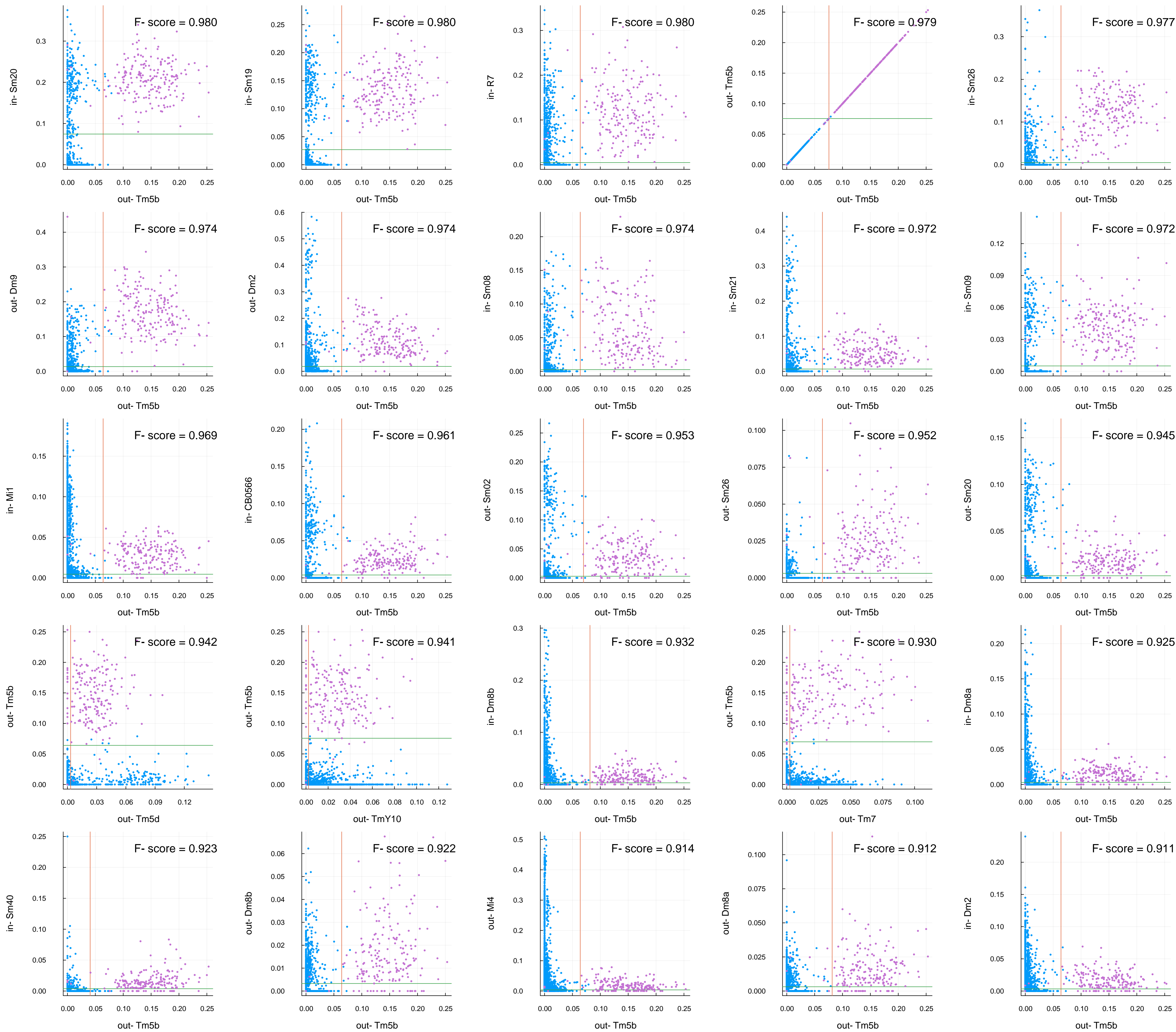

Supplement: Supplementary file 7 — Discriminating 2D projections for neuropil-intrinsic types. For each interneuron type, a pair of features is shown that can be used to discriminate that type from others in the same neuropil. Many although not all discriminations are highly accurate. Both intrinsic and boundary types are included as discriminative features. [file 41586_2024_7981_MOESM7_ESM.zip › DataS3/Dm8b.pdf]

Dm9

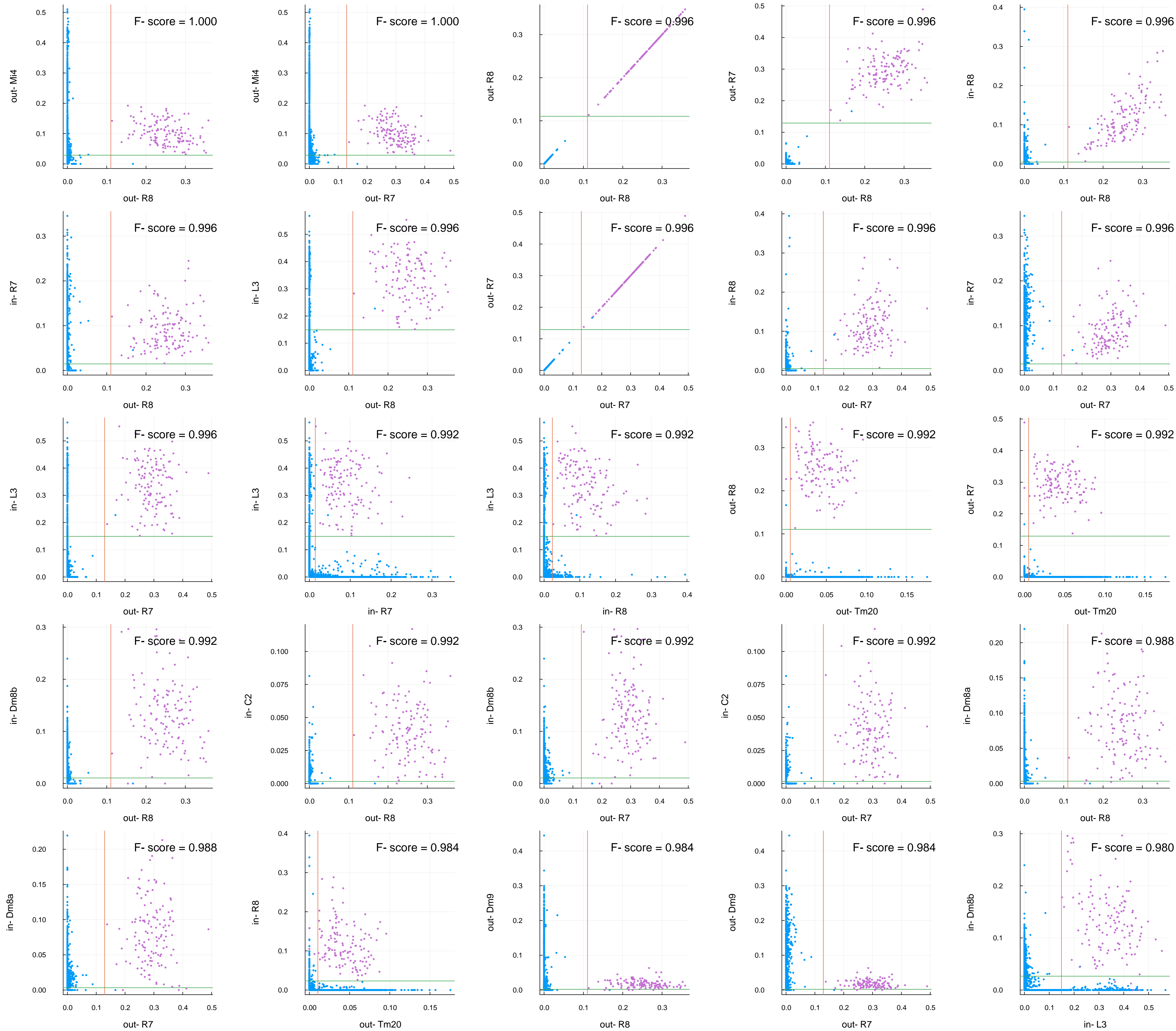

Supplement: Supplementary file 7 — Discriminating 2D projections for neuropil-intrinsic types. For each interneuron type, a pair of features is shown that can be used to discriminate that type from others in the same neuropil. Many although not all discriminations are highly accurate. Both intrinsic and boundary types are included as discriminative features. [file 41586_2024_7981_MOESM7_ESM.zip › DataS3/Dm9.pdf]

# DmDRA1

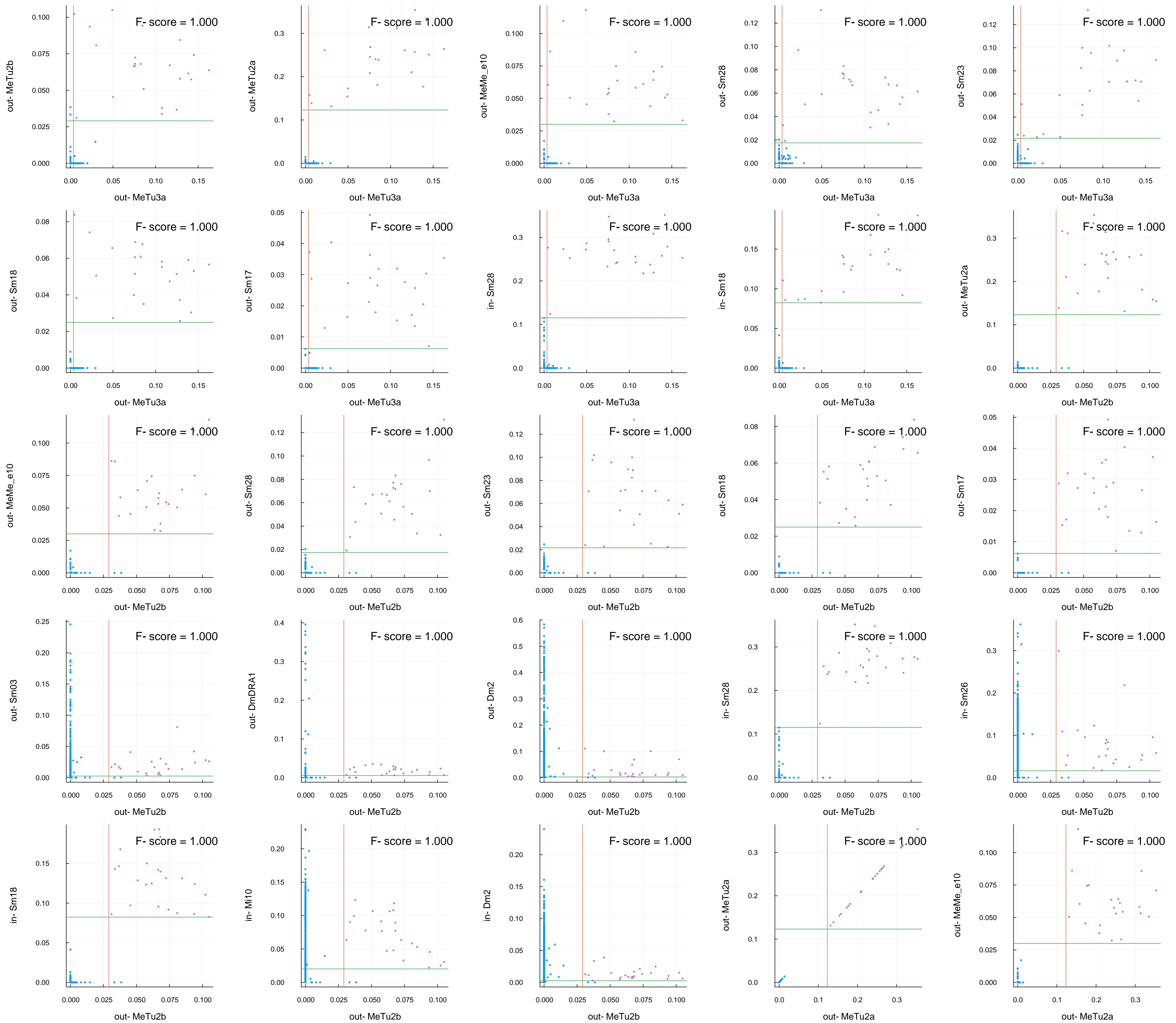

Supplement: Supplementary file 7 — Discriminating 2D projections for neuropil-intrinsic types. For each interneuron type, a pair of features is shown that can be used to discriminate that type from others in the same neuropil. Many although not all discriminations are highly accurate. Both intrinsic and boundary types are included as discriminative features. [file 41586_2024_7981_MOESM7_ESM.zip › DataS3/DmDRA1.pdf]

## DmDRA2

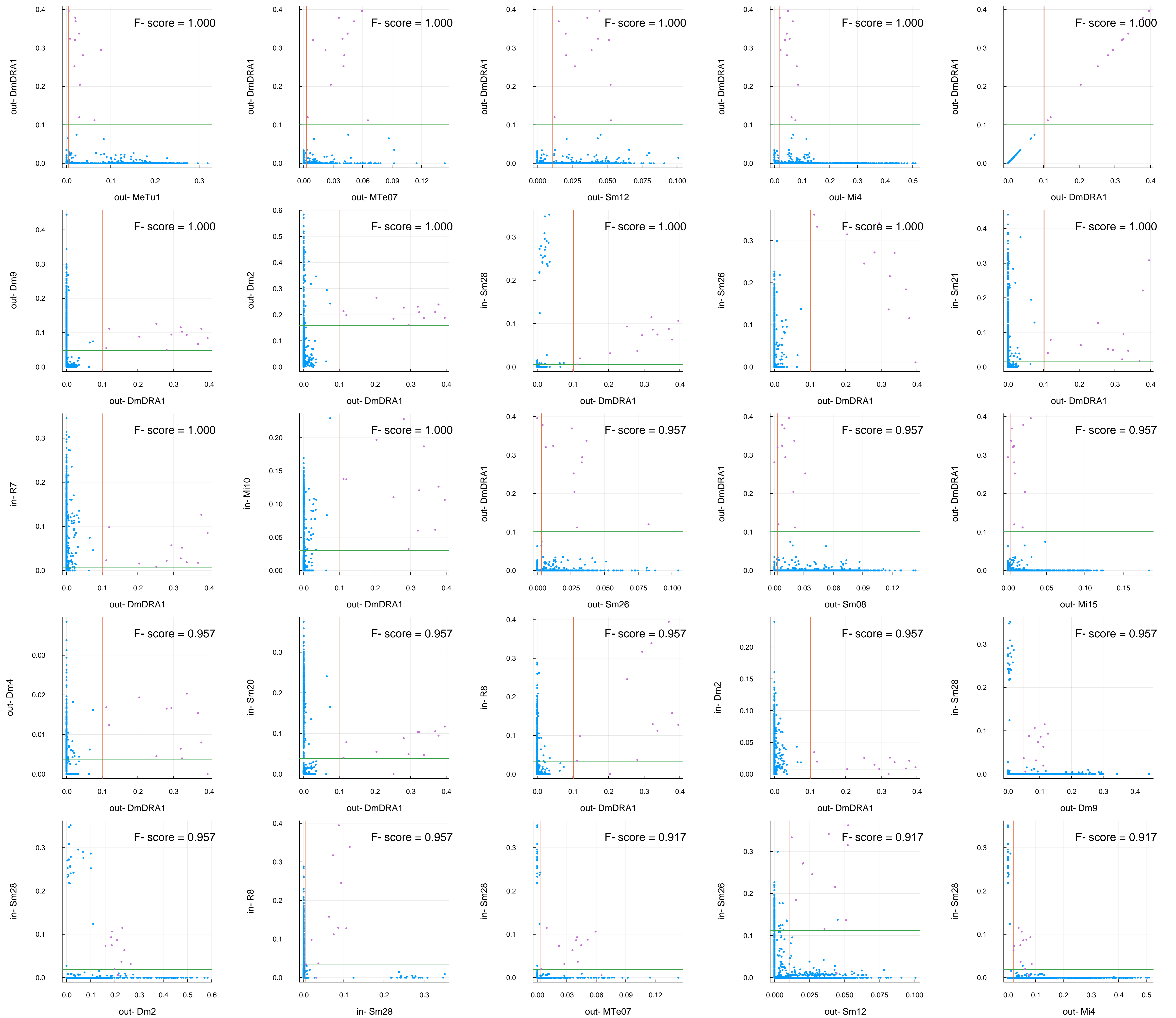

Supplement: Supplementary file 7 — Discriminating 2D projections for neuropil-intrinsic types. For each interneuron type, a pair of features is shown that can be used to discriminate that type from others in the same neuropil. Many although not all discriminations are highly accurate. Both intrinsic and boundary types are included as discriminative features. [file 41586_2024_7981_MOESM7_ESM.zip › DataS3/DmDRA2.pdf]

L1

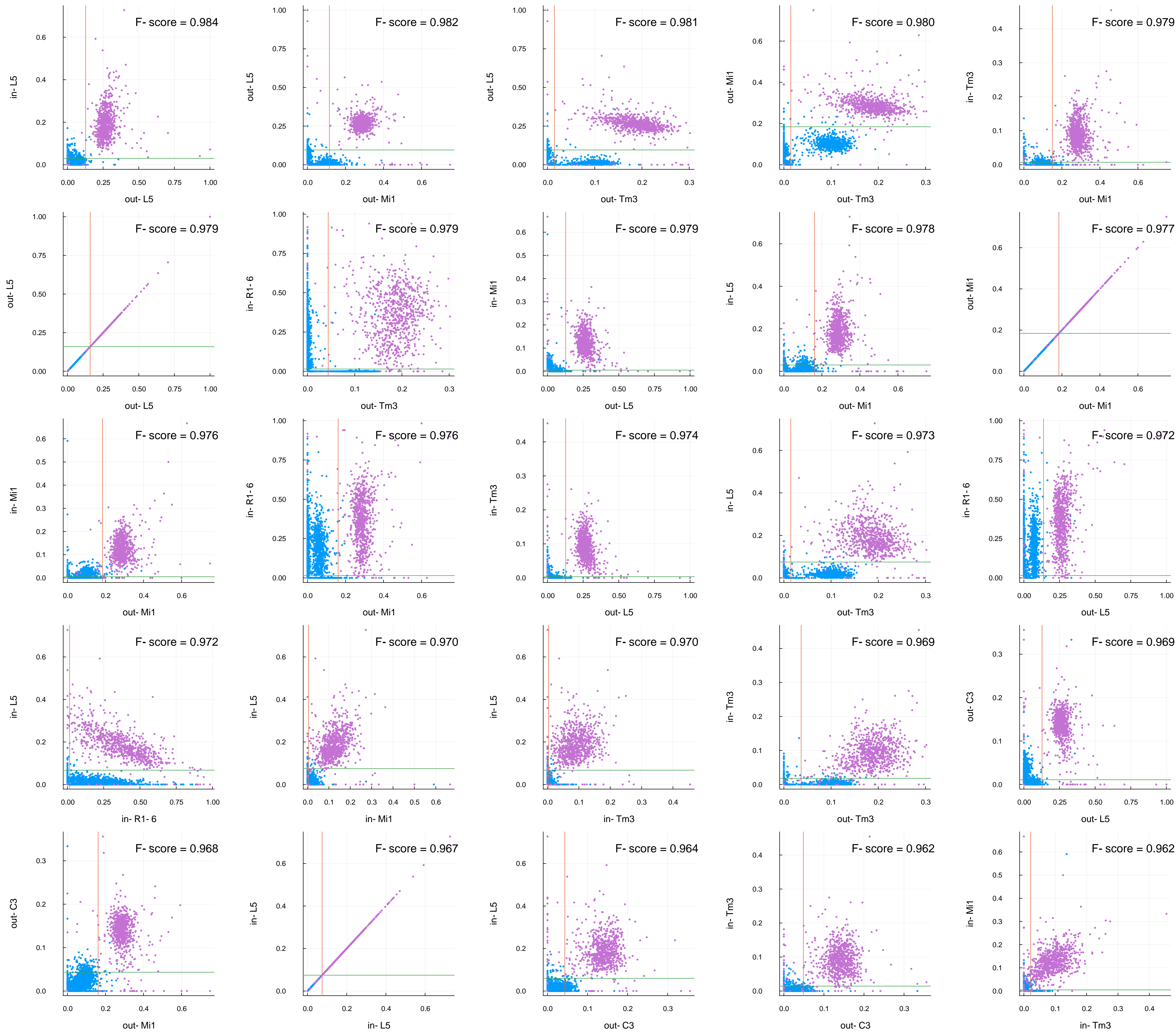

Supplement: Supplementary file 7 — Discriminating 2D projections for neuropil-intrinsic types. For each interneuron type, a pair of features is shown that can be used to discriminate that type from others in the same neuropil. Many although not all discriminations are highly accurate. Both intrinsic and boundary types are included as discriminative features. [file 41586_2024_7981_MOESM7_ESM.zip › DataS3/L1.pdf]

L2

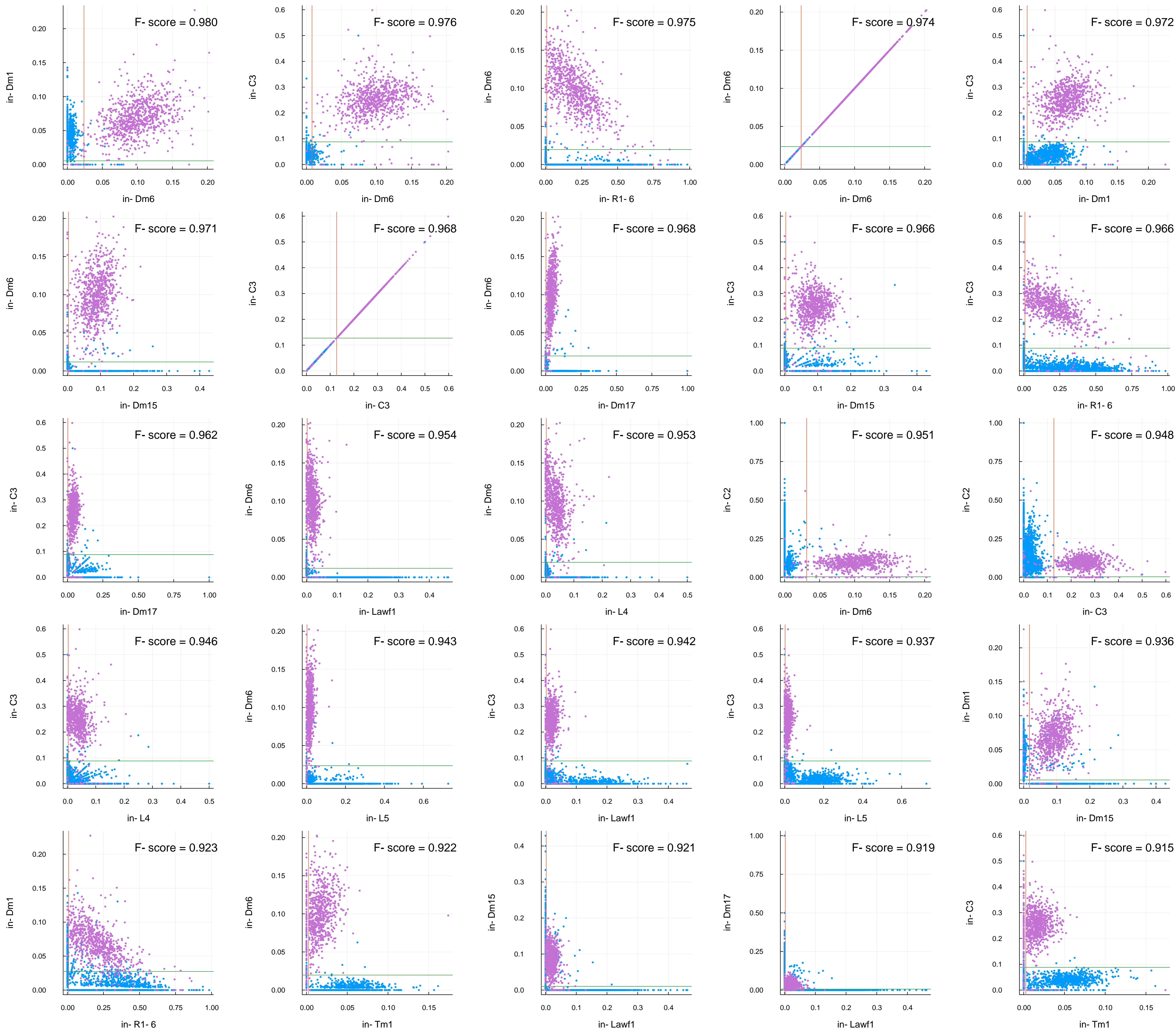

Supplement: Supplementary file 7 — Discriminating 2D projections for neuropil-intrinsic types. For each interneuron type, a pair of features is shown that can be used to discriminate that type from others in the same neuropil. Many although not all discriminations are highly accurate. Both intrinsic and boundary types are included as discriminative features. [file 41586_2024_7981_MOESM7_ESM.zip › DataS3/L2.pdf]

L3

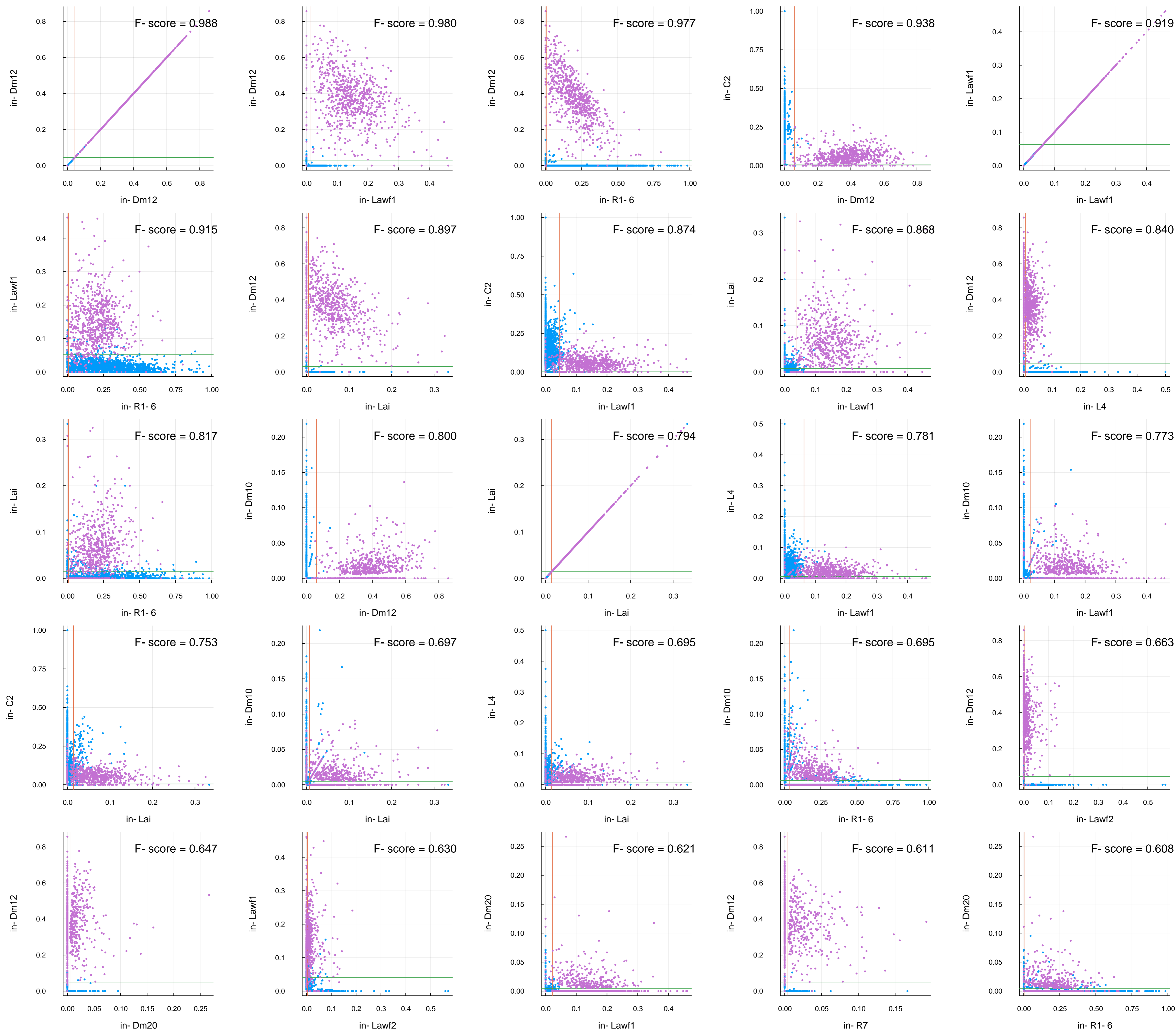

Supplement: Supplementary file 7 — Discriminating 2D projections for neuropil-intrinsic types. For each interneuron type, a pair of features is shown that can be used to discriminate that type from others in the same neuropil. Many although not all discriminations are highly accurate. Both intrinsic and boundary types are included as discriminative features. [file 41586_2024_7981_MOESM7_ESM.zip › DataS3/L3.pdf]

L4

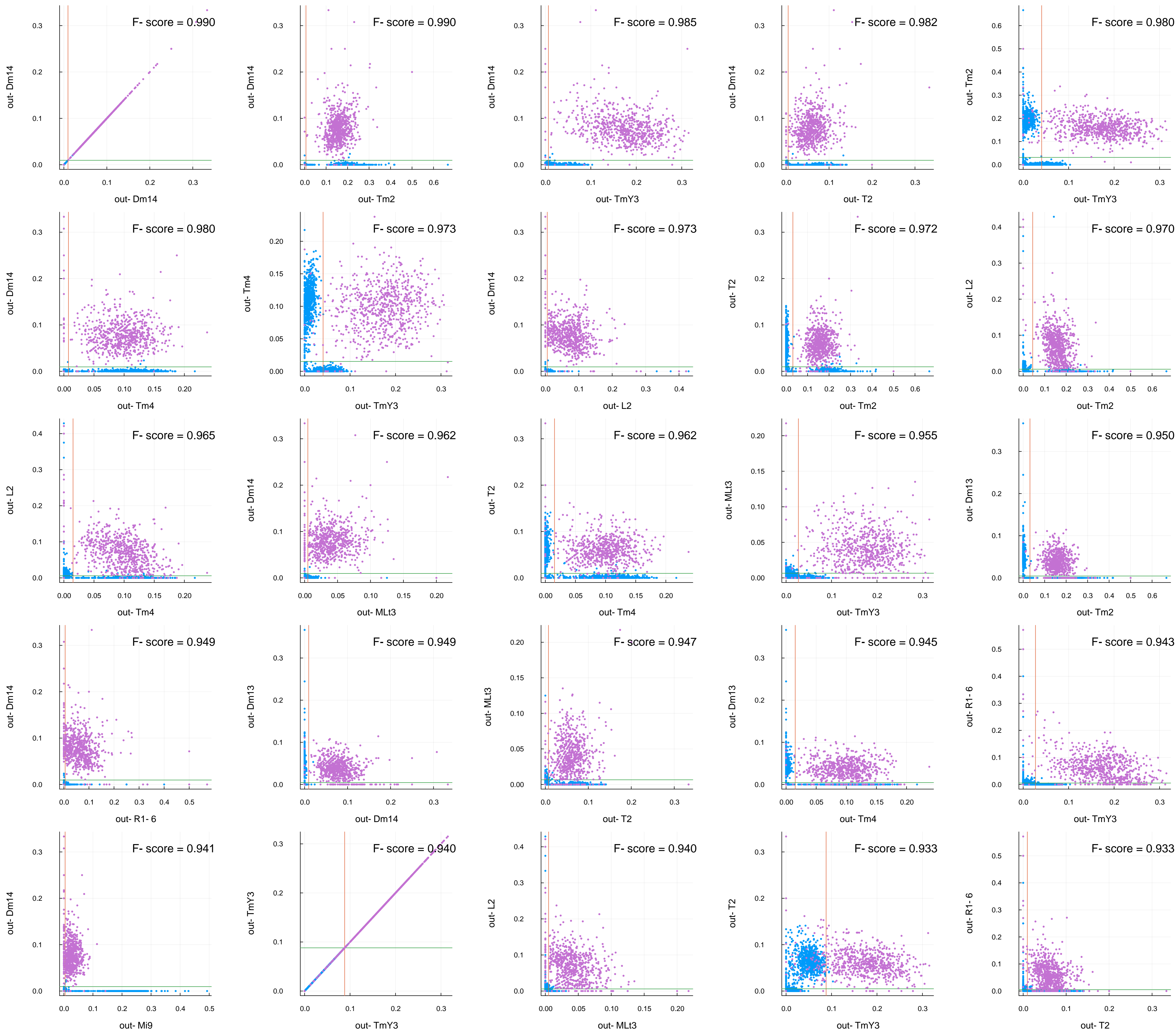

Supplement: Supplementary file 7 — Discriminating 2D projections for neuropil-intrinsic types. For each interneuron type, a pair of features is shown that can be used to discriminate that type from others in the same neuropil. Many although not all discriminations are highly accurate. Both intrinsic and boundary types are included as discriminative features. [file 41586_2024_7981_MOESM7_ESM.zip › DataS3/L4.pdf]

L5

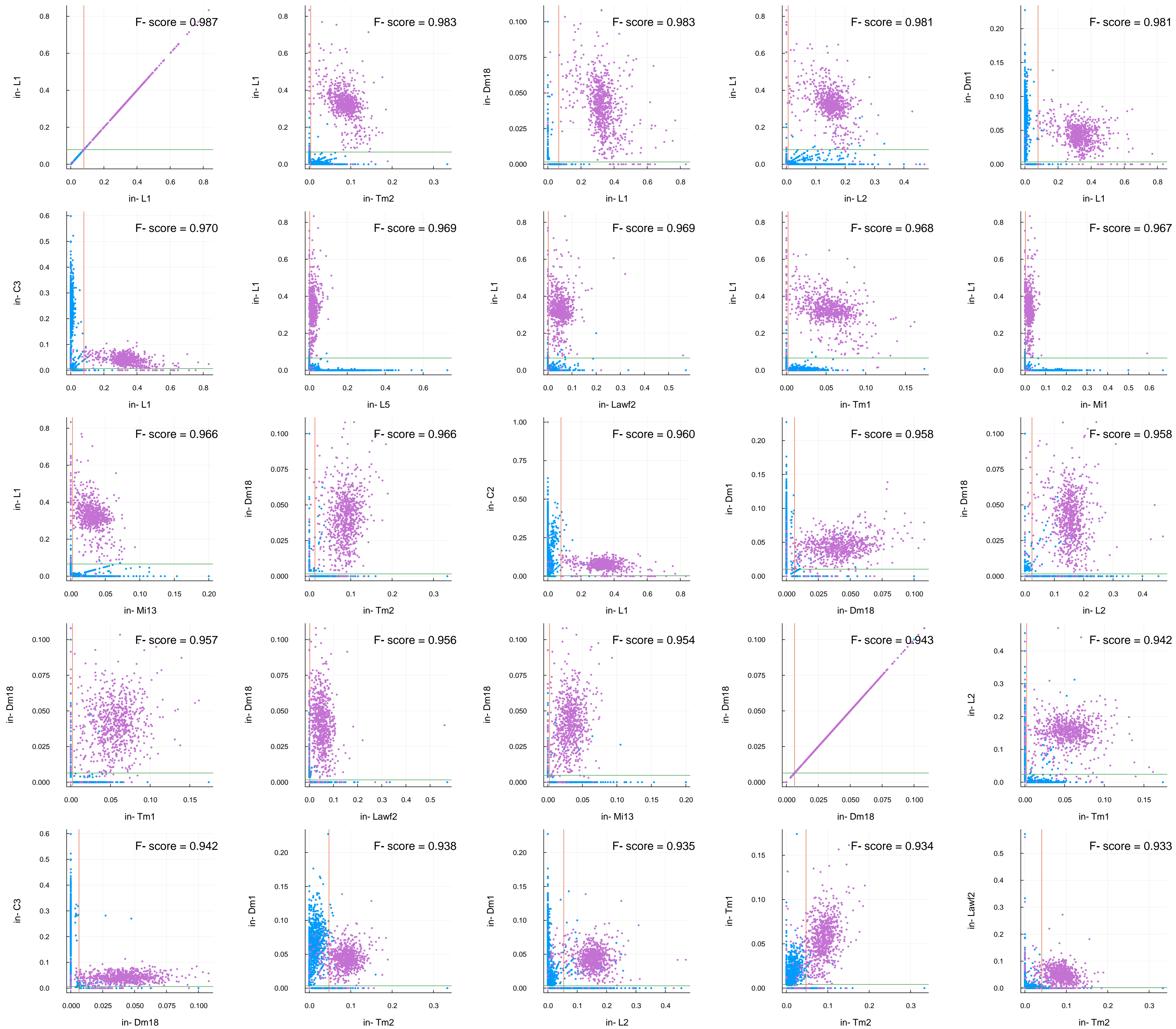

Supplement: Supplementary file 7 — Discriminating 2D projections for neuropil-intrinsic types. For each interneuron type, a pair of features is shown that can be used to discriminate that type from others in the same neuropil. Many although not all discriminations are highly accurate. Both intrinsic and boundary types are included as discriminative features. [file 41586_2024_7981_MOESM7_ESM.zip › DataS3/L5.pdf]

Lawf1

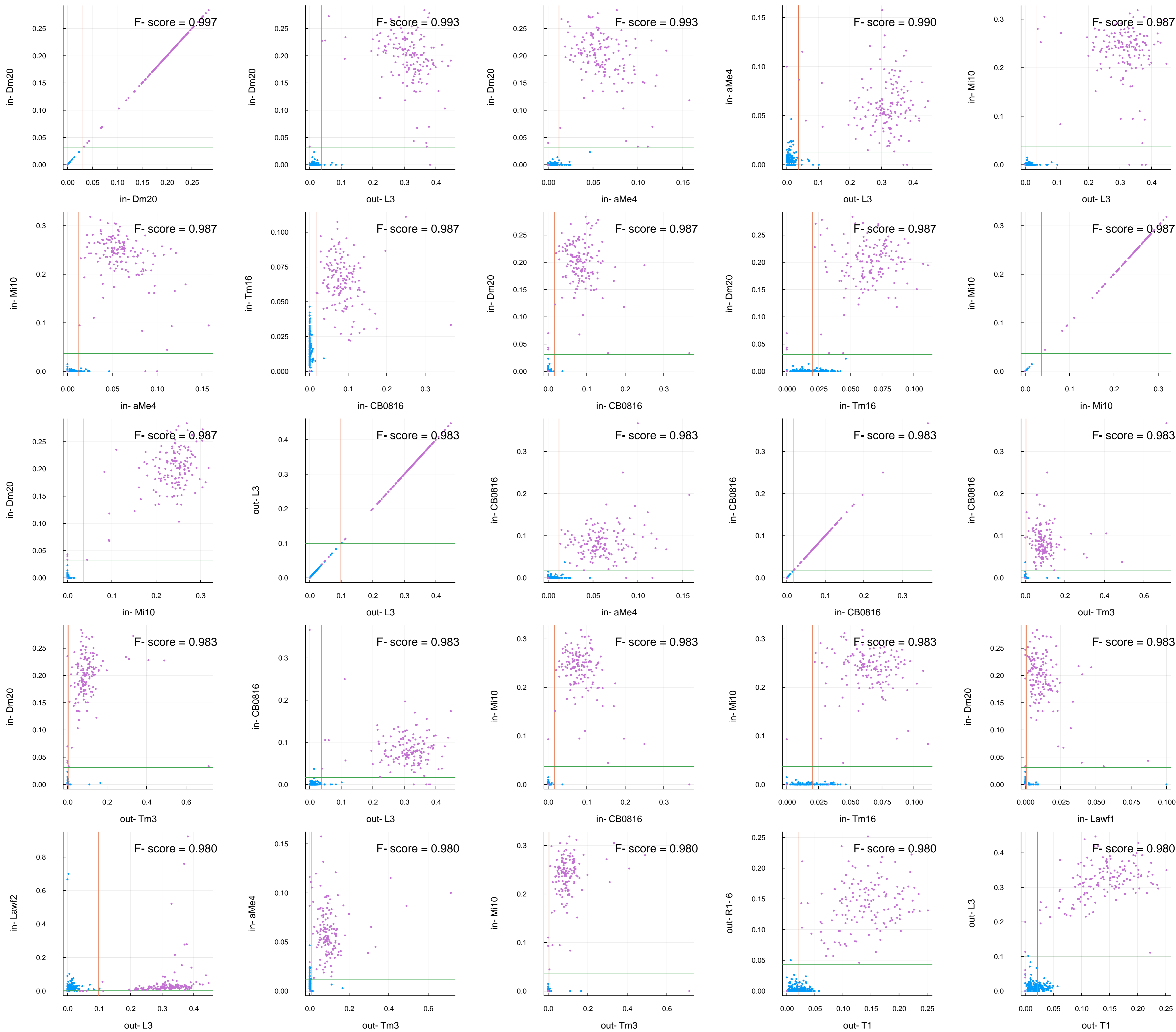

Supplement: Supplementary file 7 — Discriminating 2D projections for neuropil-intrinsic types. For each interneuron type, a pair of features is shown that can be used to discriminate that type from others in the same neuropil. Many although not all discriminations are highly accurate. Both intrinsic and boundary types are included as discriminative features. [file 41586_2024_7981_MOESM7_ESM.zip › DataS3/Lawf1.pdf]

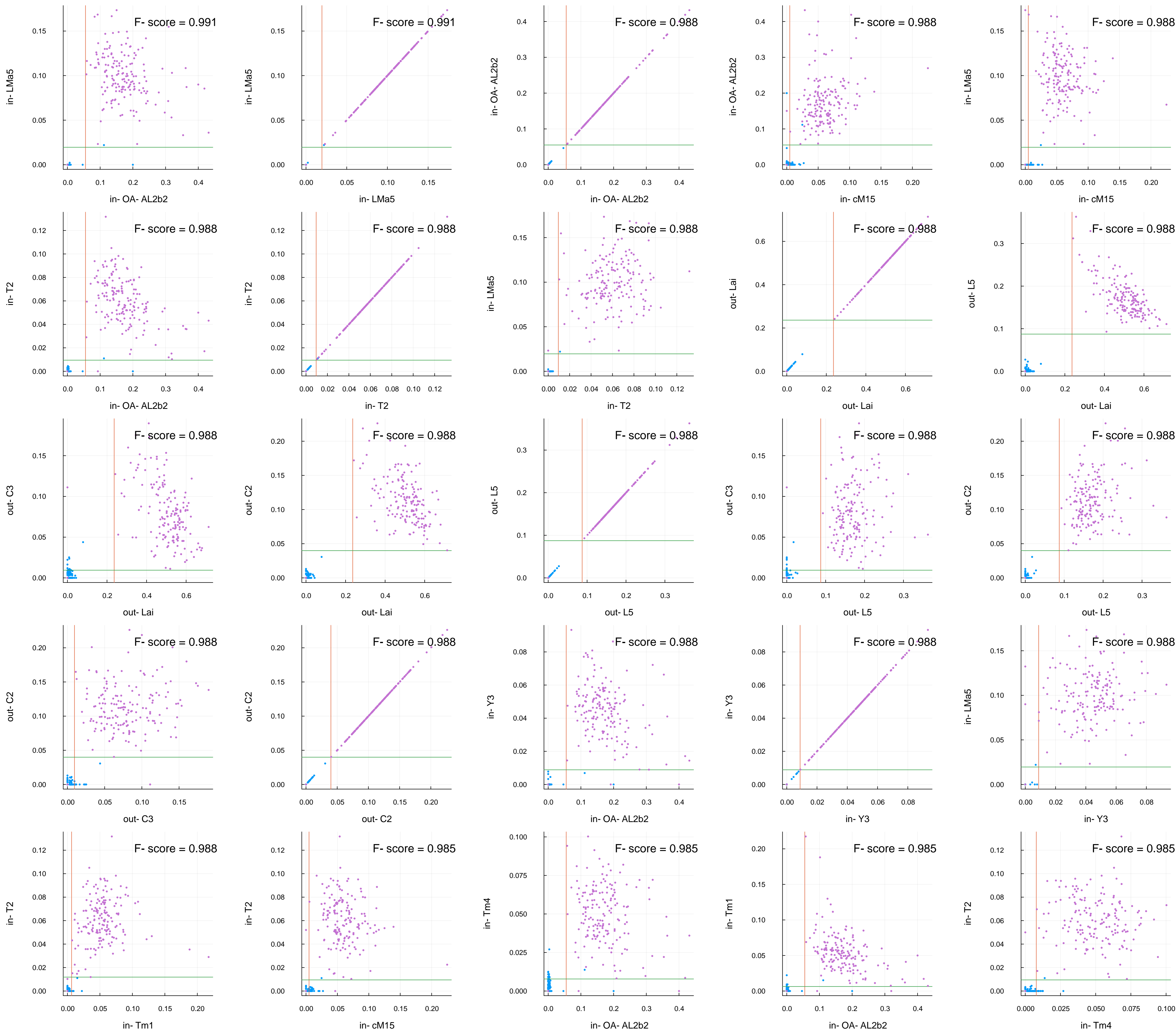

Supplement: Supplementary file 7 — Discriminating 2D projections for neuropil-intrinsic types. For each interneuron type, a pair of features is shown that can be used to discriminate that type from others in the same neuropil. Many although not all discriminations are highly accurate. Both intrinsic and boundary types are included as discriminative features. [file 41586_2024_7981_MOESM7_ESM.zip › DataS3/Lawf2.pdf]

Li01

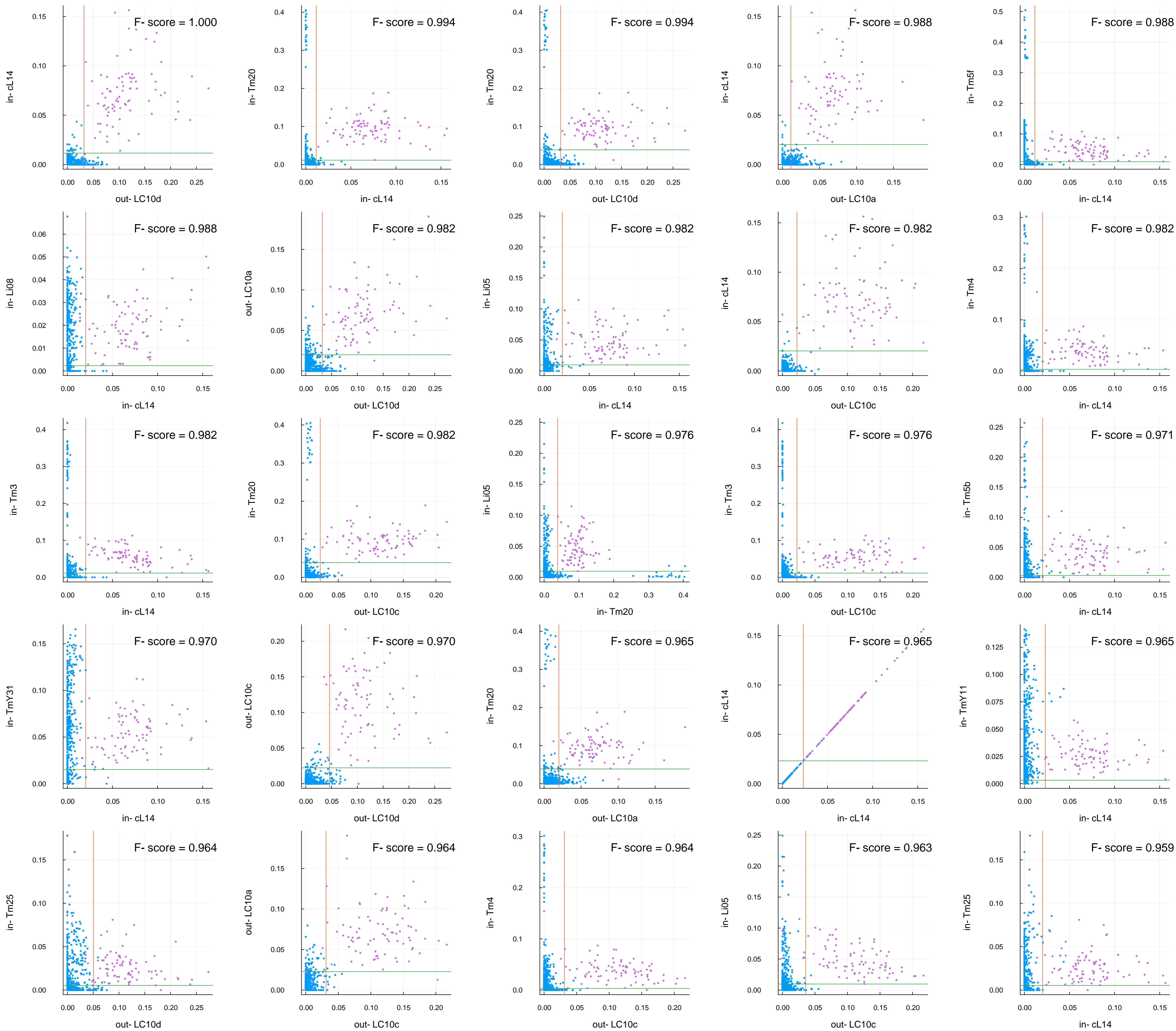

Supplement: Supplementary file 7 — Discriminating 2D projections for neuropil-intrinsic types. For each interneuron type, a pair of features is shown that can be used to discriminate that type from others in the same neuropil. Many although not all discriminations are highly accurate. Both intrinsic and boundary types are included as discriminative features. [file 41586_2024_7981_MOESM7_ESM.zip › DataS3/Li01.pdf]

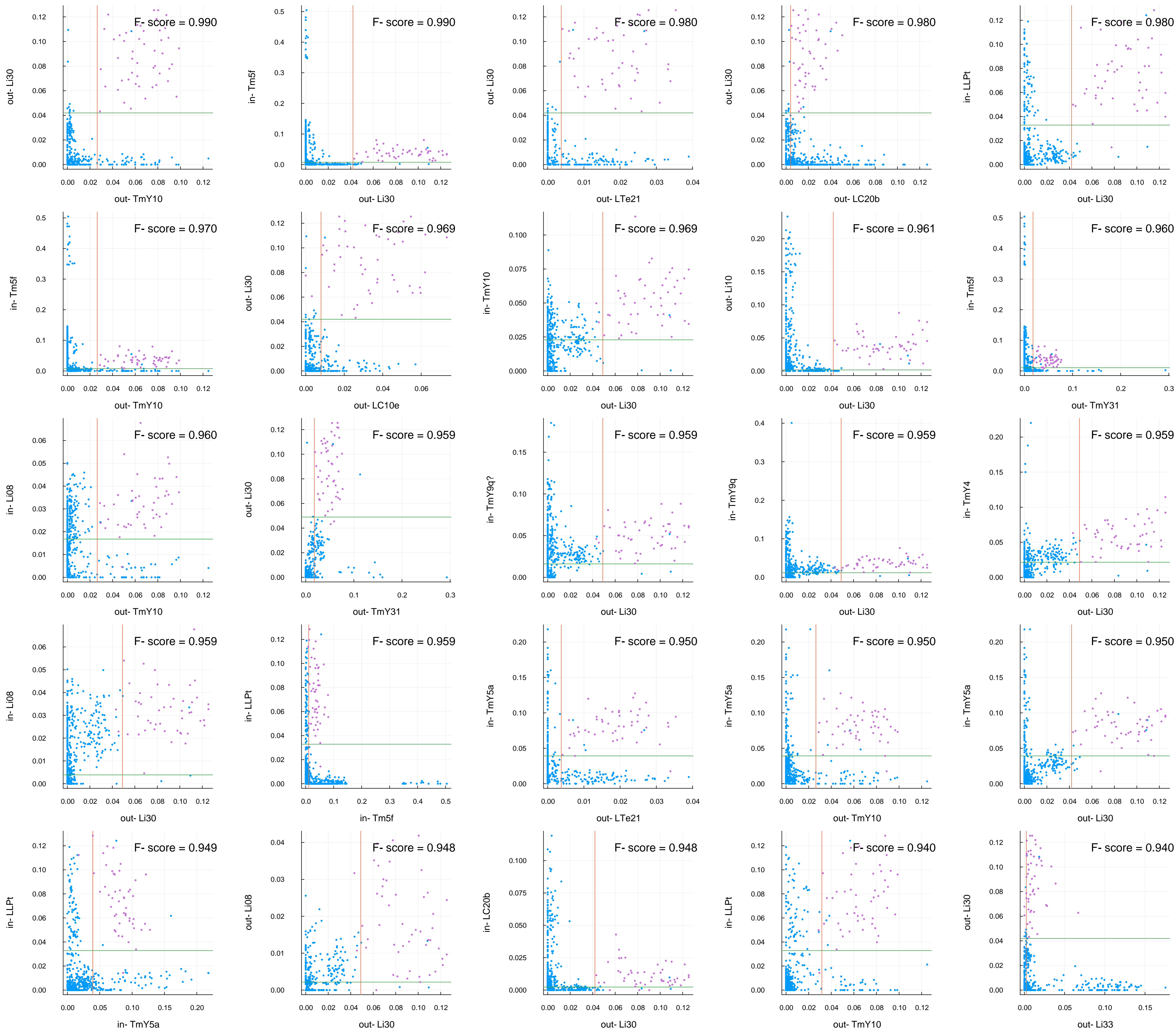

Supplement: Supplementary file 7 — Discriminating 2D projections for neuropil-intrinsic types. For each interneuron type, a pair of features is shown that can be used to discriminate that type from others in the same neuropil. Many although not all discriminations are highly accurate. Both intrinsic and boundary types are included as discriminative features. [file 41586_2024_7981_MOESM7_ESM.zip › DataS3/Li02.pdf]

Li03

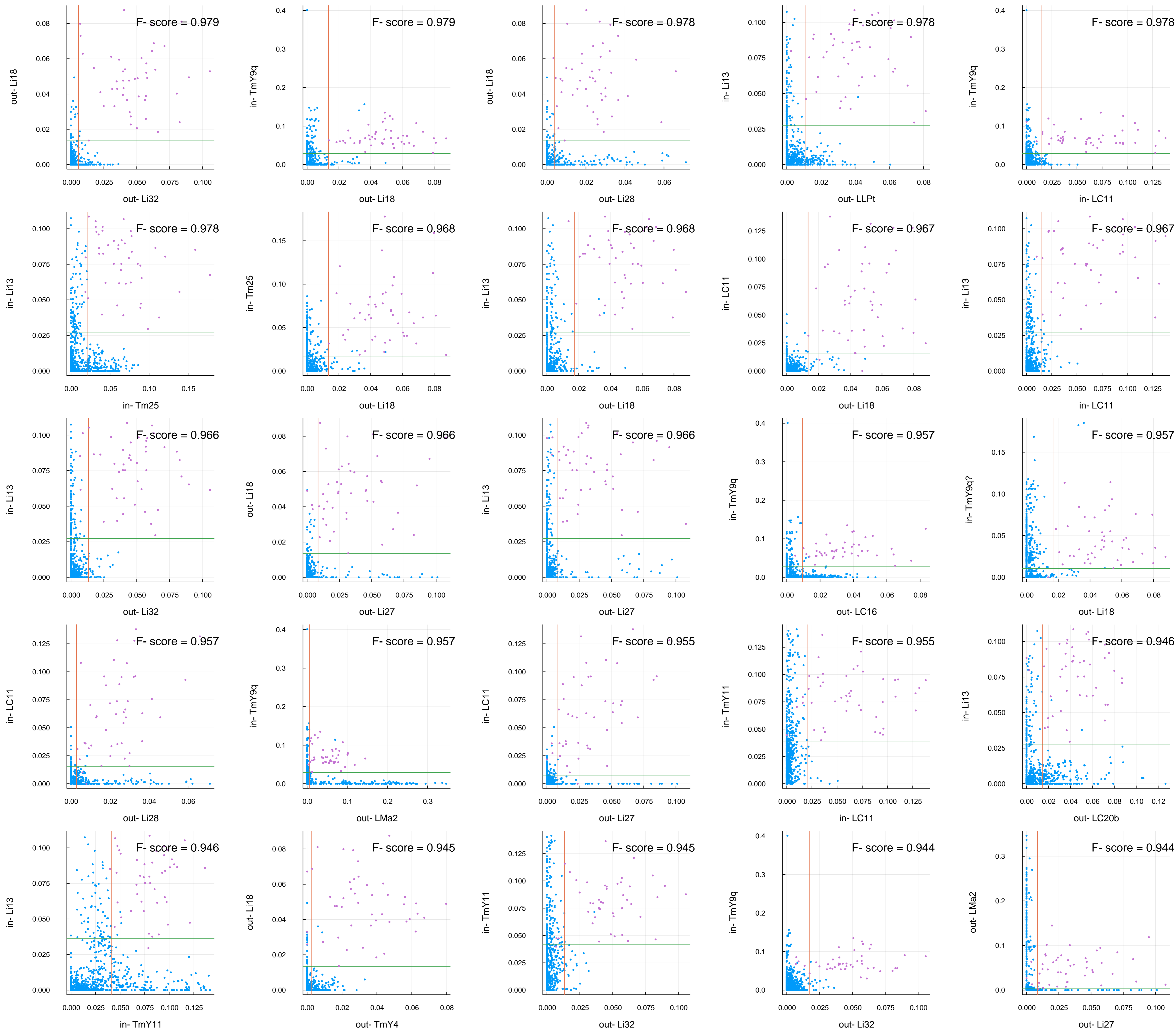

Supplement: Supplementary file 7 — Discriminating 2D projections for neuropil-intrinsic types. For each interneuron type, a pair of features is shown that can be used to discriminate that type from others in the same neuropil. Many although not all discriminations are highly accurate. Both intrinsic and boundary types are included as discriminative features. [file 41586_2024_7981_MOESM7_ESM.zip › DataS3/Li03.pdf]

Li04

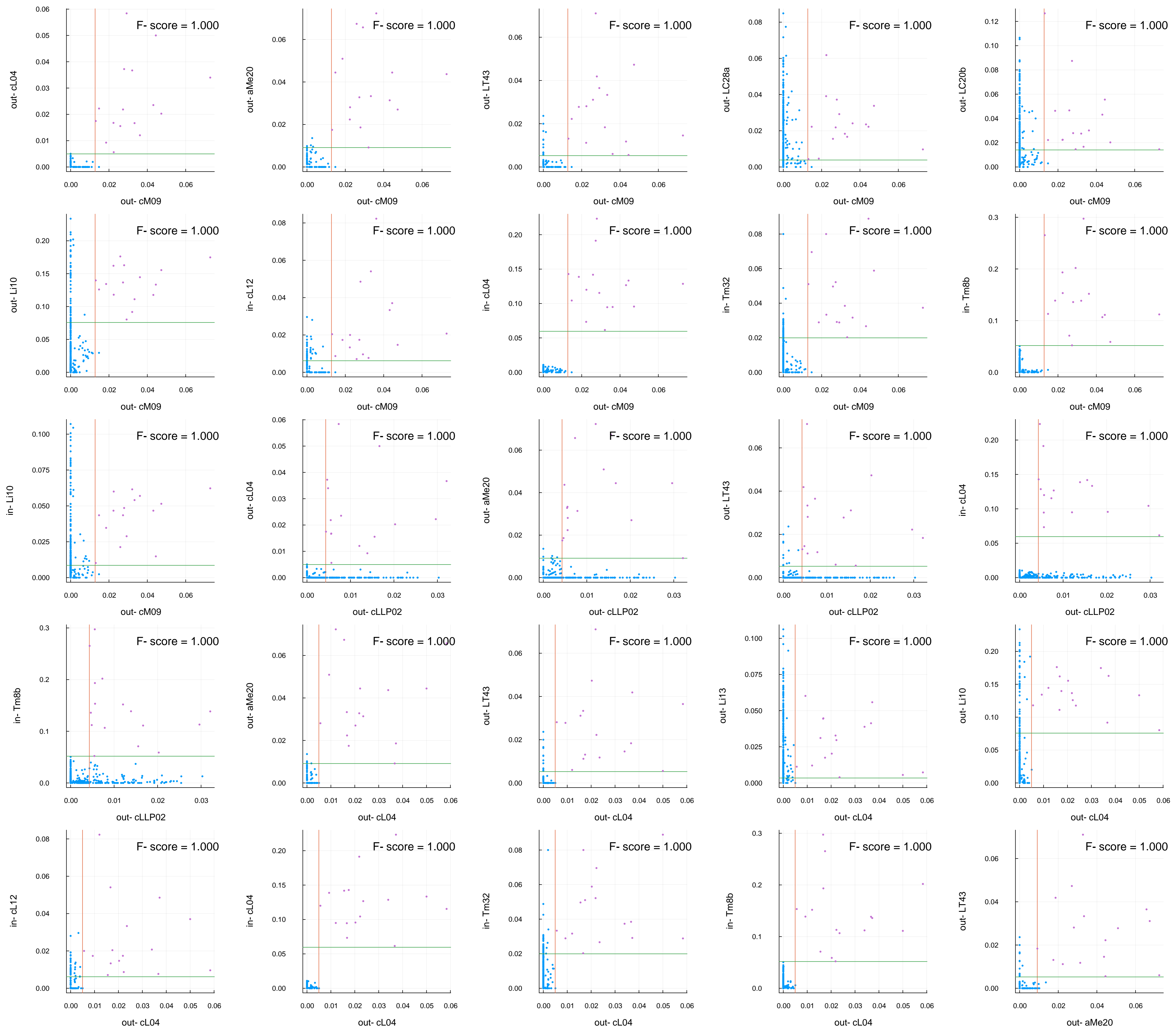

Supplement: Supplementary file 7 — Discriminating 2D projections for neuropil-intrinsic types. For each interneuron type, a pair of features is shown that can be used to discriminate that type from others in the same neuropil. Many although not all discriminations are highly accurate. Both intrinsic and boundary types are included as discriminative features. [file 41586_2024_7981_MOESM7_ESM.zip › DataS3/Li04.pdf]

Li05

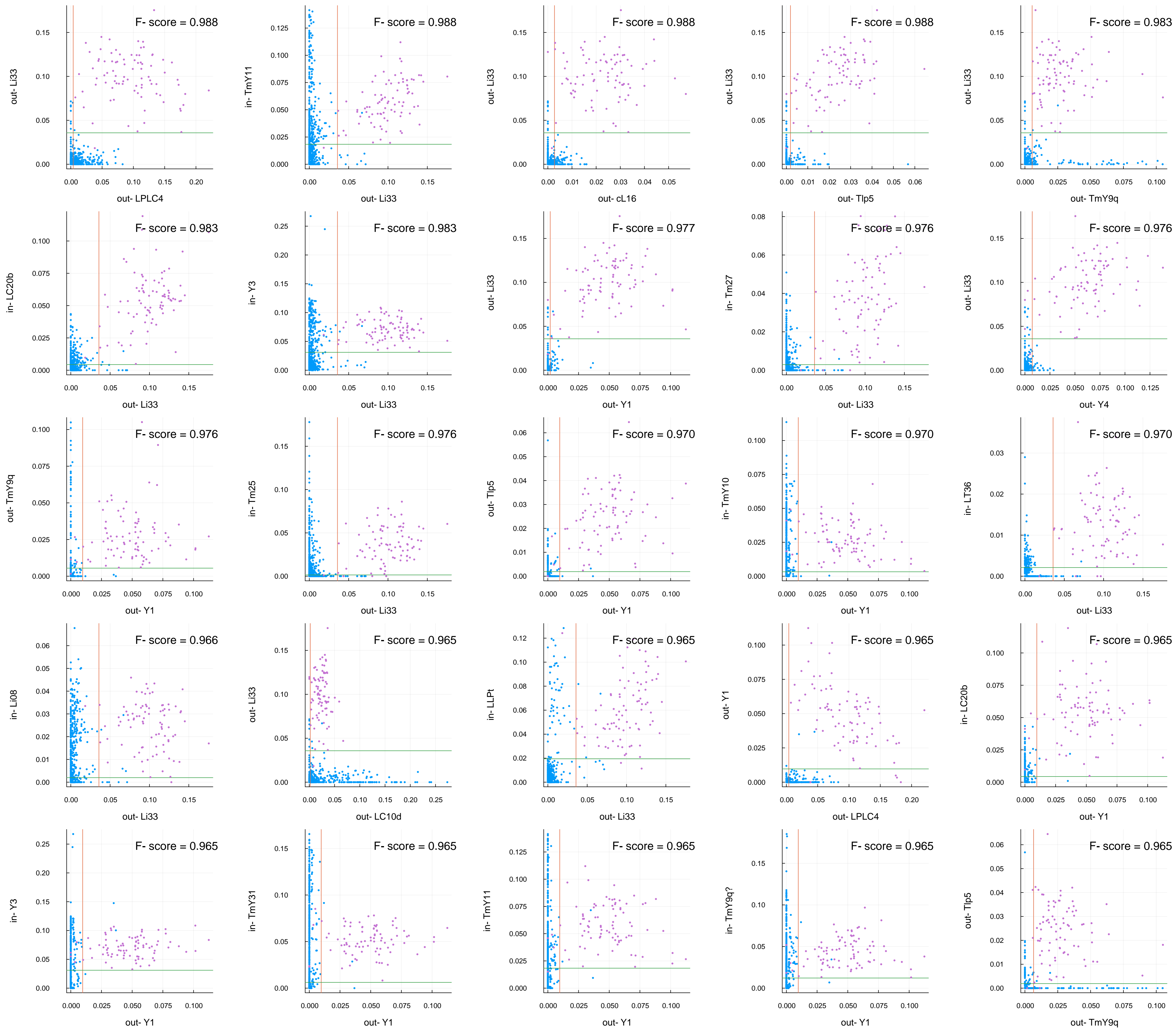

Supplement: Supplementary file 7 — Discriminating 2D projections for neuropil-intrinsic types. For each interneuron type, a pair of features is shown that can be used to discriminate that type from others in the same neuropil. Many although not all discriminations are highly accurate. Both intrinsic and boundary types are included as discriminative features. [file 41586_2024_7981_MOESM7_ESM.zip › DataS3/Li05.pdf]

Li06

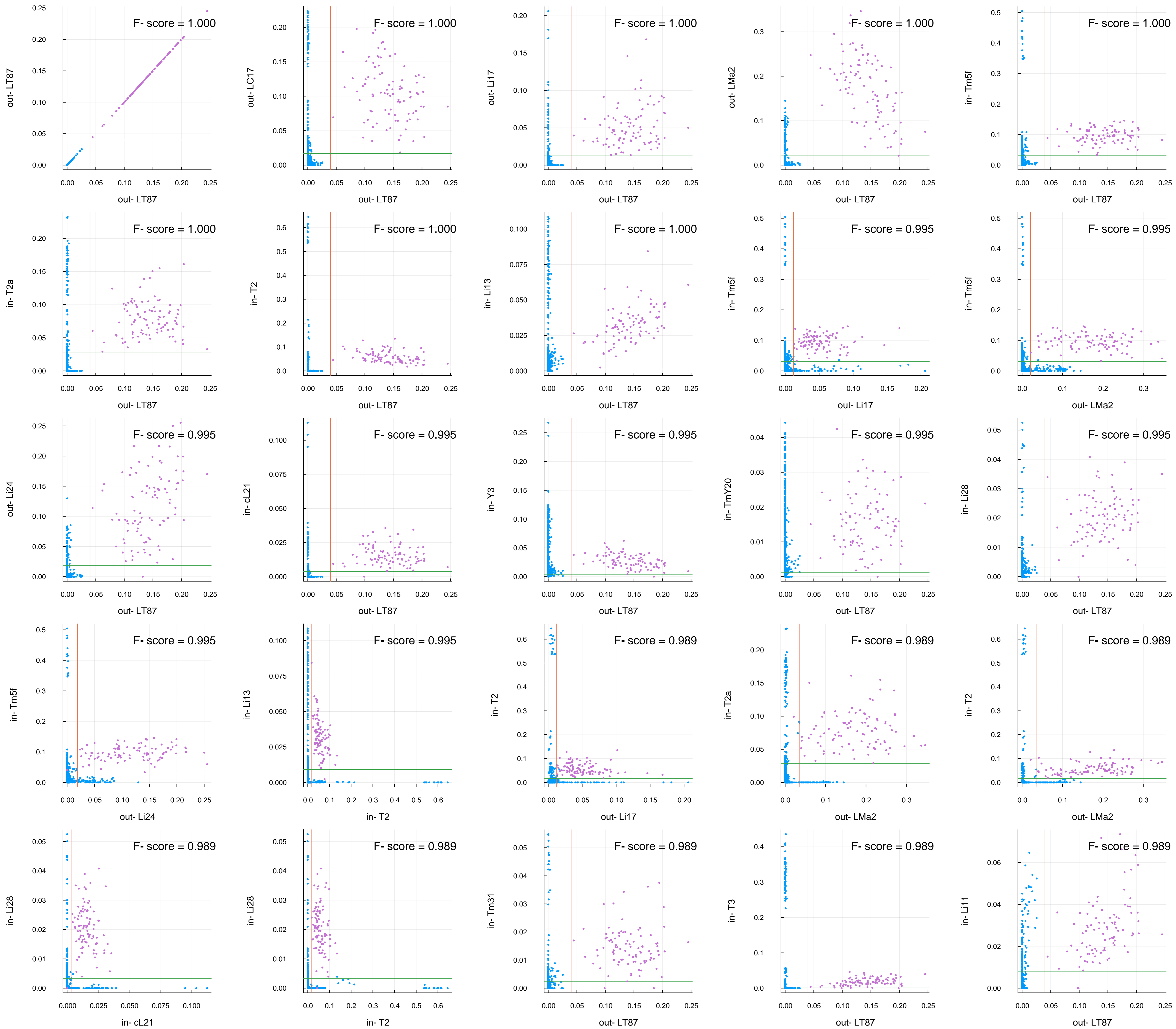

Supplement: Supplementary file 7 — Discriminating 2D projections for neuropil-intrinsic types. For each interneuron type, a pair of features is shown that can be used to discriminate that type from others in the same neuropil. Many although not all discriminations are highly accurate. Both intrinsic and boundary types are included as discriminative features. [file 41586_2024_7981_MOESM7_ESM.zip › DataS3/Li06.pdf]

Li07

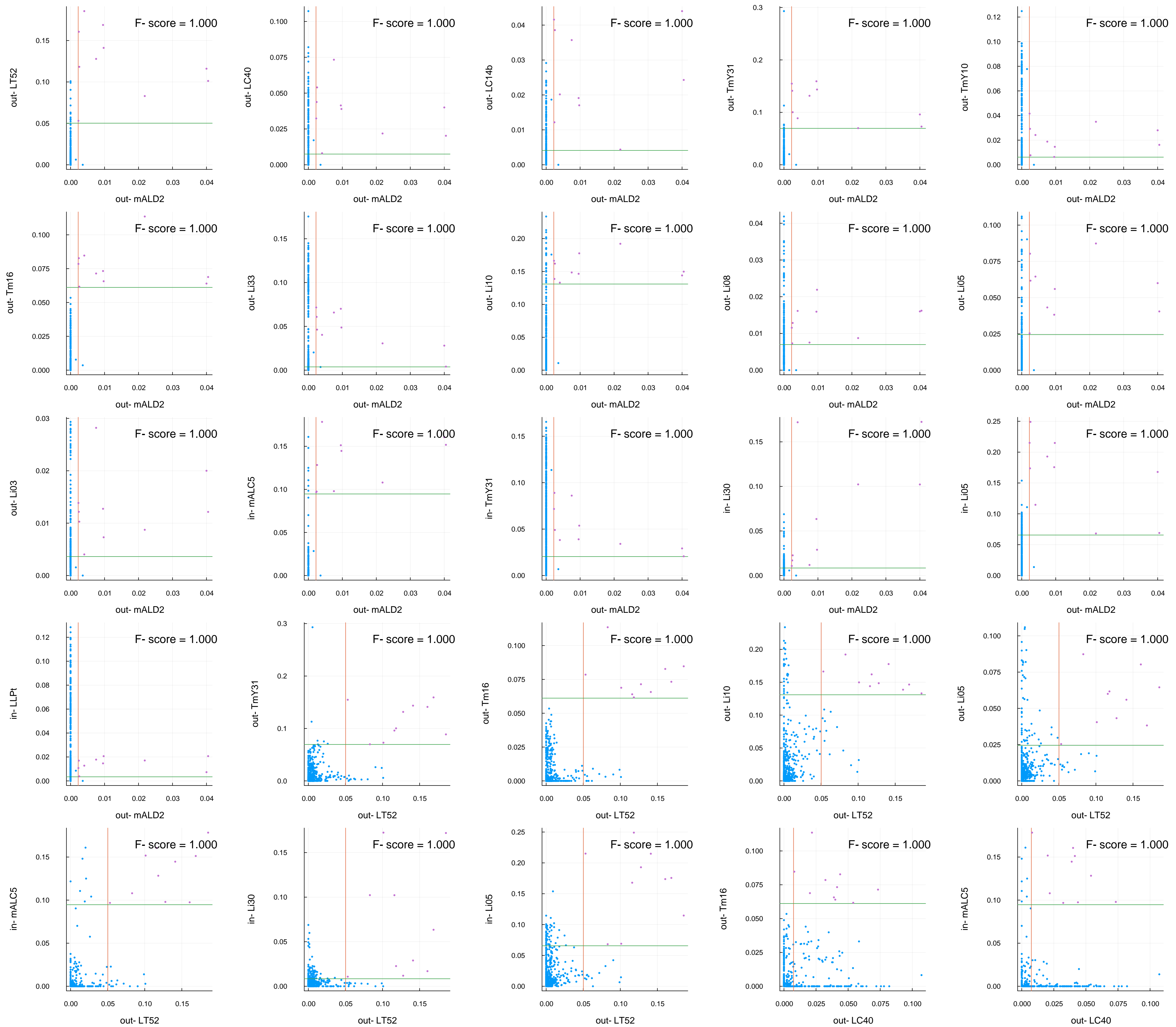

Supplement: Supplementary file 7 — Discriminating 2D projections for neuropil-intrinsic types. For each interneuron type, a pair of features is shown that can be used to discriminate that type from others in the same neuropil. Many although not all discriminations are highly accurate. Both intrinsic and boundary types are included as discriminative features. [file 41586_2024_7981_MOESM7_ESM.zip › DataS3/Li07.pdf]

Li08

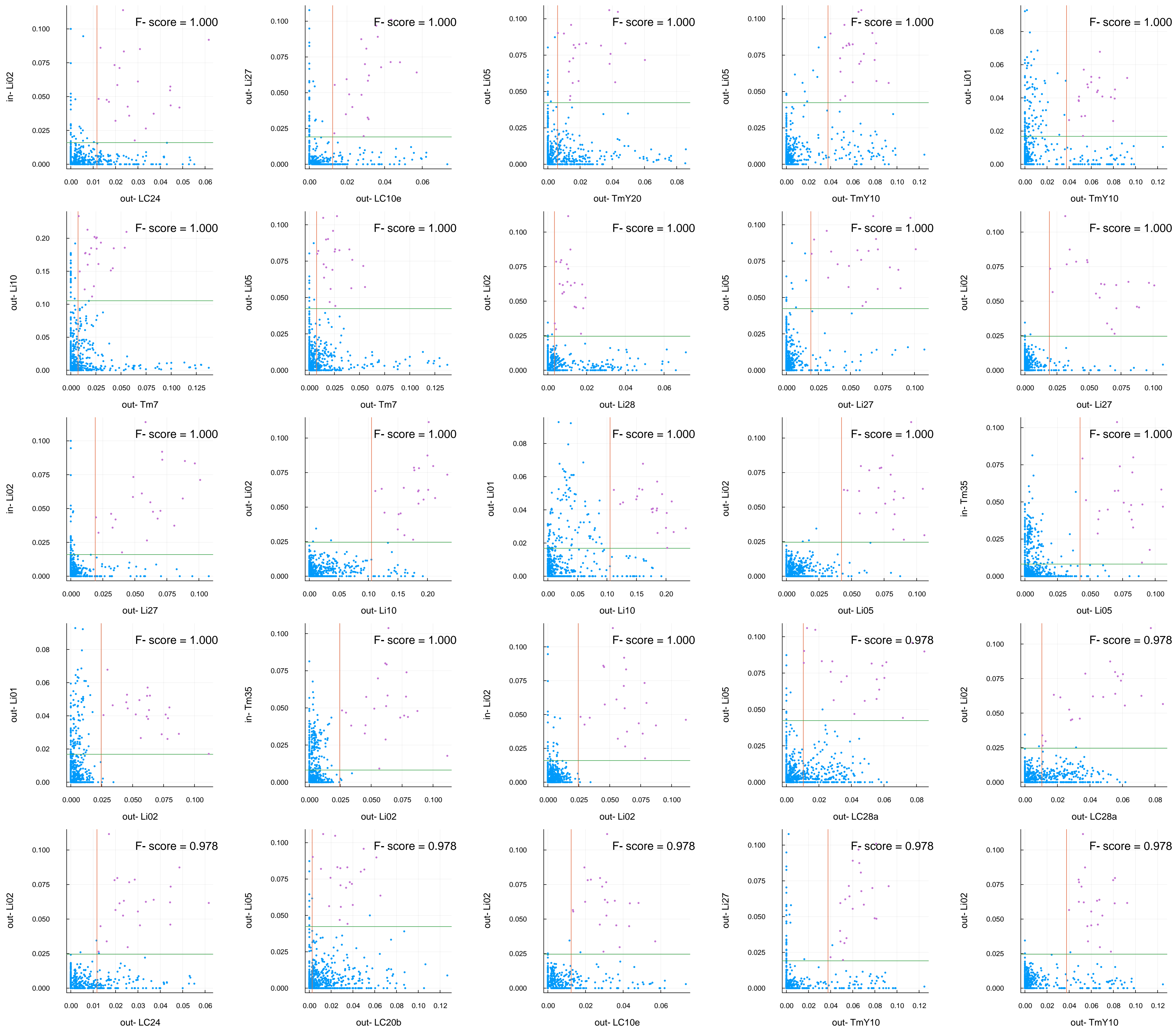

Supplement: Supplementary file 7 — Discriminating 2D projections for neuropil-intrinsic types. For each interneuron type, a pair of features is shown that can be used to discriminate that type from others in the same neuropil. Many although not all discriminations are highly accurate. Both intrinsic and boundary types are included as discriminative features. [file 41586_2024_7981_MOESM7_ESM.zip › DataS3/Li08.pdf]

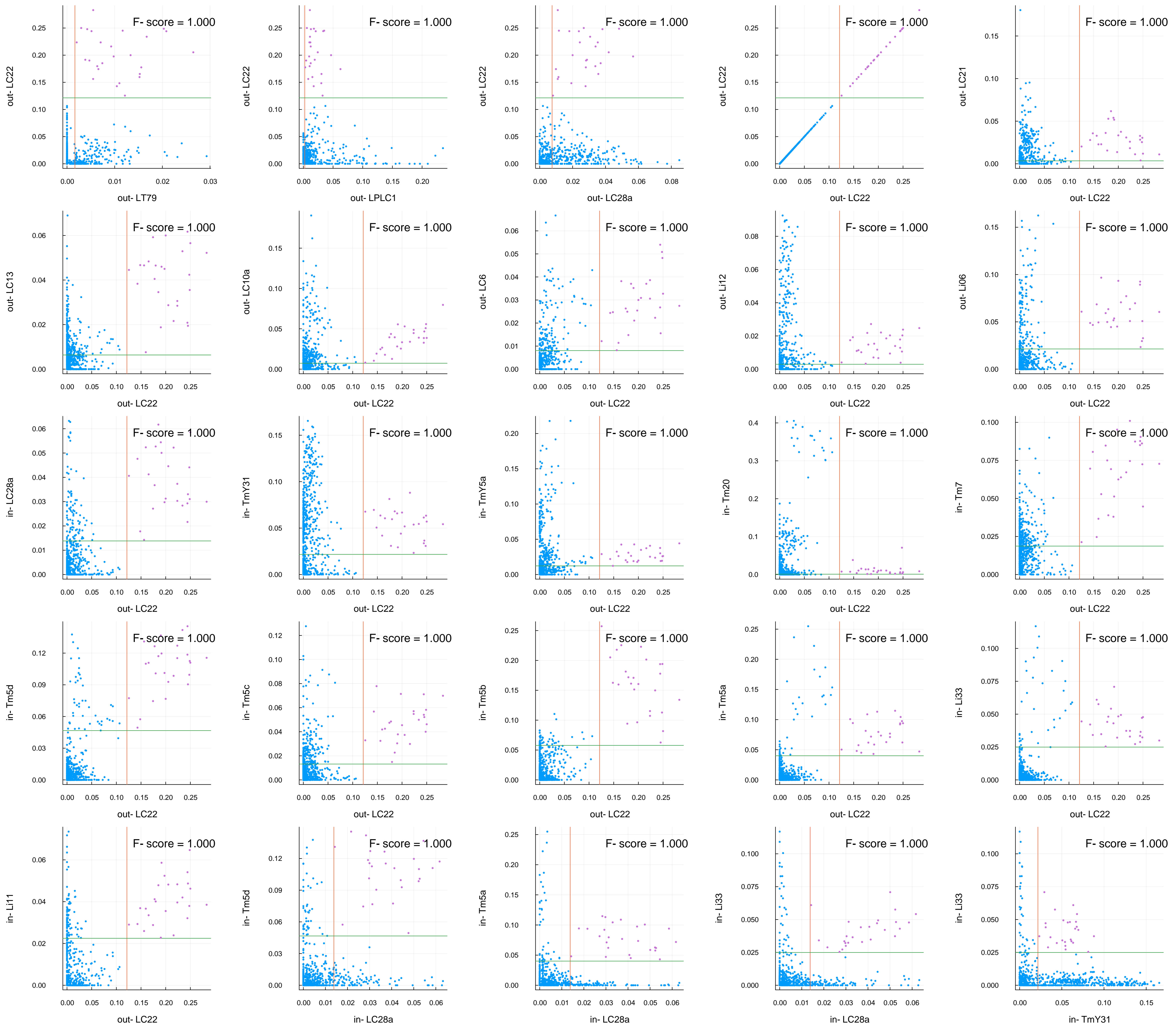

Supplement: Supplementary file 7 — Discriminating 2D projections for neuropil-intrinsic types. For each interneuron type, a pair of features is shown that can be used to discriminate that type from others in the same neuropil. Many although not all discriminations are highly accurate. Both intrinsic and boundary types are included as discriminative features. [file 41586_2024_7981_MOESM7_ESM.zip › DataS3/Li09.pdf]
