# Supplementary figures and images for: Neuronal parts list and wiring diagram for a visual system (part 3 of 3)
Source: Nature. 2024 Oct 2;634(8032):166–80. doi: 10.1038/s41586-024-07981-1 (PMC11446827; doi:10.1038/s41586-024-07981-1)

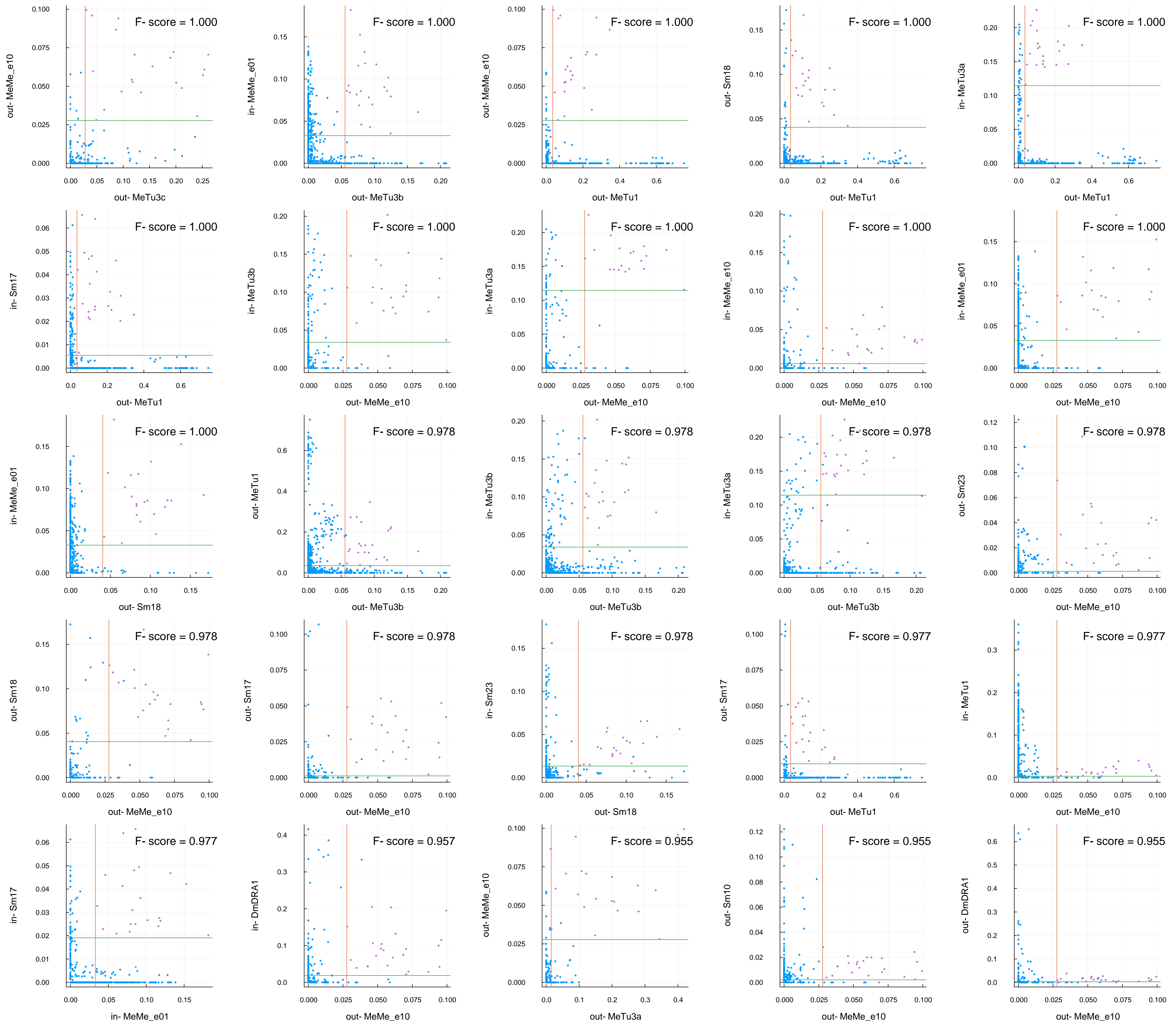

Supplement: Supplementary file 7 — Discriminating 2D projections for neuropil-intrinsic types. For each interneuron type, a pair of features is shown that can be used to discriminate that type from others in the same neuropil. Many although not all discriminations are highly accurate. Both intrinsic and boundary types are included as discriminative features. [file 41586_2024_7981_MOESM7_ESM.zip › DataS3/Sm23.pdf]

Sm24

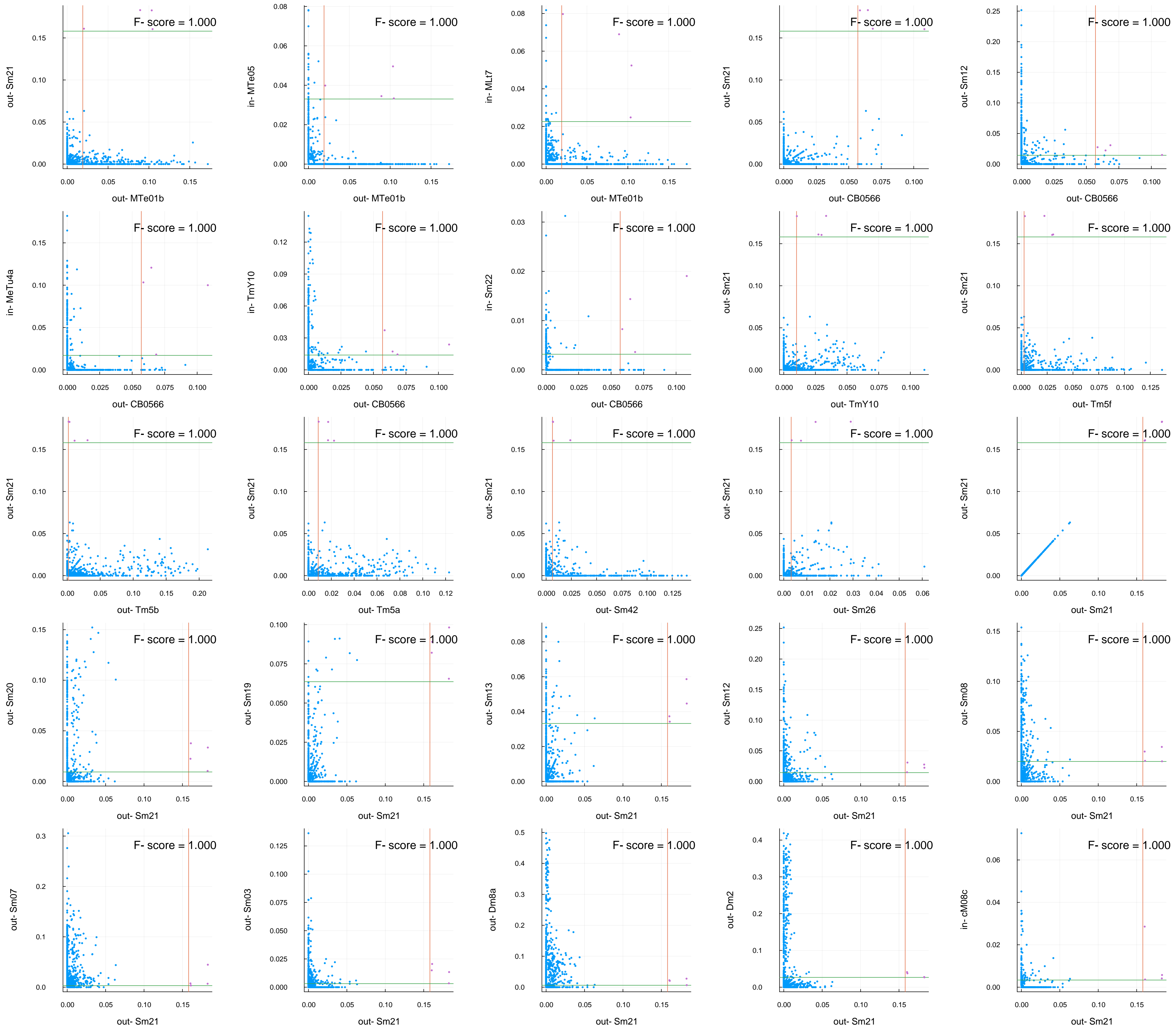

Supplement: Supplementary file 7 — Discriminating 2D projections for neuropil-intrinsic types. For each interneuron type, a pair of features is shown that can be used to discriminate that type from others in the same neuropil. Many although not all discriminations are highly accurate. Both intrinsic and boundary types are included as discriminative features. [file 41586_2024_7981_MOESM7_ESM.zip › DataS3/Sm24.pdf]

Sm25

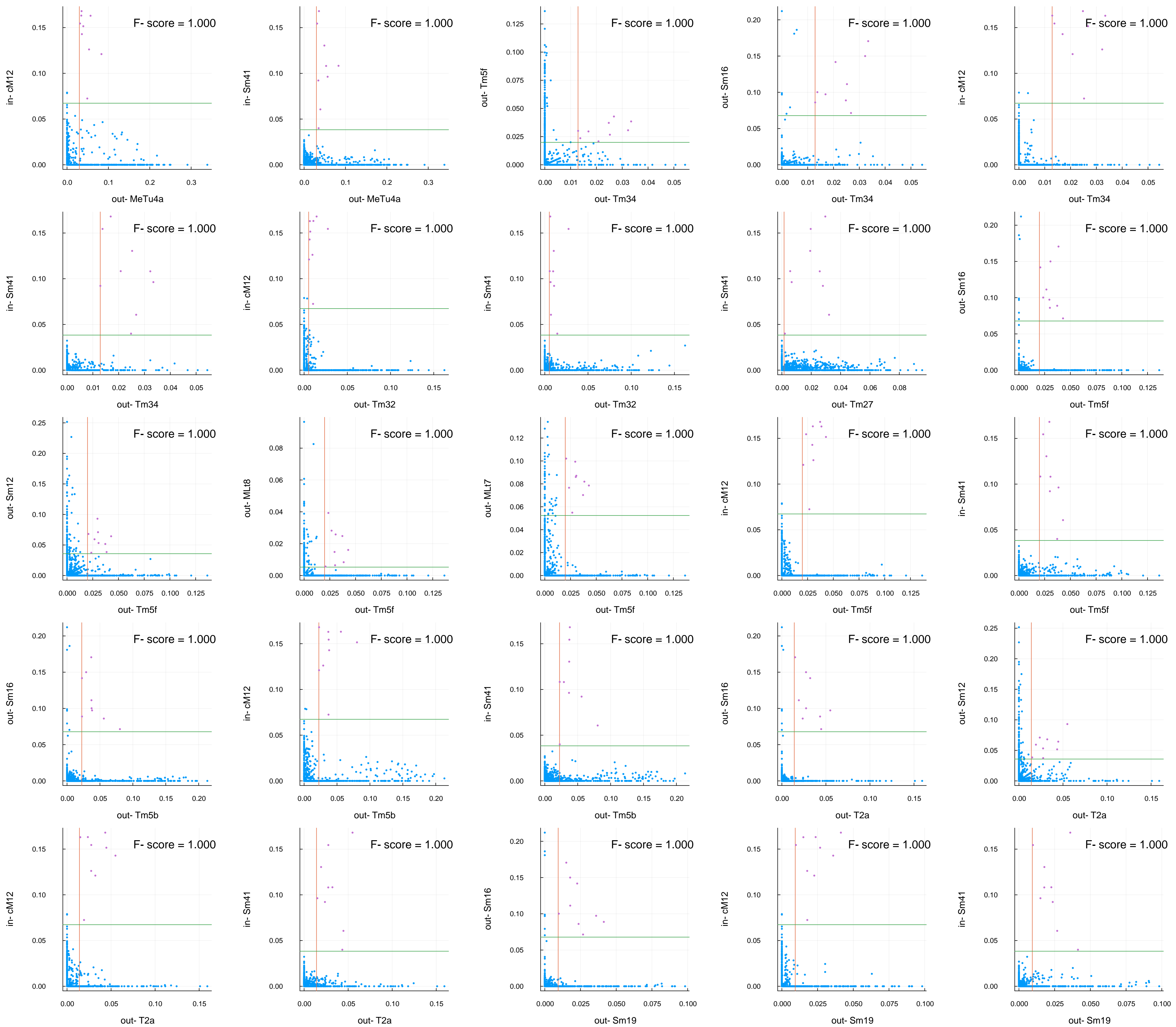

Supplement: Supplementary file 7 — Discriminating 2D projections for neuropil-intrinsic types. For each interneuron type, a pair of features is shown that can be used to discriminate that type from others in the same neuropil. Many although not all discriminations are highly accurate. Both intrinsic and boundary types are included as discriminative features. [file 41586_2024_7981_MOESM7_ESM.zip › DataS3/Sm25.pdf]

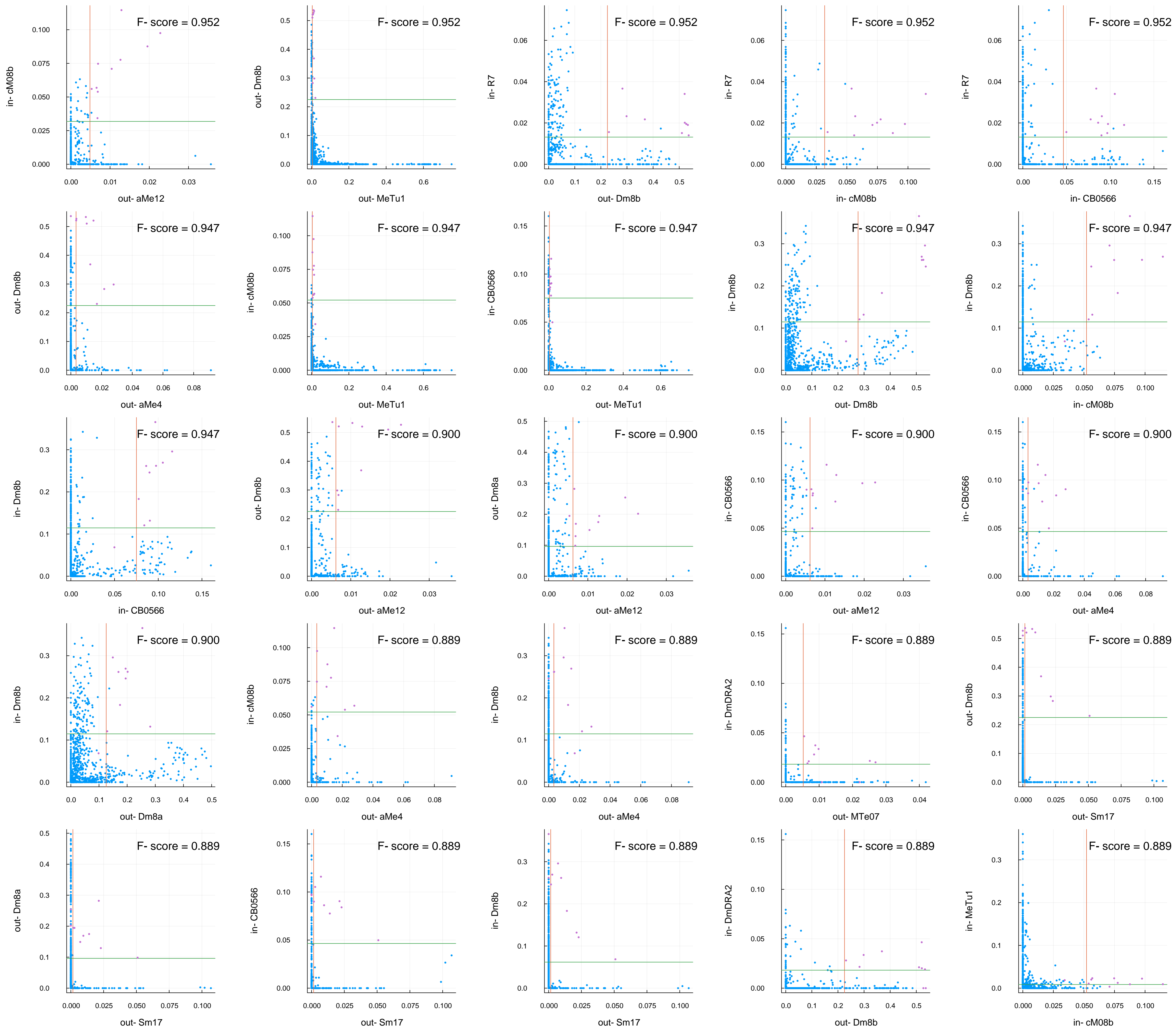

Supplement: Supplementary file 7 — Discriminating 2D projections for neuropil-intrinsic types. For each interneuron type, a pair of features is shown that can be used to discriminate that type from others in the same neuropil. Many although not all discriminations are highly accurate. Both intrinsic and boundary types are included as discriminative features. [file 41586_2024_7981_MOESM7_ESM.zip › DataS3/Sm26.pdf]

## Sm27

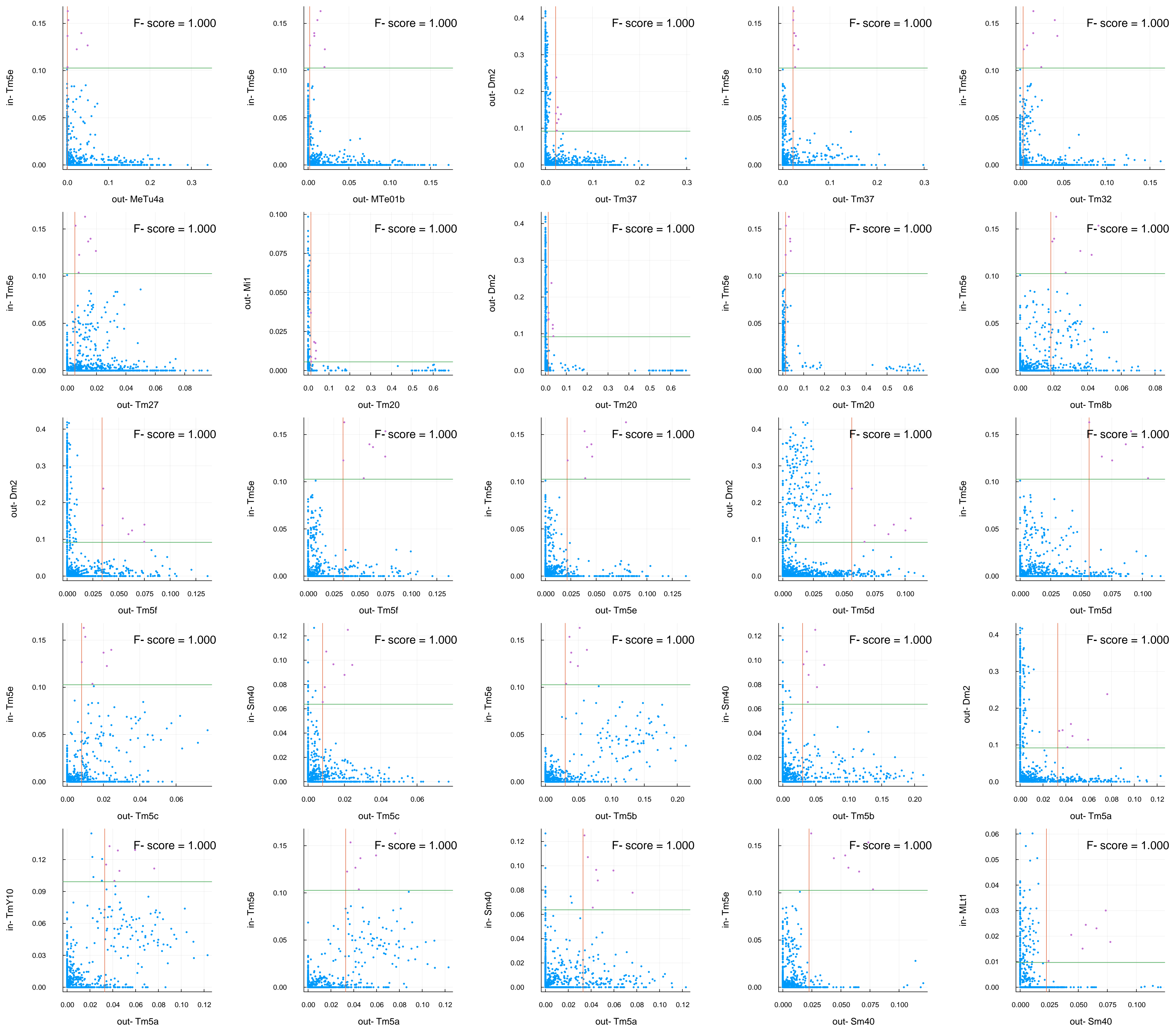

Supplement: Supplementary file 7 — Discriminating 2D projections for neuropil-intrinsic types. For each interneuron type, a pair of features is shown that can be used to discriminate that type from others in the same neuropil. Many although not all discriminations are highly accurate. Both intrinsic and boundary types are included as discriminative features. [file 41586_2024_7981_MOESM7_ESM.zip › DataS3/Sm27.pdf]

Sm28

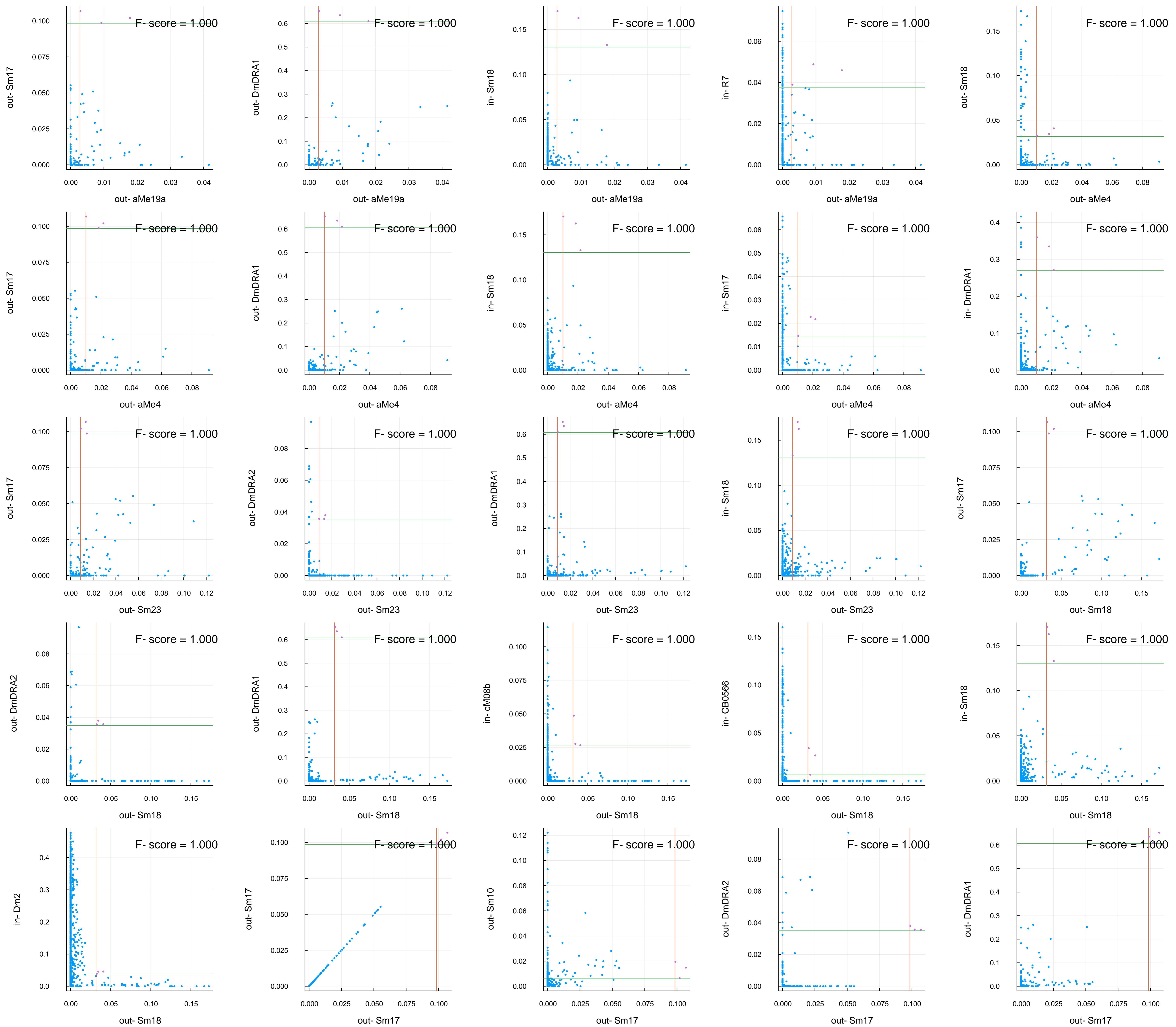

Supplement: Supplementary file 7 — Discriminating 2D projections for neuropil-intrinsic types. For each interneuron type, a pair of features is shown that can be used to discriminate that type from others in the same neuropil. Many although not all discriminations are highly accurate. Both intrinsic and boundary types are included as discriminative features. [file 41586_2024_7981_MOESM7_ESM.zip › DataS3/Sm28.pdf]

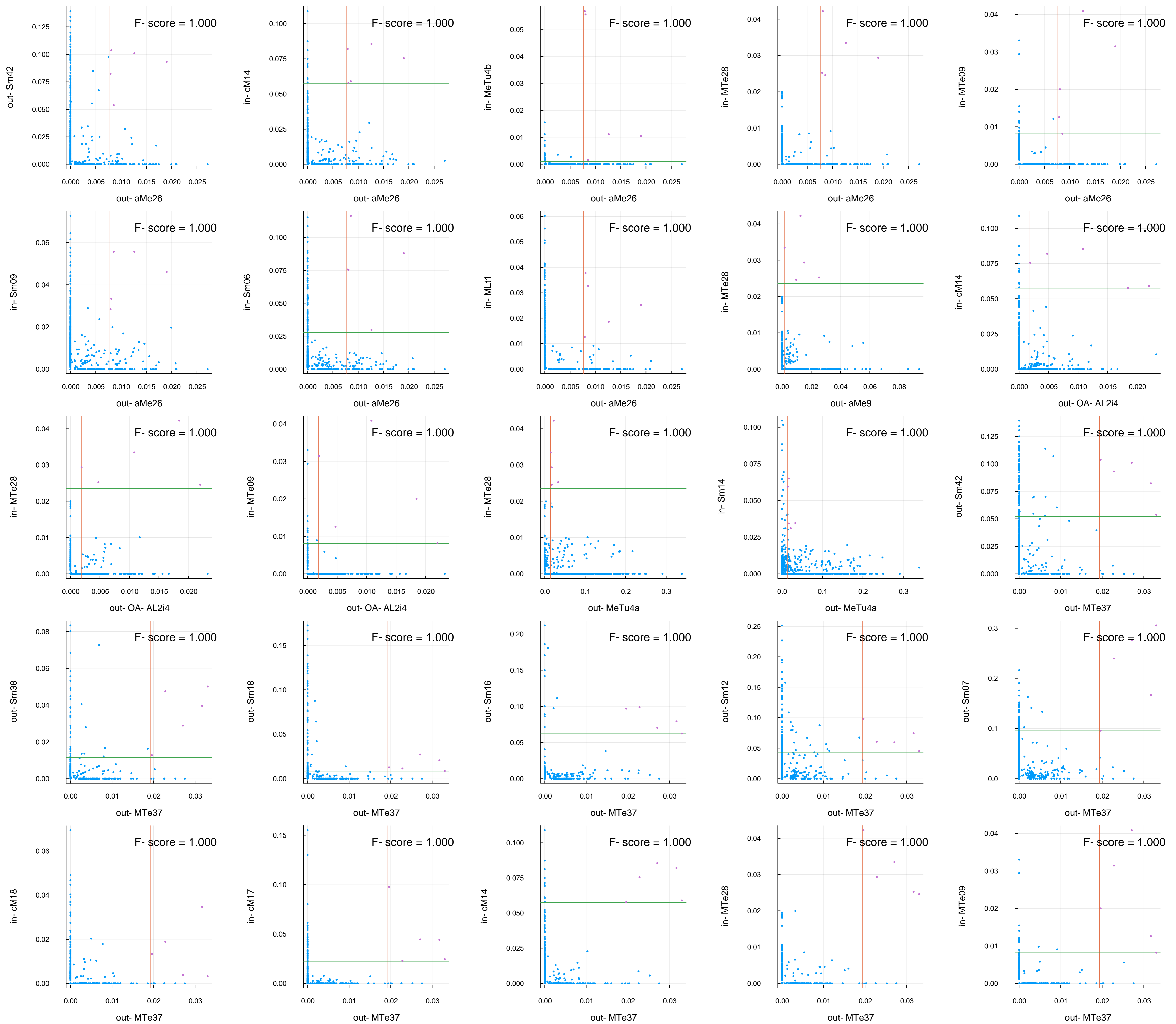

Supplement: Supplementary file 7 — Discriminating 2D projections for neuropil-intrinsic types. For each interneuron type, a pair of features is shown that can be used to discriminate that type from others in the same neuropil. Many although not all discriminations are highly accurate. Both intrinsic and boundary types are included as discriminative features. [file 41586_2024_7981_MOESM7_ESM.zip › DataS3/Sm29.pdf]

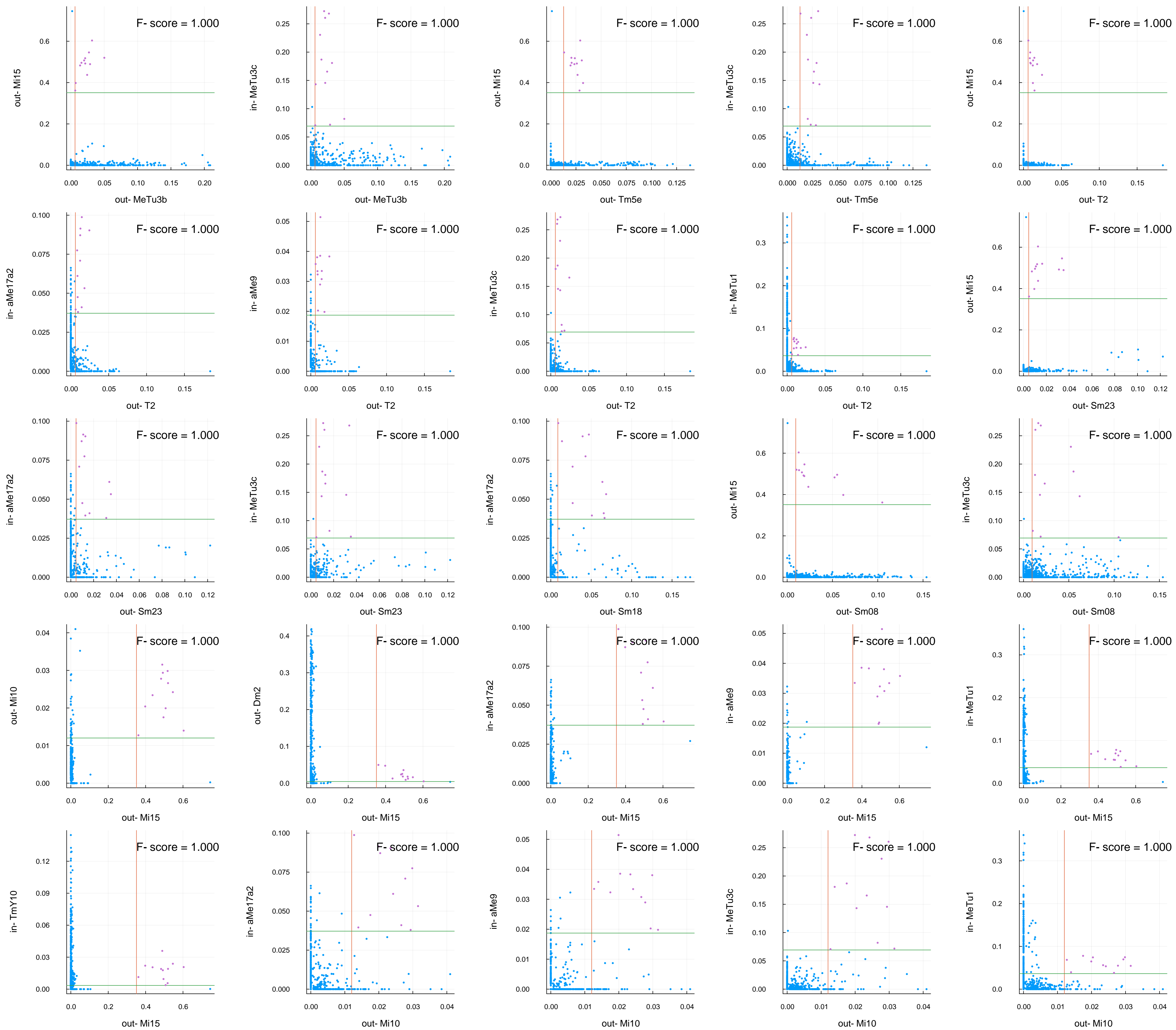

Supplement: Supplementary file 7 — Discriminating 2D projections for neuropil-intrinsic types. For each interneuron type, a pair of features is shown that can be used to discriminate that type from others in the same neuropil. Many although not all discriminations are highly accurate. Both intrinsic and boundary types are included as discriminative features. [file 41586_2024_7981_MOESM7_ESM.zip › DataS3/Sm30.pdf]

## Sm31

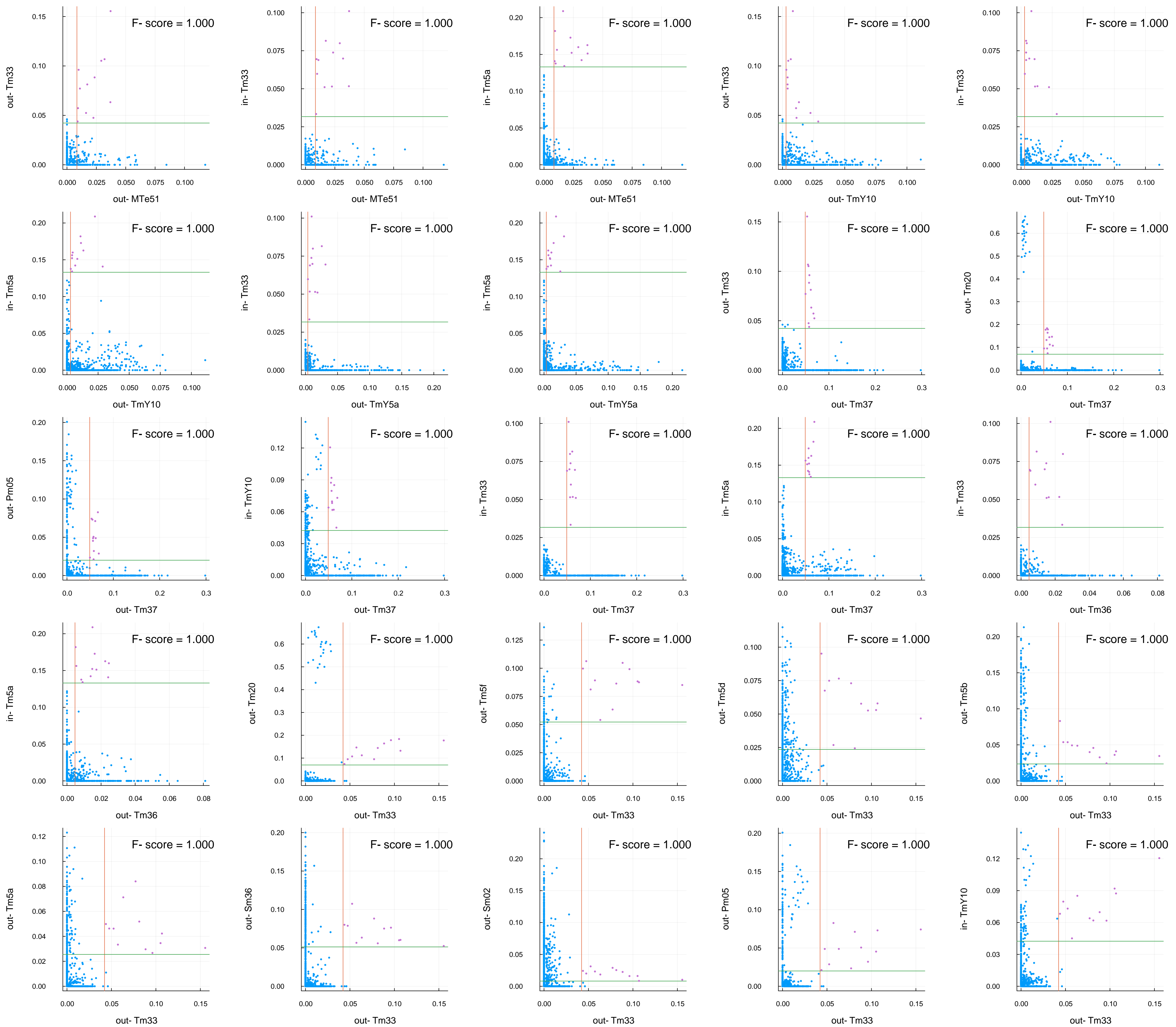

Supplement: Supplementary file 7 — Discriminating 2D projections for neuropil-intrinsic types. For each interneuron type, a pair of features is shown that can be used to discriminate that type from others in the same neuropil. Many although not all discriminations are highly accurate. Both intrinsic and boundary types are included as discriminative features. [file 41586_2024_7981_MOESM7_ESM.zip › DataS3/Sm31.pdf]

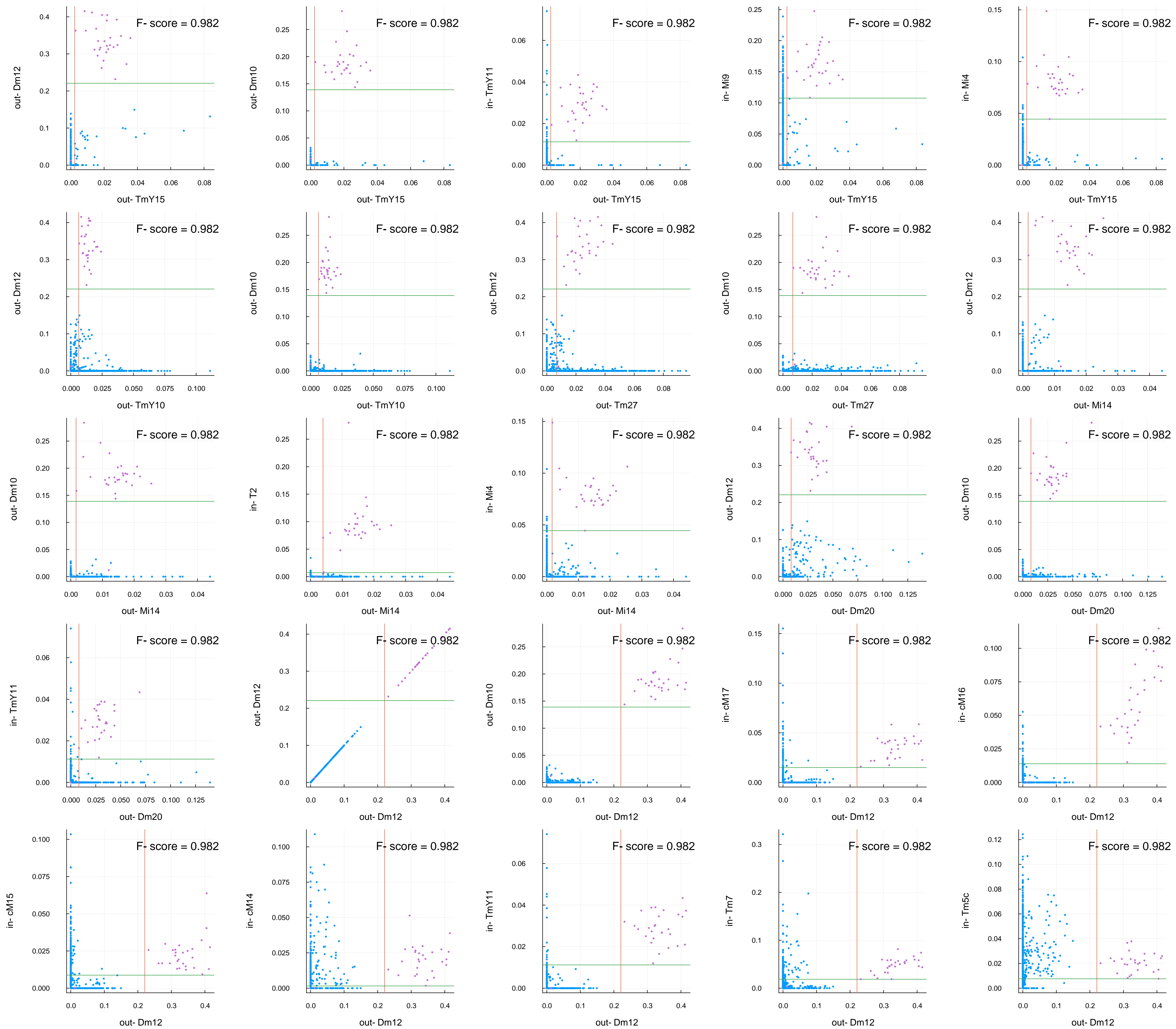

Supplement: Supplementary file 7 — Discriminating 2D projections for neuropil-intrinsic types. For each interneuron type, a pair of features is shown that can be used to discriminate that type from others in the same neuropil. Many although not all discriminations are highly accurate. Both intrinsic and boundary types are included as discriminative features. [file 41586_2024_7981_MOESM7_ESM.zip › DataS3/Sm32.pdf]

Sm33

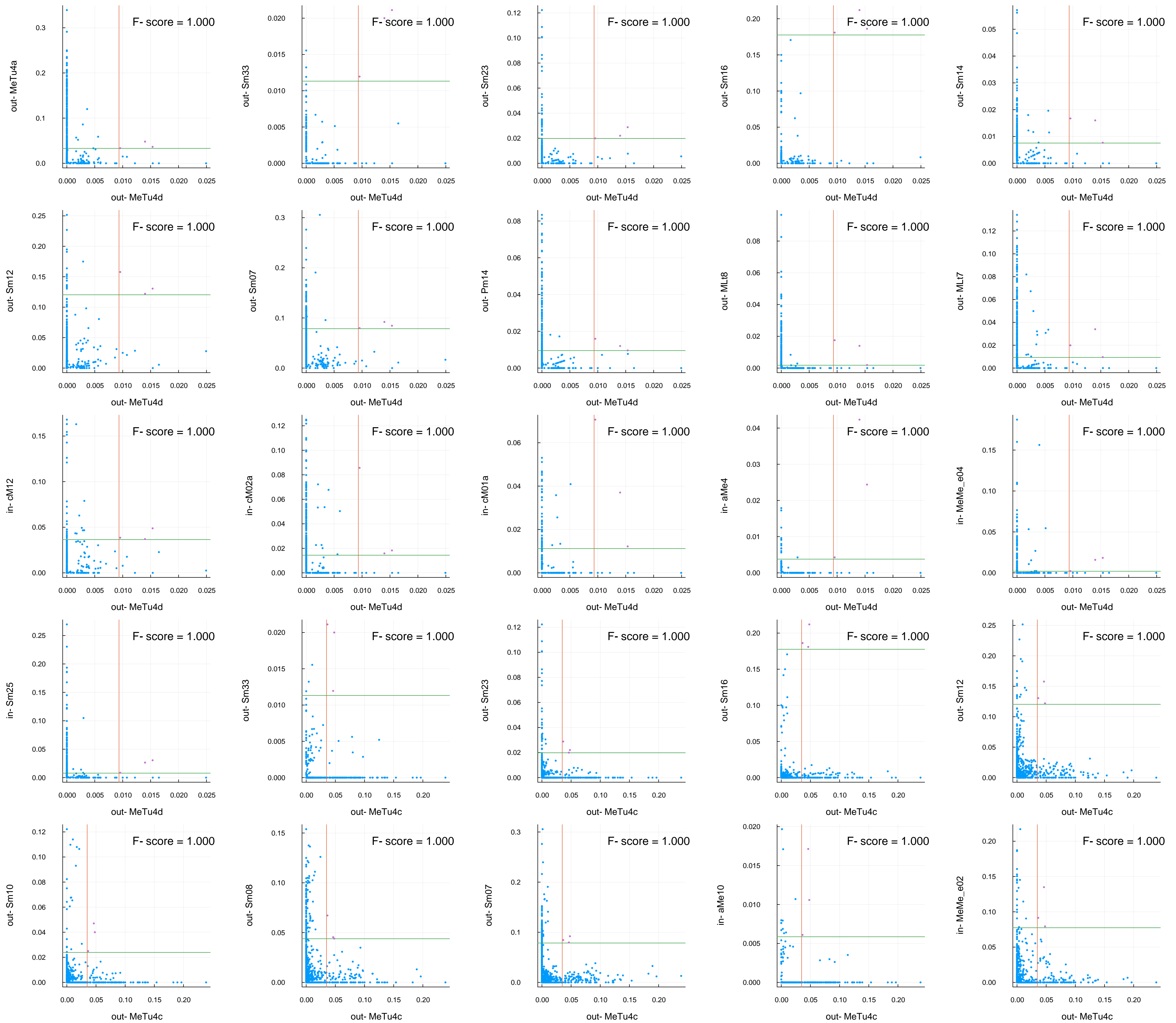

Supplement: Supplementary file 7 — Discriminating 2D projections for neuropil-intrinsic types. For each interneuron type, a pair of features is shown that can be used to discriminate that type from others in the same neuropil. Many although not all discriminations are highly accurate. Both intrinsic and boundary types are included as discriminative features. [file 41586_2024_7981_MOESM7_ESM.zip › DataS3/Sm33.pdf]

Sm34

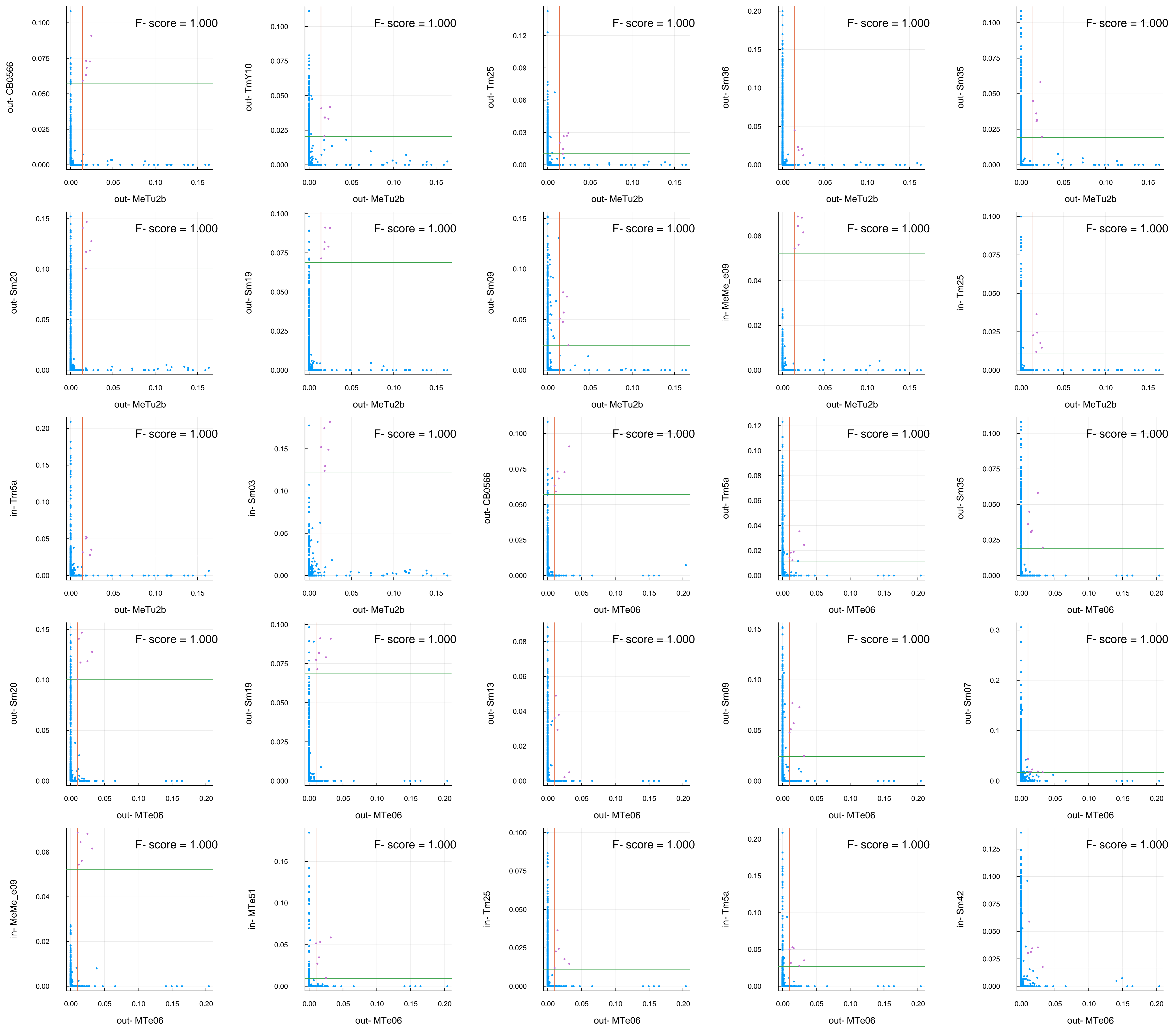

Supplement: Supplementary file 7 — Discriminating 2D projections for neuropil-intrinsic types. For each interneuron type, a pair of features is shown that can be used to discriminate that type from others in the same neuropil. Many although not all discriminations are highly accurate. Both intrinsic and boundary types are included as discriminative features. [file 41586_2024_7981_MOESM7_ESM.zip › DataS3/Sm34.pdf]

Sm35

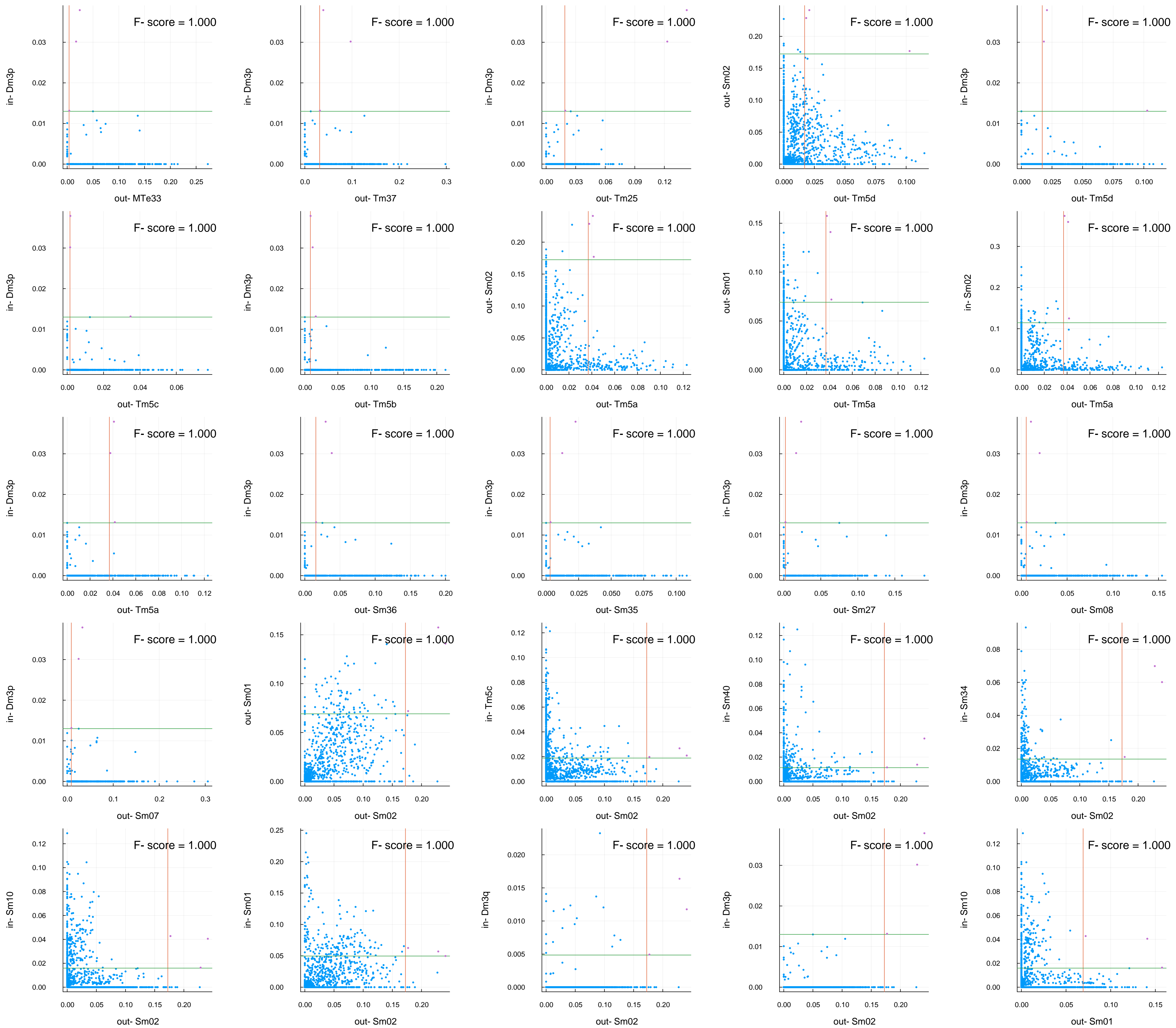

Supplement: Supplementary file 7 — Discriminating 2D projections for neuropil-intrinsic types. For each interneuron type, a pair of features is shown that can be used to discriminate that type from others in the same neuropil. Many although not all discriminations are highly accurate. Both intrinsic and boundary types are included as discriminative features. [file 41586_2024_7981_MOESM7_ESM.zip › DataS3/Sm35.pdf]

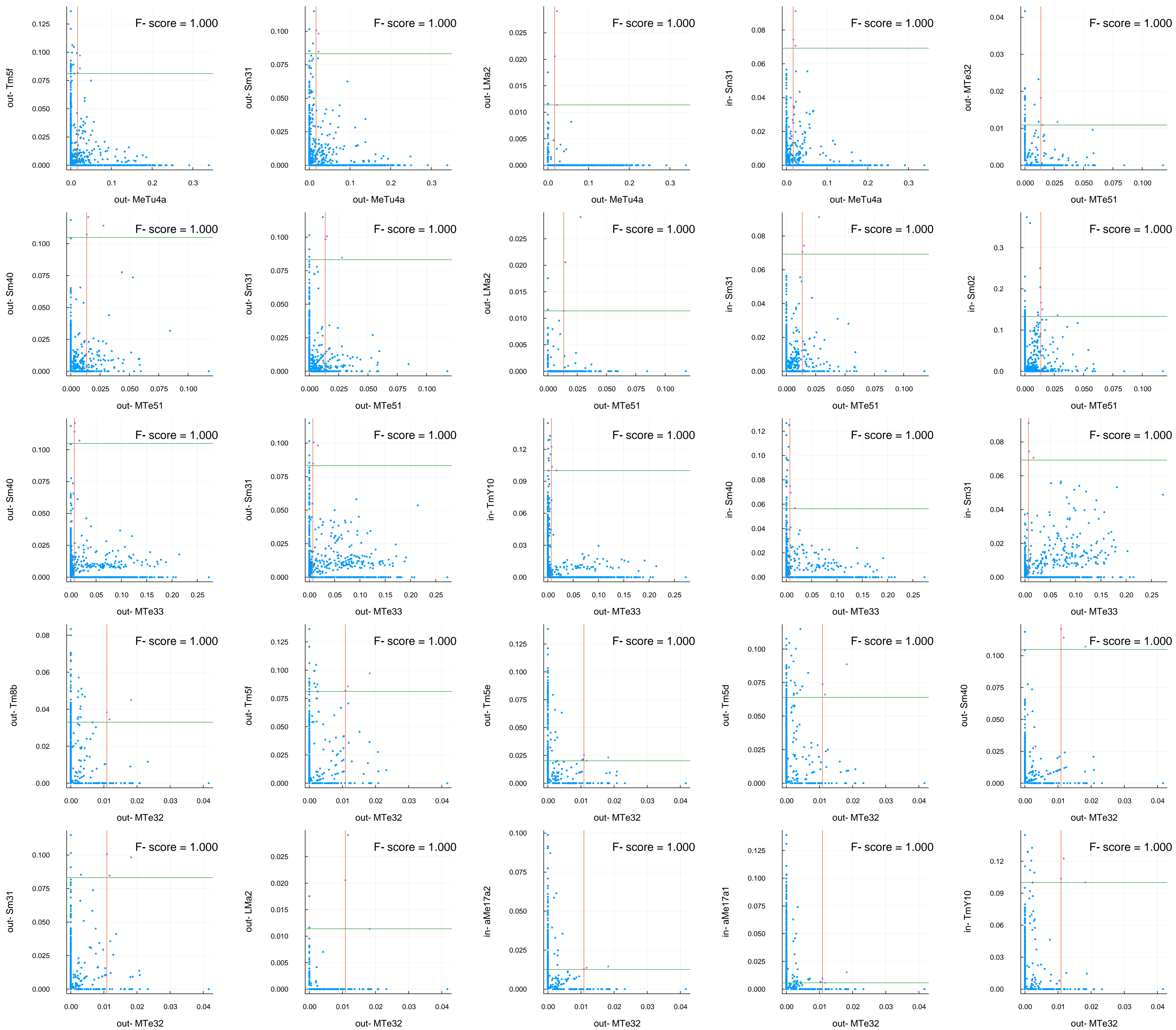

Supplement: Supplementary file 7 — Discriminating 2D projections for neuropil-intrinsic types. For each interneuron type, a pair of features is shown that can be used to discriminate that type from others in the same neuropil. Many although not all discriminations are highly accurate. Both intrinsic and boundary types are included as discriminative features. [file 41586_2024_7981_MOESM7_ESM.zip › DataS3/Sm36.pdf]

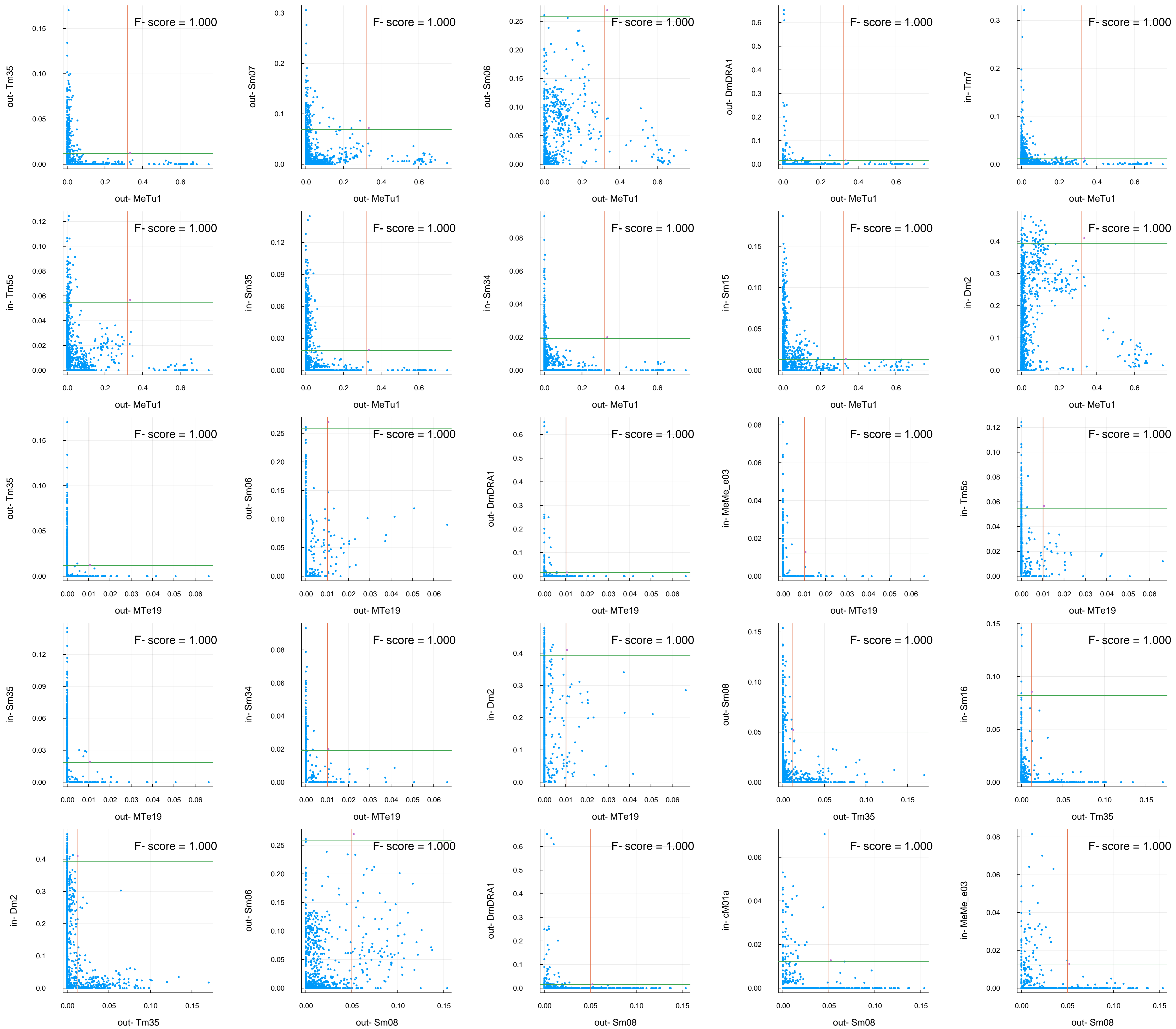

Supplement: Supplementary file 7 — Discriminating 2D projections for neuropil-intrinsic types. For each interneuron type, a pair of features is shown that can be used to discriminate that type from others in the same neuropil. Many although not all discriminations are highly accurate. Both intrinsic and boundary types are included as discriminative features. [file 41586_2024_7981_MOESM7_ESM.zip › DataS3/Sm37.pdf]

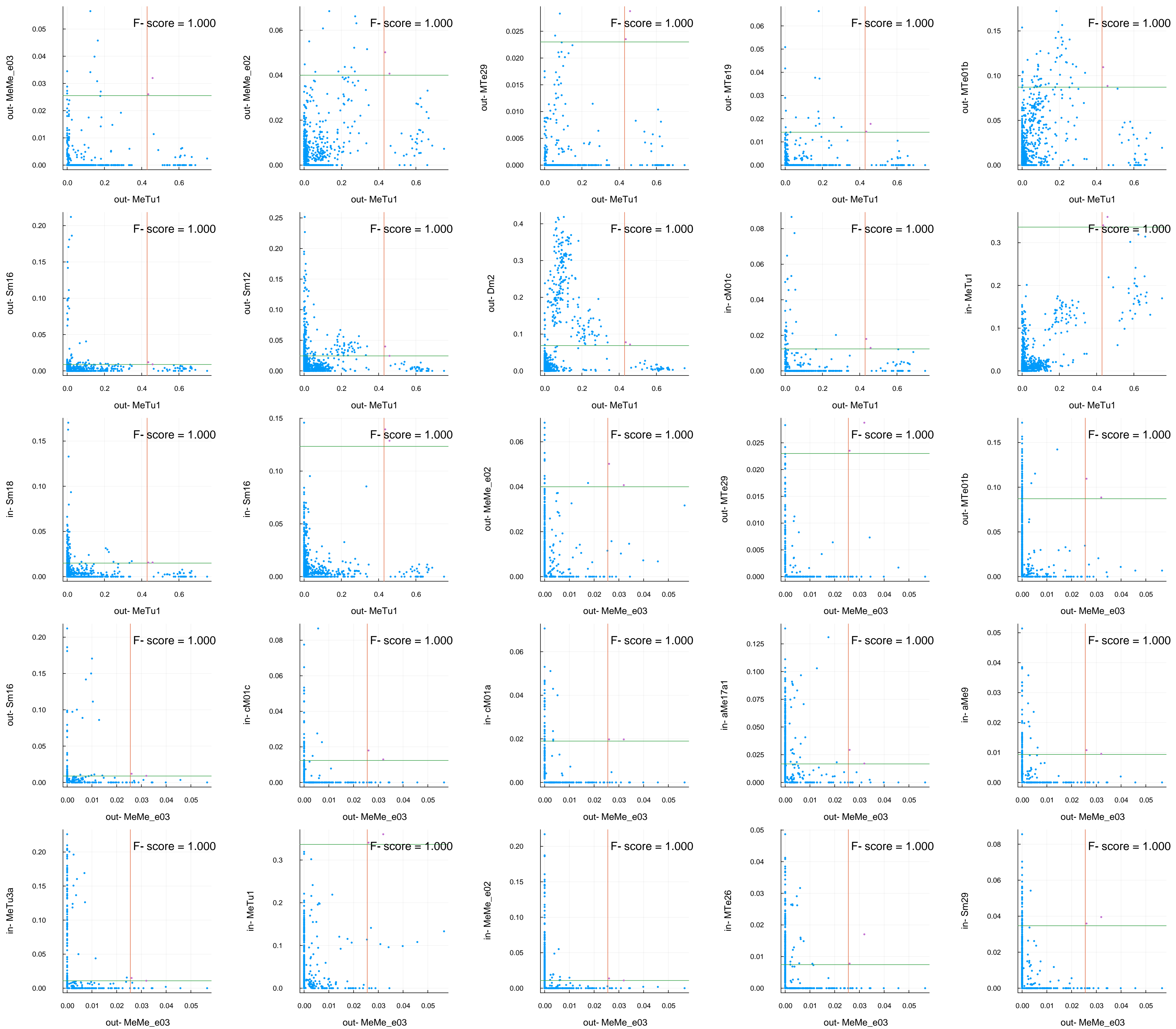

Supplement: Supplementary file 7 — Discriminating 2D projections for neuropil-intrinsic types. For each interneuron type, a pair of features is shown that can be used to discriminate that type from others in the same neuropil. Many although not all discriminations are highly accurate. Both intrinsic and boundary types are included as discriminative features. [file 41586_2024_7981_MOESM7_ESM.zip › DataS3/Sm38.pdf]

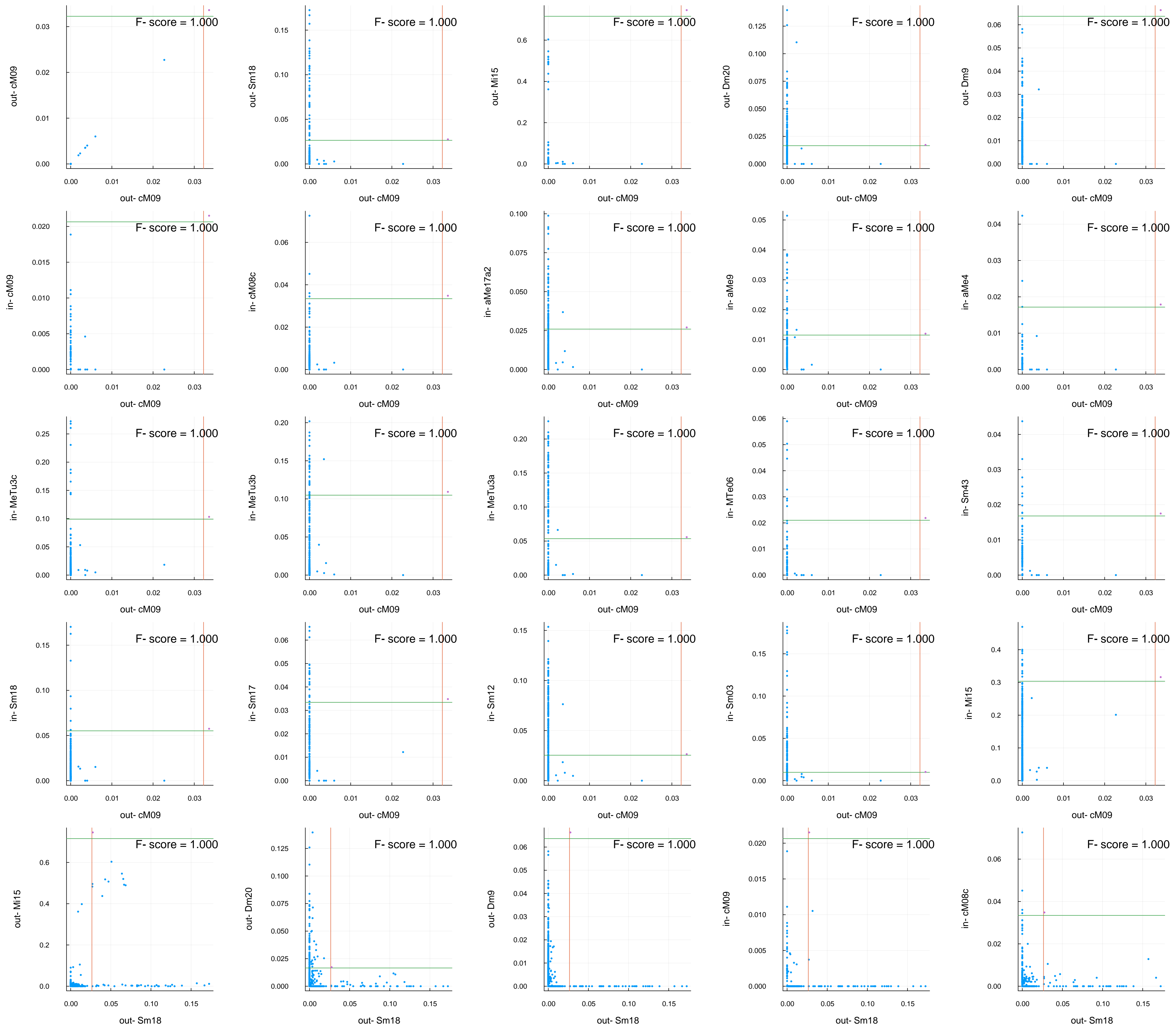

Supplement: Supplementary file 7 — Discriminating 2D projections for neuropil-intrinsic types. For each interneuron type, a pair of features is shown that can be used to discriminate that type from others in the same neuropil. Many although not all discriminations are highly accurate. Both intrinsic and boundary types are included as discriminative features. [file 41586_2024_7981_MOESM7_ESM.zip › DataS3/Sm39.pdf]

## Sm40

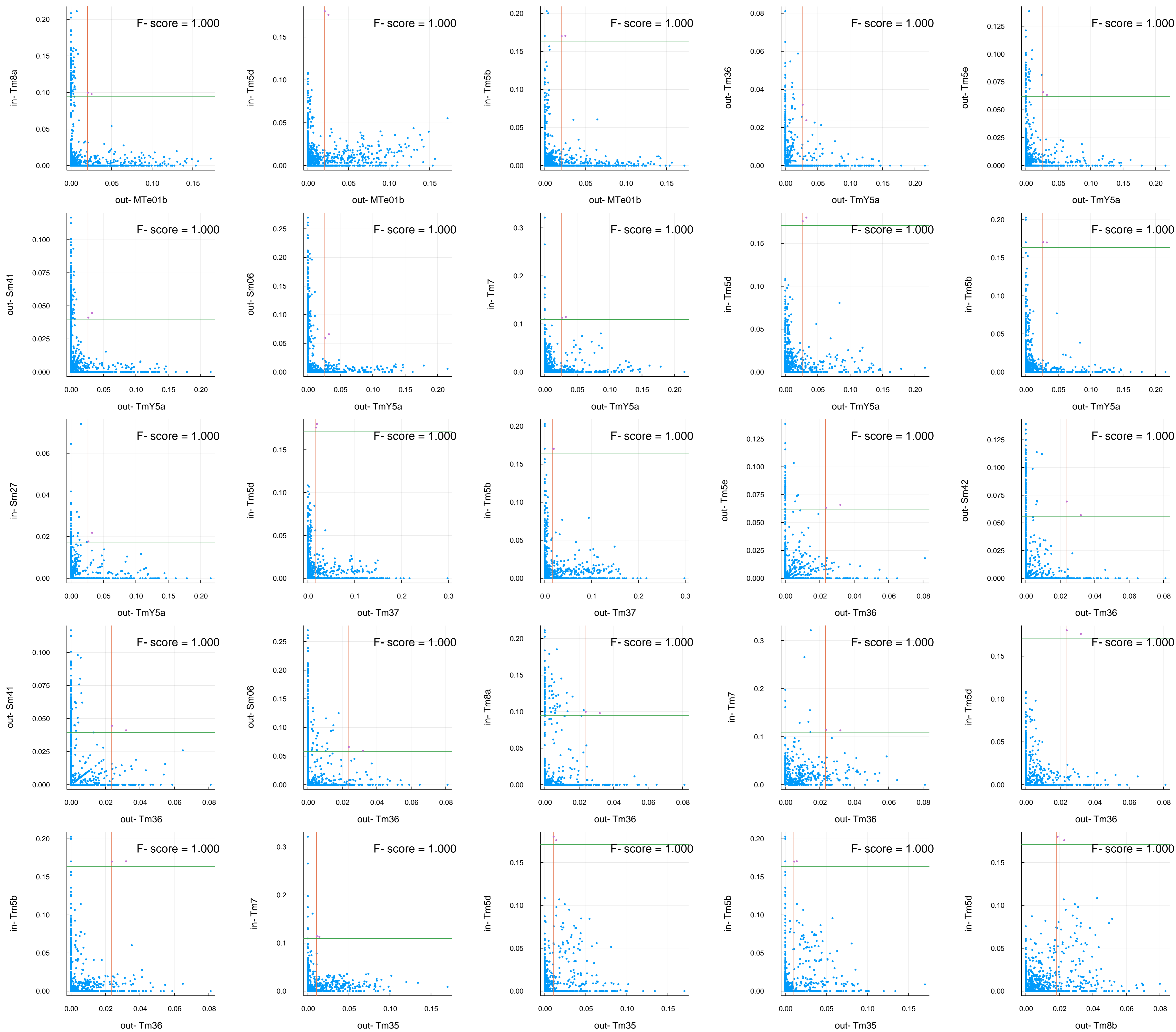

Supplement: Supplementary file 7 — Discriminating 2D projections for neuropil-intrinsic types. For each interneuron type, a pair of features is shown that can be used to discriminate that type from others in the same neuropil. Many although not all discriminations are highly accurate. Both intrinsic and boundary types are included as discriminative features. [file 41586_2024_7981_MOESM7_ESM.zip › DataS3/Sm40.pdf]

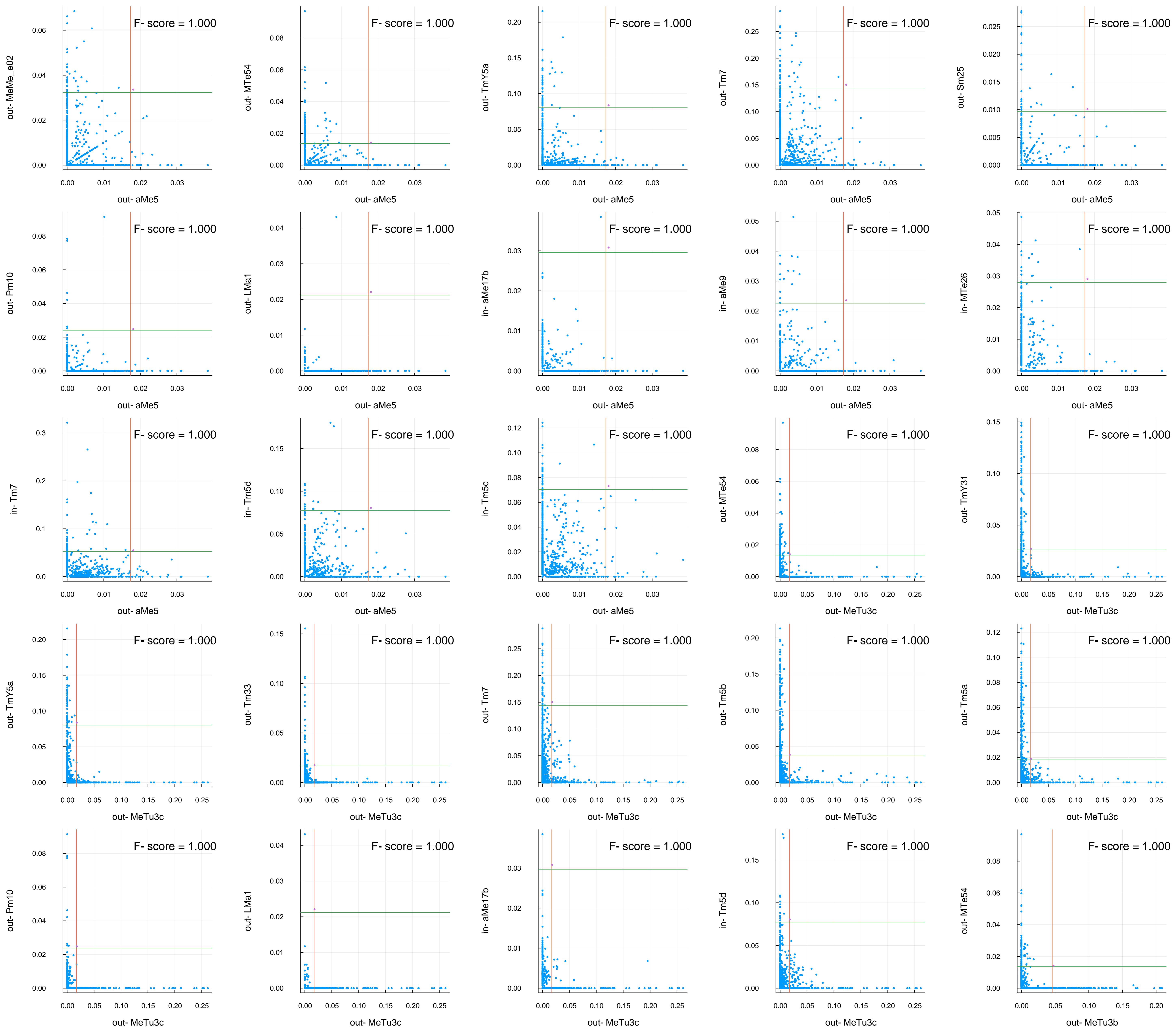

Supplement: Supplementary file 7 — Discriminating 2D projections for neuropil-intrinsic types. For each interneuron type, a pair of features is shown that can be used to discriminate that type from others in the same neuropil. Many although not all discriminations are highly accurate. Both intrinsic and boundary types are included as discriminative features. [file 41586_2024_7981_MOESM7_ESM.zip › DataS3/Sm41.pdf]

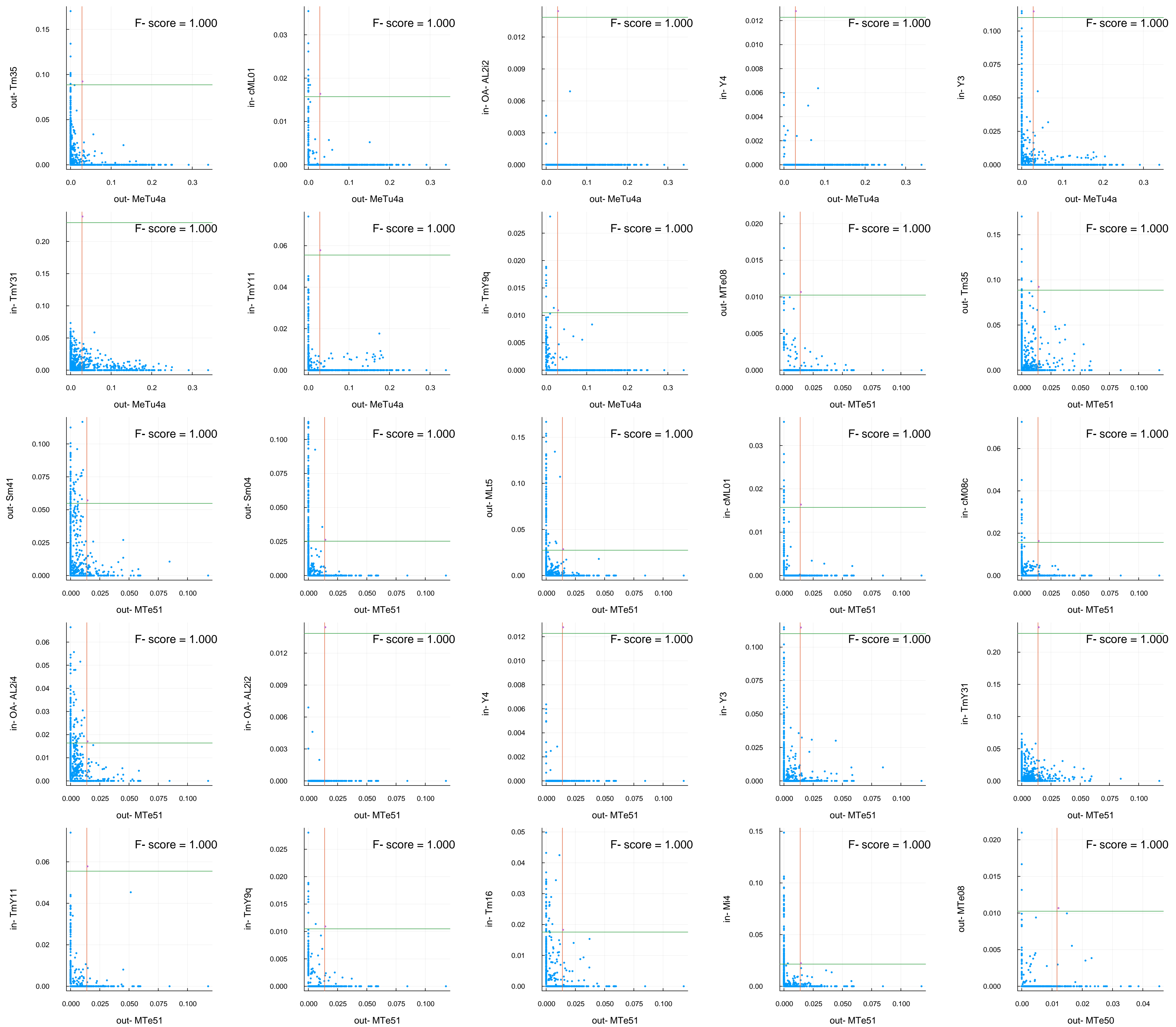

Supplement: Supplementary file 7 — Discriminating 2D projections for neuropil-intrinsic types. For each interneuron type, a pair of features is shown that can be used to discriminate that type from others in the same neuropil. Many although not all discriminations are highly accurate. Both intrinsic and boundary types are included as discriminative features. [file 41586_2024_7981_MOESM7_ESM.zip › DataS3/Sm42.pdf]

Sm43

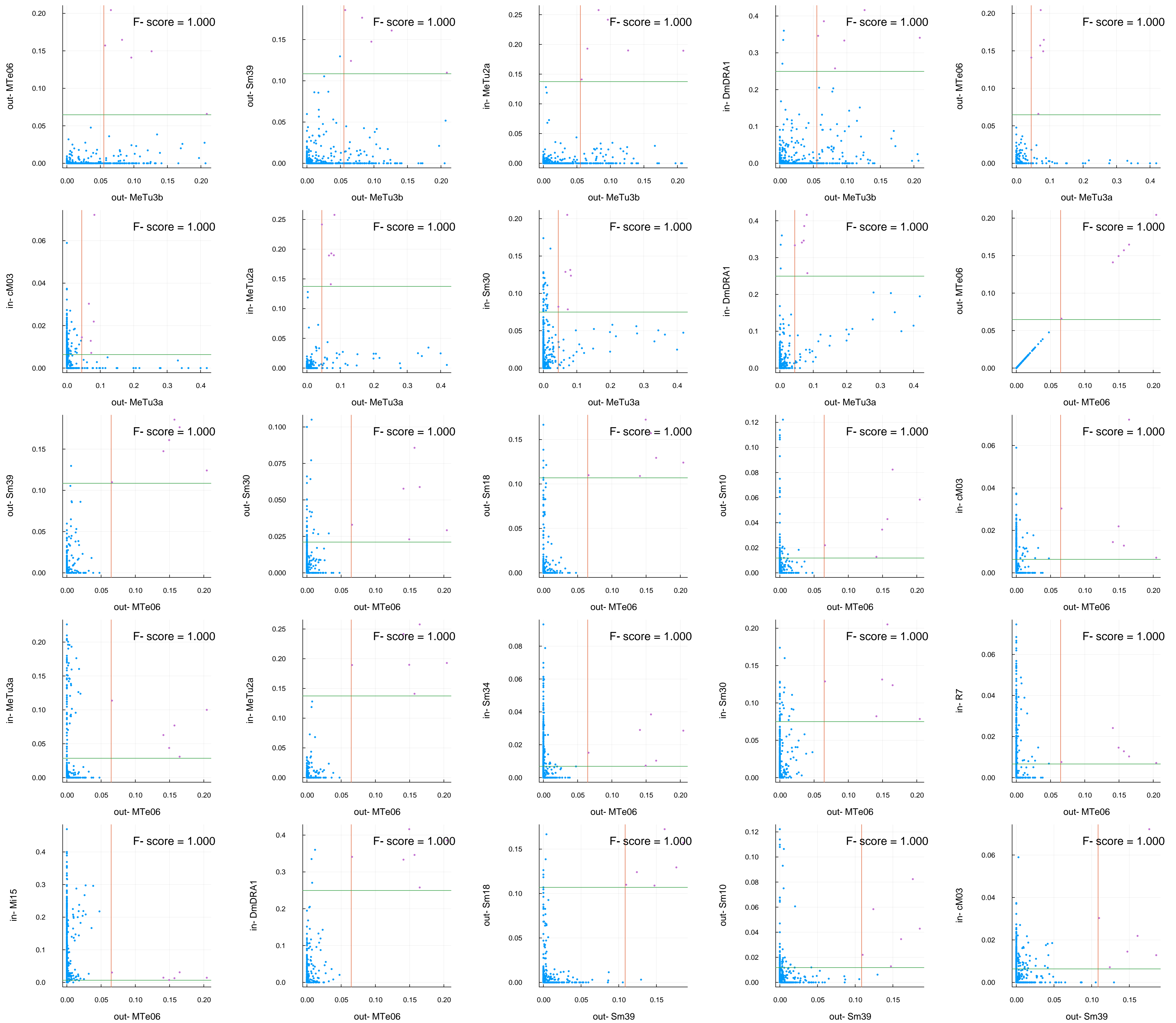

Supplement: Supplementary file 7 — Discriminating 2D projections for neuropil-intrinsic types. For each interneuron type, a pair of features is shown that can be used to discriminate that type from others in the same neuropil. Many although not all discriminations are highly accurate. Both intrinsic and boundary types are included as discriminative features. [file 41586_2024_7981_MOESM7_ESM.zip › DataS3/Sm43.pdf]

T2

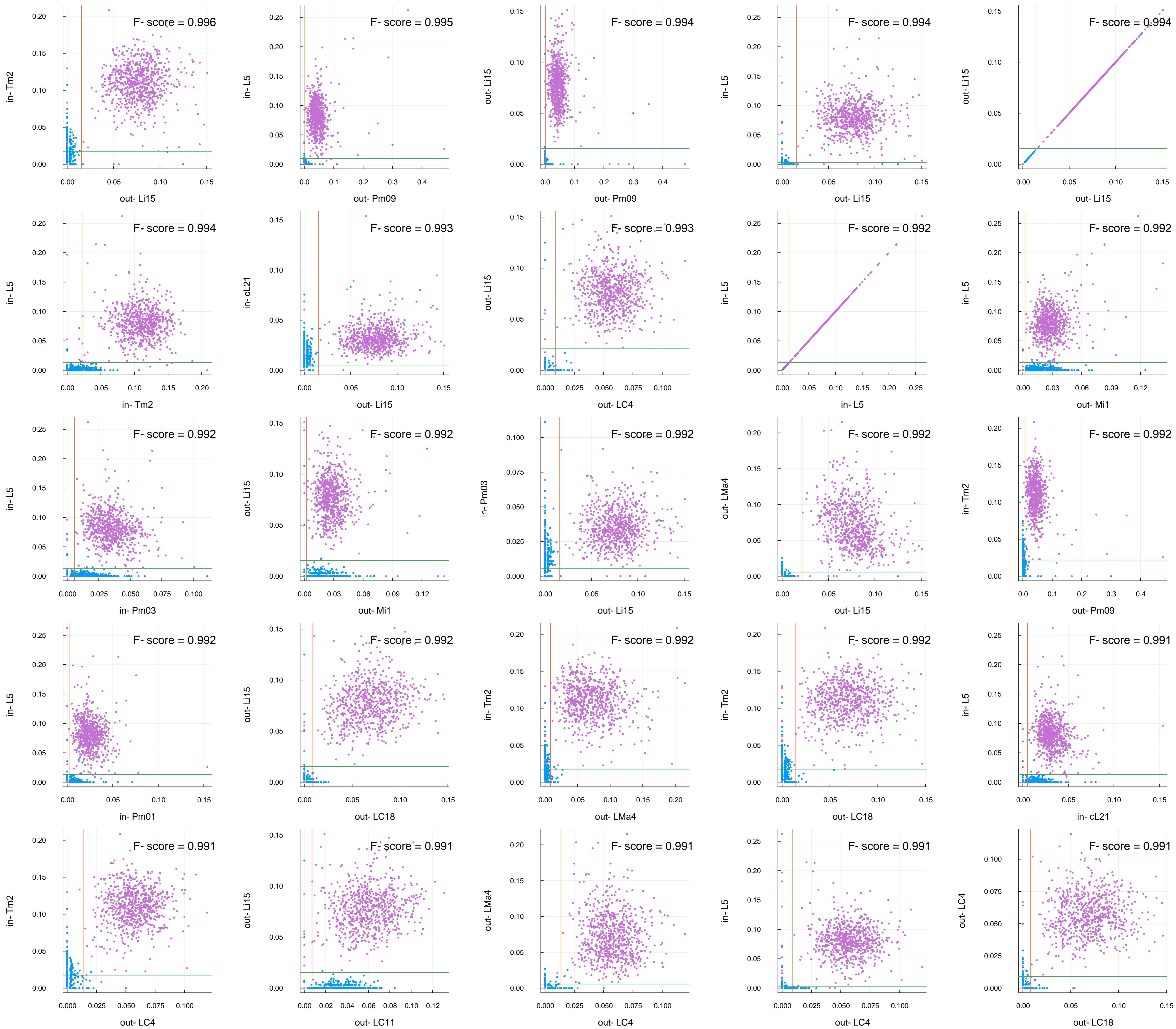

Supplement: Supplementary file 7 — Discriminating 2D projections for neuropil-intrinsic types. For each interneuron type, a pair of features is shown that can be used to discriminate that type from others in the same neuropil. Many although not all discriminations are highly accurate. Both intrinsic and boundary types are included as discriminative features. [file 41586_2024_7981_MOESM7_ESM.zip › DataS3/T2.pdf]

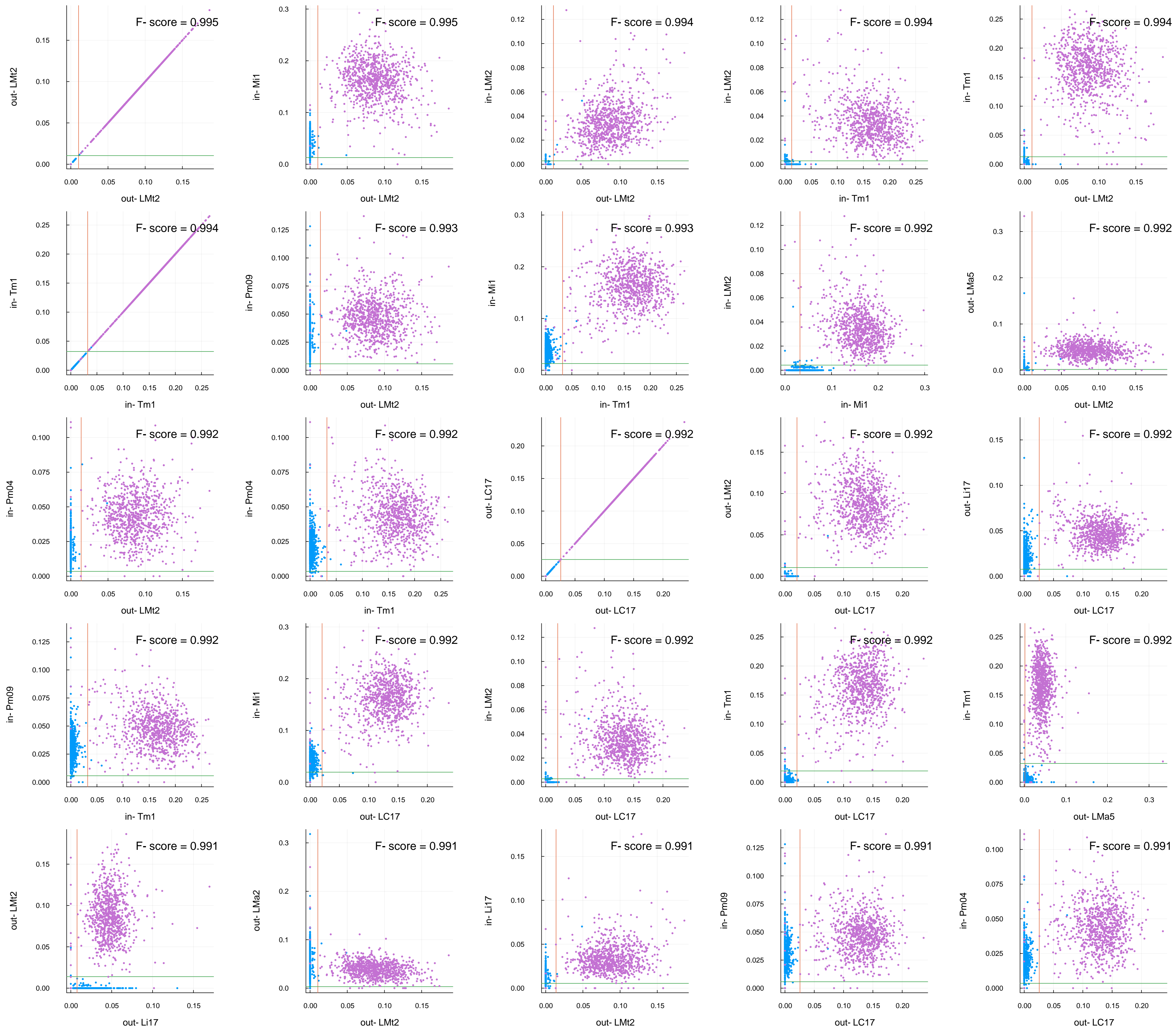

Supplement: Supplementary file 7 — Discriminating 2D projections for neuropil-intrinsic types. For each interneuron type, a pair of features is shown that can be used to discriminate that type from others in the same neuropil. Many although not all discriminations are highly accurate. Both intrinsic and boundary types are included as discriminative features. [file 41586_2024_7981_MOESM7_ESM.zip › DataS3/T2a.pdf]

T4a

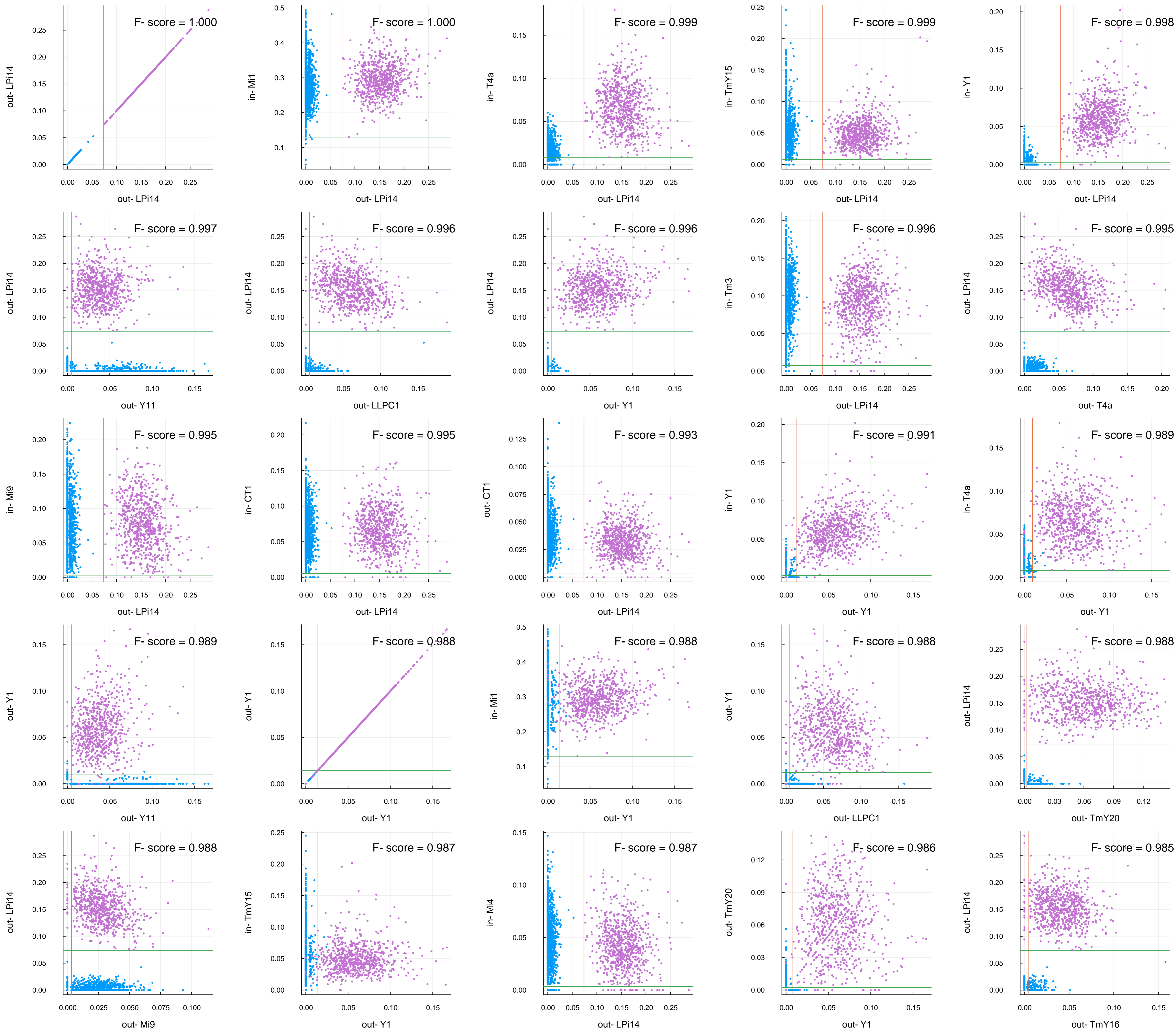

Supplement: Supplementary file 7 — Discriminating 2D projections for neuropil-intrinsic types. For each interneuron type, a pair of features is shown that can be used to discriminate that type from others in the same neuropil. Many although not all discriminations are highly accurate. Both intrinsic and boundary types are included as discriminative features. [file 41586_2024_7981_MOESM7_ESM.zip › DataS3/T4a.pdf]

T4b

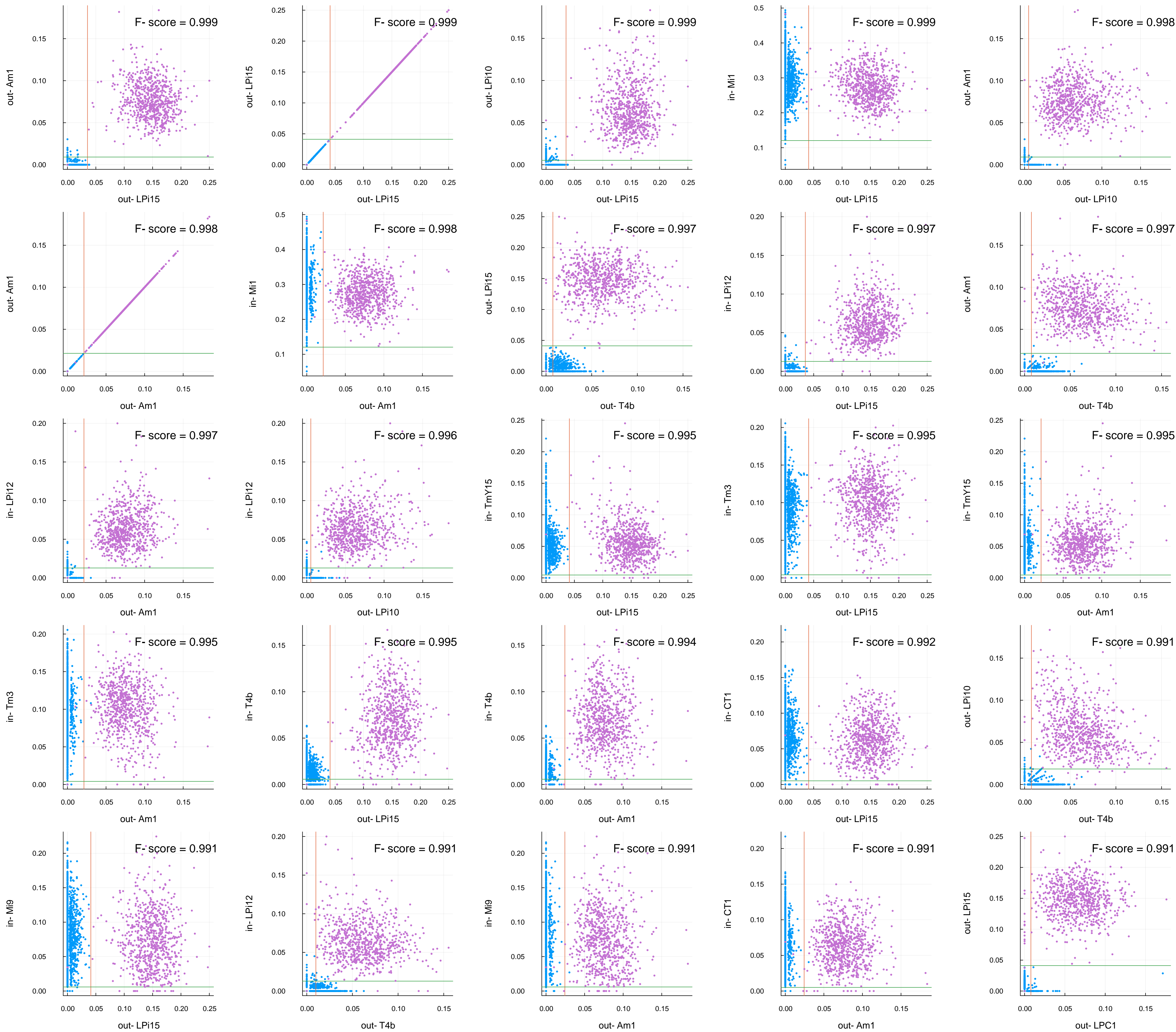

Supplement: Supplementary file 7 — Discriminating 2D projections for neuropil-intrinsic types. For each interneuron type, a pair of features is shown that can be used to discriminate that type from others in the same neuropil. Many although not all discriminations are highly accurate. Both intrinsic and boundary types are included as discriminative features. [file 41586_2024_7981_MOESM7_ESM.zip › DataS3/T4b.pdf]

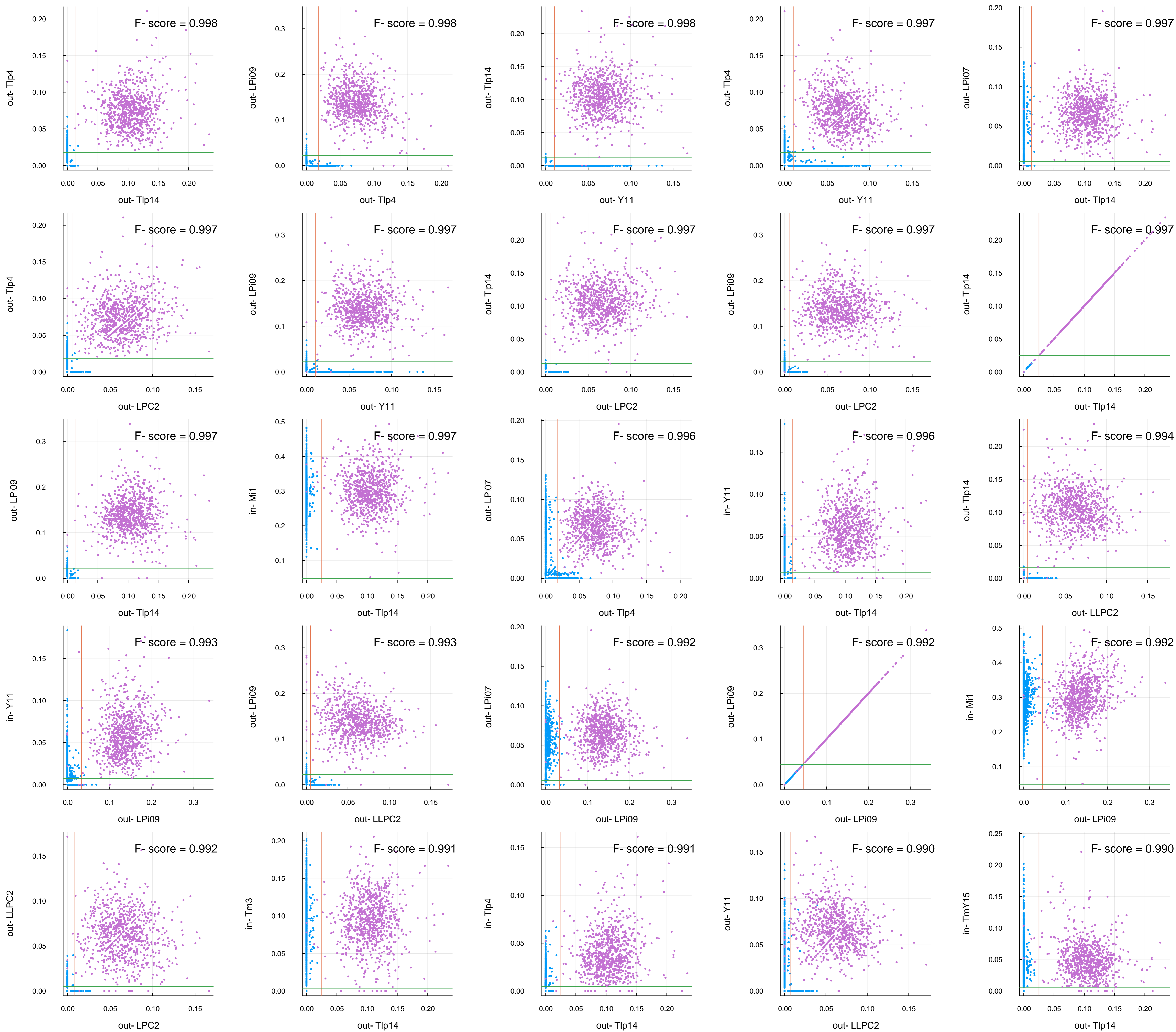

Supplement: Supplementary file 7 — Discriminating 2D projections for neuropil-intrinsic types. For each interneuron type, a pair of features is shown that can be used to discriminate that type from others in the same neuropil. Many although not all discriminations are highly accurate. Both intrinsic and boundary types are included as discriminative features. [file 41586_2024_7981_MOESM7_ESM.zip › DataS3/T4c.pdf]

T4d

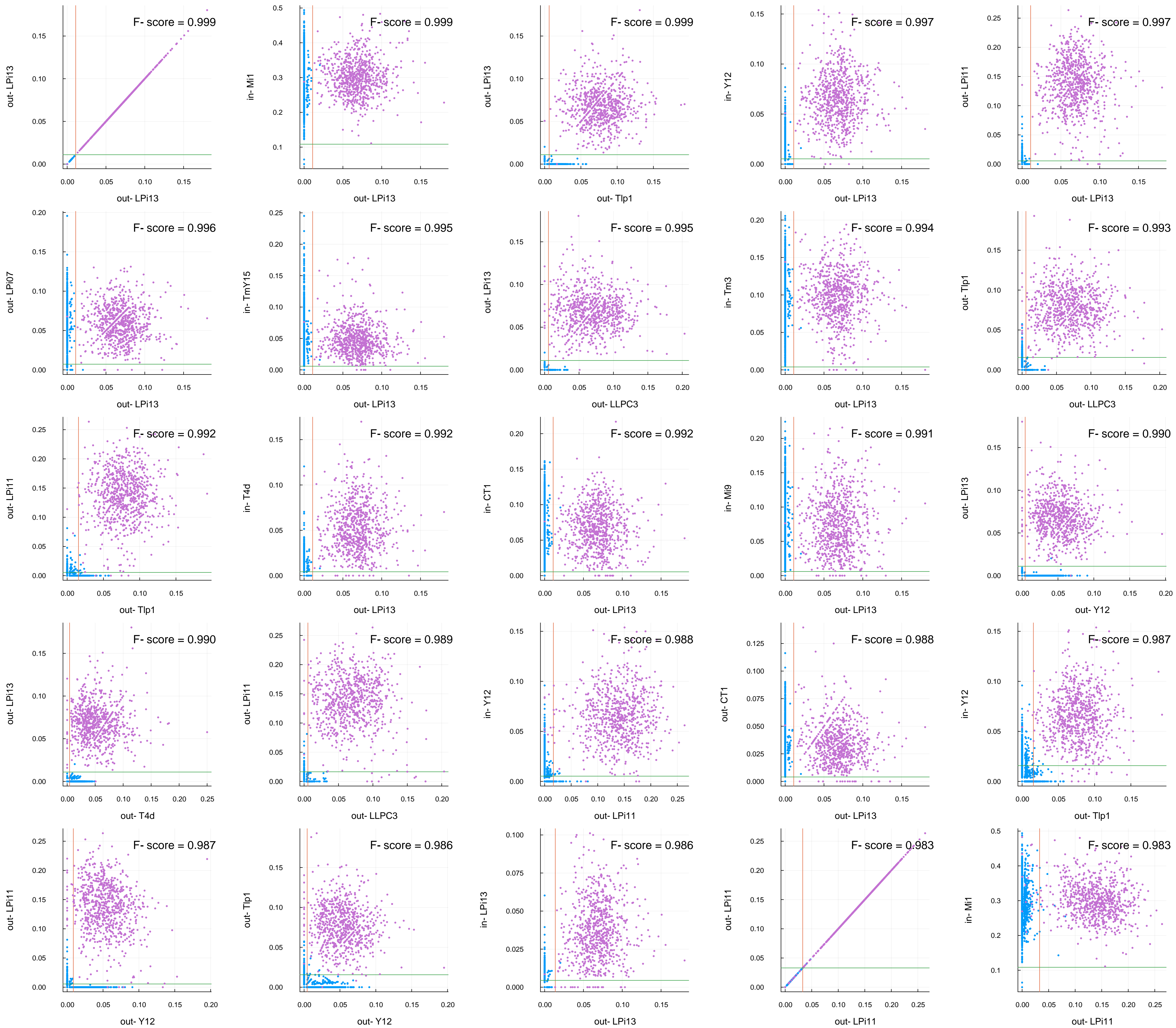

Supplement: Supplementary file 7 — Discriminating 2D projections for neuropil-intrinsic types. For each interneuron type, a pair of features is shown that can be used to discriminate that type from others in the same neuropil. Many although not all discriminations are highly accurate. Both intrinsic and boundary types are included as discriminative features. [file 41586_2024_7981_MOESM7_ESM.zip › DataS3/T4d.pdf]

T5a

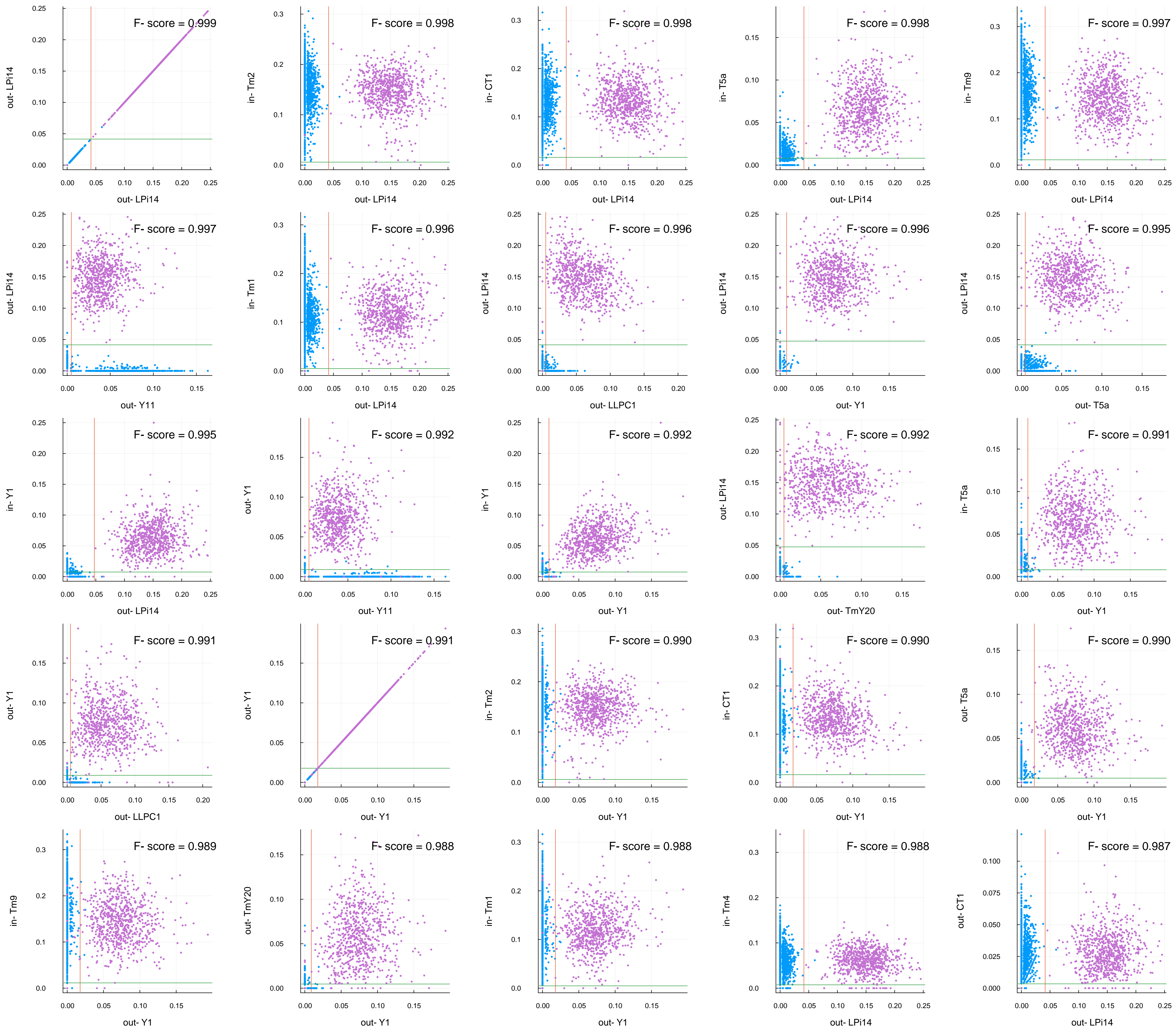

Supplement: Supplementary file 7 — Discriminating 2D projections for neuropil-intrinsic types. For each interneuron type, a pair of features is shown that can be used to discriminate that type from others in the same neuropil. Many although not all discriminations are highly accurate. Both intrinsic and boundary types are included as discriminative features. [file 41586_2024_7981_MOESM7_ESM.zip › DataS3/T5a.pdf]

T5b

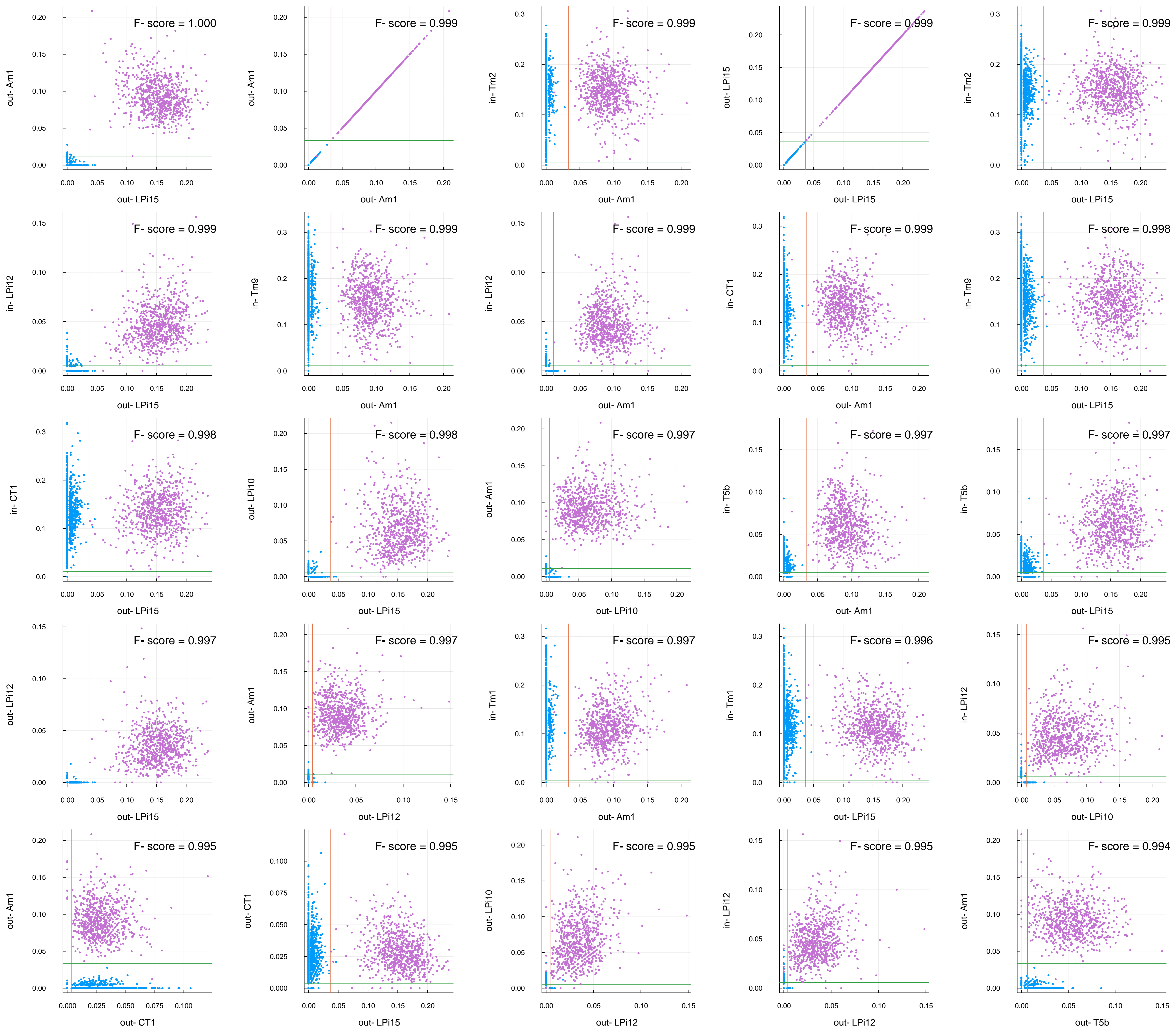

Supplement: Supplementary file 7 — Discriminating 2D projections for neuropil-intrinsic types. For each interneuron type, a pair of features is shown that can be used to discriminate that type from others in the same neuropil. Many although not all discriminations are highly accurate. Both intrinsic and boundary types are included as discriminative features. [file 41586_2024_7981_MOESM7_ESM.zip › DataS3/T5b.pdf]

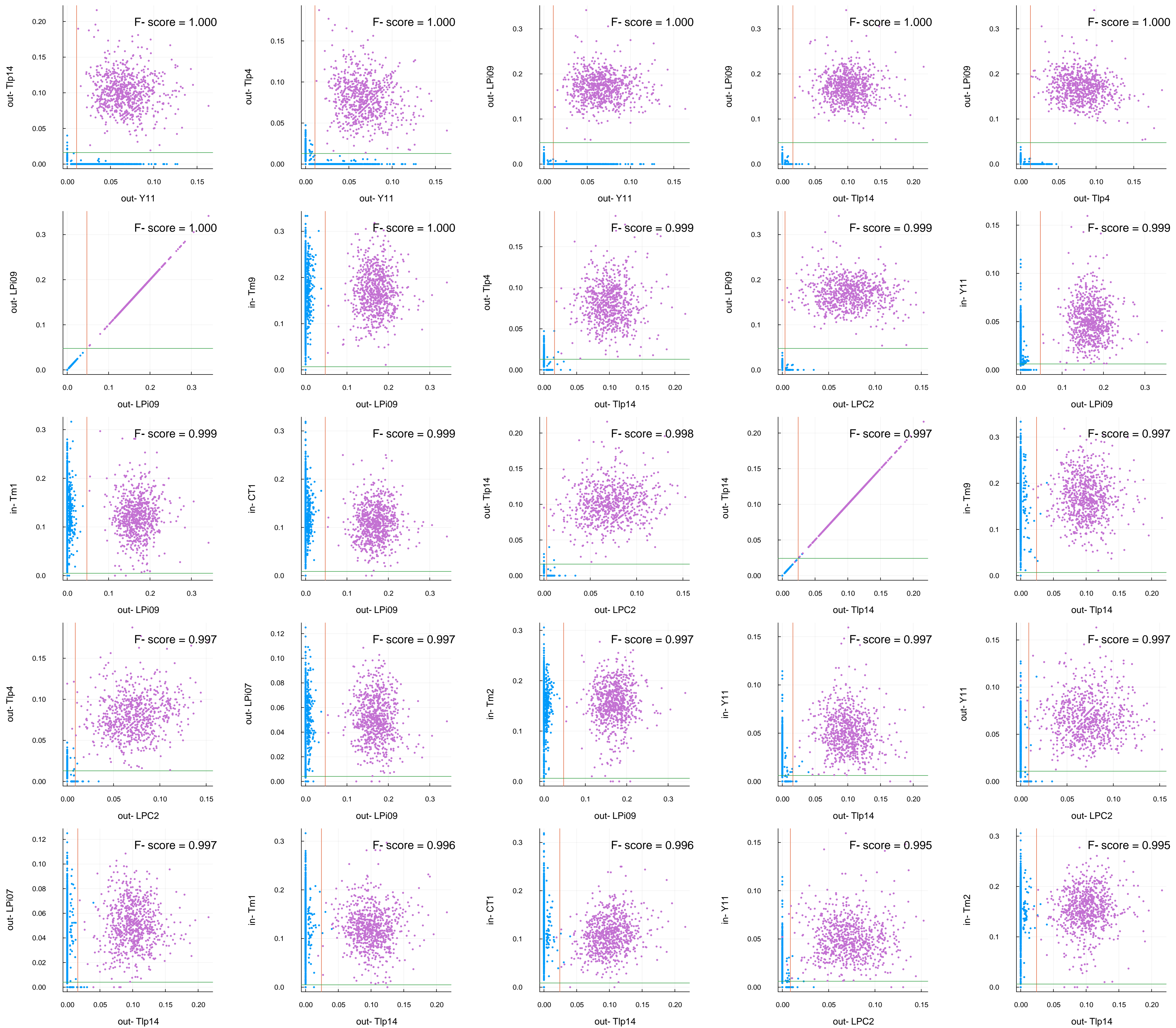

Supplement: Supplementary file 7 — Discriminating 2D projections for neuropil-intrinsic types. For each interneuron type, a pair of features is shown that can be used to discriminate that type from others in the same neuropil. Many although not all discriminations are highly accurate. Both intrinsic and boundary types are included as discriminative features. [file 41586_2024_7981_MOESM7_ESM.zip › DataS3/T5c.pdf]

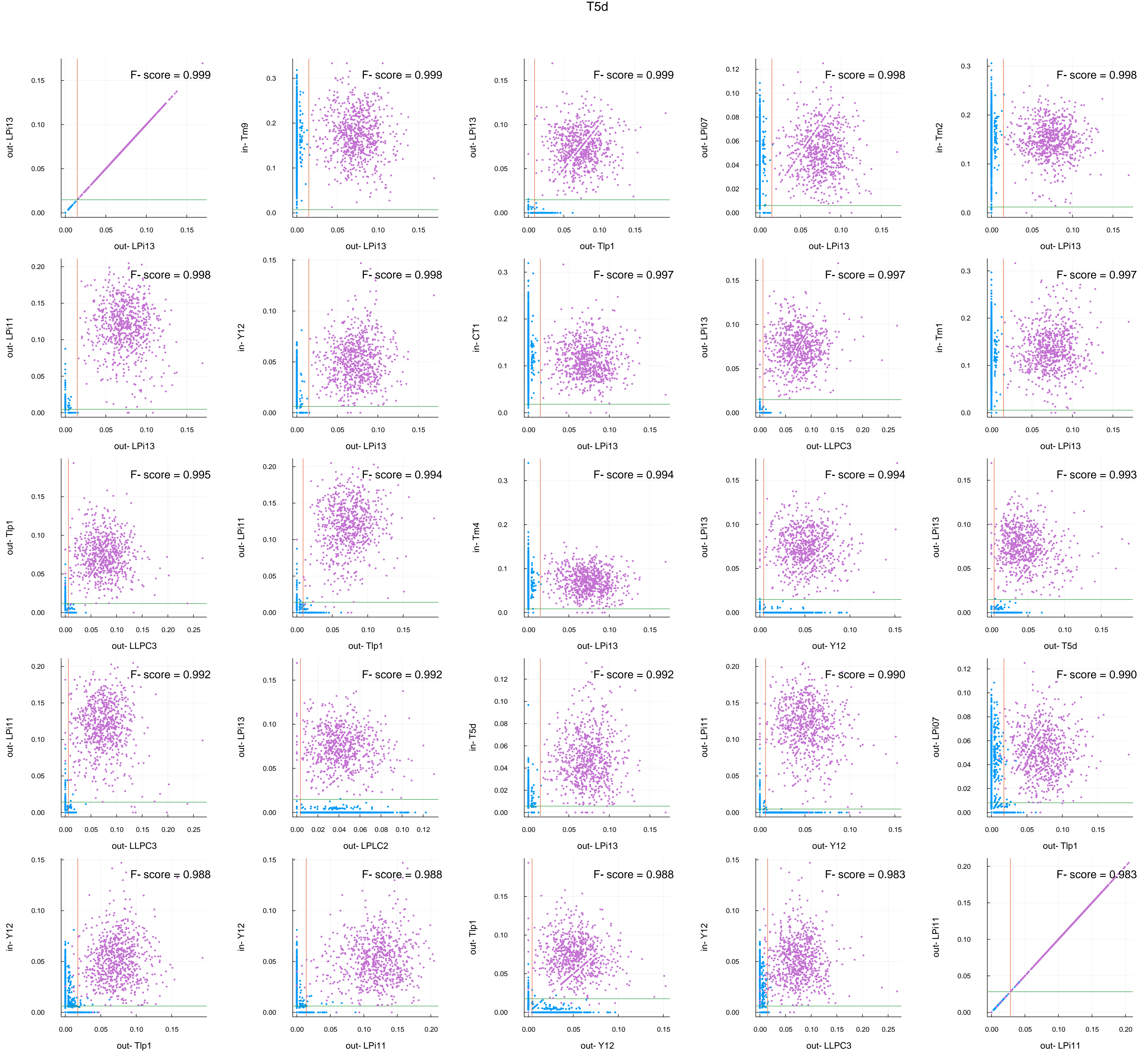

Supplement: Supplementary file 7 — Discriminating 2D projections for neuropil-intrinsic types. For each interneuron type, a pair of features is shown that can be used to discriminate that type from others in the same neuropil. Many although not all discriminations are highly accurate. Both intrinsic and boundary types are included as discriminative features. [file 41586_2024_7981_MOESM7_ESM.zip › DataS3/T5d.pdf]

Tlp1

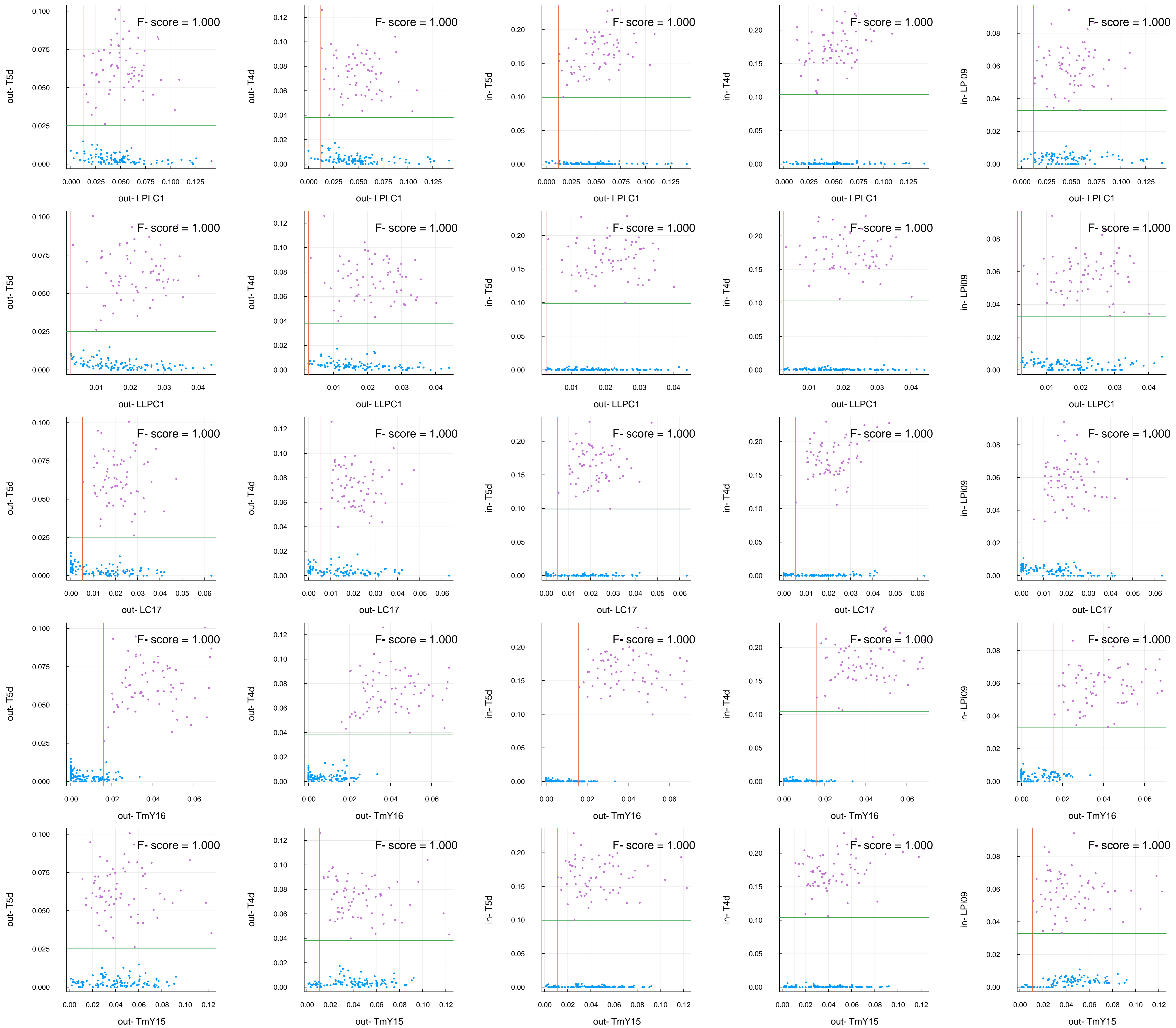

Supplement: Supplementary file 7 — Discriminating 2D projections for neuropil-intrinsic types. For each interneuron type, a pair of features is shown that can be used to discriminate that type from others in the same neuropil. Many although not all discriminations are highly accurate. Both intrinsic and boundary types are included as discriminative features. [file 41586_2024_7981_MOESM7_ESM.zip › DataS3/Tlp1.pdf]

Tlp14

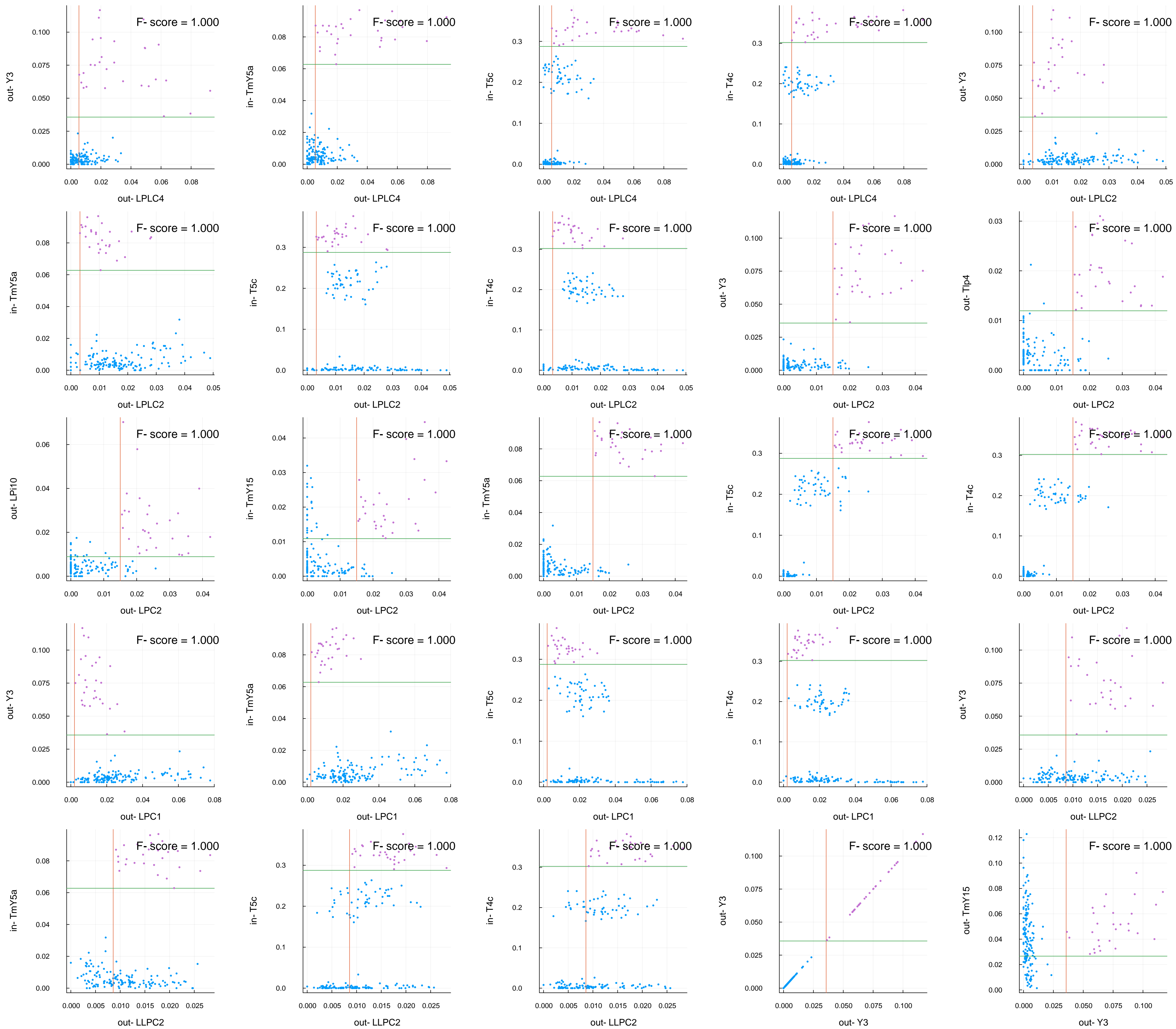

Supplement: Supplementary file 7 — Discriminating 2D projections for neuropil-intrinsic types. For each interneuron type, a pair of features is shown that can be used to discriminate that type from others in the same neuropil. Many although not all discriminations are highly accurate. Both intrinsic and boundary types are included as discriminative features. [file 41586_2024_7981_MOESM7_ESM.zip › DataS3/Tlp14.pdf]

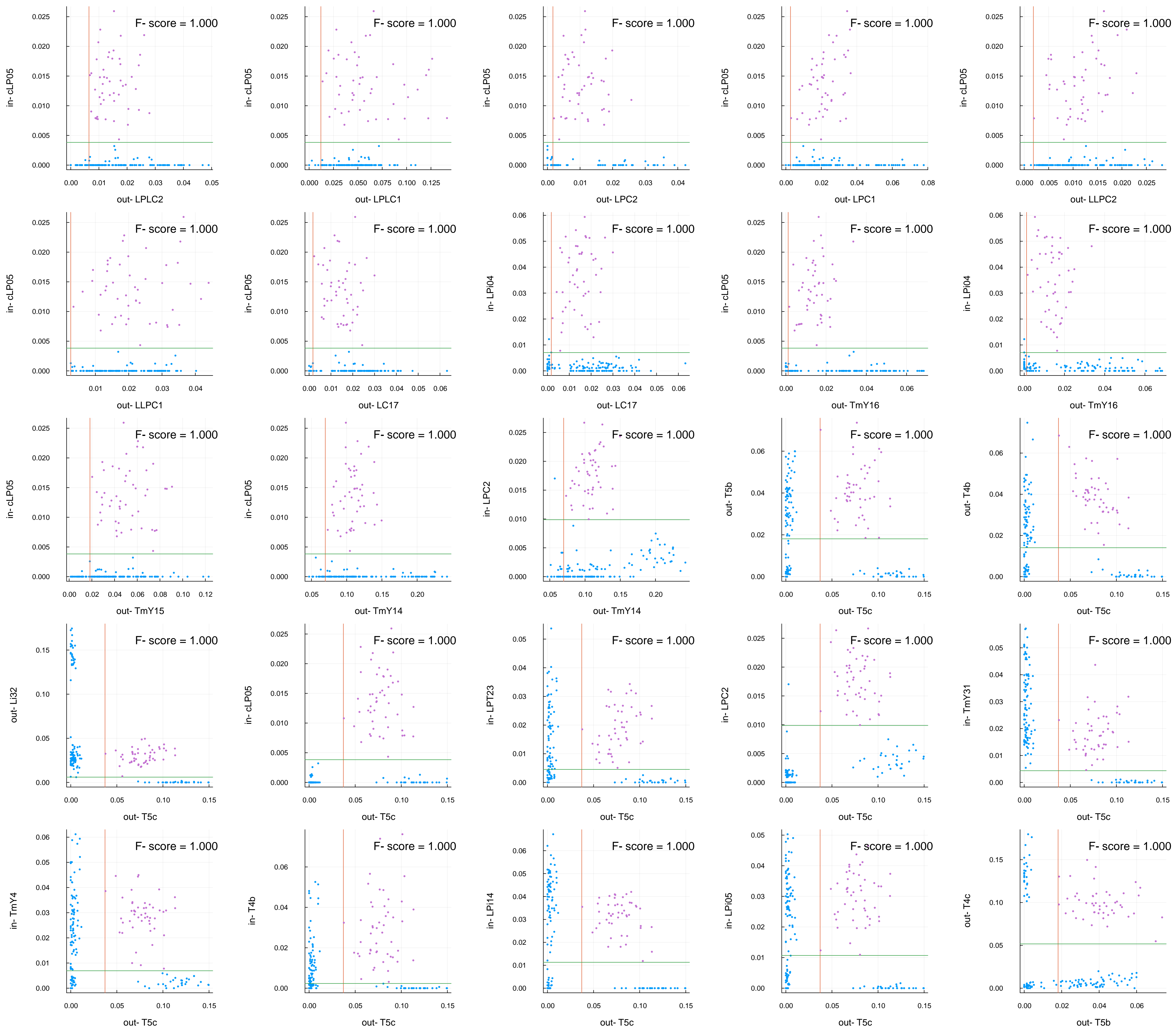

Supplement: Supplementary file 7 — Discriminating 2D projections for neuropil-intrinsic types. For each interneuron type, a pair of features is shown that can be used to discriminate that type from others in the same neuropil. Many although not all discriminations are highly accurate. Both intrinsic and boundary types are included as discriminative features. [file 41586_2024_7981_MOESM7_ESM.zip › DataS3/Tlp4.pdf]

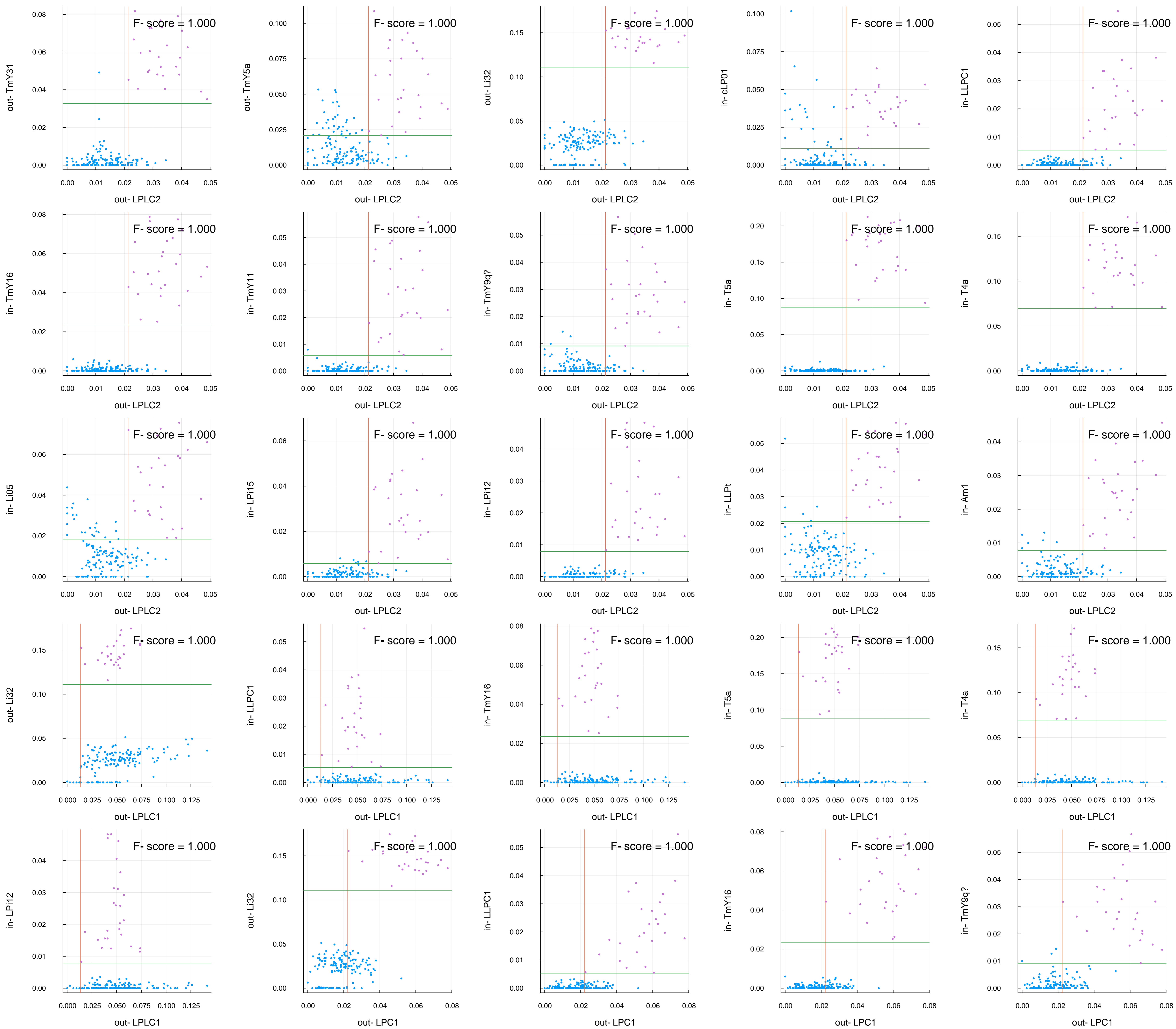

Supplement: Supplementary file 7 — Discriminating 2D projections for neuropil-intrinsic types. For each interneuron type, a pair of features is shown that can be used to discriminate that type from others in the same neuropil. Many although not all discriminations are highly accurate. Both intrinsic and boundary types are included as discriminative features. [file 41586_2024_7981_MOESM7_ESM.zip › DataS3/Tlp5.pdf]

Tm1

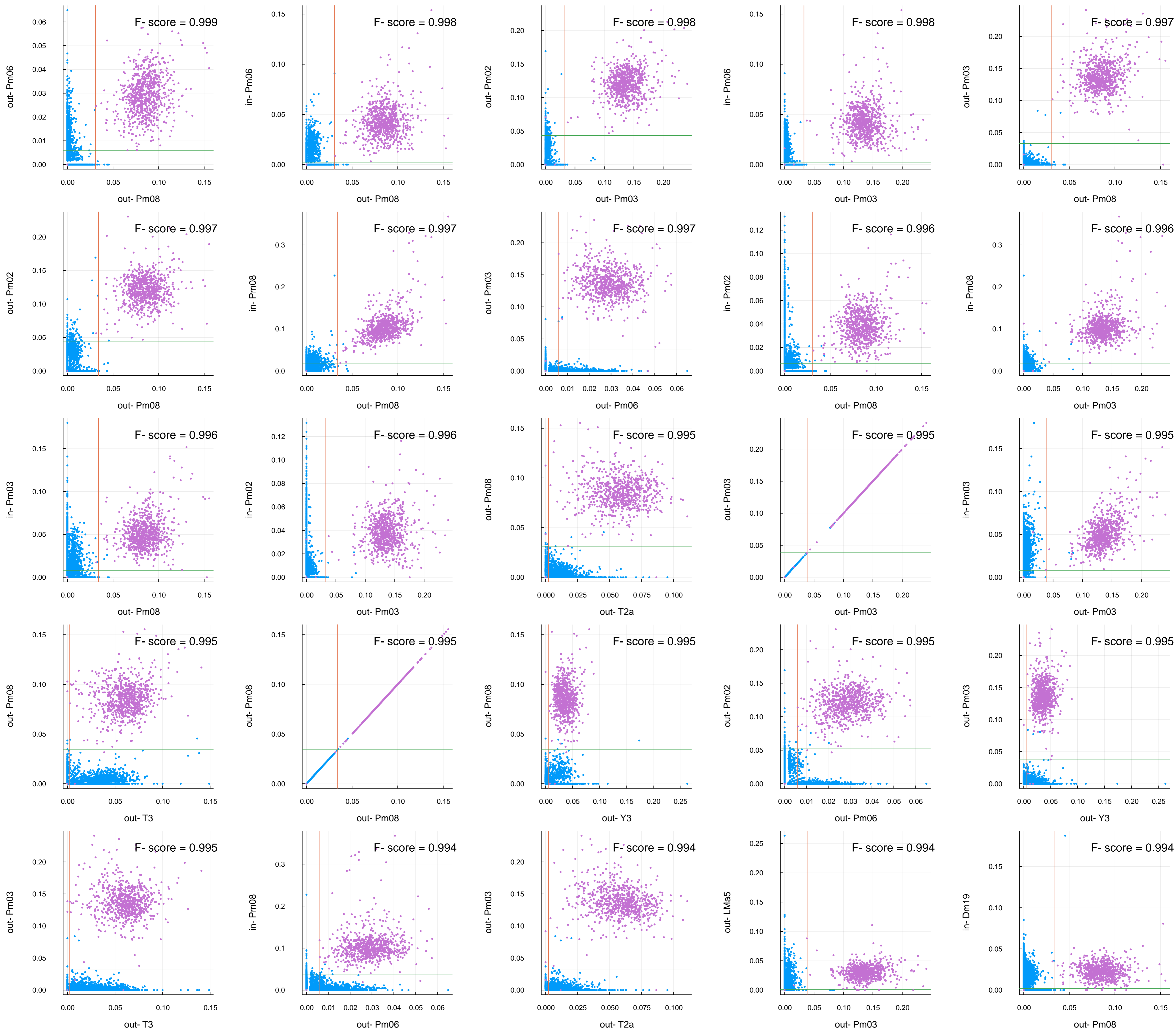

Supplement: Supplementary file 7 — Discriminating 2D projections for neuropil-intrinsic types. For each interneuron type, a pair of features is shown that can be used to discriminate that type from others in the same neuropil. Many although not all discriminations are highly accurate. Both intrinsic and boundary types are included as discriminative features. [file 41586_2024_7981_MOESM7_ESM.zip › DataS3/Tm1.pdf]

Tm16

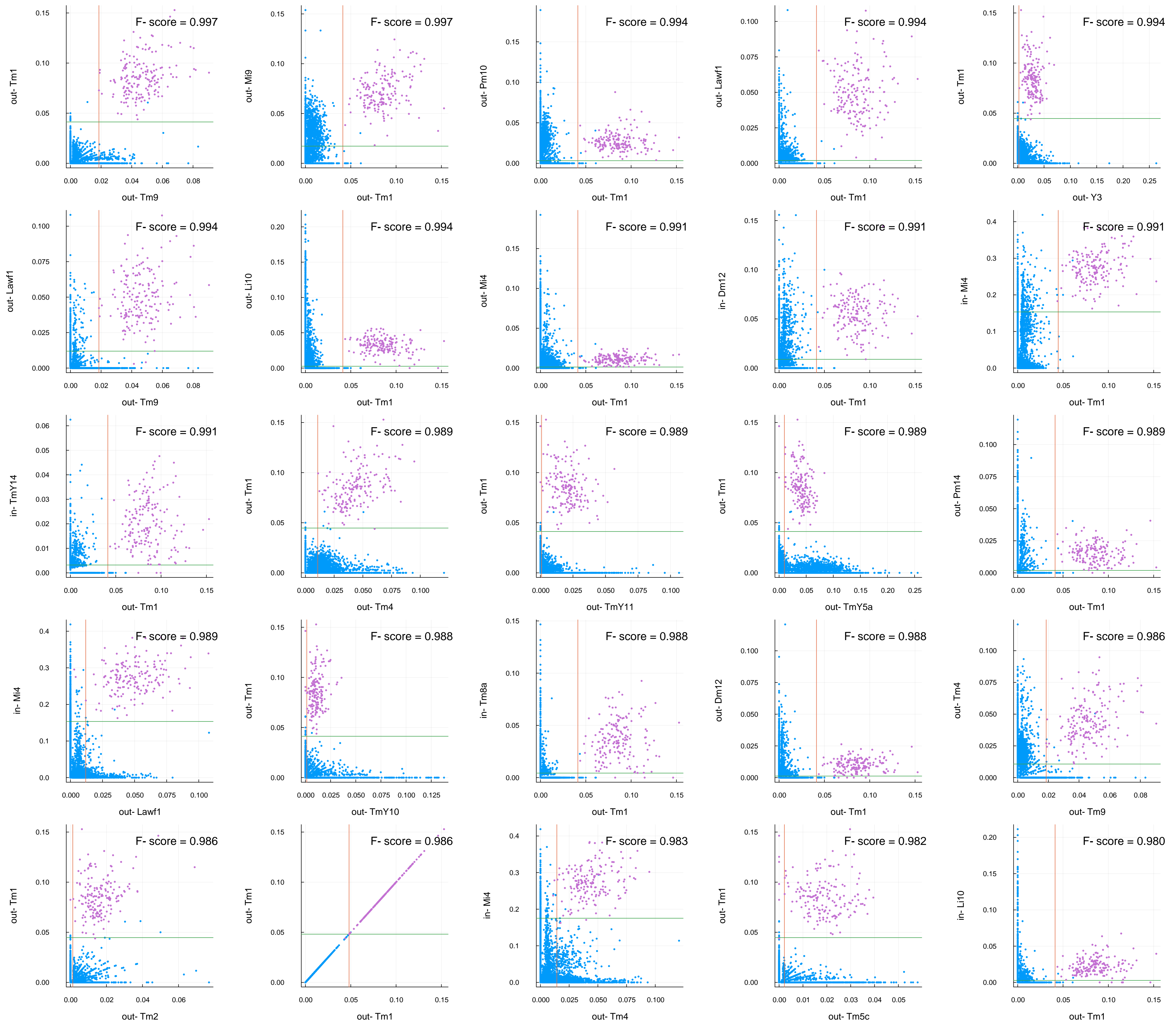

Supplement: Supplementary file 7 — Discriminating 2D projections for neuropil-intrinsic types. For each interneuron type, a pair of features is shown that can be used to discriminate that type from others in the same neuropil. Many although not all discriminations are highly accurate. Both intrinsic and boundary types are included as discriminative features. [file 41586_2024_7981_MOESM7_ESM.zip › DataS3/Tm16.pdf]

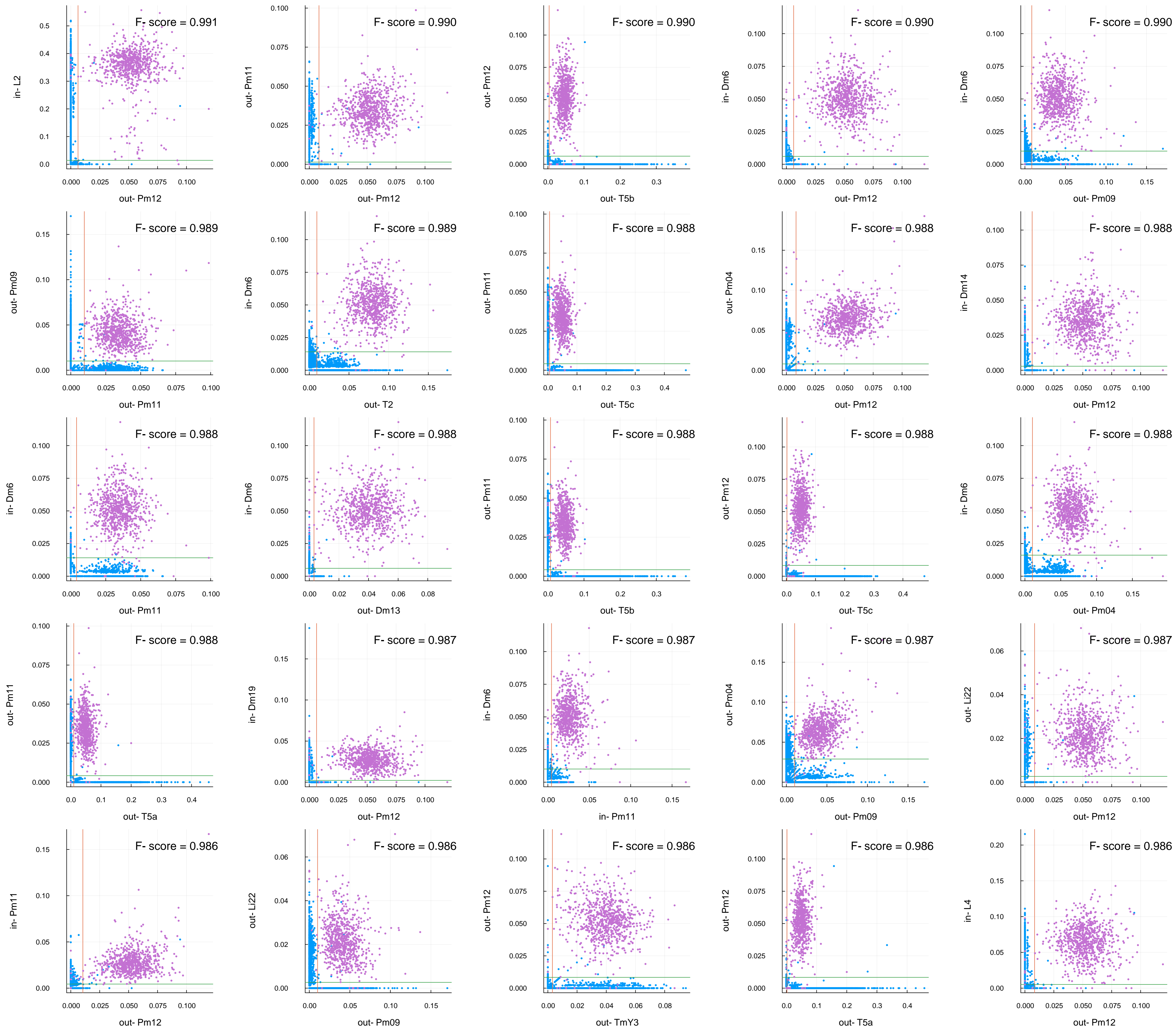

Supplement: Supplementary file 7 — Discriminating 2D projections for neuropil-intrinsic types. For each interneuron type, a pair of features is shown that can be used to discriminate that type from others in the same neuropil. Many although not all discriminations are highly accurate. Both intrinsic and boundary types are included as discriminative features. [file 41586_2024_7981_MOESM7_ESM.zip › DataS3/Tm2.pdf]

Tm20

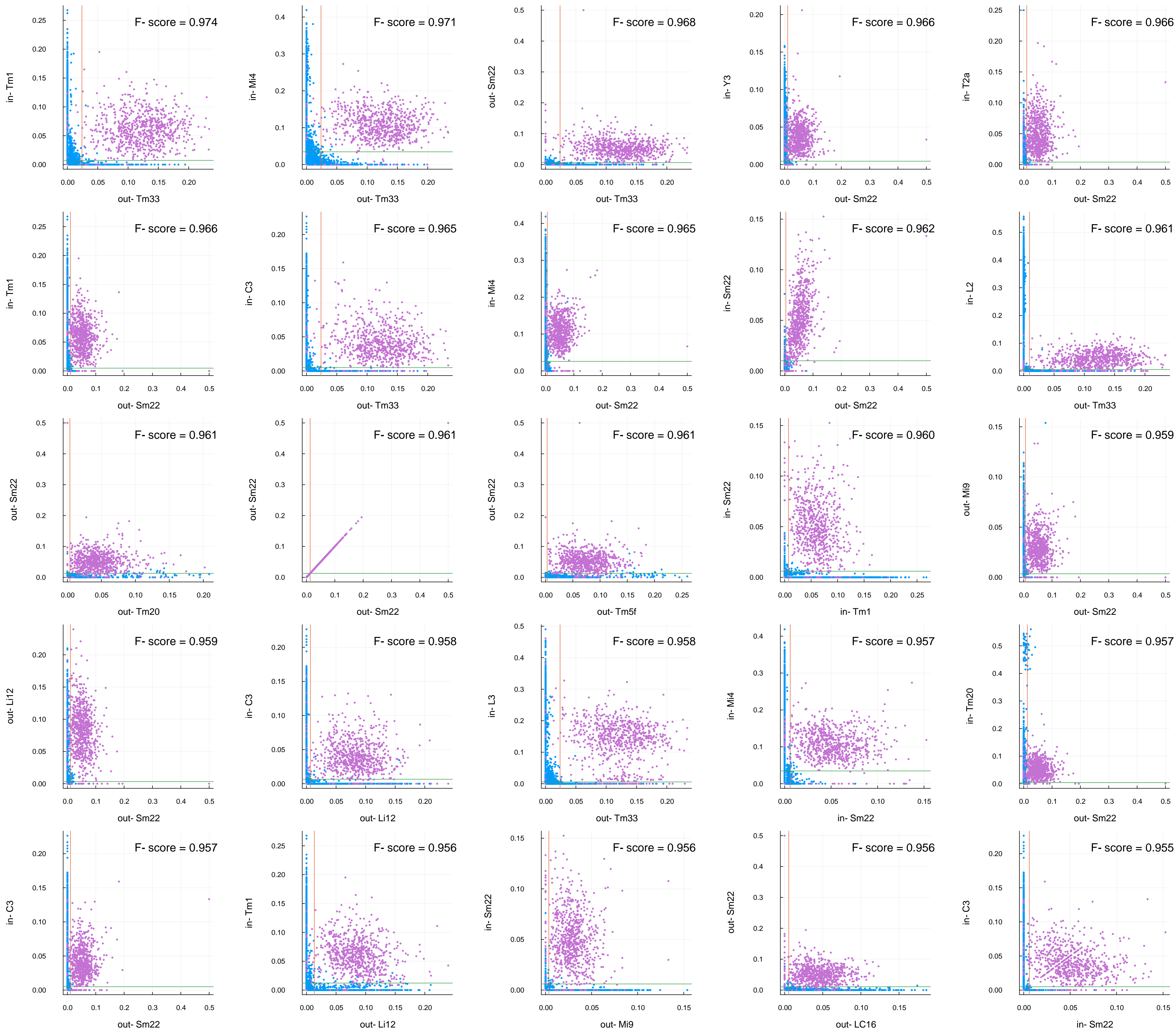

Supplement: Supplementary file 7 — Discriminating 2D projections for neuropil-intrinsic types. For each interneuron type, a pair of features is shown that can be used to discriminate that type from others in the same neuropil. Many although not all discriminations are highly accurate. Both intrinsic and boundary types are included as discriminative features. [file 41586_2024_7981_MOESM7_ESM.zip › DataS3/Tm20.pdf]

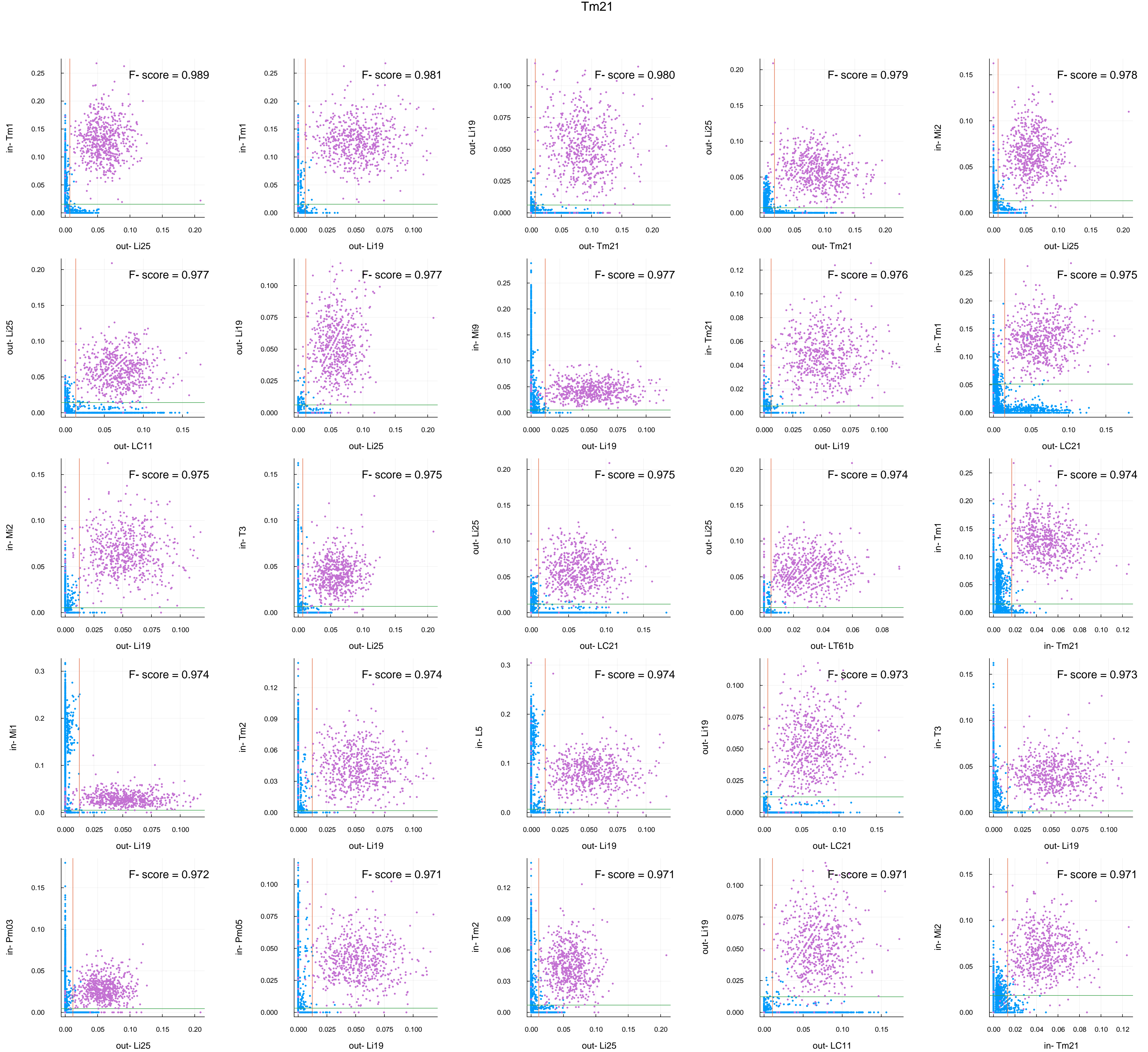

Supplement: Supplementary file 7 — Discriminating 2D projections for neuropil-intrinsic types. For each interneuron type, a pair of features is shown that can be used to discriminate that type from others in the same neuropil. Many although not all discriminations are highly accurate. Both intrinsic and boundary types are included as discriminative features. [file 41586_2024_7981_MOESM7_ESM.zip › DataS3/Tm21.pdf]

Tm25

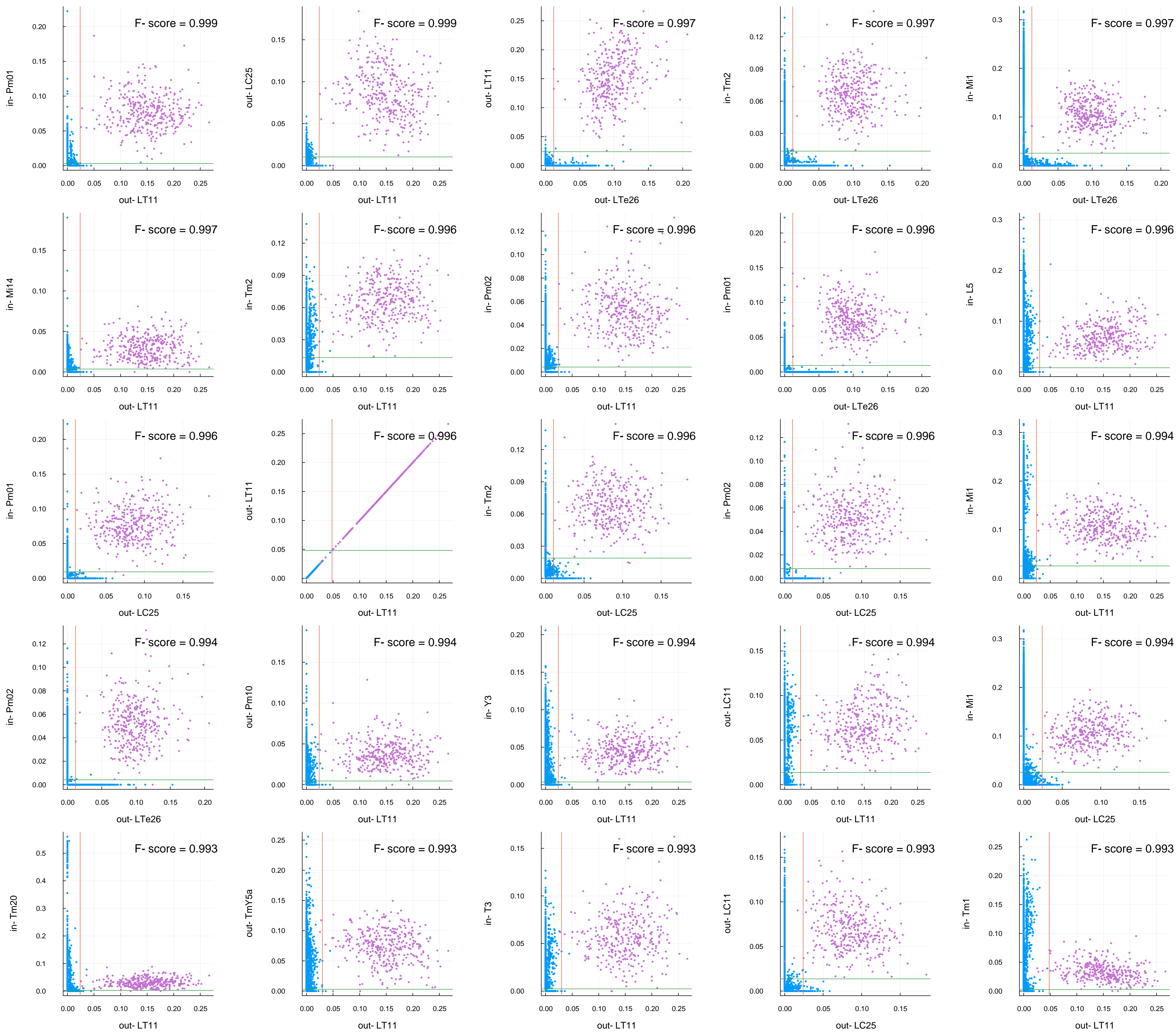

Supplement: Supplementary file 7 — Discriminating 2D projections for neuropil-intrinsic types. For each interneuron type, a pair of features is shown that can be used to discriminate that type from others in the same neuropil. Many although not all discriminations are highly accurate. Both intrinsic and boundary types are included as discriminative features. [file 41586_2024_7981_MOESM7_ESM.zip › DataS3/Tm25.pdf]

Tm27

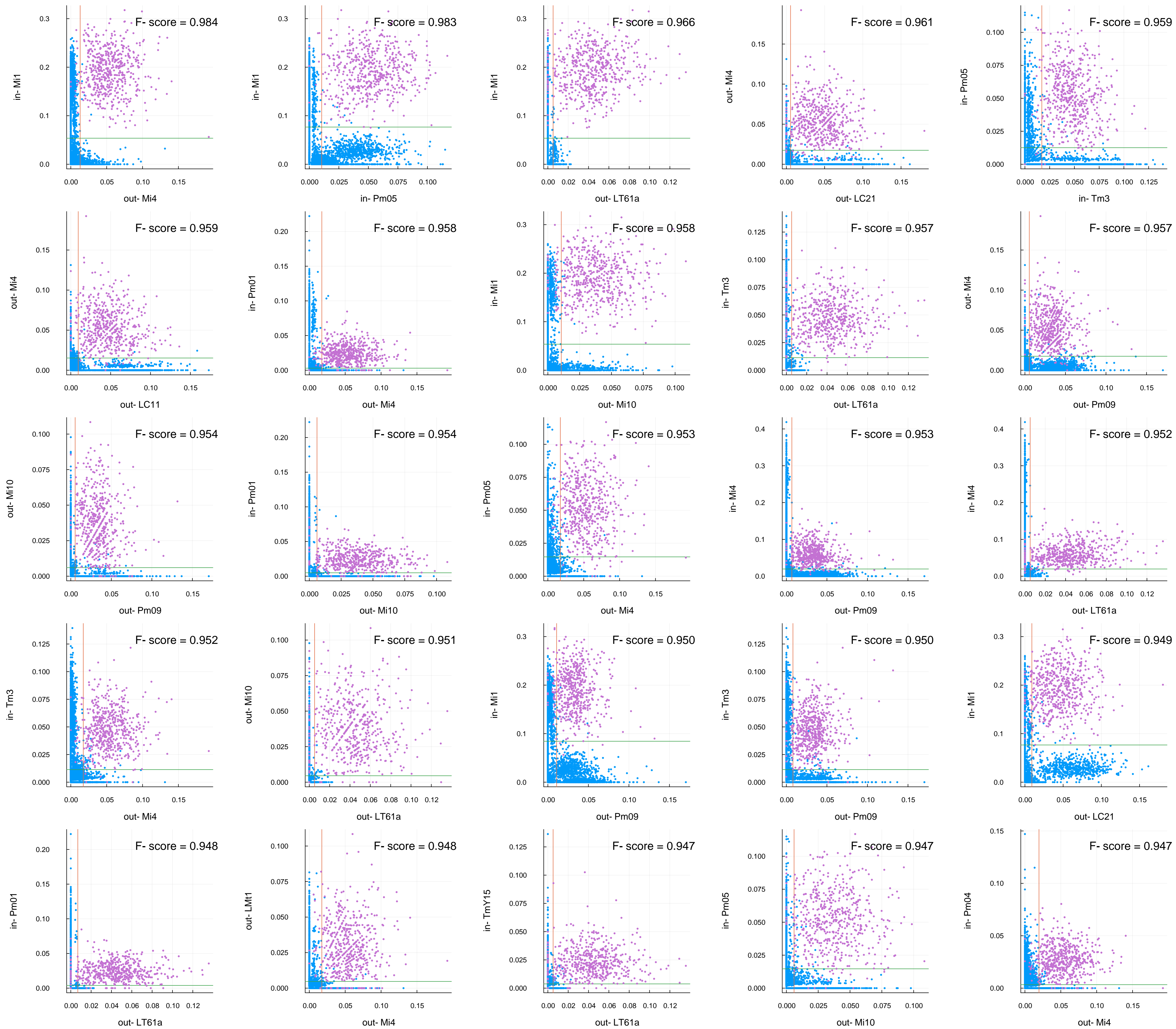

Supplement: Supplementary file 7 — Discriminating 2D projections for neuropil-intrinsic types. For each interneuron type, a pair of features is shown that can be used to discriminate that type from others in the same neuropil. Many although not all discriminations are highly accurate. Both intrinsic and boundary types are included as discriminative features. [file 41586_2024_7981_MOESM7_ESM.zip › DataS3/Tm27.pdf]

Tm3

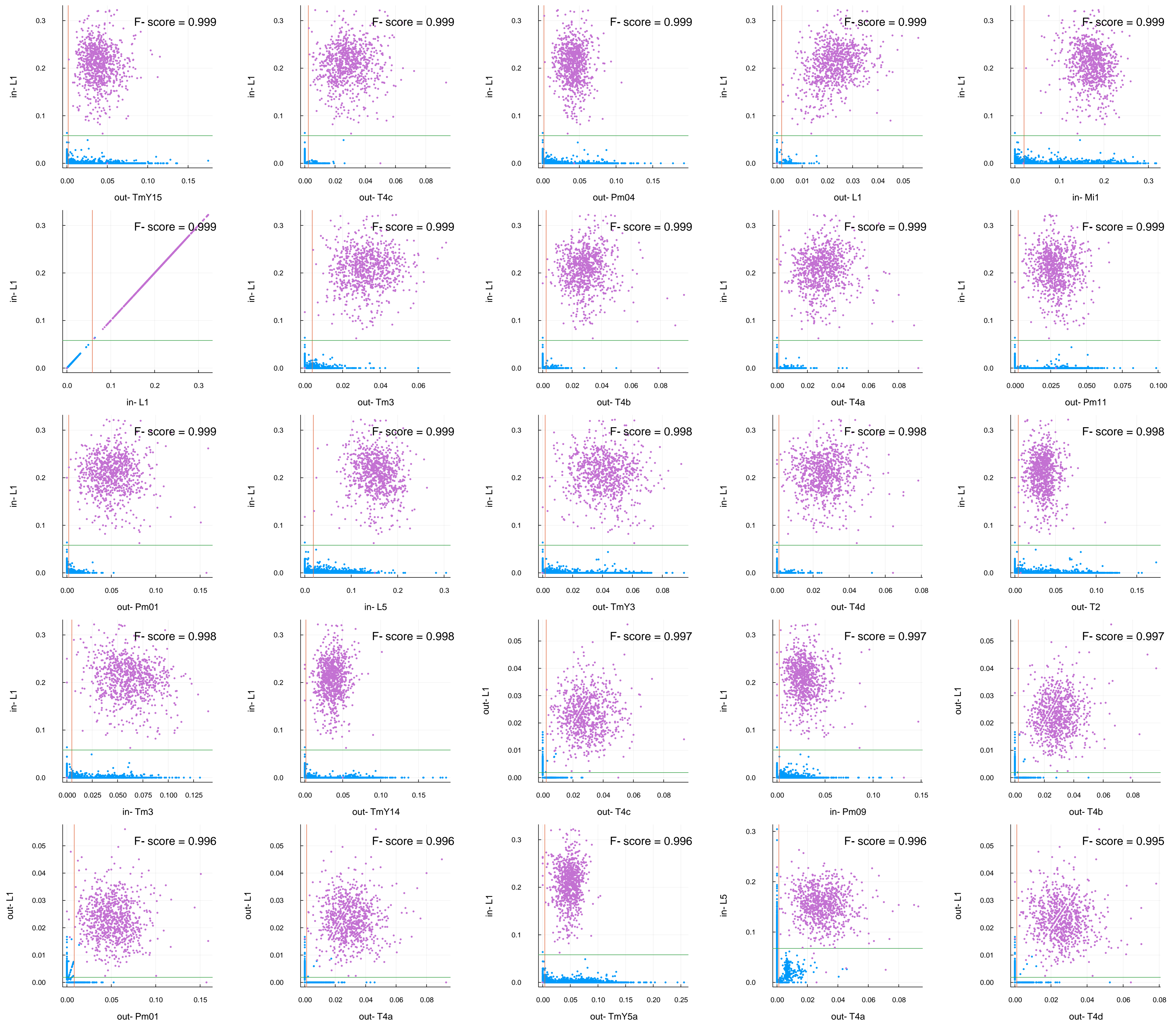

Supplement: Supplementary file 7 — Discriminating 2D projections for neuropil-intrinsic types. For each interneuron type, a pair of features is shown that can be used to discriminate that type from others in the same neuropil. Many although not all discriminations are highly accurate. Both intrinsic and boundary types are included as discriminative features. [file 41586_2024_7981_MOESM7_ESM.zip › DataS3/Tm3.pdf]

Tm31

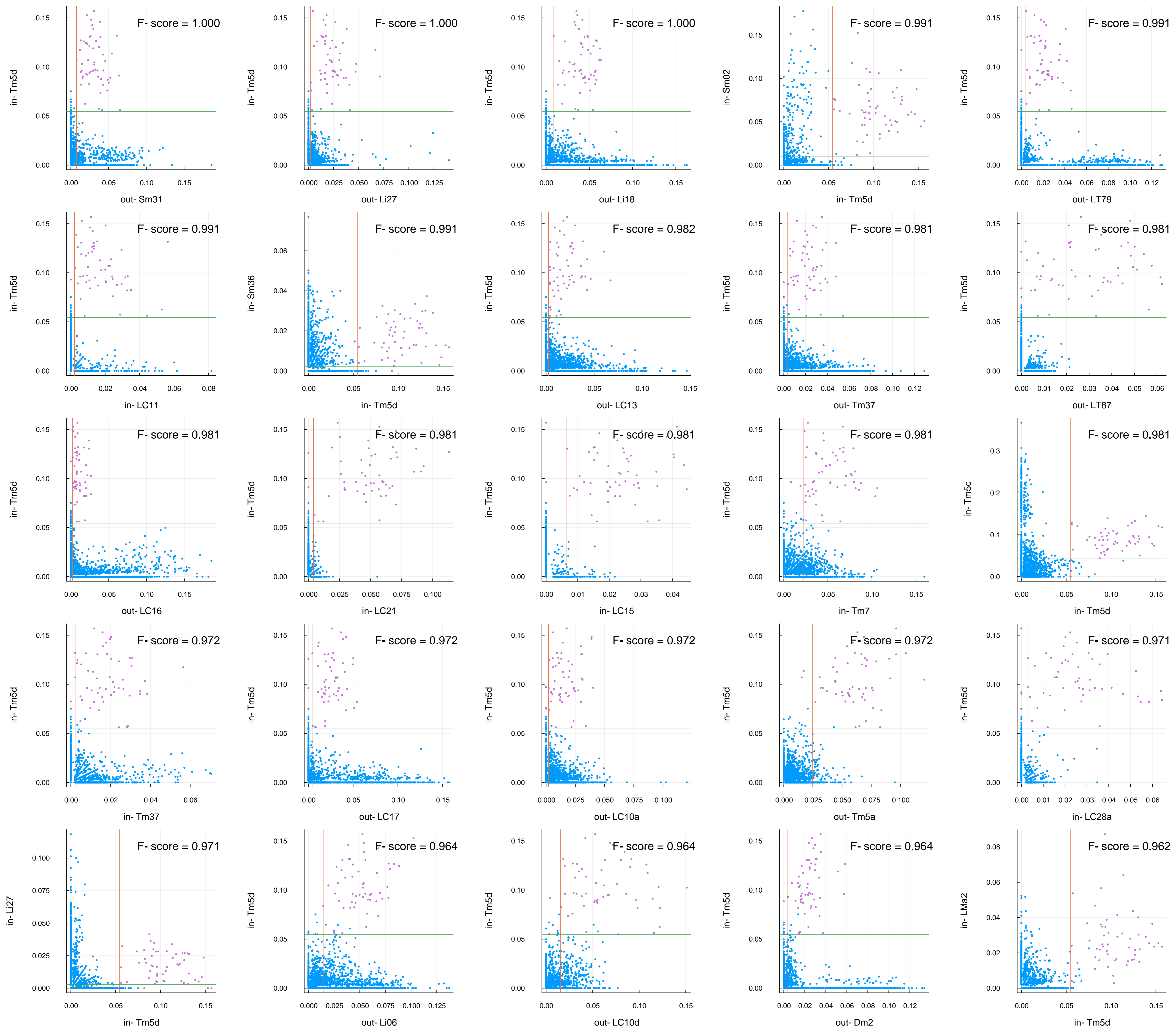

Supplement: Supplementary file 7 — Discriminating 2D projections for neuropil-intrinsic types. For each interneuron type, a pair of features is shown that can be used to discriminate that type from others in the same neuropil. Many although not all discriminations are highly accurate. Both intrinsic and boundary types are included as discriminative features. [file 41586_2024_7981_MOESM7_ESM.zip › DataS3/Tm31.pdf]

Tm32

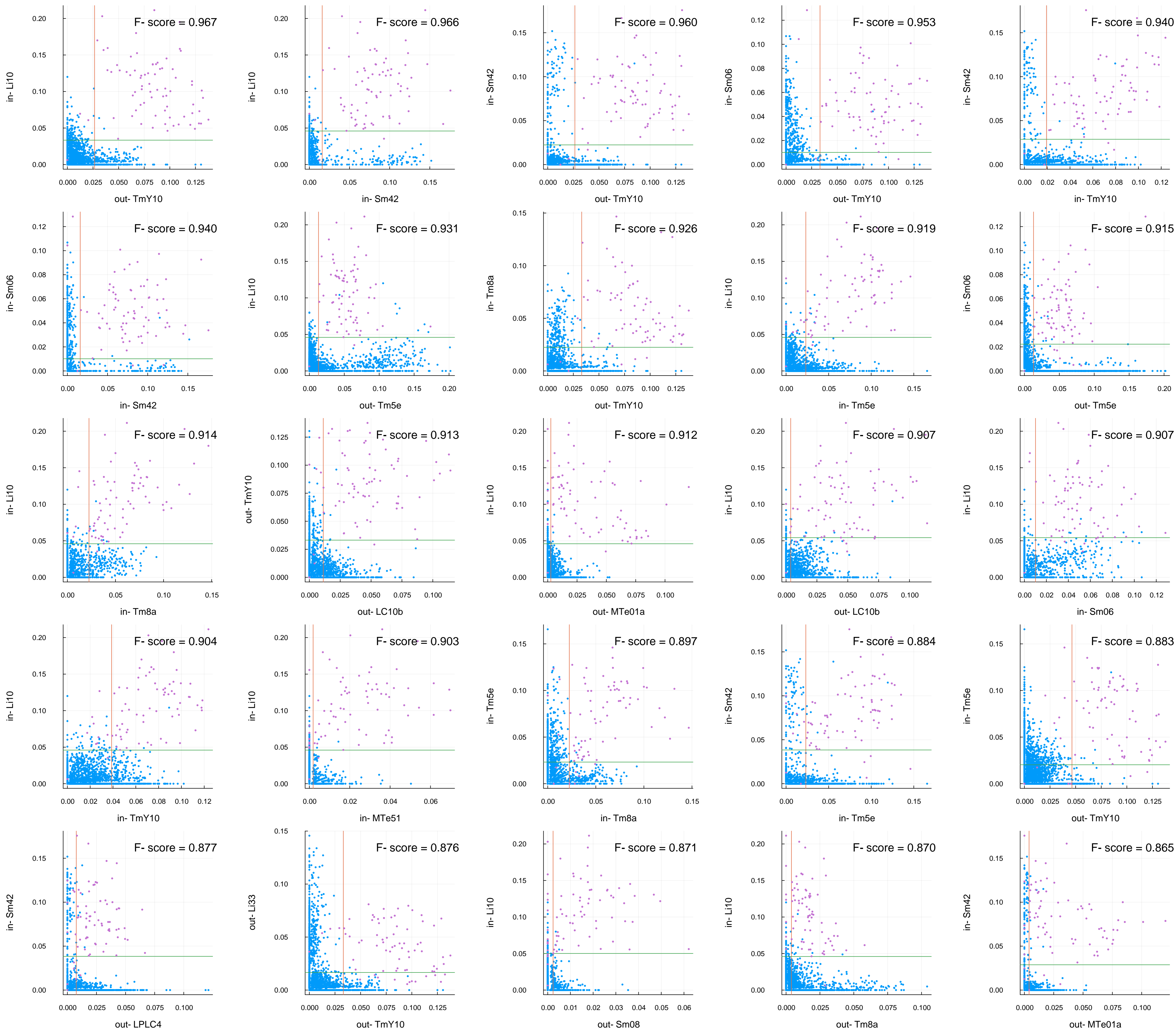

Supplement: Supplementary file 7 — Discriminating 2D projections for neuropil-intrinsic types. For each interneuron type, a pair of features is shown that can be used to discriminate that type from others in the same neuropil. Many although not all discriminations are highly accurate. Both intrinsic and boundary types are included as discriminative features. [file 41586_2024_7981_MOESM7_ESM.zip › DataS3/Tm32.pdf]

Tm33

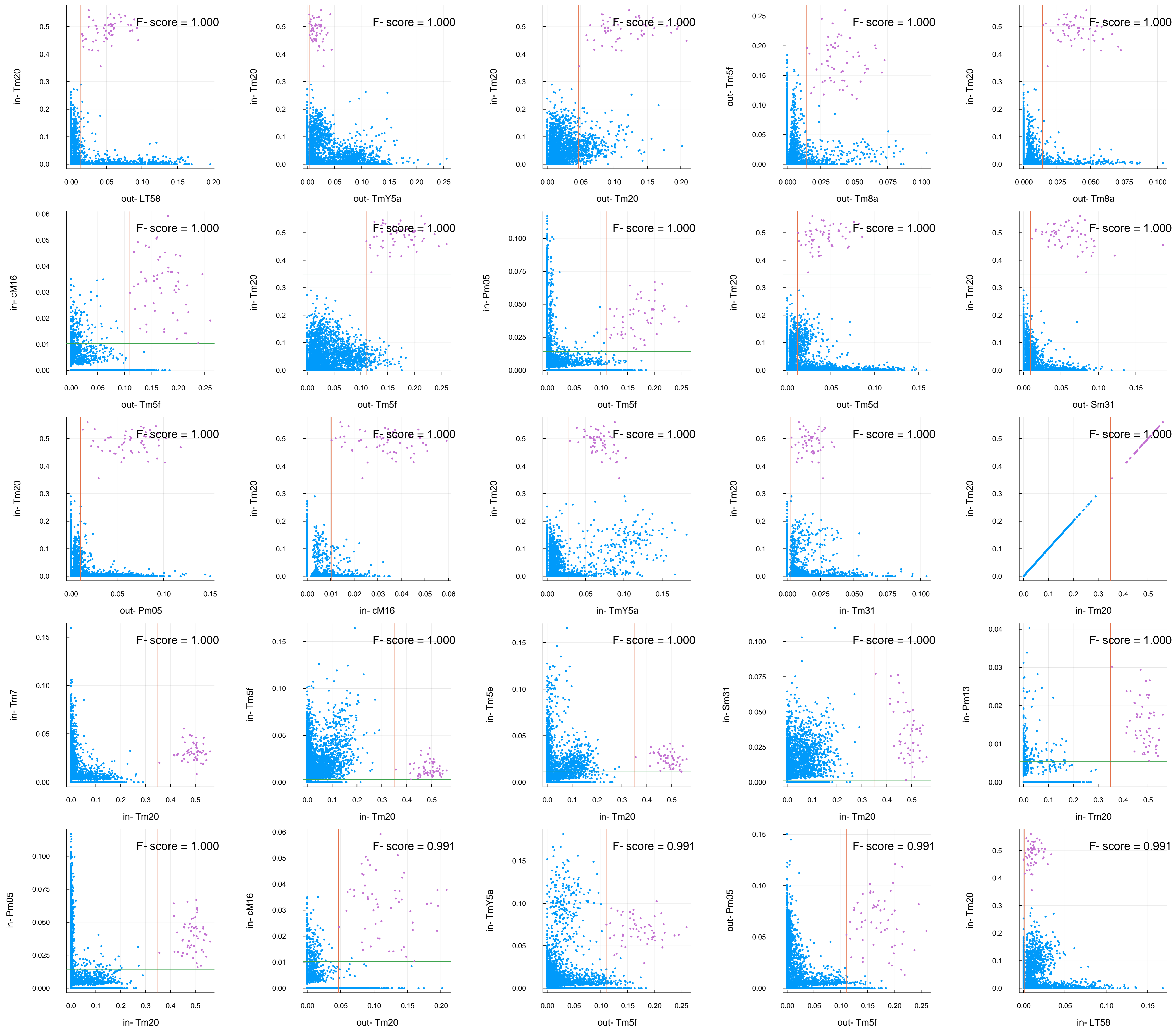

Supplement: Supplementary file 7 — Discriminating 2D projections for neuropil-intrinsic types. For each interneuron type, a pair of features is shown that can be used to discriminate that type from others in the same neuropil. Many although not all discriminations are highly accurate. Both intrinsic and boundary types are included as discriminative features. [file 41586_2024_7981_MOESM7_ESM.zip › DataS3/Tm33.pdf]

Tm34

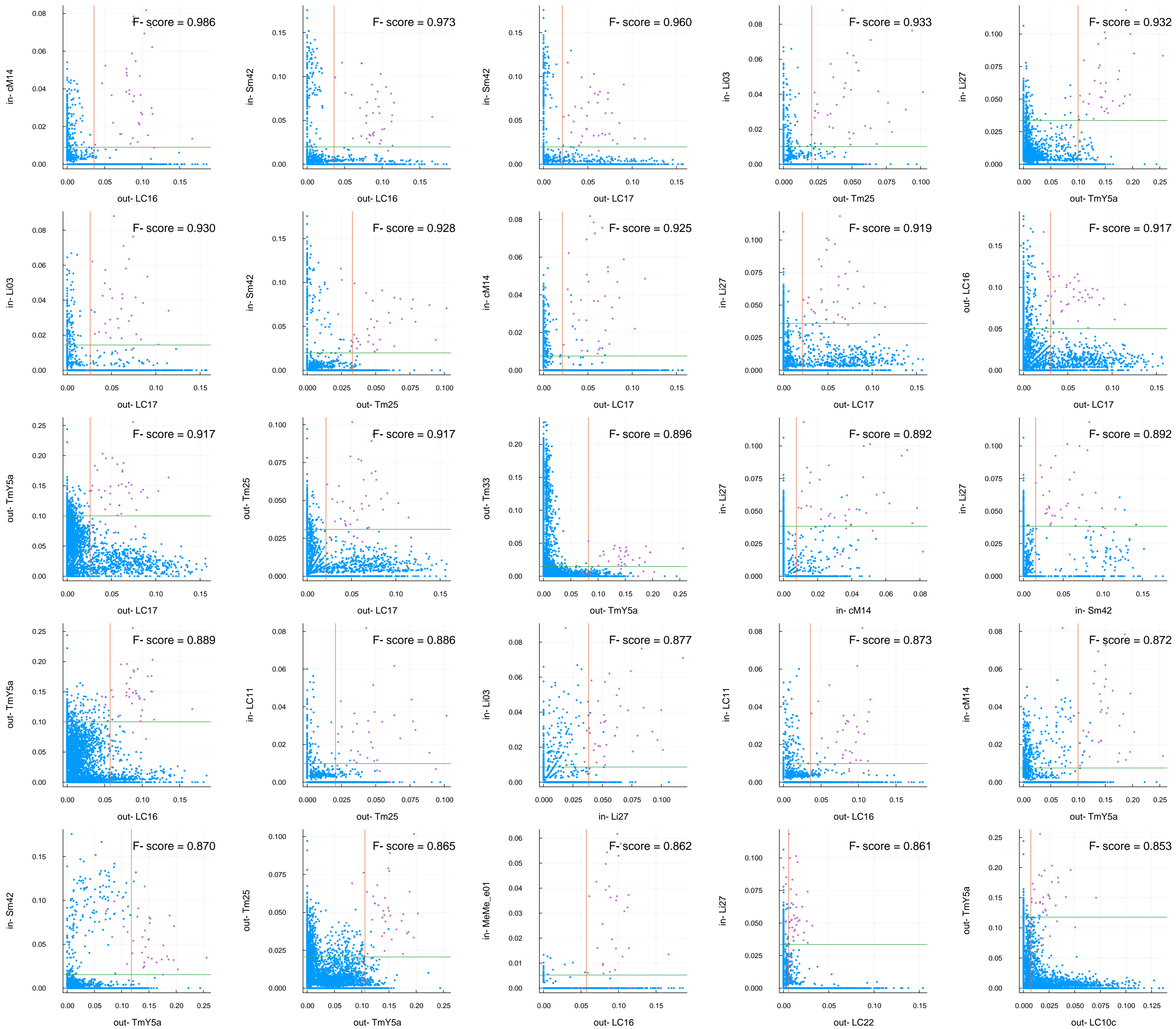

Supplement: Supplementary file 7 — Discriminating 2D projections for neuropil-intrinsic types. For each interneuron type, a pair of features is shown that can be used to discriminate that type from others in the same neuropil. Many although not all discriminations are highly accurate. Both intrinsic and boundary types are included as discriminative features. [file 41586_2024_7981_MOESM7_ESM.zip › DataS3/Tm34.pdf]

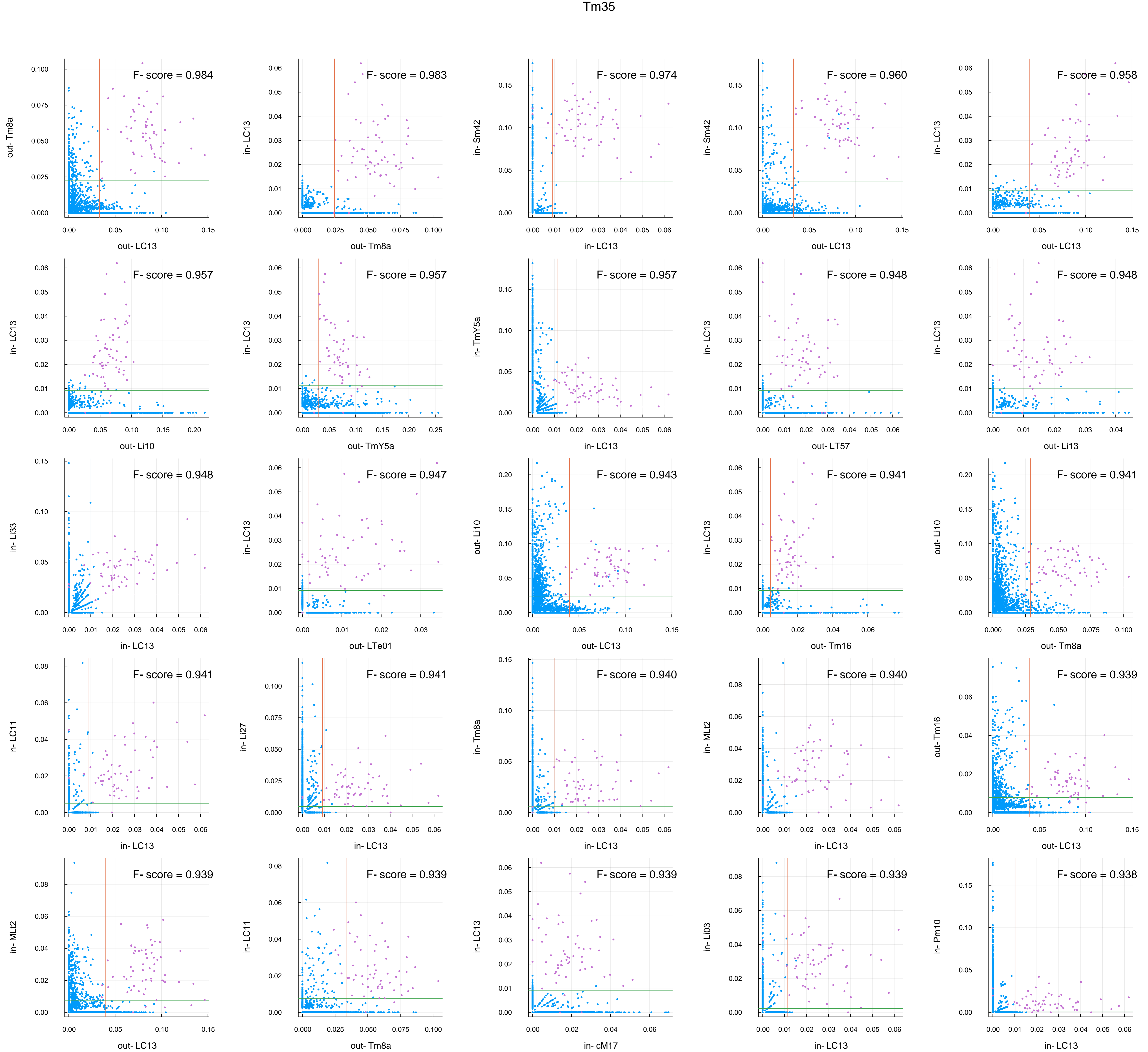

Supplement: Supplementary file 7 — Discriminating 2D projections for neuropil-intrinsic types. For each interneuron type, a pair of features is shown that can be used to discriminate that type from others in the same neuropil. Many although not all discriminations are highly accurate. Both intrinsic and boundary types are included as discriminative features. [file 41586_2024_7981_MOESM7_ESM.zip › DataS3/Tm35.pdf]

Tm36

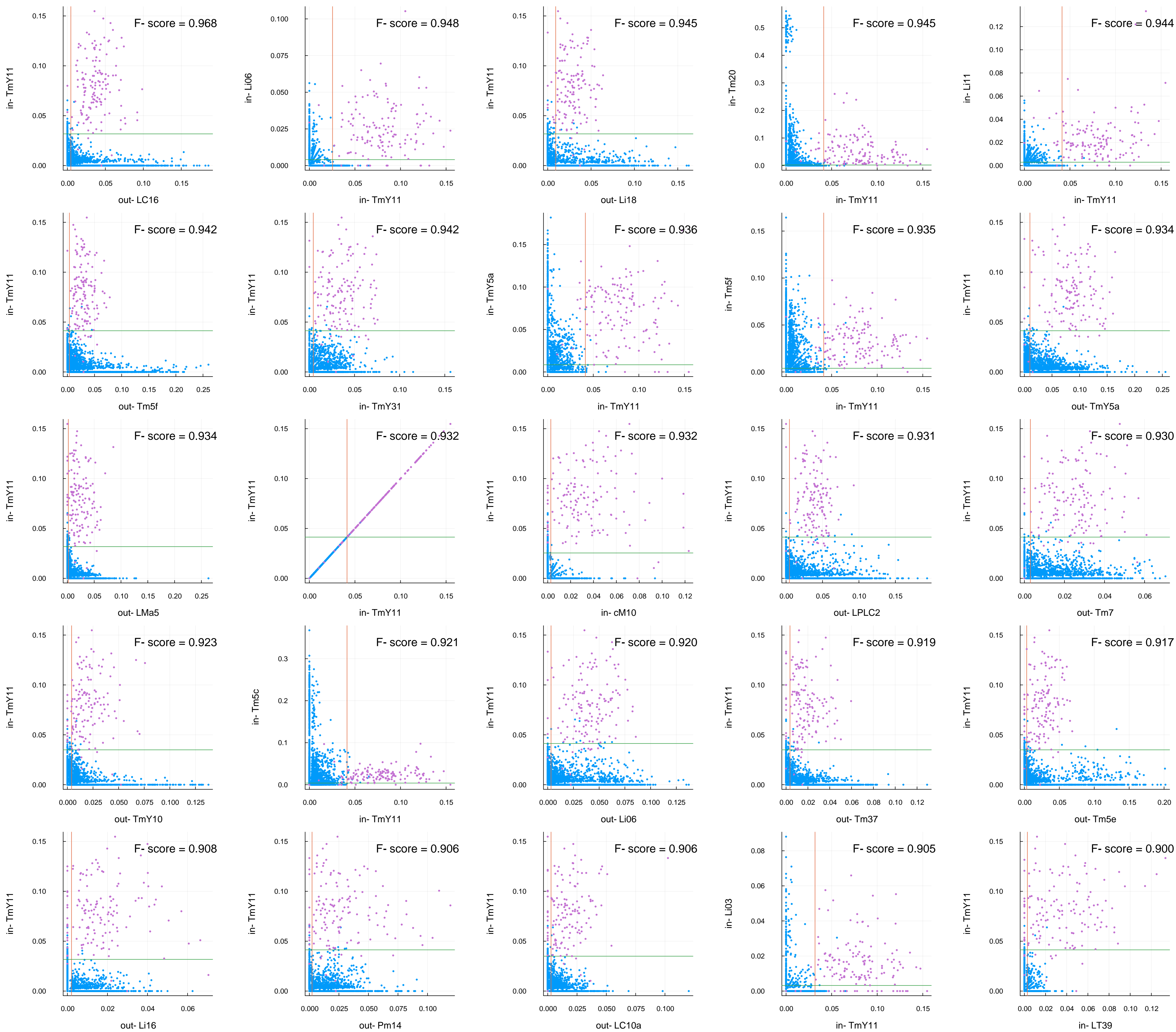

Supplement: Supplementary file 7 — Discriminating 2D projections for neuropil-intrinsic types. For each interneuron type, a pair of features is shown that can be used to discriminate that type from others in the same neuropil. Many although not all discriminations are highly accurate. Both intrinsic and boundary types are included as discriminative features. [file 41586_2024_7981_MOESM7_ESM.zip › DataS3/Tm36.pdf]

Tm37

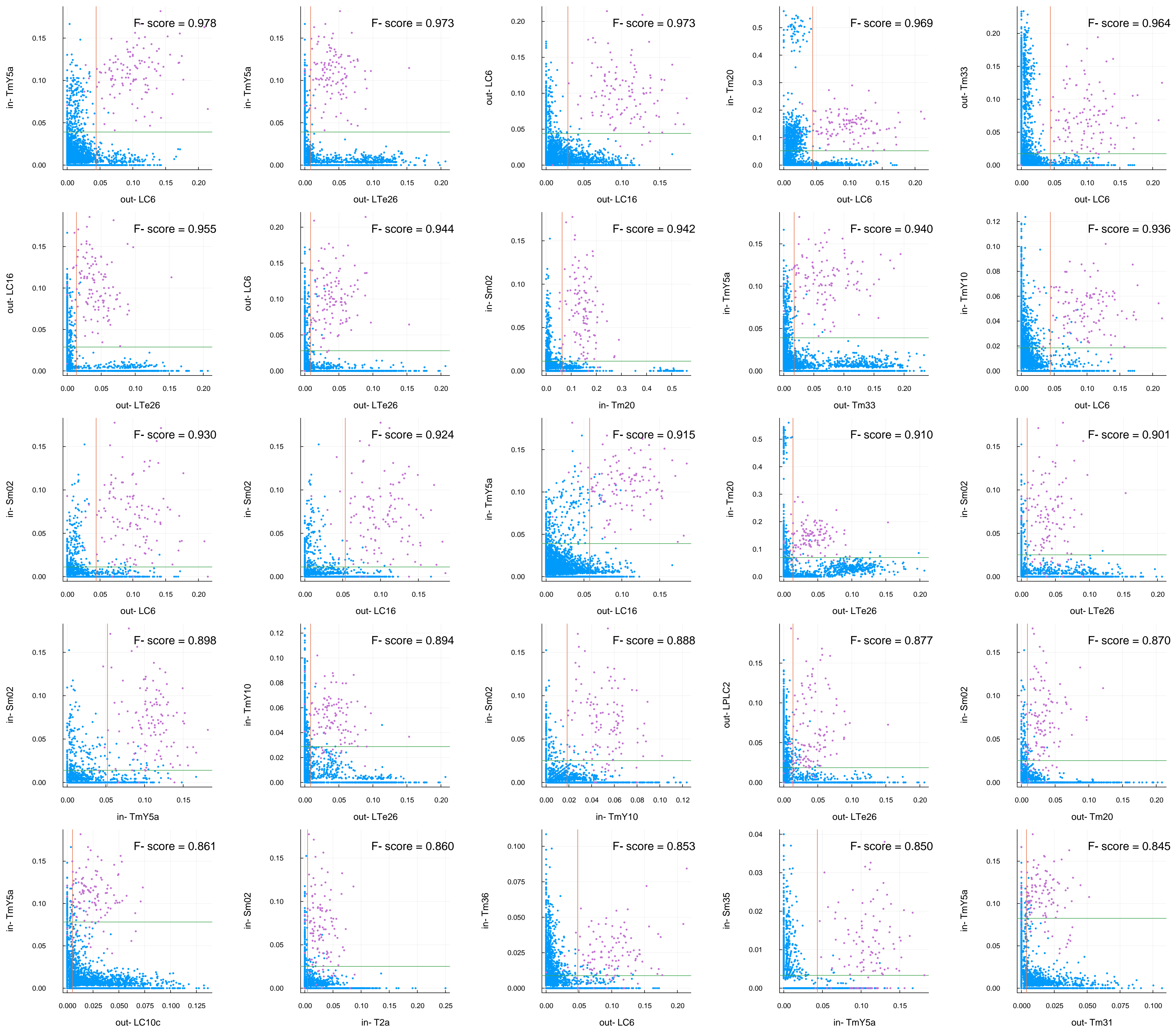

Supplement: Supplementary file 7 — Discriminating 2D projections for neuropil-intrinsic types. For each interneuron type, a pair of features is shown that can be used to discriminate that type from others in the same neuropil. Many although not all discriminations are highly accurate. Both intrinsic and boundary types are included as discriminative features. [file 41586_2024_7981_MOESM7_ESM.zip › DataS3/Tm37.pdf]

Tm4

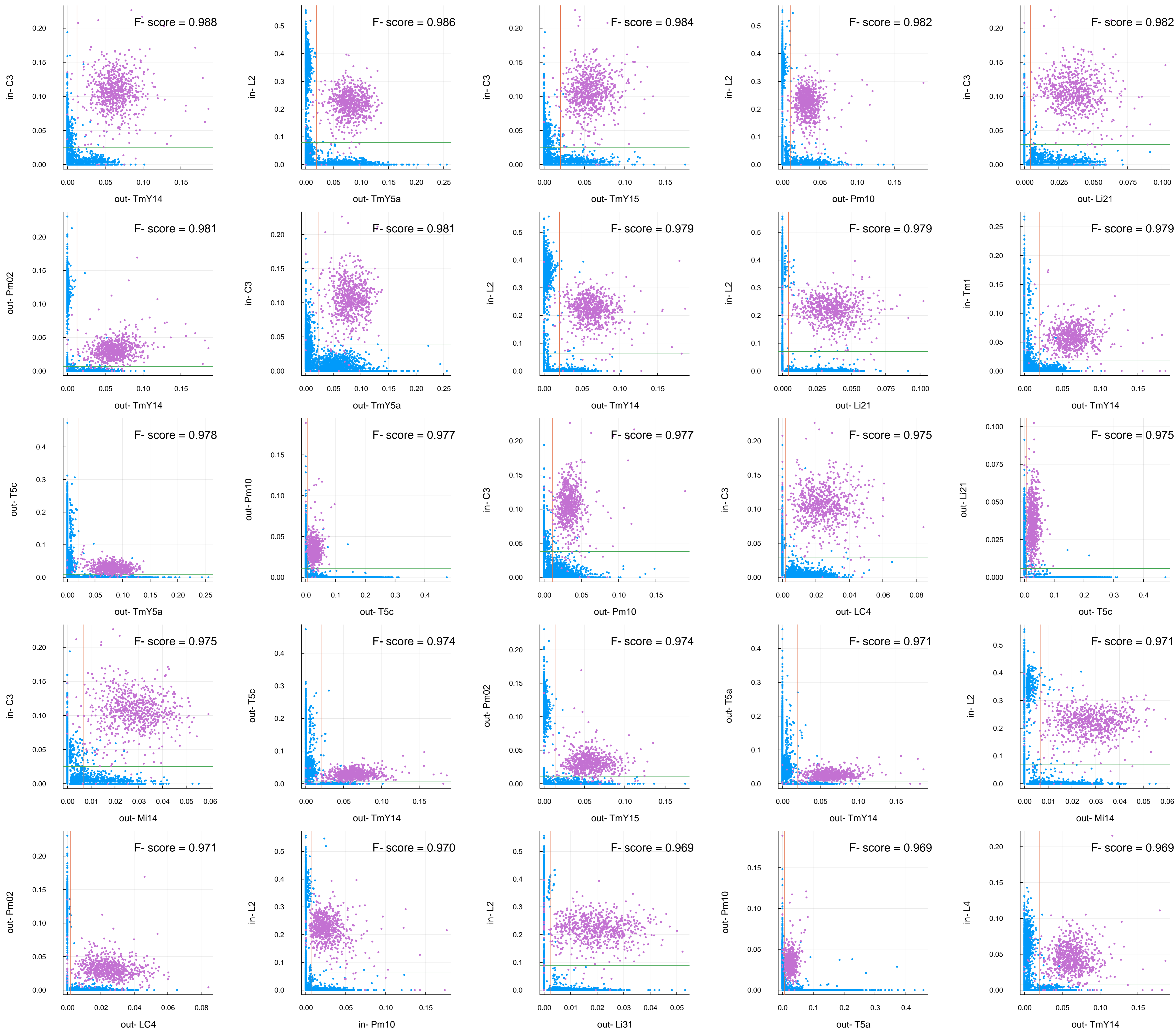

Supplement: Supplementary file 7 — Discriminating 2D projections for neuropil-intrinsic types. For each interneuron type, a pair of features is shown that can be used to discriminate that type from others in the same neuropil. Many although not all discriminations are highly accurate. Both intrinsic and boundary types are included as discriminative features. [file 41586_2024_7981_MOESM7_ESM.zip › DataS3/Tm4.pdf]

Tm5a

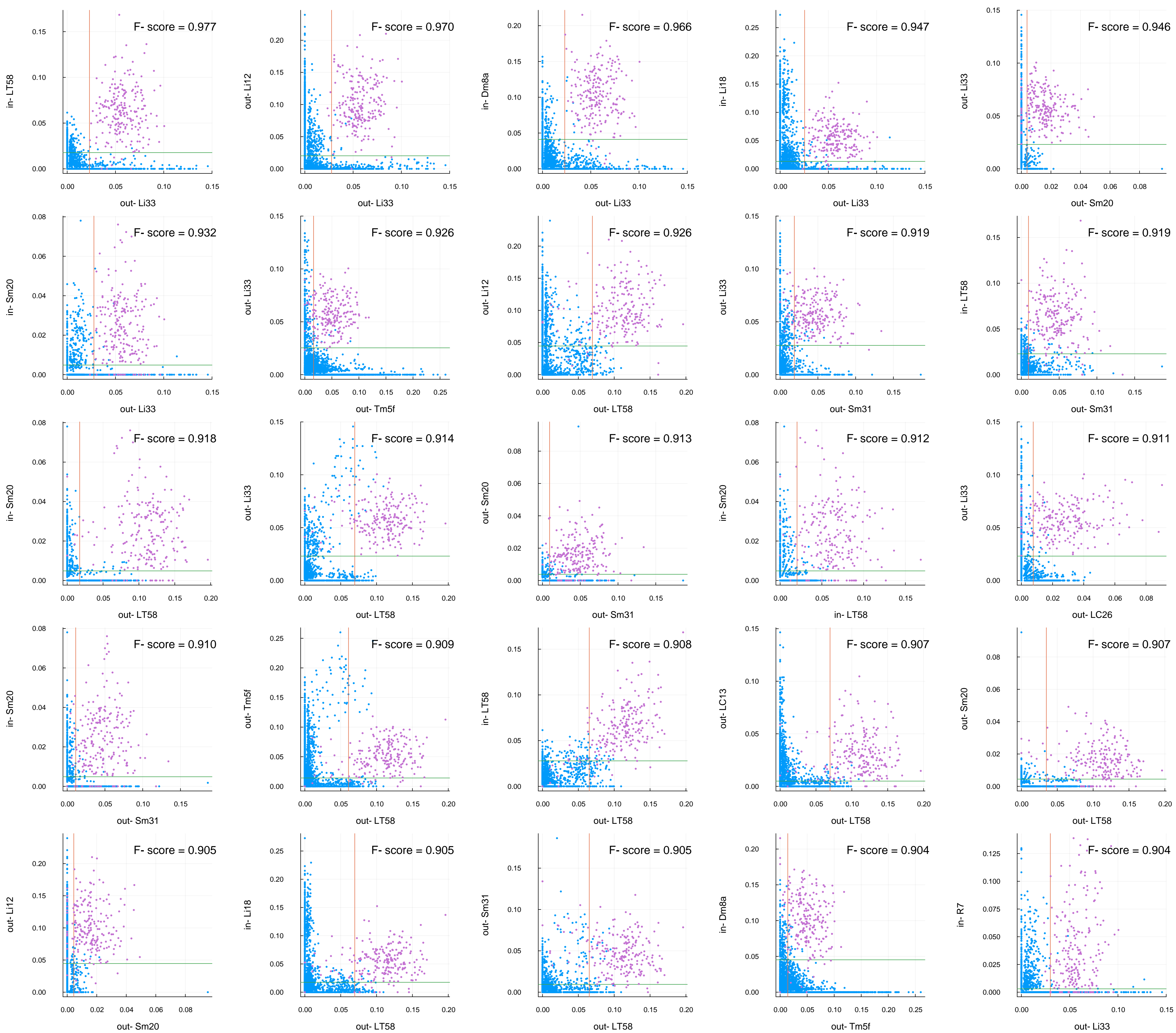

Supplement: Supplementary file 7 — Discriminating 2D projections for neuropil-intrinsic types. For each interneuron type, a pair of features is shown that can be used to discriminate that type from others in the same neuropil. Many although not all discriminations are highly accurate. Both intrinsic and boundary types are included as discriminative features. [file 41586_2024_7981_MOESM7_ESM.zip › DataS3/Tm5a.pdf]

Tm5b

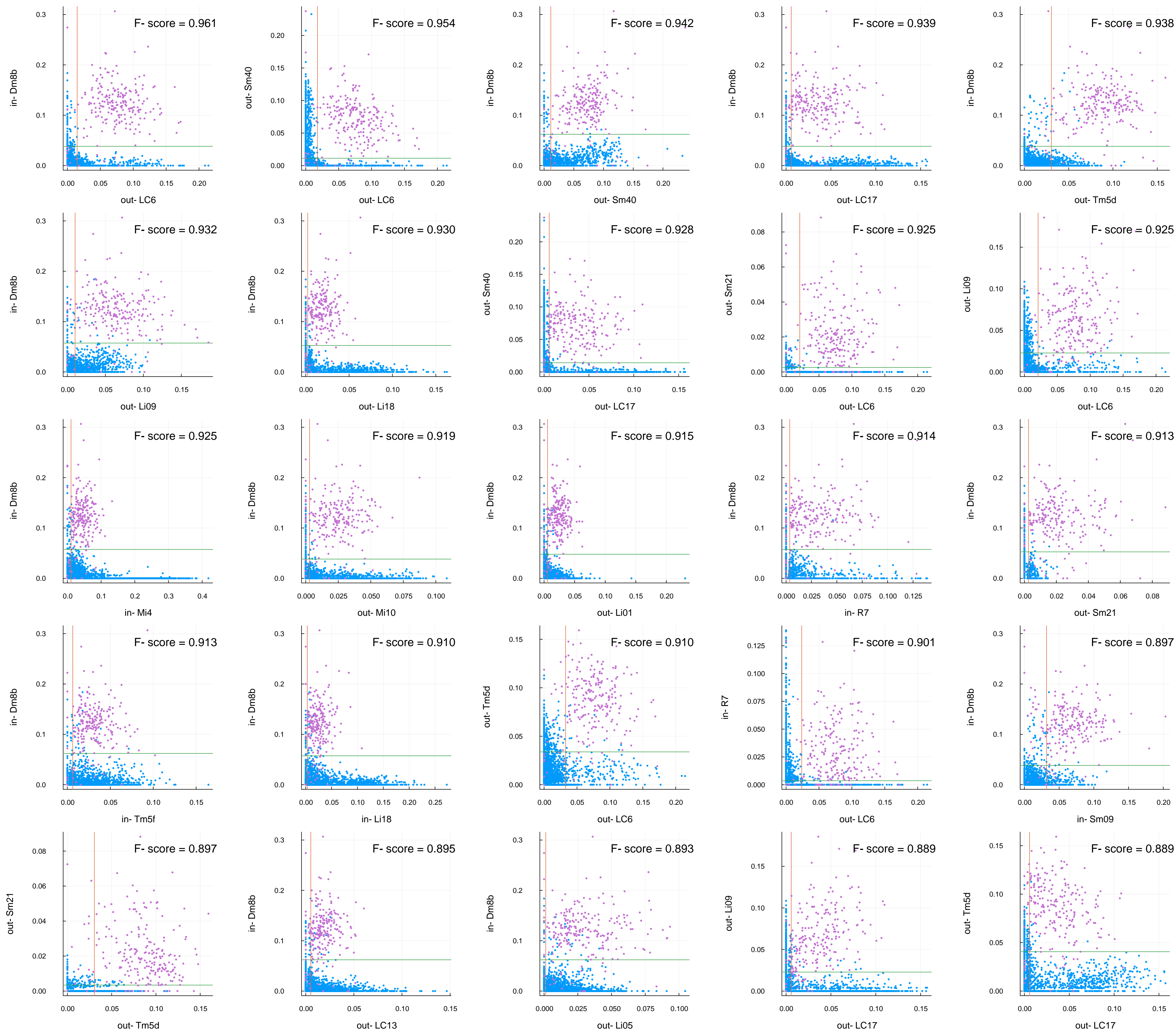

Supplement: Supplementary file 7 — Discriminating 2D projections for neuropil-intrinsic types. For each interneuron type, a pair of features is shown that can be used to discriminate that type from others in the same neuropil. Many although not all discriminations are highly accurate. Both intrinsic and boundary types are included as discriminative features. [file 41586_2024_7981_MOESM7_ESM.zip › DataS3/Tm5b.pdf]

Tm5c

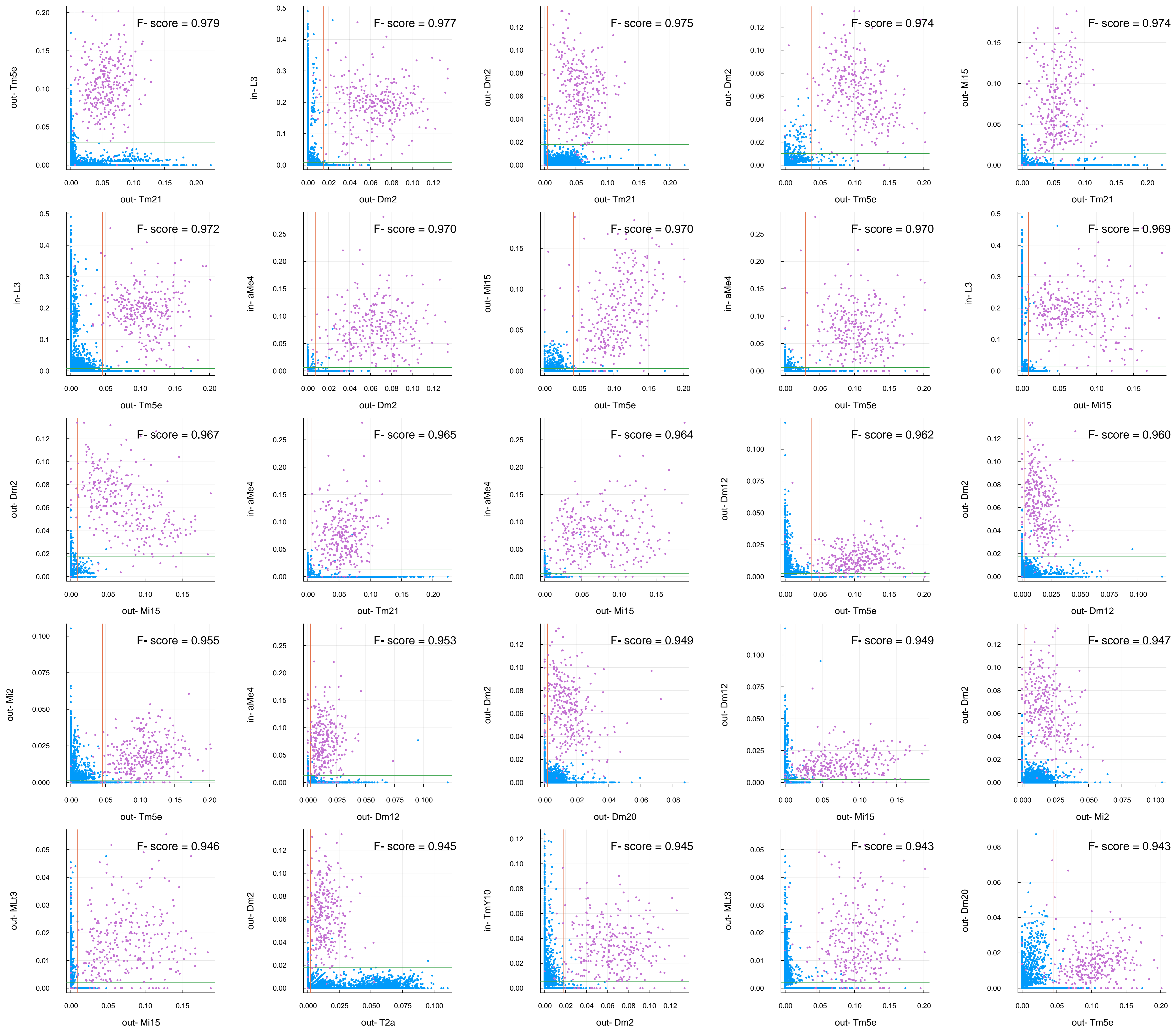

Supplement: Supplementary file 7 — Discriminating 2D projections for neuropil-intrinsic types. For each interneuron type, a pair of features is shown that can be used to discriminate that type from others in the same neuropil. Many although not all discriminations are highly accurate. Both intrinsic and boundary types are included as discriminative features. [file 41586_2024_7981_MOESM7_ESM.zip › DataS3/Tm5c.pdf]

Tm5d

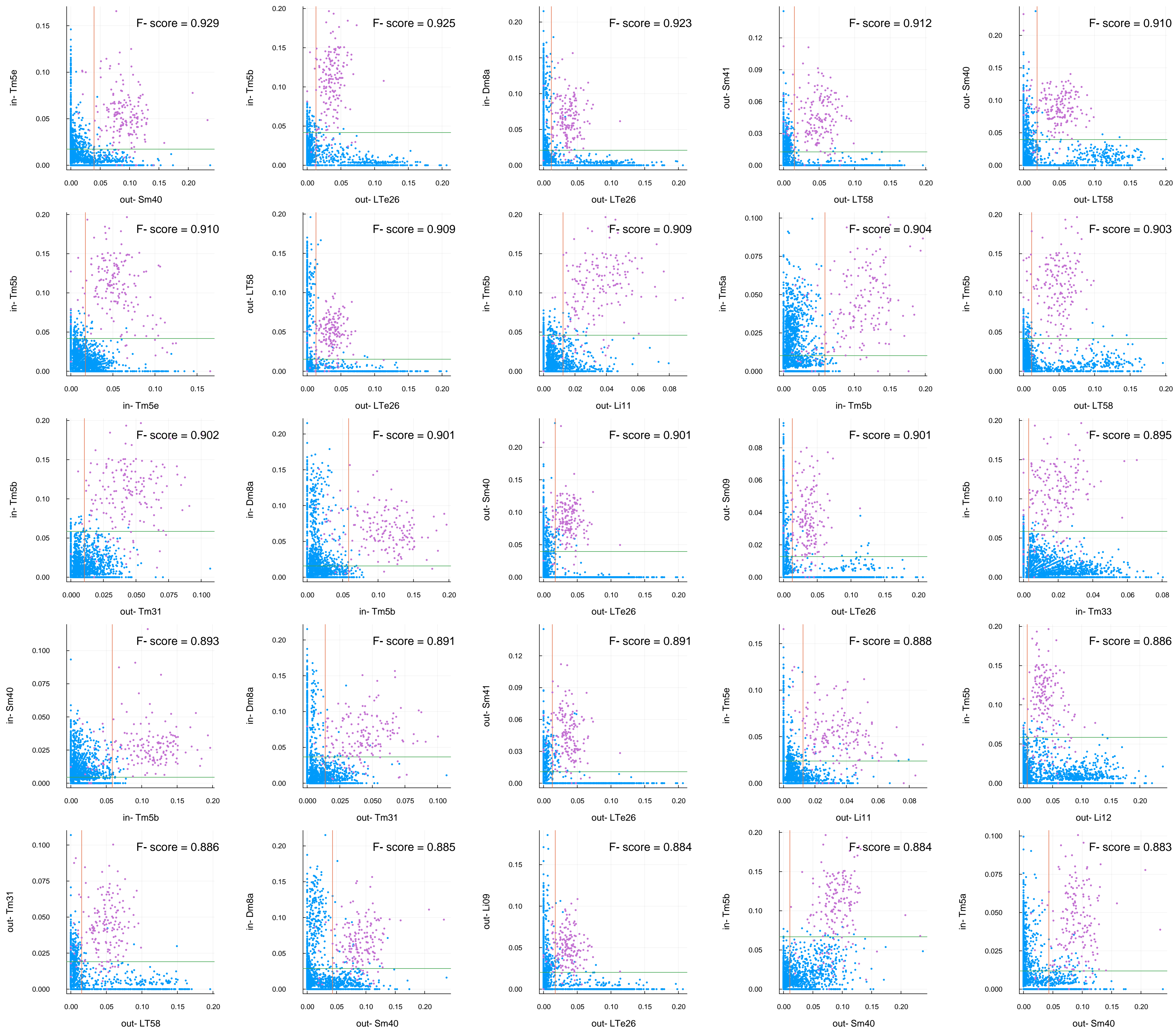

Supplement: Supplementary file 7 — Discriminating 2D projections for neuropil-intrinsic types. For each interneuron type, a pair of features is shown that can be used to discriminate that type from others in the same neuropil. Many although not all discriminations are highly accurate. Both intrinsic and boundary types are included as discriminative features. [file 41586_2024_7981_MOESM7_ESM.zip › DataS3/Tm5d.pdf]

## Tm5e

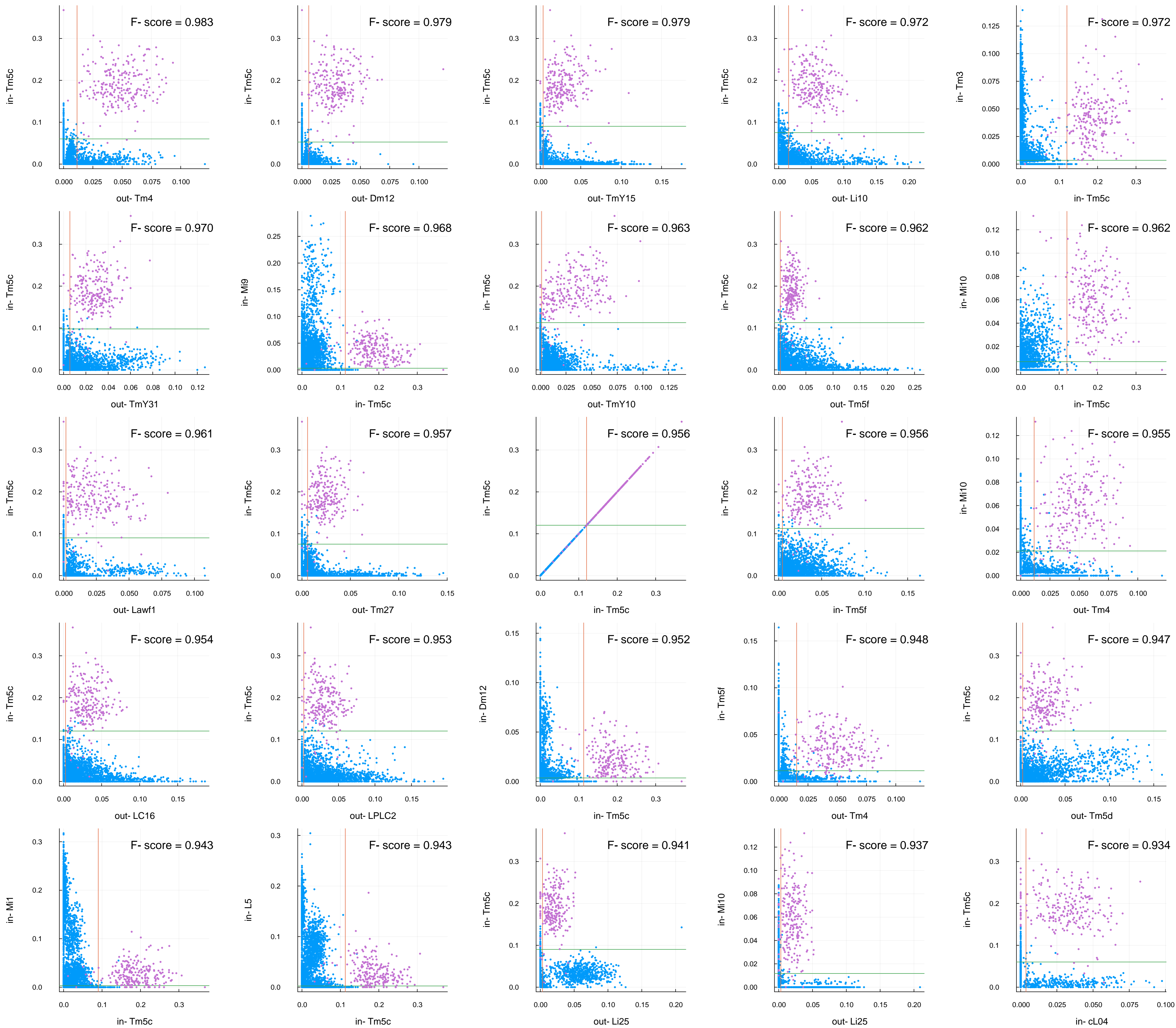

Supplement: Supplementary file 7 — Discriminating 2D projections for neuropil-intrinsic types. For each interneuron type, a pair of features is shown that can be used to discriminate that type from others in the same neuropil. Many although not all discriminations are highly accurate. Both intrinsic and boundary types are included as discriminative features. [file 41586_2024_7981_MOESM7_ESM.zip › DataS3/Tm5e.pdf]

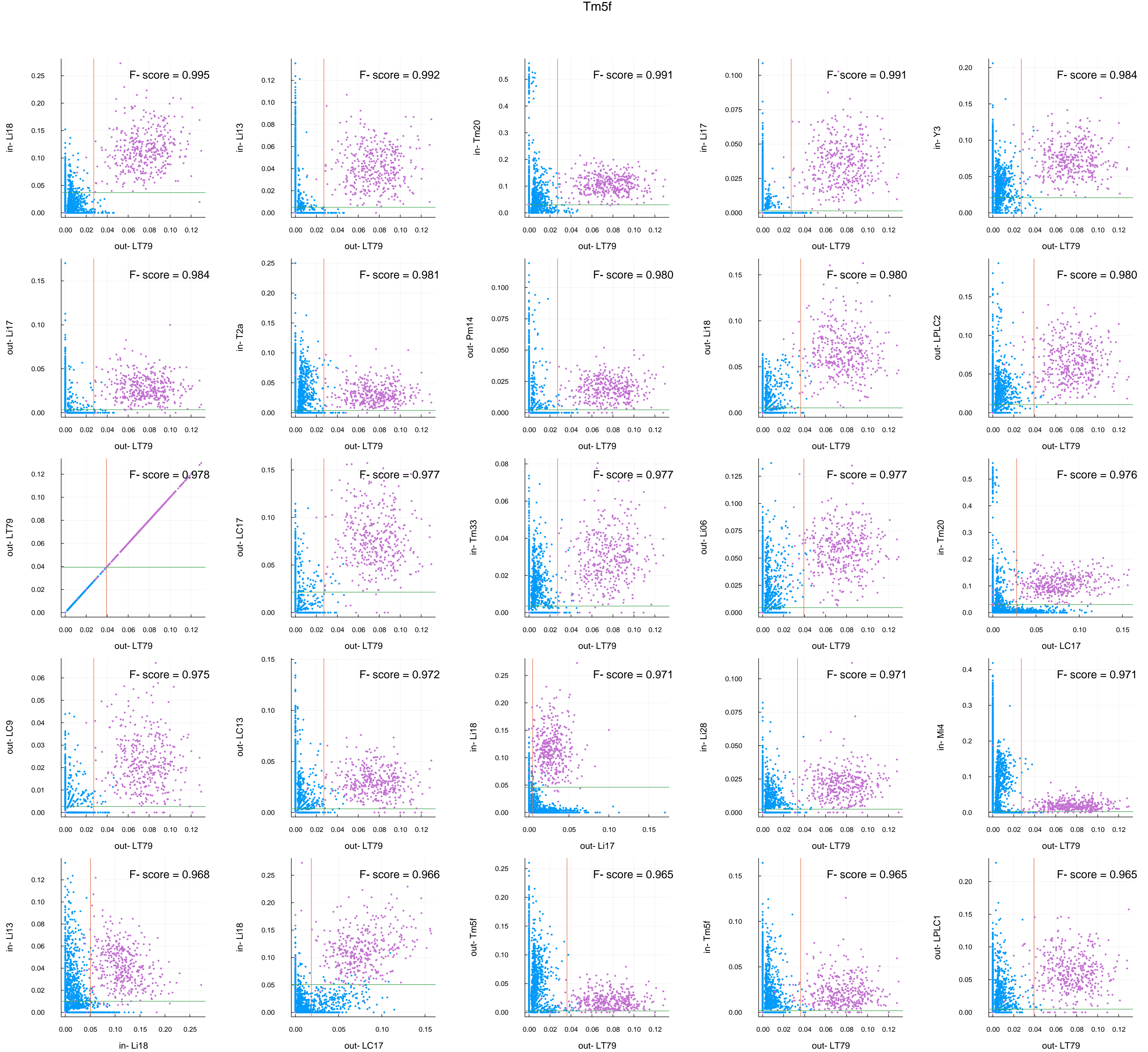

Supplement: Supplementary file 7 — Discriminating 2D projections for neuropil-intrinsic types. For each interneuron type, a pair of features is shown that can be used to discriminate that type from others in the same neuropil. Many although not all discriminations are highly accurate. Both intrinsic and boundary types are included as discriminative features. [file 41586_2024_7981_MOESM7_ESM.zip › DataS3/Tm5f.pdf]

Tm7

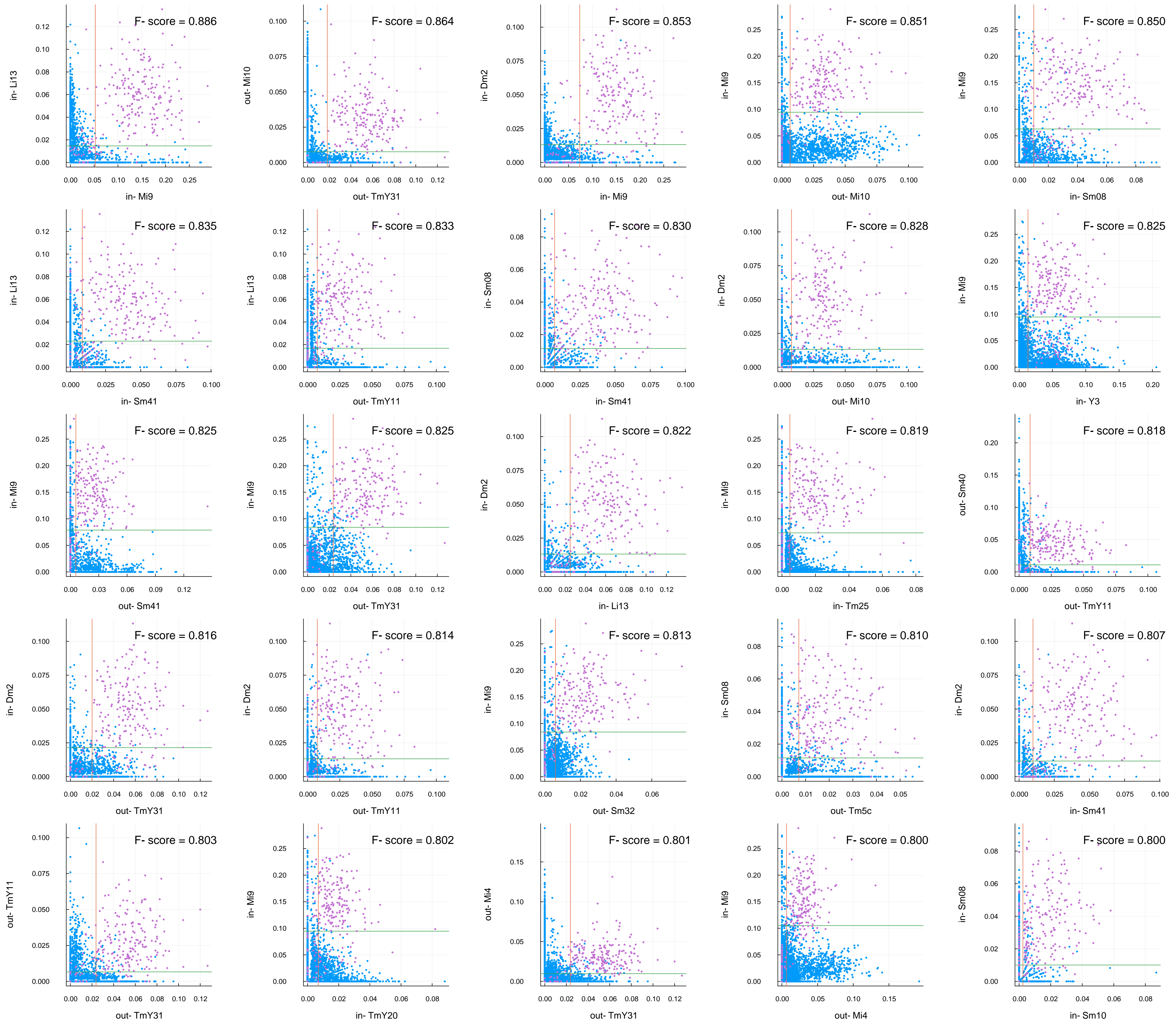

Supplement: Supplementary file 7 — Discriminating 2D projections for neuropil-intrinsic types. For each interneuron type, a pair of features is shown that can be used to discriminate that type from others in the same neuropil. Many although not all discriminations are highly accurate. Both intrinsic and boundary types are included as discriminative features. [file 41586_2024_7981_MOESM7_ESM.zip › DataS3/Tm7.pdf]

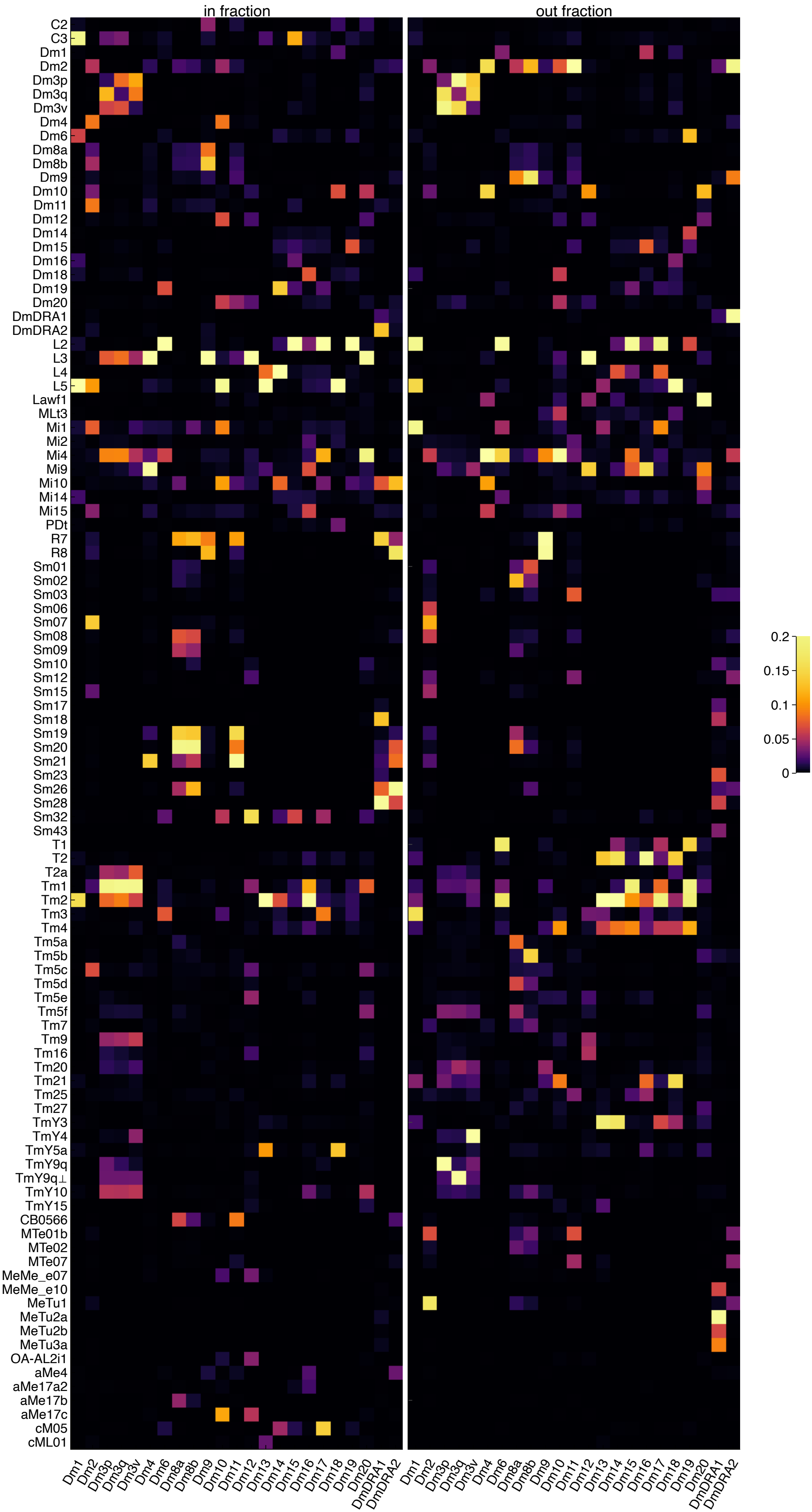

Supplement: Supplementary file 8 — Input and output fractions for cell types. Heat map columns show input/output fractions per cell type. Rows include partners contributing ≥0.02 input or output fraction to any reference type in the column. Heat maps display boundary and intrinsic types, with colour capped at 0.2. Be cautious of normalization artifacts; few synapses may falsely suggest strong connections. [file 41586_2024_7981_MOESM8_ESM.zip › DataS4/Dm.pdf]

in fraction

out fraction

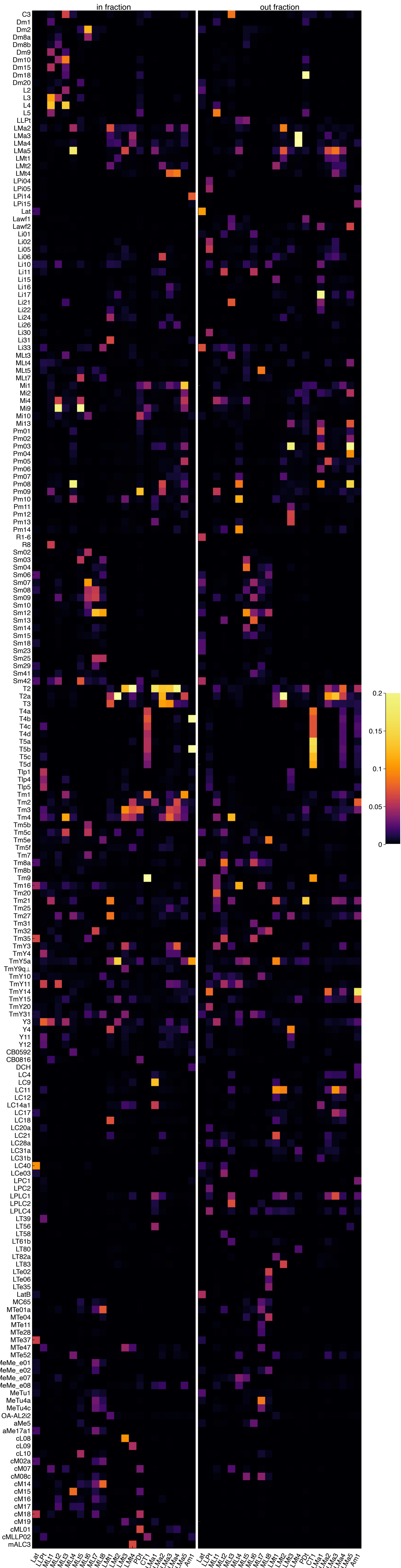

Supplement: Supplementary file 8 — Input and output fractions for cell types. Heat map columns show input/output fractions per cell type. Rows include partners contributing ≥0.02 input or output fraction to any reference type in the column. Heat maps display boundary and intrinsic types, with colour capped at 0.2. Be cautious of normalization artifacts; few synapses may falsely suggest strong connections. [file 41586_2024_7981_MOESM8_ESM.zip › DataS4/LatLLPtMLtLMtPDtLMaMLLPa.pdf]

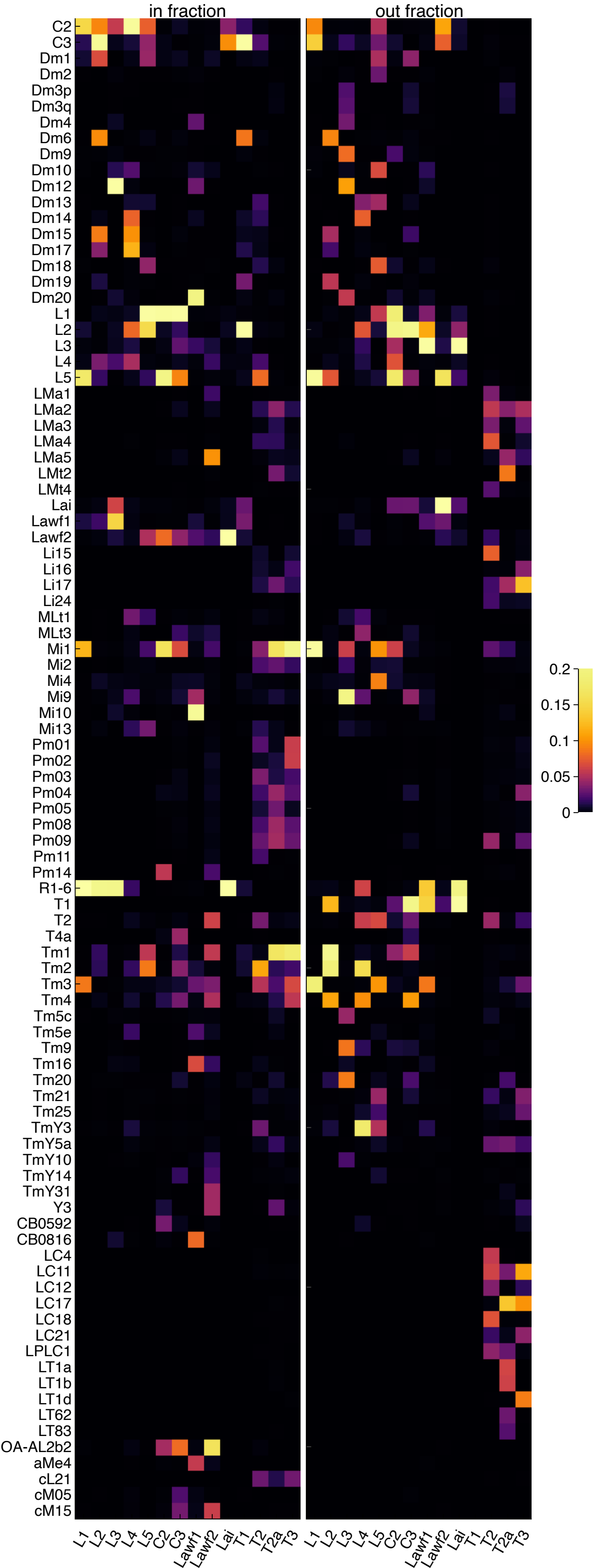

Supplement: Supplementary file 8 — Input and output fractions for cell types. Heat map columns show input/output fractions per cell type. Rows include partners contributing ≥0.02 input or output fraction to any reference type in the column. Heat maps display boundary and intrinsic types, with colour capped at 0.2. Be cautious of normalization artifacts; few synapses may falsely suggest strong connections. [file 41586_2024_7981_MOESM8_ESM.zip › DataS4/LCLawfLaiT1T2T3.pdf]

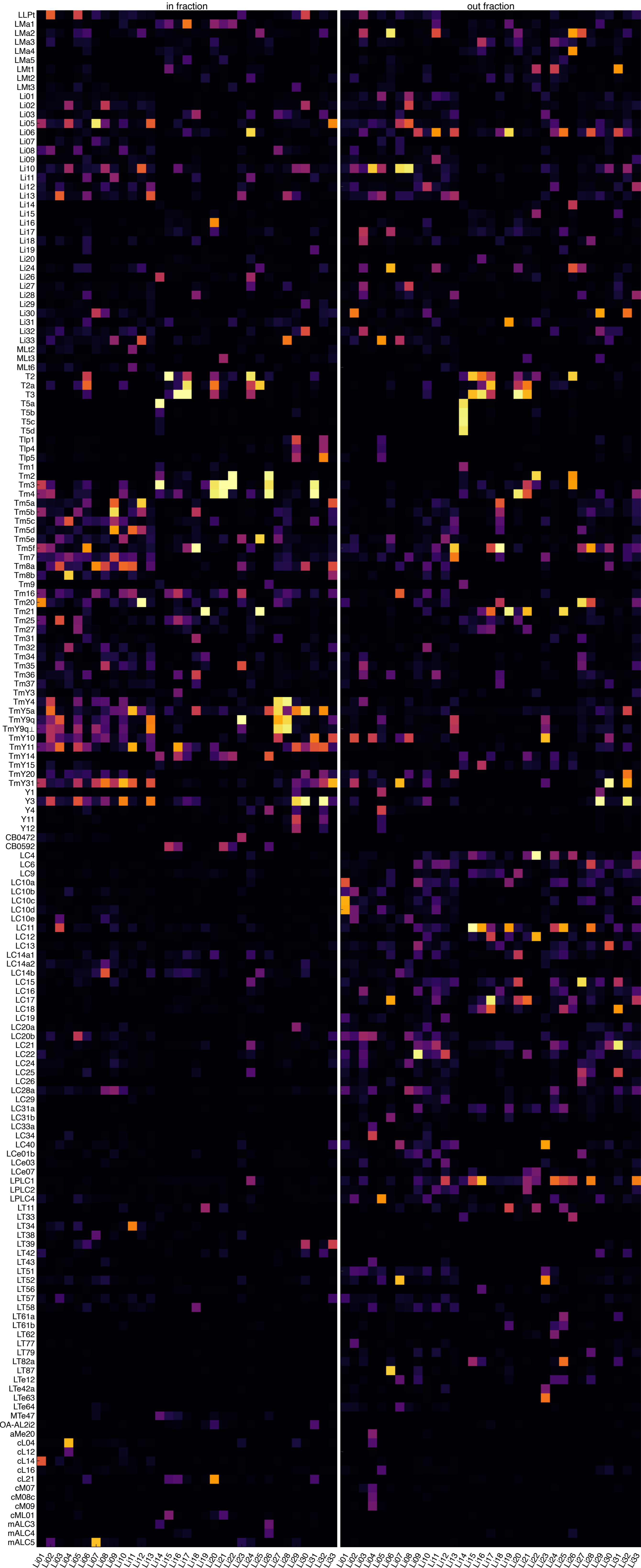

Supplement: Supplementary file 8 — Input and output fractions for cell types. Heat map columns show input/output fractions per cell type. Rows include partners contributing ≥0.02 input or output fraction to any reference type in the column. Heat maps display boundary and intrinsic types, with colour capped at 0.2. Be cautious of normalization artifacts; few synapses may falsely suggest strong connections. [file 41586_2024_7981_MOESM8_ESM.zip › DataS4/Li.pdf]

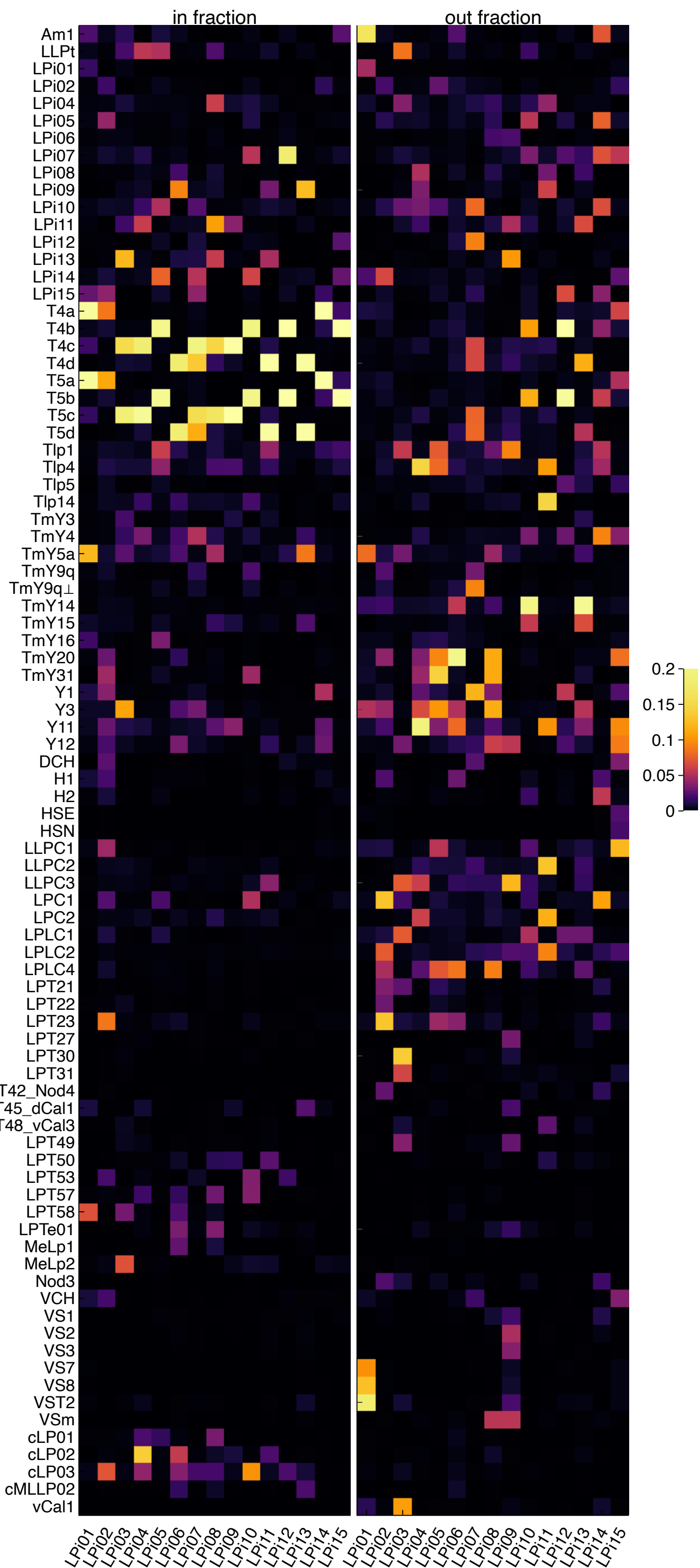

Supplement: Supplementary file 8 — Input and output fractions for cell types. Heat map columns show input/output fractions per cell type. Rows include partners contributing ≥0.02 input or output fraction to any reference type in the column. Heat maps display boundary and intrinsic types, with colour capped at 0.2. Be cautious of normalization artifacts; few synapses may falsely suggest strong connections. [file 41586_2024_7981_MOESM8_ESM.zip › DataS4/LPi.pdf]

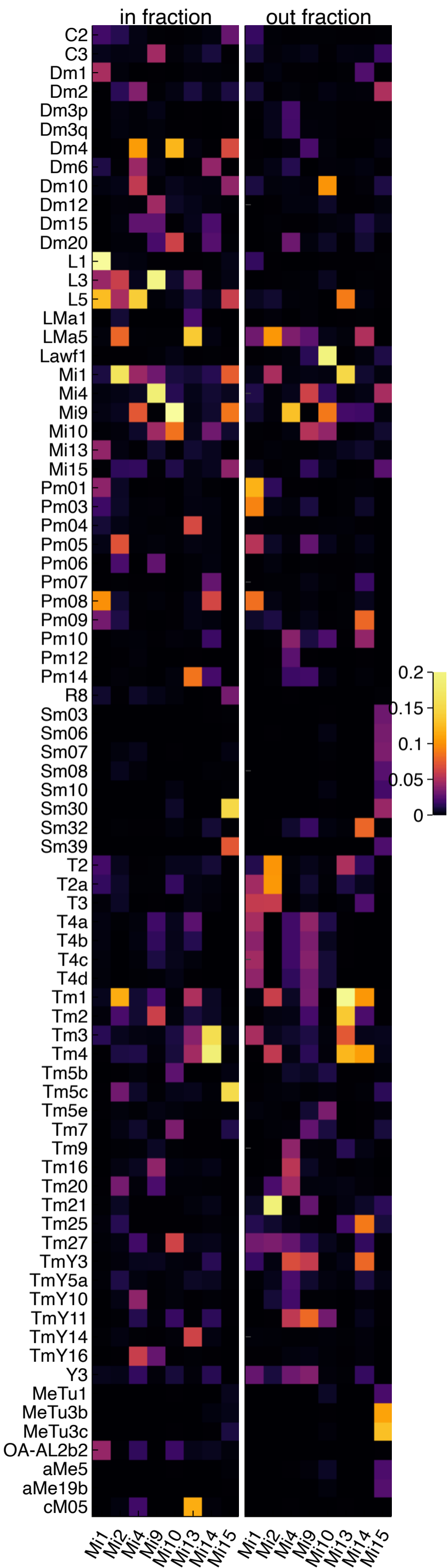

Supplement: Supplementary file 8 — Input and output fractions for cell types. Heat map columns show input/output fractions per cell type. Rows include partners contributing ≥0.02 input or output fraction to any reference type in the column. Heat maps display boundary and intrinsic types, with colour capped at 0.2. Be cautious of normalization artifacts; few synapses may falsely suggest strong connections. [file 41586_2024_7981_MOESM8_ESM.zip › DataS4/Mi.pdf]

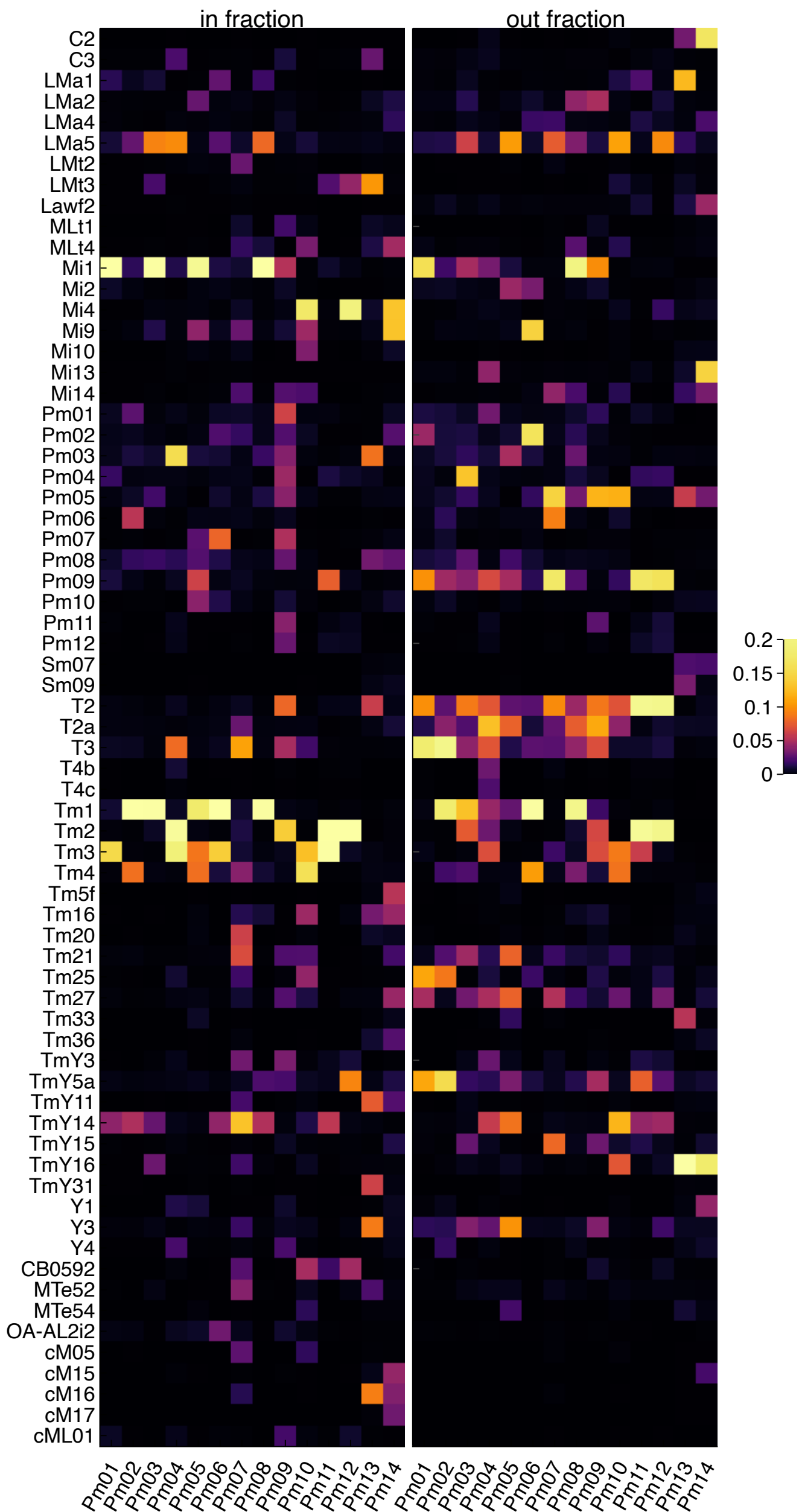

Supplement: Supplementary file 8 — Input and output fractions for cell types. Heat map columns show input/output fractions per cell type. Rows include partners contributing ≥0.02 input or output fraction to any reference type in the column. Heat maps display boundary and intrinsic types, with colour capped at 0.2. Be cautious of normalization artifacts; few synapses may falsely suggest strong connections. [file 41586_2024_7981_MOESM8_ESM.zip › DataS4/Pm.pdf]

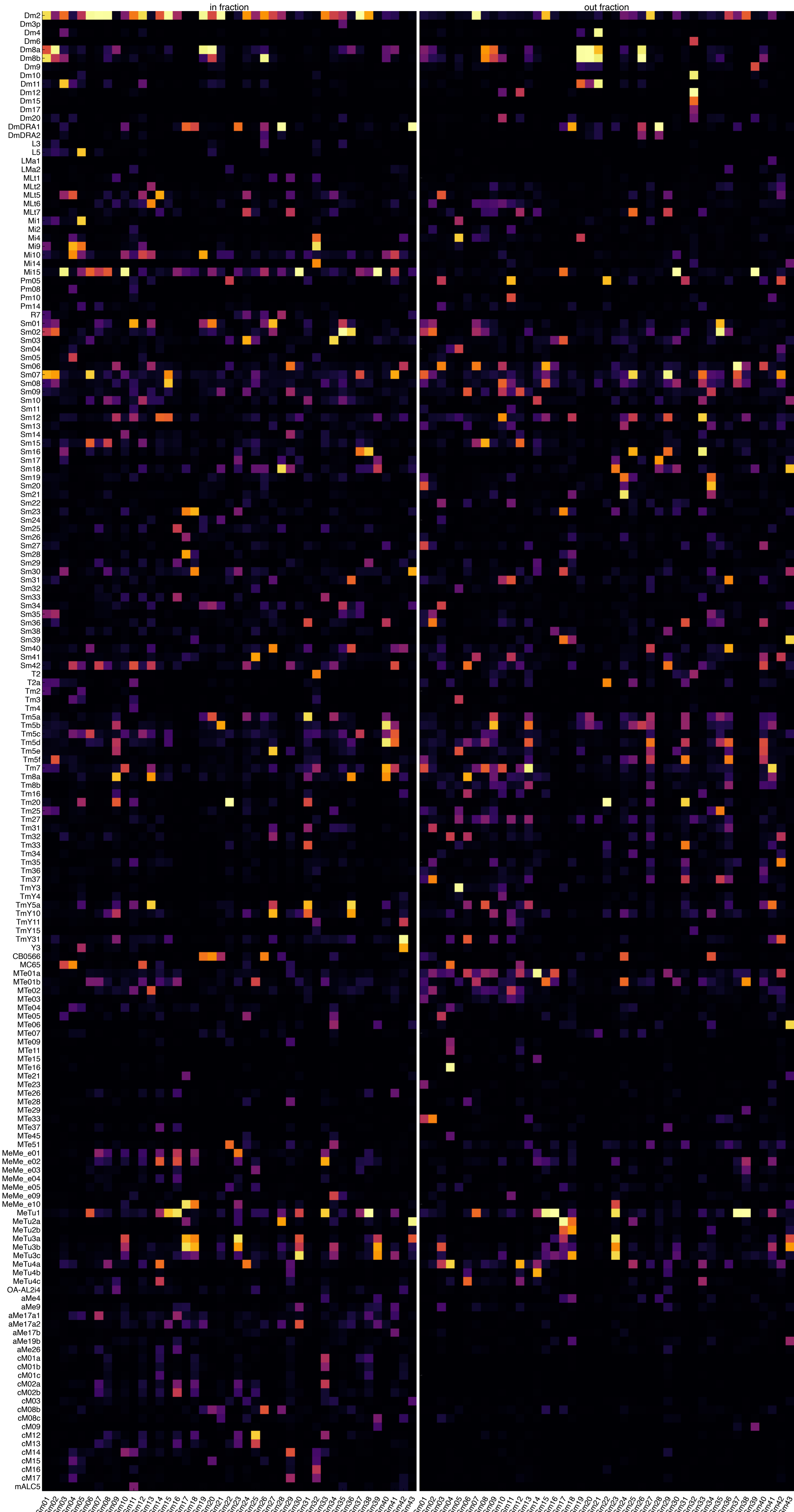

Supplement: Supplementary file 8 — Input and output fractions for cell types. Heat map columns show input/output fractions per cell type. Rows include partners contributing ≥0.02 input or output fraction to any reference type in the column. Heat maps display boundary and intrinsic types, with colour capped at 0.2. Be cautious of normalization artifacts; few synapses may falsely suggest strong connections. [file 41586_2024_7981_MOESM8_ESM.zip › DataS4/Sm.pdf]

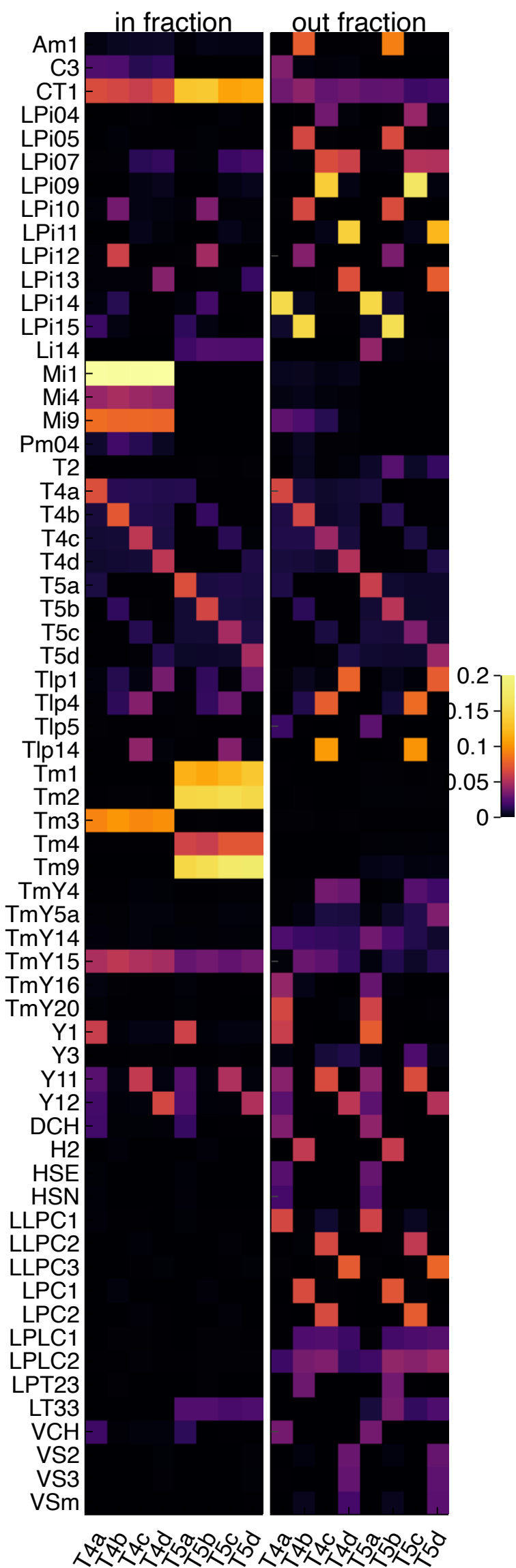

Supplement: Supplementary file 8 — Input and output fractions for cell types. Heat map columns show input/output fractions per cell type. Rows include partners contributing ≥0.02 input or output fraction to any reference type in the column. Heat maps display boundary and intrinsic types, with colour capped at 0.2. Be cautious of normalization artifacts; few synapses may falsely suggest strong connections. [file 41586_2024_7981_MOESM8_ESM.zip › DataS4/T4T5.pdf]

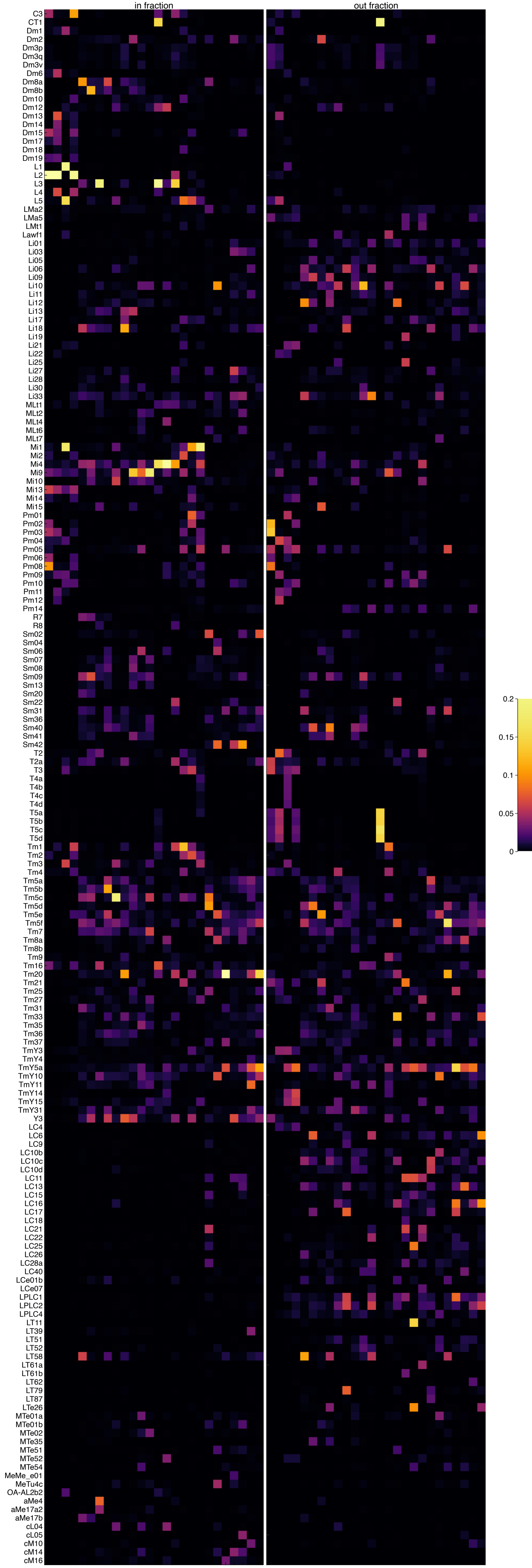

Supplement: Supplementary file 8 — Input and output fractions for cell types. Heat map columns show input/output fractions per cell type. Rows include partners contributing ≥0.02 input or output fraction to any reference type in the column. Heat maps display boundary and intrinsic types, with colour capped at 0.2. Be cautious of normalization artifacts; few synapses may falsely suggest strong connections. [file 41586_2024_7981_MOESM8_ESM.zip › DataS4/Tm.pdf]

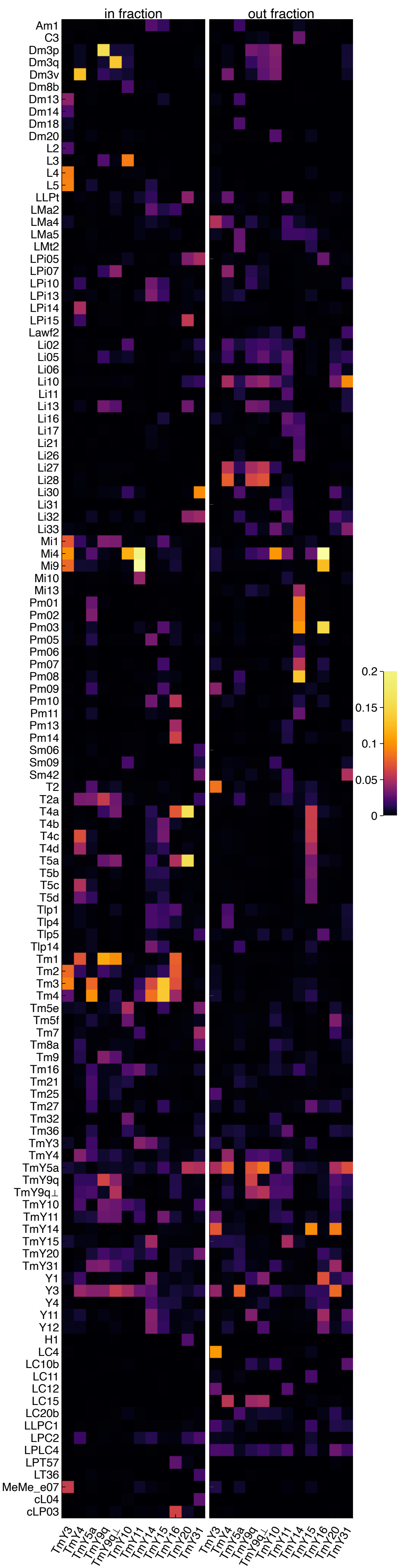

Supplement: Supplementary file 8 — Input and output fractions for cell types. Heat map columns show input/output fractions per cell type. Rows include partners contributing ≥0.02 input or output fraction to any reference type in the column. Heat maps display boundary and intrinsic types, with colour capped at 0.2. Be cautious of normalization artifacts; few synapses may falsely suggest strong connections. [file 41586_2024_7981_MOESM8_ESM.zip › DataS4/TmY.pdf]

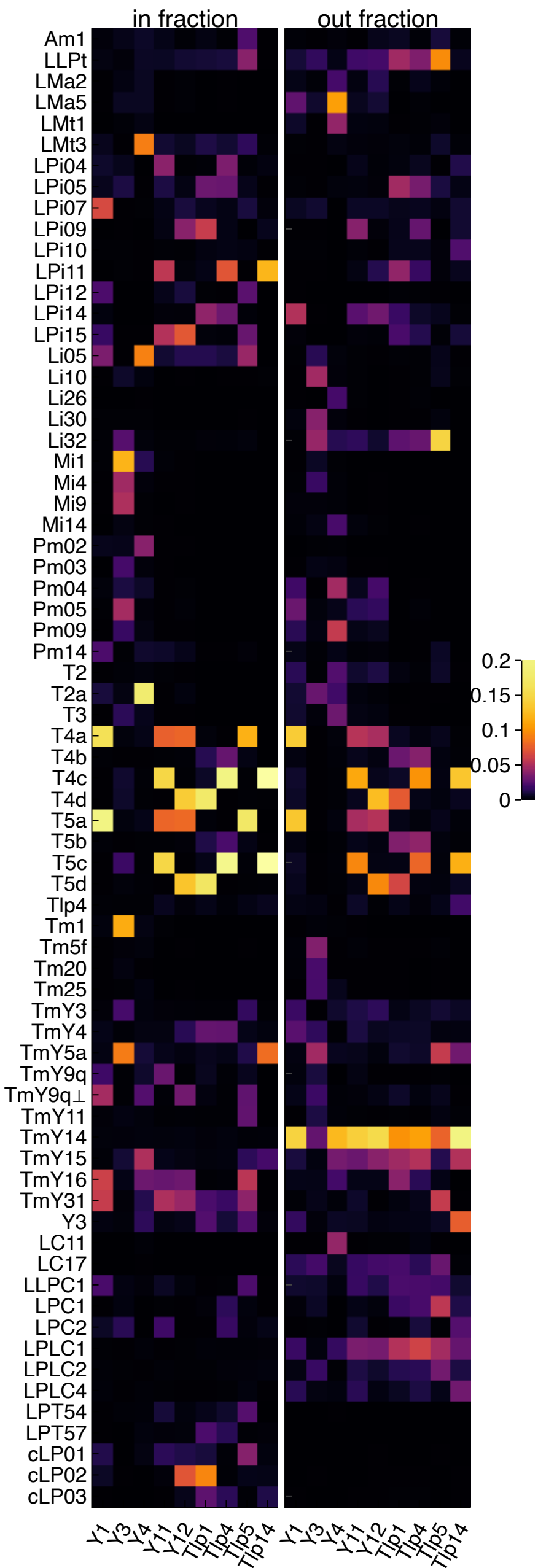

Supplement: Supplementary file 8 — Input and output fractions for cell types. Heat map columns show input/output fractions per cell type. Rows include partners contributing ≥0.02 input or output fraction to any reference type in the column. Heat maps display boundary and intrinsic types, with colour capped at 0.2. Be cautious of normalization artifacts; few synapses may falsely suggest strong connections. [file 41586_2024_7981_MOESM8_ESM.zip › DataS4/YTlp.pdf]
