# Supplementary figures and images for: Neuronal parts list and wiring diagram for a visual system (part 2 of 3)
Source: Nature. 2024 Oct 2;634(8032):166–80. doi: 10.1038/s41586-024-07981-1 (PMC11446827; doi:10.1038/s41586-024-07981-1)

Li10

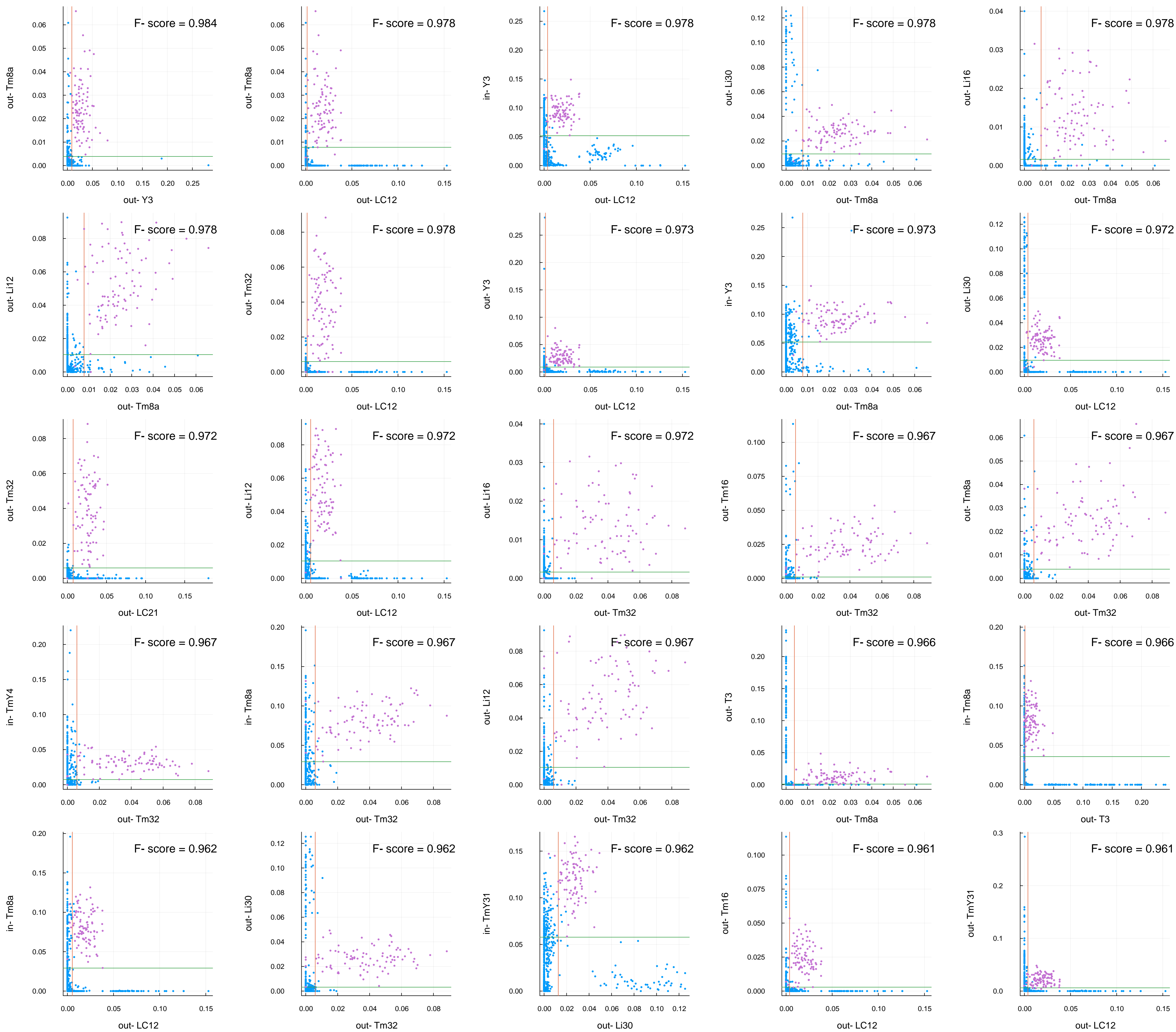

Supplement: Supplementary file 7 — Discriminating 2D projections for neuropil-intrinsic types. For each interneuron type, a pair of features is shown that can be used to discriminate that type from others in the same neuropil. Many although not all discriminations are highly accurate. Both intrinsic and boundary types are included as discriminative features. [file 41586_2024_7981_MOESM7_ESM.zip › DataS3/Li10.pdf]

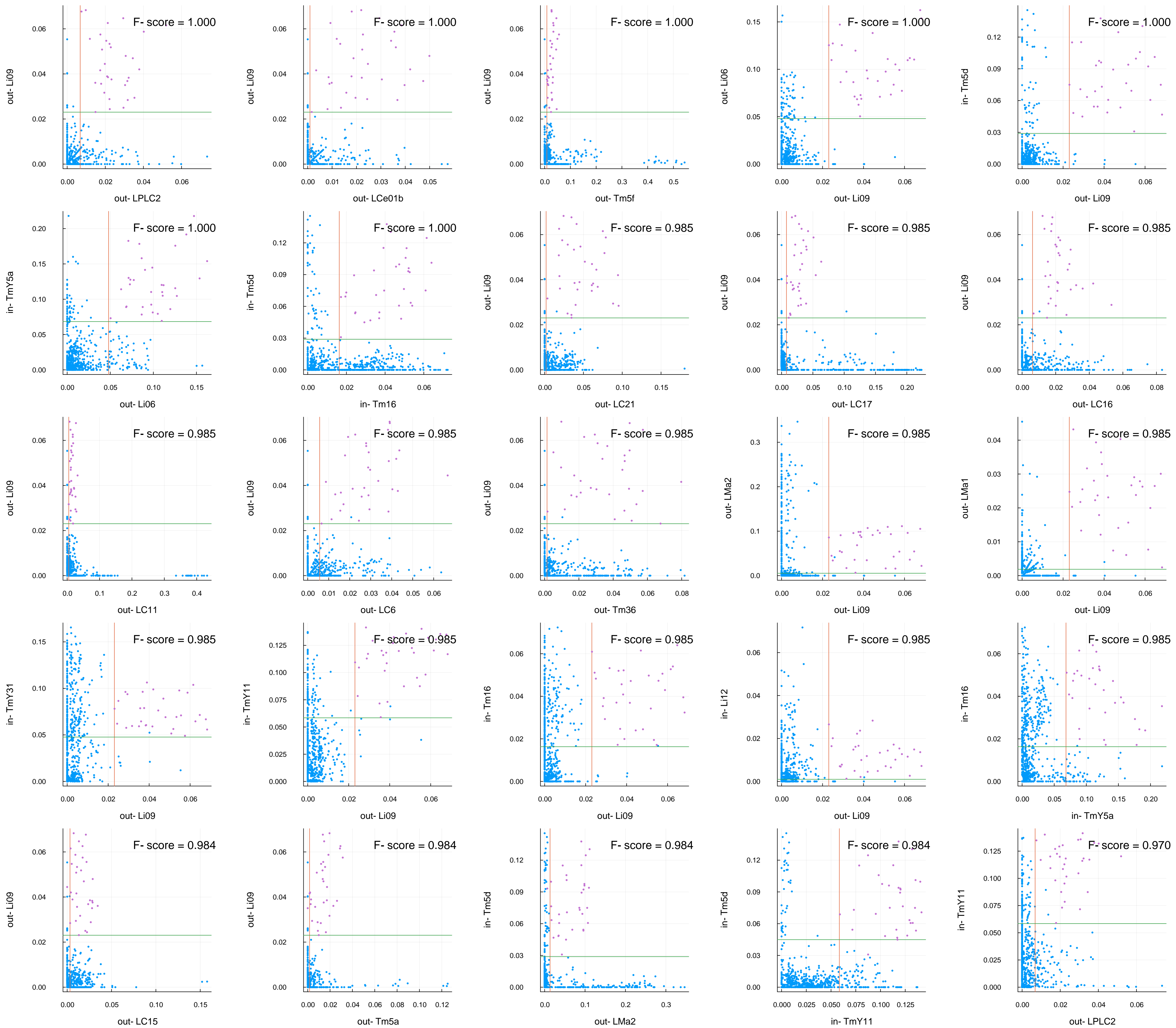

Supplement: Supplementary file 7 — Discriminating 2D projections for neuropil-intrinsic types. For each interneuron type, a pair of features is shown that can be used to discriminate that type from others in the same neuropil. Many although not all discriminations are highly accurate. Both intrinsic and boundary types are included as discriminative features. [file 41586_2024_7981_MOESM7_ESM.zip › DataS3/Li11.pdf]

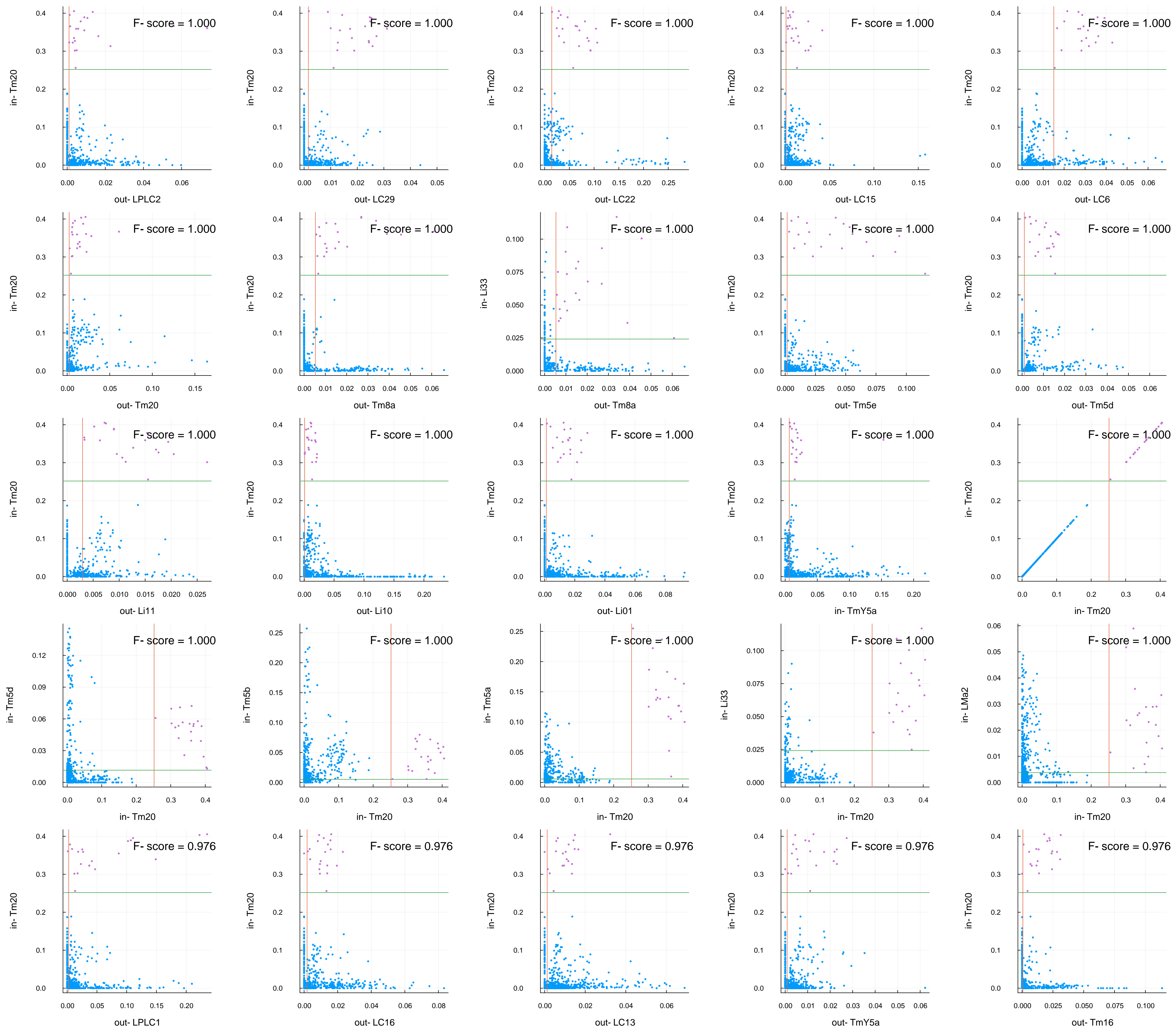

Supplement: Supplementary file 7 — Discriminating 2D projections for neuropil-intrinsic types. For each interneuron type, a pair of features is shown that can be used to discriminate that type from others in the same neuropil. Many although not all discriminations are highly accurate. Both intrinsic and boundary types are included as discriminative features. [file 41586_2024_7981_MOESM7_ESM.zip › DataS3/Li12.pdf]

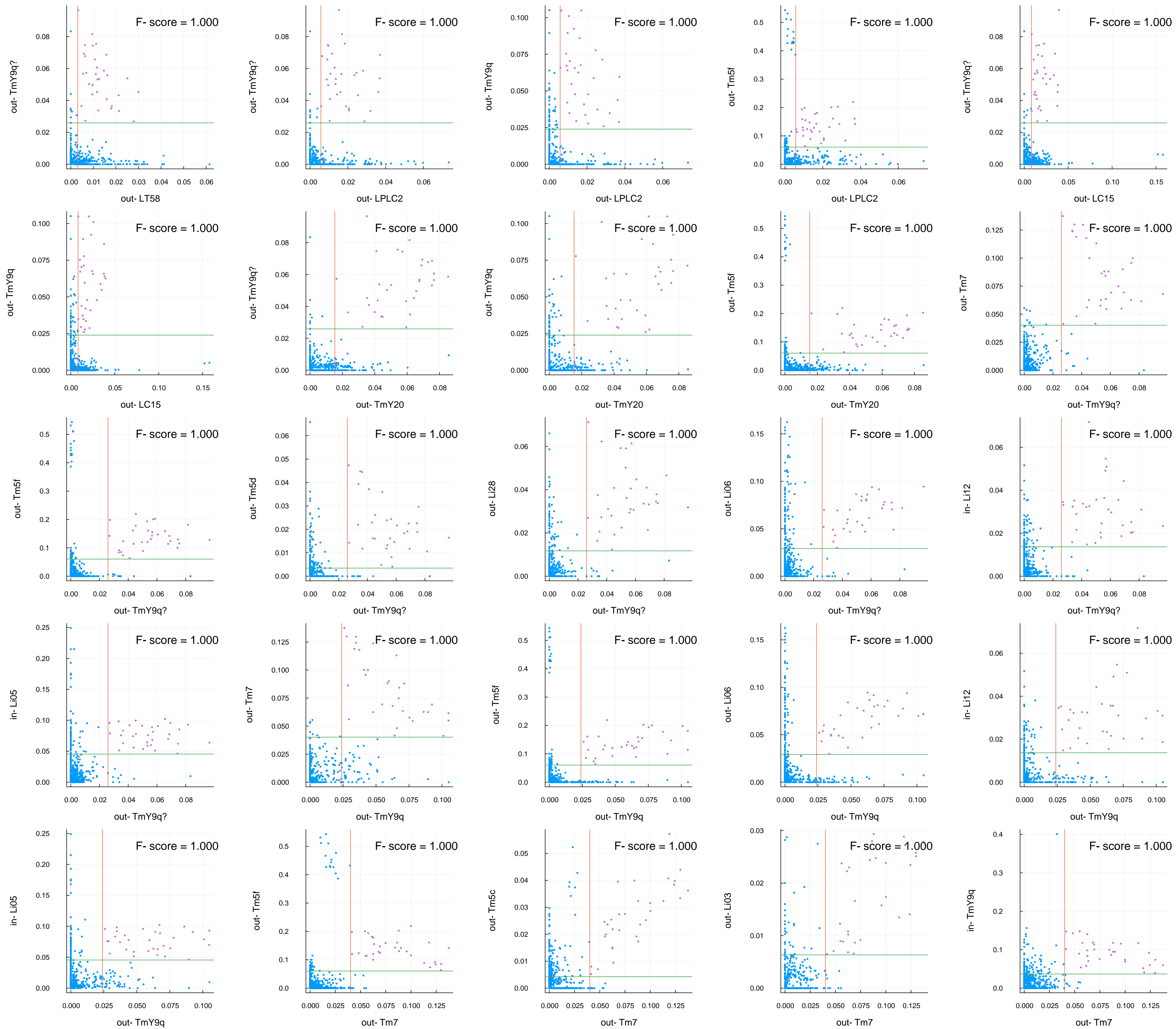

Supplement: Supplementary file 7 — Discriminating 2D projections for neuropil-intrinsic types. For each interneuron type, a pair of features is shown that can be used to discriminate that type from others in the same neuropil. Many although not all discriminations are highly accurate. Both intrinsic and boundary types are included as discriminative features. [file 41586_2024_7981_MOESM7_ESM.zip › DataS3/Li13.pdf]

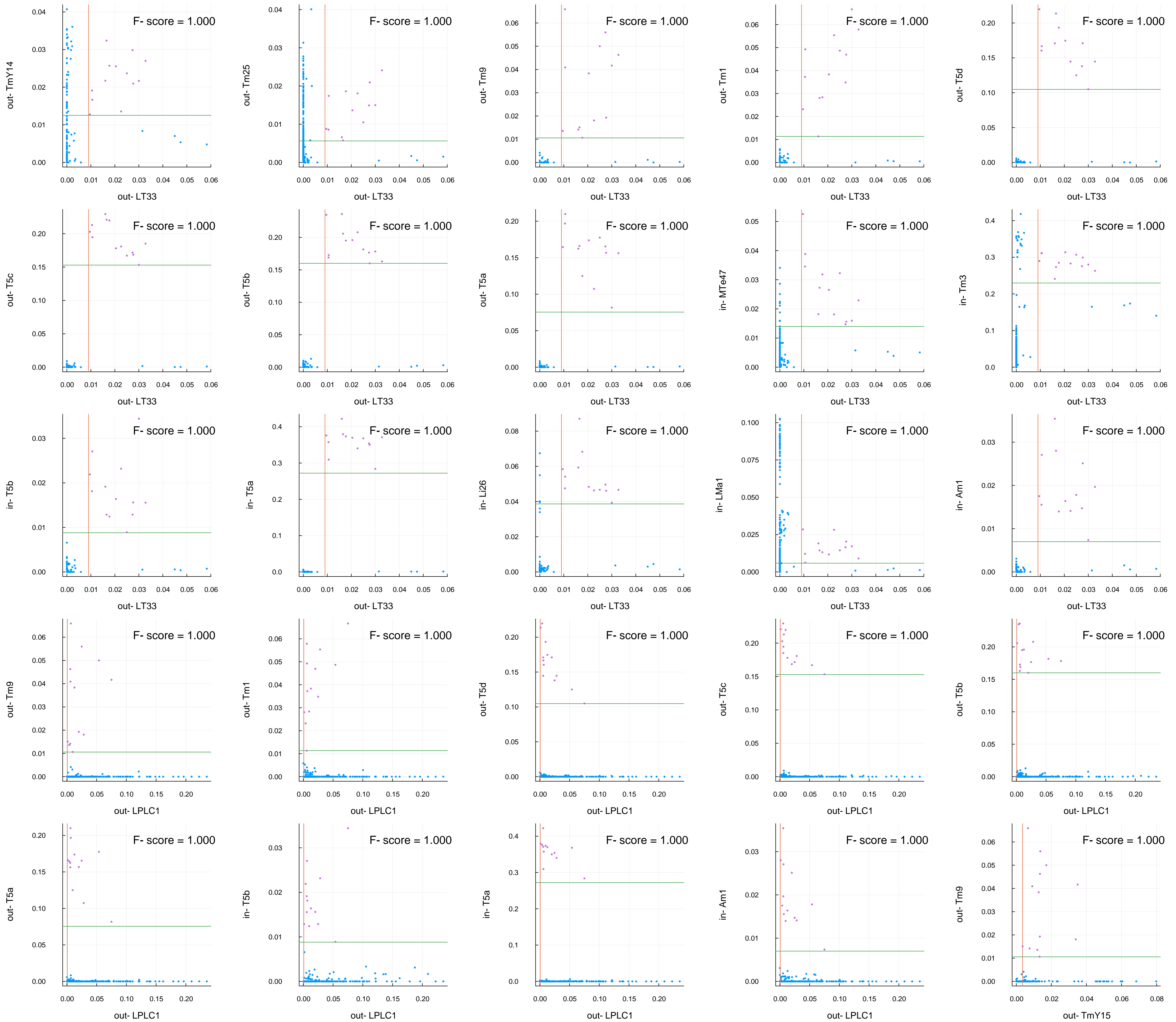

Supplement: Supplementary file 7 — Discriminating 2D projections for neuropil-intrinsic types. For each interneuron type, a pair of features is shown that can be used to discriminate that type from others in the same neuropil. Many although not all discriminations are highly accurate. Both intrinsic and boundary types are included as discriminative features. [file 41586_2024_7981_MOESM7_ESM.zip › DataS3/Li14.pdf]

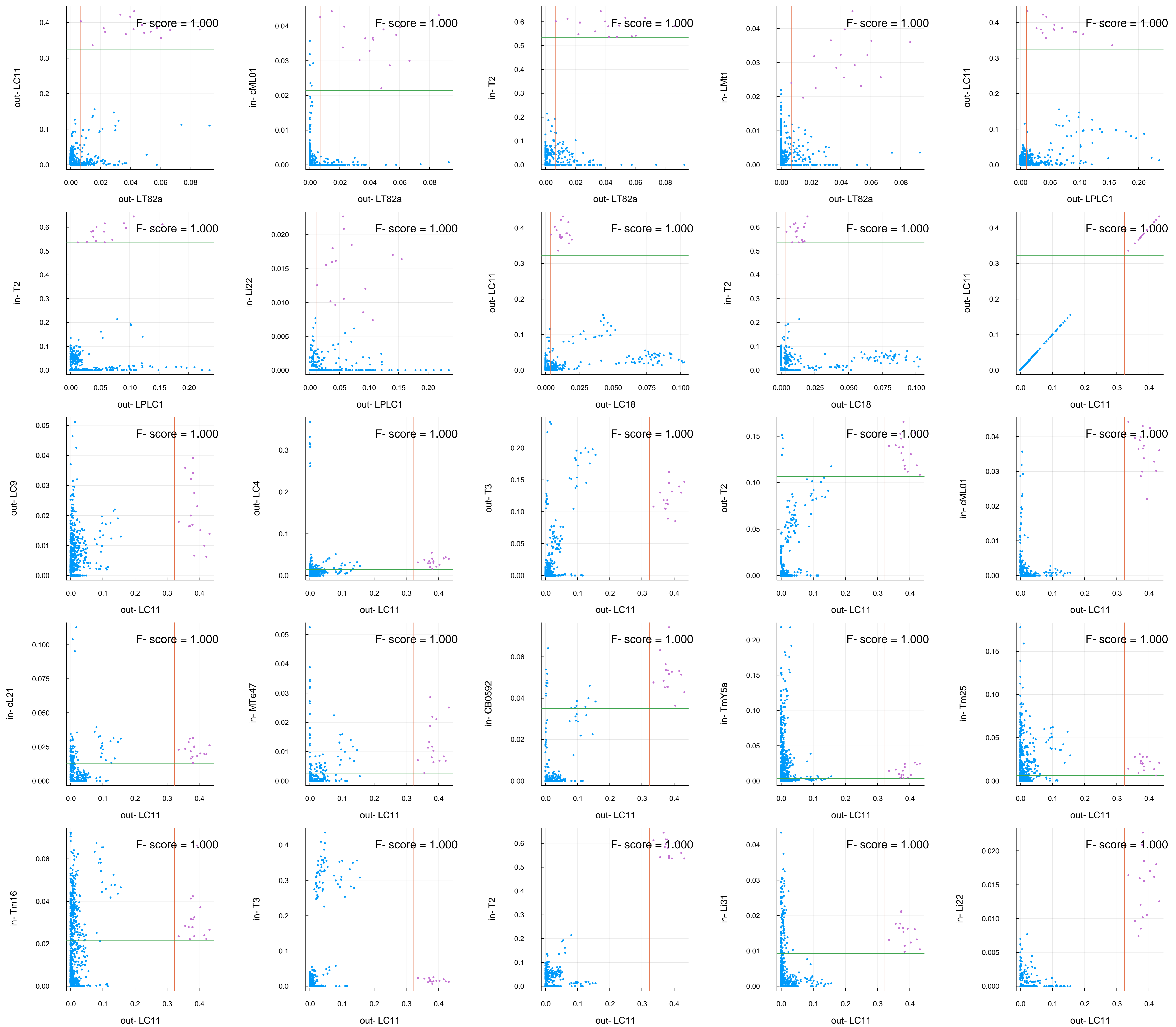

Supplement: Supplementary file 7 — Discriminating 2D projections for neuropil-intrinsic types. For each interneuron type, a pair of features is shown that can be used to discriminate that type from others in the same neuropil. Many although not all discriminations are highly accurate. Both intrinsic and boundary types are included as discriminative features. [file 41586_2024_7981_MOESM7_ESM.zip › DataS3/Li15.pdf]

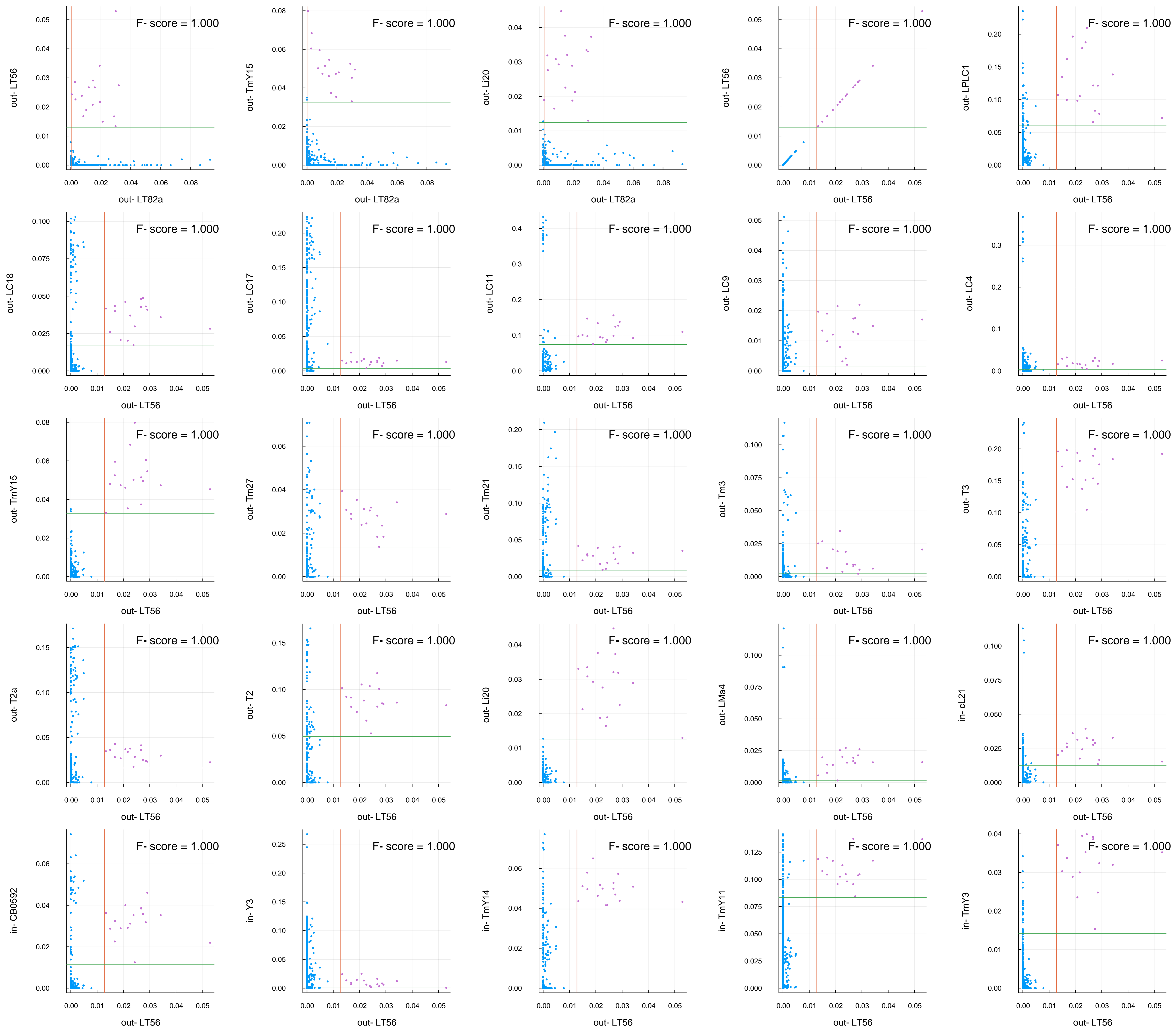

Supplement: Supplementary file 7 — Discriminating 2D projections for neuropil-intrinsic types. For each interneuron type, a pair of features is shown that can be used to discriminate that type from others in the same neuropil. Many although not all discriminations are highly accurate. Both intrinsic and boundary types are included as discriminative features. [file 41586_2024_7981_MOESM7_ESM.zip › DataS3/Li16.pdf]

Li17

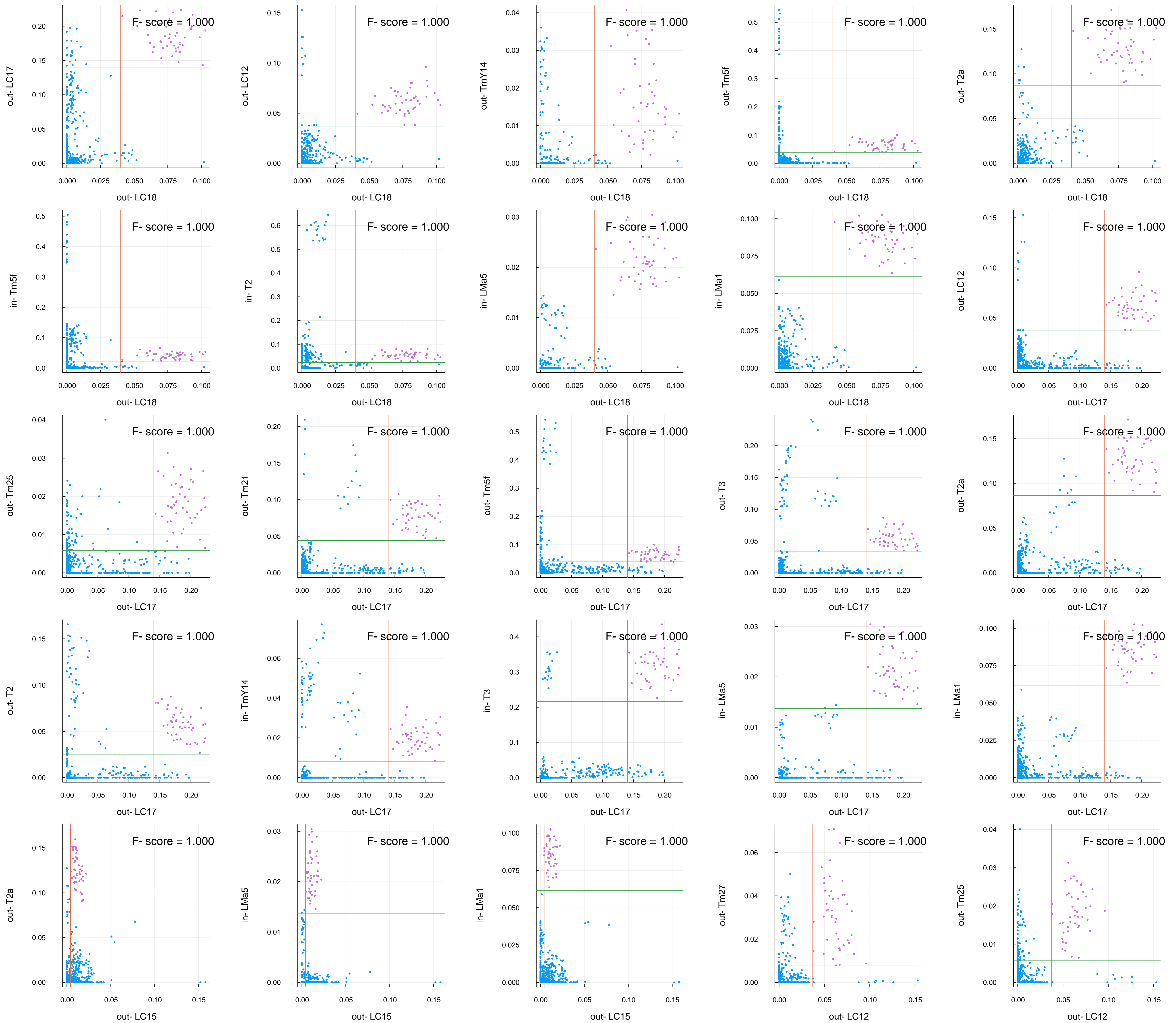

Supplement: Supplementary file 7 — Discriminating 2D projections for neuropil-intrinsic types. For each interneuron type, a pair of features is shown that can be used to discriminate that type from others in the same neuropil. Many although not all discriminations are highly accurate. Both intrinsic and boundary types are included as discriminative features. [file 41586_2024_7981_MOESM7_ESM.zip › DataS3/Li17.pdf]

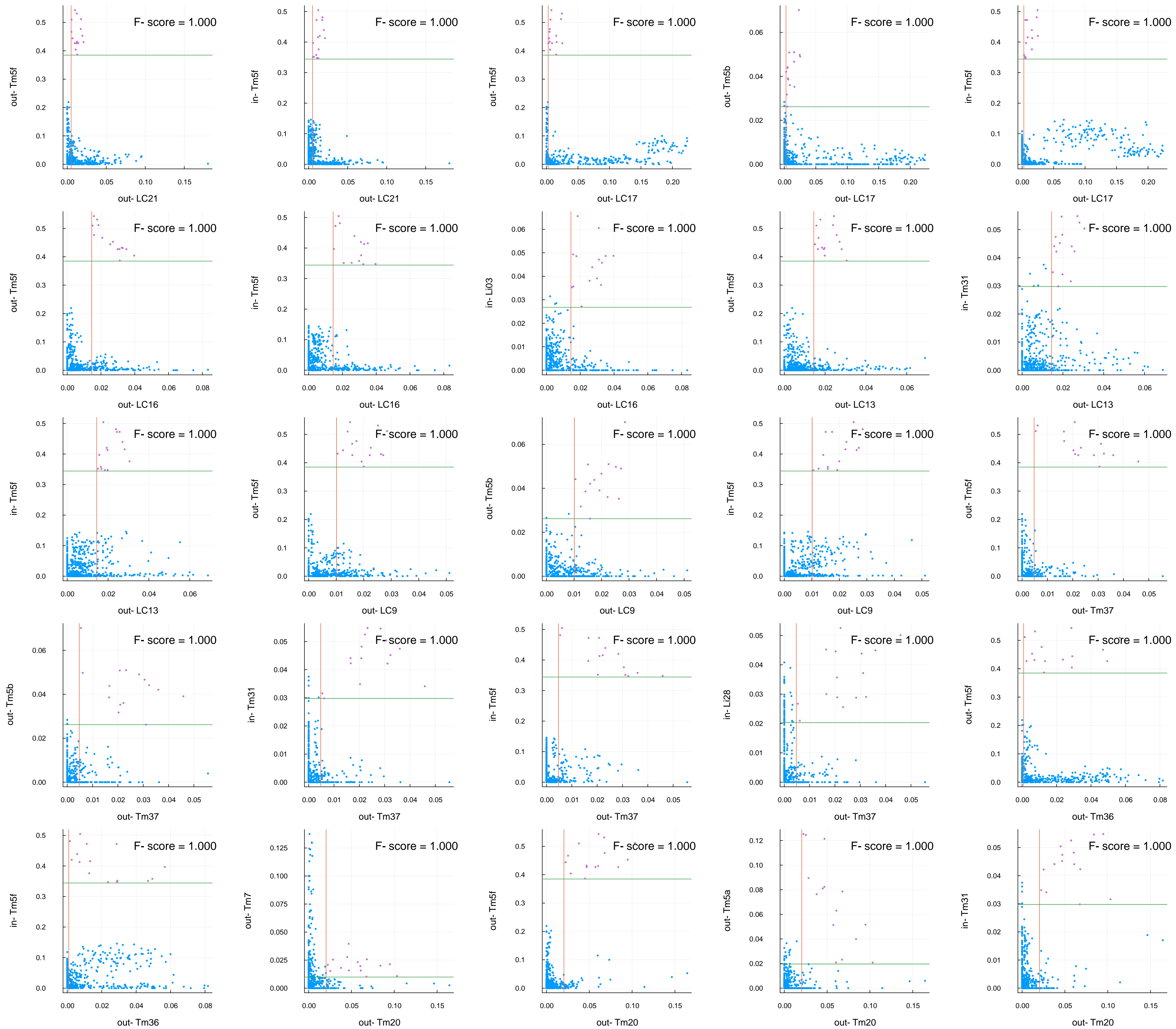

Supplement: Supplementary file 7 — Discriminating 2D projections for neuropil-intrinsic types. For each interneuron type, a pair of features is shown that can be used to discriminate that type from others in the same neuropil. Many although not all discriminations are highly accurate. Both intrinsic and boundary types are included as discriminative features. [file 41586_2024_7981_MOESM7_ESM.zip › DataS3/Li18.pdf]

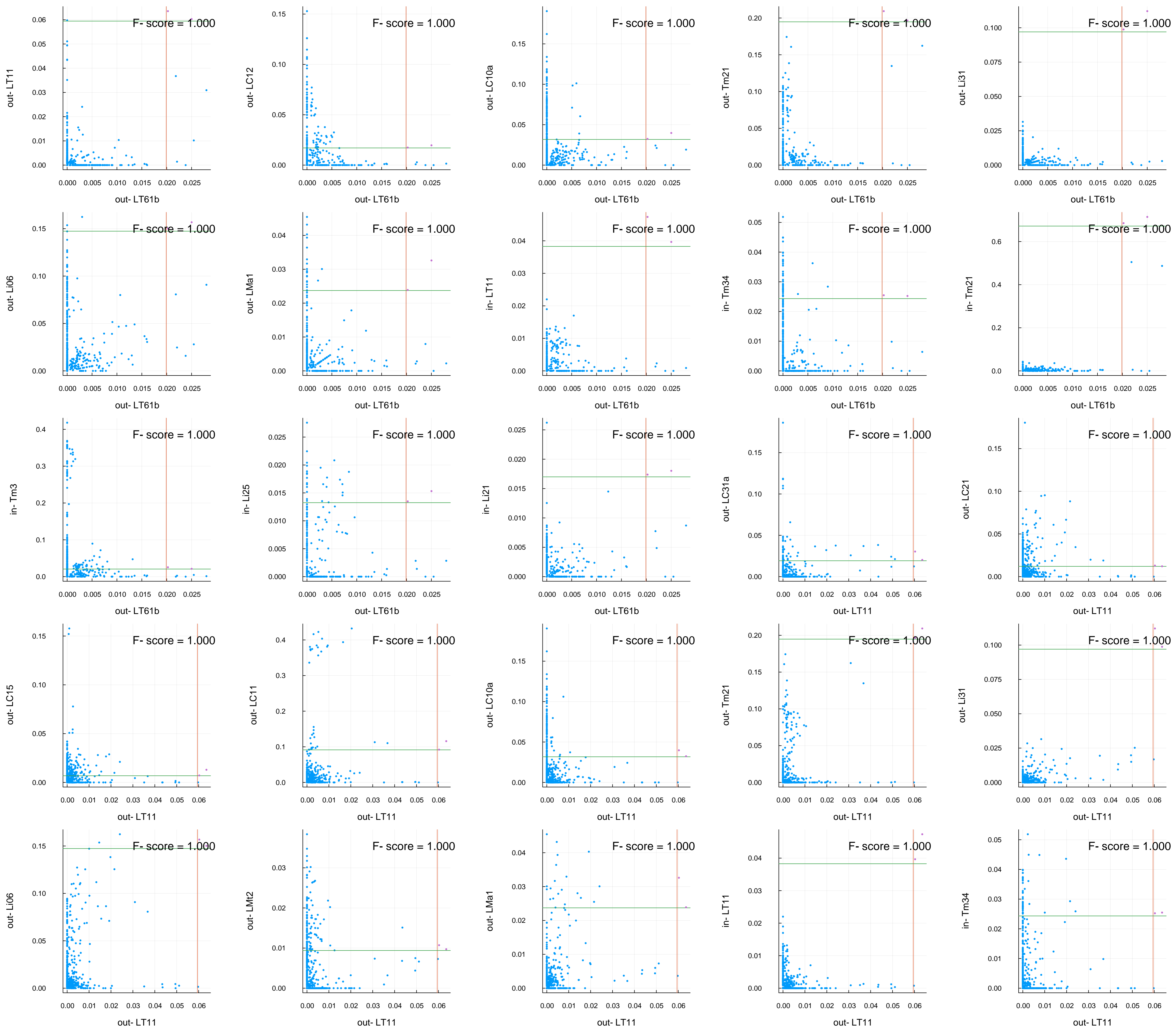

Supplement: Supplementary file 7 — Discriminating 2D projections for neuropil-intrinsic types. For each interneuron type, a pair of features is shown that can be used to discriminate that type from others in the same neuropil. Many although not all discriminations are highly accurate. Both intrinsic and boundary types are included as discriminative features. [file 41586_2024_7981_MOESM7_ESM.zip › DataS3/Li19.pdf]

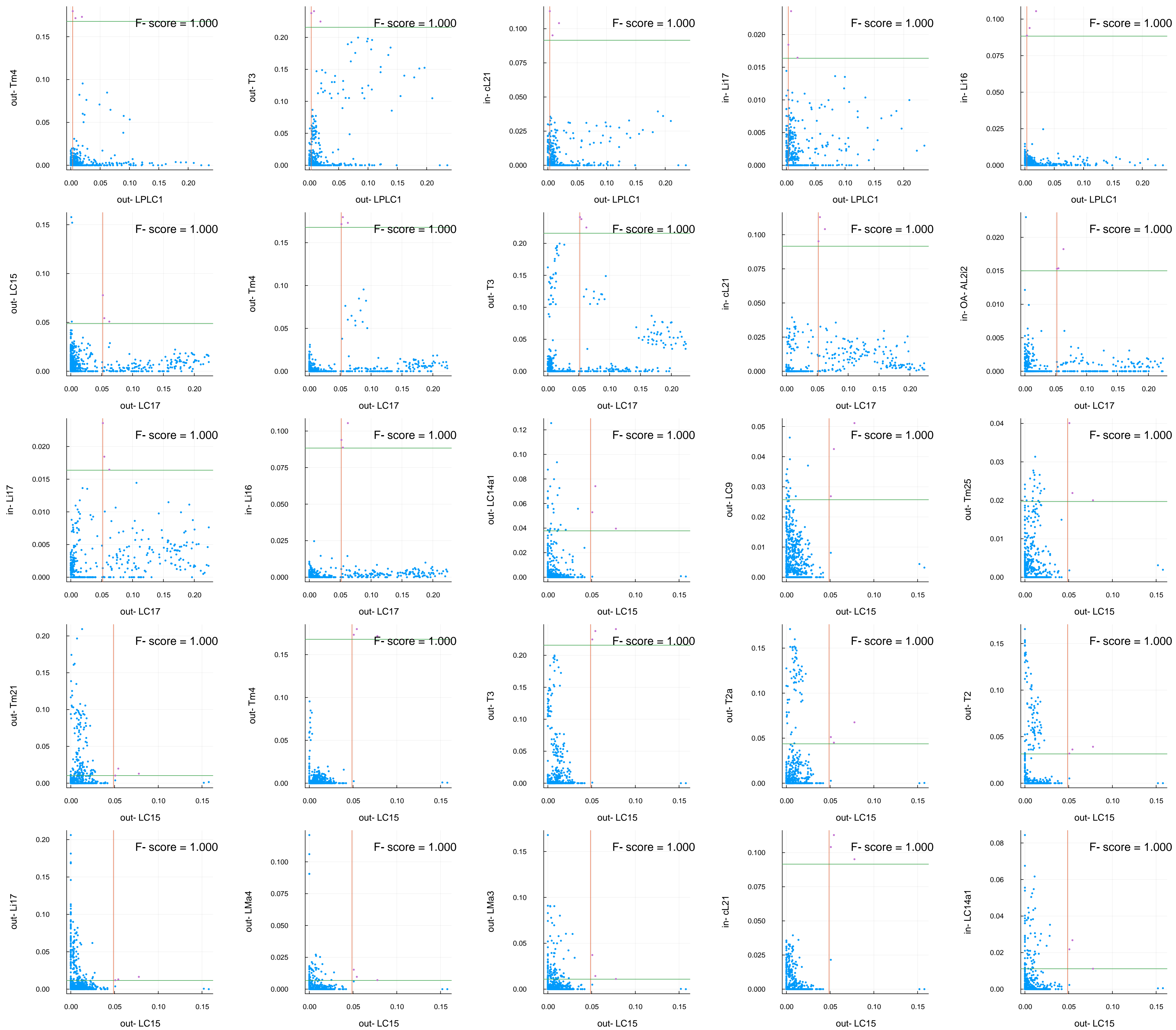

Supplement: Supplementary file 7 — Discriminating 2D projections for neuropil-intrinsic types. For each interneuron type, a pair of features is shown that can be used to discriminate that type from others in the same neuropil. Many although not all discriminations are highly accurate. Both intrinsic and boundary types are included as discriminative features. [file 41586_2024_7981_MOESM7_ESM.zip › DataS3/Li20.pdf]

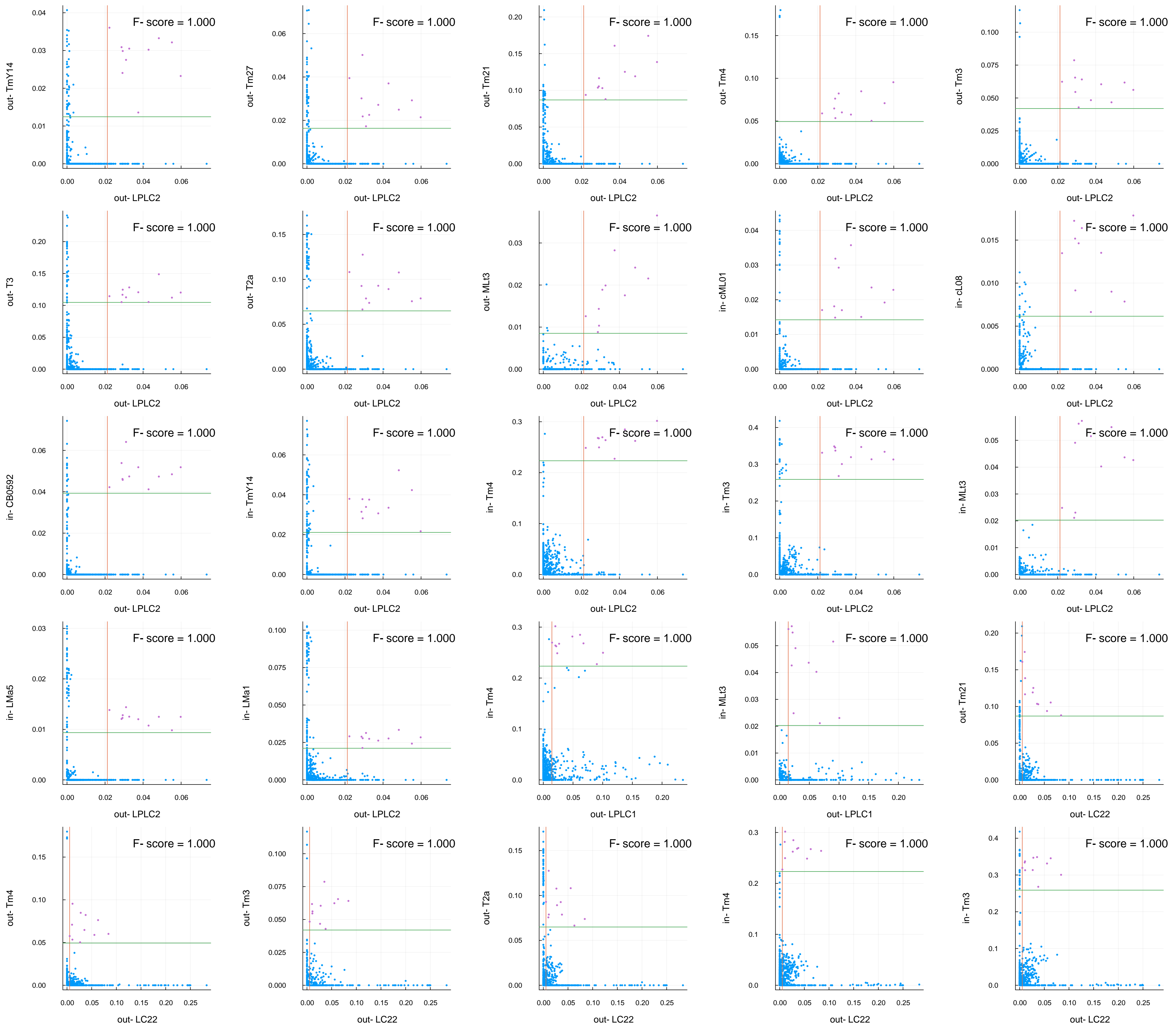

Supplement: Supplementary file 7 — Discriminating 2D projections for neuropil-intrinsic types. For each interneuron type, a pair of features is shown that can be used to discriminate that type from others in the same neuropil. Many although not all discriminations are highly accurate. Both intrinsic and boundary types are included as discriminative features. [file 41586_2024_7981_MOESM7_ESM.zip › DataS3/Li21.pdf]

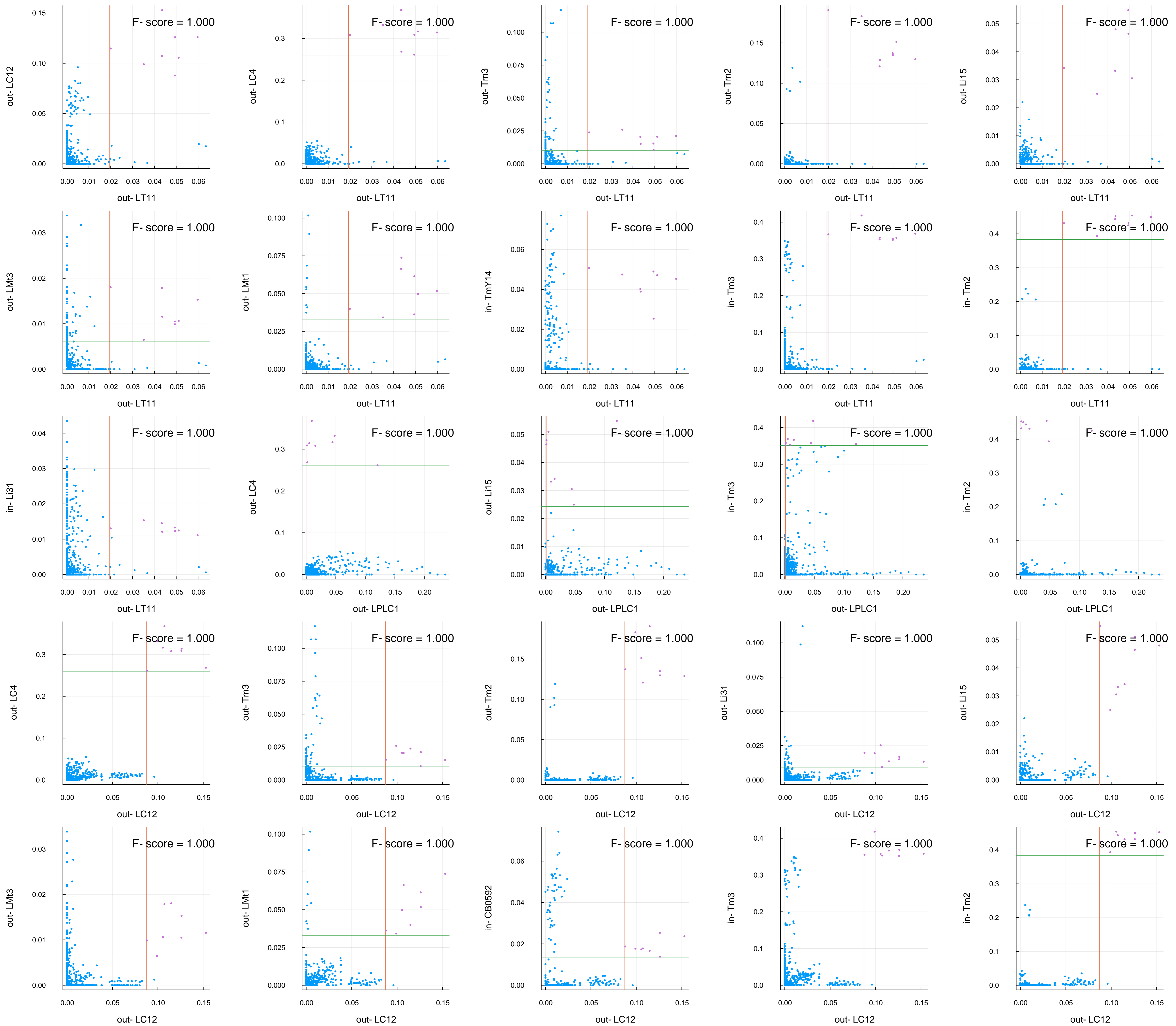

Supplement: Supplementary file 7 — Discriminating 2D projections for neuropil-intrinsic types. For each interneuron type, a pair of features is shown that can be used to discriminate that type from others in the same neuropil. Many although not all discriminations are highly accurate. Both intrinsic and boundary types are included as discriminative features. [file 41586_2024_7981_MOESM7_ESM.zip › DataS3/Li22.pdf]

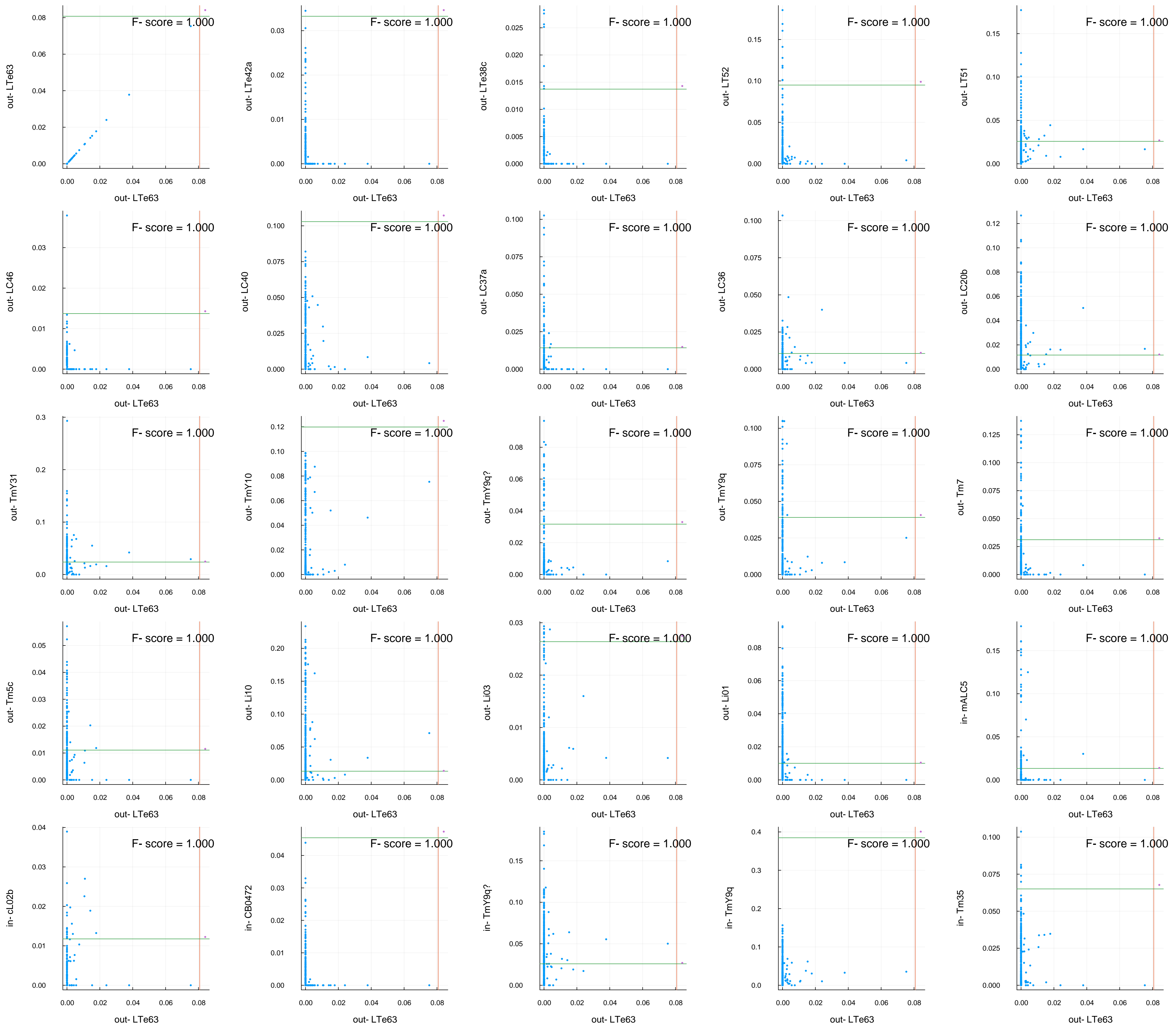

Supplement: Supplementary file 7 — Discriminating 2D projections for neuropil-intrinsic types. For each interneuron type, a pair of features is shown that can be used to discriminate that type from others in the same neuropil. Many although not all discriminations are highly accurate. Both intrinsic and boundary types are included as discriminative features. [file 41586_2024_7981_MOESM7_ESM.zip › DataS3/Li23.pdf]

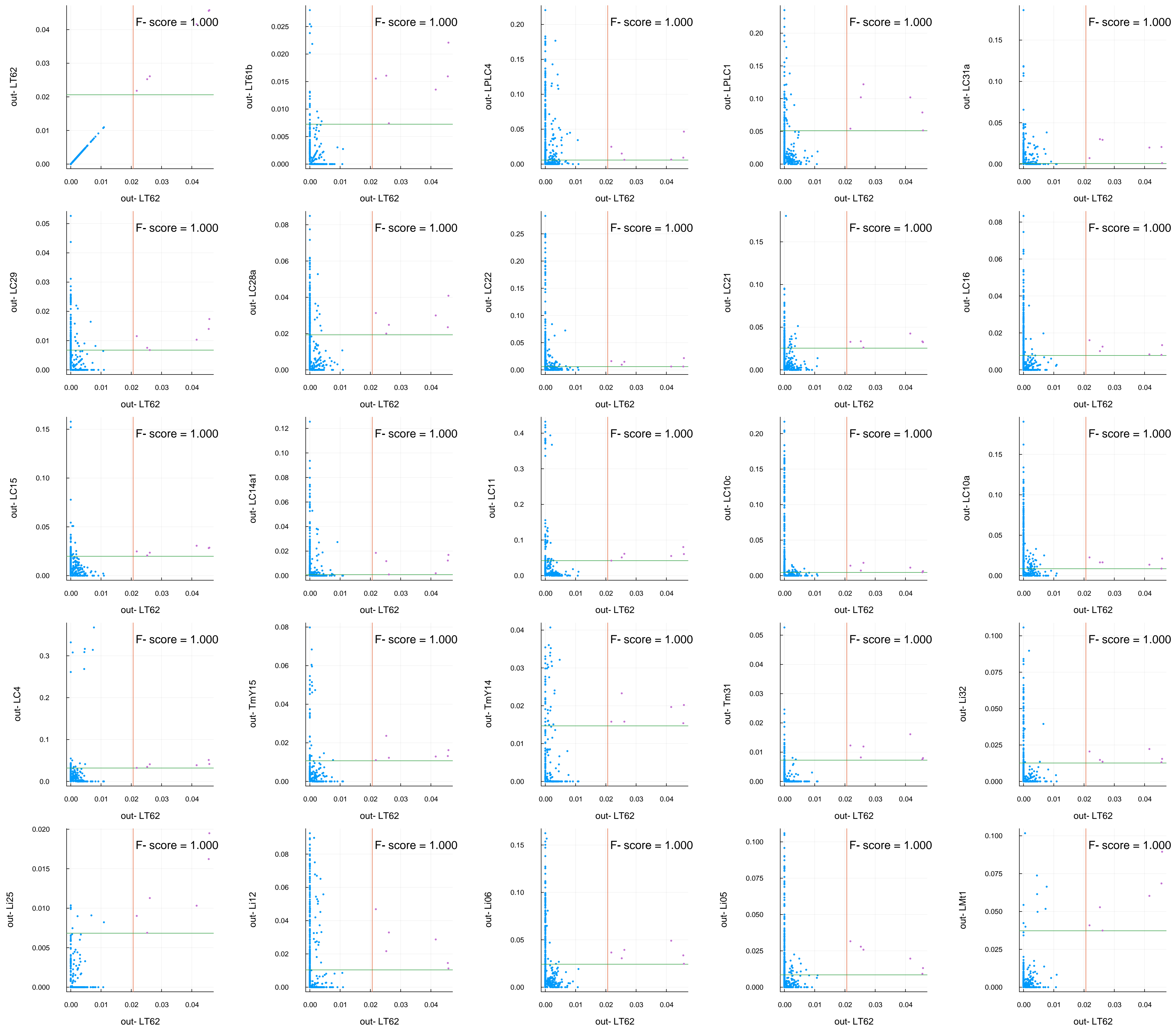

Supplement: Supplementary file 7 — Discriminating 2D projections for neuropil-intrinsic types. For each interneuron type, a pair of features is shown that can be used to discriminate that type from others in the same neuropil. Many although not all discriminations are highly accurate. Both intrinsic and boundary types are included as discriminative features. [file 41586_2024_7981_MOESM7_ESM.zip › DataS3/Li24.pdf]

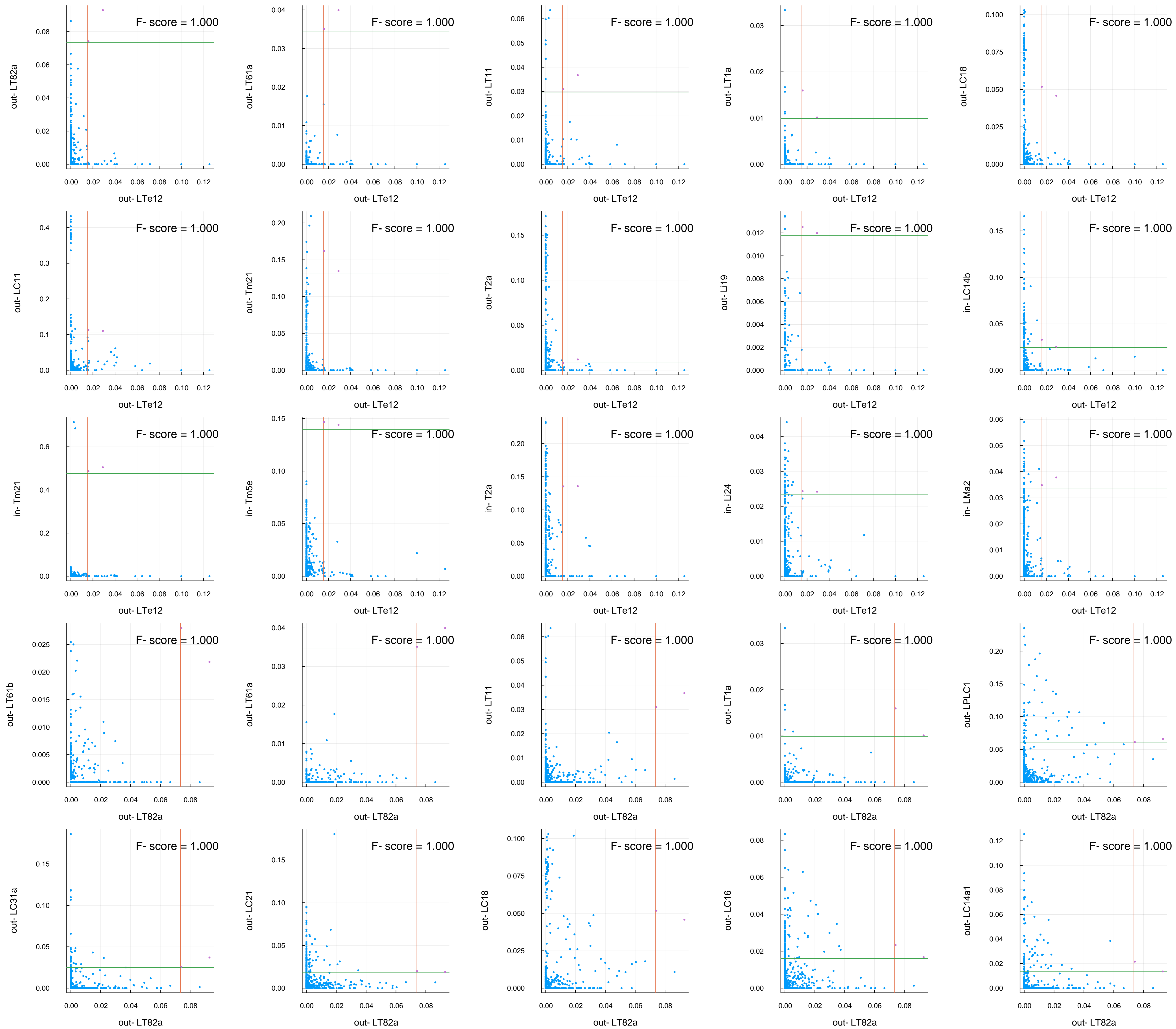

Supplement: Supplementary file 7 — Discriminating 2D projections for neuropil-intrinsic types. For each interneuron type, a pair of features is shown that can be used to discriminate that type from others in the same neuropil. Many although not all discriminations are highly accurate. Both intrinsic and boundary types are included as discriminative features. [file 41586_2024_7981_MOESM7_ESM.zip › DataS3/Li25.pdf]

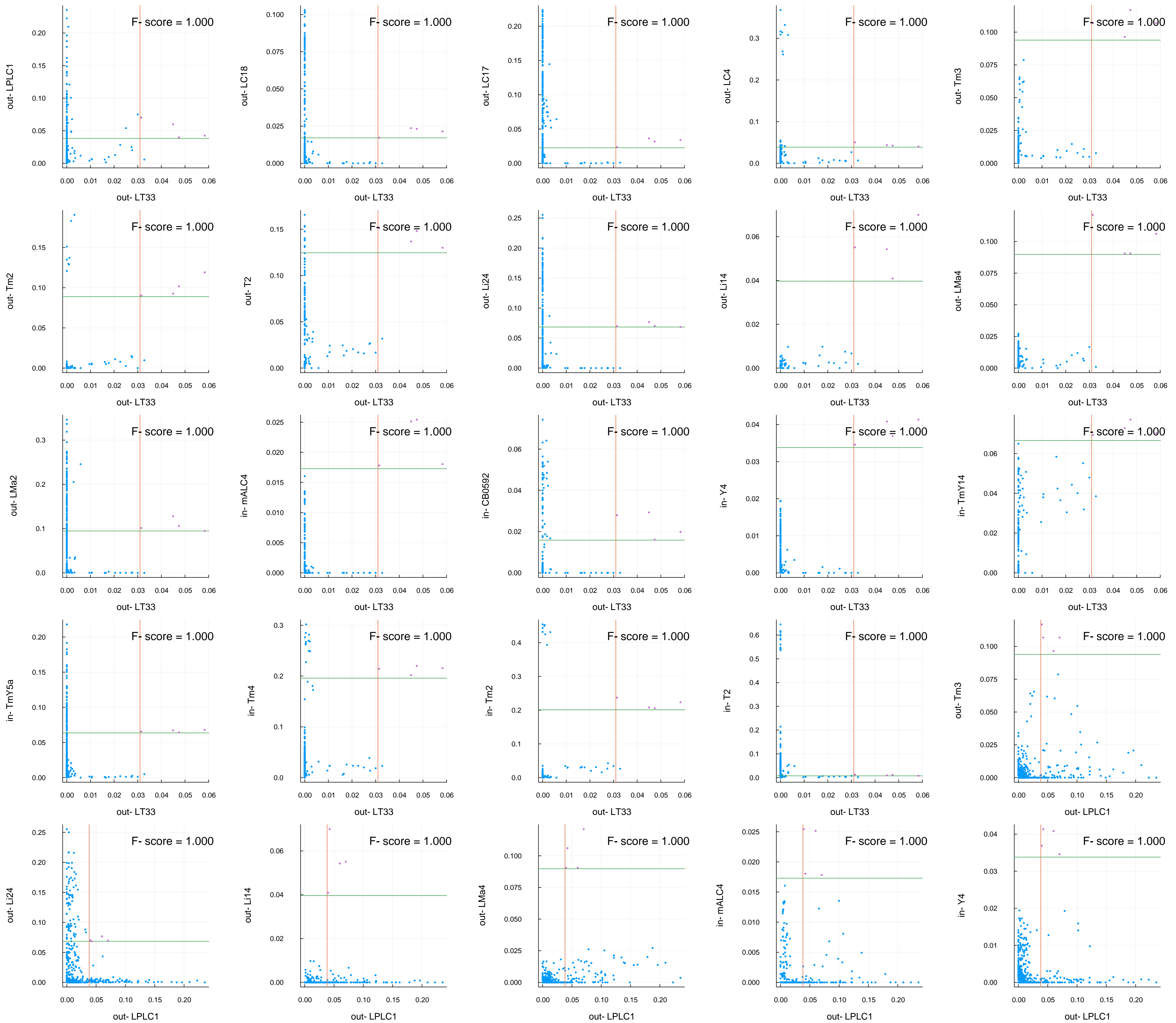

Supplement: Supplementary file 7 — Discriminating 2D projections for neuropil-intrinsic types. For each interneuron type, a pair of features is shown that can be used to discriminate that type from others in the same neuropil. Many although not all discriminations are highly accurate. Both intrinsic and boundary types are included as discriminative features. [file 41586_2024_7981_MOESM7_ESM.zip › DataS3/Li26.pdf]

Li27

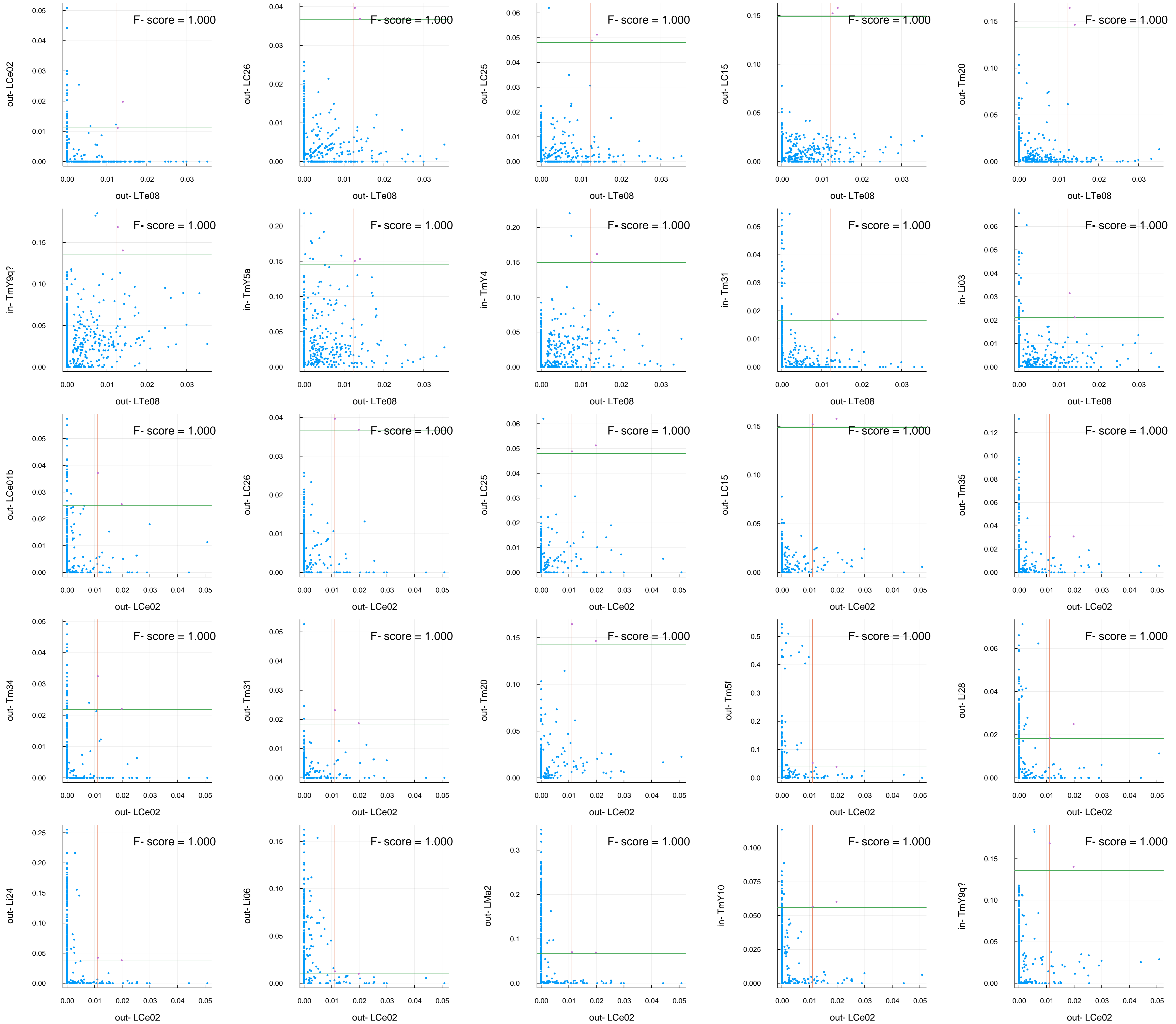

Supplement: Supplementary file 7 — Discriminating 2D projections for neuropil-intrinsic types. For each interneuron type, a pair of features is shown that can be used to discriminate that type from others in the same neuropil. Many although not all discriminations are highly accurate. Both intrinsic and boundary types are included as discriminative features. [file 41586_2024_7981_MOESM7_ESM.zip › DataS3/Li27.pdf]

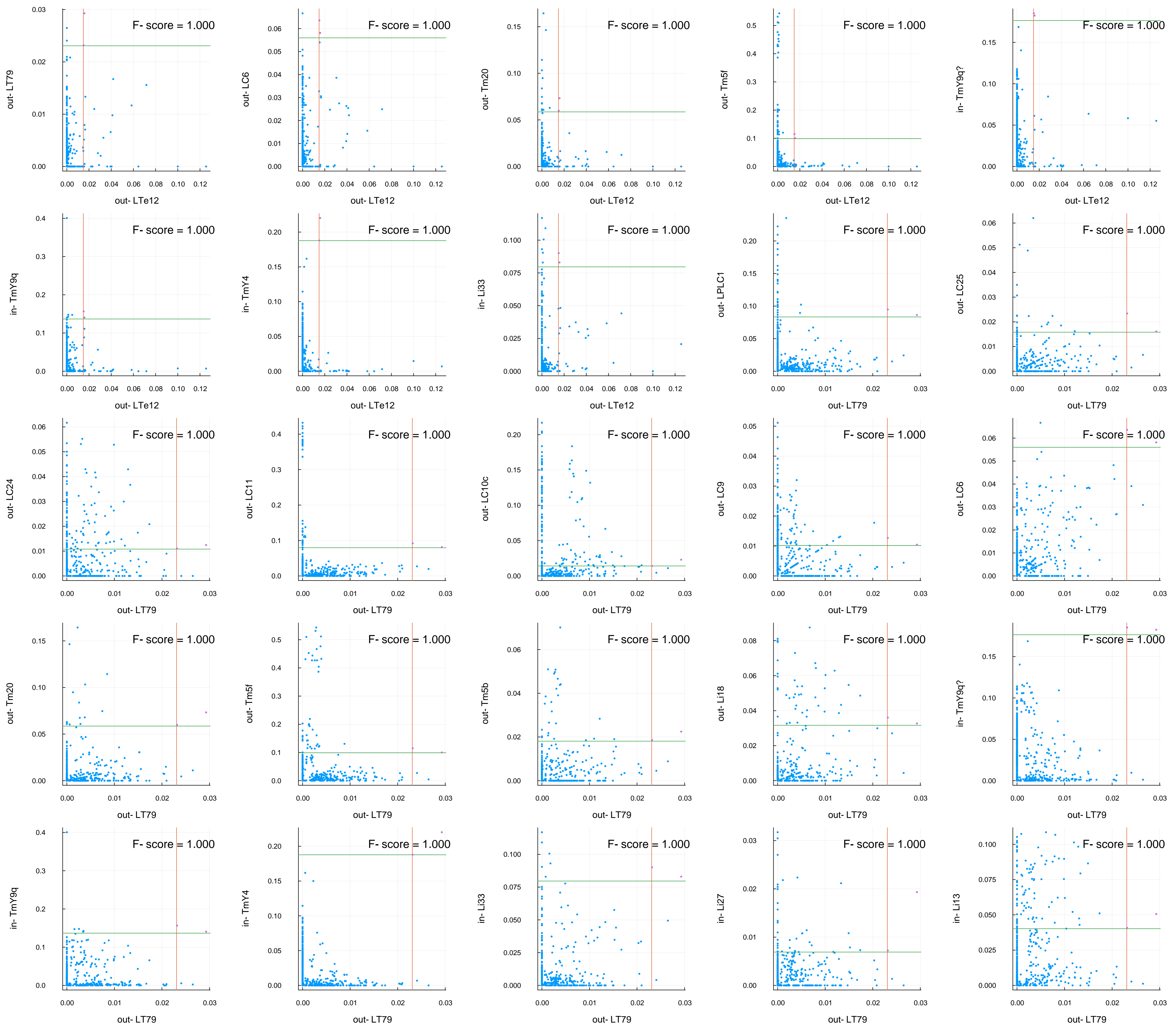

Supplement: Supplementary file 7 — Discriminating 2D projections for neuropil-intrinsic types. For each interneuron type, a pair of features is shown that can be used to discriminate that type from others in the same neuropil. Many although not all discriminations are highly accurate. Both intrinsic and boundary types are included as discriminative features. [file 41586_2024_7981_MOESM7_ESM.zip › DataS3/Li28.pdf]

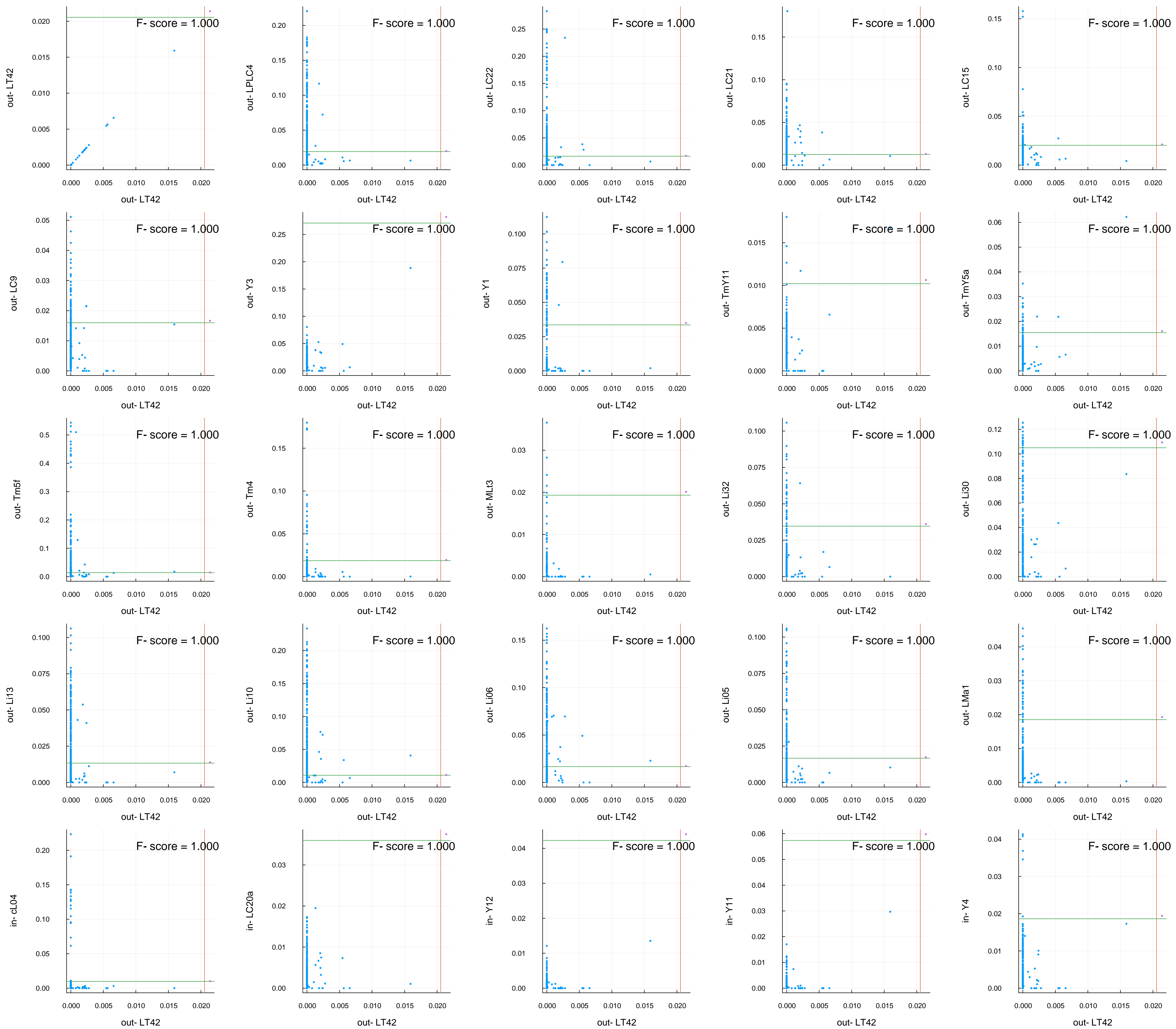

Supplement: Supplementary file 7 — Discriminating 2D projections for neuropil-intrinsic types. For each interneuron type, a pair of features is shown that can be used to discriminate that type from others in the same neuropil. Many although not all discriminations are highly accurate. Both intrinsic and boundary types are included as discriminative features. [file 41586_2024_7981_MOESM7_ESM.zip › DataS3/Li29.pdf]

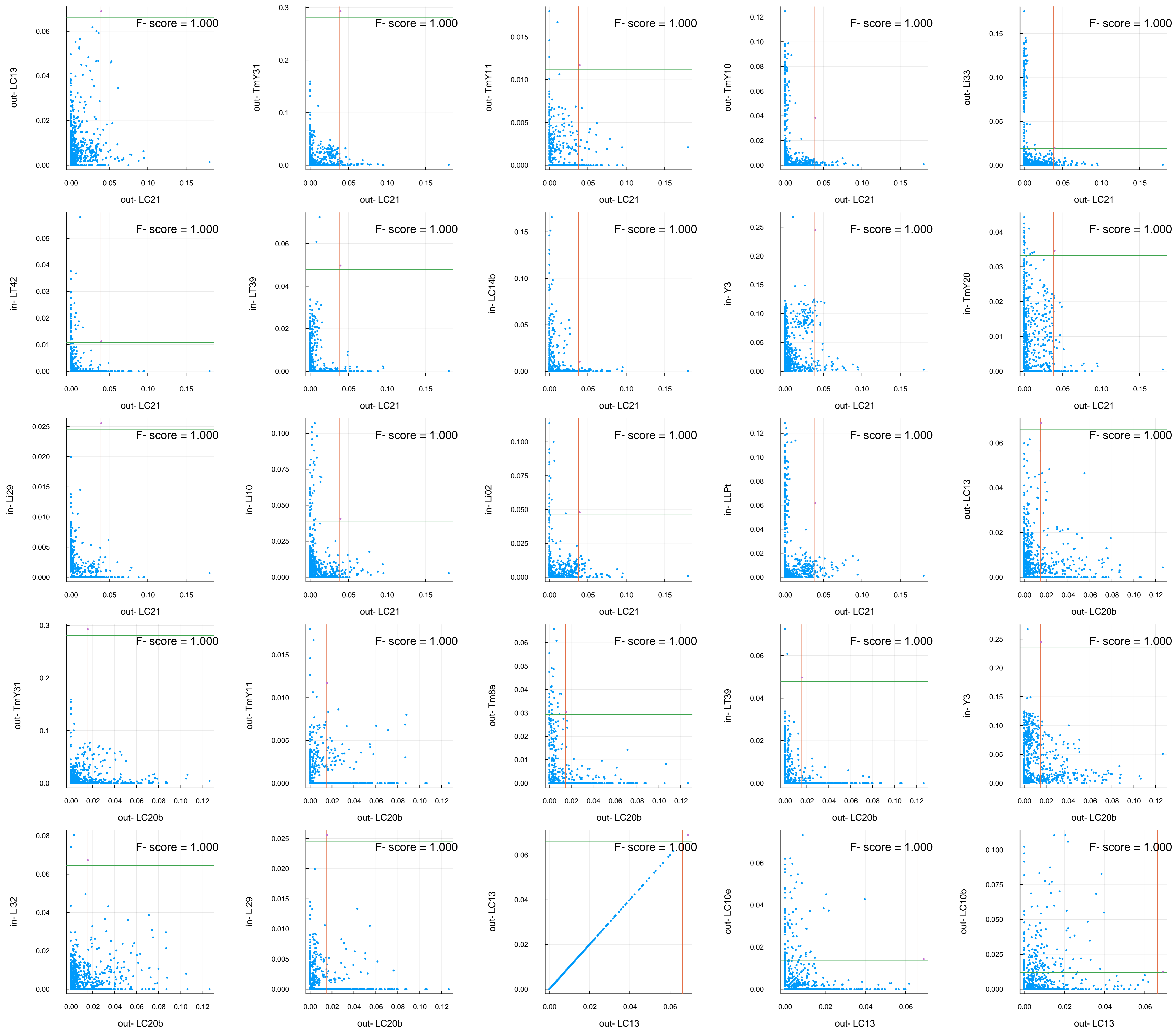

Supplement: Supplementary file 7 — Discriminating 2D projections for neuropil-intrinsic types. For each interneuron type, a pair of features is shown that can be used to discriminate that type from others in the same neuropil. Many although not all discriminations are highly accurate. Both intrinsic and boundary types are included as discriminative features. [file 41586_2024_7981_MOESM7_ESM.zip › DataS3/Li30.pdf]

Li31

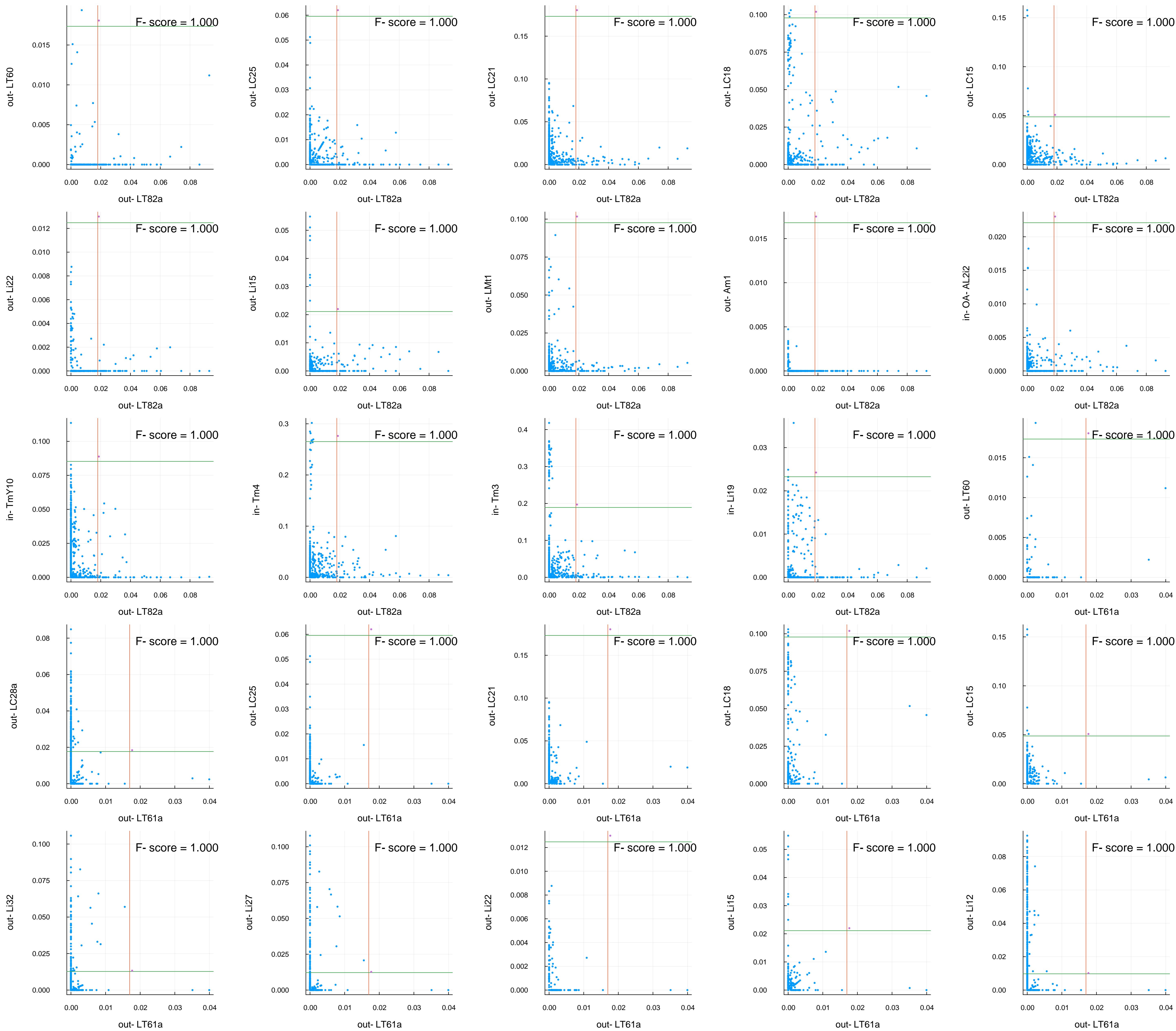

Supplement: Supplementary file 7 — Discriminating 2D projections for neuropil-intrinsic types. For each interneuron type, a pair of features is shown that can be used to discriminate that type from others in the same neuropil. Many although not all discriminations are highly accurate. Both intrinsic and boundary types are included as discriminative features. [file 41586_2024_7981_MOESM7_ESM.zip › DataS3/Li31.pdf]

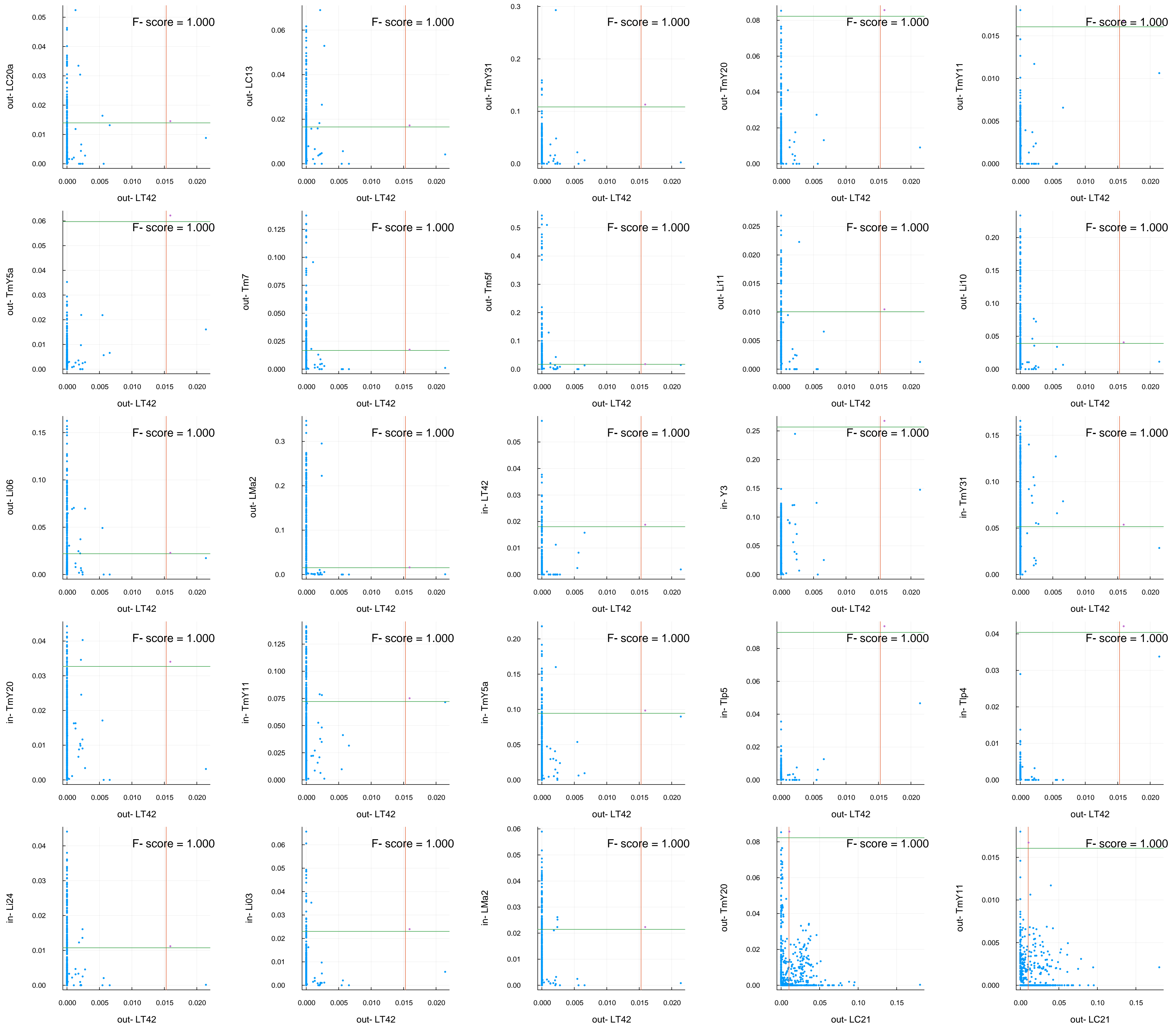

Supplement: Supplementary file 7 — Discriminating 2D projections for neuropil-intrinsic types. For each interneuron type, a pair of features is shown that can be used to discriminate that type from others in the same neuropil. Many although not all discriminations are highly accurate. Both intrinsic and boundary types are included as discriminative features. [file 41586_2024_7981_MOESM7_ESM.zip › DataS3/Li32.pdf]

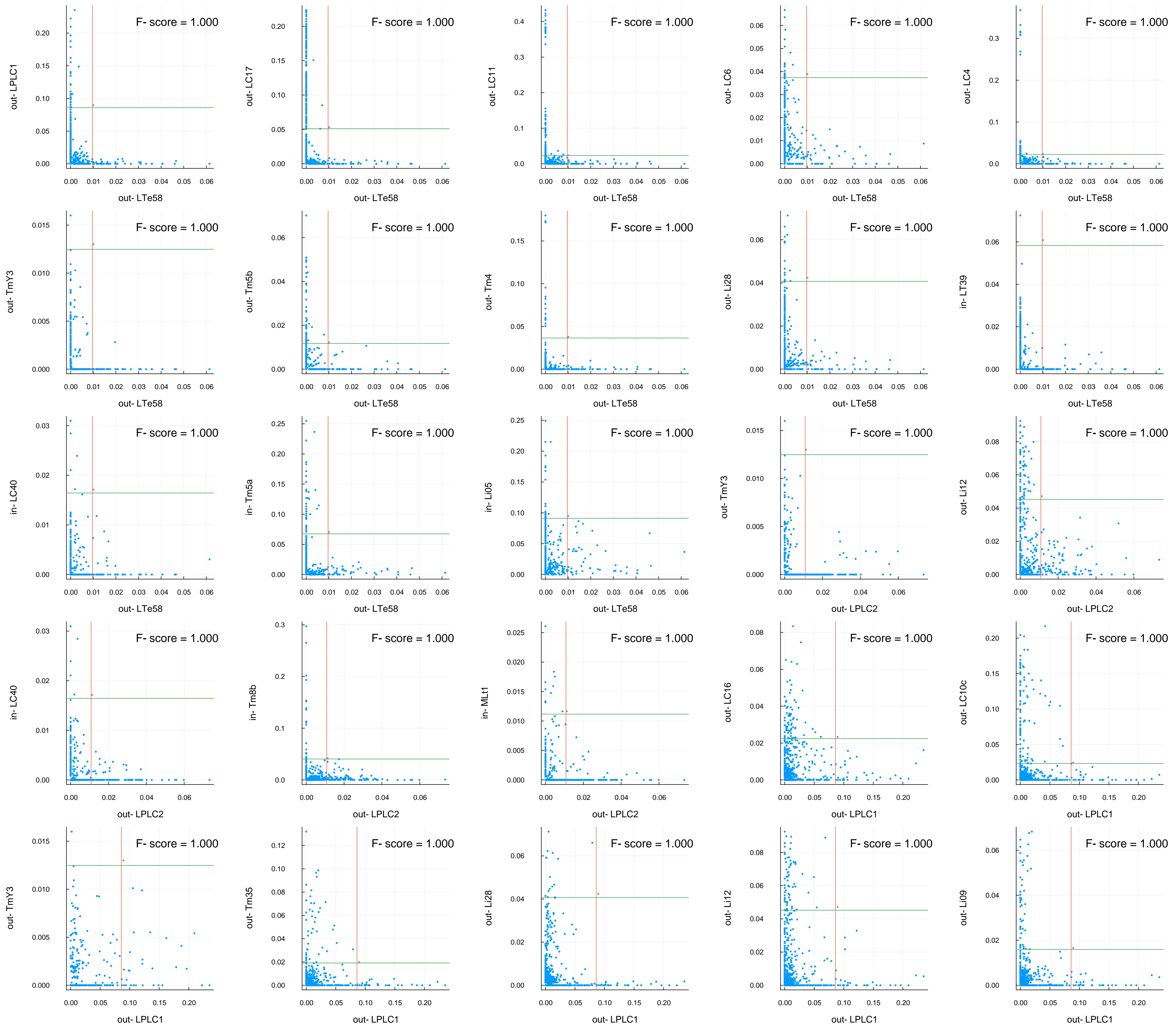

Supplement: Supplementary file 7 — Discriminating 2D projections for neuropil-intrinsic types. For each interneuron type, a pair of features is shown that can be used to discriminate that type from others in the same neuropil. Many although not all discriminations are highly accurate. Both intrinsic and boundary types are included as discriminative features. [file 41586_2024_7981_MOESM7_ESM.zip › DataS3/Li33.pdf]

LMa1

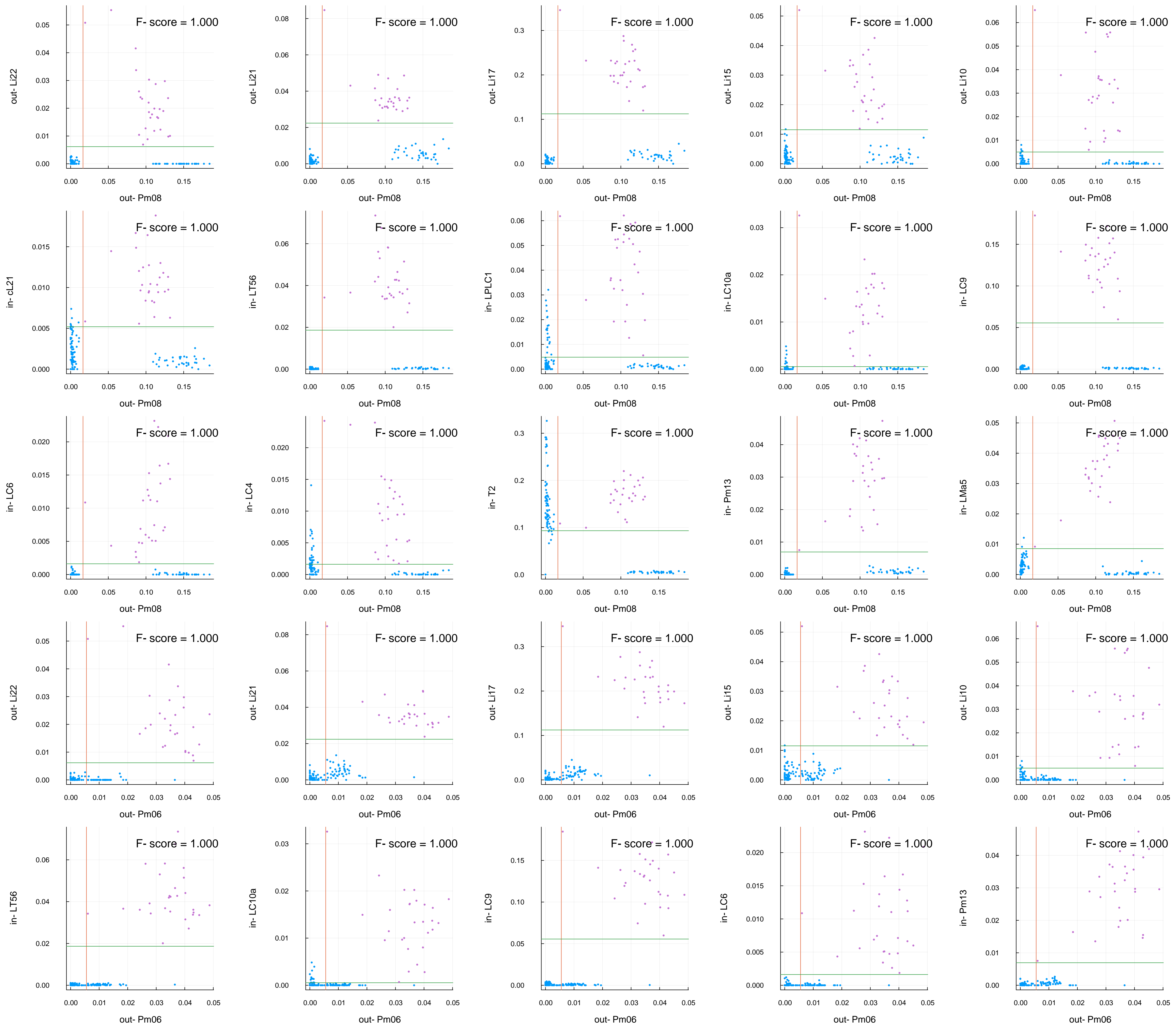

Supplement: Supplementary file 7 — Discriminating 2D projections for neuropil-intrinsic types. For each interneuron type, a pair of features is shown that can be used to discriminate that type from others in the same neuropil. Many although not all discriminations are highly accurate. Both intrinsic and boundary types are included as discriminative features. [file 41586_2024_7981_MOESM7_ESM.zip › DataS3/LMa1.pdf]

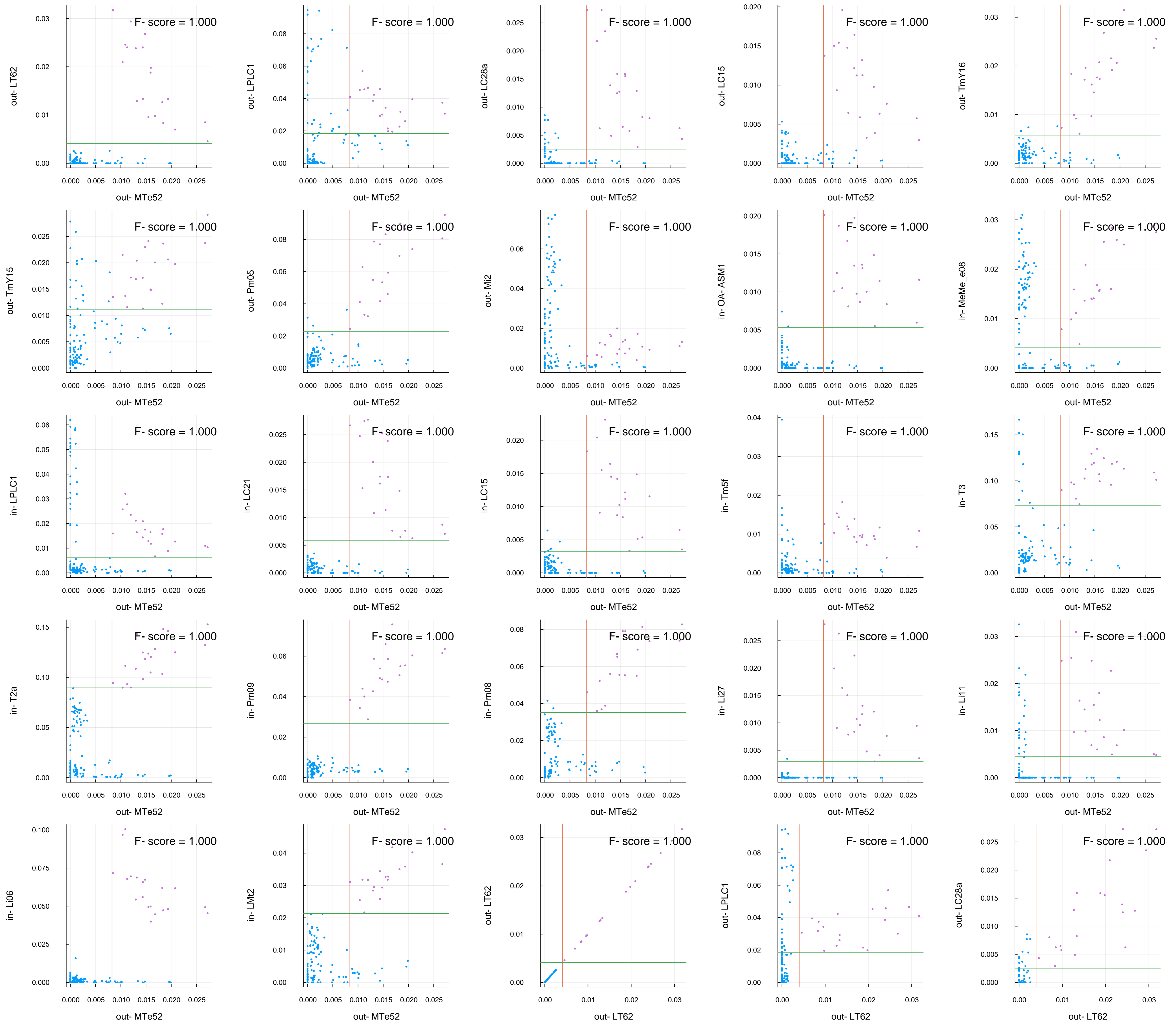

Supplement: Supplementary file 7 — Discriminating 2D projections for neuropil-intrinsic types. For each interneuron type, a pair of features is shown that can be used to discriminate that type from others in the same neuropil. Many although not all discriminations are highly accurate. Both intrinsic and boundary types are included as discriminative features. [file 41586_2024_7981_MOESM7_ESM.zip › DataS3/LMa2.pdf]

# LMa3

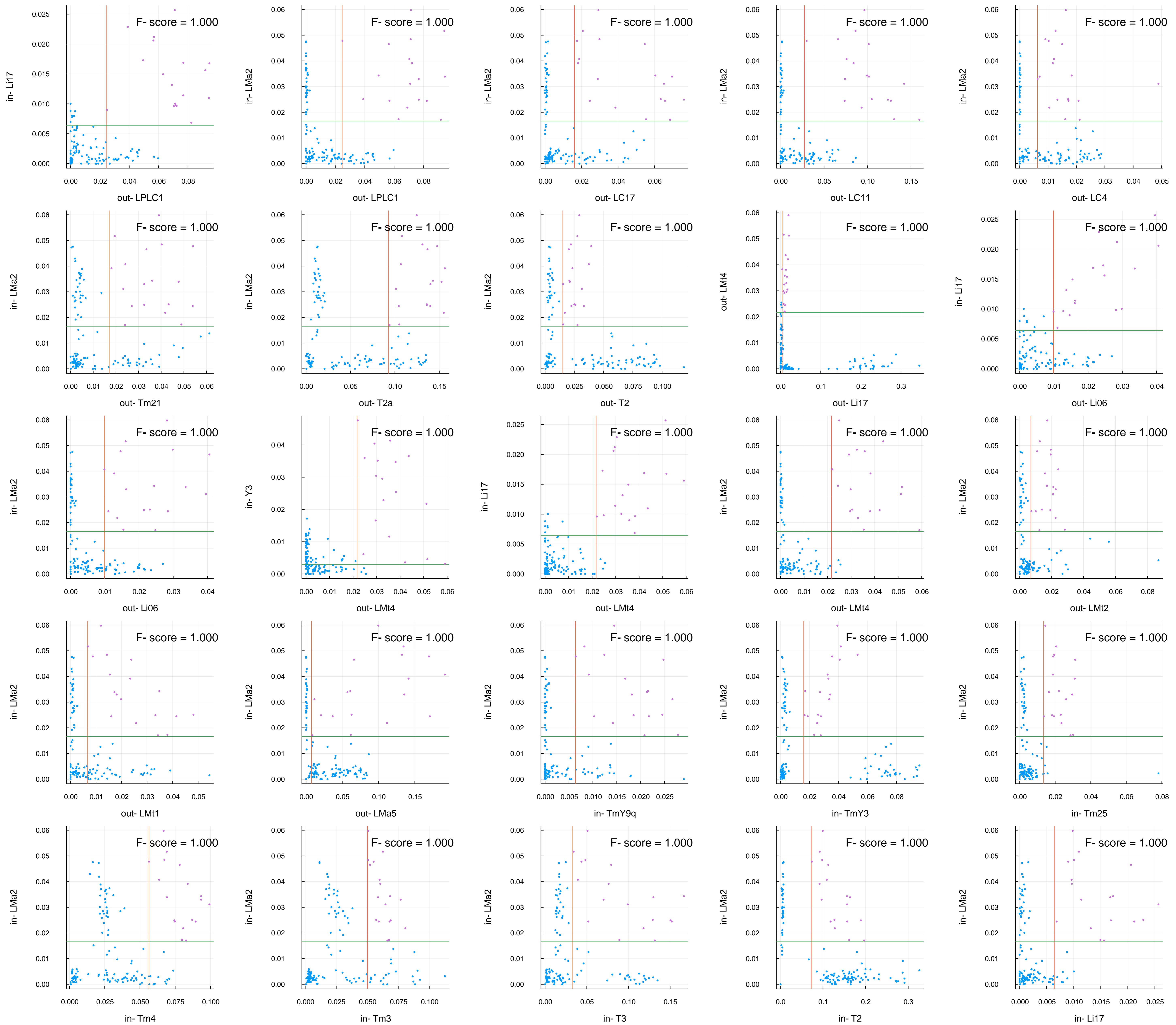

Supplement: Supplementary file 7 — Discriminating 2D projections for neuropil-intrinsic types. For each interneuron type, a pair of features is shown that can be used to discriminate that type from others in the same neuropil. Many although not all discriminations are highly accurate. Both intrinsic and boundary types are included as discriminative features. [file 41586_2024_7981_MOESM7_ESM.zip › DataS3/LMa3.pdf]

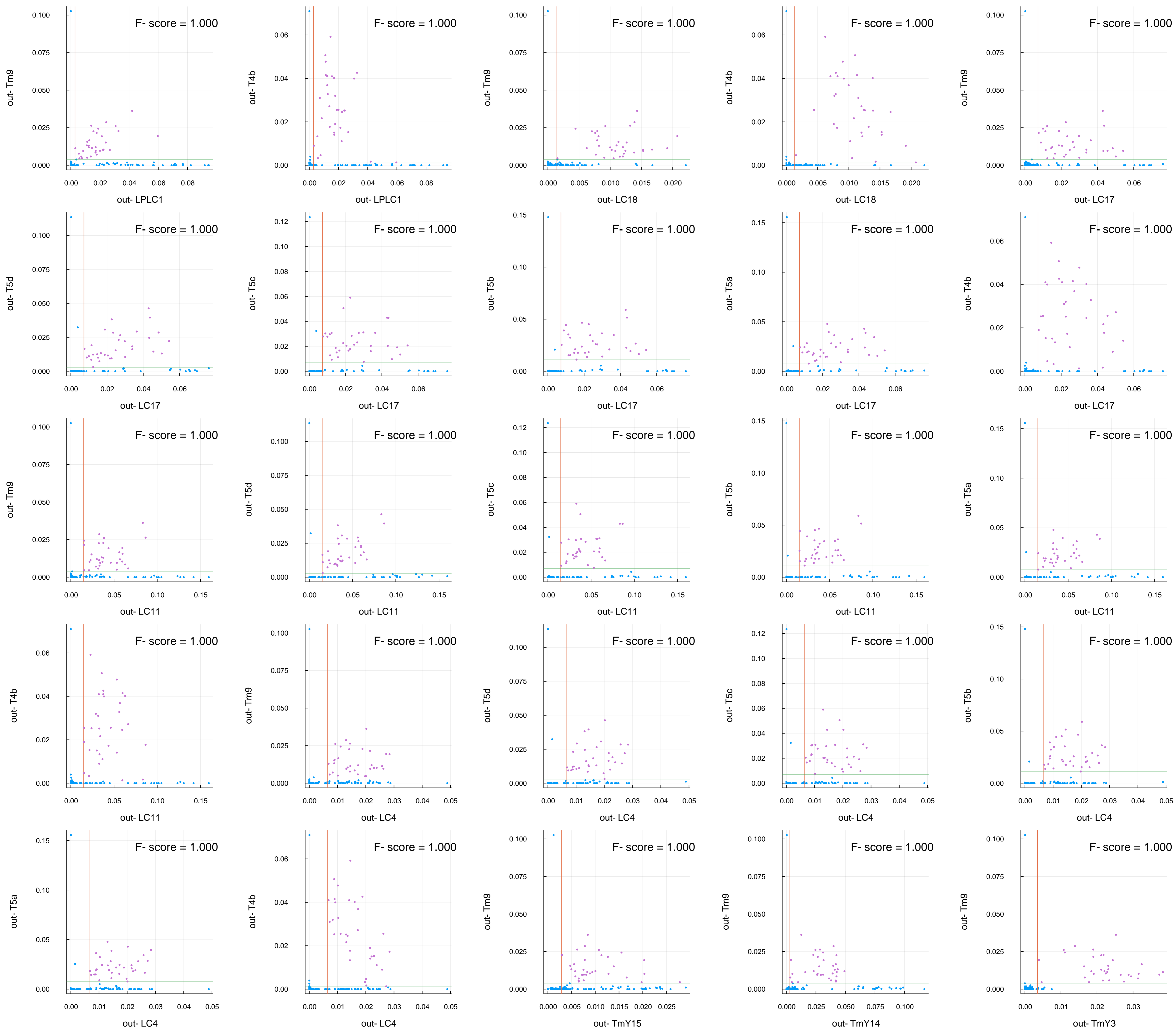

Supplement: Supplementary file 7 — Discriminating 2D projections for neuropil-intrinsic types. For each interneuron type, a pair of features is shown that can be used to discriminate that type from others in the same neuropil. Many although not all discriminations are highly accurate. Both intrinsic and boundary types are included as discriminative features. [file 41586_2024_7981_MOESM7_ESM.zip › DataS3/LMa4.pdf]

LMa5

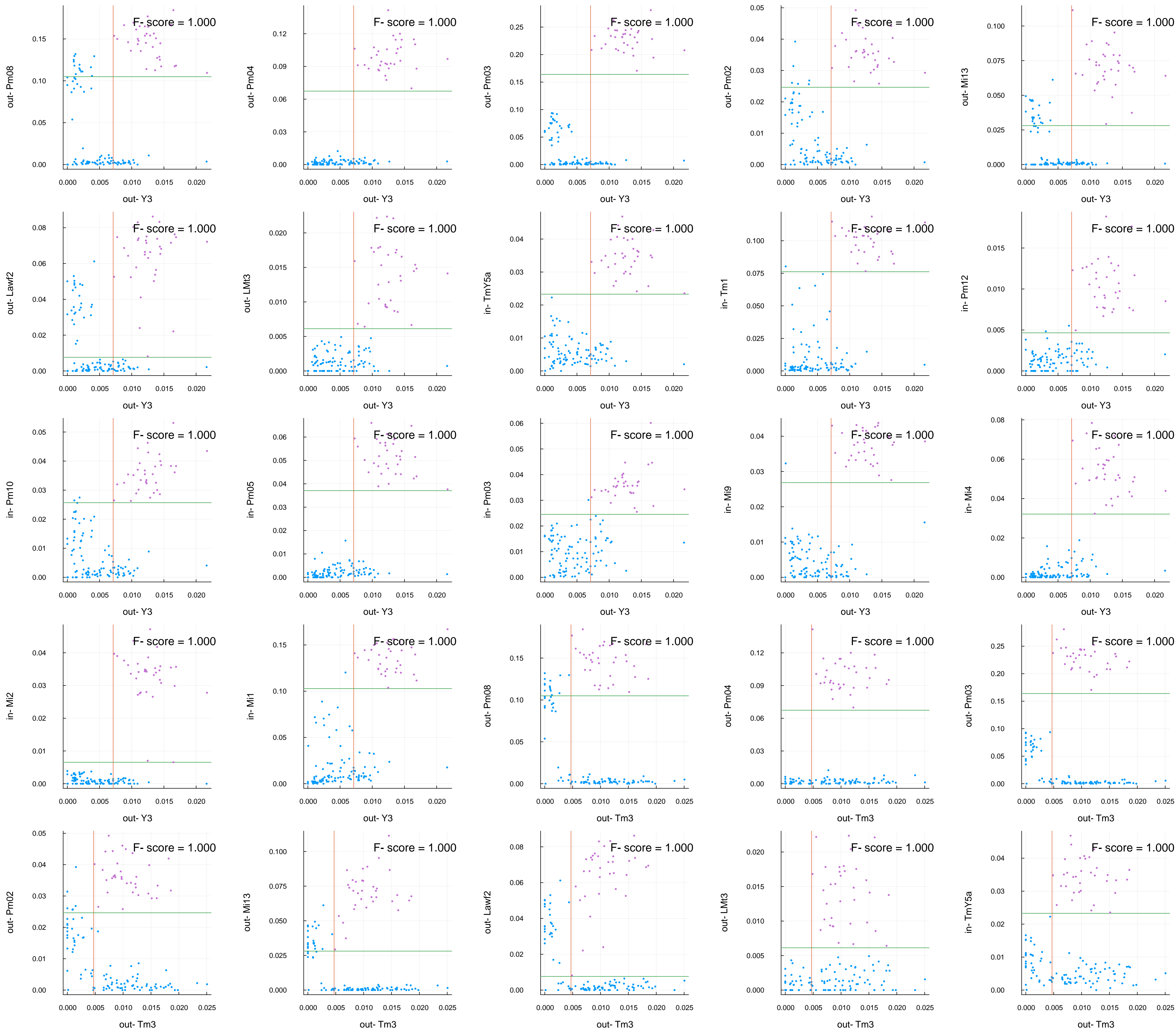

Supplement: Supplementary file 7 — Discriminating 2D projections for neuropil-intrinsic types. For each interneuron type, a pair of features is shown that can be used to discriminate that type from others in the same neuropil. Many although not all discriminations are highly accurate. Both intrinsic and boundary types are included as discriminative features. [file 41586_2024_7981_MOESM7_ESM.zip › DataS3/LMa5.pdf]

LMt1

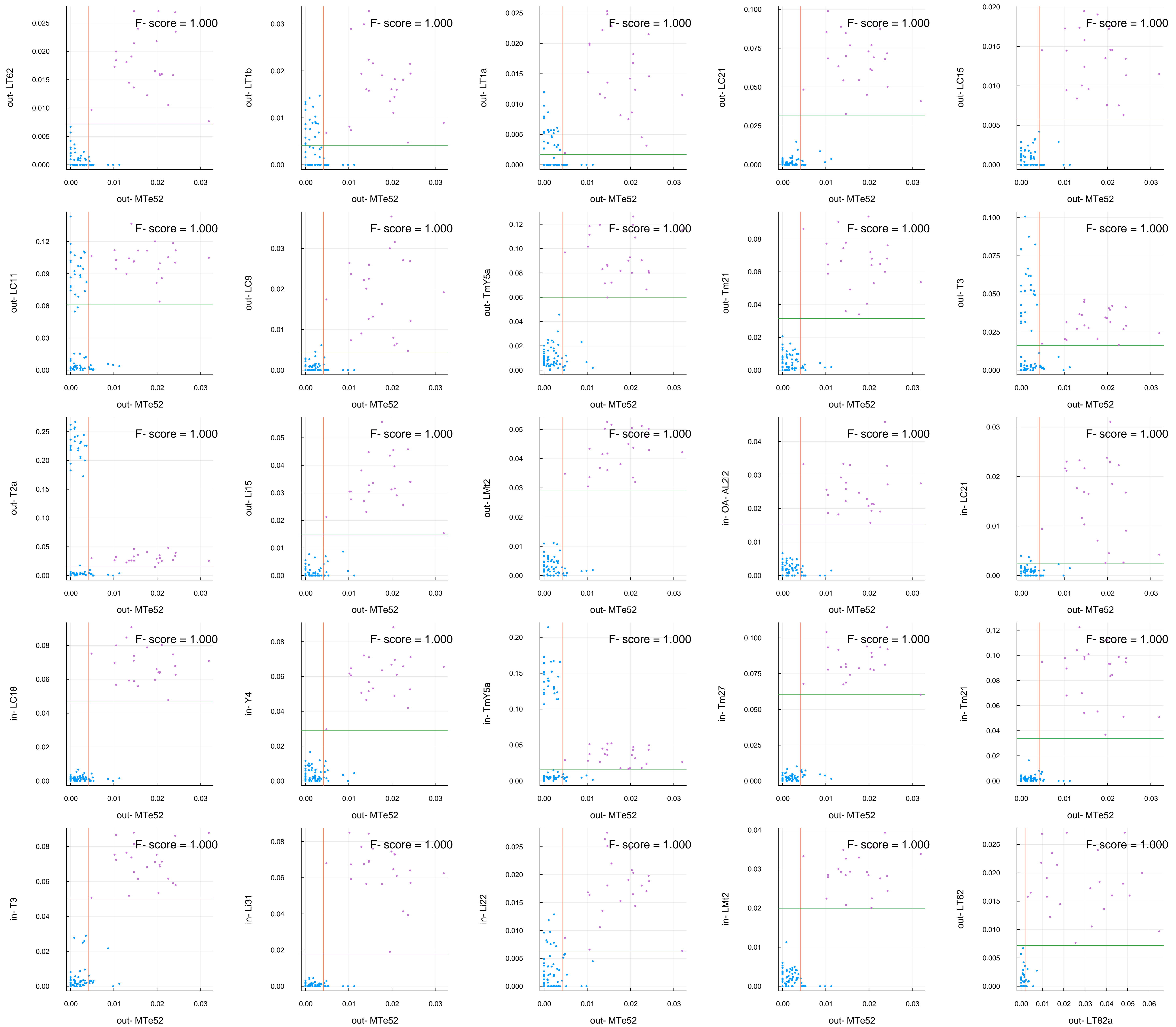

Supplement: Supplementary file 7 — Discriminating 2D projections for neuropil-intrinsic types. For each interneuron type, a pair of features is shown that can be used to discriminate that type from others in the same neuropil. Many although not all discriminations are highly accurate. Both intrinsic and boundary types are included as discriminative features. [file 41586_2024_7981_MOESM7_ESM.zip › DataS3/LMt1.pdf]

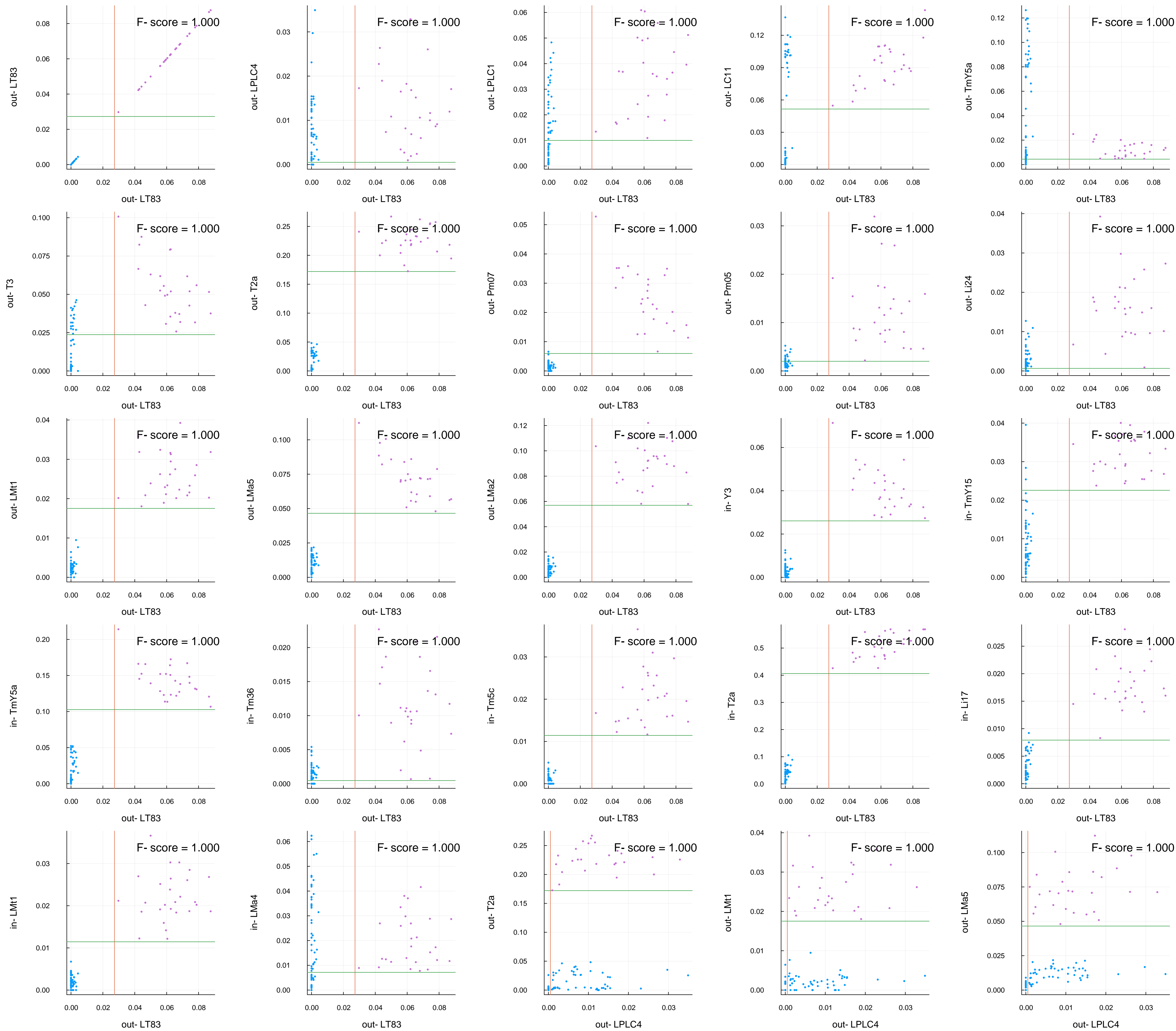

Supplement: Supplementary file 7 — Discriminating 2D projections for neuropil-intrinsic types. For each interneuron type, a pair of features is shown that can be used to discriminate that type from others in the same neuropil. Many although not all discriminations are highly accurate. Both intrinsic and boundary types are included as discriminative features. [file 41586_2024_7981_MOESM7_ESM.zip › DataS3/LMt2.pdf]

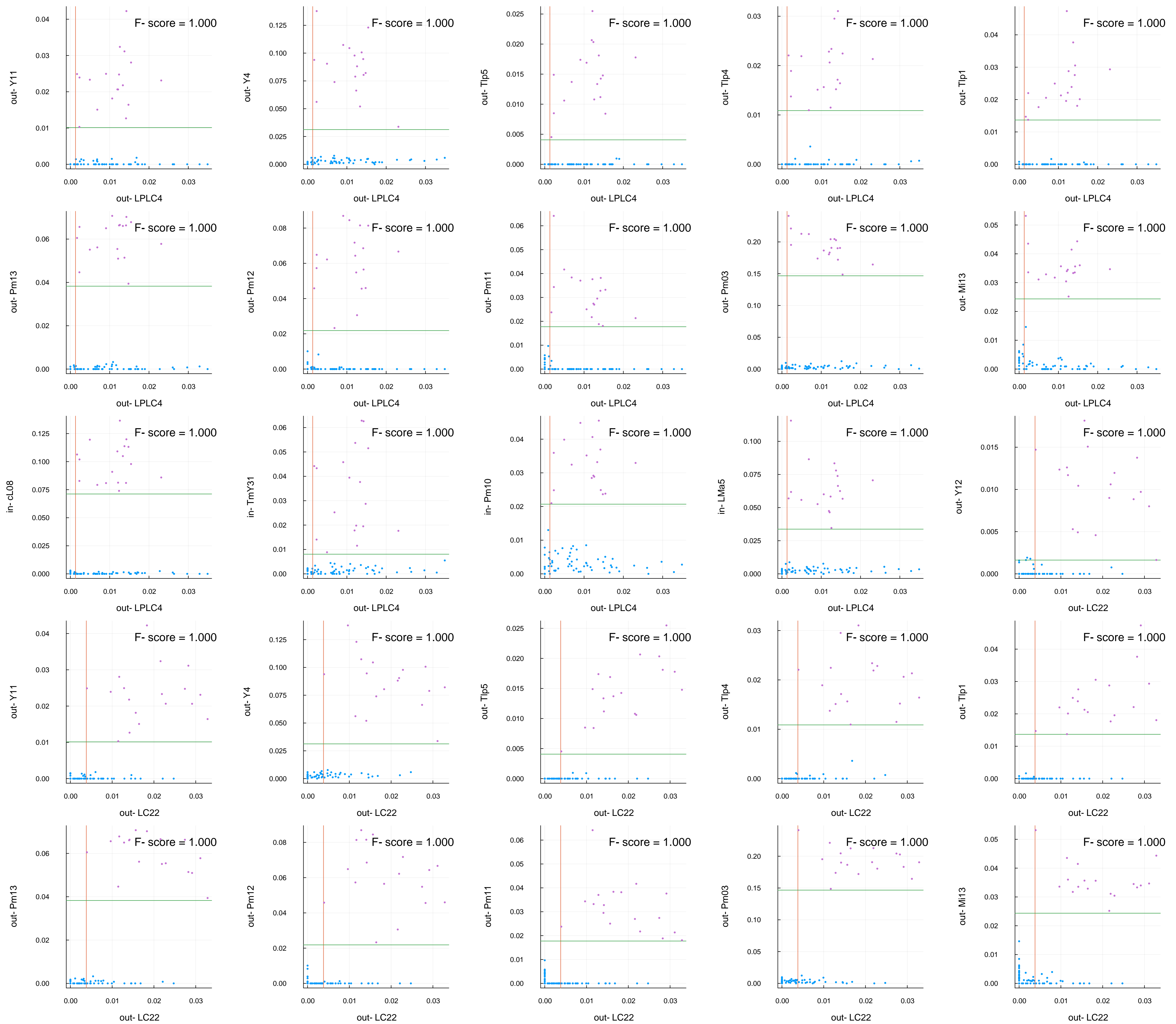

Supplement: Supplementary file 7 — Discriminating 2D projections for neuropil-intrinsic types. For each interneuron type, a pair of features is shown that can be used to discriminate that type from others in the same neuropil. Many although not all discriminations are highly accurate. Both intrinsic and boundary types are included as discriminative features. [file 41586_2024_7981_MOESM7_ESM.zip › DataS3/LMt3.pdf]

## LMt4

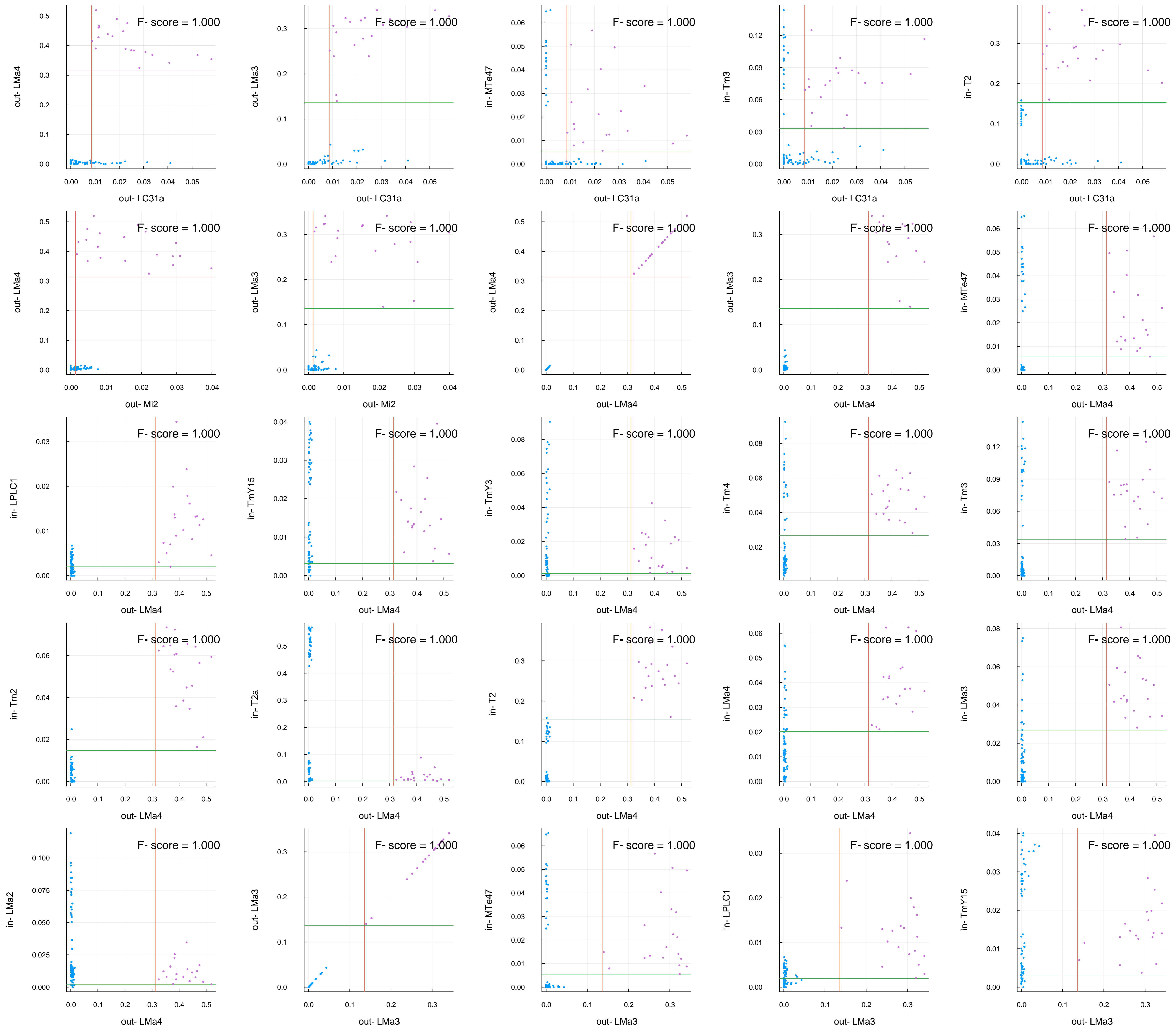

Supplement: Supplementary file 7 — Discriminating 2D projections for neuropil-intrinsic types. For each interneuron type, a pair of features is shown that can be used to discriminate that type from others in the same neuropil. Many although not all discriminations are highly accurate. Both intrinsic and boundary types are included as discriminative features. [file 41586_2024_7981_MOESM7_ESM.zip › DataS3/LMt4.pdf]

LPi01

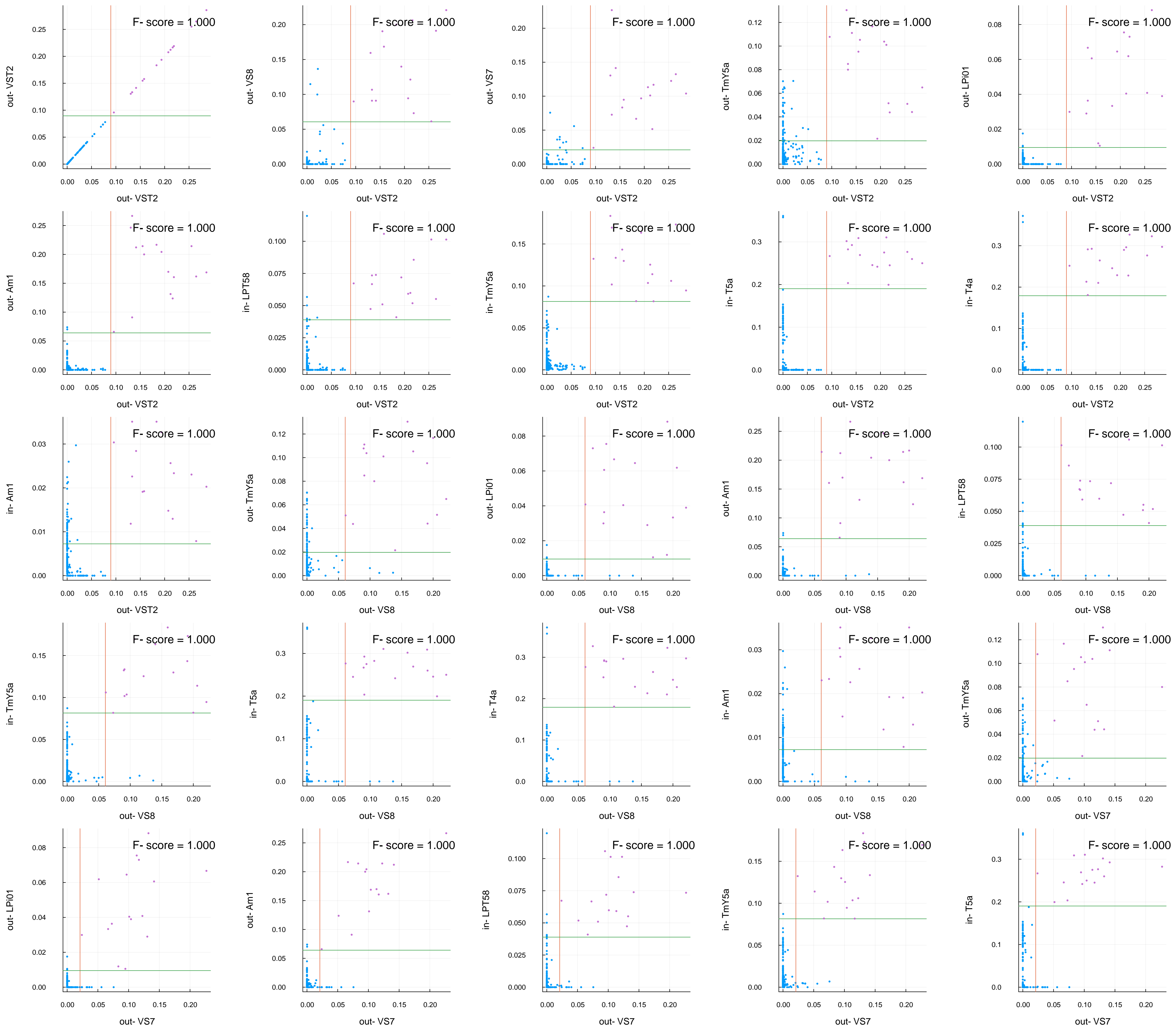

Supplement: Supplementary file 7 — Discriminating 2D projections for neuropil-intrinsic types. For each interneuron type, a pair of features is shown that can be used to discriminate that type from others in the same neuropil. Many although not all discriminations are highly accurate. Both intrinsic and boundary types are included as discriminative features. [file 41586_2024_7981_MOESM7_ESM.zip › DataS3/LPi01.pdf]

LPI02

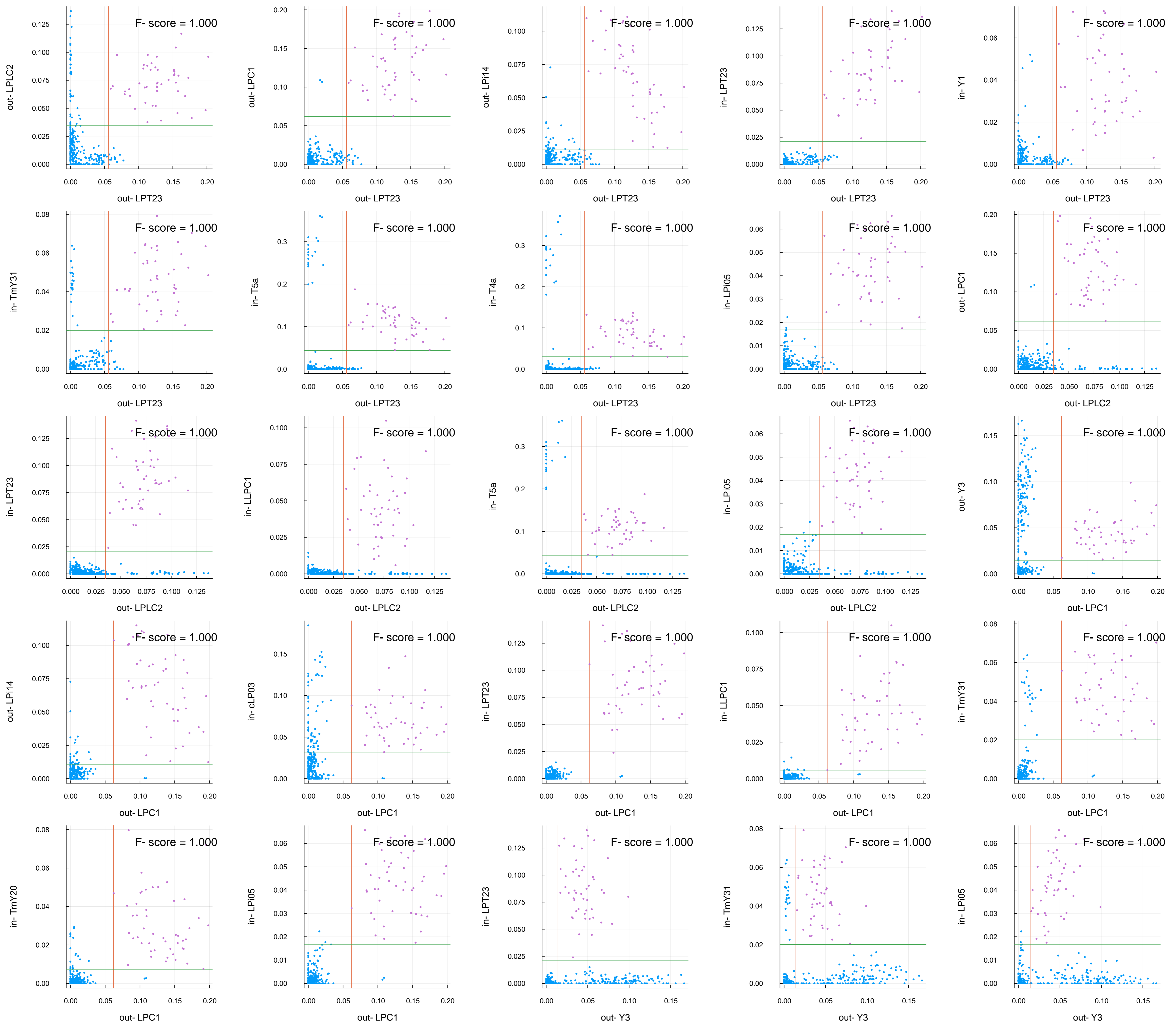

Supplement: Supplementary file 7 — Discriminating 2D projections for neuropil-intrinsic types. For each interneuron type, a pair of features is shown that can be used to discriminate that type from others in the same neuropil. Many although not all discriminations are highly accurate. Both intrinsic and boundary types are included as discriminative features. [file 41586_2024_7981_MOESM7_ESM.zip › DataS3/LPi02.pdf]

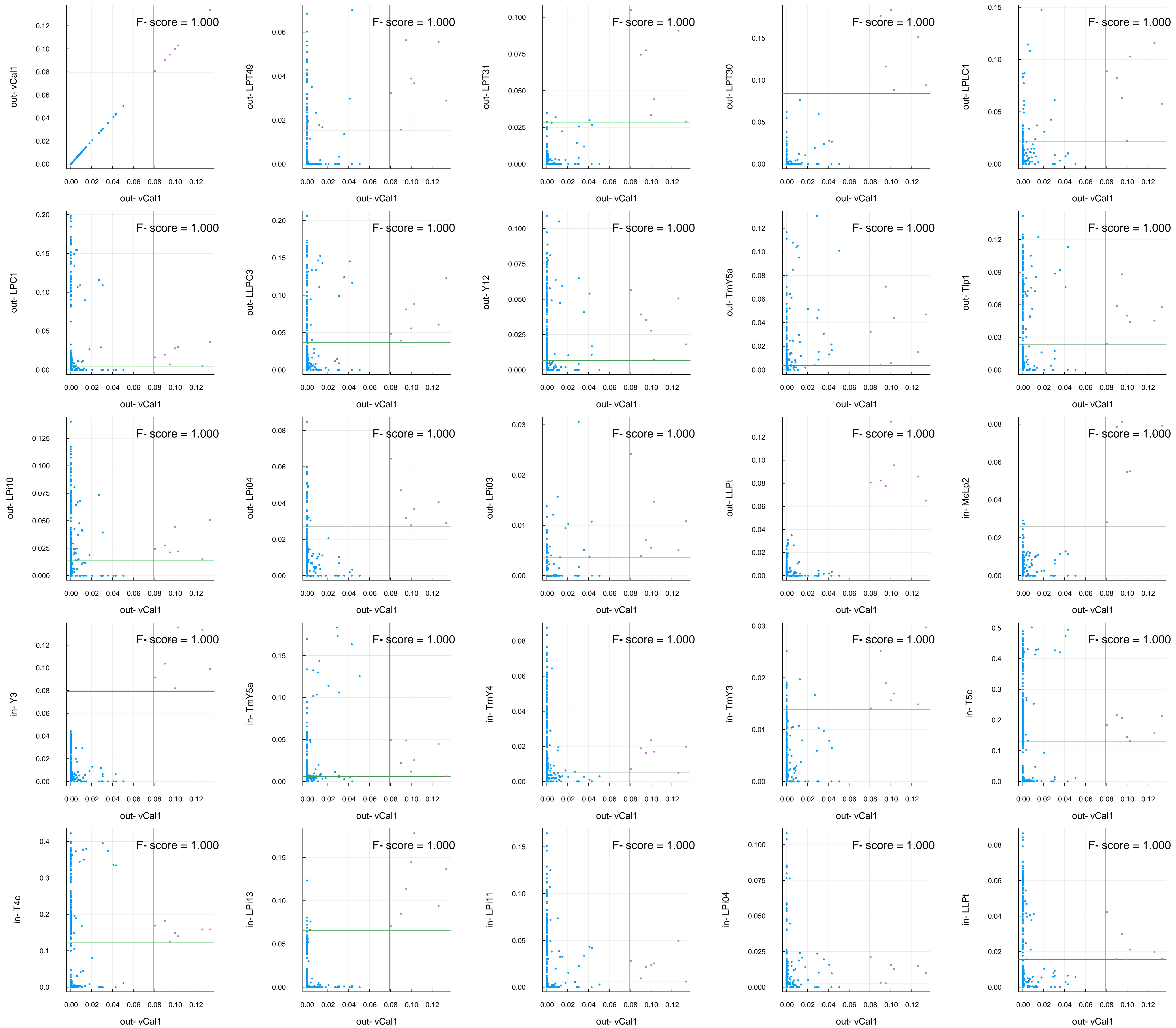

Supplement: Supplementary file 7 — Discriminating 2D projections for neuropil-intrinsic types. For each interneuron type, a pair of features is shown that can be used to discriminate that type from others in the same neuropil. Many although not all discriminations are highly accurate. Both intrinsic and boundary types are included as discriminative features. [file 41586_2024_7981_MOESM7_ESM.zip › DataS3/LPi03.pdf]

LPI04

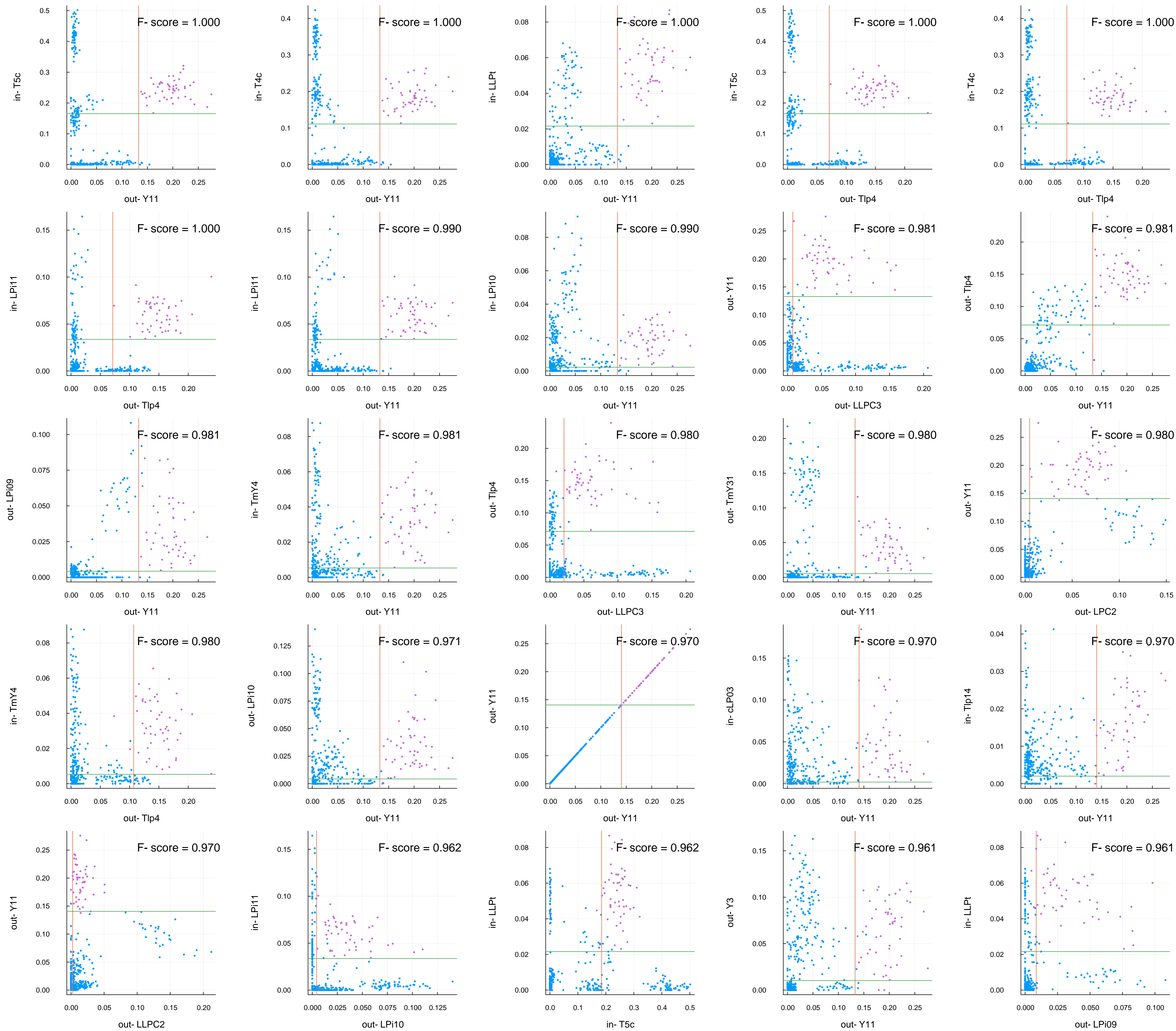

Supplement: Supplementary file 7 — Discriminating 2D projections for neuropil-intrinsic types. For each interneuron type, a pair of features is shown that can be used to discriminate that type from others in the same neuropil. Many although not all discriminations are highly accurate. Both intrinsic and boundary types are included as discriminative features. [file 41586_2024_7981_MOESM7_ESM.zip › DataS3/LPi04.pdf]

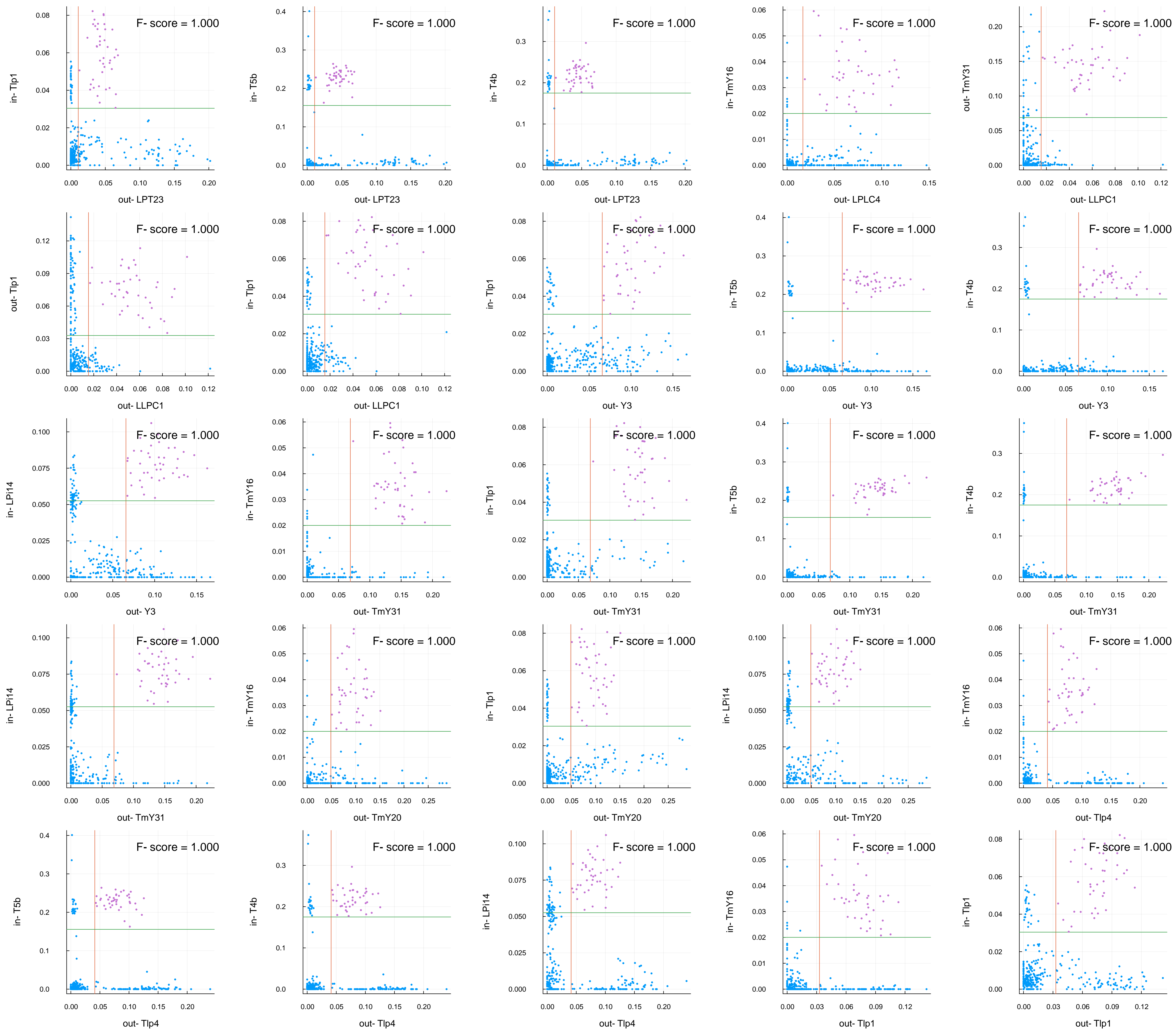

Supplement: Supplementary file 7 — Discriminating 2D projections for neuropil-intrinsic types. For each interneuron type, a pair of features is shown that can be used to discriminate that type from others in the same neuropil. Many although not all discriminations are highly accurate. Both intrinsic and boundary types are included as discriminative features. [file 41586_2024_7981_MOESM7_ESM.zip › DataS3/LPi05.pdf]

LPI06

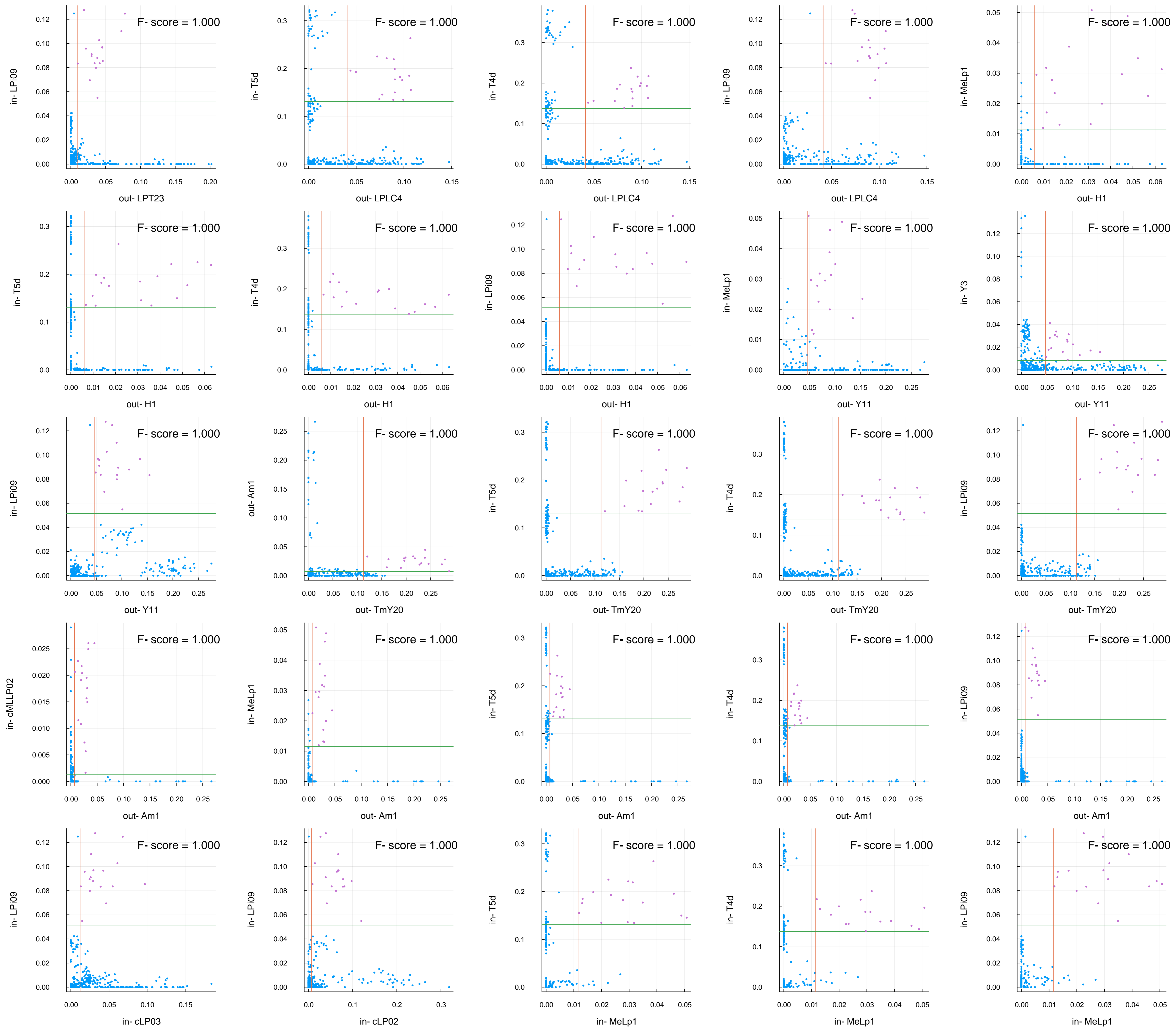

Supplement: Supplementary file 7 — Discriminating 2D projections for neuropil-intrinsic types. For each interneuron type, a pair of features is shown that can be used to discriminate that type from others in the same neuropil. Many although not all discriminations are highly accurate. Both intrinsic and boundary types are included as discriminative features. [file 41586_2024_7981_MOESM7_ESM.zip › DataS3/LPi06.pdf]

## LPi07

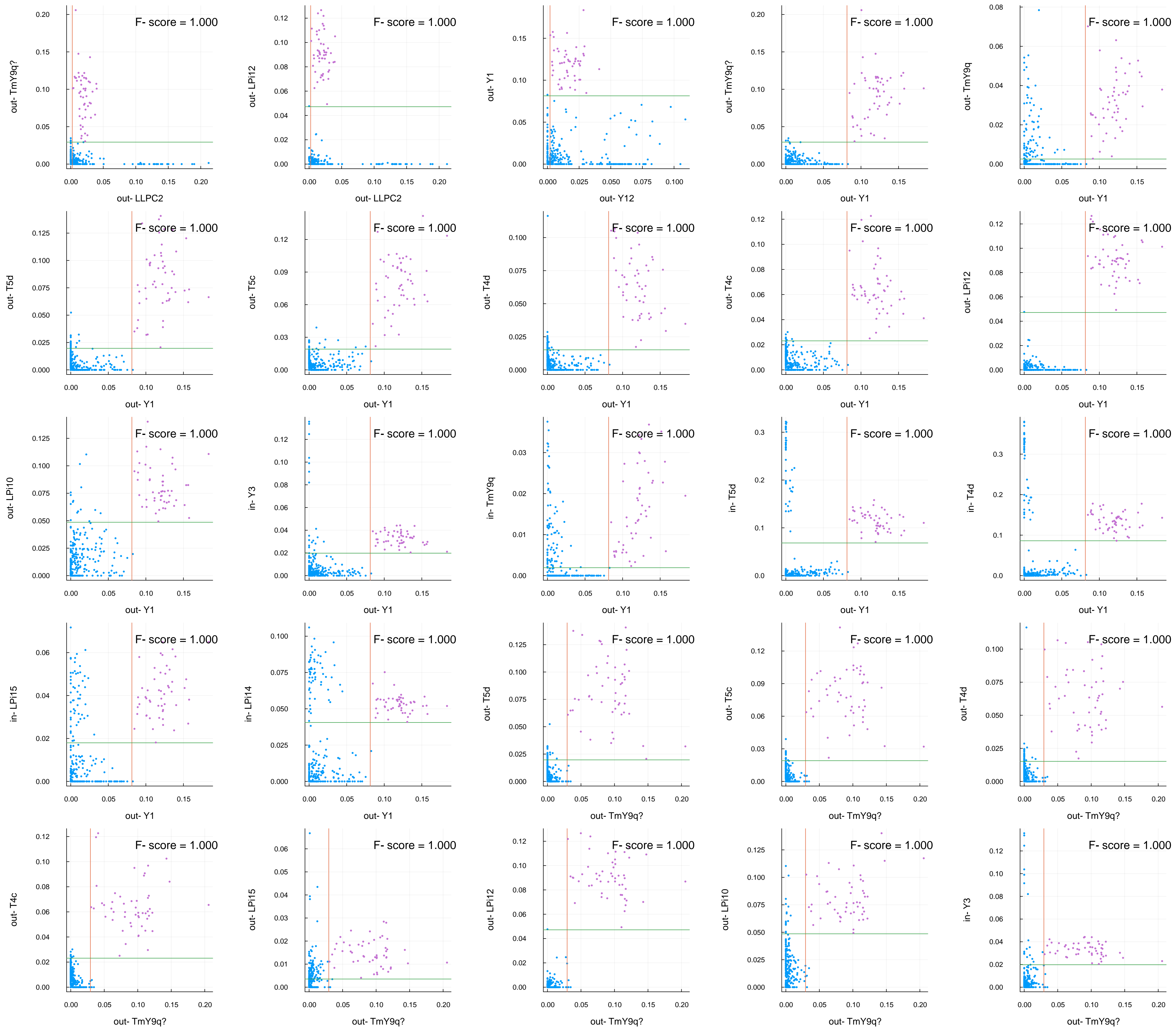

Supplement: Supplementary file 7 — Discriminating 2D projections for neuropil-intrinsic types. For each interneuron type, a pair of features is shown that can be used to discriminate that type from others in the same neuropil. Many although not all discriminations are highly accurate. Both intrinsic and boundary types are included as discriminative features. [file 41586_2024_7981_MOESM7_ESM.zip › DataS3/LPi07.pdf]

LPI08

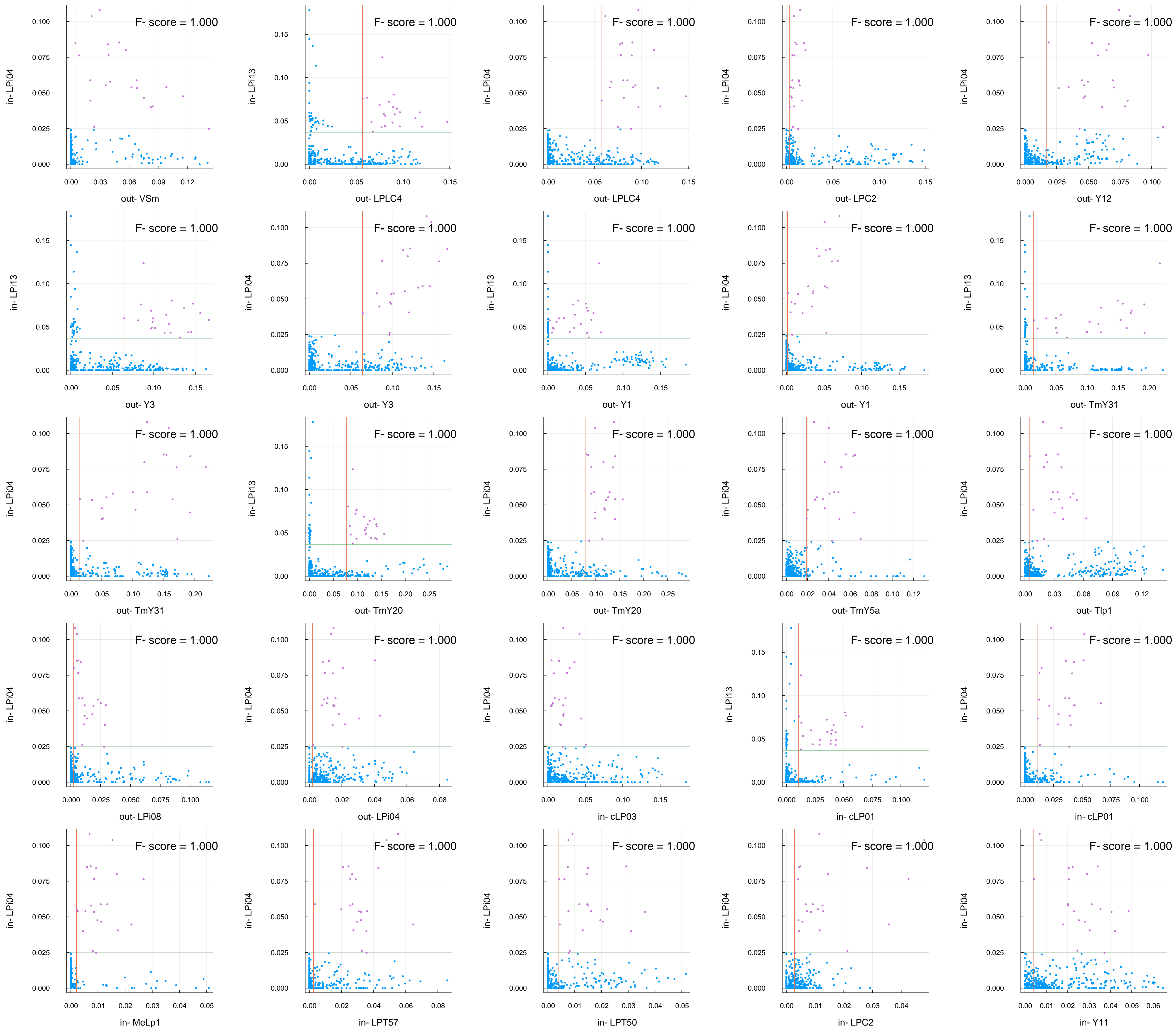

Supplement: Supplementary file 7 — Discriminating 2D projections for neuropil-intrinsic types. For each interneuron type, a pair of features is shown that can be used to discriminate that type from others in the same neuropil. Many although not all discriminations are highly accurate. Both intrinsic and boundary types are included as discriminative features. [file 41586_2024_7981_MOESM7_ESM.zip › DataS3/LPi08.pdf]

LPi09

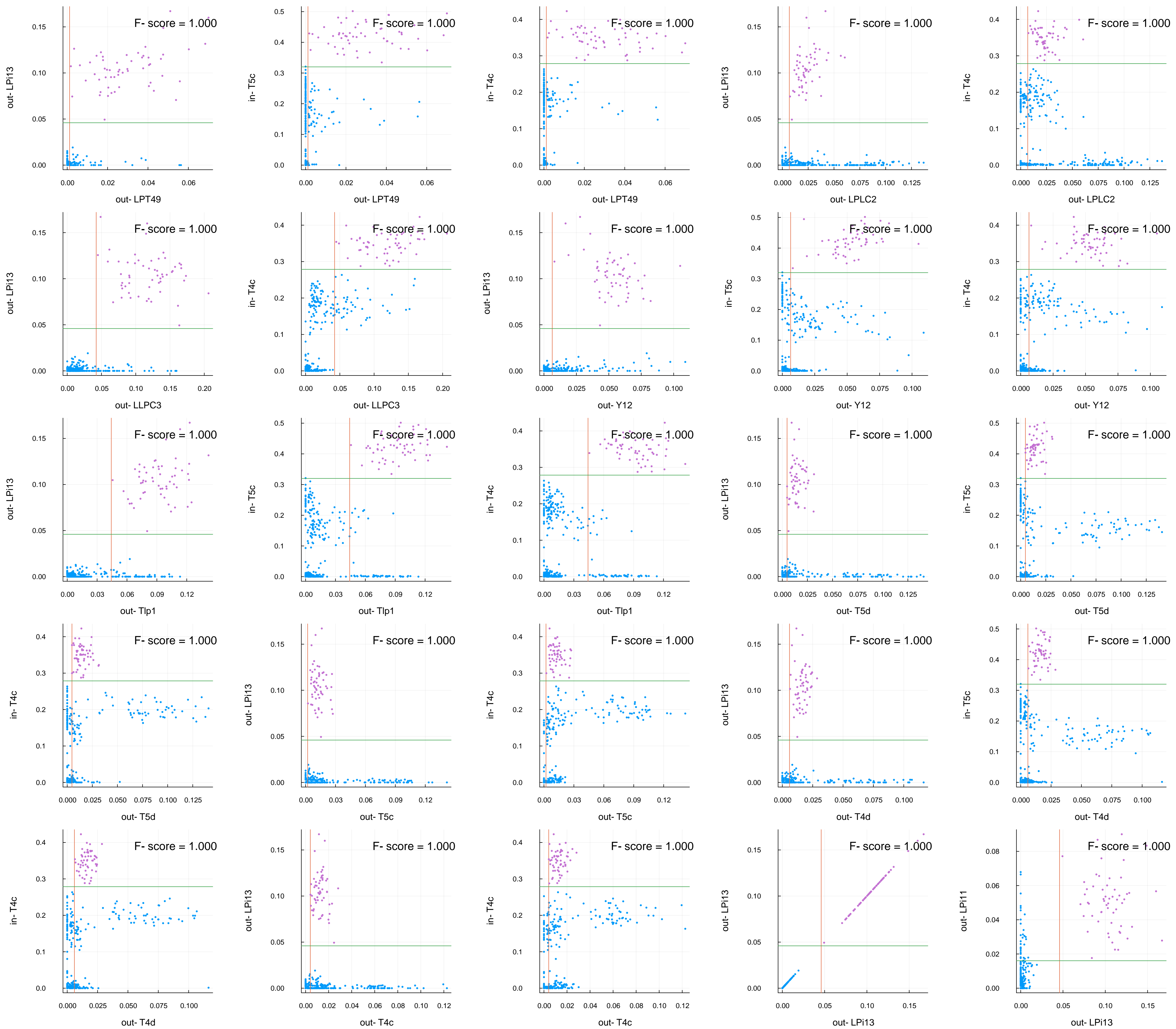

Supplement: Supplementary file 7 — Discriminating 2D projections for neuropil-intrinsic types. For each interneuron type, a pair of features is shown that can be used to discriminate that type from others in the same neuropil. Many although not all discriminations are highly accurate. Both intrinsic and boundary types are included as discriminative features. [file 41586_2024_7981_MOESM7_ESM.zip › DataS3/LPi09.pdf]

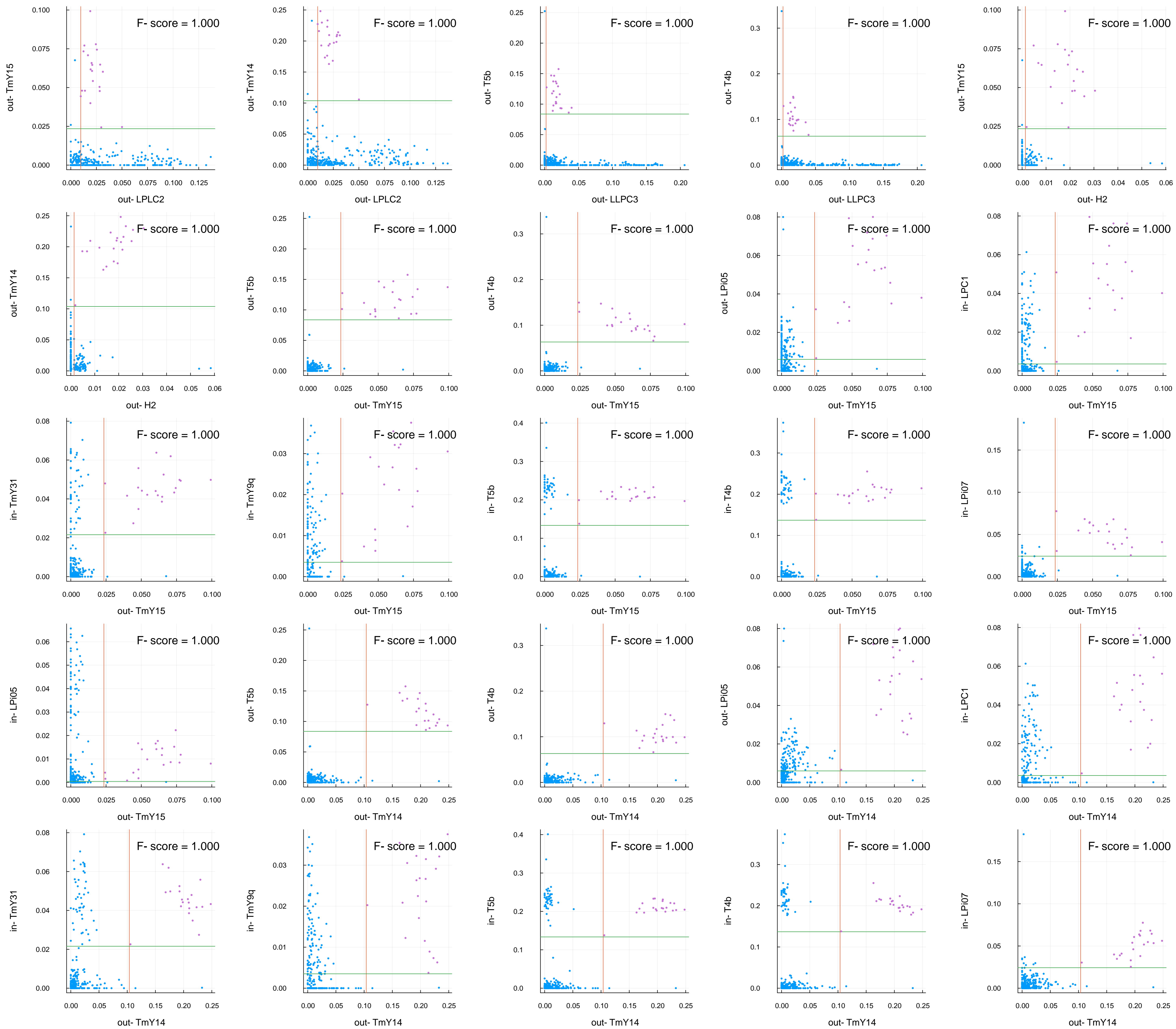

Supplement: Supplementary file 7 — Discriminating 2D projections for neuropil-intrinsic types. For each interneuron type, a pair of features is shown that can be used to discriminate that type from others in the same neuropil. Many although not all discriminations are highly accurate. Both intrinsic and boundary types are included as discriminative features. [file 41586_2024_7981_MOESM7_ESM.zip › DataS3/LPi10.pdf]

LPI11

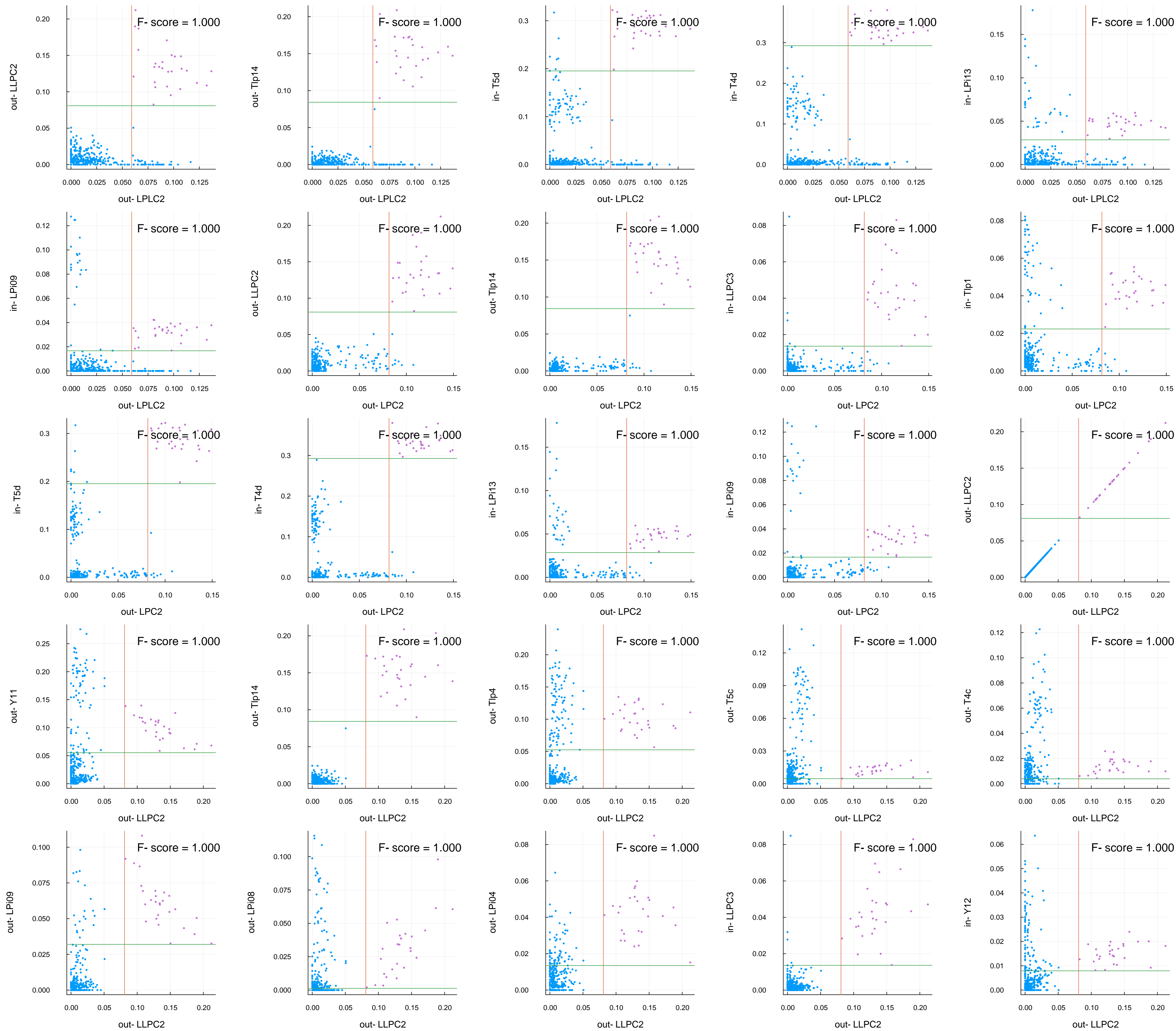

Supplement: Supplementary file 7 — Discriminating 2D projections for neuropil-intrinsic types. For each interneuron type, a pair of features is shown that can be used to discriminate that type from others in the same neuropil. Many although not all discriminations are highly accurate. Both intrinsic and boundary types are included as discriminative features. [file 41586_2024_7981_MOESM7_ESM.zip › DataS3/LPi11.pdf]

LPI12

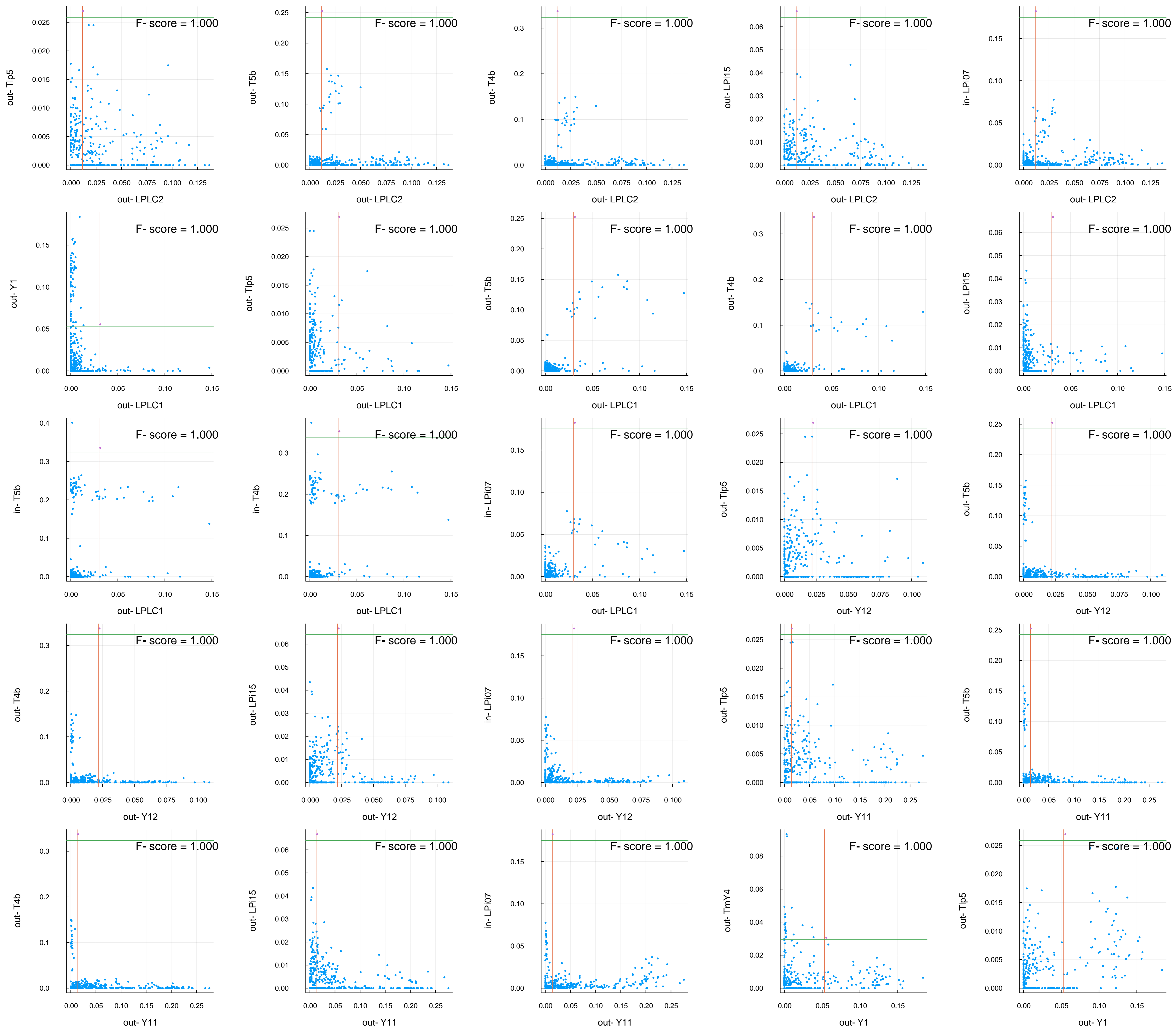

Supplement: Supplementary file 7 — Discriminating 2D projections for neuropil-intrinsic types. For each interneuron type, a pair of features is shown that can be used to discriminate that type from others in the same neuropil. Many although not all discriminations are highly accurate. Both intrinsic and boundary types are included as discriminative features. [file 41586_2024_7981_MOESM7_ESM.zip › DataS3/LPi12.pdf]

LPI13

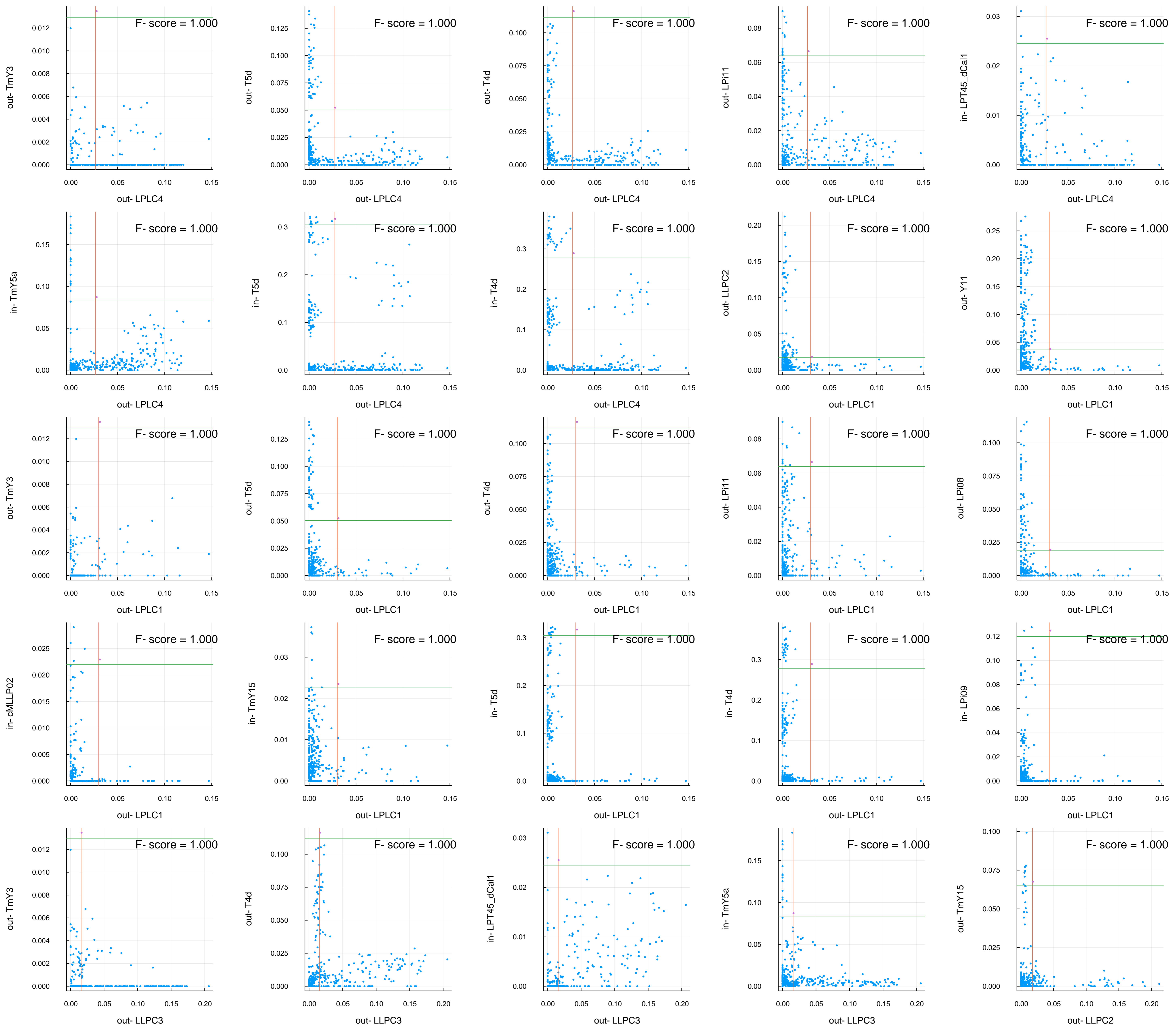

Supplement: Supplementary file 7 — Discriminating 2D projections for neuropil-intrinsic types. For each interneuron type, a pair of features is shown that can be used to discriminate that type from others in the same neuropil. Many although not all discriminations are highly accurate. Both intrinsic and boundary types are included as discriminative features. [file 41586_2024_7981_MOESM7_ESM.zip › DataS3/LPi13.pdf]

LPI14

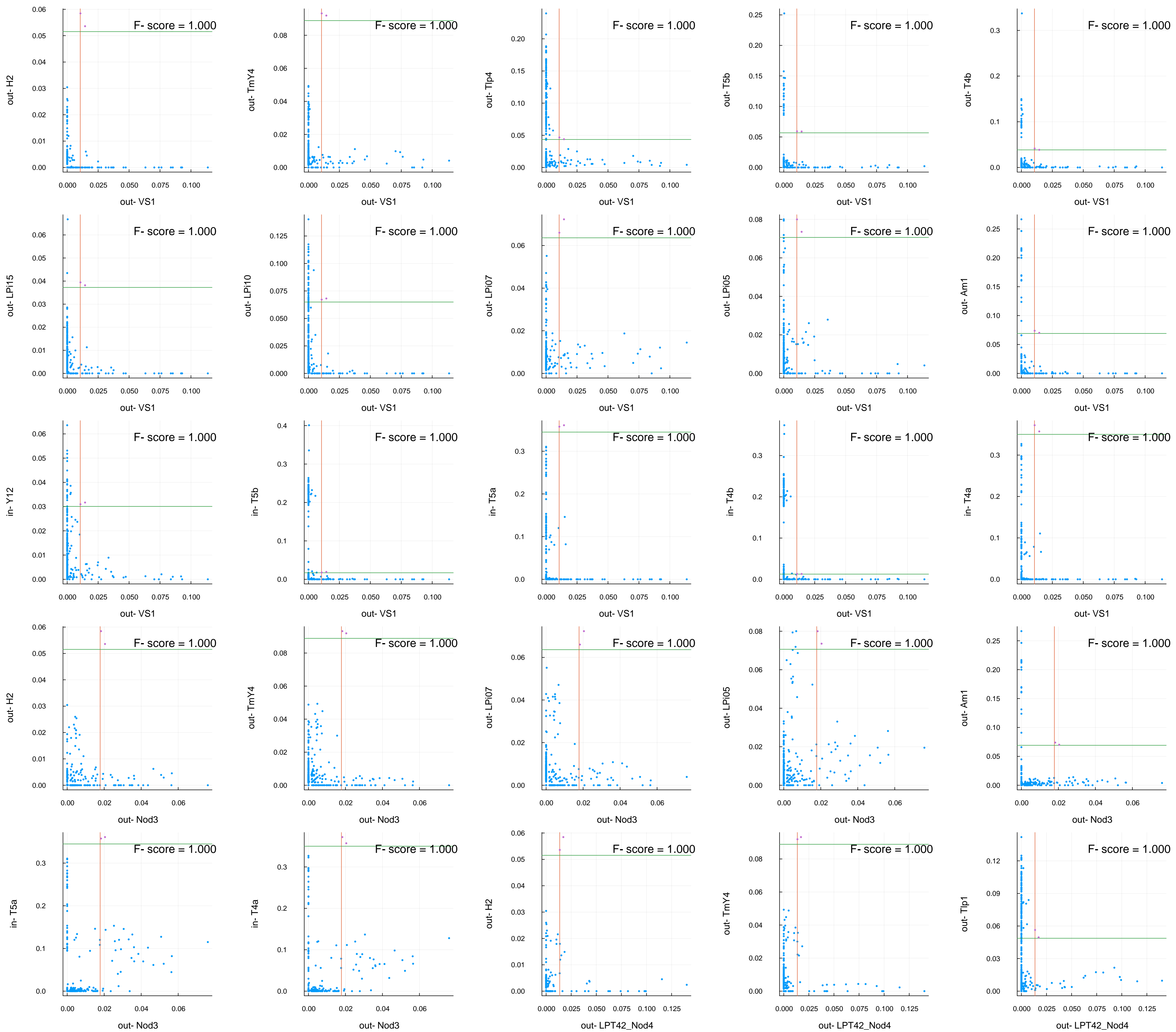

Supplement: Supplementary file 7 — Discriminating 2D projections for neuropil-intrinsic types. For each interneuron type, a pair of features is shown that can be used to discriminate that type from others in the same neuropil. Many although not all discriminations are highly accurate. Both intrinsic and boundary types are included as discriminative features. [file 41586_2024_7981_MOESM7_ESM.zip › DataS3/LPi14.pdf]

# LPi15

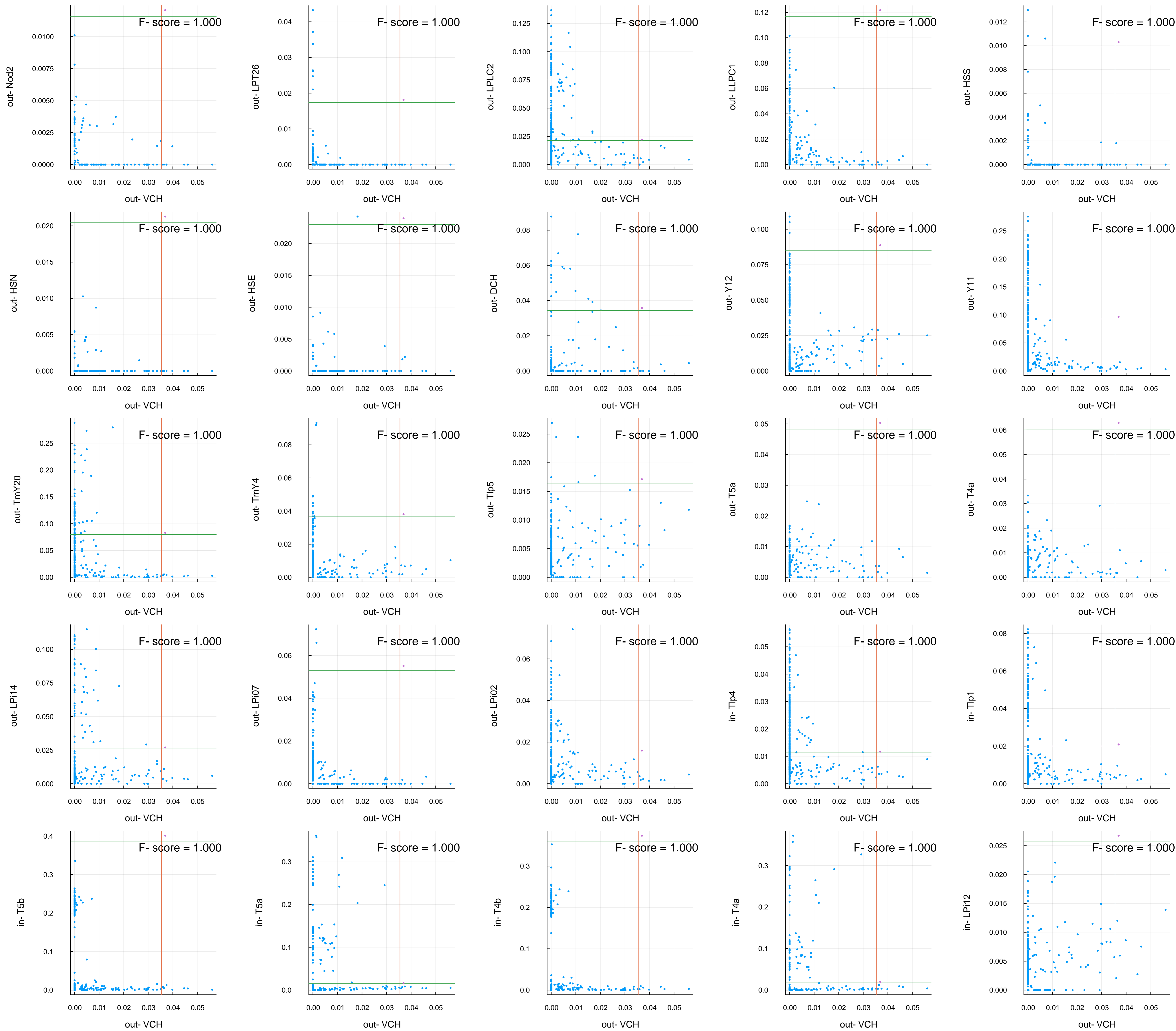

Supplement: Supplementary file 7 — Discriminating 2D projections for neuropil-intrinsic types. For each interneuron type, a pair of features is shown that can be used to discriminate that type from others in the same neuropil. Many although not all discriminations are highly accurate. Both intrinsic and boundary types are included as discriminative features. [file 41586_2024_7981_MOESM7_ESM.zip › DataS3/LPi15.pdf]

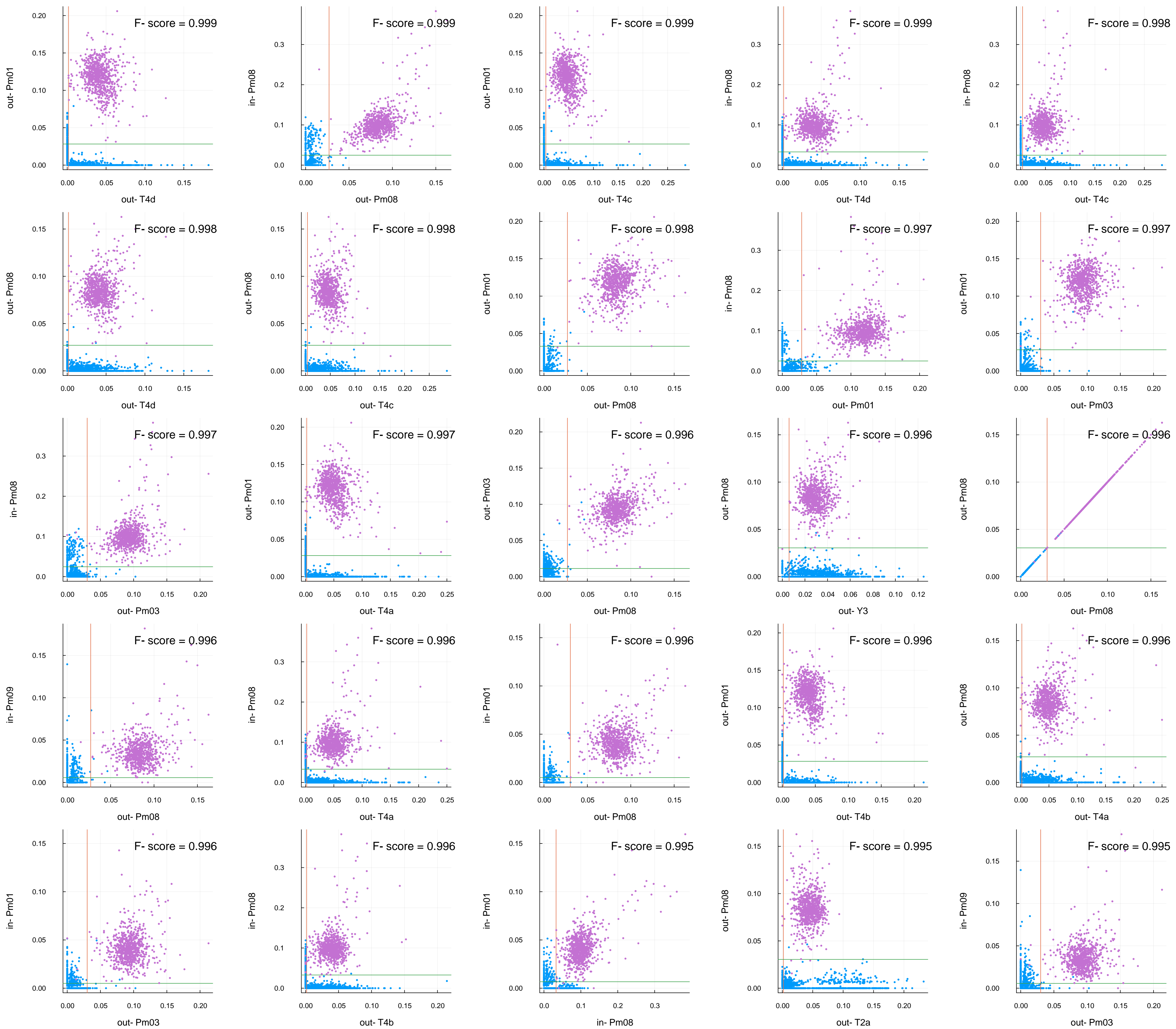

Supplement: Supplementary file 7 — Discriminating 2D projections for neuropil-intrinsic types. For each interneuron type, a pair of features is shown that can be used to discriminate that type from others in the same neuropil. Many although not all discriminations are highly accurate. Both intrinsic and boundary types are included as discriminative features. [file 41586_2024_7981_MOESM7_ESM.zip › DataS3/Mi1.pdf]

# Mi10

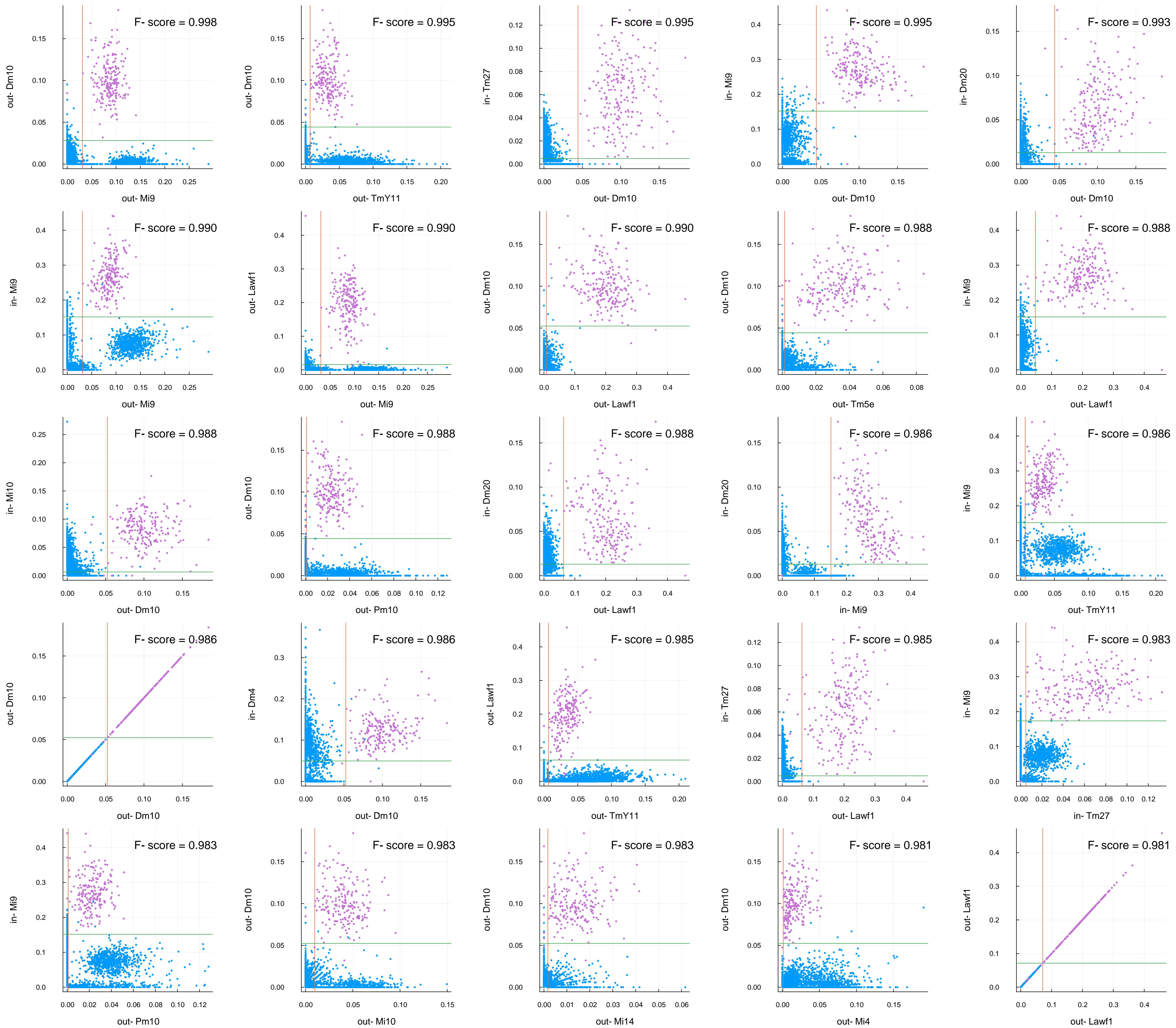

Supplement: Supplementary file 7 — Discriminating 2D projections for neuropil-intrinsic types. For each interneuron type, a pair of features is shown that can be used to discriminate that type from others in the same neuropil. Many although not all discriminations are highly accurate. Both intrinsic and boundary types are included as discriminative features. [file 41586_2024_7981_MOESM7_ESM.zip › DataS3/Mi10.pdf]

Mi13

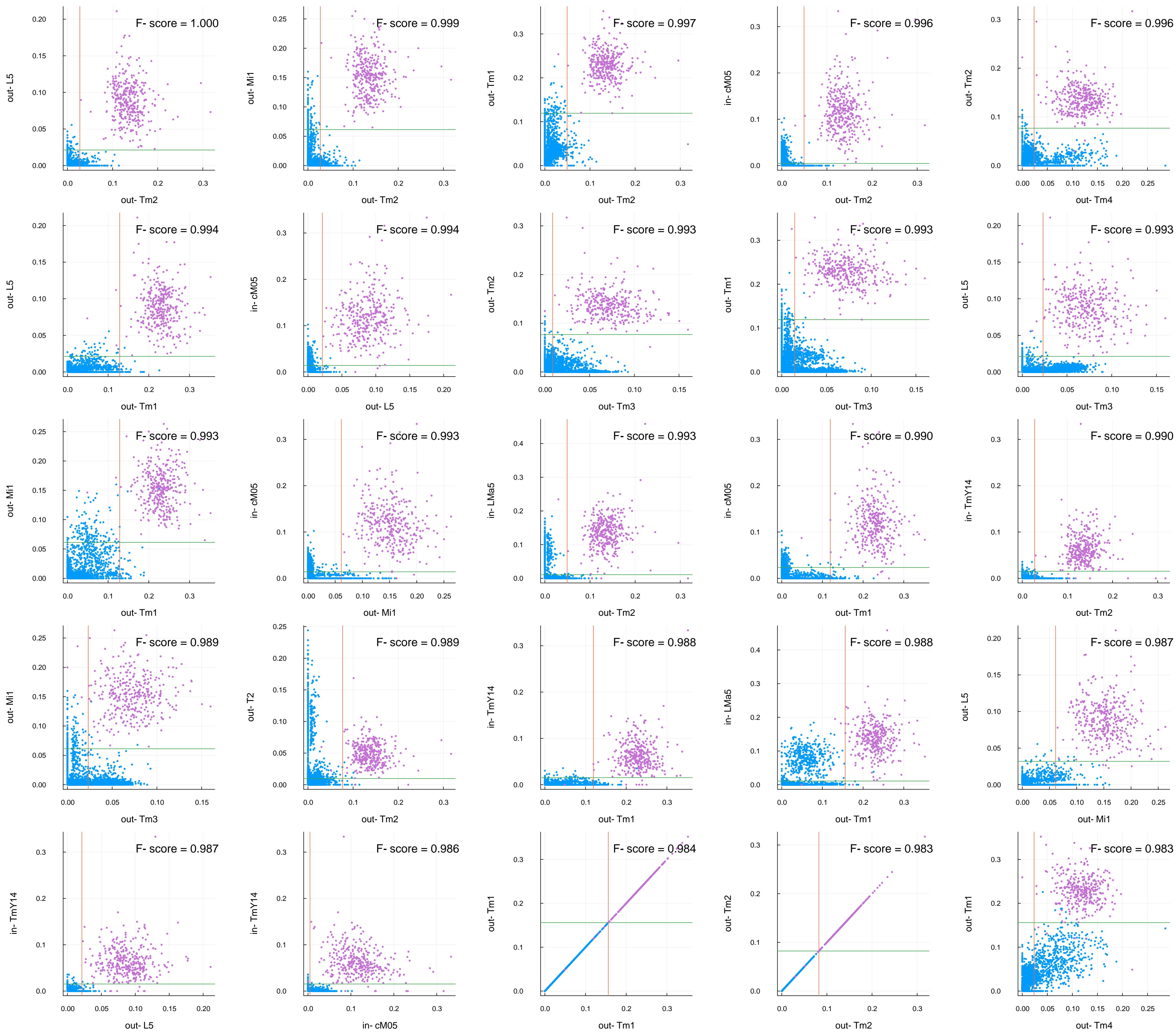

Supplement: Supplementary file 7 — Discriminating 2D projections for neuropil-intrinsic types. For each interneuron type, a pair of features is shown that can be used to discriminate that type from others in the same neuropil. Many although not all discriminations are highly accurate. Both intrinsic and boundary types are included as discriminative features. [file 41586_2024_7981_MOESM7_ESM.zip › DataS3/Mi13.pdf]

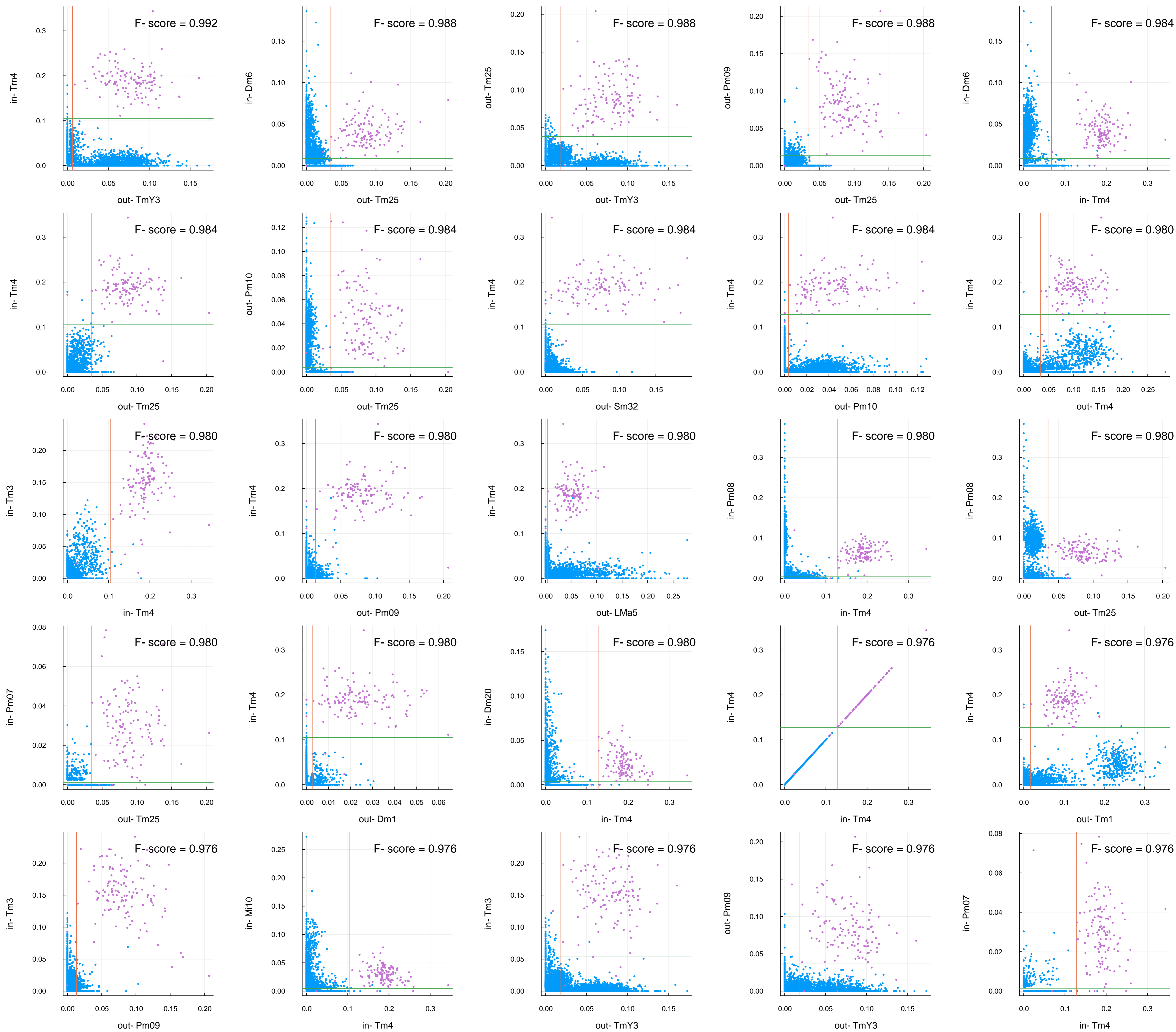

Supplement: Supplementary file 7 — Discriminating 2D projections for neuropil-intrinsic types. For each interneuron type, a pair of features is shown that can be used to discriminate that type from others in the same neuropil. Many although not all discriminations are highly accurate. Both intrinsic and boundary types are included as discriminative features. [file 41586_2024_7981_MOESM7_ESM.zip › DataS3/Mi14.pdf]

# Mi15

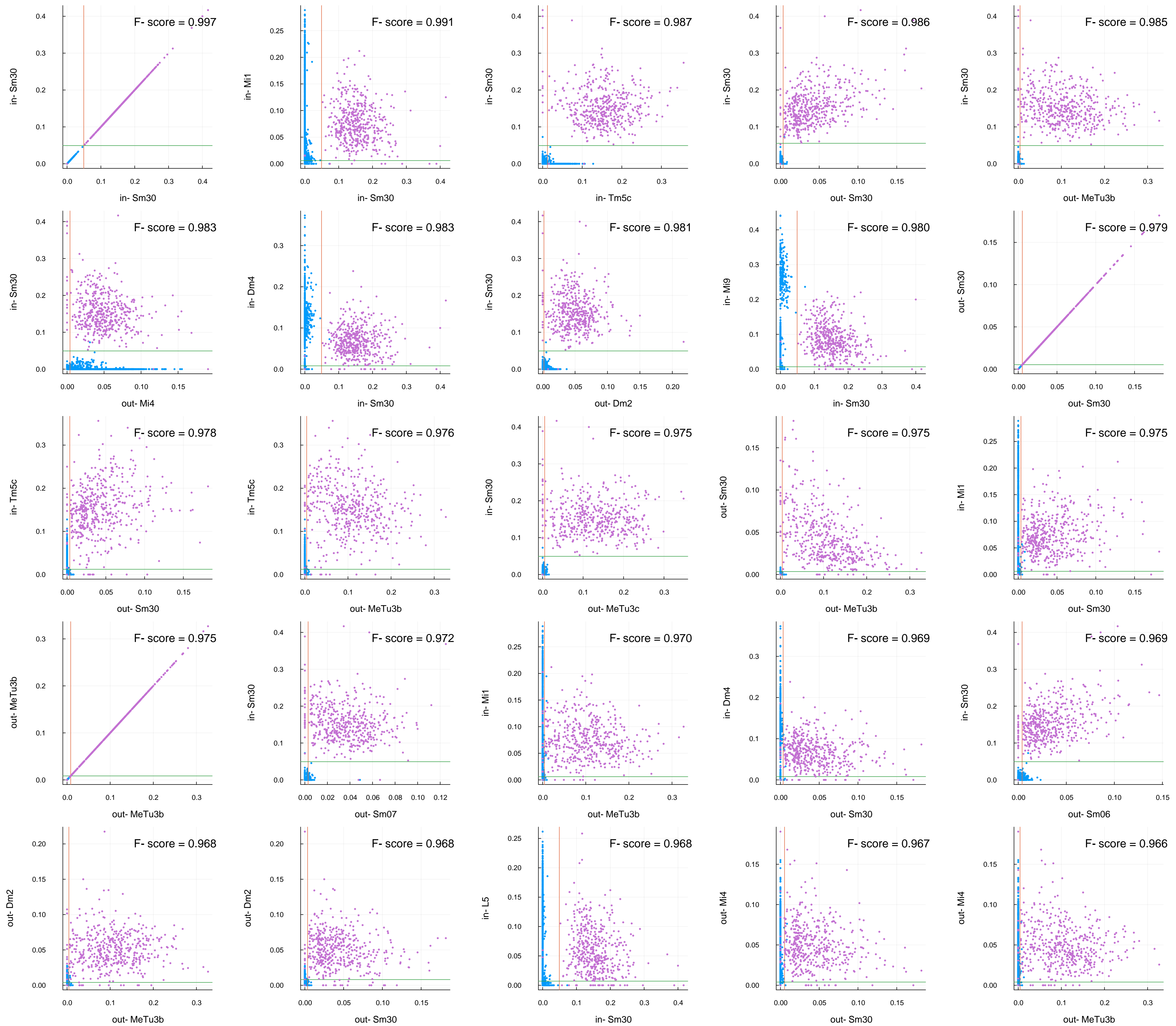

Supplement: Supplementary file 7 — Discriminating 2D projections for neuropil-intrinsic types. For each interneuron type, a pair of features is shown that can be used to discriminate that type from others in the same neuropil. Many although not all discriminations are highly accurate. Both intrinsic and boundary types are included as discriminative features. [file 41586_2024_7981_MOESM7_ESM.zip › DataS3/Mi15.pdf]

Mi2

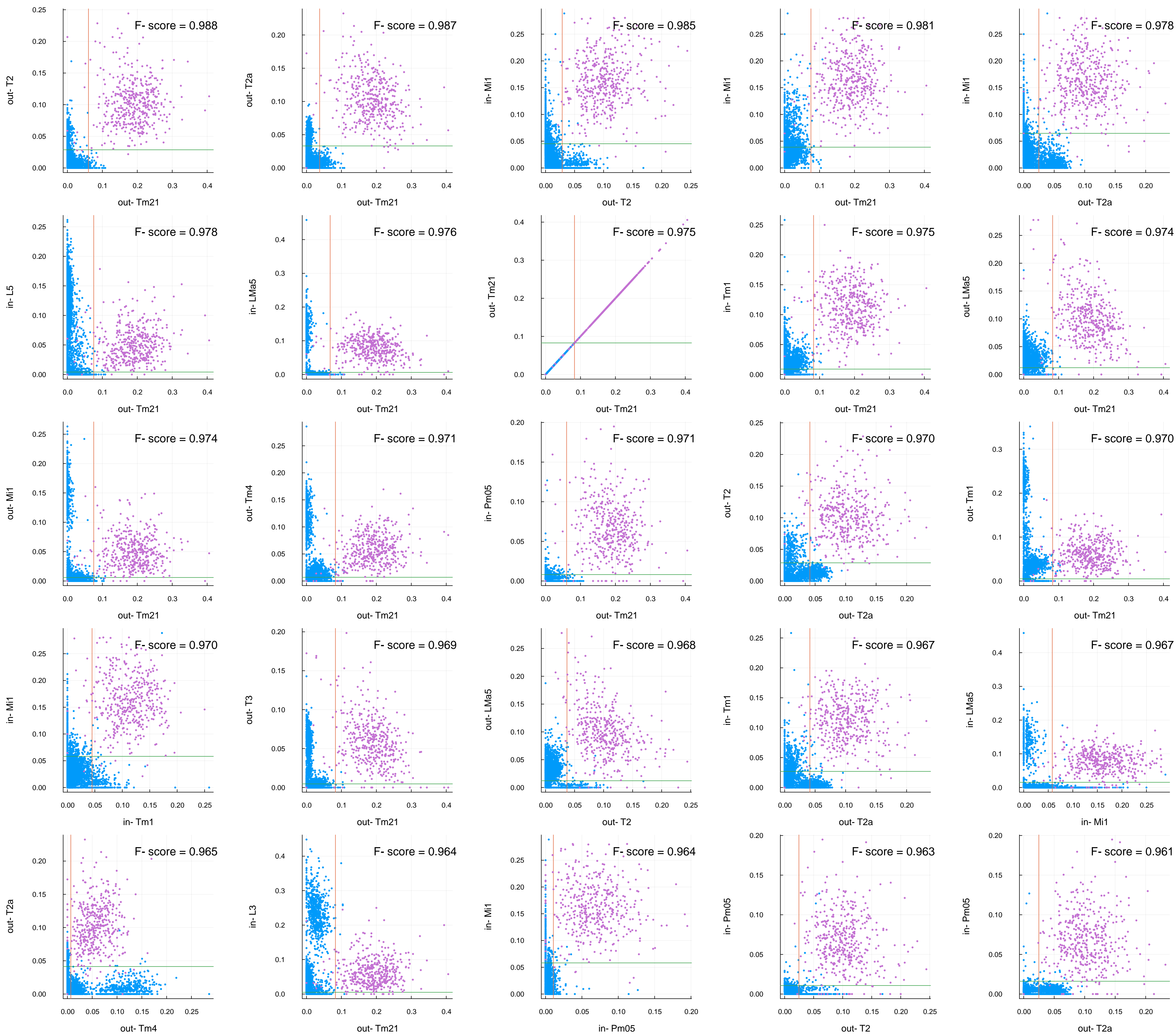

Supplement: Supplementary file 7 — Discriminating 2D projections for neuropil-intrinsic types. For each interneuron type, a pair of features is shown that can be used to discriminate that type from others in the same neuropil. Many although not all discriminations are highly accurate. Both intrinsic and boundary types are included as discriminative features. [file 41586_2024_7981_MOESM7_ESM.zip › DataS3/Mi2.pdf]

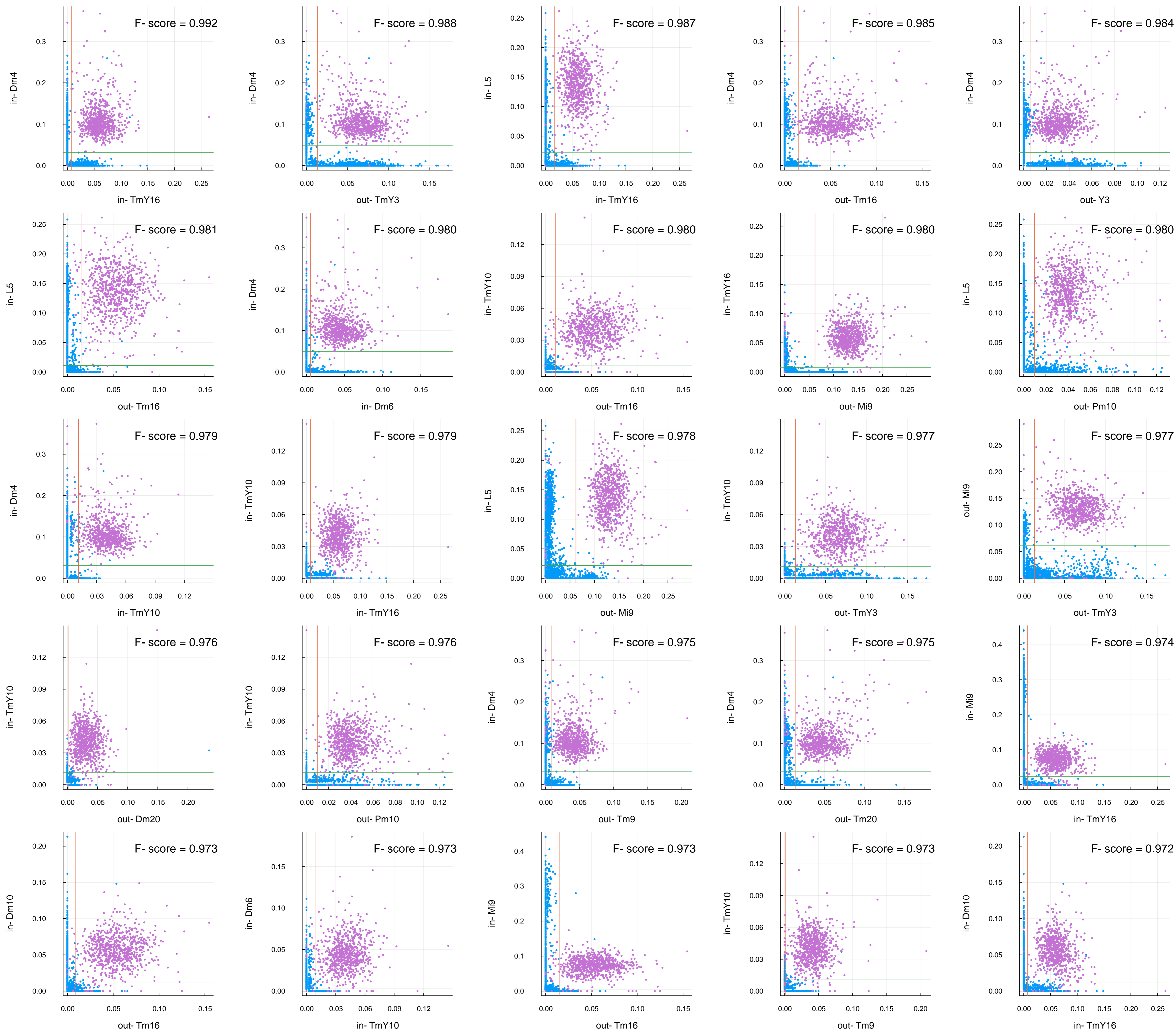

Supplement: Supplementary file 7 — Discriminating 2D projections for neuropil-intrinsic types. For each interneuron type, a pair of features is shown that can be used to discriminate that type from others in the same neuropil. Many although not all discriminations are highly accurate. Both intrinsic and boundary types are included as discriminative features. [file 41586_2024_7981_MOESM7_ESM.zip › DataS3/Mi4.pdf]

Mi9

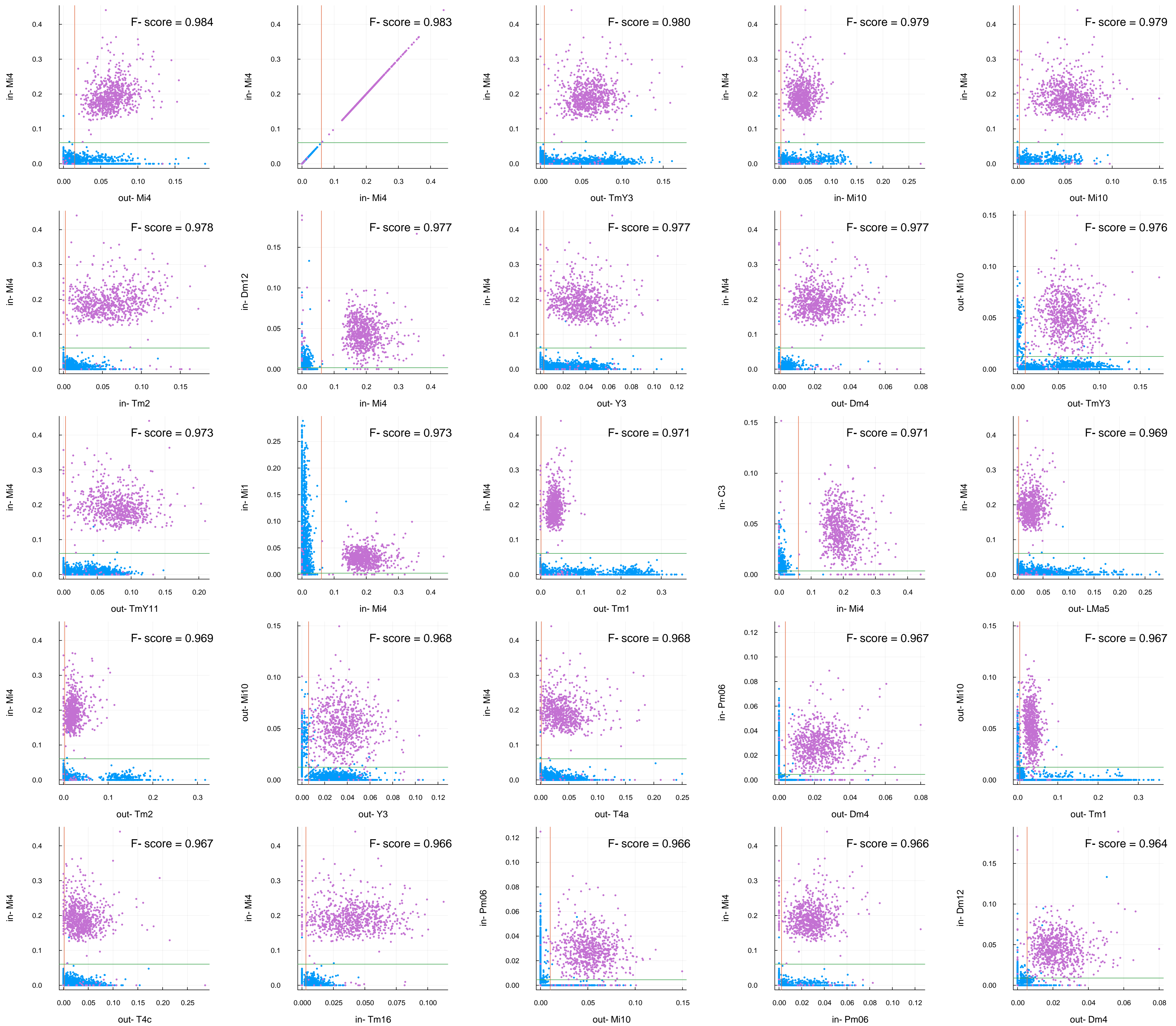

Supplement: Supplementary file 7 — Discriminating 2D projections for neuropil-intrinsic types. For each interneuron type, a pair of features is shown that can be used to discriminate that type from others in the same neuropil. Many although not all discriminations are highly accurate. Both intrinsic and boundary types are included as discriminative features. [file 41586_2024_7981_MOESM7_ESM.zip › DataS3/Mi9.pdf]

MLt1

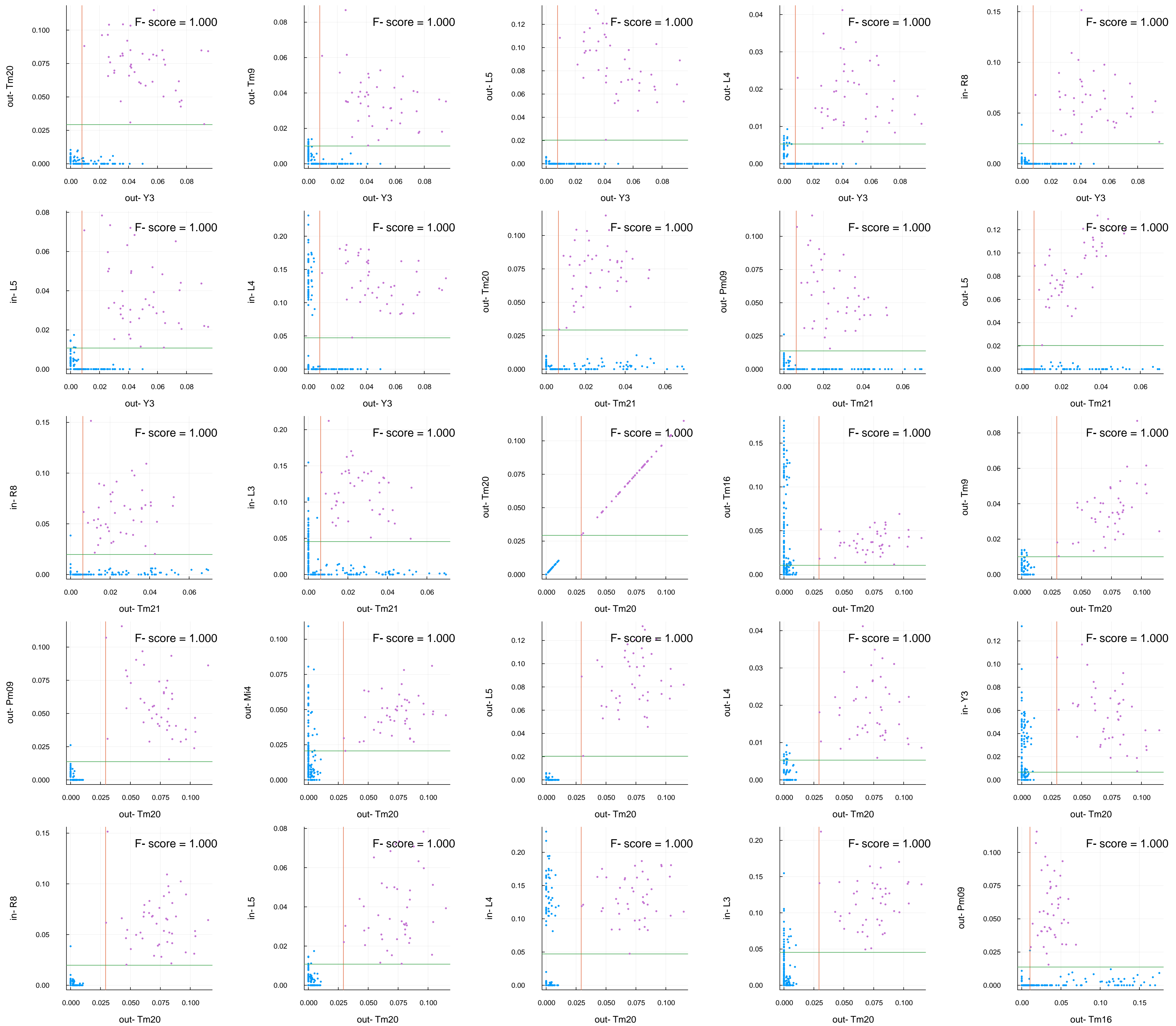

Supplement: Supplementary file 7 — Discriminating 2D projections for neuropil-intrinsic types. For each interneuron type, a pair of features is shown that can be used to discriminate that type from others in the same neuropil. Many although not all discriminations are highly accurate. Both intrinsic and boundary types are included as discriminative features. [file 41586_2024_7981_MOESM7_ESM.zip › DataS3/MLt1.pdf]

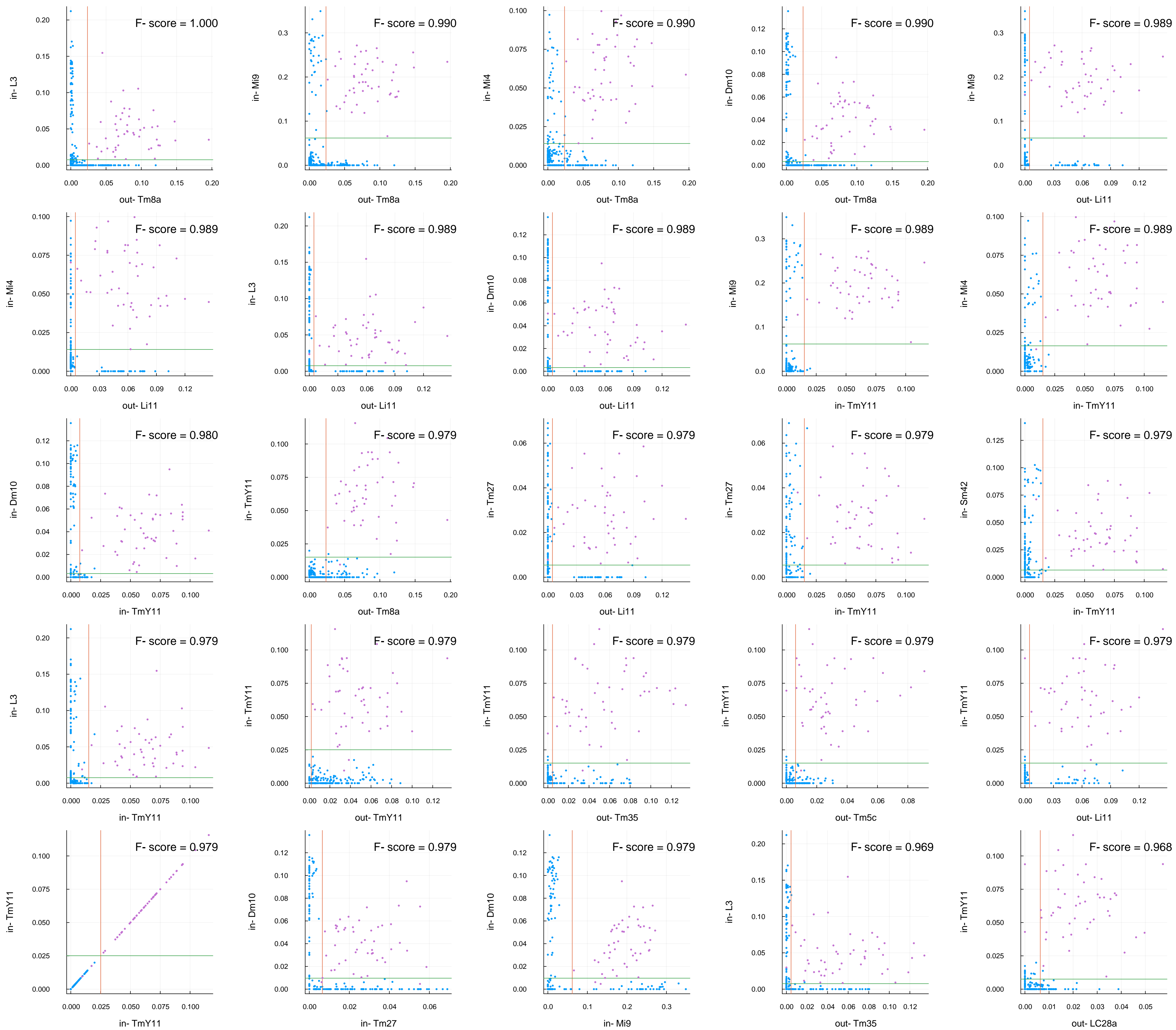

Supplement: Supplementary file 7 — Discriminating 2D projections for neuropil-intrinsic types. For each interneuron type, a pair of features is shown that can be used to discriminate that type from others in the same neuropil. Many although not all discriminations are highly accurate. Both intrinsic and boundary types are included as discriminative features. [file 41586_2024_7981_MOESM7_ESM.zip › DataS3/MLt2.pdf]

MLt3

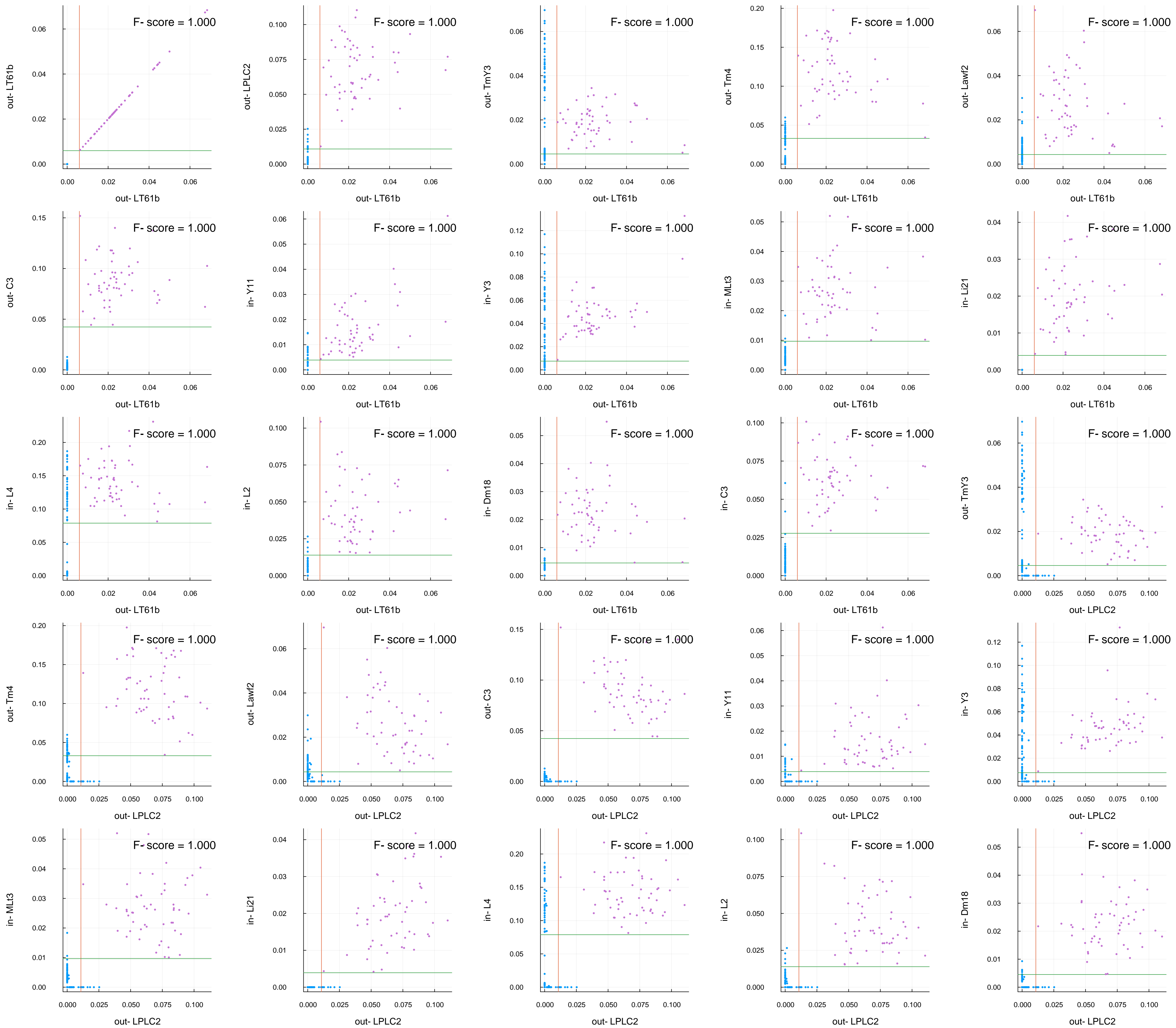

Supplement: Supplementary file 7 — Discriminating 2D projections for neuropil-intrinsic types. For each interneuron type, a pair of features is shown that can be used to discriminate that type from others in the same neuropil. Many although not all discriminations are highly accurate. Both intrinsic and boundary types are included as discriminative features. [file 41586_2024_7981_MOESM7_ESM.zip › DataS3/MLt3.pdf]

MLt4

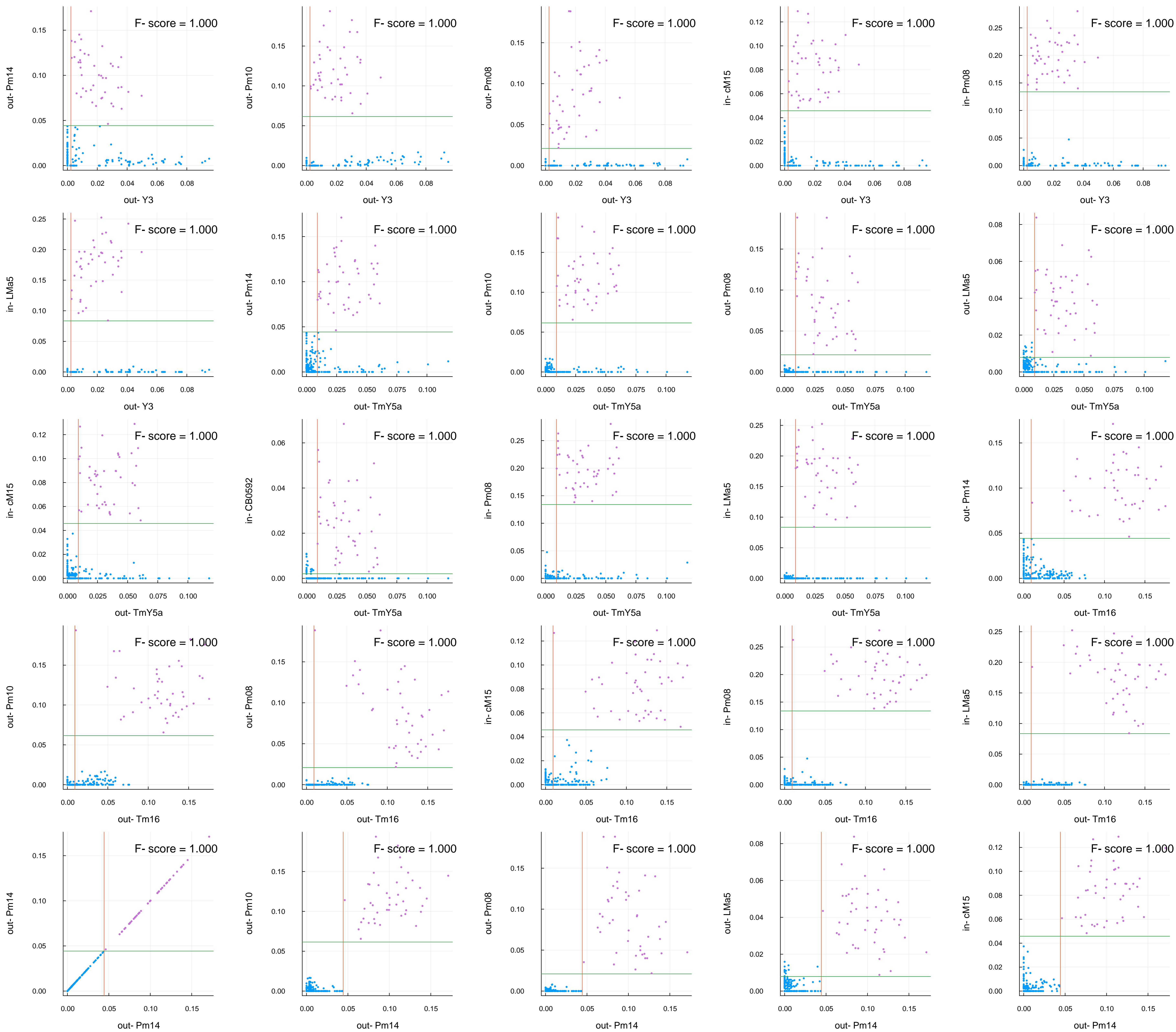

Supplement: Supplementary file 7 — Discriminating 2D projections for neuropil-intrinsic types. For each interneuron type, a pair of features is shown that can be used to discriminate that type from others in the same neuropil. Many although not all discriminations are highly accurate. Both intrinsic and boundary types are included as discriminative features. [file 41586_2024_7981_MOESM7_ESM.zip › DataS3/MLt4.pdf]

MLt5

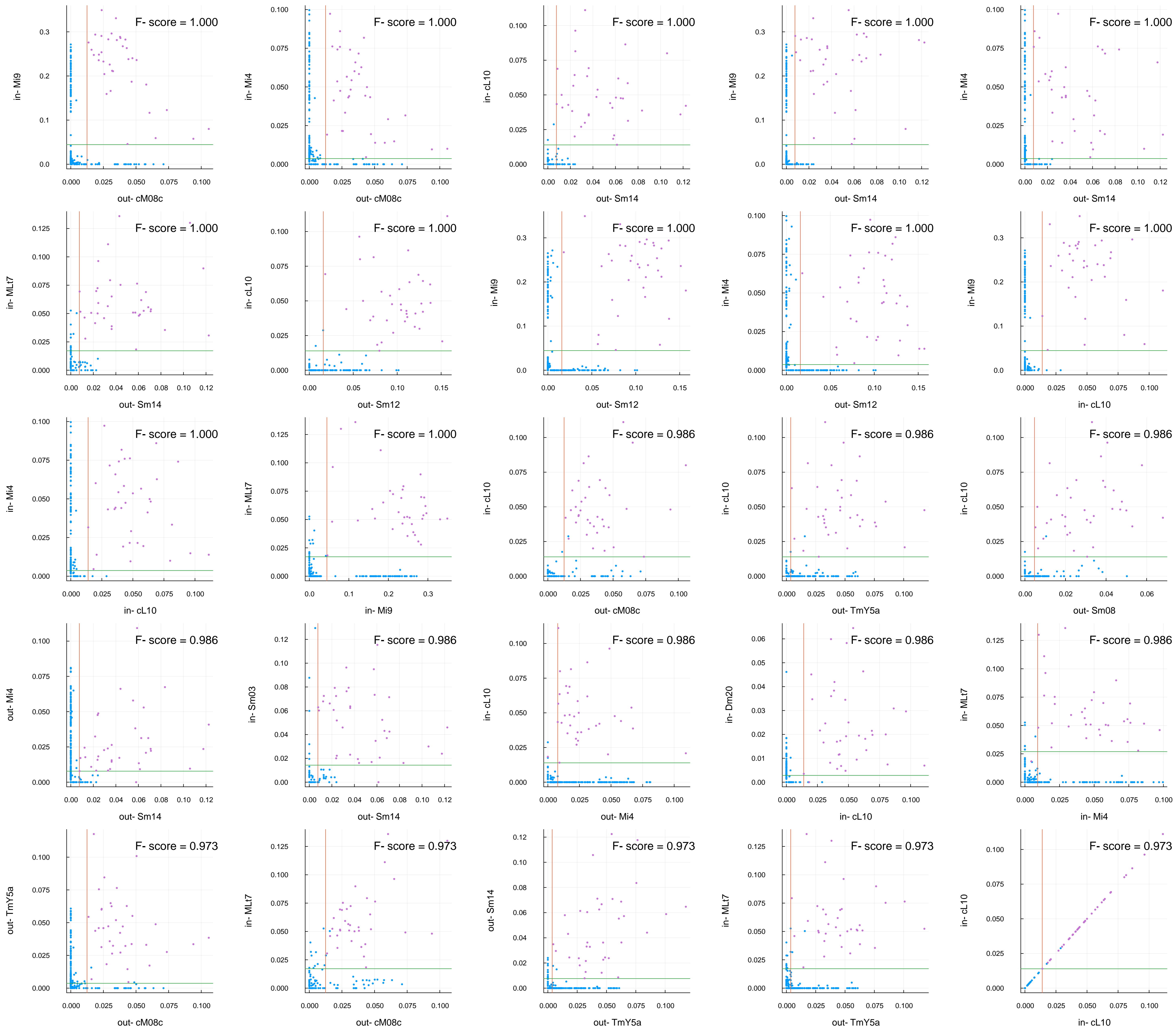

Supplement: Supplementary file 7 — Discriminating 2D projections for neuropil-intrinsic types. For each interneuron type, a pair of features is shown that can be used to discriminate that type from others in the same neuropil. Many although not all discriminations are highly accurate. Both intrinsic and boundary types are included as discriminative features. [file 41586_2024_7981_MOESM7_ESM.zip › DataS3/MLt5.pdf]

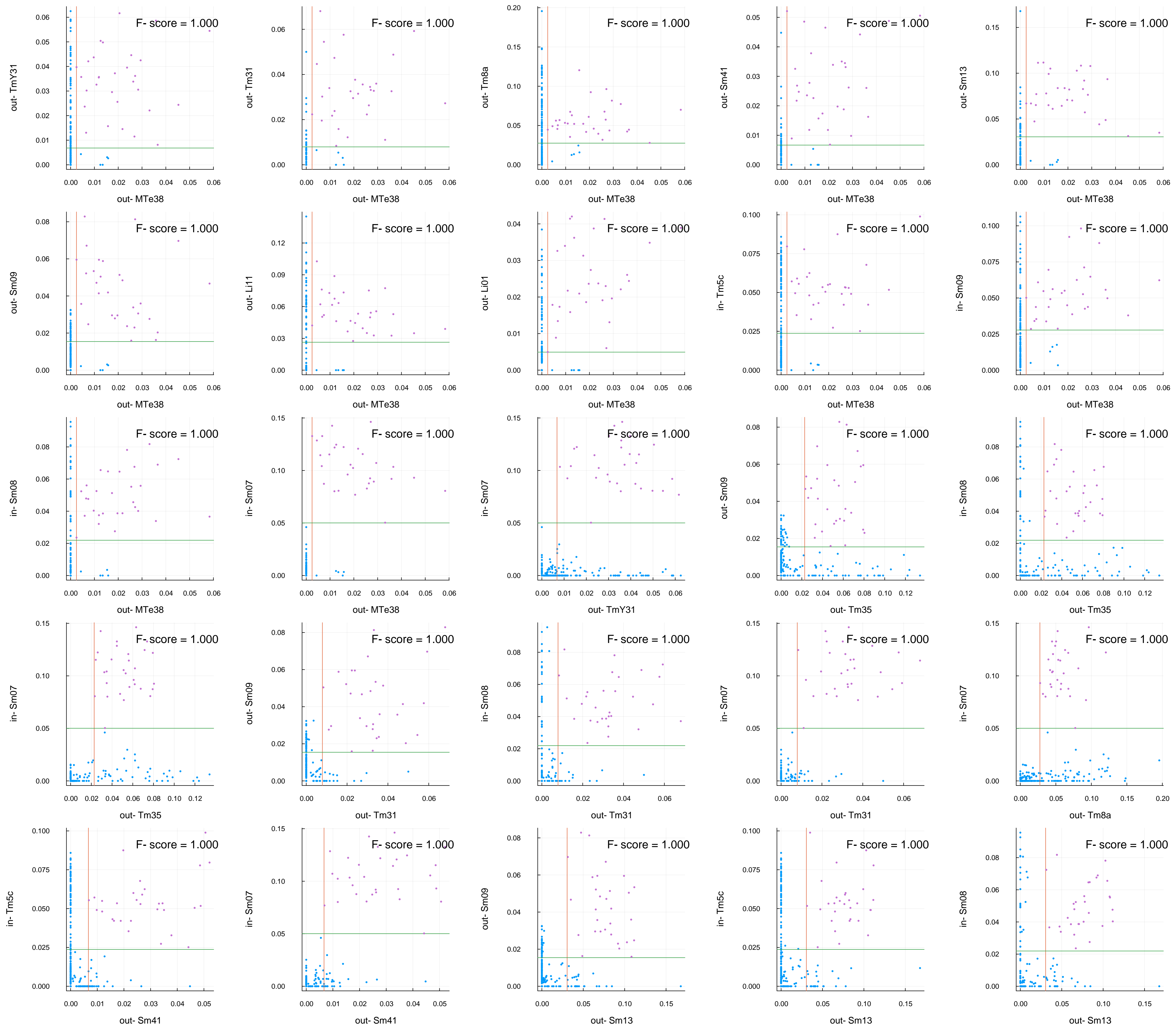

Supplement: Supplementary file 7 — Discriminating 2D projections for neuropil-intrinsic types. For each interneuron type, a pair of features is shown that can be used to discriminate that type from others in the same neuropil. Many although not all discriminations are highly accurate. Both intrinsic and boundary types are included as discriminative features. [file 41586_2024_7981_MOESM7_ESM.zip › DataS3/MLt6.pdf]

MLt7

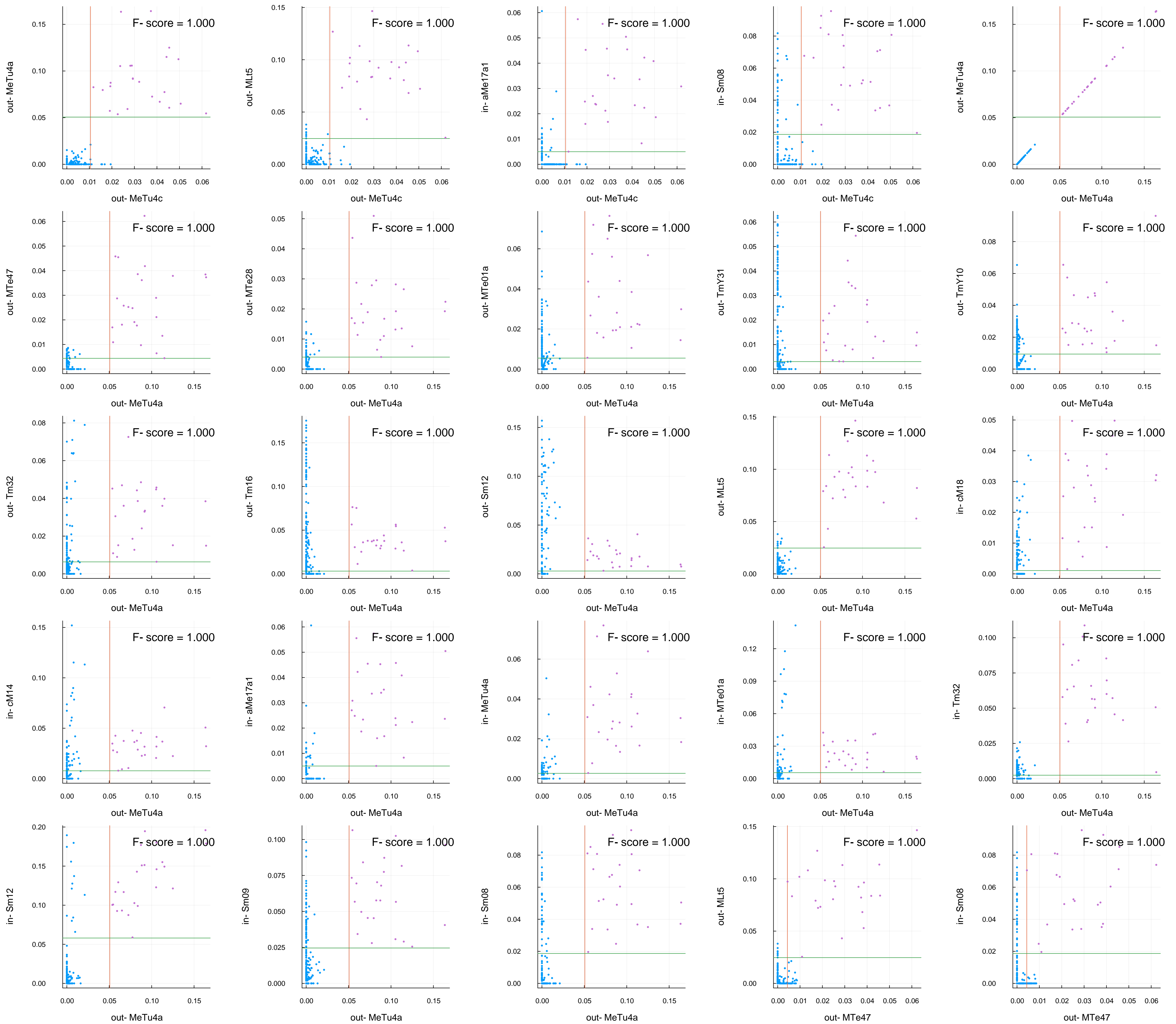

Supplement: Supplementary file 7 — Discriminating 2D projections for neuropil-intrinsic types. For each interneuron type, a pair of features is shown that can be used to discriminate that type from others in the same neuropil. Many although not all discriminations are highly accurate. Both intrinsic and boundary types are included as discriminative features. [file 41586_2024_7981_MOESM7_ESM.zip › DataS3/MLt7.pdf]

MLt8

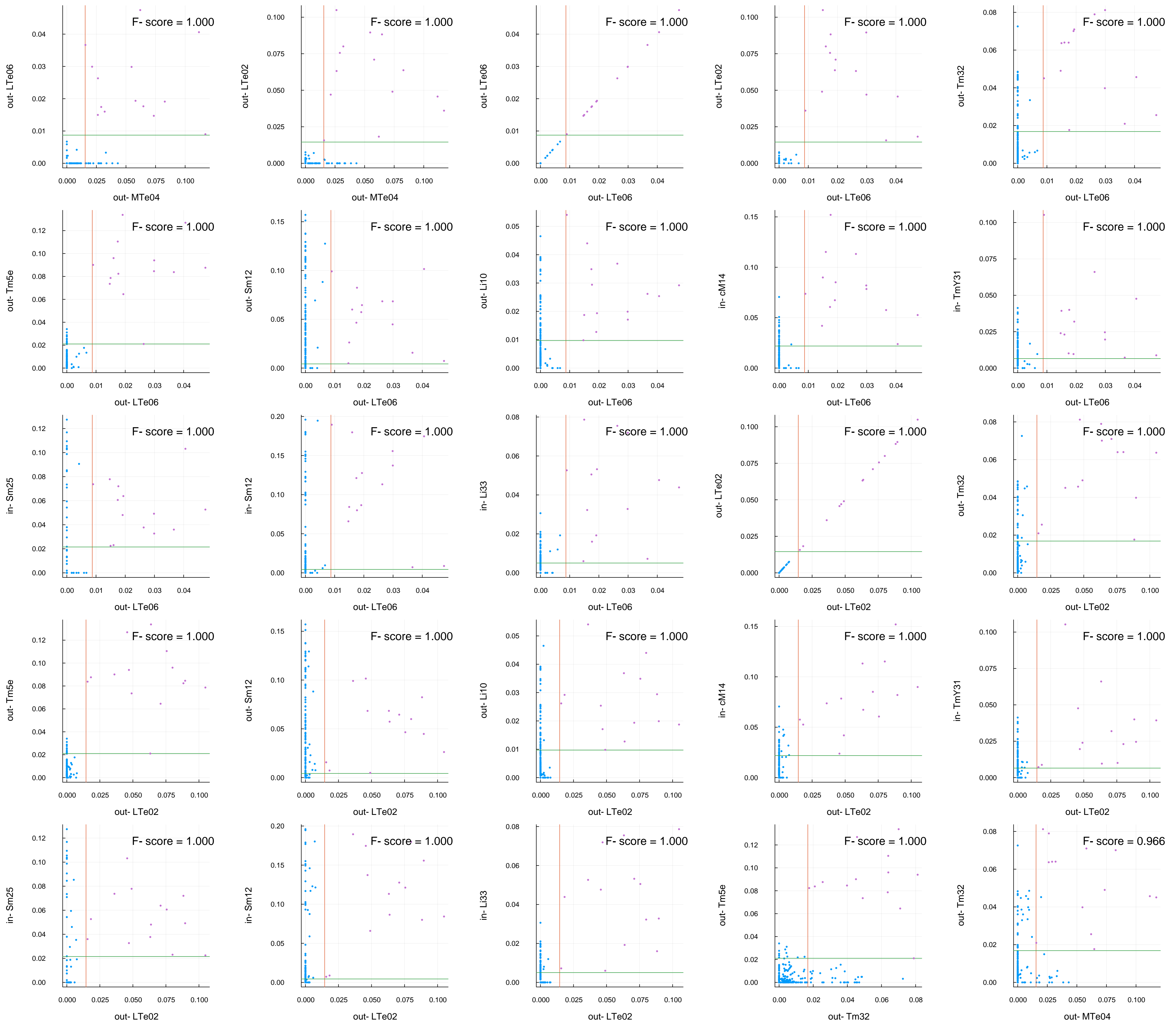

Supplement: Supplementary file 7 — Discriminating 2D projections for neuropil-intrinsic types. For each interneuron type, a pair of features is shown that can be used to discriminate that type from others in the same neuropil. Many although not all discriminations are highly accurate. Both intrinsic and boundary types are included as discriminative features. [file 41586_2024_7981_MOESM7_ESM.zip › DataS3/MLt8.pdf]

Pm01

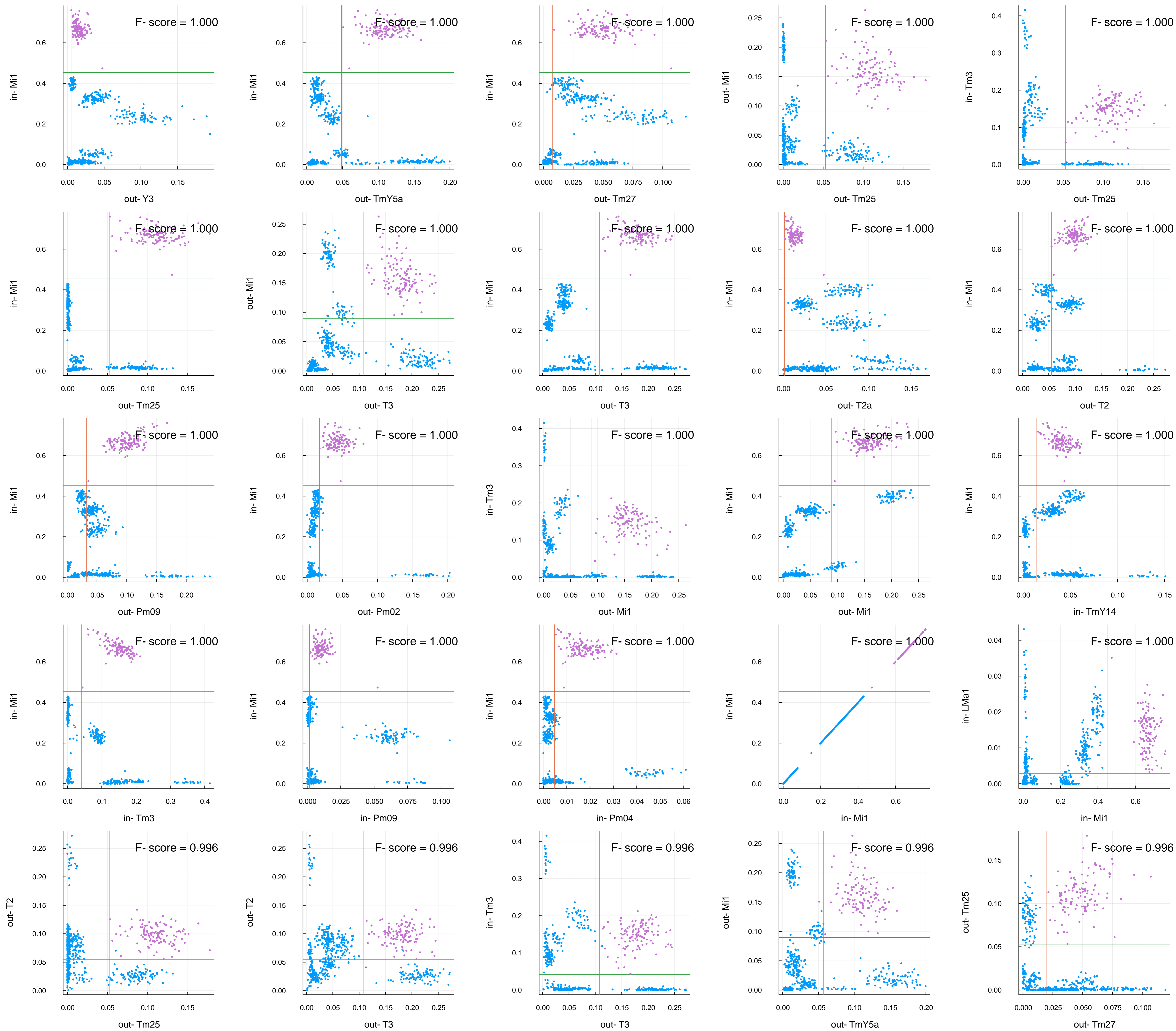

Supplement: Supplementary file 7 — Discriminating 2D projections for neuropil-intrinsic types. For each interneuron type, a pair of features is shown that can be used to discriminate that type from others in the same neuropil. Many although not all discriminations are highly accurate. Both intrinsic and boundary types are included as discriminative features. [file 41586_2024_7981_MOESM7_ESM.zip › DataS3/Pm01.pdf]

Pm02

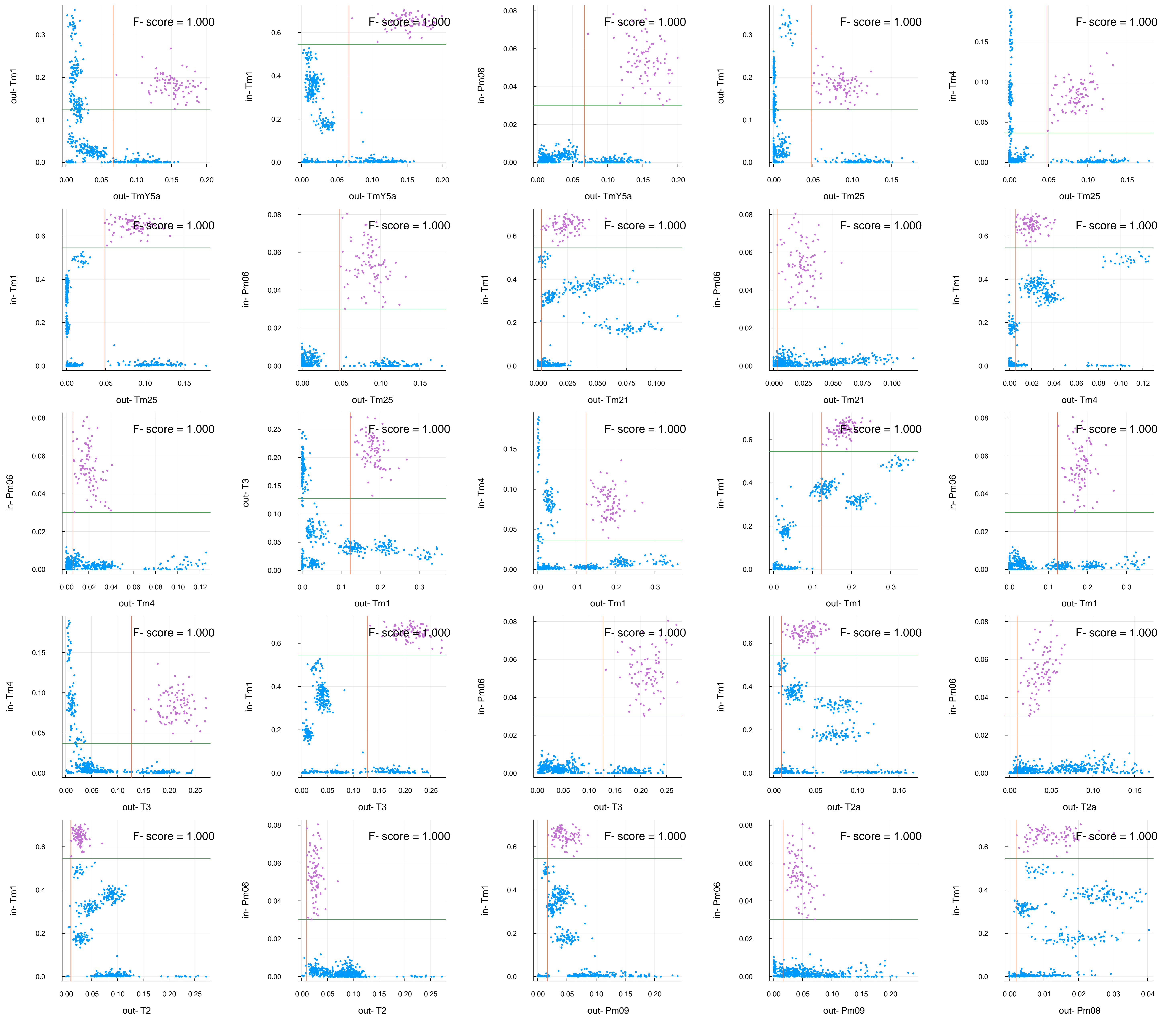

Supplement: Supplementary file 7 — Discriminating 2D projections for neuropil-intrinsic types. For each interneuron type, a pair of features is shown that can be used to discriminate that type from others in the same neuropil. Many although not all discriminations are highly accurate. Both intrinsic and boundary types are included as discriminative features. [file 41586_2024_7981_MOESM7_ESM.zip › DataS3/Pm02.pdf]

Pm03

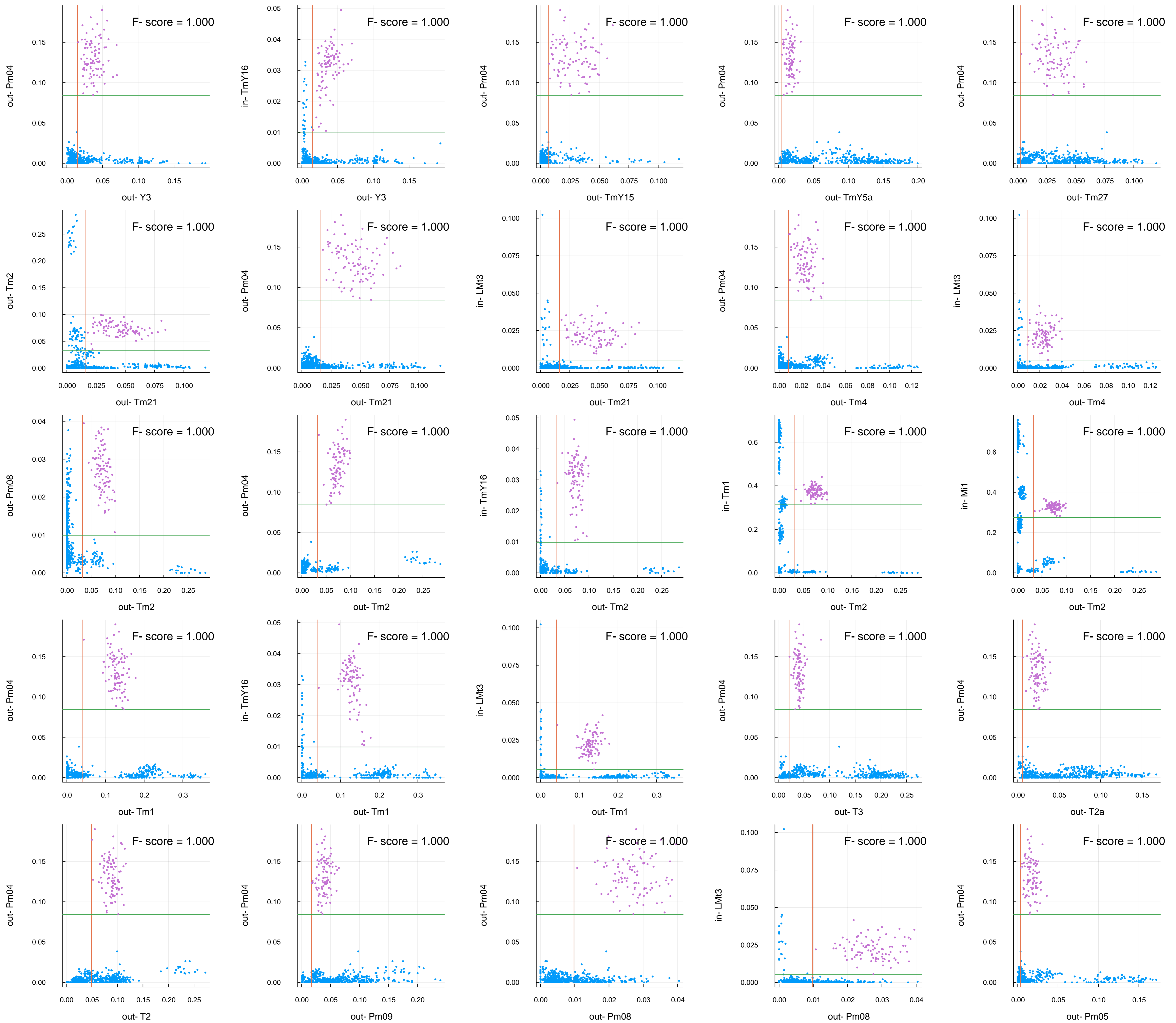

Supplement: Supplementary file 7 — Discriminating 2D projections for neuropil-intrinsic types. For each interneuron type, a pair of features is shown that can be used to discriminate that type from others in the same neuropil. Many although not all discriminations are highly accurate. Both intrinsic and boundary types are included as discriminative features. [file 41586_2024_7981_MOESM7_ESM.zip › DataS3/Pm03.pdf]

Pm04

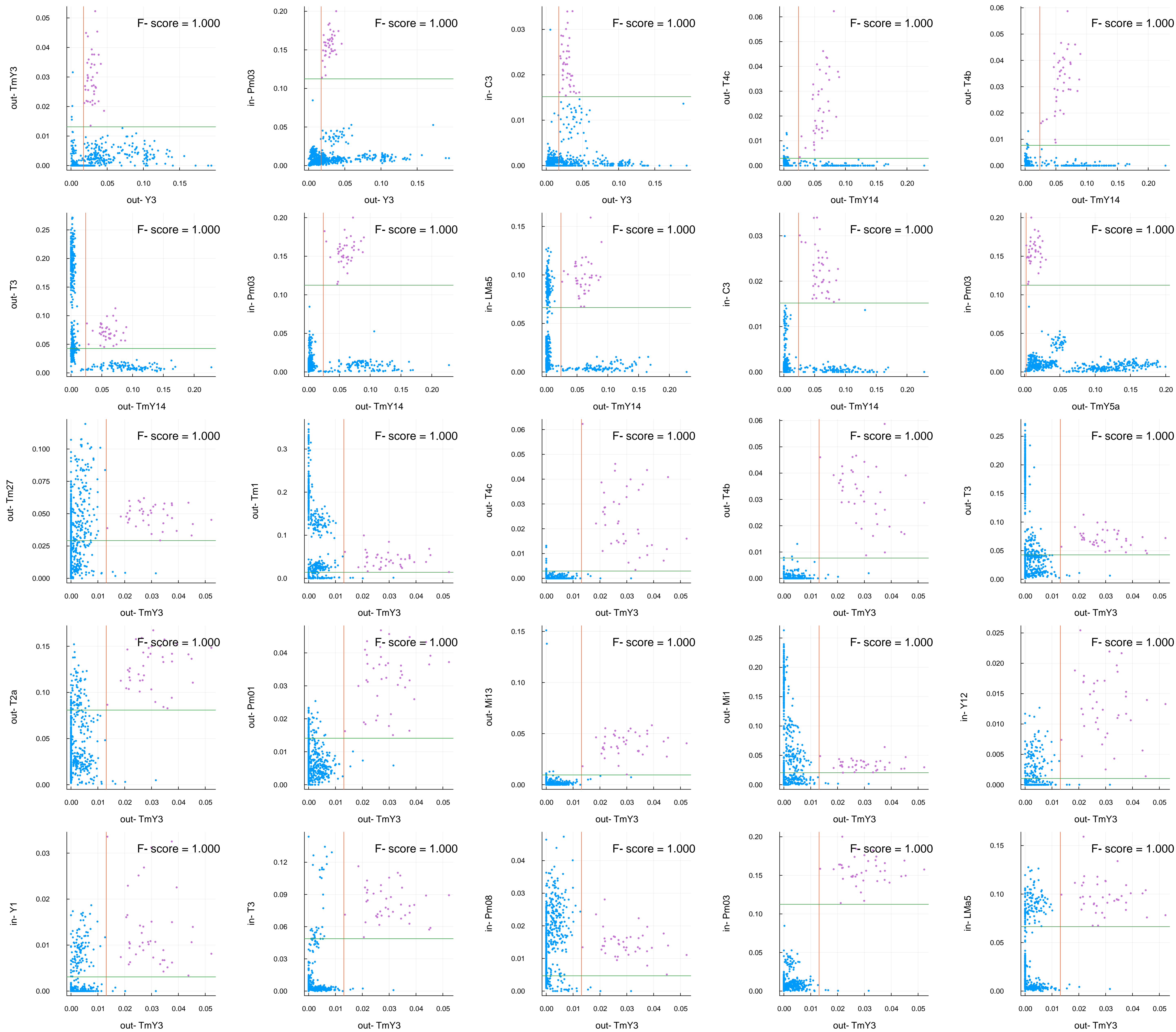

Supplement: Supplementary file 7 — Discriminating 2D projections for neuropil-intrinsic types. For each interneuron type, a pair of features is shown that can be used to discriminate that type from others in the same neuropil. Many although not all discriminations are highly accurate. Both intrinsic and boundary types are included as discriminative features. [file 41586_2024_7981_MOESM7_ESM.zip › DataS3/Pm04.pdf]

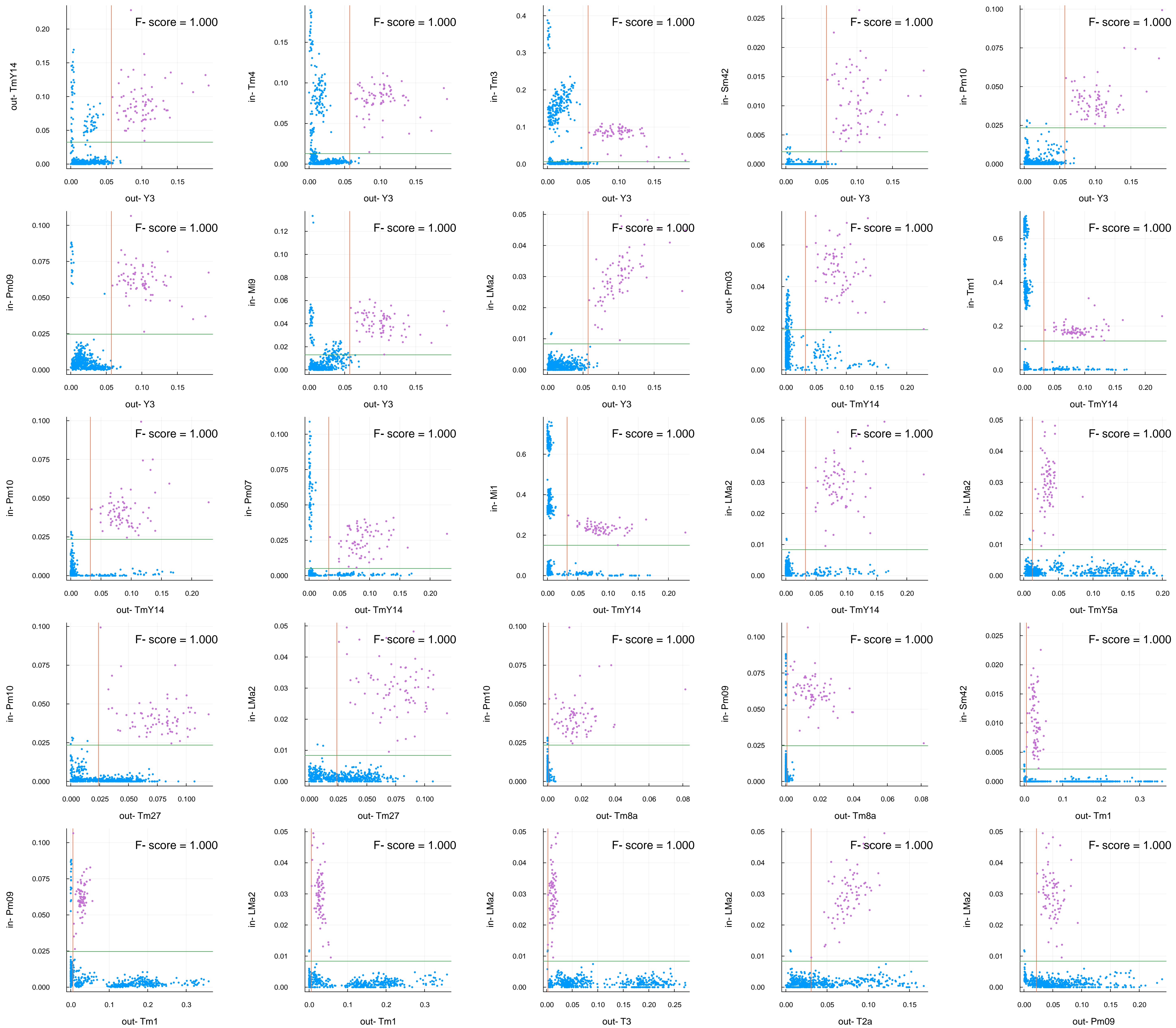

Supplement: Supplementary file 7 — Discriminating 2D projections for neuropil-intrinsic types. For each interneuron type, a pair of features is shown that can be used to discriminate that type from others in the same neuropil. Many although not all discriminations are highly accurate. Both intrinsic and boundary types are included as discriminative features. [file 41586_2024_7981_MOESM7_ESM.zip › DataS3/Pm05.pdf]

Pm06

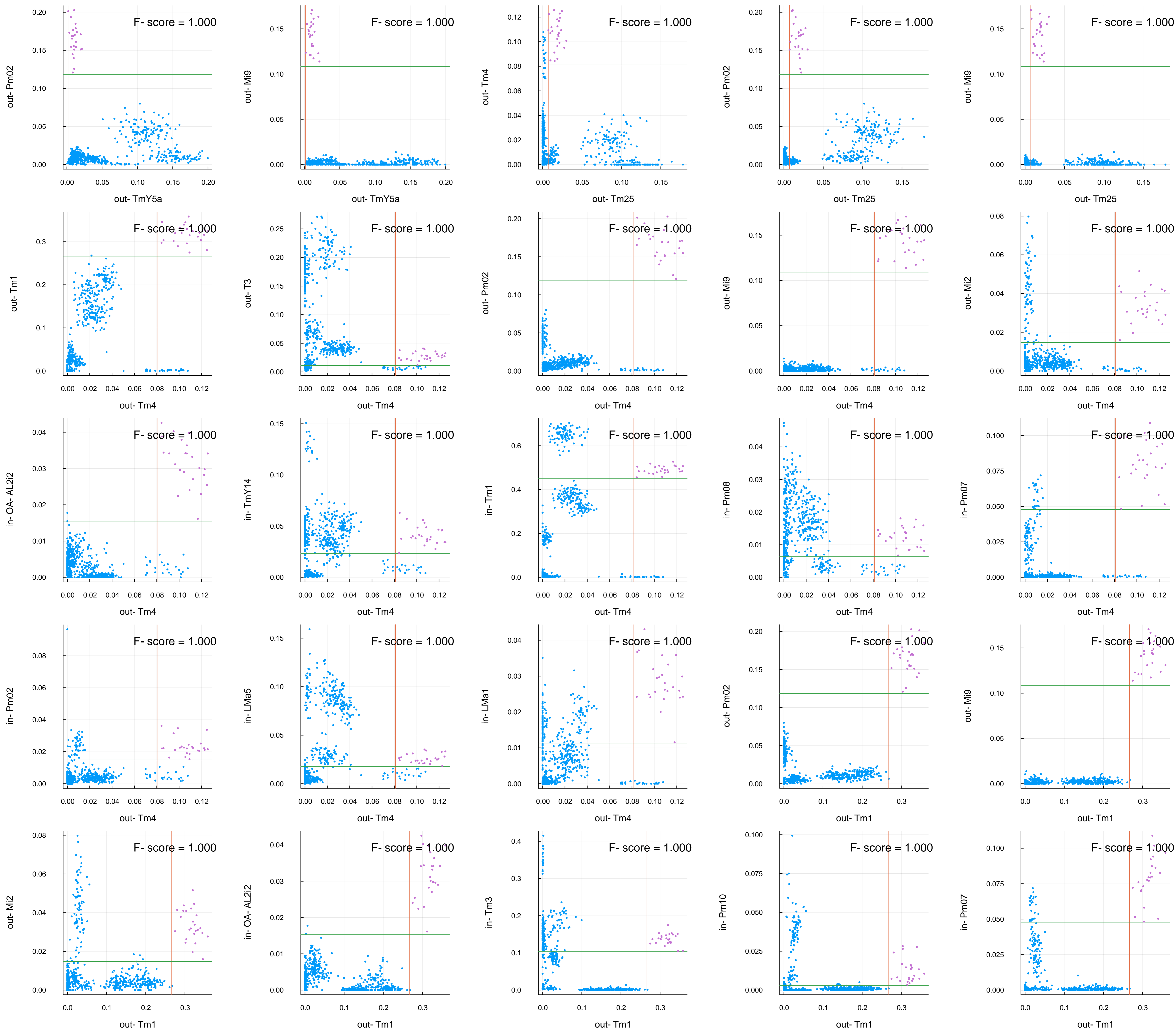

Supplement: Supplementary file 7 — Discriminating 2D projections for neuropil-intrinsic types. For each interneuron type, a pair of features is shown that can be used to discriminate that type from others in the same neuropil. Many although not all discriminations are highly accurate. Both intrinsic and boundary types are included as discriminative features. [file 41586_2024_7981_MOESM7_ESM.zip › DataS3/Pm06.pdf]

Pm07

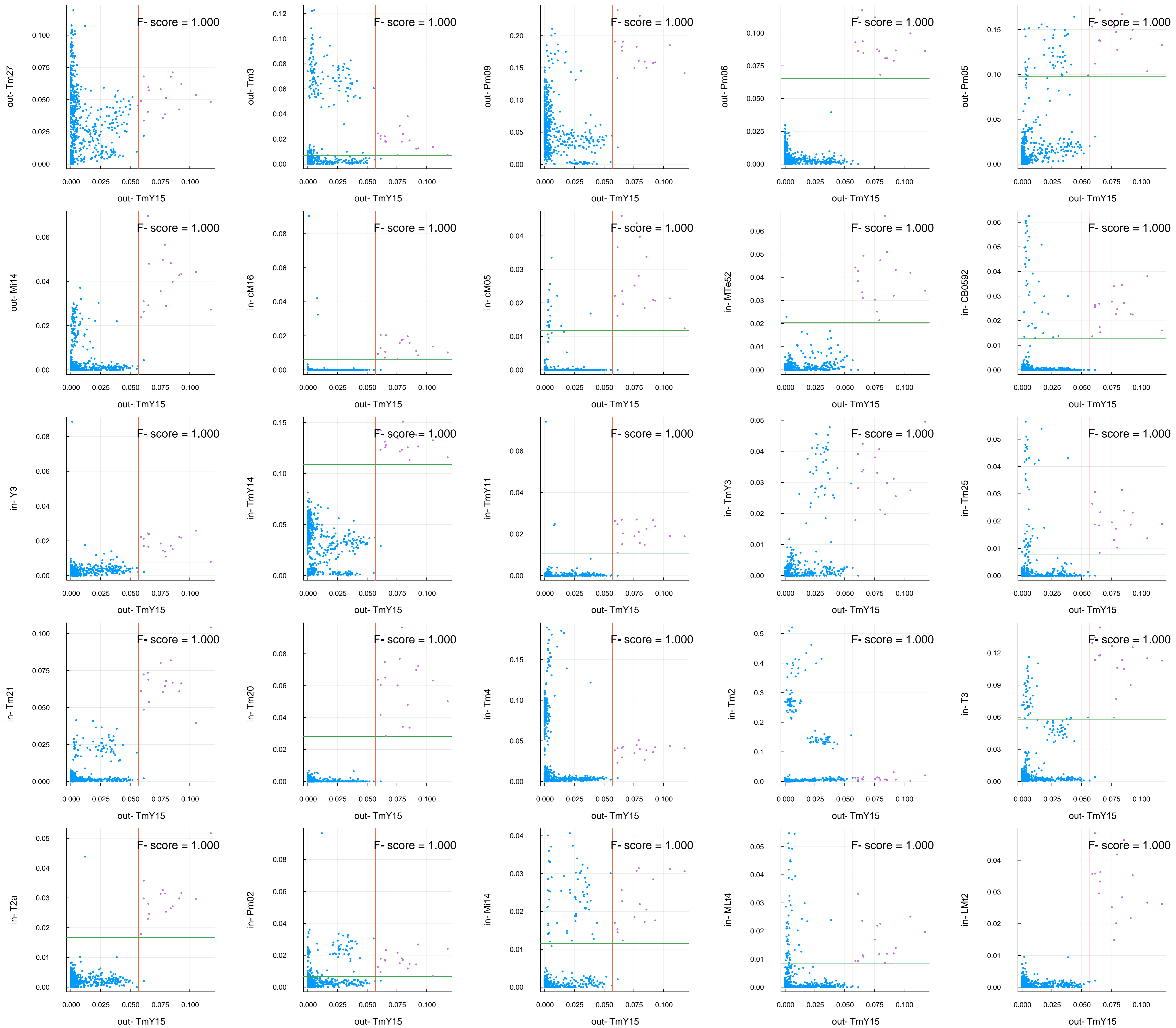

Supplement: Supplementary file 7 — Discriminating 2D projections for neuropil-intrinsic types. For each interneuron type, a pair of features is shown that can be used to discriminate that type from others in the same neuropil. Many although not all discriminations are highly accurate. Both intrinsic and boundary types are included as discriminative features. [file 41586_2024_7981_MOESM7_ESM.zip › DataS3/Pm07.pdf]

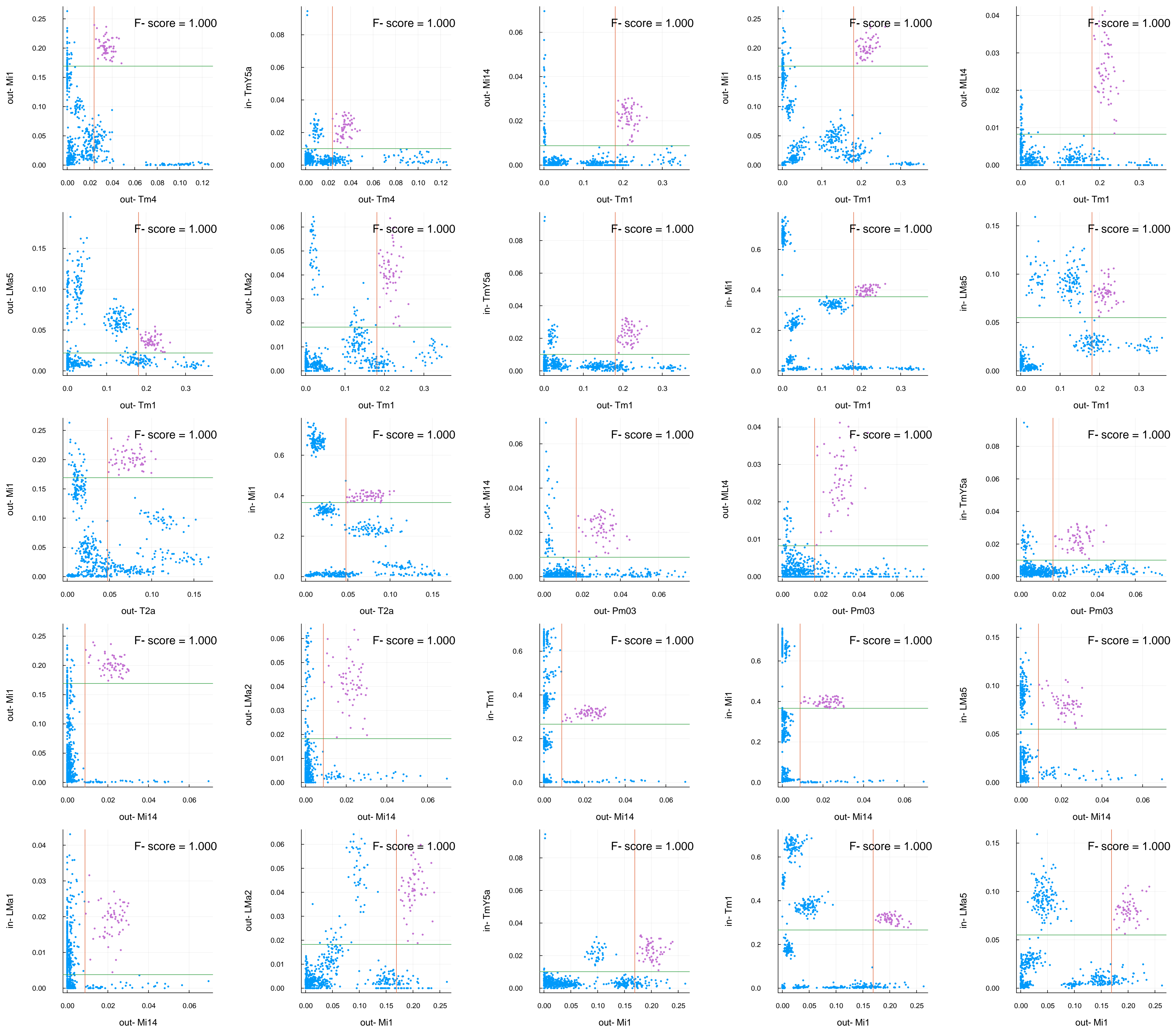

Supplement: Supplementary file 7 — Discriminating 2D projections for neuropil-intrinsic types. For each interneuron type, a pair of features is shown that can be used to discriminate that type from others in the same neuropil. Many although not all discriminations are highly accurate. Both intrinsic and boundary types are included as discriminative features. [file 41586_2024_7981_MOESM7_ESM.zip › DataS3/Pm08.pdf]

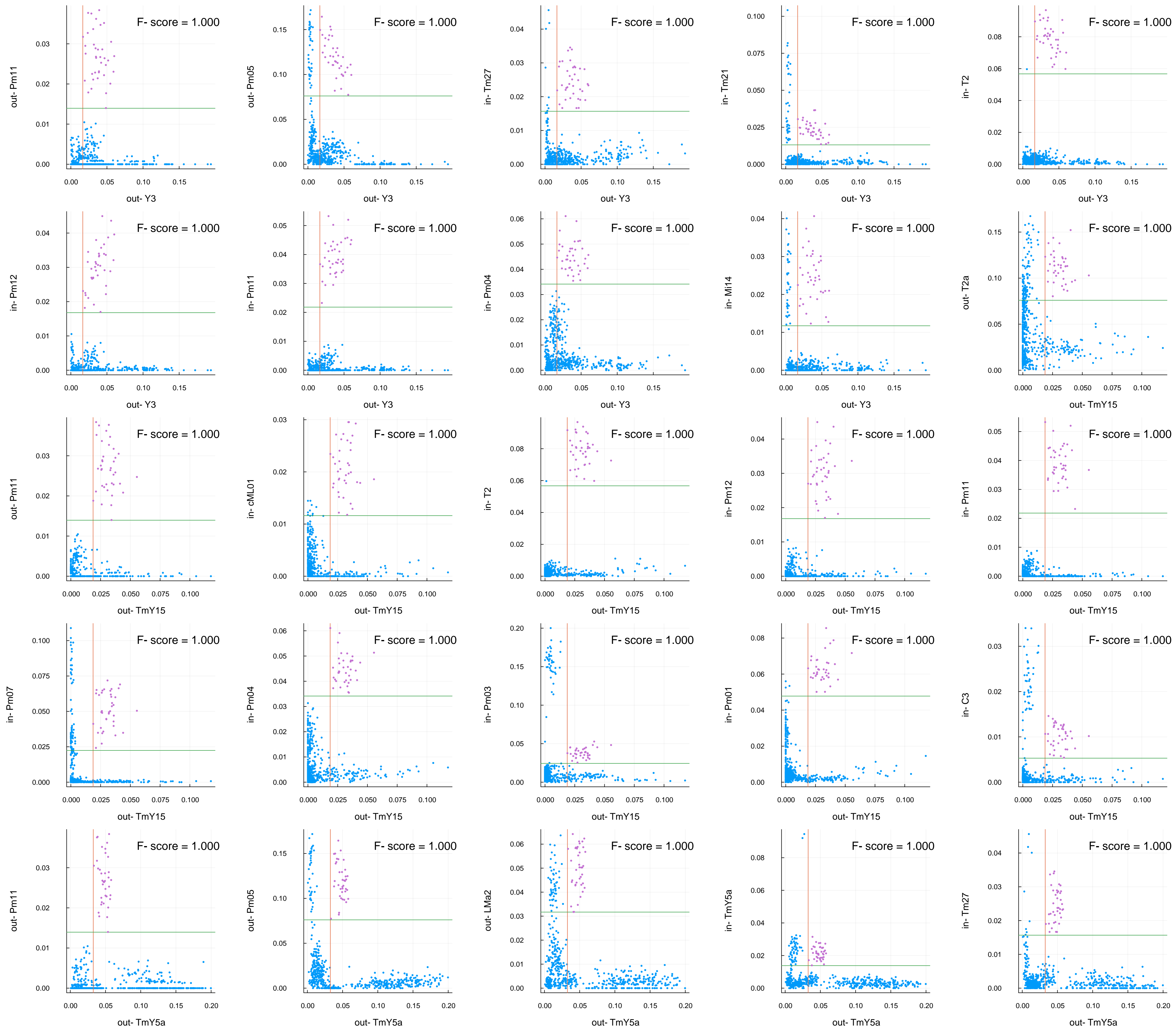

Supplement: Supplementary file 7 — Discriminating 2D projections for neuropil-intrinsic types. For each interneuron type, a pair of features is shown that can be used to discriminate that type from others in the same neuropil. Many although not all discriminations are highly accurate. Both intrinsic and boundary types are included as discriminative features. [file 41586_2024_7981_MOESM7_ESM.zip › DataS3/Pm09.pdf]

Pm10

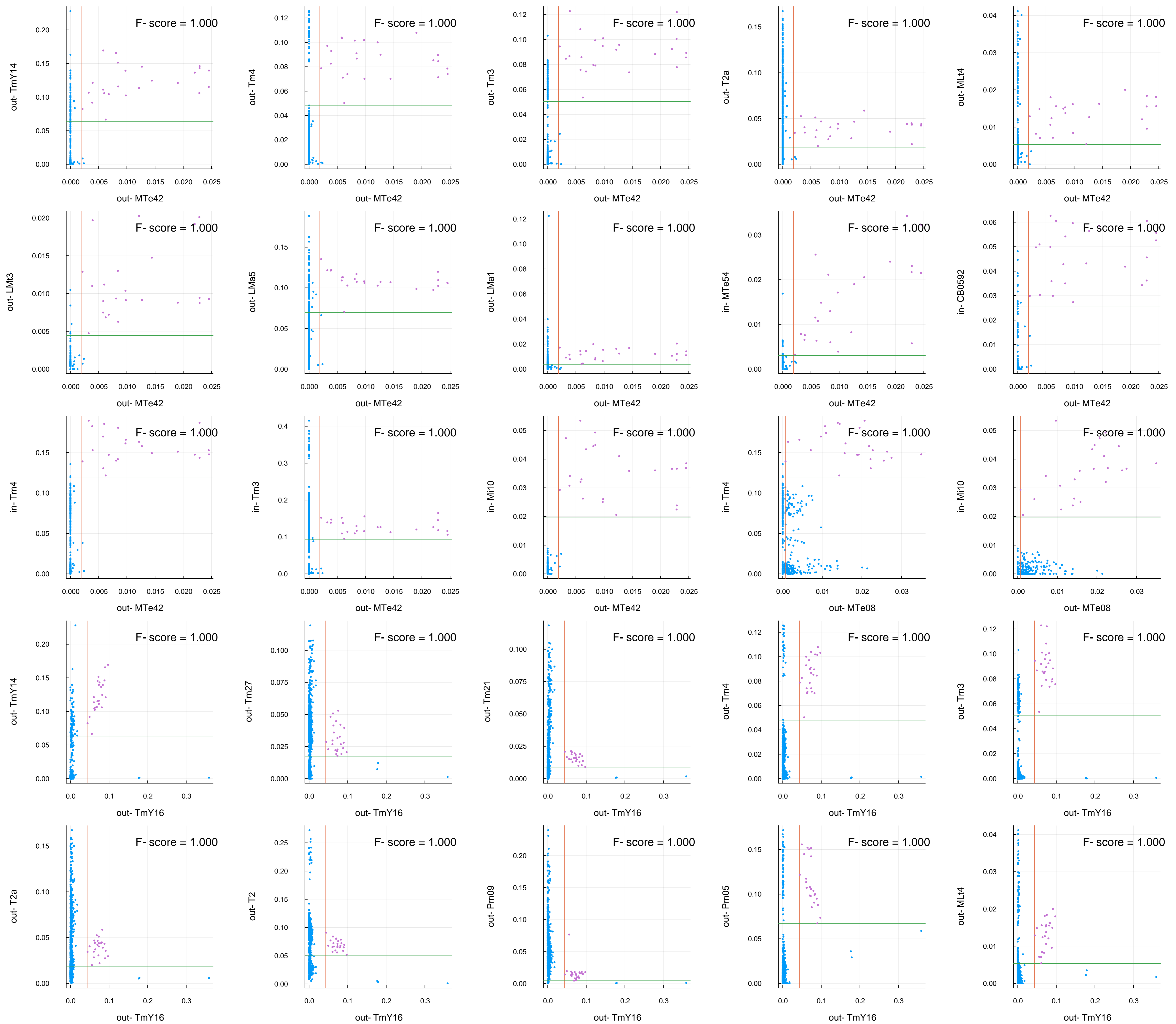

Supplement: Supplementary file 7 — Discriminating 2D projections for neuropil-intrinsic types. For each interneuron type, a pair of features is shown that can be used to discriminate that type from others in the same neuropil. Many although not all discriminations are highly accurate. Both intrinsic and boundary types are included as discriminative features. [file 41586_2024_7981_MOESM7_ESM.zip › DataS3/Pm10.pdf]

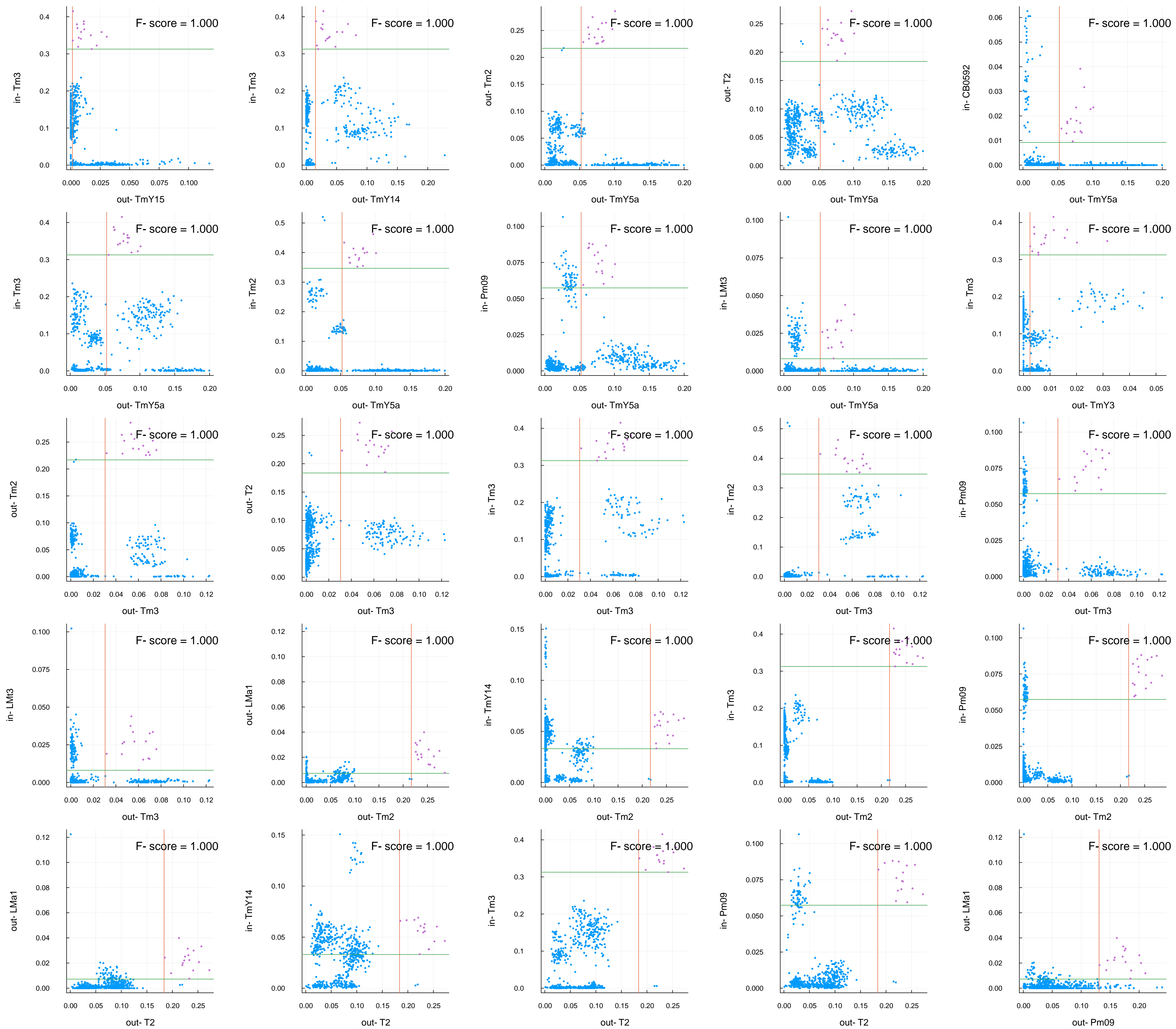

Supplement: Supplementary file 7 — Discriminating 2D projections for neuropil-intrinsic types. For each interneuron type, a pair of features is shown that can be used to discriminate that type from others in the same neuropil. Many although not all discriminations are highly accurate. Both intrinsic and boundary types are included as discriminative features. [file 41586_2024_7981_MOESM7_ESM.zip › DataS3/Pm11.pdf]

Pm12

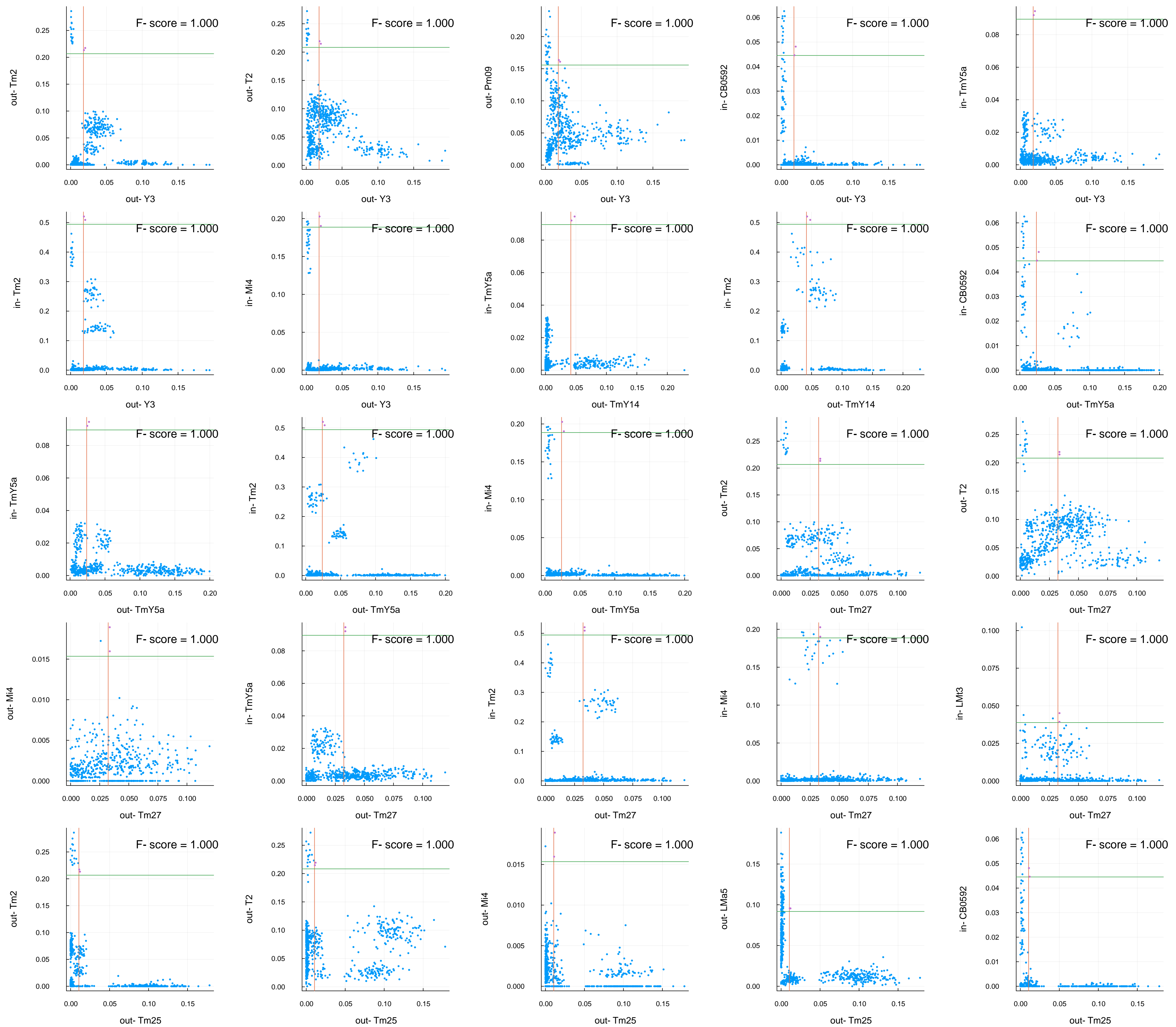

Supplement: Supplementary file 7 — Discriminating 2D projections for neuropil-intrinsic types. For each interneuron type, a pair of features is shown that can be used to discriminate that type from others in the same neuropil. Many although not all discriminations are highly accurate. Both intrinsic and boundary types are included as discriminative features. [file 41586_2024_7981_MOESM7_ESM.zip › DataS3/Pm12.pdf]

Pm13

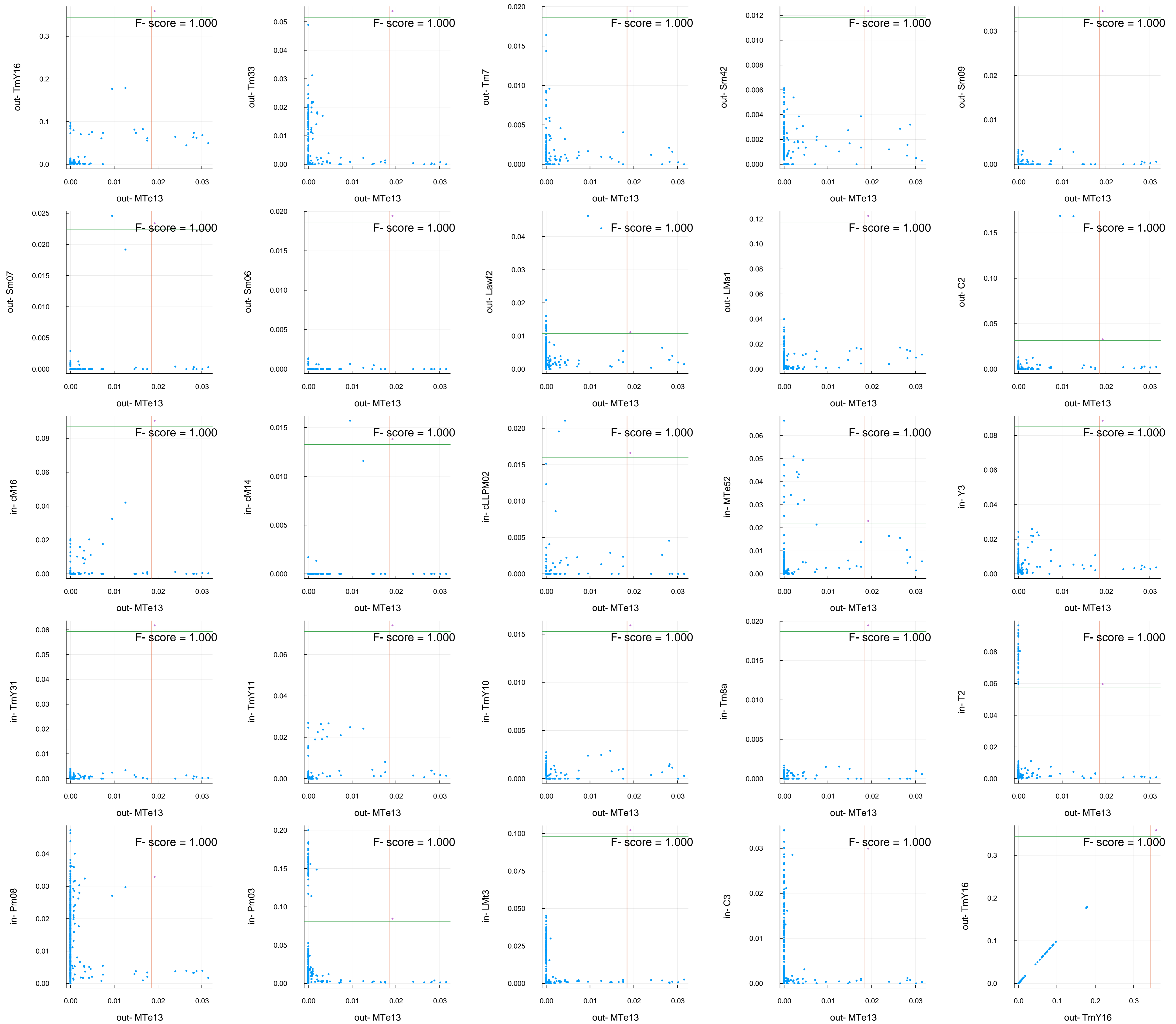

Supplement: Supplementary file 7 — Discriminating 2D projections for neuropil-intrinsic types. For each interneuron type, a pair of features is shown that can be used to discriminate that type from others in the same neuropil. Many although not all discriminations are highly accurate. Both intrinsic and boundary types are included as discriminative features. [file 41586_2024_7981_MOESM7_ESM.zip › DataS3/Pm13.pdf]

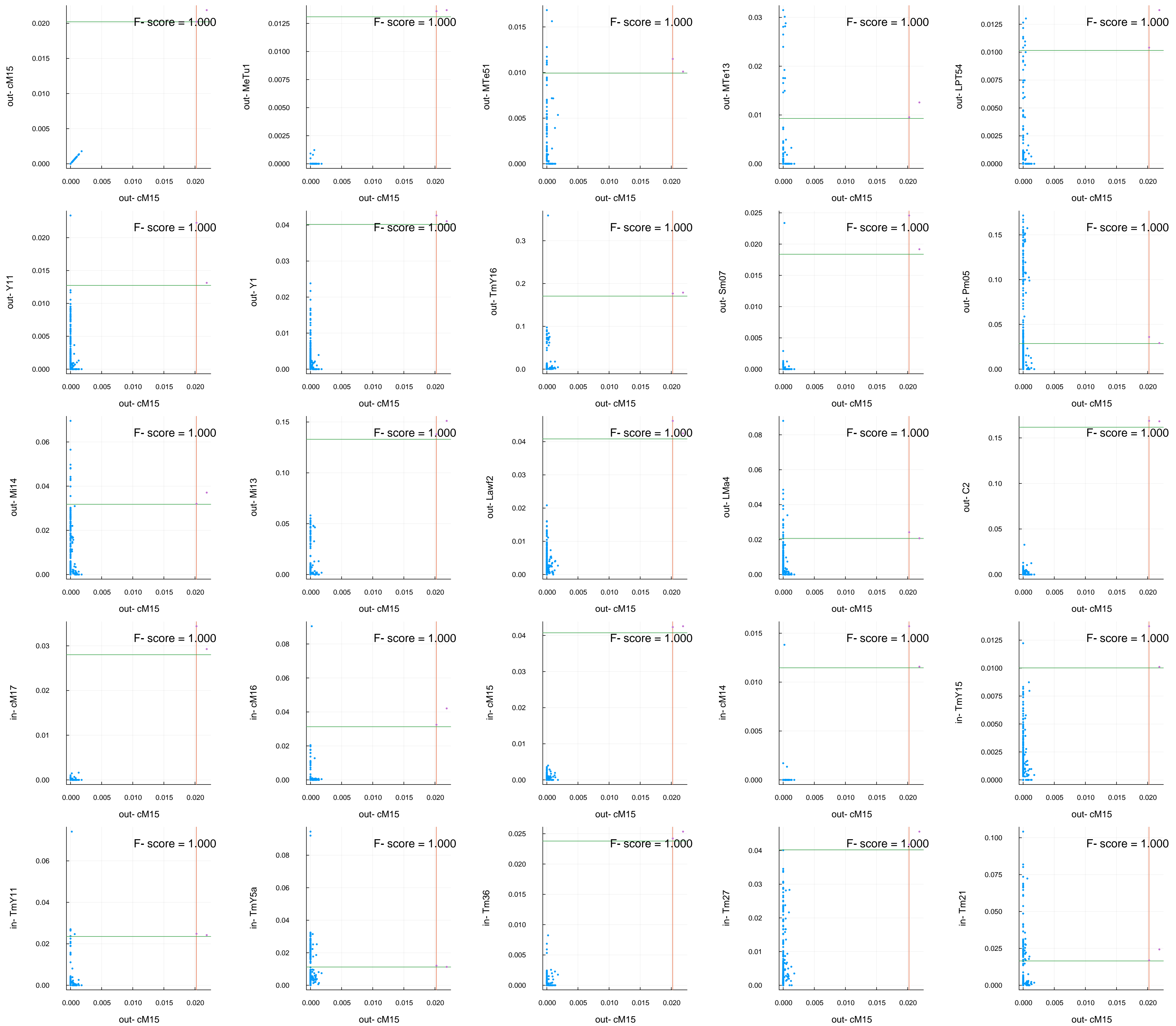

Supplement: Supplementary file 7 — Discriminating 2D projections for neuropil-intrinsic types. For each interneuron type, a pair of features is shown that can be used to discriminate that type from others in the same neuropil. Many although not all discriminations are highly accurate. Both intrinsic and boundary types are included as discriminative features. [file 41586_2024_7981_MOESM7_ESM.zip › DataS3/Pm14.pdf]

Sm01

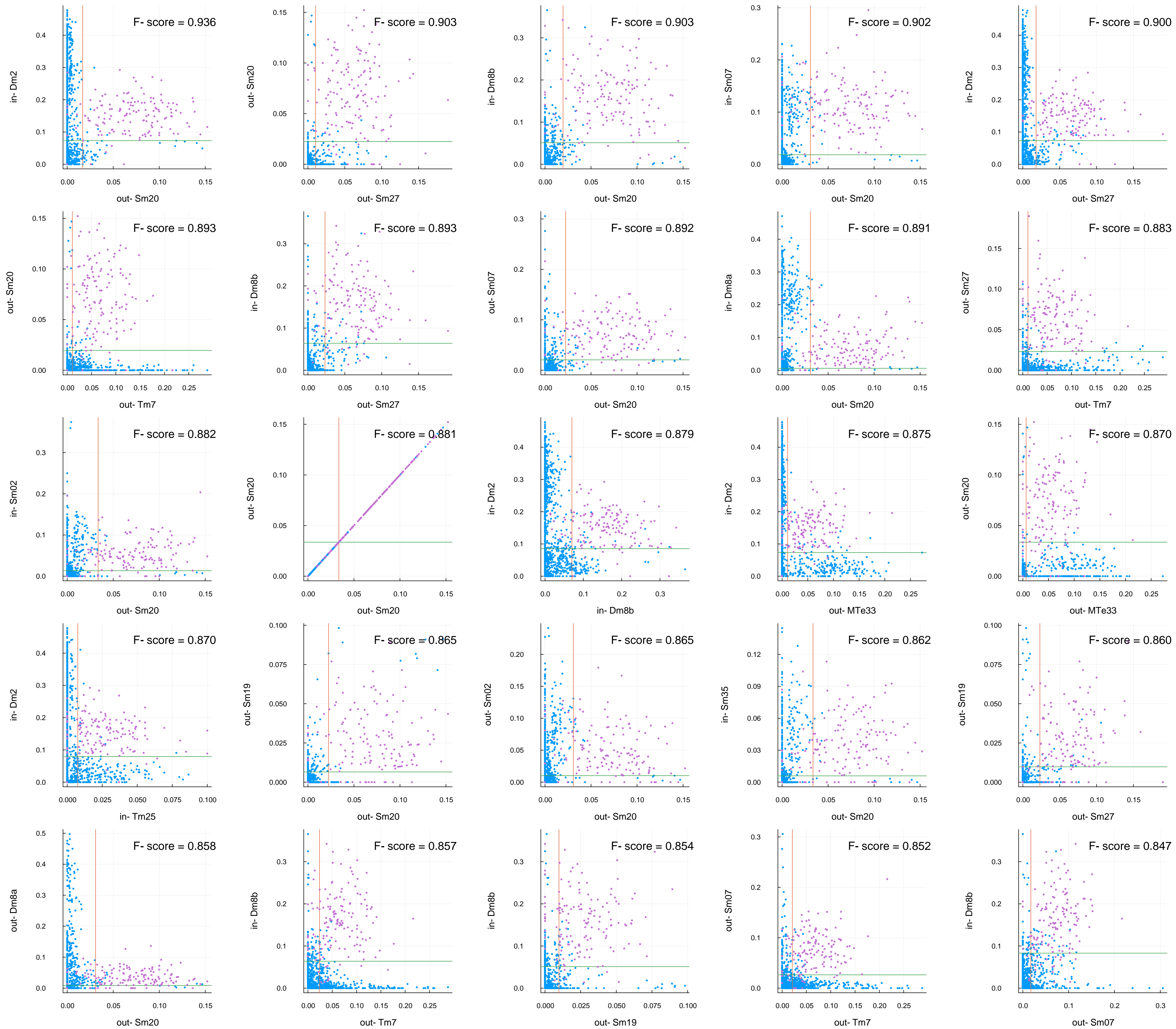

Supplement: Supplementary file 7 — Discriminating 2D projections for neuropil-intrinsic types. For each interneuron type, a pair of features is shown that can be used to discriminate that type from others in the same neuropil. Many although not all discriminations are highly accurate. Both intrinsic and boundary types are included as discriminative features. [file 41586_2024_7981_MOESM7_ESM.zip › DataS3/Sm01.pdf]

Sm02

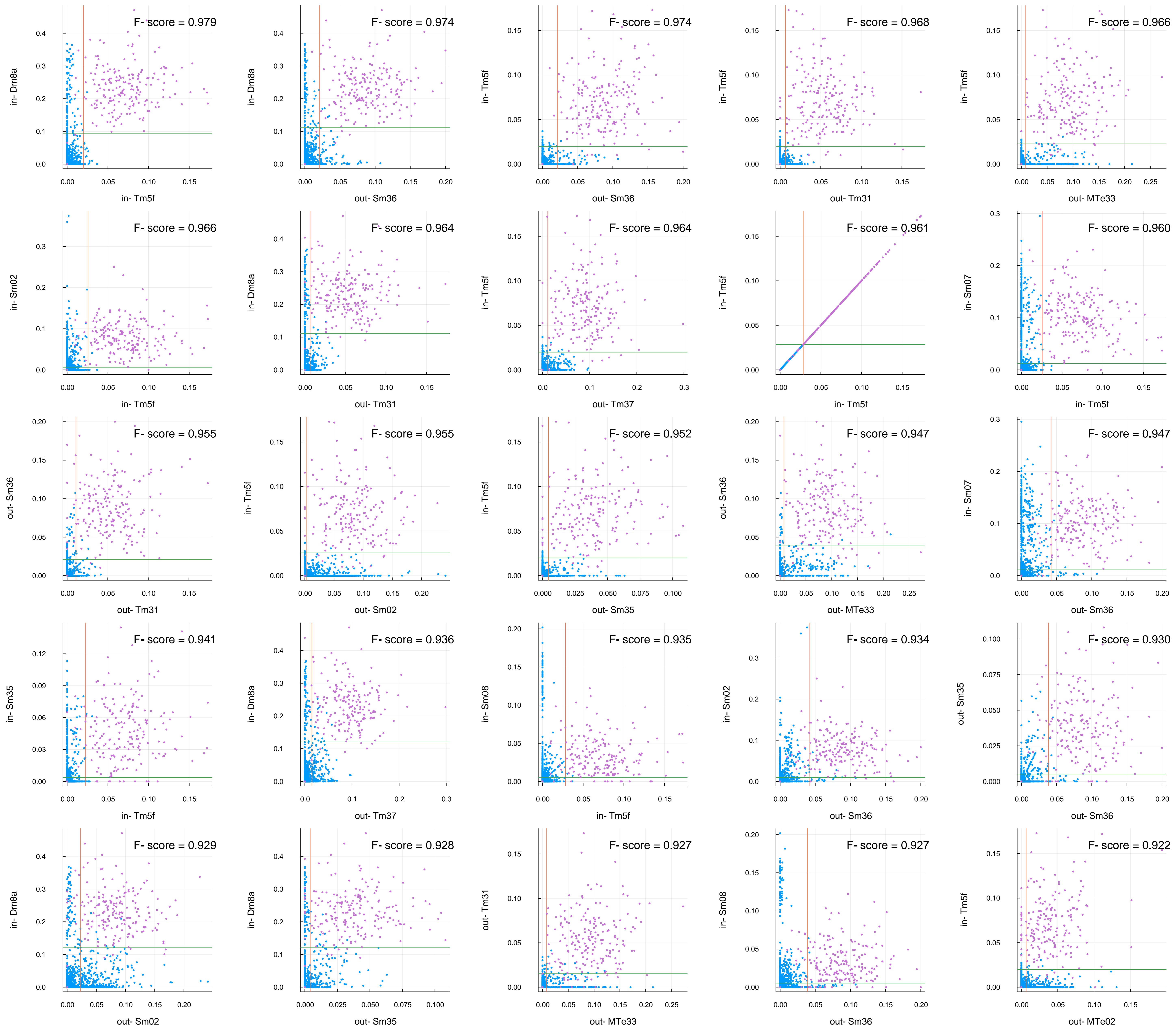

Supplement: Supplementary file 7 — Discriminating 2D projections for neuropil-intrinsic types. For each interneuron type, a pair of features is shown that can be used to discriminate that type from others in the same neuropil. Many although not all discriminations are highly accurate. Both intrinsic and boundary types are included as discriminative features. [file 41586_2024_7981_MOESM7_ESM.zip › DataS3/Sm02.pdf]

Sm03

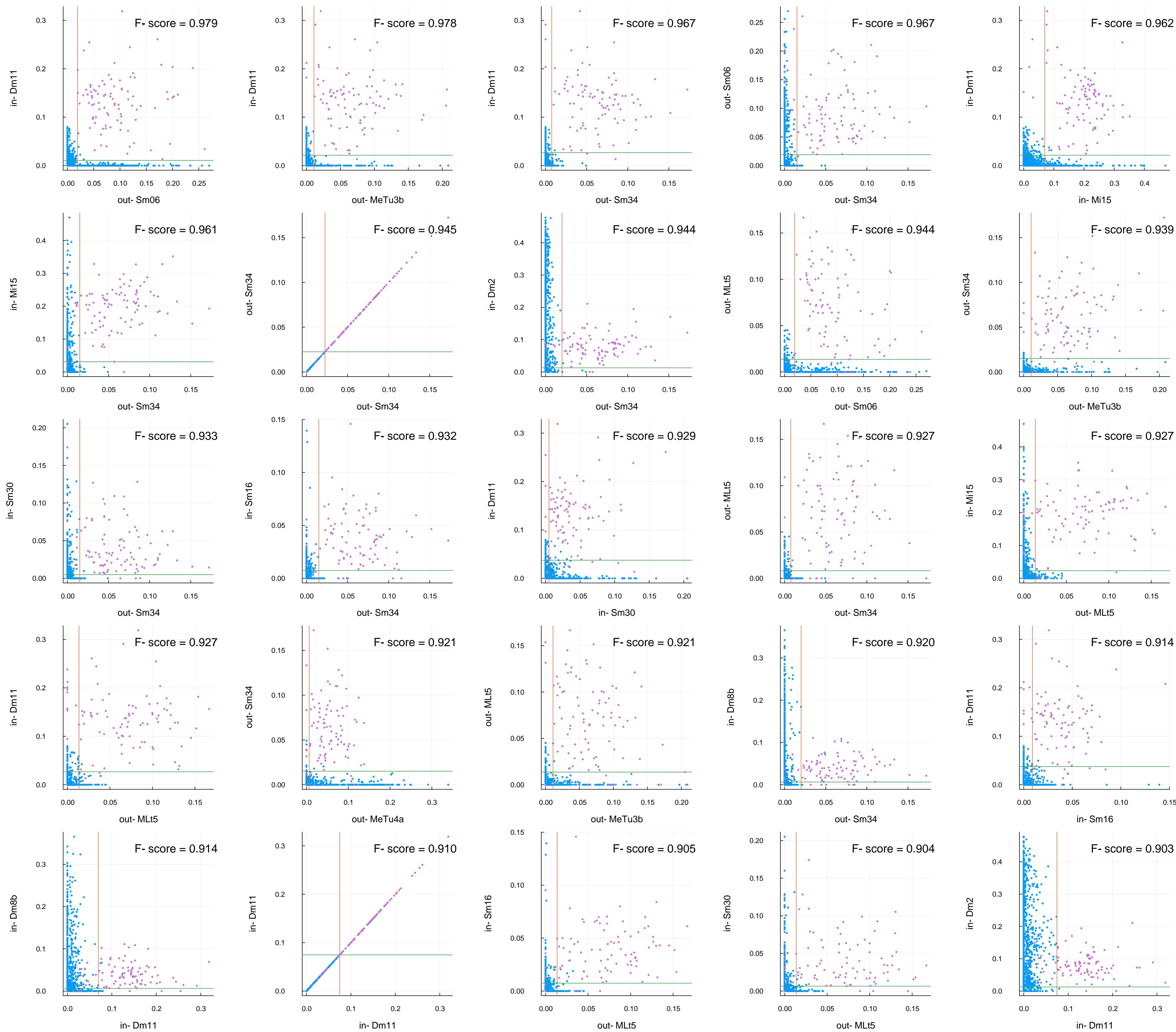

Supplement: Supplementary file 7 — Discriminating 2D projections for neuropil-intrinsic types. For each interneuron type, a pair of features is shown that can be used to discriminate that type from others in the same neuropil. Many although not all discriminations are highly accurate. Both intrinsic and boundary types are included as discriminative features. [file 41586_2024_7981_MOESM7_ESM.zip › DataS3/Sm03.pdf]

Sm04

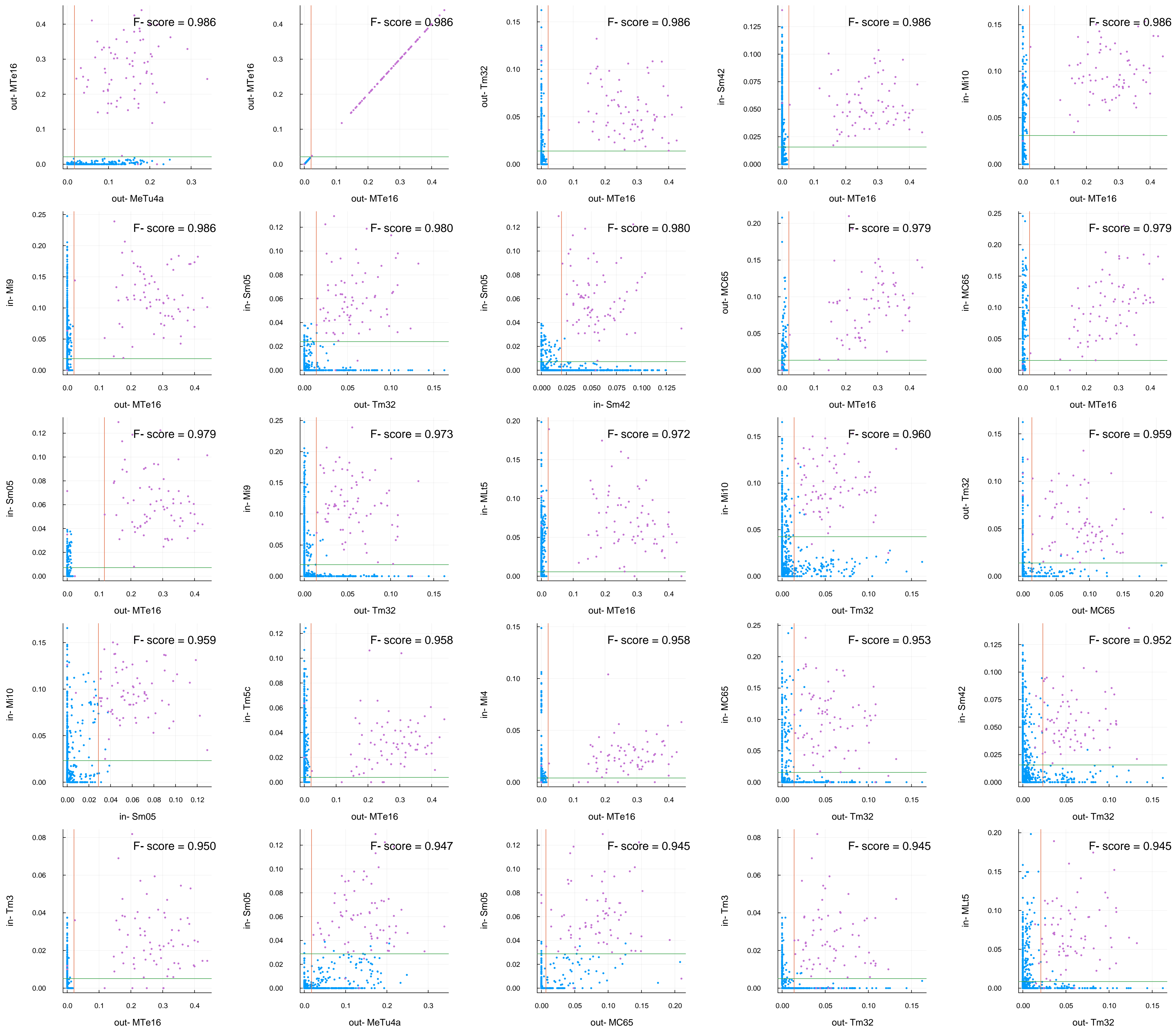

Supplement: Supplementary file 7 — Discriminating 2D projections for neuropil-intrinsic types. For each interneuron type, a pair of features is shown that can be used to discriminate that type from others in the same neuropil. Many although not all discriminations are highly accurate. Both intrinsic and boundary types are included as discriminative features. [file 41586_2024_7981_MOESM7_ESM.zip › DataS3/Sm04.pdf]

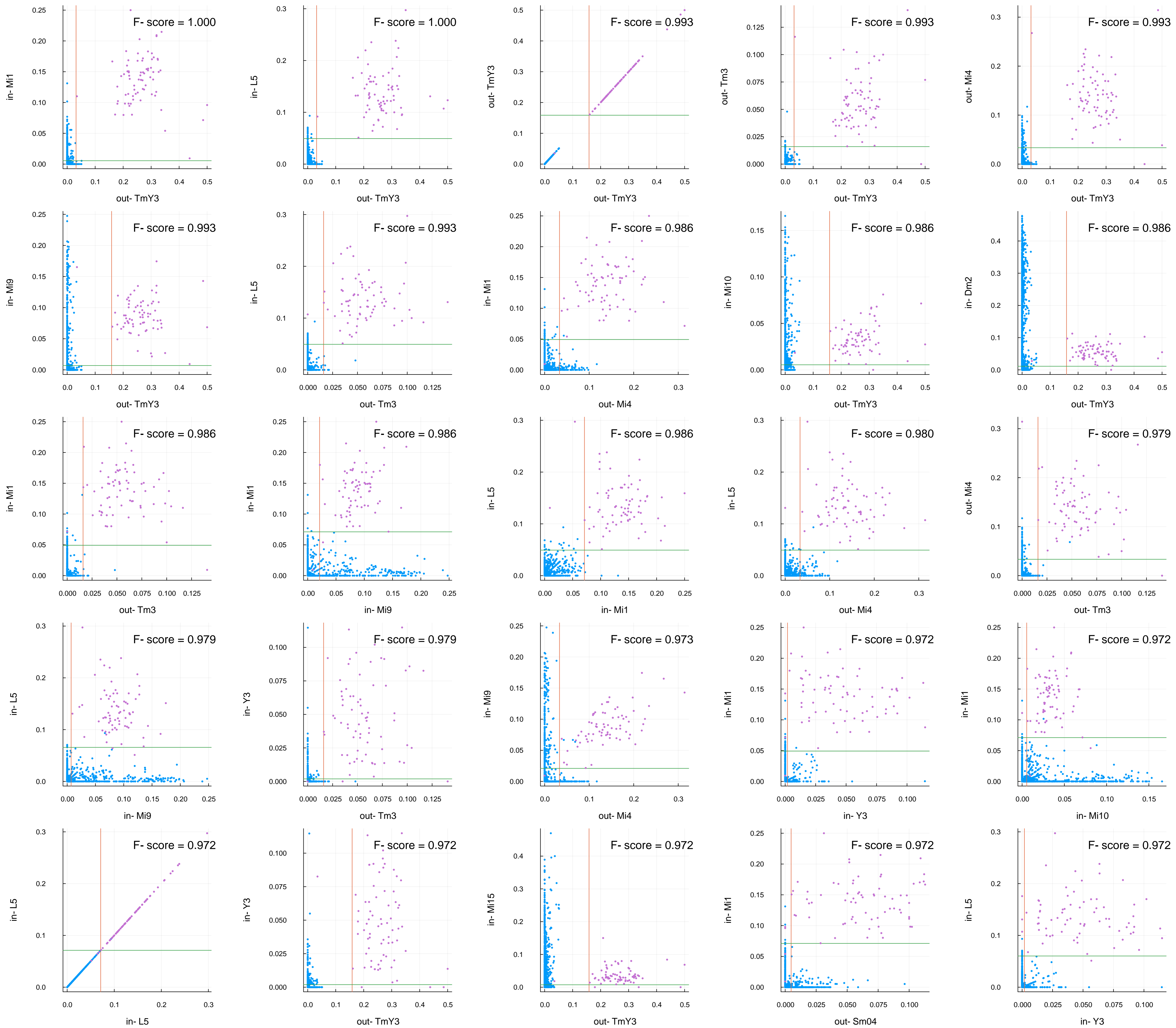

Supplement: Supplementary file 7 — Discriminating 2D projections for neuropil-intrinsic types. For each interneuron type, a pair of features is shown that can be used to discriminate that type from others in the same neuropil. Many although not all discriminations are highly accurate. Both intrinsic and boundary types are included as discriminative features. [file 41586_2024_7981_MOESM7_ESM.zip › DataS3/Sm05.pdf]

Sm06

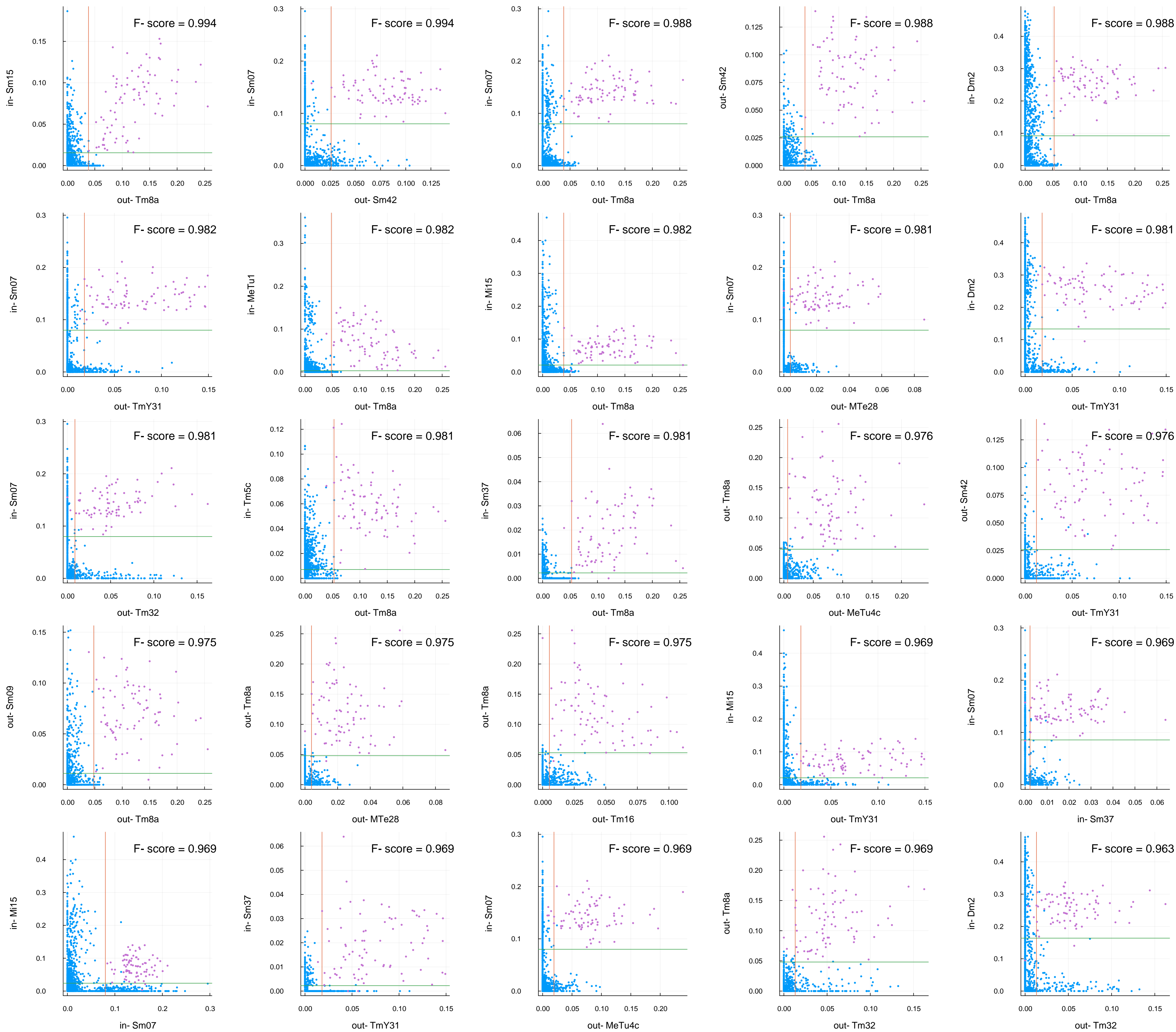

Supplement: Supplementary file 7 — Discriminating 2D projections for neuropil-intrinsic types. For each interneuron type, a pair of features is shown that can be used to discriminate that type from others in the same neuropil. Many although not all discriminations are highly accurate. Both intrinsic and boundary types are included as discriminative features. [file 41586_2024_7981_MOESM7_ESM.zip › DataS3/Sm06.pdf]

Sm07

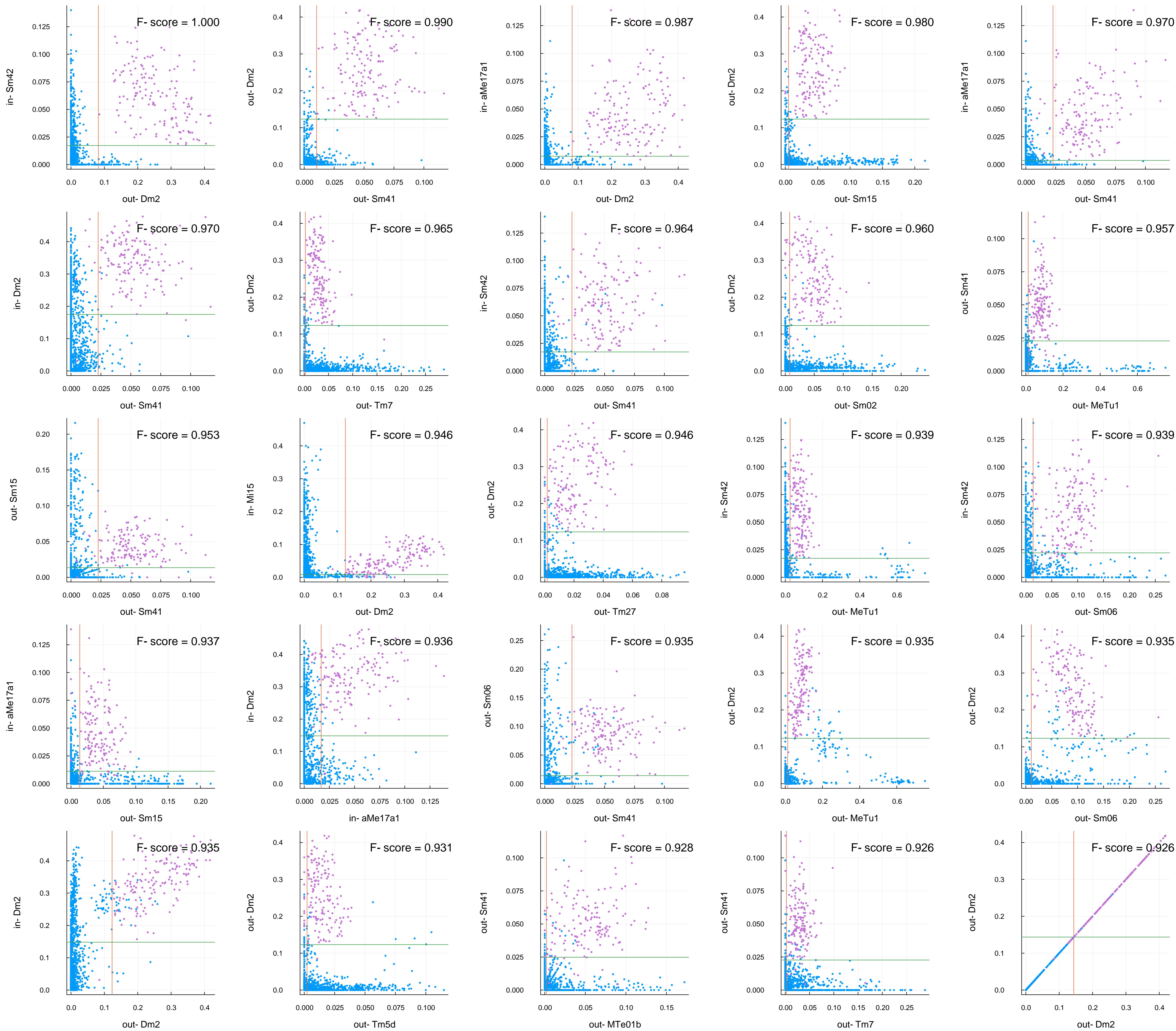

Supplement: Supplementary file 7 — Discriminating 2D projections for neuropil-intrinsic types. For each interneuron type, a pair of features is shown that can be used to discriminate that type from others in the same neuropil. Many although not all discriminations are highly accurate. Both intrinsic and boundary types are included as discriminative features. [file 41586_2024_7981_MOESM7_ESM.zip › DataS3/Sm07.pdf]

Sm08

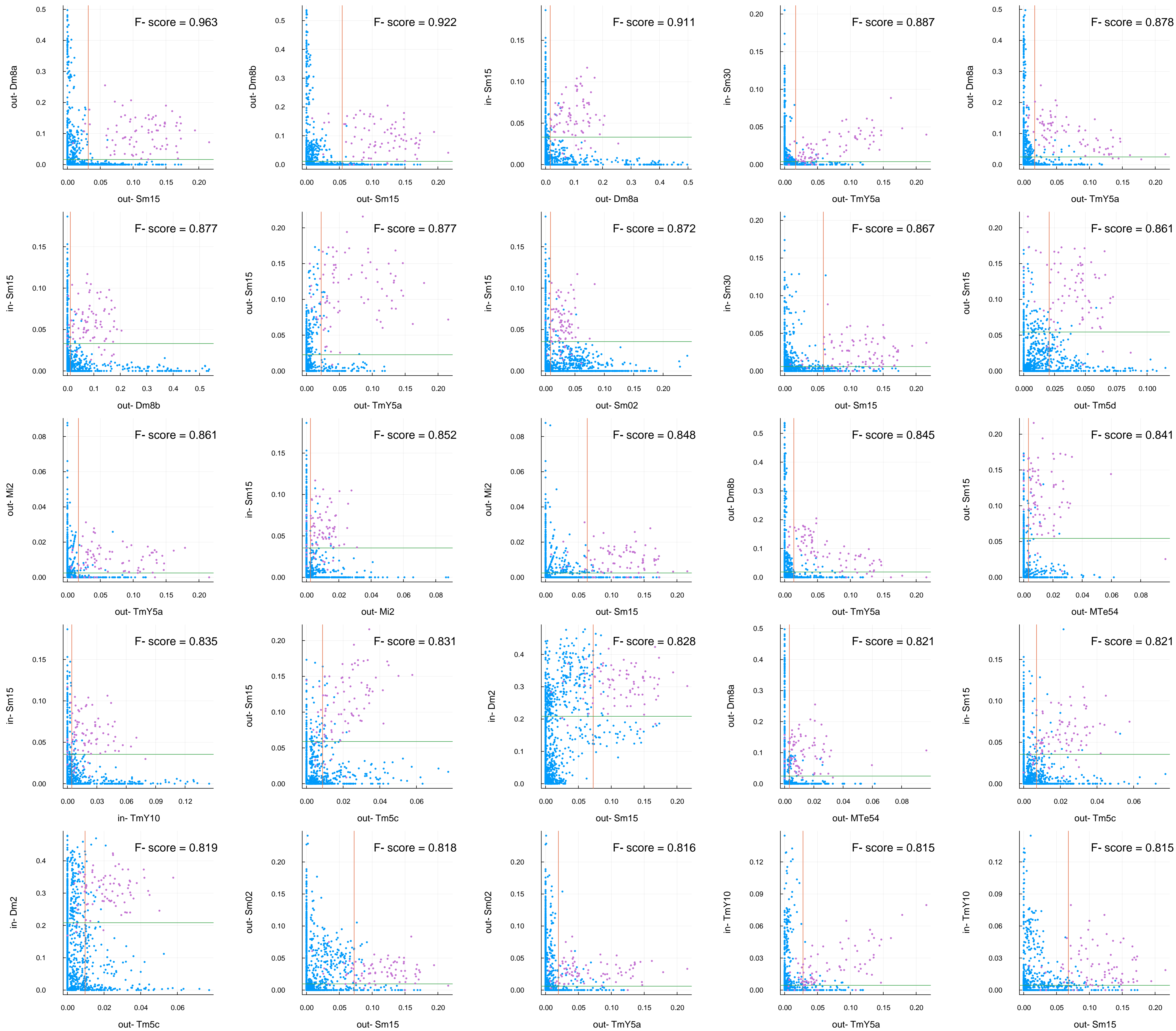

Supplement: Supplementary file 7 — Discriminating 2D projections for neuropil-intrinsic types. For each interneuron type, a pair of features is shown that can be used to discriminate that type from others in the same neuropil. Many although not all discriminations are highly accurate. Both intrinsic and boundary types are included as discriminative features. [file 41586_2024_7981_MOESM7_ESM.zip › DataS3/Sm08.pdf]

Sm09

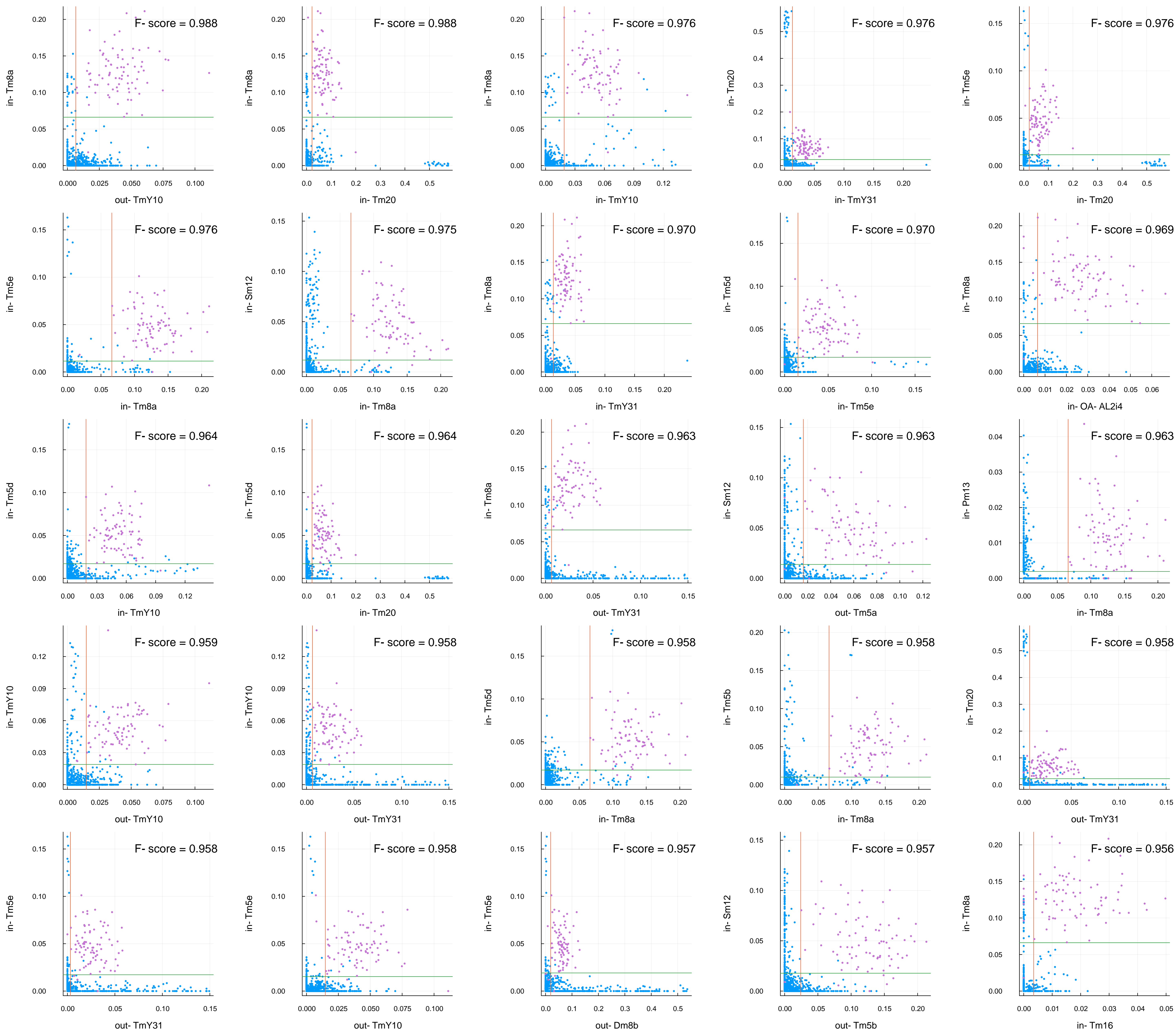

Supplement: Supplementary file 7 — Discriminating 2D projections for neuropil-intrinsic types. For each interneuron type, a pair of features is shown that can be used to discriminate that type from others in the same neuropil. Many although not all discriminations are highly accurate. Both intrinsic and boundary types are included as discriminative features. [file 41586_2024_7981_MOESM7_ESM.zip › DataS3/Sm09.pdf]

Sm10

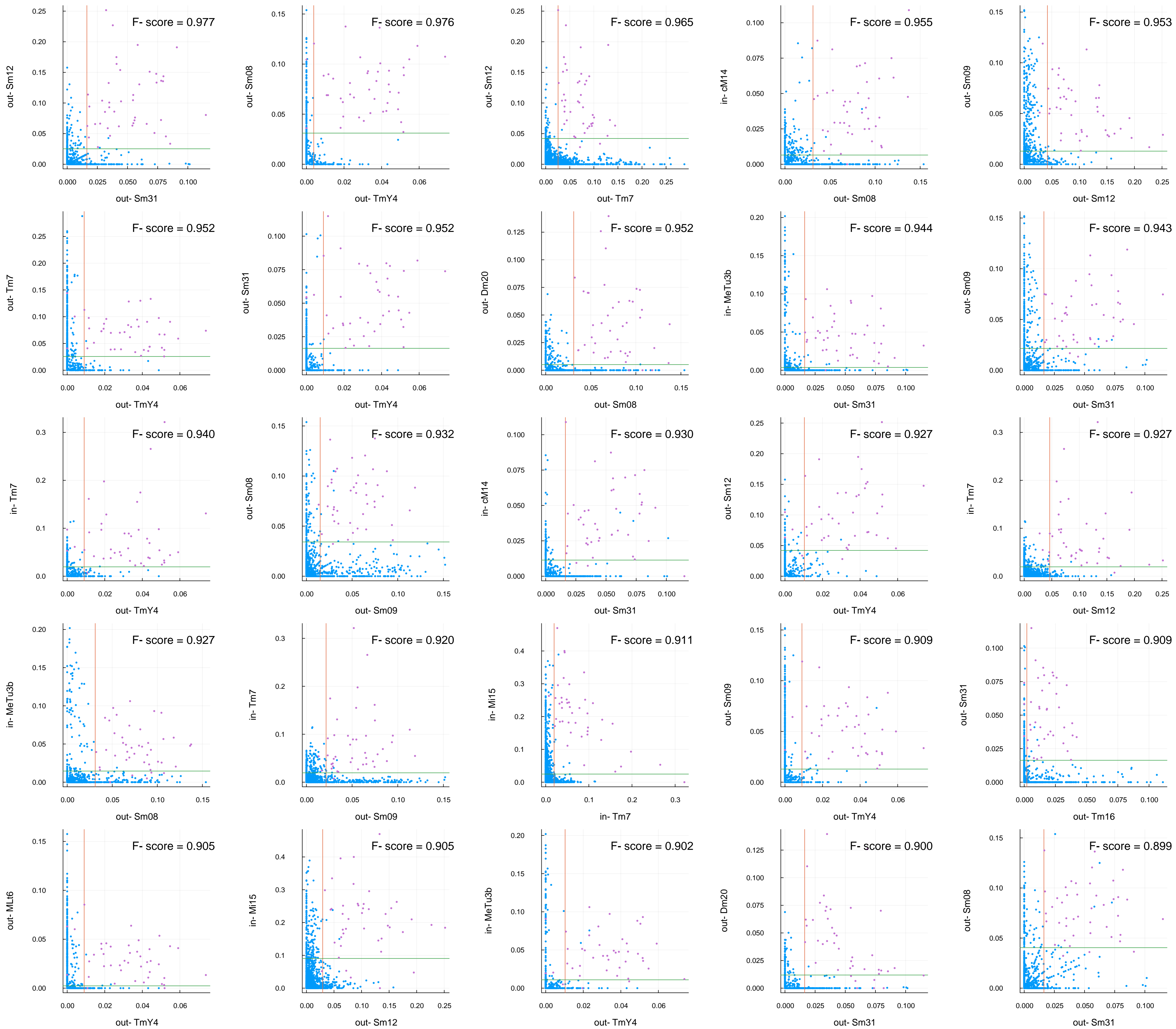

Supplement: Supplementary file 7 — Discriminating 2D projections for neuropil-intrinsic types. For each interneuron type, a pair of features is shown that can be used to discriminate that type from others in the same neuropil. Many although not all discriminations are highly accurate. Both intrinsic and boundary types are included as discriminative features. [file 41586_2024_7981_MOESM7_ESM.zip › DataS3/Sm10.pdf]

Sm11

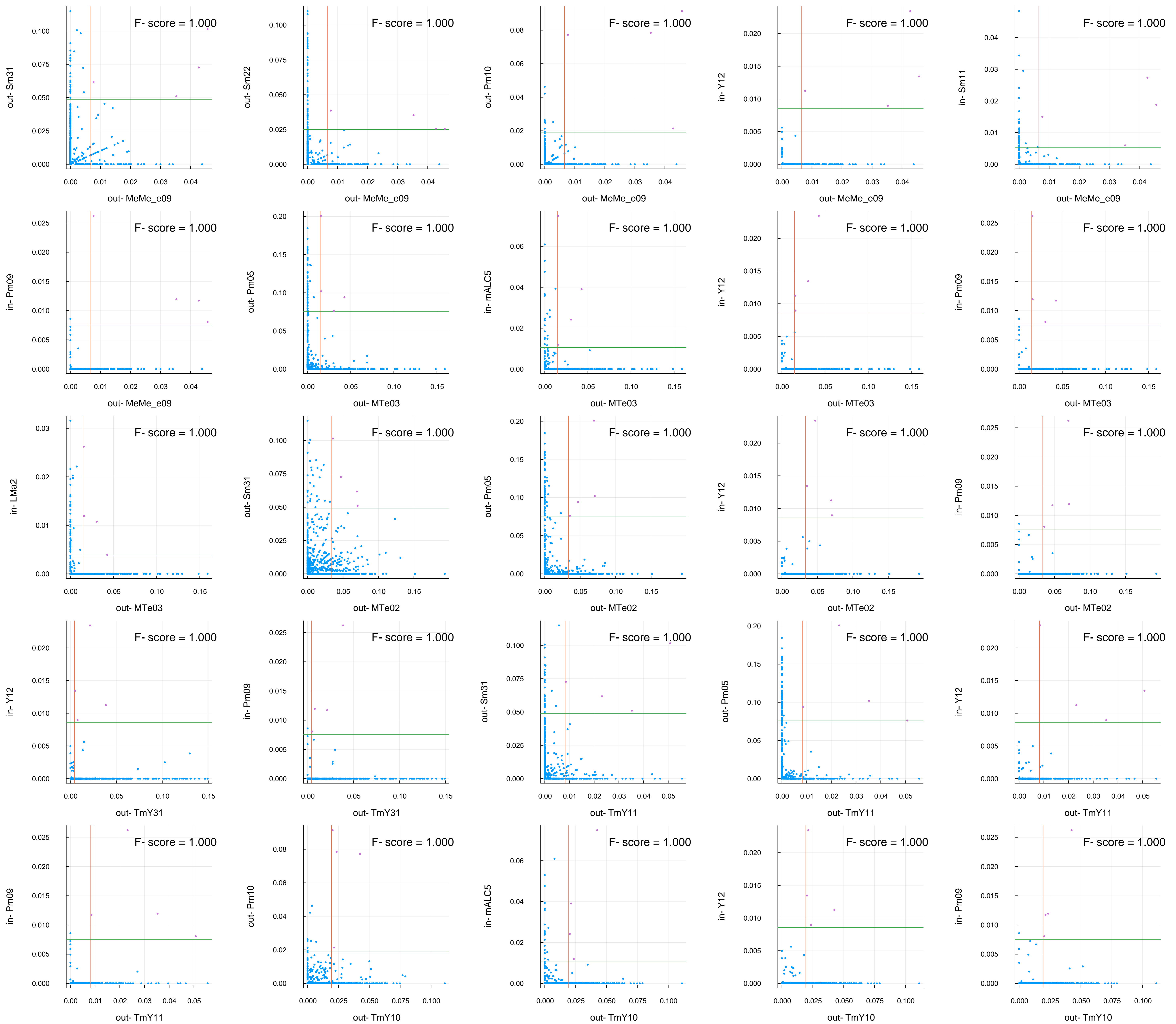

Supplement: Supplementary file 7 — Discriminating 2D projections for neuropil-intrinsic types. For each interneuron type, a pair of features is shown that can be used to discriminate that type from others in the same neuropil. Many although not all discriminations are highly accurate. Both intrinsic and boundary types are included as discriminative features. [file 41586_2024_7981_MOESM7_ESM.zip › DataS3/Sm11.pdf]

Sm12

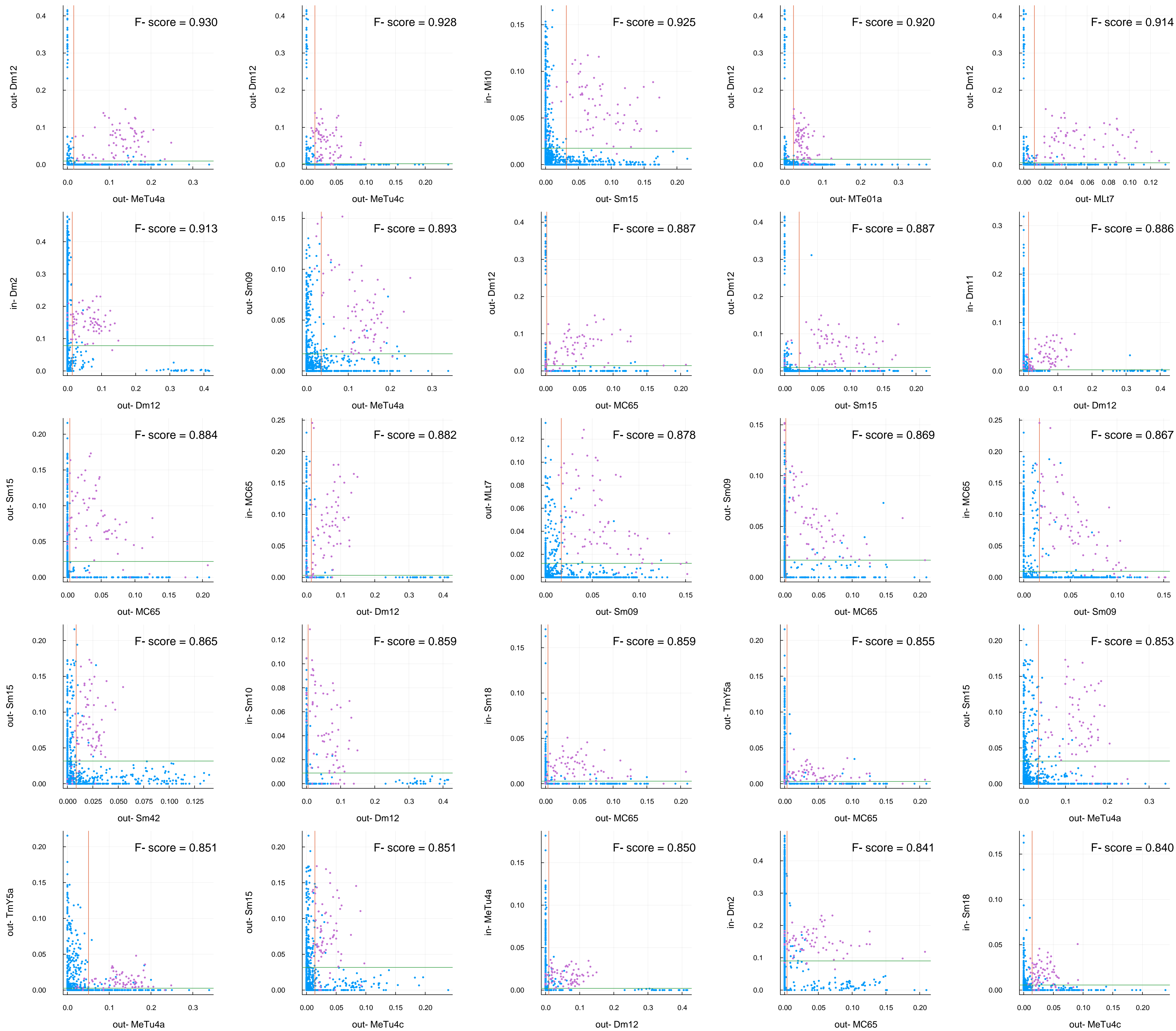

Supplement: Supplementary file 7 — Discriminating 2D projections for neuropil-intrinsic types. For each interneuron type, a pair of features is shown that can be used to discriminate that type from others in the same neuropil. Many although not all discriminations are highly accurate. Both intrinsic and boundary types are included as discriminative features. [file 41586_2024_7981_MOESM7_ESM.zip › DataS3/Sm12.pdf]

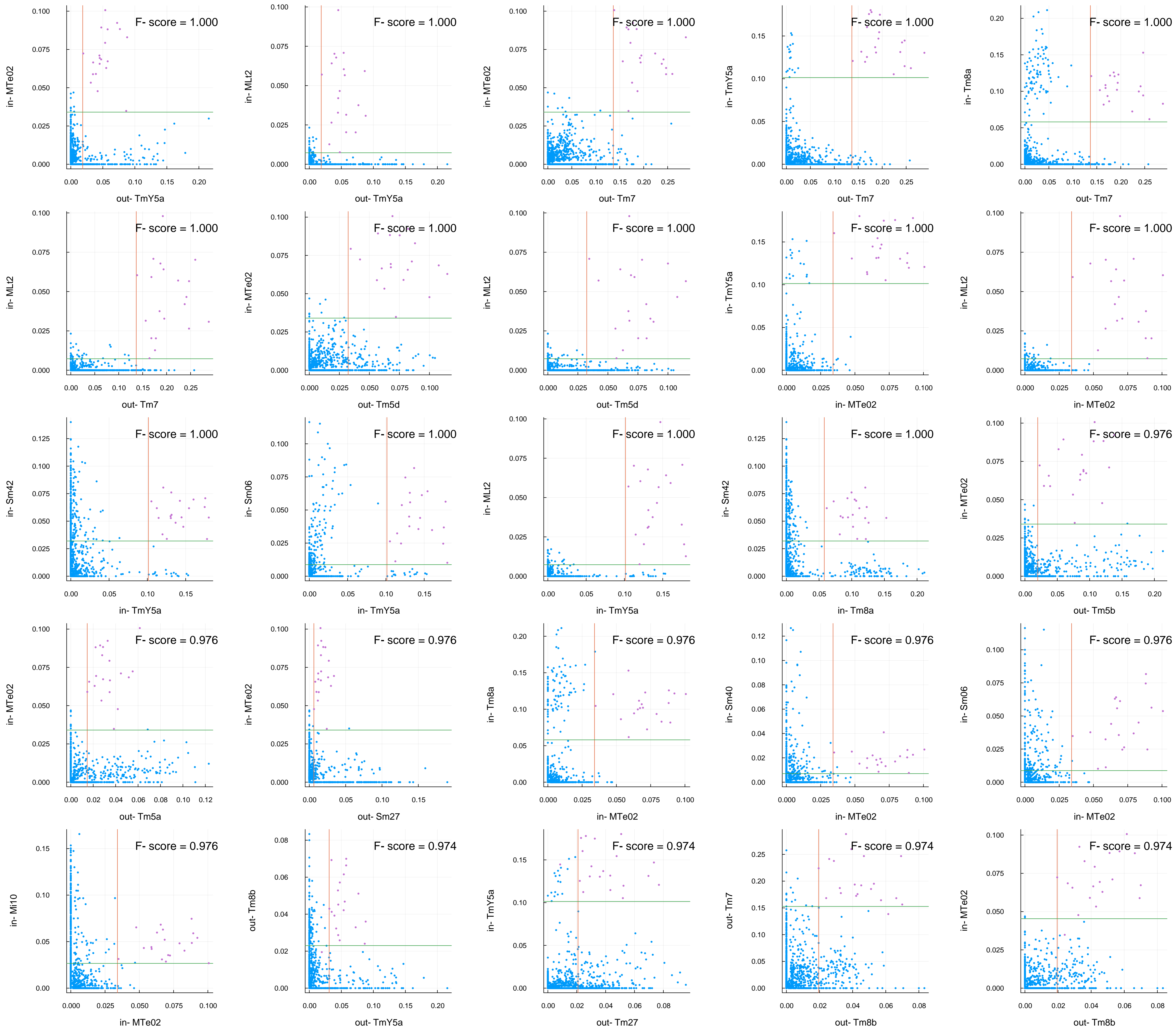

Supplement: Supplementary file 7 — Discriminating 2D projections for neuropil-intrinsic types. For each interneuron type, a pair of features is shown that can be used to discriminate that type from others in the same neuropil. Many although not all discriminations are highly accurate. Both intrinsic and boundary types are included as discriminative features. [file 41586_2024_7981_MOESM7_ESM.zip › DataS3/Sm13.pdf]

Sm14

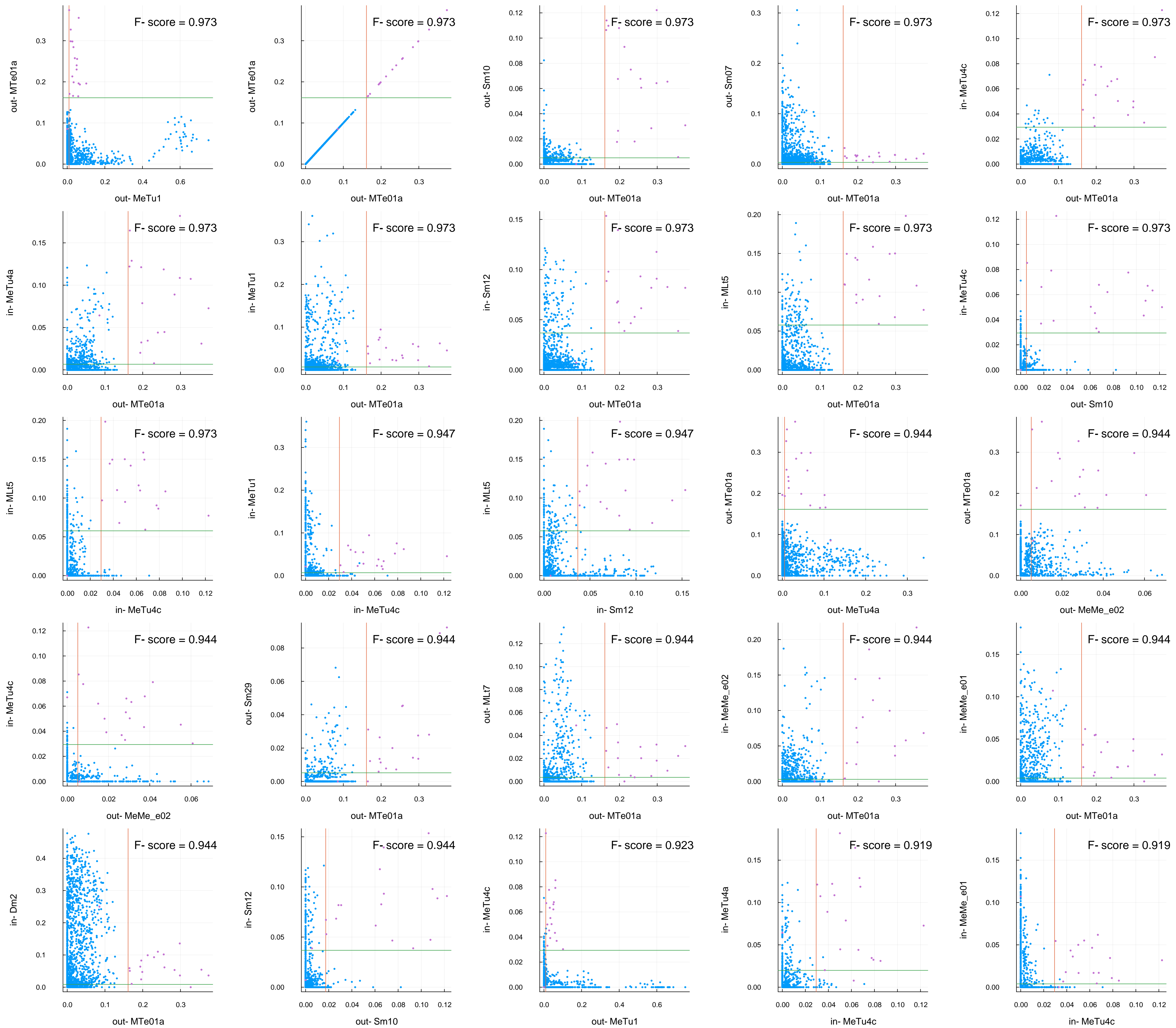

Supplement: Supplementary file 7 — Discriminating 2D projections for neuropil-intrinsic types. For each interneuron type, a pair of features is shown that can be used to discriminate that type from others in the same neuropil. Many although not all discriminations are highly accurate. Both intrinsic and boundary types are included as discriminative features. [file 41586_2024_7981_MOESM7_ESM.zip › DataS3/Sm14.pdf]

Sm15

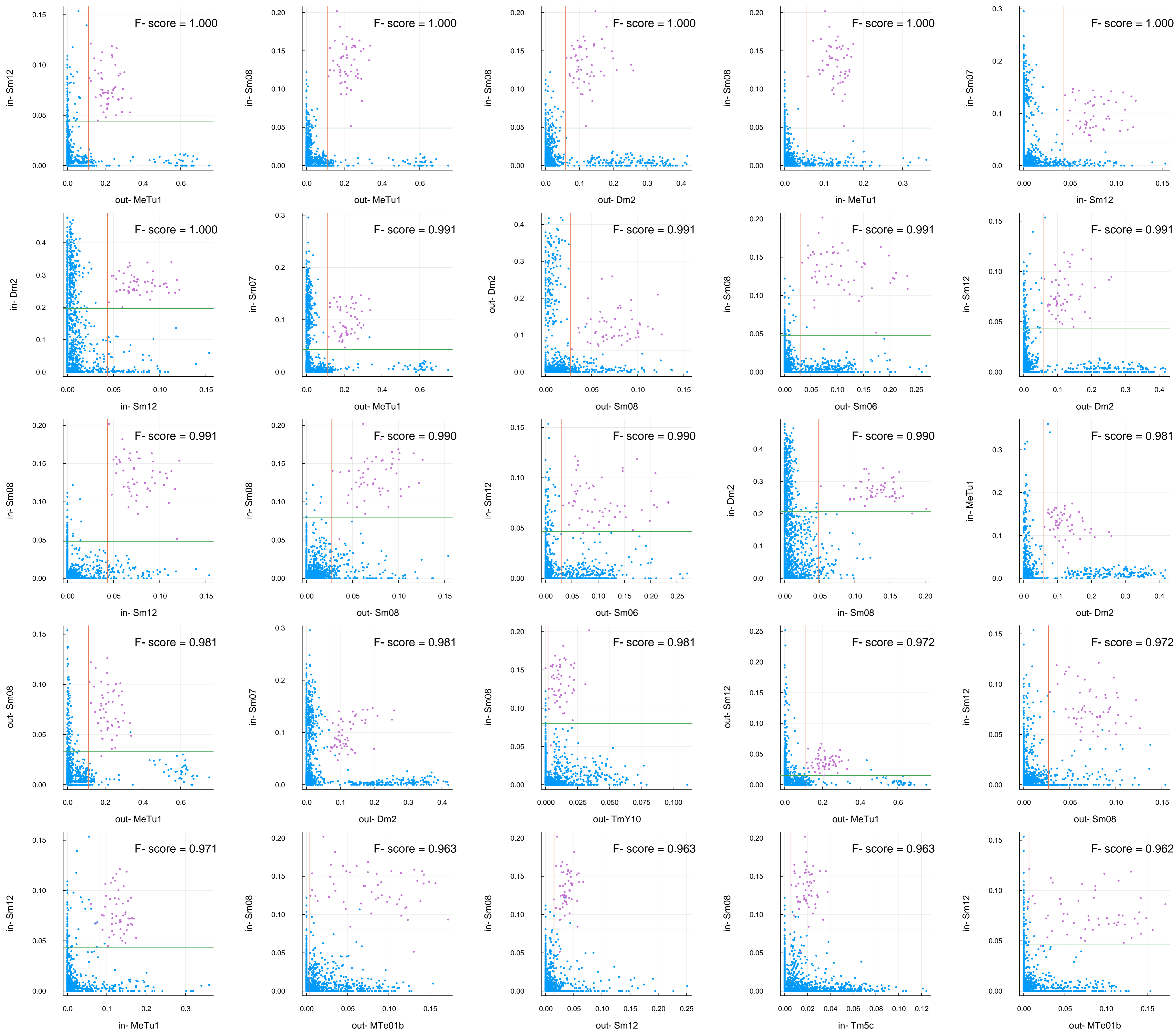

Supplement: Supplementary file 7 — Discriminating 2D projections for neuropil-intrinsic types. For each interneuron type, a pair of features is shown that can be used to discriminate that type from others in the same neuropil. Many although not all discriminations are highly accurate. Both intrinsic and boundary types are included as discriminative features. [file 41586_2024_7981_MOESM7_ESM.zip › DataS3/Sm15.pdf]

# Sm16

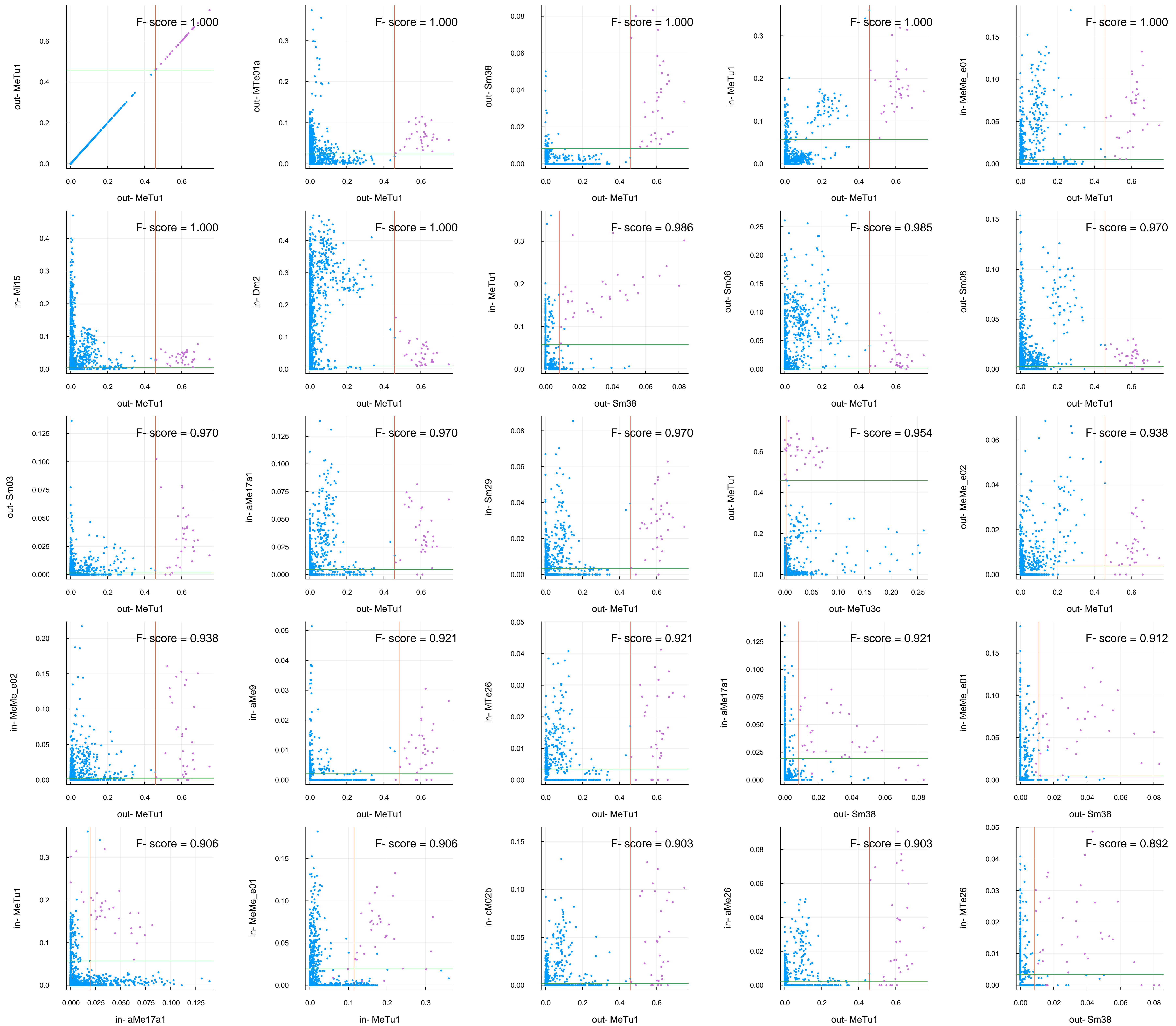

Supplement: Supplementary file 7 — Discriminating 2D projections for neuropil-intrinsic types. For each interneuron type, a pair of features is shown that can be used to discriminate that type from others in the same neuropil. Many although not all discriminations are highly accurate. Both intrinsic and boundary types are included as discriminative features. [file 41586_2024_7981_MOESM7_ESM.zip › DataS3/Sm16.pdf]

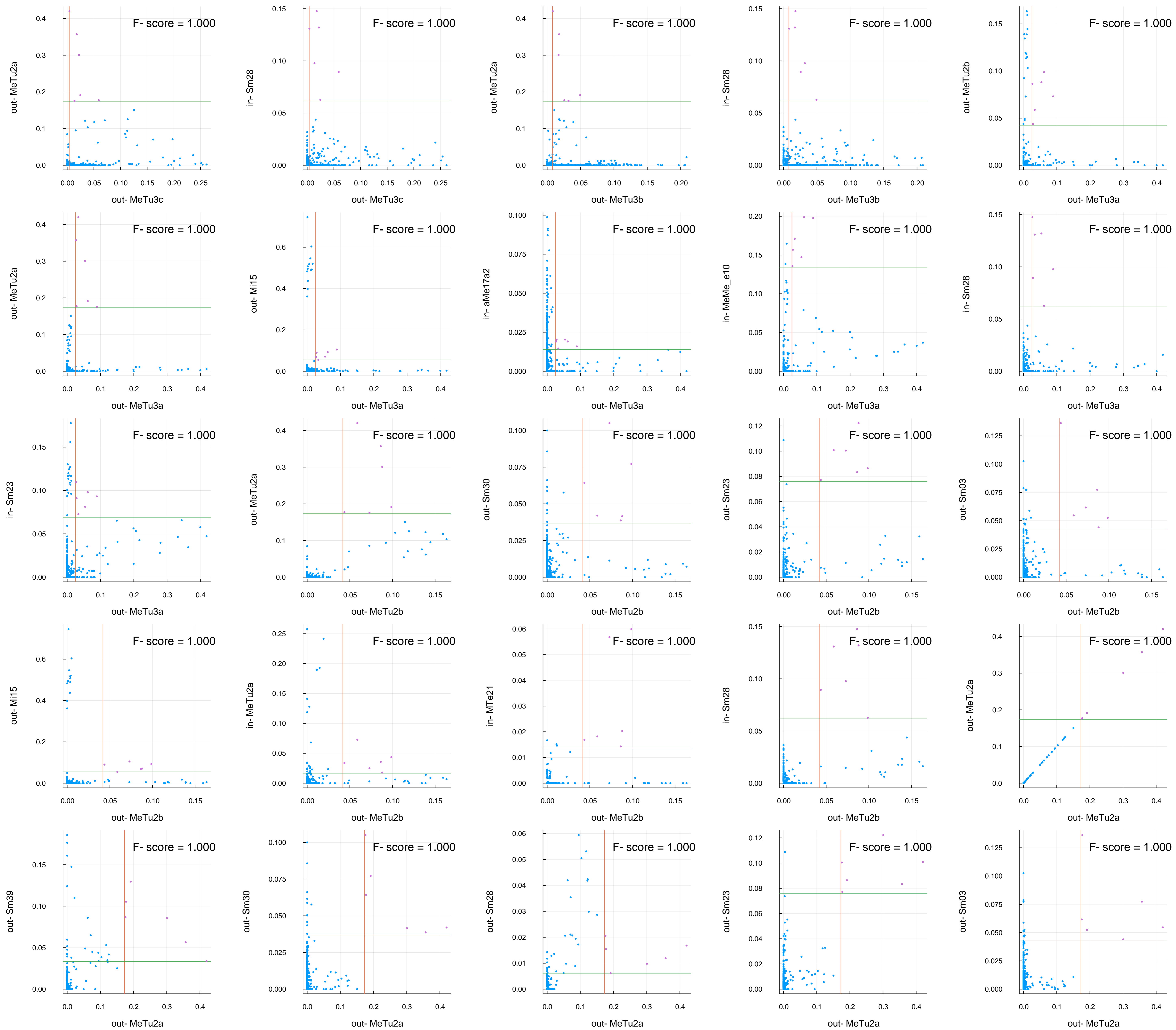

Supplement: Supplementary file 7 — Discriminating 2D projections for neuropil-intrinsic types. For each interneuron type, a pair of features is shown that can be used to discriminate that type from others in the same neuropil. Many although not all discriminations are highly accurate. Both intrinsic and boundary types are included as discriminative features. [file 41586_2024_7981_MOESM7_ESM.zip › DataS3/Sm17.pdf]

Sm18

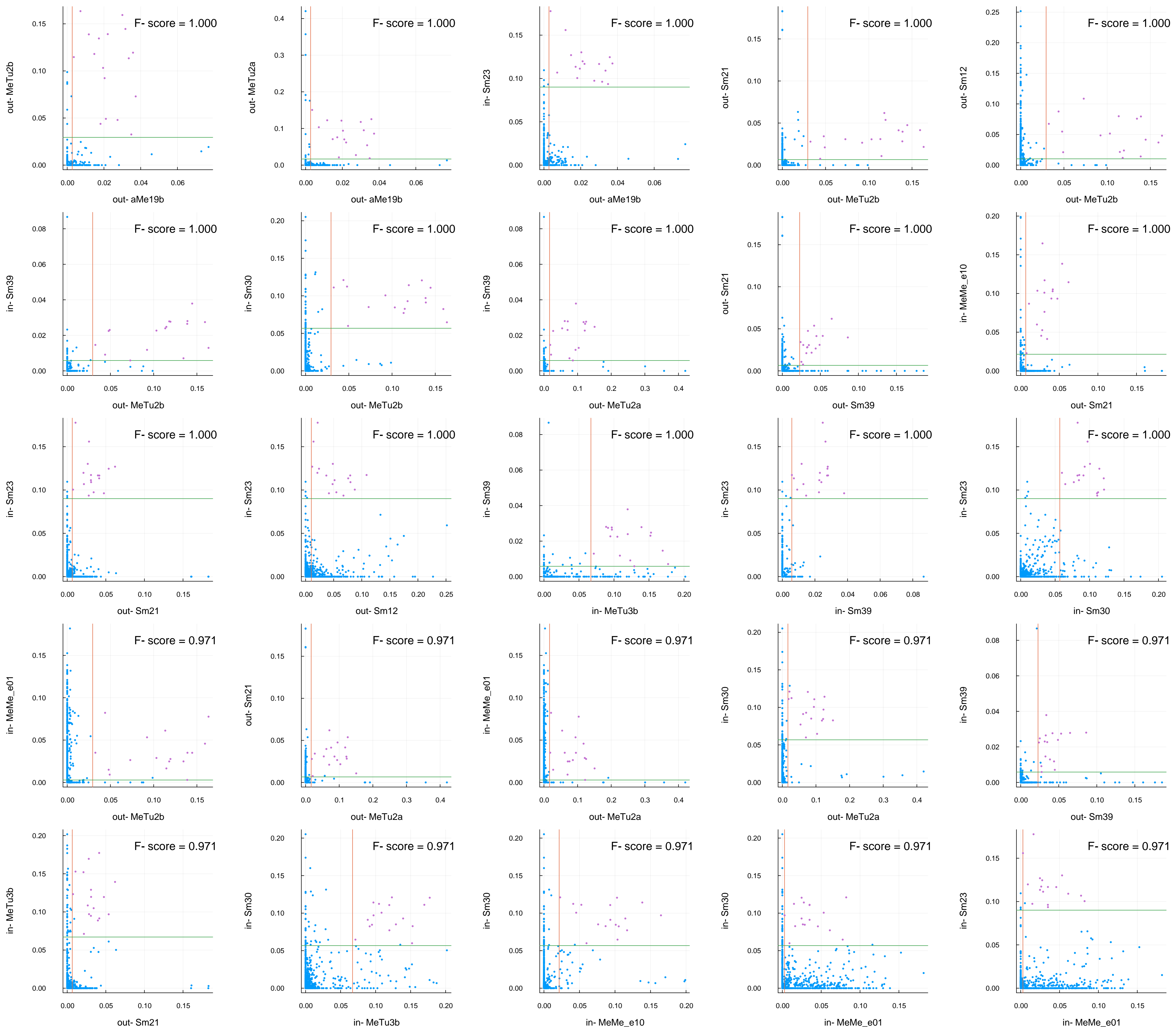

Supplement: Supplementary file 7 — Discriminating 2D projections for neuropil-intrinsic types. For each interneuron type, a pair of features is shown that can be used to discriminate that type from others in the same neuropil. Many although not all discriminations are highly accurate. Both intrinsic and boundary types are included as discriminative features. [file 41586_2024_7981_MOESM7_ESM.zip › DataS3/Sm18.pdf]

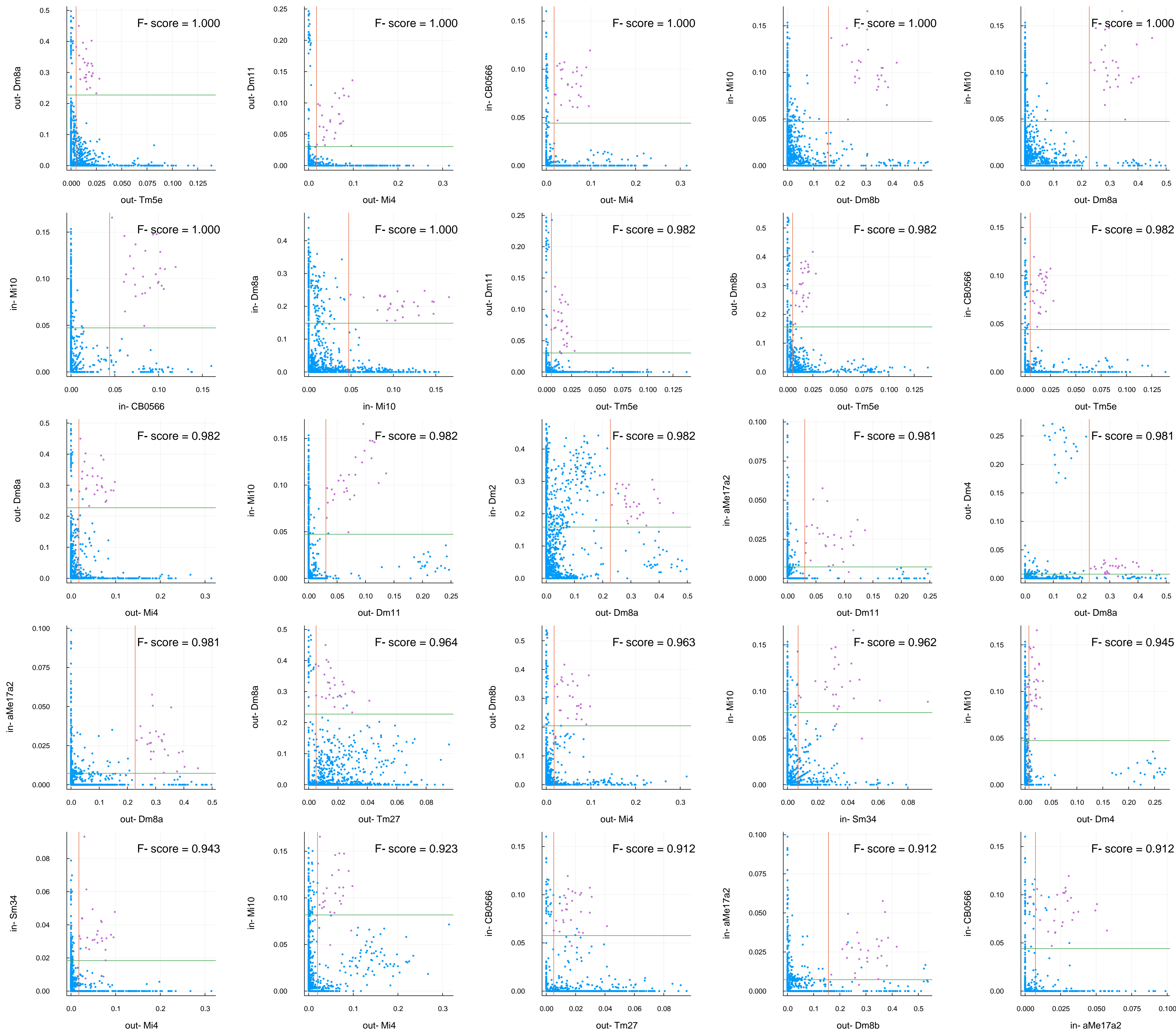

Supplement: Supplementary file 7 — Discriminating 2D projections for neuropil-intrinsic types. For each interneuron type, a pair of features is shown that can be used to discriminate that type from others in the same neuropil. Many although not all discriminations are highly accurate. Both intrinsic and boundary types are included as discriminative features. [file 41586_2024_7981_MOESM7_ESM.zip › DataS3/Sm19.pdf]

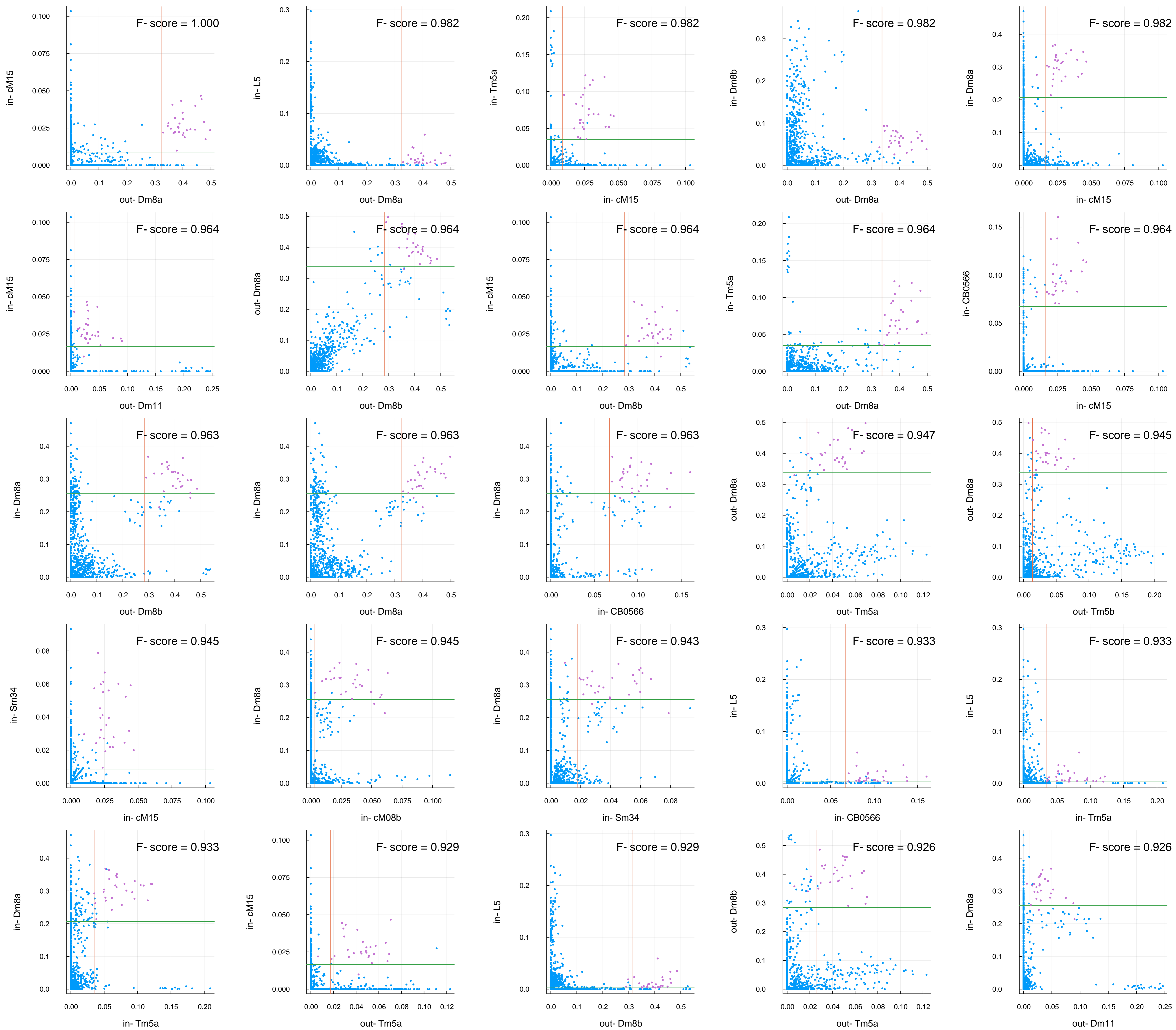

Supplement: Supplementary file 7 — Discriminating 2D projections for neuropil-intrinsic types. For each interneuron type, a pair of features is shown that can be used to discriminate that type from others in the same neuropil. Many although not all discriminations are highly accurate. Both intrinsic and boundary types are included as discriminative features. [file 41586_2024_7981_MOESM7_ESM.zip › DataS3/Sm20.pdf]

Sm21

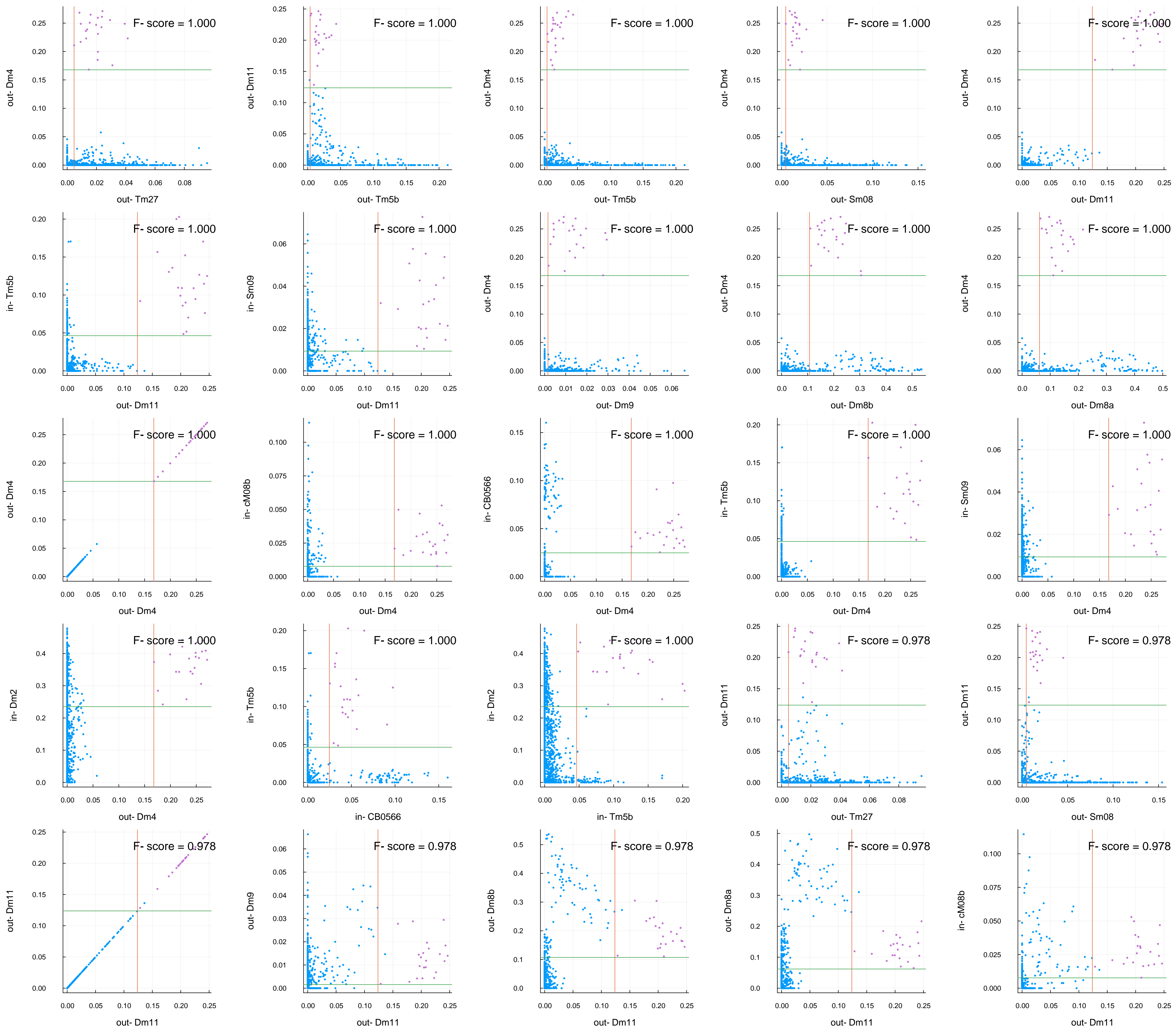

Supplement: Supplementary file 7 — Discriminating 2D projections for neuropil-intrinsic types. For each interneuron type, a pair of features is shown that can be used to discriminate that type from others in the same neuropil. Many although not all discriminations are highly accurate. Both intrinsic and boundary types are included as discriminative features. [file 41586_2024_7981_MOESM7_ESM.zip › DataS3/Sm21.pdf]

Sm22

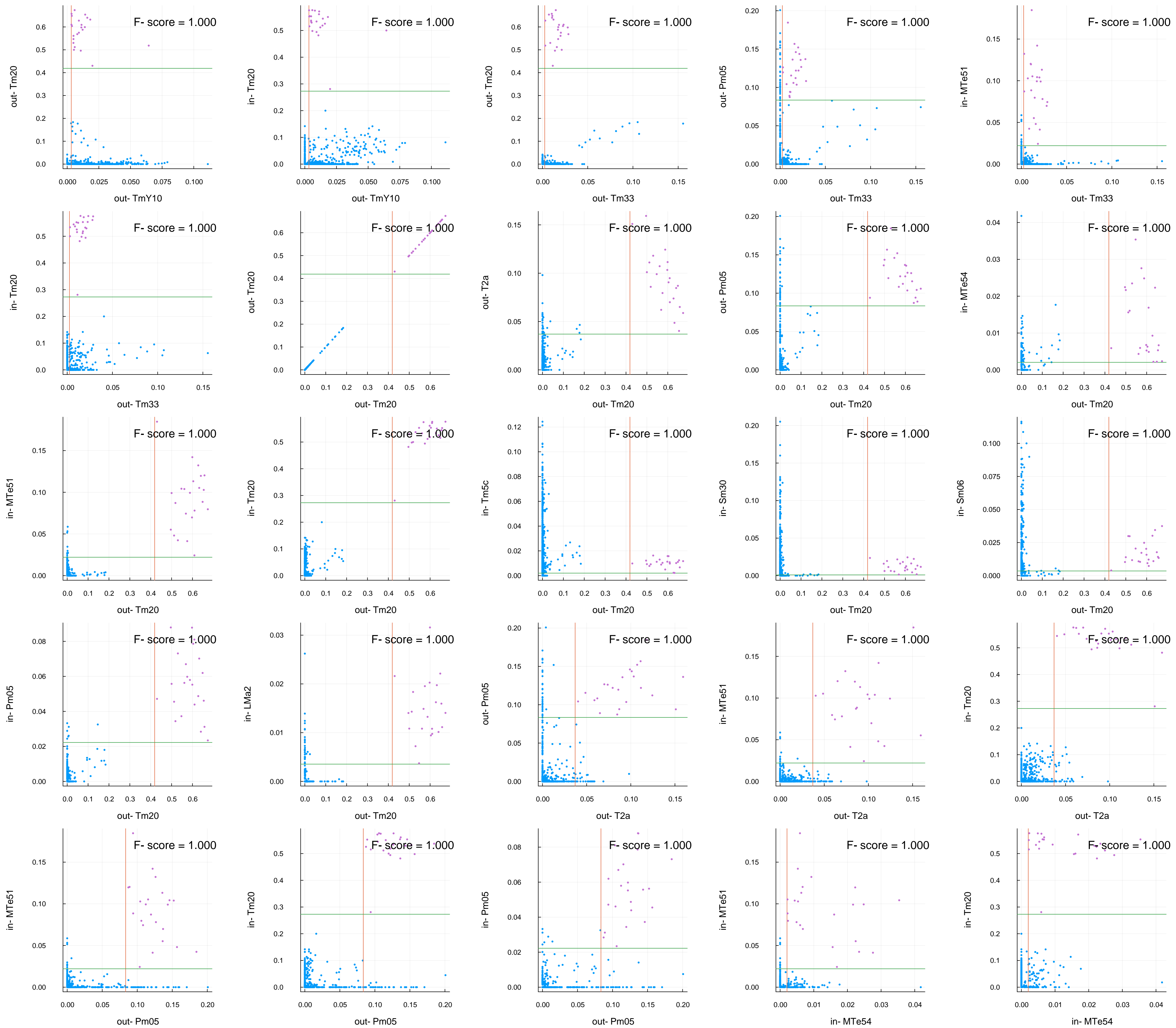

Supplement: Supplementary file 7 — Discriminating 2D projections for neuropil-intrinsic types. For each interneuron type, a pair of features is shown that can be used to discriminate that type from others in the same neuropil. Many although not all discriminations are highly accurate. Both intrinsic and boundary types are included as discriminative features. [file 41586_2024_7981_MOESM7_ESM.zip › DataS3/Sm22.pdf]
