# Supplementary material for: Neuronal parts list and wiring diagram for a visual system
Source: Nature. 2024 Oct 2;634(8032):166–80. doi: 10.1038/s41586-024-07981-1 (PMC11446827; doi:10.1038/s41586-024-07981-1)
Supplement: Supplementary file 7 — Discriminating 2D projections for neuropil-intrinsic types. For each interneuron type, a pair of features is shown that can be used to discriminate that type from others in the same neuropil. Many although not all discriminations are highly accurate. Both intrinsic and boundary types are included as discriminative features. [file 41586_2024_7981_MOESM7_ESM.zip › DataS3/Tm8a.pdf]

Tm8a

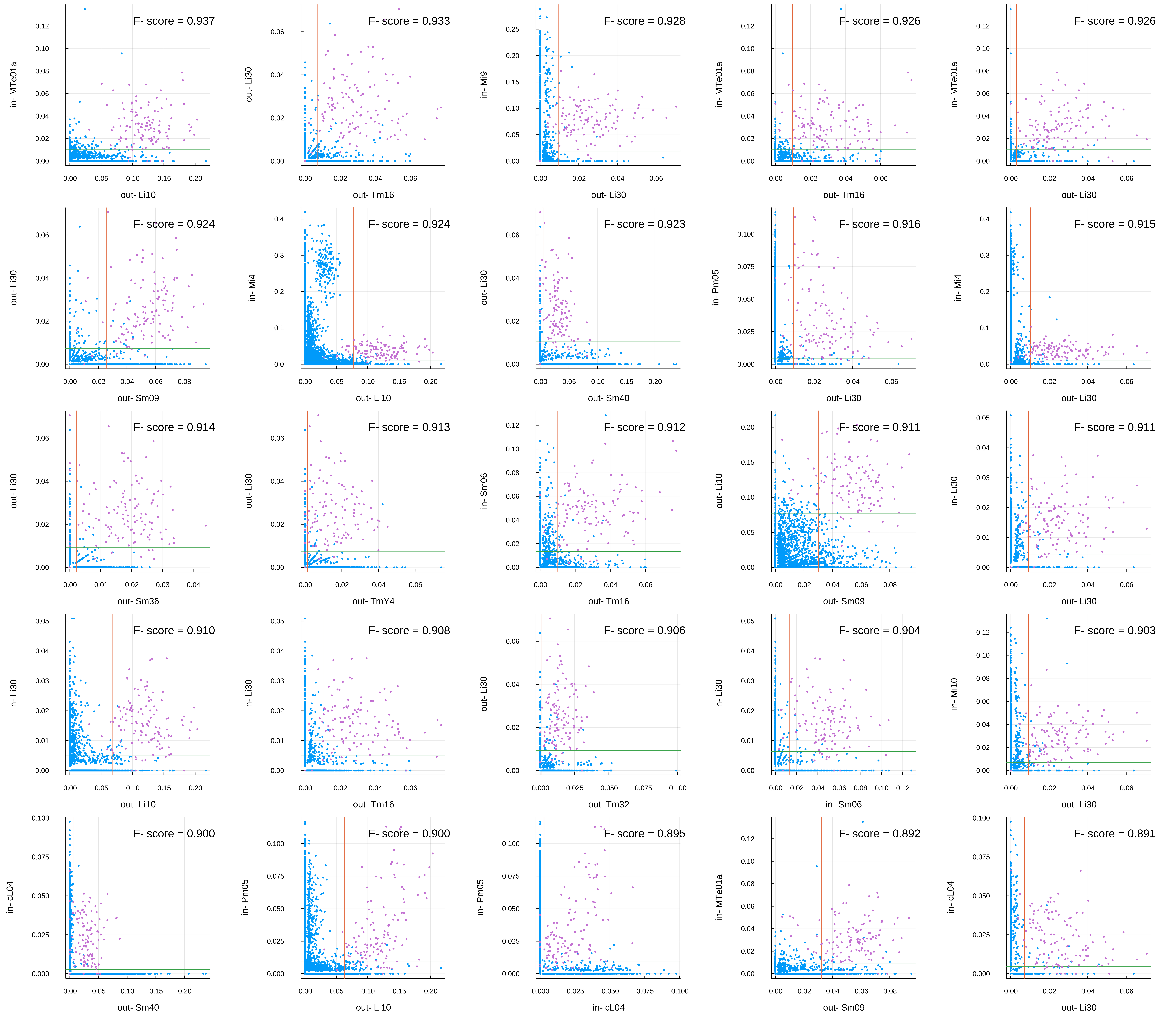

F- score = 0.937
in- MTe01a
out- Li10
F- score = 0.933
out- Li30
out- Tm16
F- score = 0.928
in- Mi9
out- Li30
F- score = 0.926
in- MTe01a
out- Tm16
F- score = 0.926
in- MTe01a
out- Li30
F- score = 0.924
out- Li30
out- Sm09
F- score = 0.924
in- Mi4
out- Li10
F- score = 0.923
out- Li30
out- Sm40
F- score = 0.916
in- Pm05
out- Li30
F- score = 0.915
in- Mi4
out- Li30
F- score = 0.914
out- Li30
out- Sm36
F- score = 0.913
out- Li30
out- TmY4
F- score = 0.912
in- Sm06
out- Tm16
F- score = 0.911
out- Li10
out- Sm09
F- score = 0.911
in- Li30
out- Li30
F- score = 0.910
in- Li30
out- Li10
F- score = 0.908
in- Li30
out- Tm16
F- score = 0.906
out- Li30
out- Tm32
F- score = 0.904
in- Li30
in- Sm06
F- score = 0.903
in- Mi10
out- Li30
F- score = 0.900
in- cL04
out- Sm40
F- score = 0.900
in- Pm05
out- Li10
F- score = 0.895
in- Pm05
in- cL04
F- score = 0.892
in- MTe01a
out- Sm09
F- score = 0.891
in- cL04
out- Li30
