# Supplementary material for: Neuronal parts list and wiring diagram for a visual system
Source: Nature. 2024 Oct 2;634(8032):166–80. doi: 10.1038/s41586-024-07981-1 (PMC11446827; doi:10.1038/s41586-024-07981-1)
Supplement: Supplementary file 7 — Discriminating 2D projections for neuropil-intrinsic types. For each interneuron type, a pair of features is shown that can be used to discriminate that type from others in the same neuropil. Many although not all discriminations are highly accurate. Both intrinsic and boundary types are included as discriminative features. [file 41586_2024_7981_MOESM7_ESM.zip › DataS3/Tm8b.pdf]

Tm8b

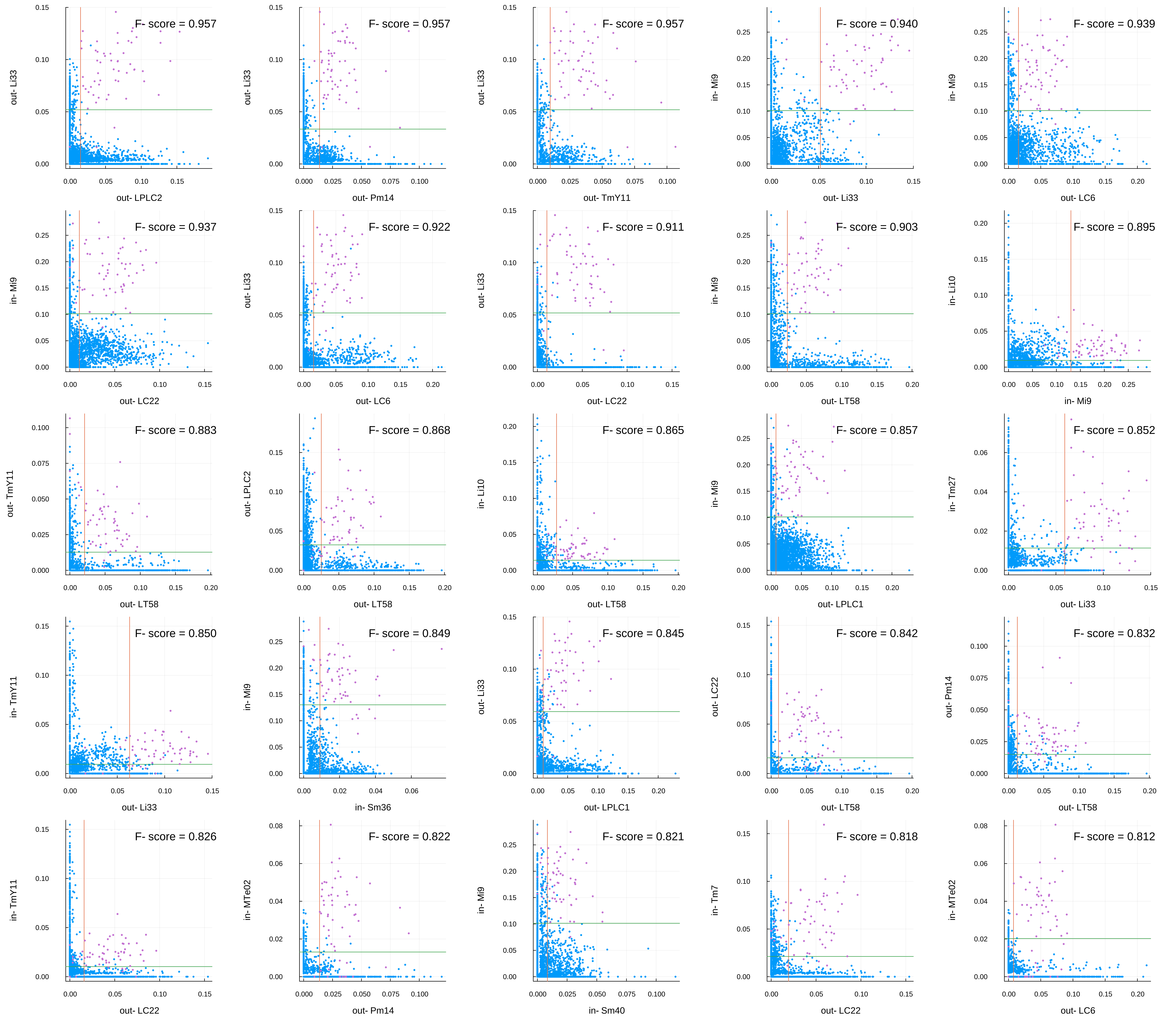
F- score = 0.957
out- Li33
out- LPLC2
F- score = 0.957
out- Li33
out- Pm14
F- score = 0.957
out- Li33
out- TmY11
F- score = 0.940
in- Mi9
out- Li33
F- score = 0.939
in- Mi9
out- LC6
F- score = 0.937
in- Mi9
out- LC22
F- score = 0.922
out- Li33
out- LC6
F- score = 0.911
out- Li33
out- LC22
F- score = 0.903
in- Mi9
out- LT58
F- score = 0.895
in- Li10
in- Mi9
F- score = 0.883
out- TmY11
out- LT58
F- score = 0.868
out- LPLC2
out- LT58
F- score = 0.865
in- Li10
out- LT58
F- score = 0.857
in- Mi9
out- LPLC1
F- score = 0.852
in- Tm27
out- Li33
F- score = 0.850
in- TmY11
out- Li33
F- score = 0.849
in- Mi9
in- Sm36
F- score = 0.845
out- Li33
out- LPLC1
F- score = 0.842
out- LC22
out- LT58
F- score = 0.832
out- Pm14
out- LT58
F- score = 0.826
in- TmY11
out- LC22
F- score = 0.822
in- MTe02
out- Pm14
F- score = 0.821
in- Mi9
in- Sm40
F- score = 0.818
in- Tm7
out- LC22
F- score = 0.812
in- MTe02
out- LC6
