# Supplementary material for: Neuronal parts list and wiring diagram for a visual system
Source: Nature. 2024 Oct 2;634(8032):166–80. doi: 10.1038/s41586-024-07981-1 (PMC11446827; doi:10.1038/s41586-024-07981-1)
Supplement: Supplementary file 7 — Discriminating 2D projections for neuropil-intrinsic types. For each interneuron type, a pair of features is shown that can be used to discriminate that type from others in the same neuropil. Many although not all discriminations are highly accurate. Both intrinsic and boundary types are included as discriminative features. [file 41586_2024_7981_MOESM7_ESM.zip › DataS3/Tm9.pdf]

Tm9

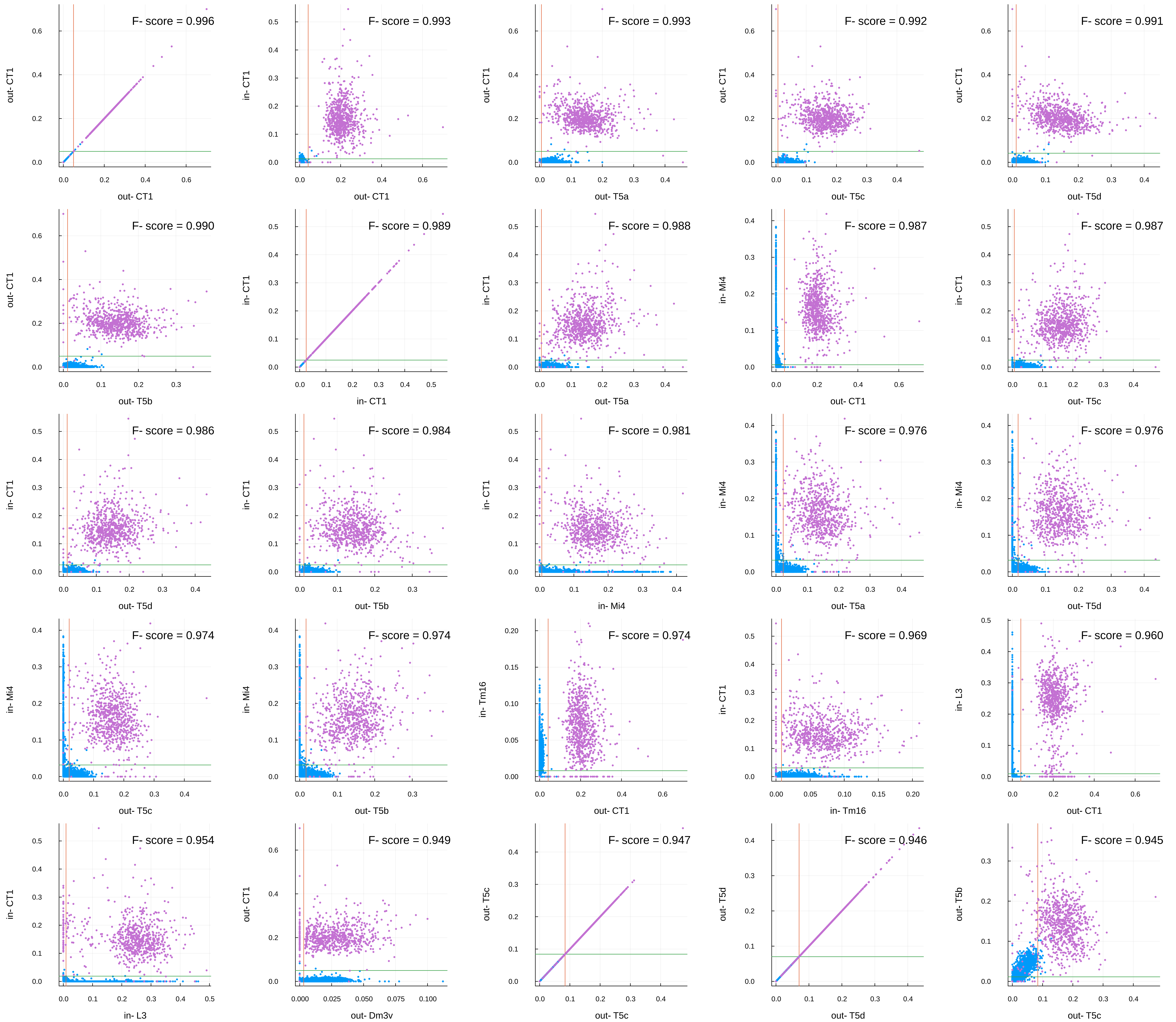
F- score = 0.996
out- CT1
out- CT1
F- score = 0.993
in- CT1
out- CT1
F- score = 0.993
out- CT1
out- T5a
F- score = 0.992
out- CT1
out- T5c
F- score = 0.991
out- CT1
out- T5d
F- score = 0.990
out- CT1
out- T5b
F- score = 0.989
in- CT1
in- CT1
F- score = 0.988
in- CT1
out- T5a
F- score = 0.987
in- Mi4
out- CT1
F- score = 0.987
in- CT1
out- T5c
F- score = 0.986
in- CT1
out- T5d
F- score = 0.984
in- CT1
out- T5b
F- score = 0.981
in- CT1
in- Mi4
F- score = 0.976
in- Mi4
out- T5a
F- score = 0.976
in- Mi4
out- T5d
F- score = 0.974
in- Mi4
out- T5c
F- score = 0.974
in- Mi4
out- T5b
F- score = 0.974
in- Tm16
out- CT1
F- score = 0.969
in- CT1
in- Tm16
F- score = 0.960
in- L3
out- CT1
F- score = 0.954
in- CT1
in- L3
F- score = 0.949
out- CT1
out- Dm3v
F- score = 0.947
out- T5c
out- T5c
F- score = 0.946
out- T5d
out- T5d
F- score = 0.945
out- T5b
out- T5c
