# Supplementary material for: Neuronal parts list and wiring diagram for a visual system
Source: Nature. 2024 Oct 2;634(8032):166–80. doi: 10.1038/s41586-024-07981-1 (PMC11446827; doi:10.1038/s41586-024-07981-1)
Supplement: Supplementary file 7 — Discriminating 2D projections for neuropil-intrinsic types. For each interneuron type, a pair of features is shown that can be used to discriminate that type from others in the same neuropil. Many although not all discriminations are highly accurate. Both intrinsic and boundary types are included as discriminative features. [file 41586_2024_7981_MOESM7_ESM.zip › DataS3/TmY10.pdf]

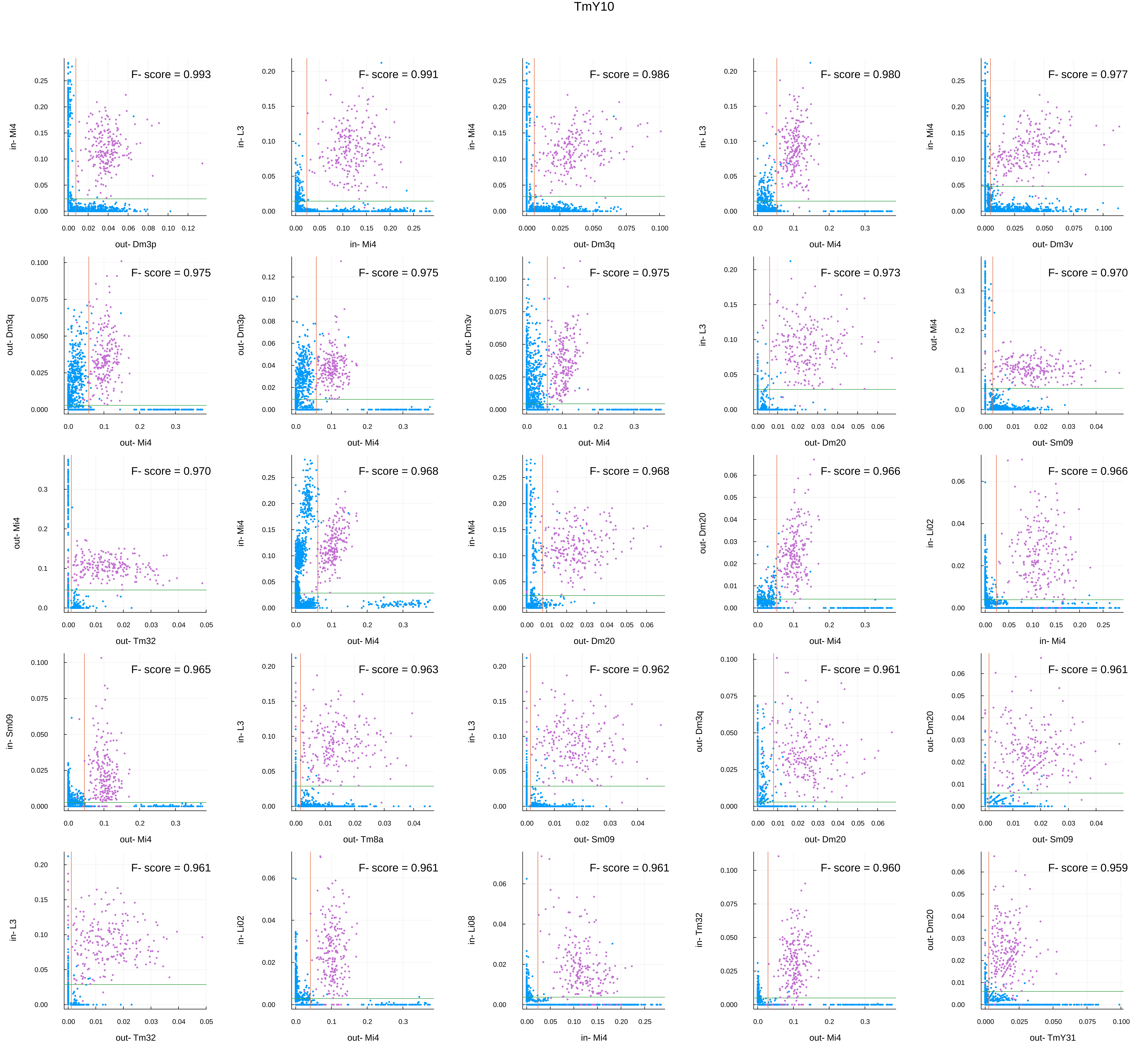
TmY10
F- score = 0.993
in- Mi4
out- Dm3p
F- score = 0.991
in- L3
in- Mi4
F- score = 0.986
in- Mi4
out- Dm3q
F- score = 0.980
in- L3
out- Mi4
F- score = 0.977
in- Mi4
out- Dm3v
F- score = 0.975
out- Dm3q
out- Mi4
F- score = 0.975
out- Dm3p
out- Mi4
F- score = 0.975
out- Dm3v
out- Mi4
F- score = 0.973
in- L3
out- Dm20
F- score = 0.970
out- Mi4
out- Sm09
F- score = 0.970
out- Mi4
out- Tm32
F- score = 0.968
in- Mi4
out- Mi4
F- score = 0.968
in- Mi4
out- Dm20
F- score = 0.966
out- Dm20
out- Mi4
F- score = 0.966
in- Li02
in- Mi4
F- score = 0.965
in- Sm09
out- Mi4
F- score = 0.963
in- L3
out- Tm8a
F- score = 0.962
in- L3
out- Sm09
F- score = 0.961
out- Dm3q
out- Dm20
F- score = 0.961
out- Dm20
out- Sm09
F- score = 0.961
in- L3
out- Tm32
F- score = 0.961
in- Li02
out- Mi4
F- score = 0.961
in- Li08
in- Mi4
F- score = 0.960
in- Tm32
out- Mi4
F- score = 0.959
out- Dm20
out- TmY31
