# Supplementary material for: Neuronal parts list and wiring diagram for a visual system
Source: Nature. 2024 Oct 2;634(8032):166–80. doi: 10.1038/s41586-024-07981-1 (PMC11446827; doi:10.1038/s41586-024-07981-1)
Supplement: Supplementary file 7 — Discriminating 2D projections for neuropil-intrinsic types. For each interneuron type, a pair of features is shown that can be used to discriminate that type from others in the same neuropil. Many although not all discriminations are highly accurate. Both intrinsic and boundary types are included as discriminative features. [file 41586_2024_7981_MOESM7_ESM.zip › DataS3/TmY11.pdf]

TmY11

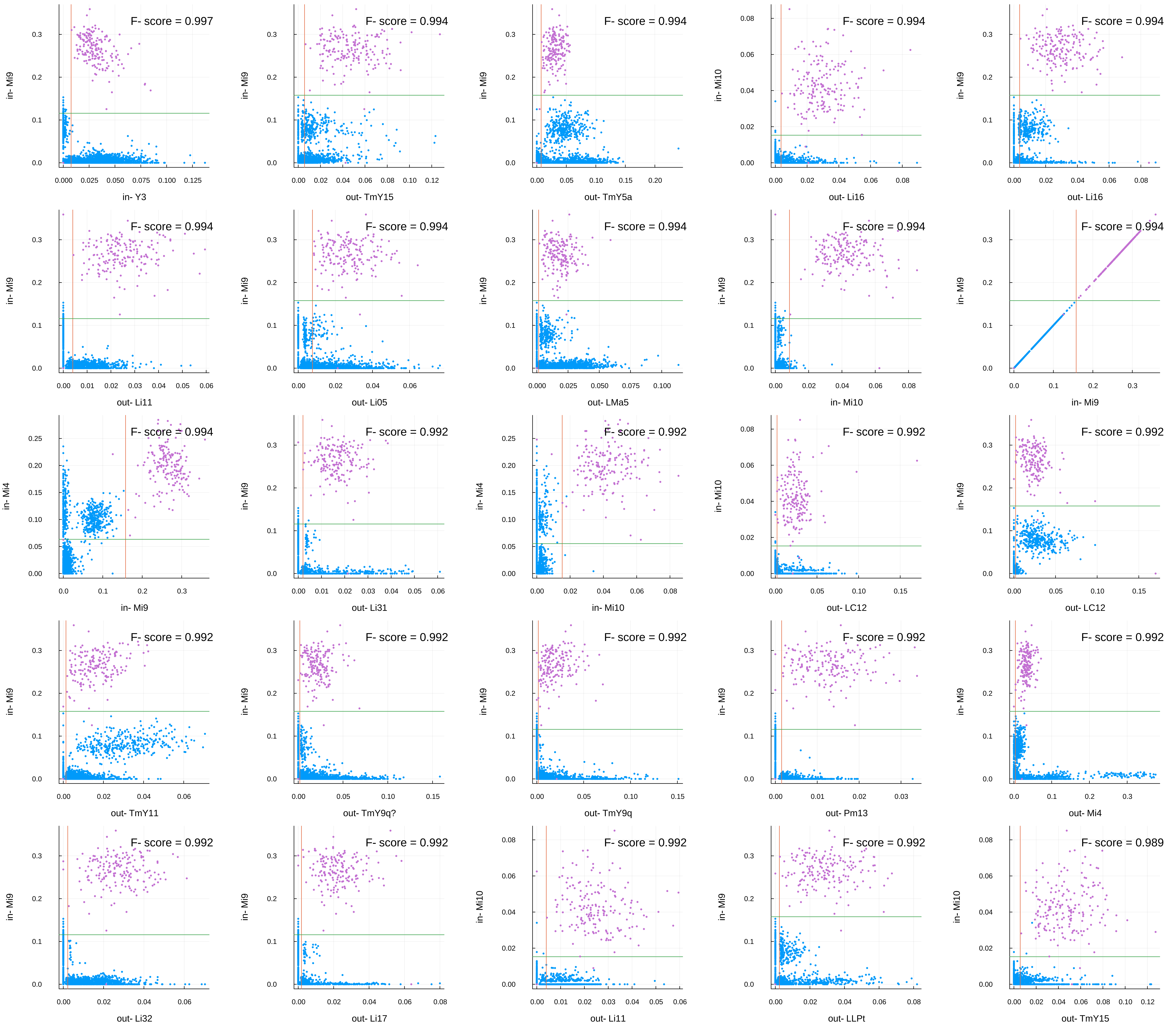

F- score = 0.997
in- Mi9
in- Y3
F- score = 0.994
in- Mi9
out- TmY15
F- score = 0.994
in- Mi9
out- TmY5a
F- score = 0.994
in- Mi10
out- Li16
F- score = 0.994
in- Mi9
out- Li16
F- score = 0.994
in- Mi9
out- Li11
F- score = 0.994
in- Mi9
out- Li05
F- score = 0.994
in- Mi9
out- LMa5
F- score = 0.994
in- Mi9
in- Mi10
F- score = 0.994
in- Mi9
in- Mi9
F- score = 0.994
in- Mi4
in- Mi9
F- score = 0.992
in- Mi9
out- Li31
F- score = 0.992
in- Mi4
in- Mi10
F- score = 0.992
in- Mi10
out- LC12
F- score = 0.992
in- Mi9
out- LC12
F- score = 0.992
in- Mi9
out- TmY11
F- score = 0.992
in- Mi9
out- TmY9q?
F- score = 0.992
in- Mi9
out- TmY9q
F- score = 0.992
in- Mi9
out- Pm13
F- score = 0.992
in- Mi9
out- Mi4
F- score = 0.992
in- Mi9
out- Li32
F- score = 0.992
in- Mi9
out- Li17
F- score = 0.992
in- Mi10
out- Li11
F- score = 0.992
in- Mi9
out- LLPt
F- score = 0.989
in- Mi10
out- TmY15
