# Supplementary material for: Neuronal parts list and wiring diagram for a visual system
Source: Nature. 2024 Oct 2;634(8032):166–80. doi: 10.1038/s41586-024-07981-1 (PMC11446827; doi:10.1038/s41586-024-07981-1)
Supplement: Supplementary file 7 — Discriminating 2D projections for neuropil-intrinsic types. For each interneuron type, a pair of features is shown that can be used to discriminate that type from others in the same neuropil. Many although not all discriminations are highly accurate. Both intrinsic and boundary types are included as discriminative features. [file 41586_2024_7981_MOESM7_ESM.zip › DataS3/TmY14.pdf]

TmY14

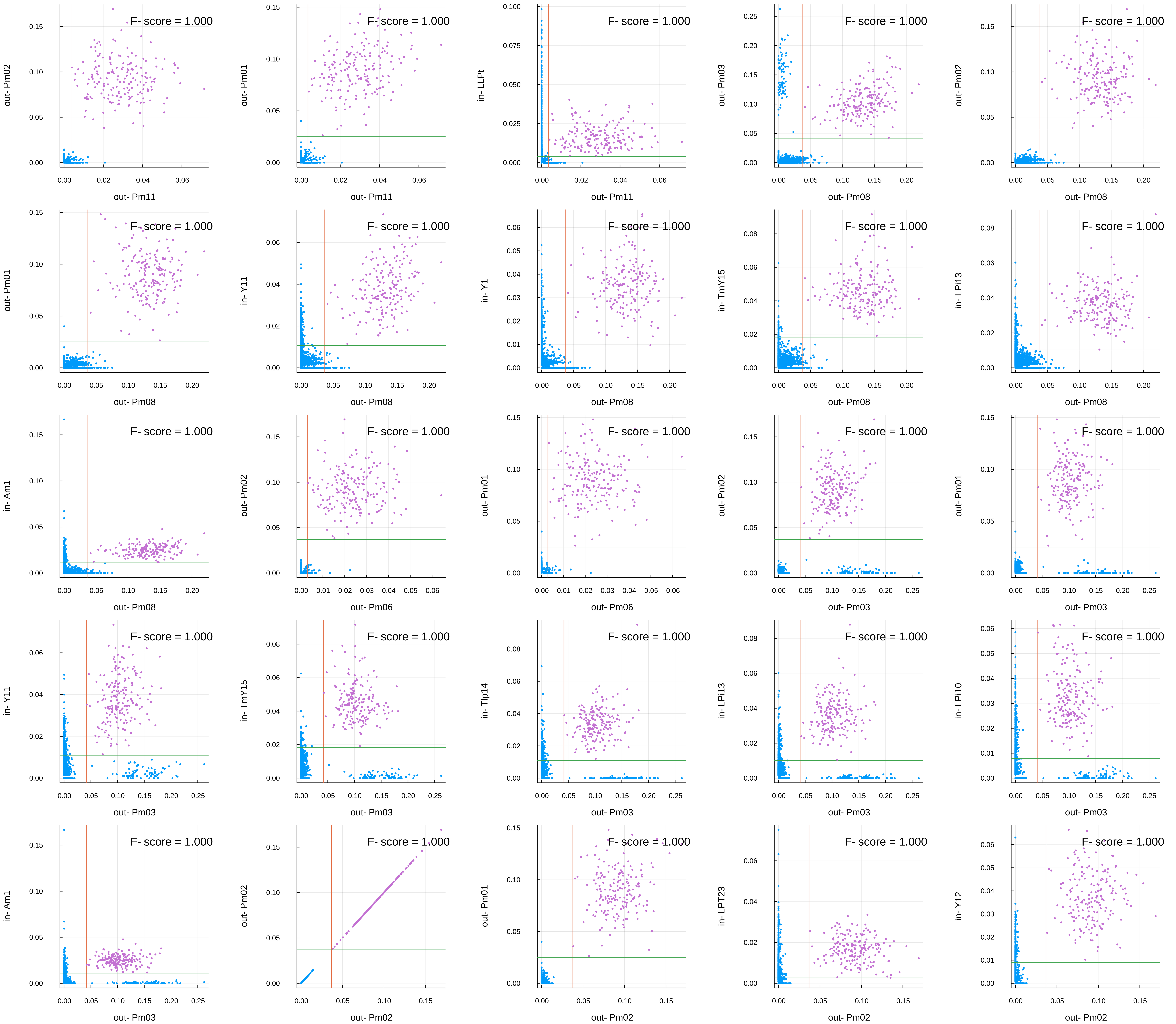
F- score = 1.000
out- Pm02
out- Pm11
out- Pm01
out- Pm11
in- LLPt
out- Pm11
out- Pm03
out- Pm08
out- Pm02
out- Pm08
out- Pm01
out- Pm08
in- Y11
out- Pm08
in- Y1
out- Pm08
in- TmY15
out- Pm08
in- LPi13
out- Pm08
in- Am1
out- Pm08
out- Pm02
out- Pm06
out- Pm01
out- Pm06
out- Pm02
out- Pm03
out- Pm01
out- Pm03
in- Y11
out- Pm03
in- TmY15
out- Pm03
in- Tlp14
out- Pm03
in- LPi13
out- Pm03
in- LPi10
out- Pm03
in- Am1
out- Pm03
out- Pm02
out- Pm02
out- Pm01
out- Pm02
in- LPT23
out- Pm02
in- Y12
out- Pm02
