# Supplementary material for: Neuronal parts list and wiring diagram for a visual system
Source: Nature. 2024 Oct 2;634(8032):166–80. doi: 10.1038/s41586-024-07981-1 (PMC11446827; doi:10.1038/s41586-024-07981-1)
Supplement: Supplementary file 7 — Discriminating 2D projections for neuropil-intrinsic types. For each interneuron type, a pair of features is shown that can be used to discriminate that type from others in the same neuropil. Many although not all discriminations are highly accurate. Both intrinsic and boundary types are included as discriminative features. [file 41586_2024_7981_MOESM7_ESM.zip › DataS3/TmY15.pdf]

TmY15

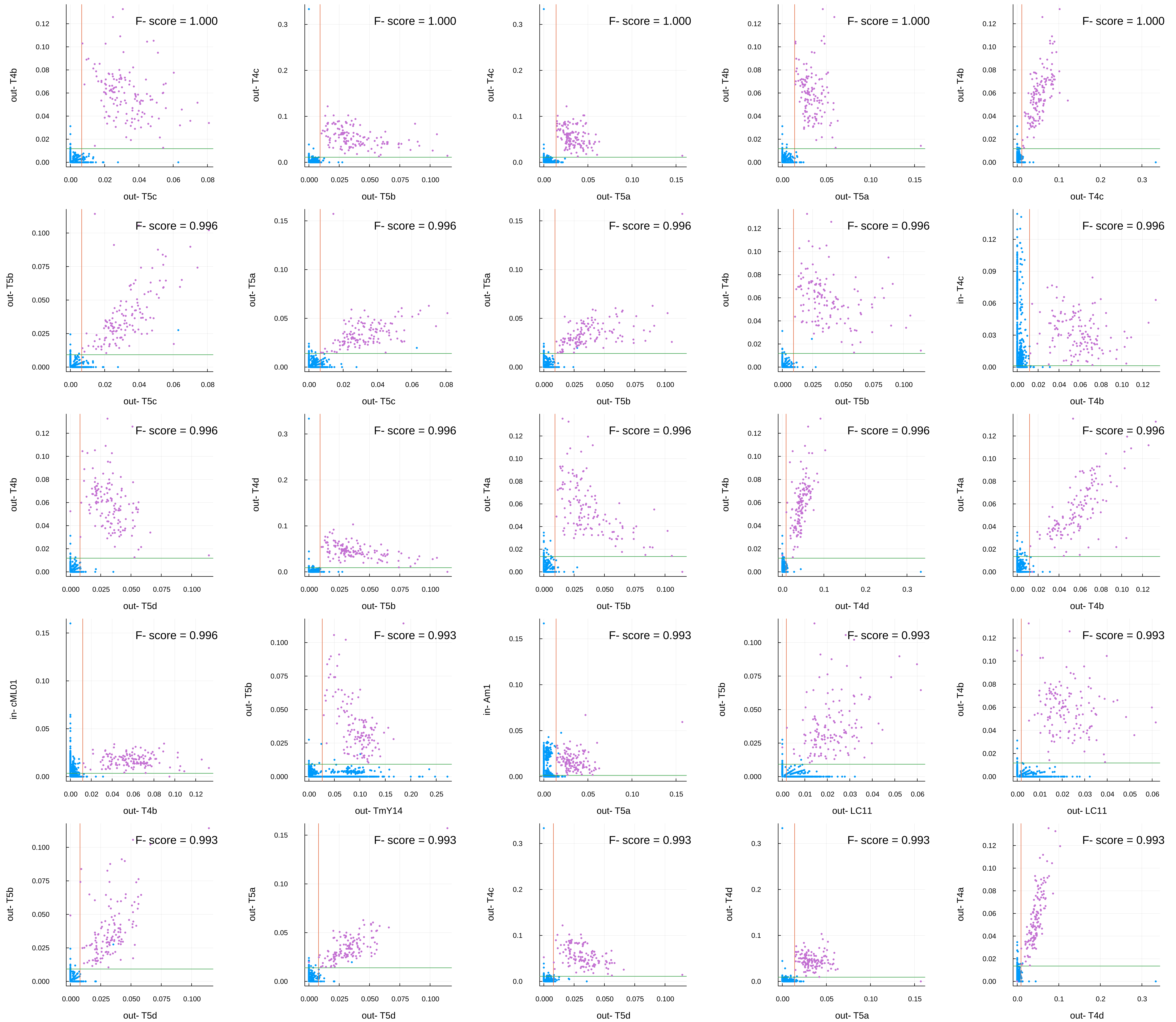

F- score = 1.000
out- T4b
out- T5c
F- score = 1.000
out- T4c
out- T5b
F- score = 1.000
out- T4c
out- T5a
F- score = 1.000
out- T4b
out- T5a
F- score = 1.000
out- T4b
out- T4c
F- score = 0.996
out- T5b
out- T5c
F- score = 0.996
out- T5a
out- T5c
F- score = 0.996
out- T5a
out- T5b
F- score = 0.996
out- T4b
out- T5b
F- score = 0.996
in- T4c
out- T4b
F- score = 0.996
out- T4b
out- T5d
F- score = 0.996
out- T4d
out- T5b
F- score = 0.996
out- T4a
out- T5b
F- score = 0.996
out- T4b
out- T4d
F- score = 0.996
out- T4a
out- T4b
F- score = 0.996
in- cML01
out- T4b
F- score = 0.993
out- T5b
out- TmY14
F- score = 0.993
in- Am1
out- T5a
F- score = 0.993
out- T5b
out- LC11
F- score = 0.993
out- T4b
out- LC11
F- score = 0.993
out- T5b
out- T5d
F- score = 0.993
out- T5a
out- T5d
F- score = 0.993
out- T4c
out- T5d
F- score = 0.993
out- T4d
out- T5a
F- score = 0.993
out- T4a
out- T4d
