# Supplementary material for: Neuronal parts list and wiring diagram for a visual system
Source: Nature. 2024 Oct 2;634(8032):166–80. doi: 10.1038/s41586-024-07981-1 (PMC11446827; doi:10.1038/s41586-024-07981-1)
Supplement: Supplementary file 7 — Discriminating 2D projections for neuropil-intrinsic types. For each interneuron type, a pair of features is shown that can be used to discriminate that type from others in the same neuropil. Many although not all discriminations are highly accurate. Both intrinsic and boundary types are included as discriminative features. [file 41586_2024_7981_MOESM7_ESM.zip › DataS3/TmY16.pdf]

TmY16

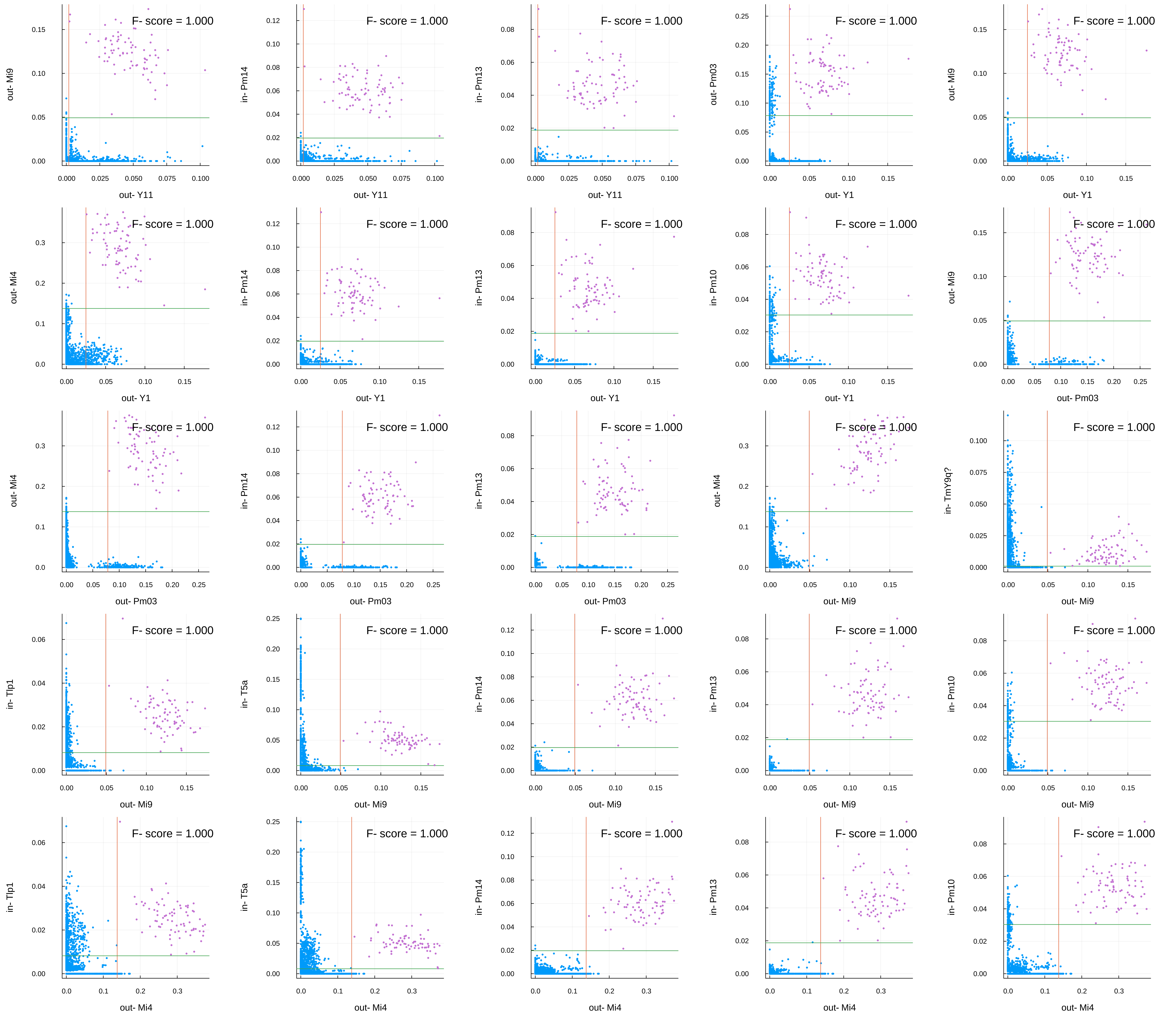
F- score = 1.000
out- Mi9
out- Y11
F- score = 1.000
in- Pm14
out- Y11
F- score = 1.000
in- Pm13
out- Y11
F- score = 1.000
out- Pm03
out- Y1
F- score = 1.000
out- Mi9
out- Y1
F- score = 1.000
out- Mi4
out- Y1
F- score = 1.000
in- Pm14
out- Y1
F- score = 1.000
in- Pm13
out- Y1
F- score = 1.000
in- Pm10
out- Y1
F- score = 1.000
out- Mi9
out- Pm03
F- score = 1.000
out- Mi4
out- Pm03
F- score = 1.000
in- Pm14
out- Pm03
F- score = 1.000
in- Pm13
out- Pm03
F- score = 1.000
out- Mi4
out- Mi9
F- score = 1.000
in- TmY9q?
out- Mi9
F- score = 1.000
in- Tlp1
out- Mi9
F- score = 1.000
in- T5a
out- Mi9
F- score = 1.000
in- Pm14
out- Mi9
F- score = 1.000
in- Pm13
out- Mi9
F- score = 1.000
in- Pm10
out- Mi9
F- score = 1.000
in- Tlp1
out- Mi4
F- score = 1.000
in- T5a
out- Mi4
F- score = 1.000
in- Pm14
out- Mi4
F- score = 1.000
in- Pm13
out- Mi4
F- score = 1.000
in- Pm10
out- Mi4
