# Supplementary material for: Neuronal parts list and wiring diagram for a visual system
Source: Nature. 2024 Oct 2;634(8032):166–80. doi: 10.1038/s41586-024-07981-1 (PMC11446827; doi:10.1038/s41586-024-07981-1)
Supplement: Supplementary file 7 — Discriminating 2D projections for neuropil-intrinsic types. For each interneuron type, a pair of features is shown that can be used to discriminate that type from others in the same neuropil. Many although not all discriminations are highly accurate. Both intrinsic and boundary types are included as discriminative features. [file 41586_2024_7981_MOESM7_ESM.zip › DataS3/TmY20.pdf]

TmY20

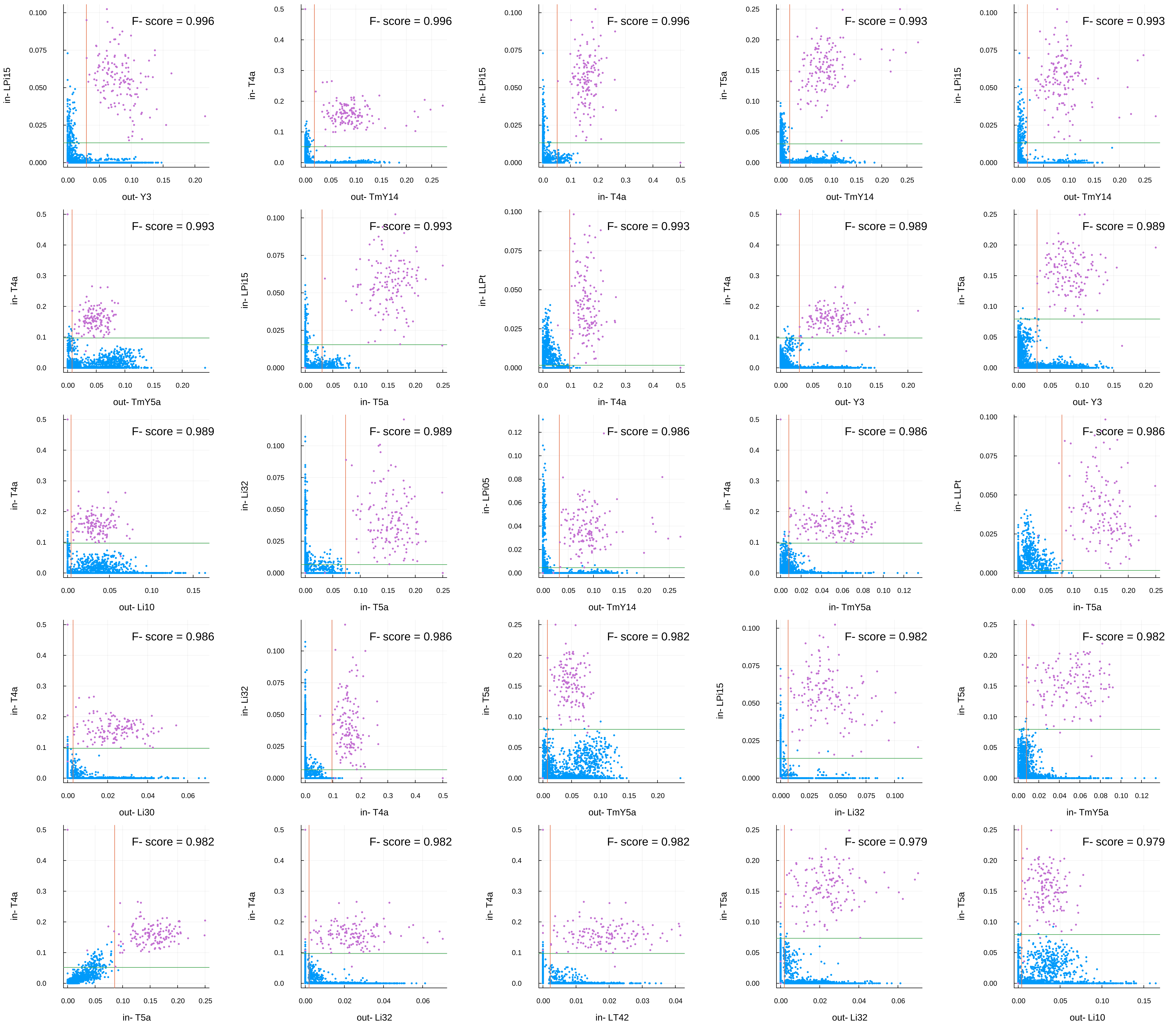

F- score = 0.996
in- LPi15
out- Y3
F- score = 0.996
in- T4a
out- TmY14
F- score = 0.996
in- LPi15
in- T4a
F- score = 0.993
in- T5a
out- TmY14
F- score = 0.993
in- LPi15
out- TmY14
F- score = 0.993
in- T4a
out- TmY5a
F- score = 0.993
in- LPi15
in- T5a
F- score = 0.993
in- LLPt
in- T4a
F- score = 0.989
in- T4a
out- Y3
F- score = 0.989
in- T5a
out- Y3
F- score = 0.989
in- T4a
out- Li10
F- score = 0.989
in- Li32
in- T5a
F- score = 0.986
in- LPi05
out- TmY14
F- score = 0.986
in- T4a
in- TmY5a
F- score = 0.986
in- LLPt
in- T5a
F- score = 0.986
in- T4a
out- Li30
F- score = 0.986
in- Li32
in- T4a
F- score = 0.982
in- T5a
out- TmY5a
F- score = 0.982
in- LPi15
in- Li32
F- score = 0.982
in- T5a
in- TmY5a
F- score = 0.982
in- T4a
in- T5a
F- score = 0.982
in- T4a
out- Li32
F- score = 0.982
in- T4a
in- LT42
F- score = 0.979
in- T5a
out- Li32
F- score = 0.979
in- T5a
out- Li10
