# Supplementary material for: Neuronal parts list and wiring diagram for a visual system
Source: Nature. 2024 Oct 2;634(8032):166–80. doi: 10.1038/s41586-024-07981-1 (PMC11446827; doi:10.1038/s41586-024-07981-1)
Supplement: Supplementary file 7 — Discriminating 2D projections for neuropil-intrinsic types. For each interneuron type, a pair of features is shown that can be used to discriminate that type from others in the same neuropil. Many although not all discriminations are highly accurate. Both intrinsic and boundary types are included as discriminative features. [file 41586_2024_7981_MOESM7_ESM.zip › DataS3/TmY3.pdf]

TmY3

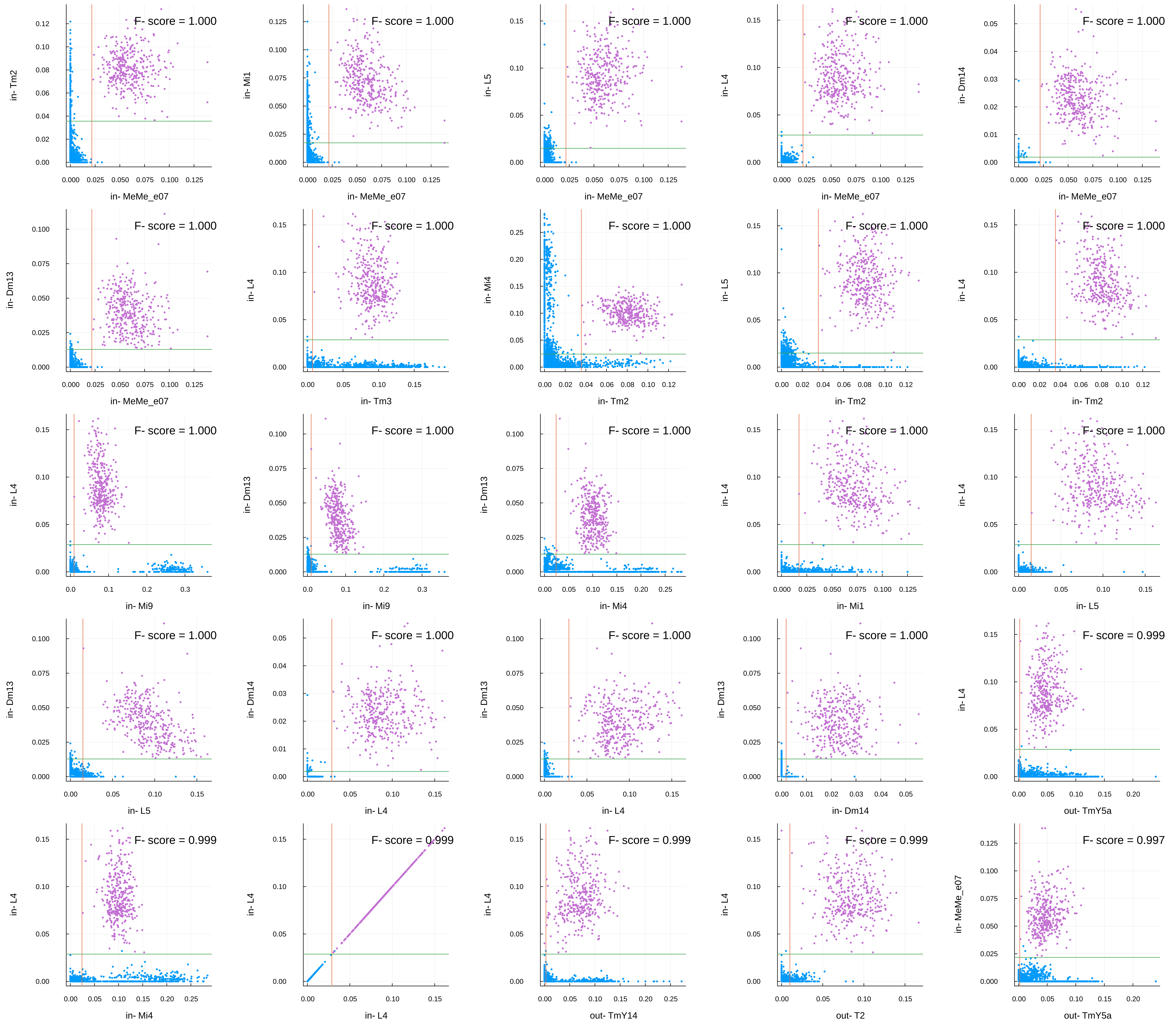
F- score = 1.000
in- Tm2
in- MeMe_e07
F- score = 1.000
in- Mi1
in- MeMe_e07
F- score = 1.000
in- L5
in- MeMe_e07
F- score = 1.000
in- L4
in- MeMe_e07
F- score = 1.000
in- Dm14
in- MeMe_e07
F- score = 1.000
in- Dm13
in- MeMe_e07
F- score = 1.000
in- L4
in- Tm3
F- score = 1.000
in- Mi4
in- Tm2
F- score = 1.000
in- L5
in- Tm2
F- score = 1.000
in- L4
in- Tm2
F- score = 1.000
in- L4
in- Mi9
F- score = 1.000
in- Dm13
in- Mi9
F- score = 1.000
in- Dm13
in- Mi4
F- score = 1.000
in- L4
in- Mi1
F- score = 1.000
in- L4
in- L5
F- score = 1.000
in- Dm13
in- L5
F- score = 1.000
in- Dm14
in- L4
F- score = 1.000
in- Dm13
in- L4
F- score = 1.000
in- Dm13
in- Dm14
F- score = 0.999
in- L4
out- TmY5a
F- score = 0.999
in- L4
in- Mi4
F- score = 0.999
in- L4
in- L4
F- score = 0.999
in- L4
out- TmY14
F- score = 0.999
in- L4
out- T2
F- score = 0.997
in- MeMe_e07
out- TmY5a
