# Supplementary material for: Neuronal parts list and wiring diagram for a visual system
Source: Nature. 2024 Oct 2;634(8032):166–80. doi: 10.1038/s41586-024-07981-1 (PMC11446827; doi:10.1038/s41586-024-07981-1)
Supplement: Supplementary file 7 — Discriminating 2D projections for neuropil-intrinsic types. For each interneuron type, a pair of features is shown that can be used to discriminate that type from others in the same neuropil. Many although not all discriminations are highly accurate. Both intrinsic and boundary types are included as discriminative features. [file 41586_2024_7981_MOESM7_ESM.zip › DataS3/TmY31.pdf]

TmY31

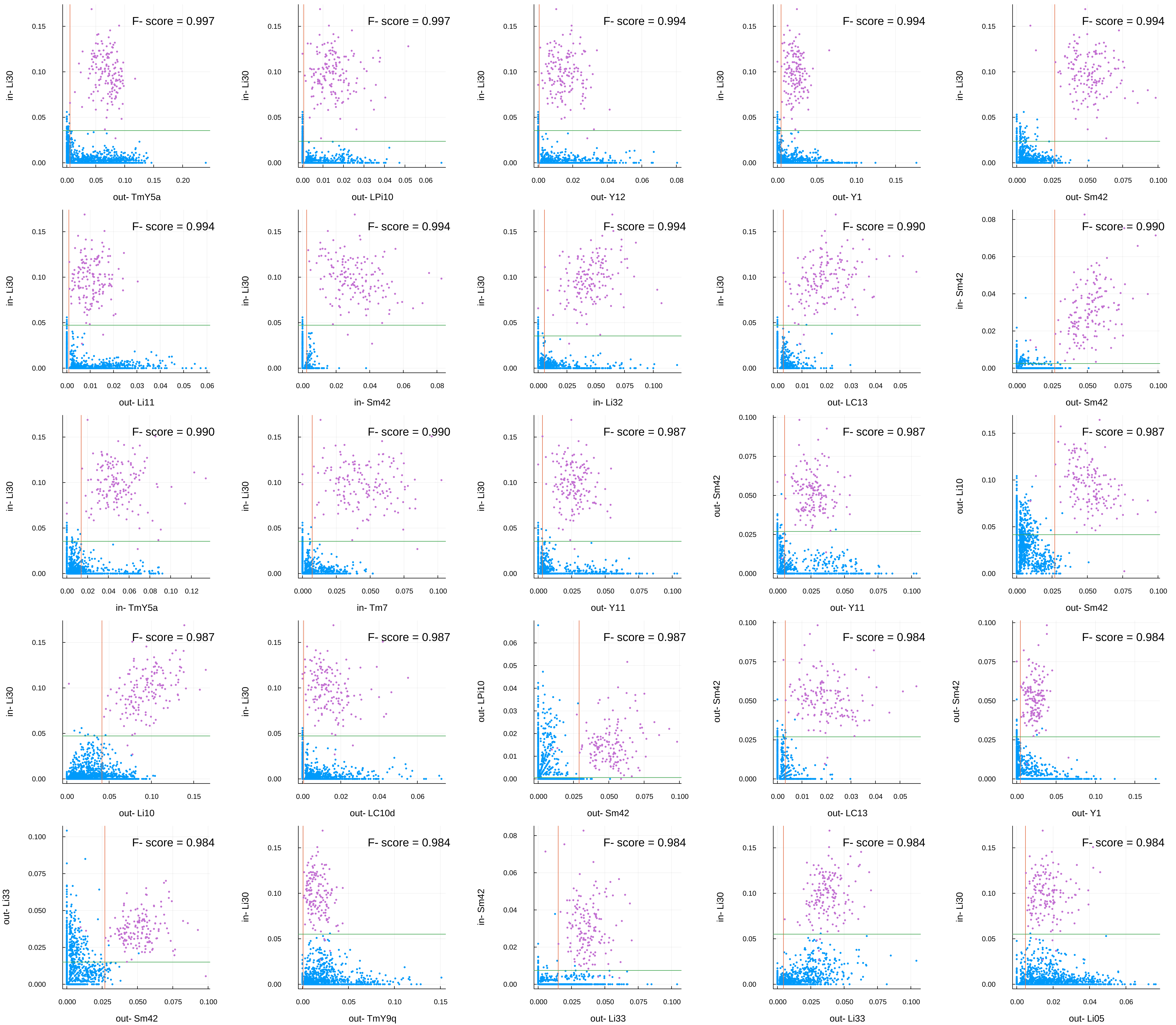

F- score = 0.997
in- Li30
out- TmY5a
F- score = 0.997
in- Li30
out- LPi10
F- score = 0.994
in- Li30
out- Y12
F- score = 0.994
in- Li30
out- Y1
F- score = 0.994
in- Li30
out- Sm42
F- score = 0.994
in- Li30
out- Li11
F- score = 0.994
in- Li30
in- Sm42
F- score = 0.994
in- Li30
in- Li32
F- score = 0.990
in- Li30
out- LC13
F- score = 0.990
in- Sm42
out- Sm42
F- score = 0.990
in- Li30
in- TmY5a
F- score = 0.990
in- Li30
in- Tm7
F- score = 0.987
in- Li30
out- Y11
F- score = 0.987
out- Sm42
out- Y11
F- score = 0.987
out- Li10
out- Sm42
F- score = 0.987
in- Li30
out- Li10
F- score = 0.987
in- Li30
out- LC10d
F- score = 0.987
out- LPi10
out- Sm42
F- score = 0.984
out- Sm42
out- LC13
F- score = 0.984
out- Sm42
out- Y1
F- score = 0.984
out- Li33
out- Sm42
F- score = 0.984
in- Li30
out- TmY9q
F- score = 0.984
in- Sm42
out- Li33
F- score = 0.984
in- Li30
out- Li33
F- score = 0.984
in- Li30
out- Li05
