# Supplementary material for: Neuronal parts list and wiring diagram for a visual system
Source: Nature. 2024 Oct 2;634(8032):166–80. doi: 10.1038/s41586-024-07981-1 (PMC11446827; doi:10.1038/s41586-024-07981-1)
Supplement: Supplementary file 7 — Discriminating 2D projections for neuropil-intrinsic types. For each interneuron type, a pair of features is shown that can be used to discriminate that type from others in the same neuropil. Many although not all discriminations are highly accurate. Both intrinsic and boundary types are included as discriminative features. [file 41586_2024_7981_MOESM7_ESM.zip › DataS3/TmY4.pdf]

TmY4

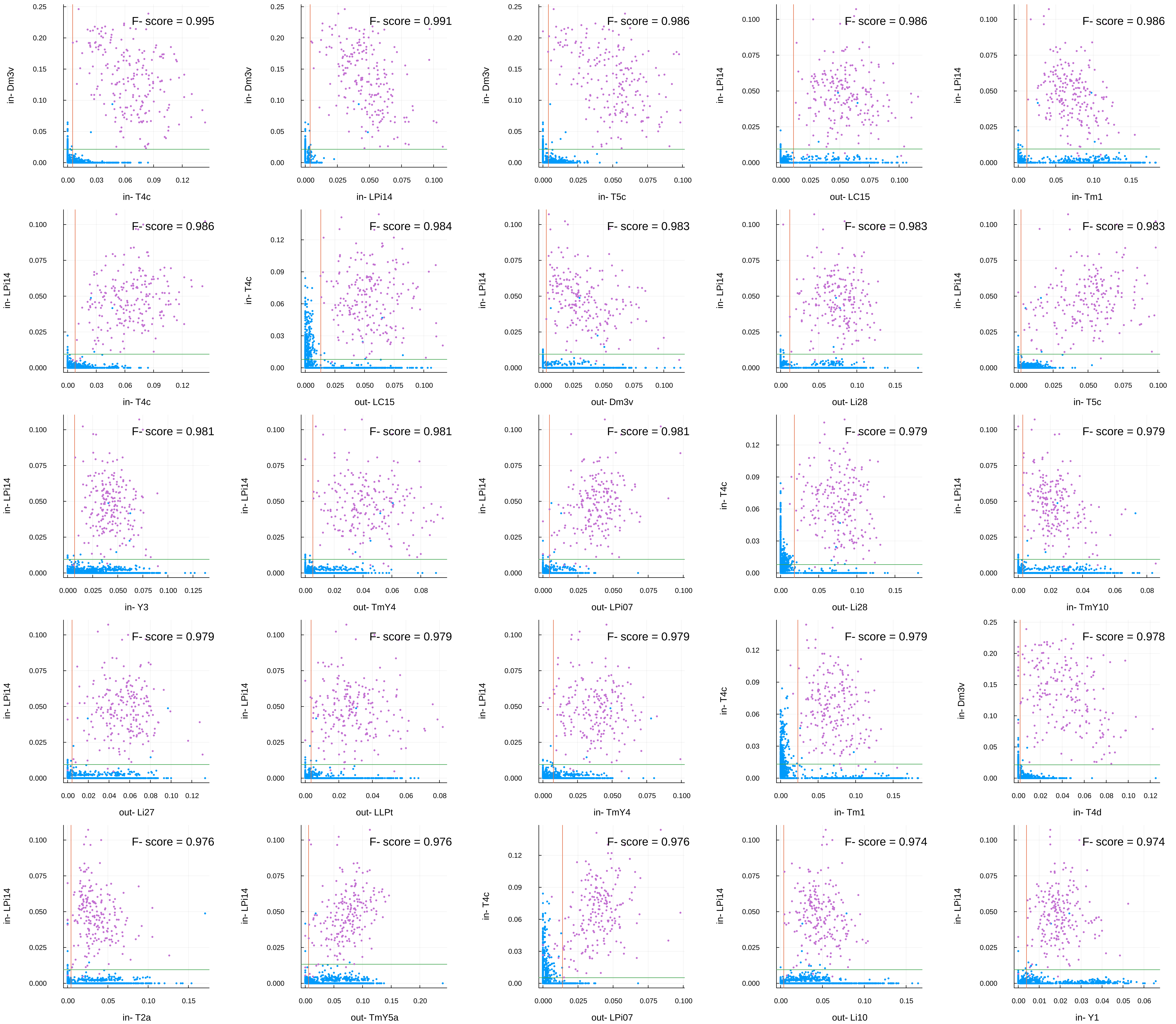

F- score = 0.995
in- Dm3v
in- T4c
F- score = 0.991
in- Dm3v
in- LPi14
F- score = 0.986
in- Dm3v
in- T5c
F- score = 0.986
in- LPi14
out- LC15
F- score = 0.986
in- LPi14
in- Tm1
F- score = 0.986
in- LPi14
in- T4c
F- score = 0.984
in- T4c
out- LC15
F- score = 0.983
in- LPi14
out- Dm3v
F- score = 0.983
in- LPi14
out- Li28
F- score = 0.983
in- LPi14
in- T5c
F- score = 0.981
in- LPi14
in- Y3
F- score = 0.981
in- LPi14
out- TmY4
F- score = 0.981
in- LPi14
out- LPi07
F- score = 0.979
in- T4c
out- Li28
F- score = 0.979
in- LPi14
in- TmY10
F- score = 0.979
in- LPi14
out- Li27
F- score = 0.979
in- LPi14
out- LLPt
F- score = 0.979
in- LPi14
in- TmY4
F- score = 0.979
in- T4c
in- Tm1
F- score = 0.978
in- Dm3v
in- T4d
F- score = 0.976
in- LPi14
in- T2a
F- score = 0.976
in- LPi14
out- TmY5a
F- score = 0.976
in- T4c
out- LPi07
F- score = 0.974
in- LPi14
out- Li10
F- score = 0.974
in- LPi14
in- Y1
