# Supplementary material for: Neuronal parts list and wiring diagram for a visual system
Source: Nature. 2024 Oct 2;634(8032):166–80. doi: 10.1038/s41586-024-07981-1 (PMC11446827; doi:10.1038/s41586-024-07981-1)
Supplement: Supplementary file 7 — Discriminating 2D projections for neuropil-intrinsic types. For each interneuron type, a pair of features is shown that can be used to discriminate that type from others in the same neuropil. Many although not all discriminations are highly accurate. Both intrinsic and boundary types are included as discriminative features. [file 41586_2024_7981_MOESM7_ESM.zip › DataS3/TmY5a.pdf]

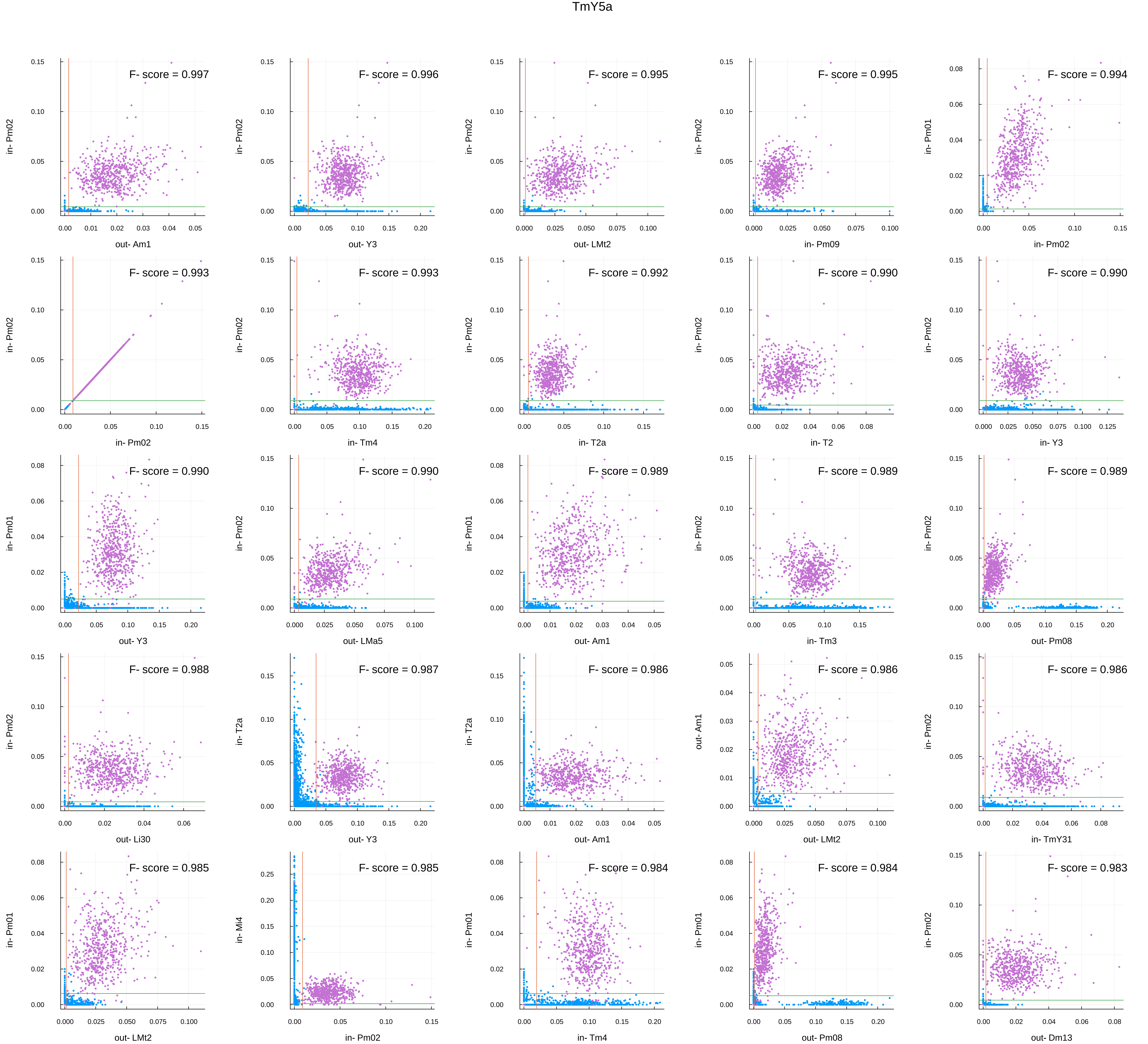
TmY5a
F- score = 0.997
in- Pm02
out- Am1
F- score = 0.996
out- Y3
F- score = 0.995
out- LMt2
F- score = 0.995
in- Pm09
F- score = 0.994
in- Pm01
in- Pm02
F- score = 0.993
in- Pm02
F- score = 0.993
in- Tm4
F- score = 0.992
in- T2a
F- score = 0.990
in- T2
F- score = 0.990
in- Y3
F- score = 0.990
in- Pm01
out- Y3
F- score = 0.990
out- LMa5
F- score = 0.989
out- Am1
F- score = 0.989
in- Tm3
F- score = 0.989
out- Pm08
F- score = 0.988
out- Li30
F- score = 0.987
in- T2a
out- Y3
F- score = 0.986
out- Am1
F- score = 0.986
out- Am1
out- LMt2
F- score = 0.986
in- TmY31
F- score = 0.985
out- LMt2
F- score = 0.985
in- Mi4
in- Pm02
F- score = 0.984
in- Tm4
F- score = 0.984
out- Pm08
F- score = 0.983
out- Dm13
