# Supplementary material for: Neuronal parts list and wiring diagram for a visual system
Source: Nature. 2024 Oct 2;634(8032):166–80. doi: 10.1038/s41586-024-07981-1 (PMC11446827; doi:10.1038/s41586-024-07981-1)
Supplement: Supplementary file 7 — Discriminating 2D projections for neuropil-intrinsic types. For each interneuron type, a pair of features is shown that can be used to discriminate that type from others in the same neuropil. Many although not all discriminations are highly accurate. Both intrinsic and boundary types are included as discriminative features. [file 41586_2024_7981_MOESM7_ESM.zip › DataS3/TmY9q.pdf]

TmY9q

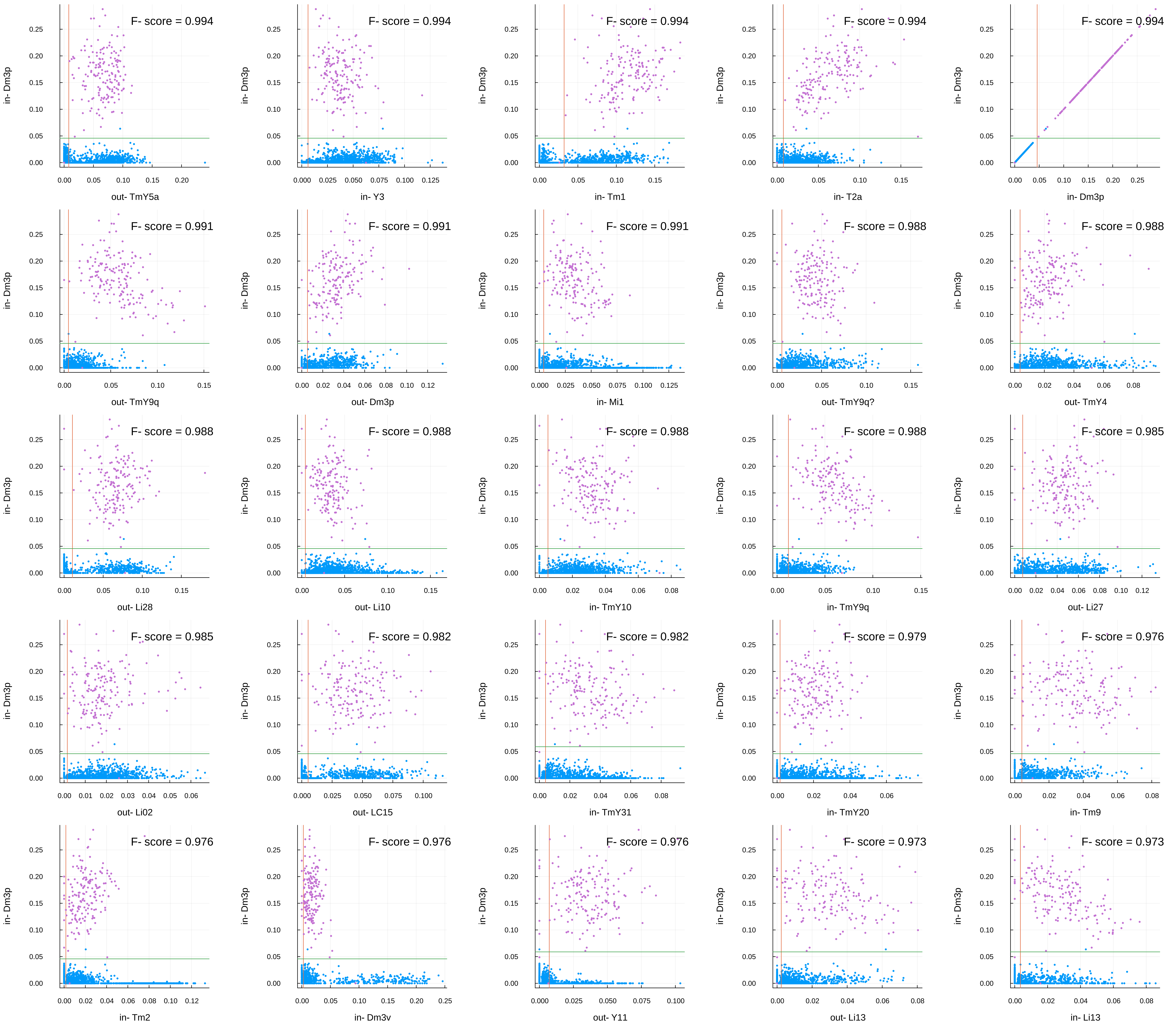

F- score = 0.994
in- Dm3p
out- TmY5a
F- score = 0.994
in- Y3
F- score = 0.994
in- Tm1
F- score = 0.994
in- T2a
F- score = 0.994
in- Dm3p
F- score = 0.991
out- TmY9q
F- score = 0.991
out- Dm3p
F- score = 0.991
in- Mi1
F- score = 0.988
out- TmY9q?
F- score = 0.988
out- TmY4
F- score = 0.988
out- Li28
F- score = 0.988
out- Li10
F- score = 0.988
in- TmY10
F- score = 0.988
in- TmY9q
F- score = 0.985
out- Li27
F- score = 0.985
out- Li02
F- score = 0.982
out- LC15
F- score = 0.982
in- TmY31
F- score = 0.979
in- TmY20
F- score = 0.976
in- Tm9
F- score = 0.976
in- Tm2
F- score = 0.976
in- Dm3v
F- score = 0.976
out- Y11
F- score = 0.973
out- Li13
F- score = 0.973
in- Li13
