# Supplementary material for: Neuronal parts list and wiring diagram for a visual system
Source: Nature. 2024 Oct 2;634(8032):166–80. doi: 10.1038/s41586-024-07981-1 (PMC11446827; doi:10.1038/s41586-024-07981-1)
Supplement: Supplementary file 7 — Discriminating 2D projections for neuropil-intrinsic types. For each interneuron type, a pair of features is shown that can be used to discriminate that type from others in the same neuropil. Many although not all discriminations are highly accurate. Both intrinsic and boundary types are included as discriminative features. [file 41586_2024_7981_MOESM7_ESM.zip › DataS3/TmY9qΓèÑ.pdf]

TmY9q?

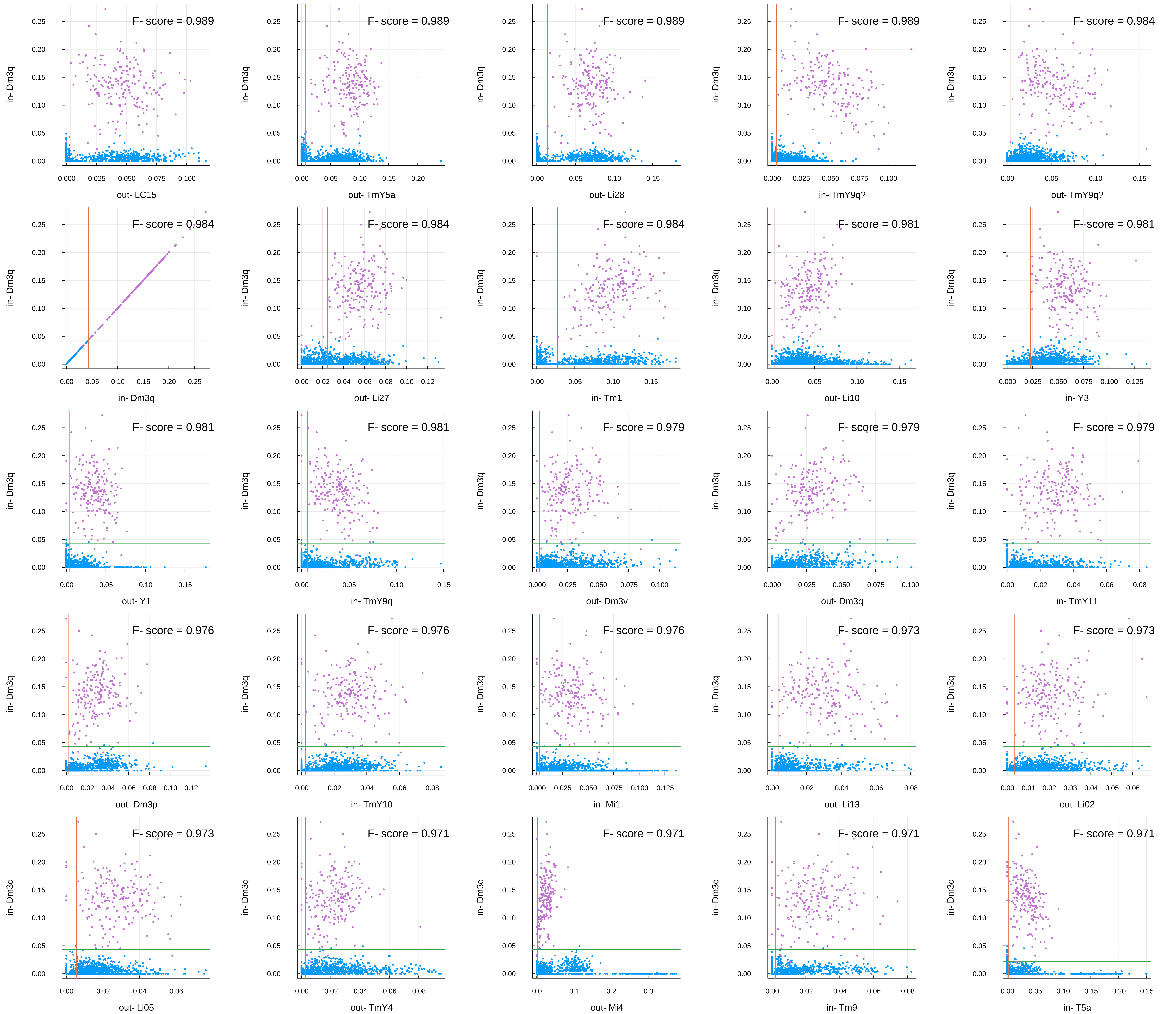
F- score = 0.989
in- Dm3q
out- LC15
F- score = 0.989
in- Dm3q
out- TmY5a
F- score = 0.989
in- Dm3q
out- Li28
F- score = 0.989
in- Dm3q
in- TmY9q?
F- score = 0.984
in- Dm3q
out- TmY9q?
F- score = 0.984
in- Dm3q
in- Dm3q
F- score = 0.984
in- Dm3q
out- Li27
F- score = 0.984
in- Dm3q
in- Tm1
F- score = 0.981
in- Dm3q
out- Li10
F- score = 0.981
in- Dm3q
in- Y3
F- score = 0.981
in- Dm3q
out- Y1
F- score = 0.981
in- Dm3q
in- TmY9q
F- score = 0.979
in- Dm3q
out- Dm3v
F- score = 0.979
in- Dm3q
out- Dm3q
F- score = 0.979
in- Dm3q
in- TmY11
F- score = 0.976
in- Dm3q
out- Dm3p
F- score = 0.976
in- Dm3q
in- TmY10
F- score = 0.976
in- Dm3q
in- Mi1
F- score = 0.973
in- Dm3q
out- Li13
F- score = 0.973
in- Dm3q
out- Li02
F- score = 0.973
in- Dm3q
out- Li05
F- score = 0.971
in- Dm3q
out- TmY4
F- score = 0.971
in- Dm3q
out- Mi4
F- score = 0.971
in- Dm3q
in- Tm9
F- score = 0.971
in- Dm3q
in- T5a
