# Supplementary material for: Neuronal parts list and wiring diagram for a visual system
Source: Nature. 2024 Oct 2;634(8032):166–80. doi: 10.1038/s41586-024-07981-1 (PMC11446827; doi:10.1038/s41586-024-07981-1)
Supplement: Supplementary file 7 — Discriminating 2D projections for neuropil-intrinsic types. For each interneuron type, a pair of features is shown that can be used to discriminate that type from others in the same neuropil. Many although not all discriminations are highly accurate. Both intrinsic and boundary types are included as discriminative features. [file 41586_2024_7981_MOESM7_ESM.zip › DataS3/Y1.pdf]

Y1

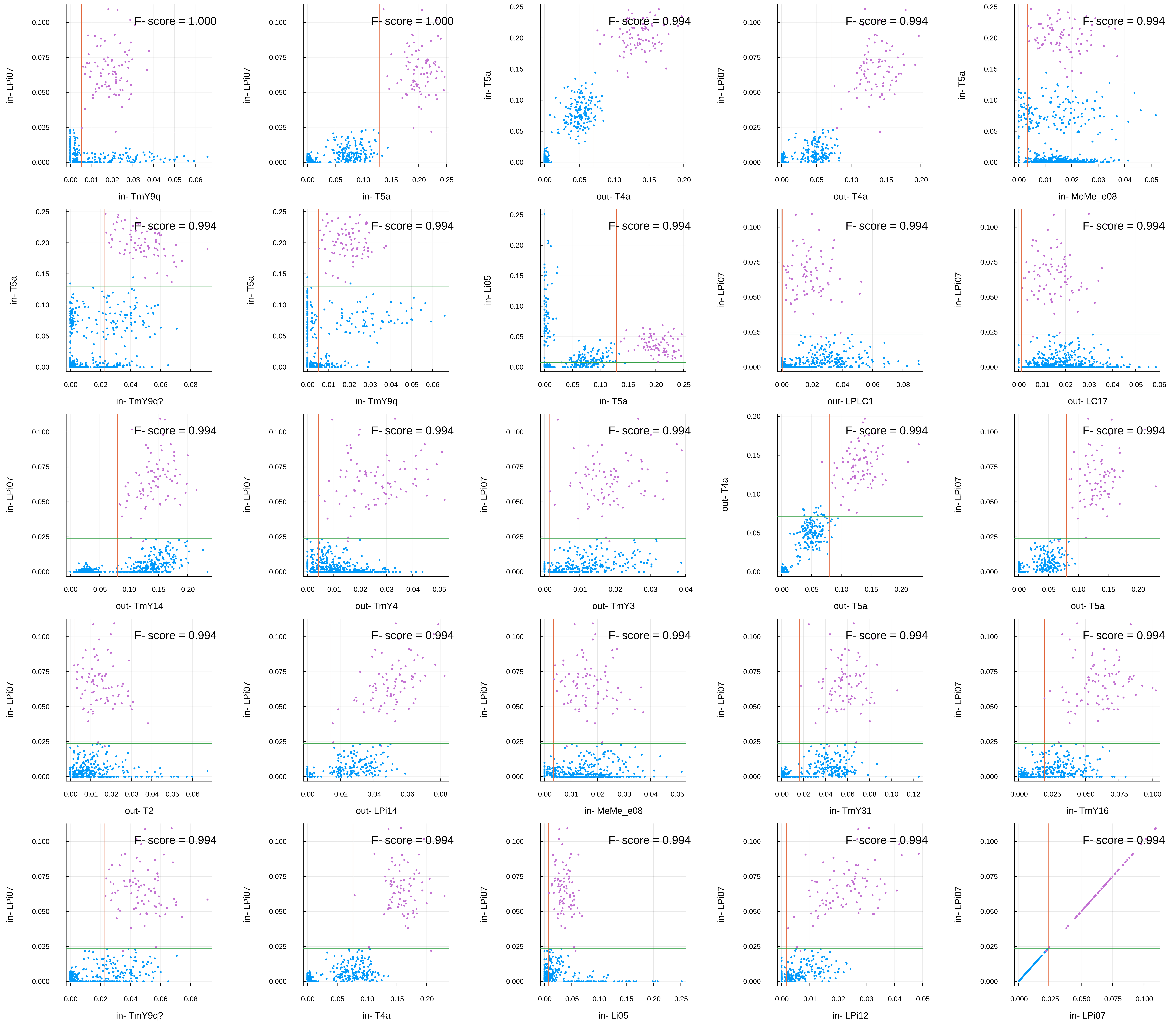
F- score = 1.000
in- LPi07
in- TmY9q
F- score = 1.000
in- LPi07
in- T5a
F- score = 0.994
in- T5a
out- T4a
F- score = 0.994
in- LPi07
out- T4a
F- score = 0.994
in- T5a
in- MeMe_e08
F- score = 0.994
in- T5a
in- TmY9q?
F- score = 0.994
in- T5a
in- TmY9q
F- score = 0.994
in- Li05
in- T5a
F- score = 0.994
in- LPi07
out- LPLC1
F- score = 0.994
in- LPi07
out- LC17
F- score = 0.994
in- LPi07
out- TmY14
F- score = 0.994
in- LPi07
out- TmY4
F- score = 0.994
in- LPi07
out- TmY3
F- score = 0.994
out- T4a
out- T5a
F- score = 0.994
in- LPi07
out- T5a
F- score = 0.994
in- LPi07
out- T2
F- score = 0.994
in- LPi07
out- LPi14
F- score = 0.994
in- LPi07
in- MeMe_e08
F- score = 0.994
in- LPi07
in- TmY31
F- score = 0.994
in- LPi07
in- TmY16
F- score = 0.994
in- LPi07
in- TmY9q?
F- score = 0.994
in- LPi07
in- T4a
F- score = 0.994
in- LPi07
in- Li05
F- score = 0.994
in- LPi07
in- LPi12
F- score = 0.994
in- LPi07
in- LPi07
