# Supplementary material for: Neuronal parts list and wiring diagram for a visual system
Source: Nature. 2024 Oct 2;634(8032):166–80. doi: 10.1038/s41586-024-07981-1 (PMC11446827; doi:10.1038/s41586-024-07981-1)
Supplement: Supplementary file 7 — Discriminating 2D projections for neuropil-intrinsic types. For each interneuron type, a pair of features is shown that can be used to discriminate that type from others in the same neuropil. Many although not all discriminations are highly accurate. Both intrinsic and boundary types are included as discriminative features. [file 41586_2024_7981_MOESM7_ESM.zip › DataS3/Y12.pdf]

Y12

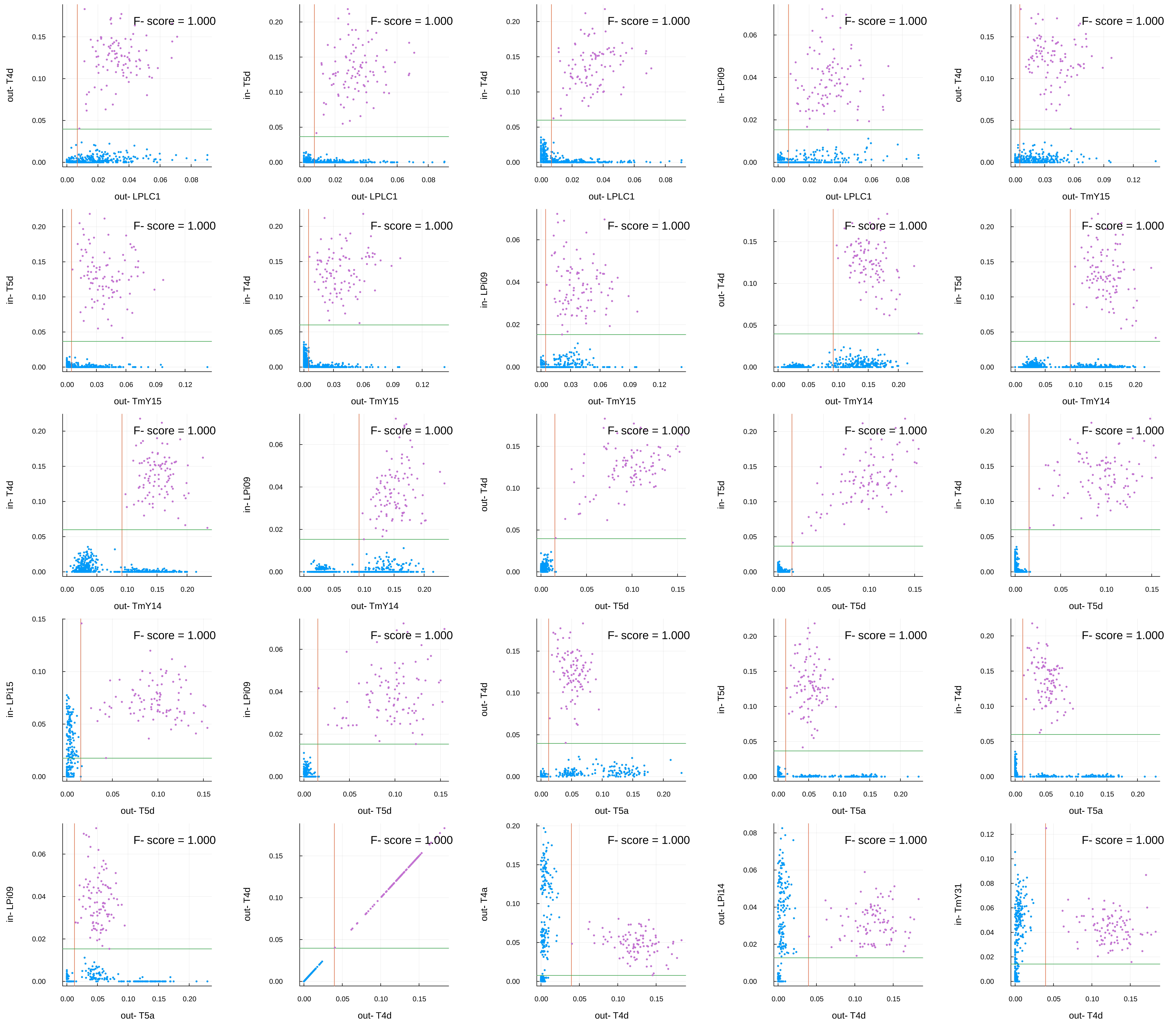

F- score = 1.000
out- T4d
out- LPLC1
in- T5d
out- LPLC1
in- T4d
out- LPLC1
in- LPi09
out- LPLC1
out- T4d
out- TmY15
in- T5d
out- TmY15
in- T4d
out- TmY15
in- LPi09
out- TmY15
out- T4d
out- TmY14
in- T5d
out- TmY14
in- T4d
out- TmY14
in- LPi09
out- TmY14
out- T4d
out- T5d
in- T5d
out- T5d
in- T4d
out- T5d
in- LPi15
out- T5d
in- LPi09
out- T5d
out- T4d
out- T5a
in- T5d
out- T5a
in- T4d
out- T5a
in- LPi09
out- T5a
out- T4d
out- T4d
out- T4a
out- T4d
out- LPi14
out- T4d
in- TmY31
out- T4d
