# Supplementary material for: Neuronal parts list and wiring diagram for a visual system
Source: Nature. 2024 Oct 2;634(8032):166–80. doi: 10.1038/s41586-024-07981-1 (PMC11446827; doi:10.1038/s41586-024-07981-1)
Supplement: Supplementary file 7 — Discriminating 2D projections for neuropil-intrinsic types. For each interneuron type, a pair of features is shown that can be used to discriminate that type from others in the same neuropil. Many although not all discriminations are highly accurate. Both intrinsic and boundary types are included as discriminative features. [file 41586_2024_7981_MOESM7_ESM.zip › DataS3/Y3.pdf]

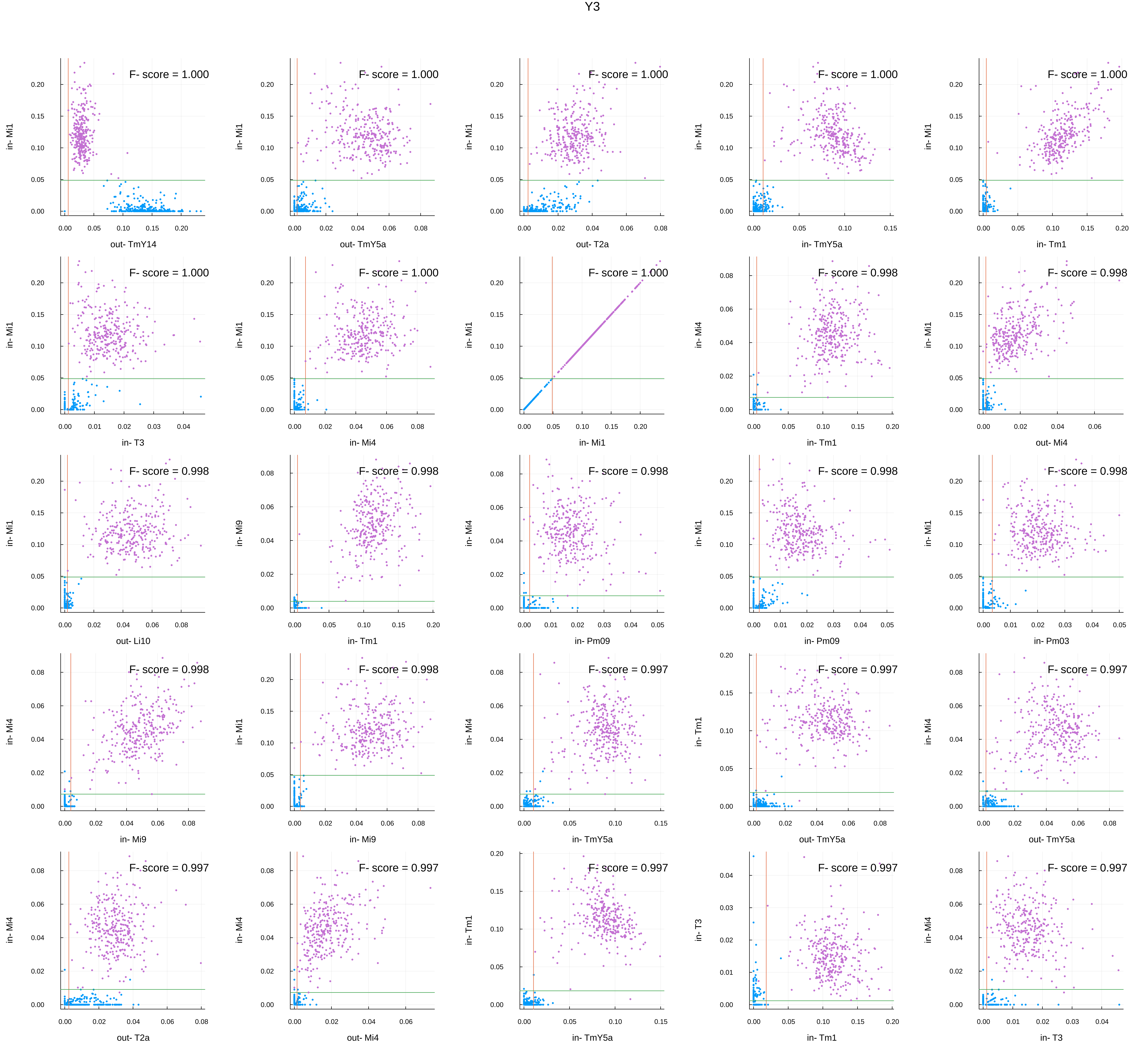
Y3
F- score = 1.000
in- Mi1
out- TmY14
F- score = 1.000
in- Mi1
out- TmY5a
F- score = 1.000
in- Mi1
out- T2a
F- score = 1.000
in- Mi1
in- TmY5a
F- score = 1.000
in- Mi1
in- Tm1
F- score = 1.000
in- Mi1
in- T3
F- score = 1.000
in- Mi1
in- Mi4
F- score = 1.000
in- Mi1
in- Mi1
F- score = 0.998
in- Mi4
in- Tm1
F- score = 0.998
in- Mi1
out- Mi4
F- score = 0.998
in- Mi1
out- Li10
F- score = 0.998
in- Mi9
in- Tm1
F- score = 0.998
in- Mi4
in- Pm09
F- score = 0.998
in- Mi1
in- Pm09
F- score = 0.998
in- Mi1
in- Pm03
F- score = 0.998
in- Mi4
in- Mi9
F- score = 0.998
in- Mi1
in- Mi9
F- score = 0.997
in- Mi4
in- TmY5a
F- score = 0.997
in- Tm1
out- TmY5a
F- score = 0.997
in- Mi4
out- TmY5a
F- score = 0.997
in- Mi4
out- T2a
F- score = 0.997
in- Mi4
out- Mi4
F- score = 0.997
in- Tm1
in- TmY5a
F- score = 0.997
in- T3
in- Tm1
F- score = 0.997
in- Mi4
in- T3
