# Supplementary material for: Neuronal parts list and wiring diagram for a visual system
Source: Nature. 2024 Oct 2;634(8032):166–80. doi: 10.1038/s41586-024-07981-1 (PMC11446827; doi:10.1038/s41586-024-07981-1)
Supplement: Supplementary file 7 — Discriminating 2D projections for neuropil-intrinsic types. For each interneuron type, a pair of features is shown that can be used to discriminate that type from others in the same neuropil. Many although not all discriminations are highly accurate. Both intrinsic and boundary types are included as discriminative features. [file 41586_2024_7981_MOESM7_ESM.zip › DataS3/Y4.pdf]

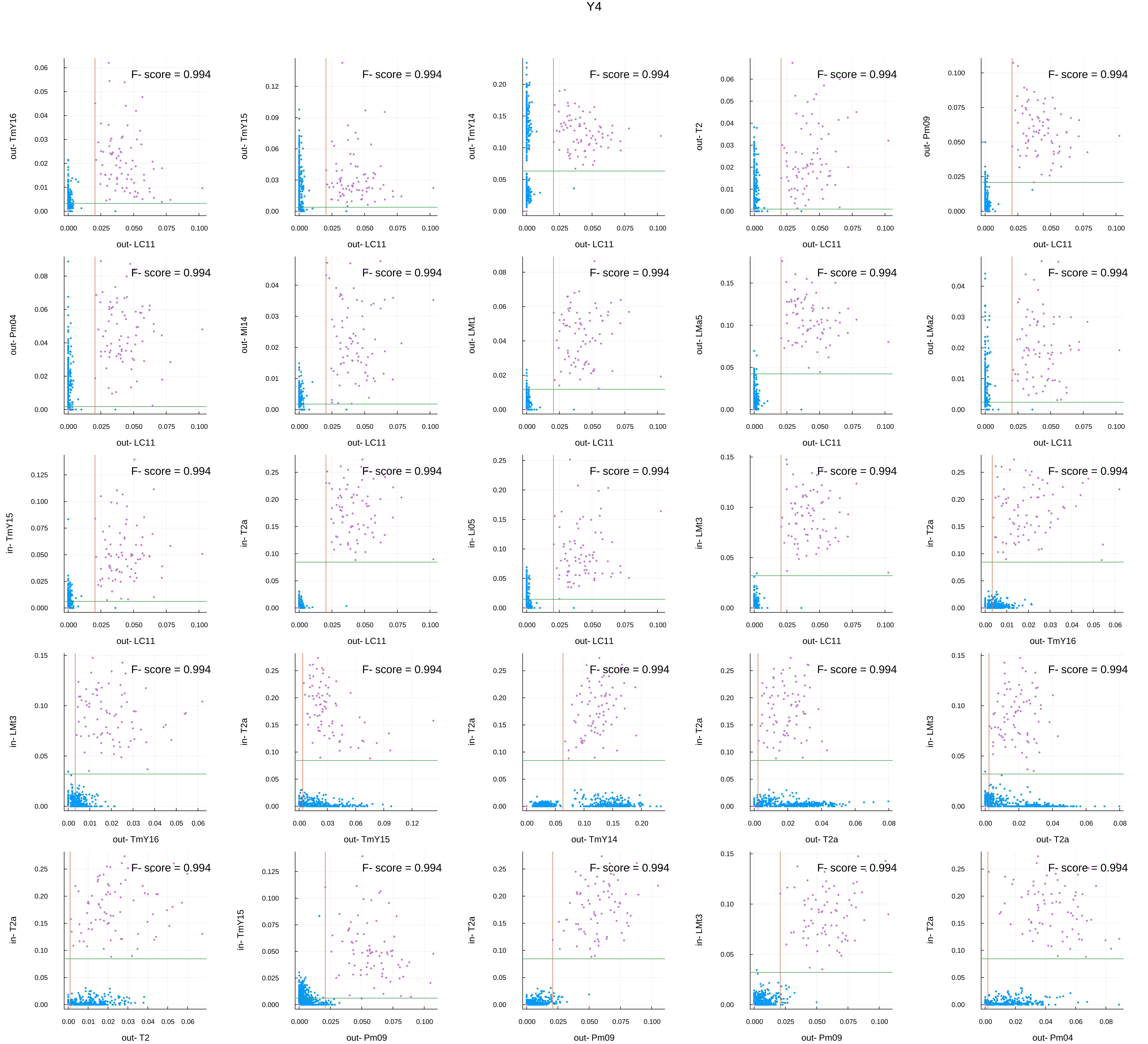
Y4
F- score = 0.994
out- TmY16
out- LC11
F- score = 0.994
out- TmY15
out- LC11
F- score = 0.994
out- TmY14
out- LC11
F- score = 0.994
out- T2
out- LC11
F- score = 0.994
out- Pm09
out- LC11
F- score = 0.994
out- Pm04
out- LC11
F- score = 0.994
out- Mi14
out- LC11
F- score = 0.994
out- LMt1
out- LC11
F- score = 0.994
out- LMa5
out- LC11
F- score = 0.994
out- LMa2
out- LC11
F- score = 0.994
in- TmY15
out- LC11
F- score = 0.994
in- T2a
out- LC11
F- score = 0.994
in- Li05
out- LC11
F- score = 0.994
in- LMt3
out- LC11
F- score = 0.994
in- T2a
out- TmY16
F- score = 0.994
in- LMt3
out- TmY16
F- score = 0.994
in- T2a
out- TmY15
F- score = 0.994
in- T2a
out- TmY14
F- score = 0.994
in- T2a
out- T2a
F- score = 0.994
in- LMt3
out- T2a
F- score = 0.994
in- T2a
out- T2
F- score = 0.994
in- TmY15
out- Pm09
F- score = 0.994
in- T2a
out- Pm09
F- score = 0.994
in- LMt3
out- Pm09
F- score = 0.994
in- T2a
out- Pm04
